# Copper-catalyzed diastereoselective aerobic intramolecular dehydrogenative coupling of hydrazones *via* sp^3^ C–H functionalization†
†Electronic supplementary information (ESI) available: Experimental details including characterization data, copies of ^1^H, ^13^C NMR and NOESY spectra. See DOI: 10.1039/c5sc01736j


**DOI:** 10.1039/c5sc01736j

**Published:** 2015-07-14

**Authors:** Xuesong Wu, Mian Wang, Guangwu Zhang, Yan Zhao, Jianyi Wang, Haibo Ge

**Affiliations:** a Department of Chemistry and Chemical Biology , Indiana University-Purdue University Indianapolis , Indianapolis , Indiana 46202 , USA . Email: geh@iupui.edu; b School of Chemistry and Chemical Engineering , Guangxi University , Nanning 530004 , Guangxi , P. R. China . Email: jianyiwang@gxu.edu.cn

## Abstract

Transition metal-catalyzed cross dehydrogenative coupling is an important tool for functionalization of the α Csp^3^–H bond of amines. Among this reaction category, copper-catalyzed selective C–C bond formation under atmospheric O_2_ is of considerable research interest and significant progress has been achieved in recent years. In comparison, development of the intramolecular version of this transformation is still in its infancy. Furthermore, diastereoselective cyclization with this transformation has not been achieved. Here, we describe the highly diastereoselective intramolecular dehydrogenative cyclization of *N*,*N*-disubstituted hydrazones by a copper-catalyzed sp^3^ C–H bond functionalization process. The reaction protocol utilizes O_2_ as the oxidant and shows great functional group compatibility. Computational studies suggest that a 5-center/6-electron disrotatory cyclization mechanism is probably involved in the process for controlling the diastereoselectivity. This work represents the first example of a copper-catalyzed, direct intramolecular diastereoselective coupling reaction *via* an iminium ion intermediate. Additionally, it provides an environmentally friendly and atom efficient approach to access substituted pyrazolines, an important structural unit in many biologically active compounds.

## Abstract 2

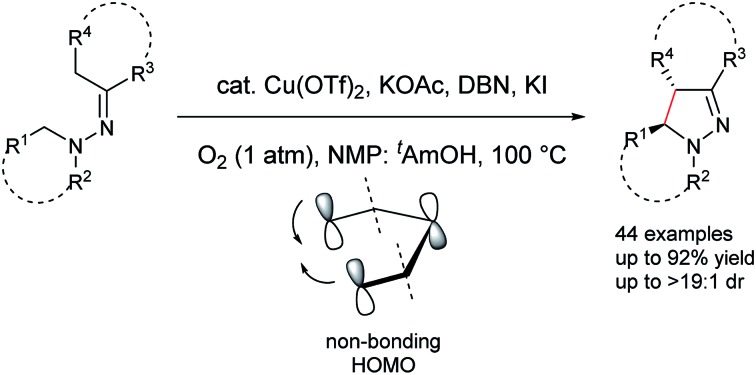
Diastereoselective aerobic dehydrogenative cyclization of hydrazones is described *via* a copper-catalyzed sp^3^ C–H functionalization process.

## Introduction

Selective carbon–carbon (C–C) bond formation is a fundamental focus in chemical research due to its importance in organic, medicinal, agricultural, and material chemistry.^1^ Conventional methods for the construction of C–C bonds, such as nucleophilic addition and substitution reactions, Friedel–Crafts-type reactions, and transition-metal-catalyzed or -mediated cross coupling reactions, rely primarily on prefunctionalized reactants, which somehow limit the synthetic application of these transformations.^2^ Additionally, stoichiometric amounts of toxic metal waste are often generated in the processes, which constitutes an environmental issue. For these reasons, transition metal-catalyzed cross coupling reactions *via* direct functionalization of relatively unreactive C–H bonds has been of great interest in the past two decades.^3^ Among this reaction class, copper-catalyzed aerobic dehydrogenative couplings *via* a double C–H bond functionalization process have received considerable attention in recent years due to the economical and ecological advantages.^4^

The oxidative dimerization of terminal alkynes, the Glaser reaction reported in 1869, is the first example of copper-catalyzed aerobic dehydrogenative coupling.^5^ Since then, considerable efforts have been devoted in this area for selective C–C bond construction, and a variety of copper-catalyzed aerobic dehydrogenative coupling reactions have been reported *via* sp or sp^2^ C–H bond functionalization. Representative examples, which have expanded product diversity, include oxidative dimerization of phenols, naphthols and electron-deficient arenes, cross coupling of terminal alkynes with electron-deficient arenes, and intramolecular dehydrogenative cyclization of anilides.4e,g,^6^ Furthermore, copper-catalyzed aerobic cross dehydrogenative coupling (CDC) *via* an sp^3^ C–H bond functionalization process has also been established on tertiary amines by Miura, Li, and others.4a,e,g It has been demonstrated that a wide range of nucleophiles, such as alkynes, nitroalkanes, malonic esters, ketones, and electron-rich (hetero)arenes, are compatible coupling partners under aerobic oxidative conditions. Mechanistically, an iminium ion intermediate is generated *via* oxidation of the corresponding amine. This intermediate then serves as an active electrophile for the subsequent nucleophilic addition reaction.^7^ Despite its considerable potential, this reaction suffers from a restricted substrate scope of tertiary amines; only substituted 1,2,3,4-tetrahydroisoquinoline and *N*,*N*-dimethylaniline derivatives have been reported as suitable substrates. Furthermore, this transformation is limited to the intermolecular version, in part due to the non-selective oxidation of the amine over the internal nucleophile.^8^

We envisaged that the use of an enamine-type motif as an internal nucleophile could potentially overcome this drawback and extend the reaction scope to intramolecular cyclizations.^9^ Based on this design, copper-catalyzed aerobic intramolecular dehydrogenative cyclization of hydrazones was examined and realized in our laboratory.^10^,^11^ However, it was noted that the initial cyclized intermediate was very unstable under the reaction conditions and was rapidly aromatized to pyrazoles. Furthermore, only α-unsubstituted 1-benzyl-1-isopropyl-2-(1-phenylethylidene)hydrazines were well tolerated under the catalyst system. Additionally, aliphatic hydrazones completely failed in this process due to competitive decomposition of the starting materials. To overcome these drawbacks and thus to provide a straightforward environmentally friendly and atom efficient access to diversified pyrazoline derivatives, copper-catalyzed diastereoselective cyclization of aromatic and aliphatic hydrazones was investigated. It is noteworthy that pyrazoline is a prominent structural motif in many pharmacological compounds, and pyrazoline derivatives display a broad spectrum of biological activities including antibacterial, antidepressant, antidiabetic, antiepileptic, antihypotensive, anti-inflammatory, antimalarial, antimicrobial, antipyretic-analgesic, antituberculotic, antitumor, immunosuppression, insecticidal, muscle relaxant, psycho analeptic, and tranquilizing activities.^12^

Here we report detailed studies in which the copper-catalyzed sp^3^ C–H functionalization strategy was thoroughly examined and further explored to expand its scope of synthetic utility. To provide important mechanistic insights into various factors that control the product distribution, density functional theory (DFT) calculations were further conducted to rationalize the diastereoselectivity observed. The combined work of chemical synthesis, NMR, and computation revealed how the interplay of molecular configurations, steric effects, and the symmetry requirement of electronic structure reactivity, can be utilized to precisely control the diastereoselectivity in the products, thereby providing a valuable guidance to rational designs of the related synthetic routes.

## Results and discussion

Our study commenced with copper-catalyzed dehydrogenative cyclization of 1,1-dibenzyl-2-(1-phenylpropylidene)hydrazine (**1a**) under atmospheric O_2_ (Table 1). After an initial screening of the catalyst, base, and solvent, it was found that the pyrazoline **2a** was obtained in 27% yield, with only trace amount of the undesired pyrazole compound **3**, by using a catalytic amount of Cu(OTf)_2_ and stoichiometric KI, KOAc, and DBU in NMP with O_2_ as the external oxidant (entry 5). Interestingly, this reaction showed high diastereoselectivity, with the *anti* isomer as the major product. Further optimization showed that this reaction was improved by using NMP and ^*t*^AmOH as co-solvent (entry 8). It was then noticed that the reaction yield was significantly increased by extending the reaction time to 12 h from 4 h (entry 9). In addition, the replacement of DBU with DBN significantly increased the reaction rate, and the desired product was obtained in 86% yield within 4 h (entry 10). Moreover, the cyclization could also be effectively catalyzed by other Cu^I^ or Cu^II^ sources under the above modified conditions (entries 13–17).

**Table 1:** Optimization of reaction conditions^*a*^

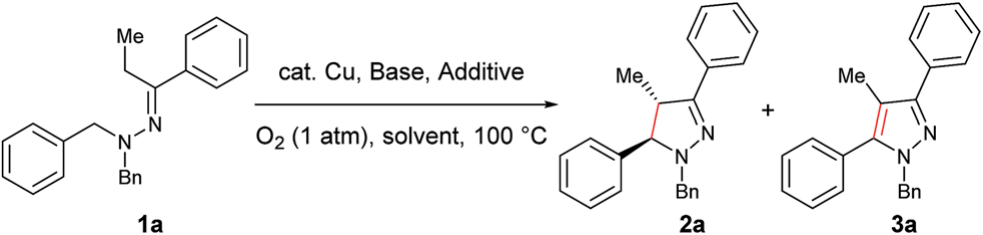							
Entry	Cu source (mol%)	Base (equiv.)	Additive (equiv.)	Solvent	Yield^*b*^ (%)		d.r.^*c*^
					**2a**	**3**	
1	Cu(OTf)_2_ (10)	KOAc (1.0)	—	NMP	<5	—	—
2	Cu(OTf)_2_ (10)	DBU (1.0)	—	NMP	<5	—	—
3	Cu(OTf)_2_ (10)	KOAc (1.0)	KI (1.0)	NMP	23	—	6.8 : 1
4	Cu(OTf)_2_ (10)	DBU (1.0)	KI (1.0)	NMP	10	—	–
5	Cu(OTf)_2_ (10)	KOAc (0.5)/DBU (0.5)	KI (1.0)	NMP	27	—	6.3 : 1
6	Cu(OTf)_2_ (10)	KOAc (0.5)/DBU (0.5)	—	NMP	<5	—	—
7	Cu(OTf)_2_ (10)	—	KI (1.0)	NMP	<5	—	—
8	Cu(OTf)_2_ (10)	KOAc (0.5)/DBU (0.5)	KI (1.0)	NMP/^*t*^AmOH^*d*^	45	3	6.0 : 1
9^*e*^	Cu(OTf)_2_ (10)	KOAc (0.5)/DBU (0.5)	KI (1.0)	NMP/^*t*^AmOH^*d*^	81	4	6.1 : 1
**10**	**Cu(OTf)**_**2**_ **(10)**	**KOAc (0.5)/DBN (0.5)**	**KI (1.0)**	**NMP/** ^***t***^ **AmOH** ^*d*^	**88 (86)** ^*f*^	**6**	**6.8** **:** **1**
11	Cu(OTf)_2_ (10)	KOAc (0.5)/DBN (0.5)	KI (1.0)	^*t*^AmOH	43	3	5.1 : 1
12	—	KOAc (0.5)/DBN (0.5)	KI (1.0)	NMP/^*t*^AmOH^*d*^	0	—	—
13	Cu(TFA)_2_ (10)	KOAc (0.5)/DBN (0.5)	KI (1.0)	NMP/^*t*^AmOH^*d*^	67	5	7.4 : 1
14	Cu(OAc)_2_ (10)	KOAc (0.5)/DBN (0.5)	KI (1.0)	NMP/^*t*^AmOH^*d*^	73	5	6.5 : 1
15	CuI (10)	KOAc (0.5)/DBN (0.5)	KI (1.0)	NMP/^*t*^AmOH^*d*^	62	4	6.3 : 1
16	CuBr (10)	KOAc (0.5)/DBN (0.5)	KI (1.0)	NMP/^*t*^AmOH^*d*^	60	4	6.3 : 1
17	(CuOTf)_2_Py (5)	KOAc (0.5)/DBN (0.5)	KI (1.0)	NMP/^*t*^AmOH^*d*^	82	5	6.7 : 1

^*a*^Reaction conditions: **1a** (0.3 mmol), Cu source, base, additive, O_2_ (1 atm), 2 mL of solvent, 100 °C, 4 h.

^*b*^Yields and conversions are based on **1a**, determined by GC/MS using diphenylketone as the internal standard.

^*c*^d.r. (*anti* : *syn*): determined by ^1^H NMR spectroscopy.

^*d*^2 : 3 (v/v).

^*e*^12 h.

^*f*^Isolated yields.

With the optimized conditions in hand, the scope of hydrazone substrates was studied (Table 2). As expected, aromatic, acyclic and cyclic aliphatic hydrazones were compatible with the oxidative reaction conditions (**2a–h**). Interestingly, the reaction showed a substrate-dependent diastereoselectivity pattern, and the *anti*-isomers of **2a** and **2b** were obtained as the major products while the *syn*-isomers of **2c–g** were the major forms. It is noteworthy that the current methods for the synthesis of *syn*-4,5-pyrazolines rely primarily on the [3 + 2]-cyclization of a diazo compound and an olefin, which often suffers from poor regioselectivity.^13^ Furthermore, the *N*-benzyl group could also be replaced with another alkyl group (**2i–n**), in which the aromatic hydrazones favored the *anti*-isomers, and the aliphatic substrates favored the *syn*-isomers. Additionally, an *N*-phenyl substituted hydrazone yielded exclusively the *syn*-isomer **2o**, providing an important and tunable strategy to selectively access either *anti* or *syn* pyrazolines. Moreover, 1,2,3,4-tetrahydroquinoline is also an effective substrate (**2p**).

**Table 2:** Scope of hydrazones^*a*^ ^,^^*b*^ ^,^^*c*^

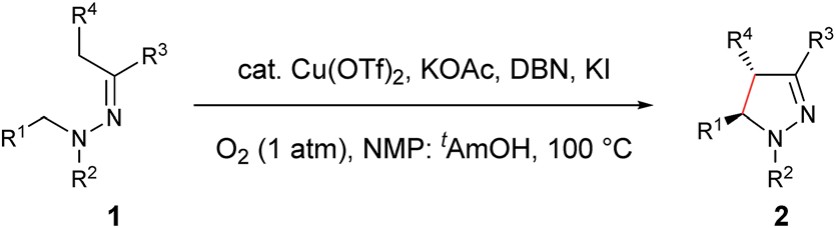
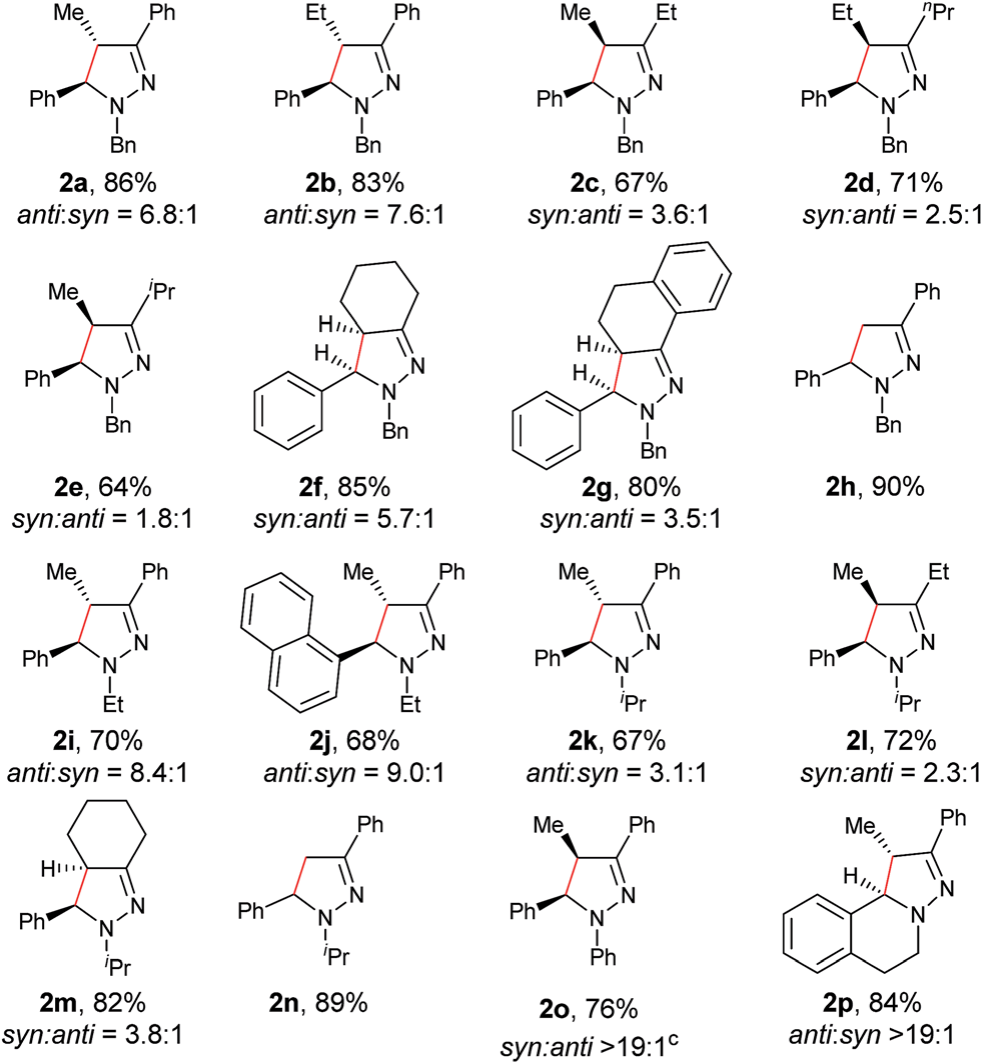

^*a*^Reaction conditions: **1** (0.3 mmol), Cu(OTf)_2_ (10 mol%), KOAc (0.5 equiv.), DBN (0.5 equiv.), KI (1.0 equiv.), O_2_ (1 atm), 2 mL co-solvent NMP and ^*t*^AmOH (NMP/^*t*^AmOH = 2 : 3, v/v), 100 °C, 3–14 h.

^*b*^Isolated yield.

^*c*^d.r. was determined by ^1^H NMR spectroscopy.

In our previous study of dehydrogenative cyclization/aromatization of 1-benzyl-1-isopropyl-2-(1-phenylethylidene)hydrazine, it has been demonstrated that both electron-donating and electron-withdrawing groups on the phenyl rings were compatible.^11^ It is also believed that an oxidized iminium ion intermediate is involved in this process, serving as the precursor for the cyclization. Therefore, in order to simplify the complexity of configuration of the iminium ion intermediates, and thus to better understand the outcome of diastereoselectivity, we carried out an extensive substrate scope study on *N*-cyclohexylidene-3,4-dihydroisoquinolin-2(1*H*)-amine (Table 3). For each substrate, only one isomeric iminium ion intermediate will be generated, which will undergo either 5-center/6-electron cyclization or intramolecular nucleophilic addition to provide the corresponding diastereoisomers. It is worth mentioning that extensive synthetic studies have been carried out with azomethine imines, which have resulted in a variety of applications of the products obtained.^14^ However, to our knowledge, there are only two reported examples which involve the direct use of a dihydroisoquinoline derivative *via* an oxidative sp^3^ C–H activation process: mercuric oxide-mediated dimerization and Rh(iii)-catalyzed cycloaddition.^9^,^15^ Furthermore, systematic electronic or steric effects of the above reactions on 1,2,3,4-tetrahydroisoquinolines have barely been studied.

**Table 3:** Scope of 1,2,3,4-tetrahydroisoquinolines^*a*^ ^,^^*b*^ ^,^^*c*^

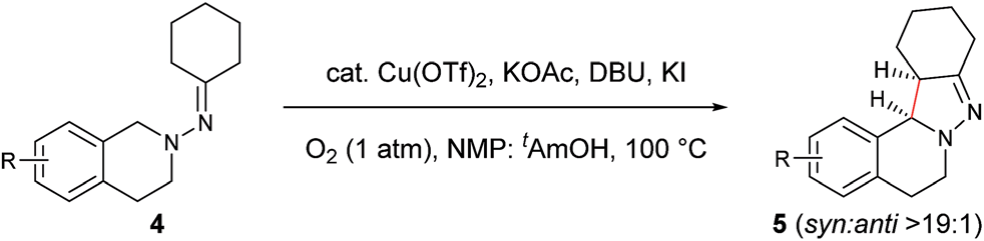
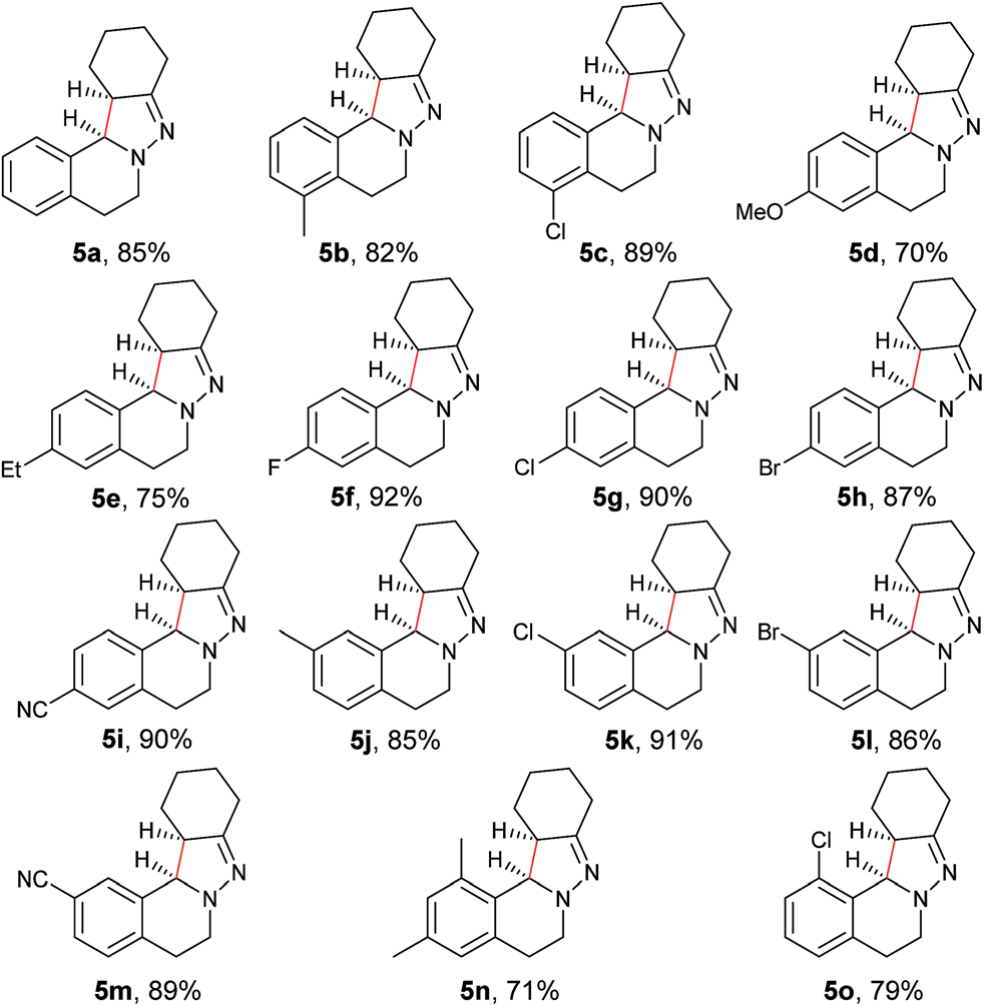

^*a*^Reaction conditions: **4** (0.3 mmol), Cu(OTf)_2_ (10 mol%), KOAc (0.5 equiv.), DBU (0.5 equiv.), KI (1.0 equiv.), O_2_ (1 atm), 2 mL co-solvent NMP and ^*t*^AmOH (NMP/^*t*^AmOH = 2 : 3, v/v), 100 °C, 3.5–4.5 h.

^*b*^Isolated yield.

^*c*^d.r. (*syn* : *anti*) was determined by ^1^H NMR spectroscopy.

As shown in Table 3, both electron-donating and electron-withdrawing groups on the phenyl ring are compatible under the current catalytic system. Interestingly, high diastereoselectivity was observed in all cases with the *syn* diastereoisomers as the major products (**5b–o**). Generally, electron-withdrawing group-substituted substrates provided better yields compared with electron-donating substituents in the same position. Considering that an iminium ion intermediate is formed prior to cyclization, these results are not surprising since an electron-withdrawing group increases the electrophilicity of the iminium ion and thus facilitates the cyclization. Additionally, an electron-withdrawing group may retard the further oxidation of pyrazolines and thus minimize formation of the by-product pyrazoles. Furthermore, a steric effect was observed for this reaction: C8-substituted hydrazones gave lower yields of desired products compared with the C5-, C6-, or C7-substituted substrates. Finally, halogens (F, Cl, and Br) are also well tolerated, which provides the opportunity for further product manipulation.

**Fig. 1:**
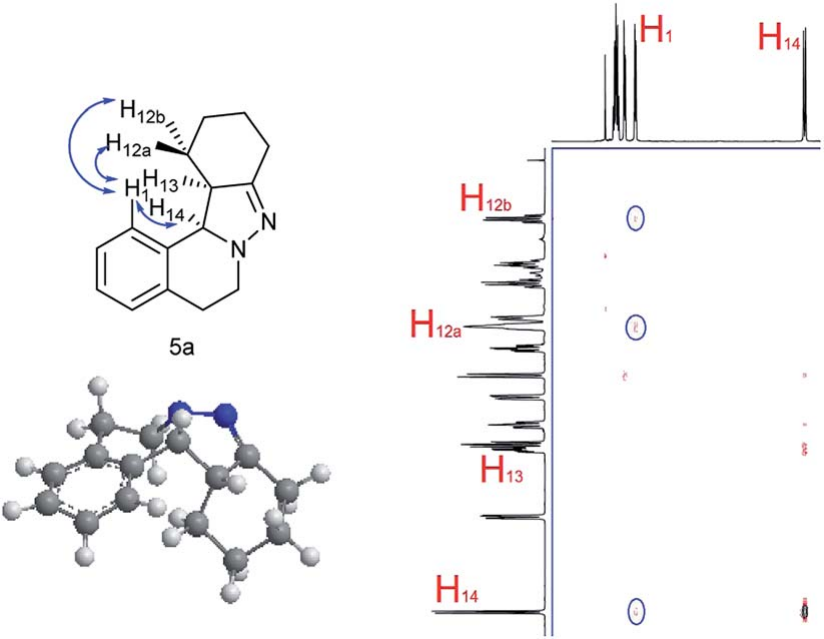
NOESY and relative configuration of **5a**.

NOESY analysis was carried out to confirm the relative configuration of the product (Fig. 1). NOEs between the aromatic proton H_1_ of **5a** and H_12a_, H_12b_ were observed. Additionally, there was no observable NOE between H_1_ and H_13_, or H_14_ and H_12a_ or H_12b_. These results indicate that H_13_ and H_14_ have the *syn* relationship.

Next, an imine substrate scope study was carried out (Table 4). The reaction occurred with high diastereoselectivity on substrates with a larger ring (**5q** and **5r**). The 5-membered ring substrate also provided a good yield of product **5p** under the modified reaction conditions. Furthermore, α,β-unsaturated hydrazones are also compatible (**5u** and **5v**), which allows for further transformation of the initial products. As expected, good to high yields of products were obtained on substrates with substituted cyclohexylidenehydrazine moieties (**5s**, **5t**, **5w–z** and **5aa**). In addition, good to excellent diastereoselectivity was observed with α-substituted cyclohexylidenehydrazine, favoring the *anti*-products (**5w–y**). In comparison, γ-substituents on the cyclohexylidenehydrazine moiety did not significantly affect the relative diastereoselectivity (**5z** and **5aa**). Additionally, linear hydrazones are also compatible with the oxidative conditions, favoring the formation of the *syn*-isomers. However, in this case, the ratio of diastereoselectivity was dramatically decreased. Considering that there are two possible conformers present in the iminium ion intermediate oxidized either from **5z** or **5aa**, the above result is not surprising.

**Table 4:** Scope of the imine moiety^*a*^ ^,^^*b*^ ^,^^*c*^

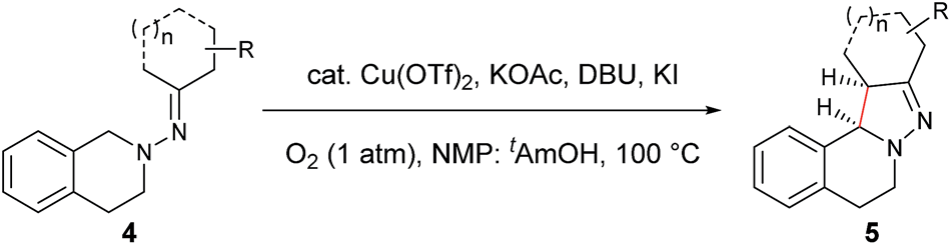
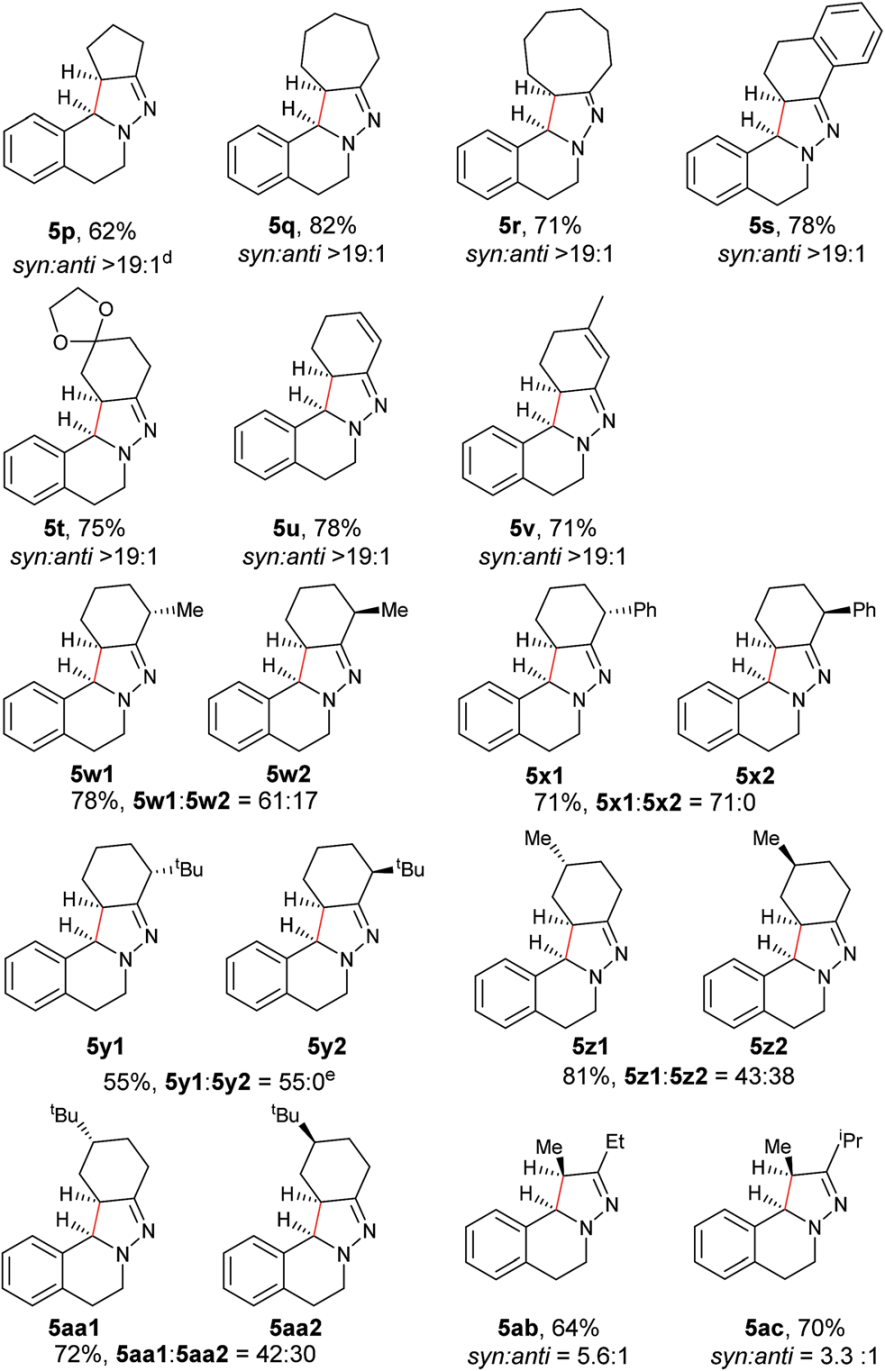

^*a*^Reaction conditions: (0.3 mmol), Cu(OTf)_2_ (10 mol%), KOAc (0.5 equiv.), DBU (0.5 equiv.), KI (1.0 equiv.), O_2_ (1 atm), 2 mL co-solvent NMP and ^*t*^AmOH (NMP/^*t*^AmOH = 2 : 3, v/v), 100 °C, 1–20 h.

^*b*^Isolated yield.

^*c*^d.r. was determined by ^1^H NMR spectroscopy.

**Scheme 1:**
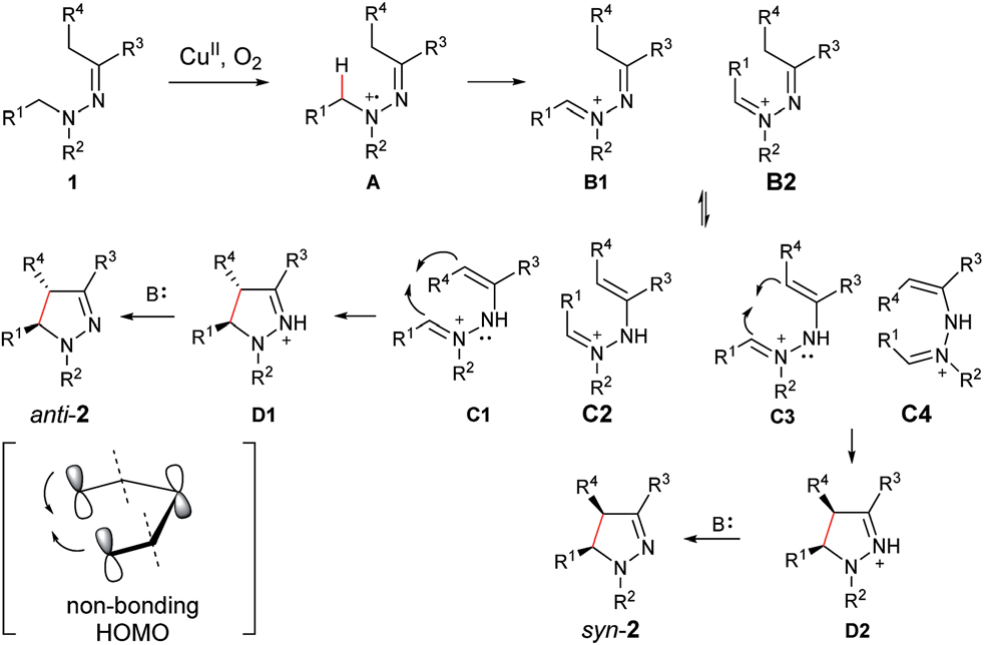
Proposed reaction mechanism.

On the basis of the above results and our previous report,^11^ a plausible reaction mechanism is proposed (Scheme 1).4g,^7^ Oxidation of the amine nitrogen on **1** generates the radical cation **A***via* an SET process. The radical cation **A** is then converted into the iminium ion intermediates **B1** and **B2***via* either an oxidation/deprotonation or a homolytic cleavage process. Tautomerization of the imine moiety on **B1** and **B2** to enamine-type diastereoisomers **C1**, **C2**, **C3** and **C4**, followed by subsequent nucleophilic addition provides the intermediate **D1** and **D2**, which then give pyrazoline **2** upon deprotonation. The rationale of high *syn* over *anti* diastereoselectivity is also proposed based on the previous reports by Hoffmann and List.^16^,^17^ It is believed that the neutral nitrogen in intermediate **C** will behave as an sp^2^ hybridized atom. Therefore, the lone-pair of electrons on the nitrogen will participate in the π-conjugation together with the four π electrons in the double bonds, resulting in a 5-center/6-electron system, similar to the situation found in a pentadienyl anion. For such homoconjugated systems, the HOMO is actually a “symmetric” nonbonding orbital, which is 1,5-bonding with the preferred U planar configuration. For thermally induced electrocyclic ring closures, the symmetric HOMO requires a disrotatory mechanism, which results in the corresponding product **2**. It should be mentioned that α-alkyl Cu species could potentially be formed from deprotonation of the intermediates **B1**/**B2** under basic conditions. Intramolecular nucleophilic addition of these Cu species could also provide the desired products.

### Mechanistic studies with computation

Toward molecular-level understanding of diastereoselective 5-center/6-electron cyclization mechanism displayed in these copper-based C–H functionalization reactions, we performed density functional theory (DFT) investigations on five representative systems (**2a**, **2f**, **2p**, **5a**, and **5ab**).

### Computational methods

All the calculations were carried out for the five systems by using Gaussian 09 program suites.^18^ The Kohn–Sham density functional theory (DFT) was solved with the B3LYP functional,^19^ and the 6-31G+(d,p) basis sets were selected. The key stationary species (**B**, **C**, **D**) related to diastereoselective cyclization in Scheme 1 of these five systems were fully optimized in the gas phase. The transition states (**TS**) from **C** → **D**, which seems to be the most relevant step in determining the diastereoselectivity of the products, were especially emphasized, and the transition states from **B** → **C** are outside the scope of this work and thus were excluded. The influence of the solvent environment on the reaction was evaluated by using the polarizable continuum models (PCM) method^20^ with the gas-phase optimized geometries. The molecular cavity was treated using the united atom Hartree–Fock (UAHF) parameterization. Since *N*-methylpyrrolidone (*ε* = 32.2) and 2-methyl-2-butanol (*ε* = 5.78) are not available in Gaussian 09, we chose ethanol as the solvent (*ε* = 24.55). Frequency analysis was carried out to verify the optimized geometry as minima or transition state, and to obtain free energy of each species. The intrinsic reaction coordinate (IRC)^21^ calculation was applied to judge whether the transition state connects the reactants and the products.

In all systems, the symbols used to represent the related species are: hydrazine (**1**), the iminium ion (**B**), the enamine-type intermediate (**C**) and the charged pyrazoline product (**D**). For **2f**, **2p**, **5a**, and **5ab**, **C1**/**C2** represents the enamine-type intermediates with *trans*/*cis* conformation for the C=C double bond; **D1**/**D2** represent the *syn*/*anti* charged pyrazoline intermediates. For **2a**, **B1**/**B2** is the iminium ion with *trans*/*cis* conformation for the C=N^+^ double bond; **D1**/**D2** are the *anti*/*syn* charged pyrazoline intermediates. Given the conformational combinations of the C=N^+^ and C=C double bonds, four enamine-type intermediates are considered for this system, which are referred to as **C1** (*trans*, *cis*), **C2** (*cis*, *trans*), **C3** (*trans*, *trans*) and **C4** (*cis*, *cis*), respectively. The transition state of a disrotatory/conrotatory reaction is specified by “a”/“b”, for example, **TS2a**/**TS2b** is the transition state of the disrotatory/conrotatory reaction of **C2**. The proposed mechanism, optimized geometries, some free energy profiles, and original data are shown in ESI.†

### Computational results for the formation of **5a**

Based on the complexity of these systems, the computational results of **5a** is first represented here. The charge of the carbon atom in the C=N^+^ bond is –0.769*e* in **C1**, –0.609*e* in **TS1a**, and –0.466*e* in **TS1b**, respectively. The charge of the terminal carbon atom in the C=C bond is 0.916*e* in **C1**, 0.679*e* in **TS1a**, and 0.729*e* in **TS1b**, respectively. As shown in Fig. S1,† the dihedral angles *D*(C=N^+^–N–C) and *D*(N^+^–N–C=C) of the pyrazoline ring in **TS1a** are 22.1° and –19.6°, respectively. The C=N^+^ and C=C bonds form a pseudo-plane with the dihedral angle *D*(C=N^+^···C=C) to be 3.1°, and the neutral nitrogen atom is a little out of that plane. For **TS1b**, the values of *D*(C=N^+^–N–C), *D*(N^+^–N–C=C) and *D*(C=N^+^···C=C) are 35.0°, 7.5°, and 40.2°, respectively, implying that the pyrazoline ring in **TS1b** is twisted and it looks like a helical folding conformation. Clearly, compared with the C=N^+^–N–C=C in **TS1b**, the C=N^+^–N–C= **C** in **TS1a** is closer to a coplanar conformation. The distance *R*(C=N^+^···C=C) between the two terminal carbon atoms of the pyrazoline ring is 2.25 Å in **TS1a** and 2.15 Å in **TS1b**. These show that the **C** → **D** reaction is a 5-center/6-electron cyclization constituted by C=N^+^–NH–C=C, although this system does not completely satisfy the Hückel rule (not typically coplanar).

**Fig. 2:**
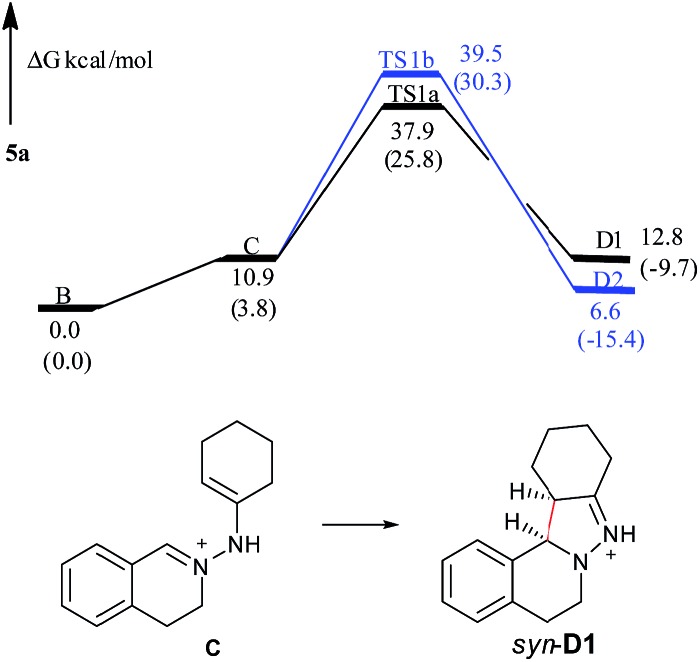
Free energy profiles of the reaction generating **5a** in the gas phase. Free energies in the solution phase are given in parentheses.

In the free energy profiles (Fig. 2), **C** is less stable than its tautomer **B** by 10.9 kcal mol^–1^. The reaction is endothermic by 1.9 kcal mol^–1^ for **C** → **D1** process, and exothermic by 4.3 kcal mol^–1^ for **C** → **D2** process. Even though the conformation of the cyclohexane is chair in **D1** and twist boat in **D2**, **D2** (*anti*) shows more stability than **D1** (*syn*). The reason may be that the two bulky substituents are on the same side of the pyrazoline ring in **D1**, while the two substituents are on the different side of the pyrazoline ring in **D2**, causing the steric repulsion being stronger in **D1** than in **D2**. However, the active free energy generating **D1** is 1.6 kcal mol^–1^ lower than that generating **D2**, indicating that **C** prefers to undergo disrotatory process (**TS1a**) to form **D1**, rather than conrotatory process (**TS1b**) to afford **D2**. This may be because the C=N^+^–N–C=C (5-center/6-electron) in **TS1a** is more coplanar and more satisfies the Hückel rule compared with that in **TS1b**. These are in good agreement with the diastereoselectivity observed in the copper-based C–H functionalization reactions (*syn*-**D1** is major product).

**Fig. 3:**
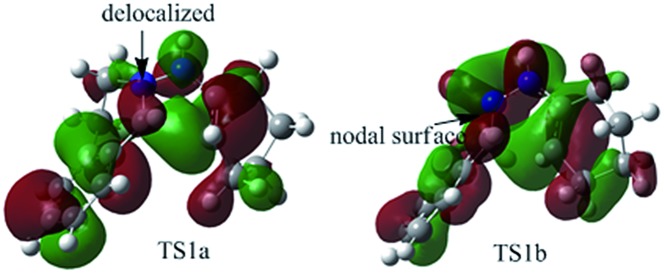
HOMOs of **TS1a** and **TS1b** generating **5a**.

To better understand this point, HOMOs of **TS1a** and **TS1b** were compared. As shown in Fig. 3, the under lobe of the orbital of the C atom in the C=N^+^ bond overlaps with the under lobe of the orbital of the terminal C atom in the C=C bond, and C=N^+^–NH constitutes a delocalized system in **TS1a**. In **TS1b**, the under lobe of the orbital of the C atom in the C=N^+^ bond overlaps with the upper lobe of the orbital of the terminal C atom in the C=C bond. However, the delocalized orbitals of the N–NH bond do not overlap with the C atom in the C=N^+^ bond, generating an orbital nodal surface between the C atom and the N atoms. These demonstrate that **TS1b** is less stable and more difficult to cross than **TS1a**, supporting that the intermediate **C** tends to undergo a disrotatory process to form **D1**, rather than a conrotatory process to afford **D2**.

The presence of a solvent can obviously improve the reaction process (the **C** → **D1**/**D2** processes are exothermic and the active free energy barriers are easier to overcome, shown in parentheses in Fig. 2), but the overall diastereoselective tendency is the same as that captured from gas-phase calculations. Such a huge improvement of the reaction is mainly attributed to the solvation of ions.

### Computational results for the formation of **2a**

The charge of the carbon atom in the C=N^+^ bond is –0.464*e* in **C1**, –0.321*e* in **C2**, –0.313*e* in **C3**, –0.579*e* in **C4**, –1.407*e* in **TS1a**, –0.388*e* in **TS1b**, –0.983*e* in **TS2a** and –0.656*e* in **TS2b**, respectively. The charge of the terminal carbon atom in the C=C bond is separately 0.139, 0.337, 0.641, –0.077, 0.131, 0.297, 0.042 and –0.161*e* for the corresponding species. Seen from Fig. S2,† the dihedral angles *D*(C=N^+^–N–C) and *D*(N^+^–N–C=C) of the pyrazoline ring in **TS1a** are 30.0° and –33.8°, respectively. The C=N^+^ and C=C bonds form a pseudo-plane with the dihedral angle *D*(C=N^+^···C=C) of –2.4°, and the neutral nitrogen atom is a little out of that plane. The corresponding values are separately –44.1°, 4.3° and –37.7° in **TS1b**, also suggesting that the pyrazoline ring in **TS1b** is a pseudo-plane. However, this pyrazoline ring in **TS1b** is slightly apart from the coplanar conformation compared with that in **TS1a**. In **TS2a**, the values of *D*(C=N^+^–N–C), *D*(N^+^–N–C=C) and *D*(C=N^+^···C=C) are separately 37.6°, 23.3° and 56.5°, implying that the pyrazoline ring is twisted and it looks like a helical folding conformation. The corresponding values are separately –29.2, –18.9° and –43.7° in **TS2b**, showing that the pyrazoline ring in **TS2b** is twisted with the opposite direction, but the twist with the opposite direction would increase the steric hindrance between the methyl group and the benzyl group. The distance *R*(C=N^+^···C=C) between the two terminal carbon atoms of the pyrazoline ring is 2.36 Å in **TS1a**, 2.32 Å in **TS1b**, 2.34 Å in **TS2a**, and 2.34 Å in **TS2b**. These also demonstrate that the C=N^+^–NH–C=C constitutes a 5-center/6-electron system, but not a typical Hückel system (not completely coplanar).

**Fig. 4:**
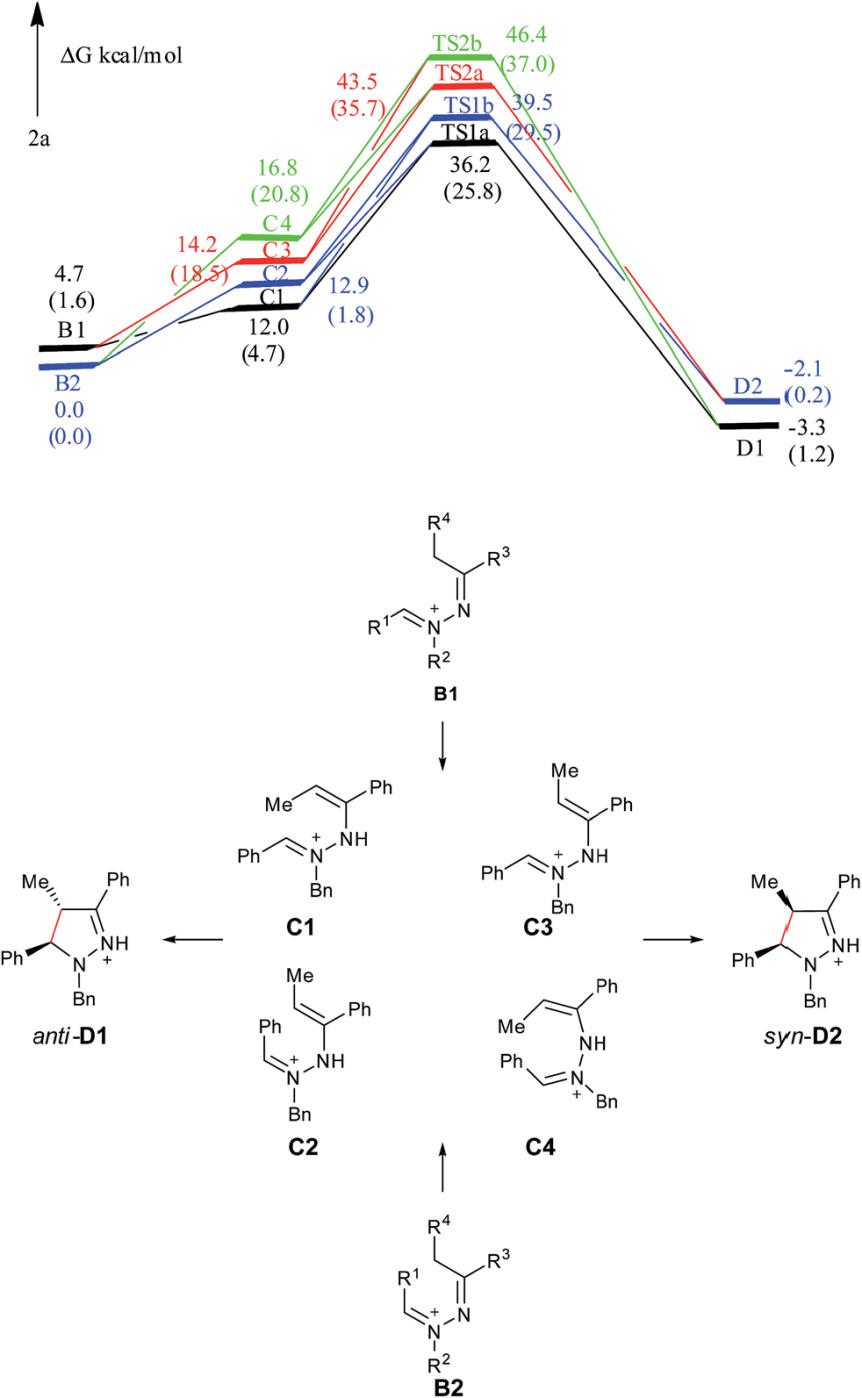
Free energy profiles of the reaction generating **2a** in the gas phase. Free energies in the solution phase are given in parentheses.

As shown in Fig. 4, **C1** and **C2** are more stable than **C3** and **C4**, supporting that **C1** and **C2** are major intermediate compared with **C3** and **C4**. The *anti* product **D1** is more stable than the *syn* product **D2** in the gas phase, which is also attributed to the steric repulsion between the two bulky substituents on the same side of the pyrazoline ring in **D2** compared with **D1**. **TS1a** is –3.3 kcal mol^–1^ relative to **TS1b**, and **TS2a** is –3.1 kcal mol^–1^ relative to **TS2b**, showing that **C** tends to undergo a disrotatory (**TS1a**/**TS2a**) process to give the product, rather than a conrotatory process (**TS1b**/**TS2b**) to afford the product. The reason may be that the C=N^+^–N–C=C in **TS1a** is closer to the Hückel system compared with that in **TS1b** (5-center/6-electron and coplanar conformation), and the steric hindrance between the methyl group and the benzyl group in **TS2a** is smaller than that in **TS2b**. Compared with **C3** and **C4**, **C1** and **C2** overcome less of a free energy barrier, determining the diastereoselectivity of this reaction. Similar to **5a**, the presence of a solvent can obviously improve the reaction process (the **C** → **D1**/**D2** processes are exothermic and the active energy barriers become easier to overcome), but the overall diastereoselective trend is the same as that captured from gas-phase calculations. The main reason for such a huge improvement of the reaction is also the solvation of ions.

**Fig. 5:**
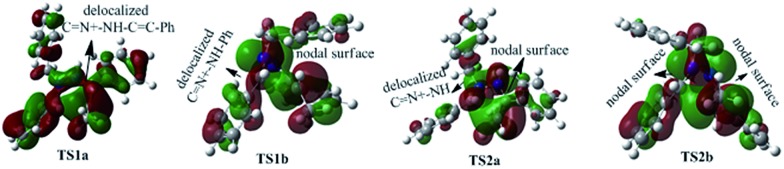
HOMOs of **TS1a**, **TS1b**, **TS2a** and **TS2b** generating **2a**.

To better understand this point, HOMOs of **TS1a**, **TS1b**, **TS2a** and **TS2b** were compared. Seen from Fig. 5, the under lobe of the orbital of the C atom in the C=N^+^ bond overlaps with the under lobe of the orbital of the terminal C atom in the C=C bond, and C=N^+^–NH–C=C and phenyl group constitute a delocalized system in **TS1a**. In **TS1b** the under lobe of orbital of C atom in C=N^+^ bond overlaps with the under lobe of the orbital of the terminal C atom in the C=C bond, but the delocalized system only consists of C=N^+^–NH and phenyl groups. The delocalized system in **TS1b** is smaller than **TS1a**, suggesting that **TS1b** is less stable and more difficult to overcome than **TS1a**. Compared with the orbital in **TS2a**, the C=N^+^–NH–C=C in **TS2b** does not constitute a complete delocalized orbital, and two orbital nodal surfaces are found between the C and N^+^–NH, and N^+^–NH and C=C bonds, also suggesting that **TS2b** is less stable and more difficult to cross than **TS2a**. Therefore, **C1** (**C2**) goes through a disrotatory process to form **D1** (**D2**), instead of a conrotatory process to afford **D2** (**D1**).

**Scheme 2:**
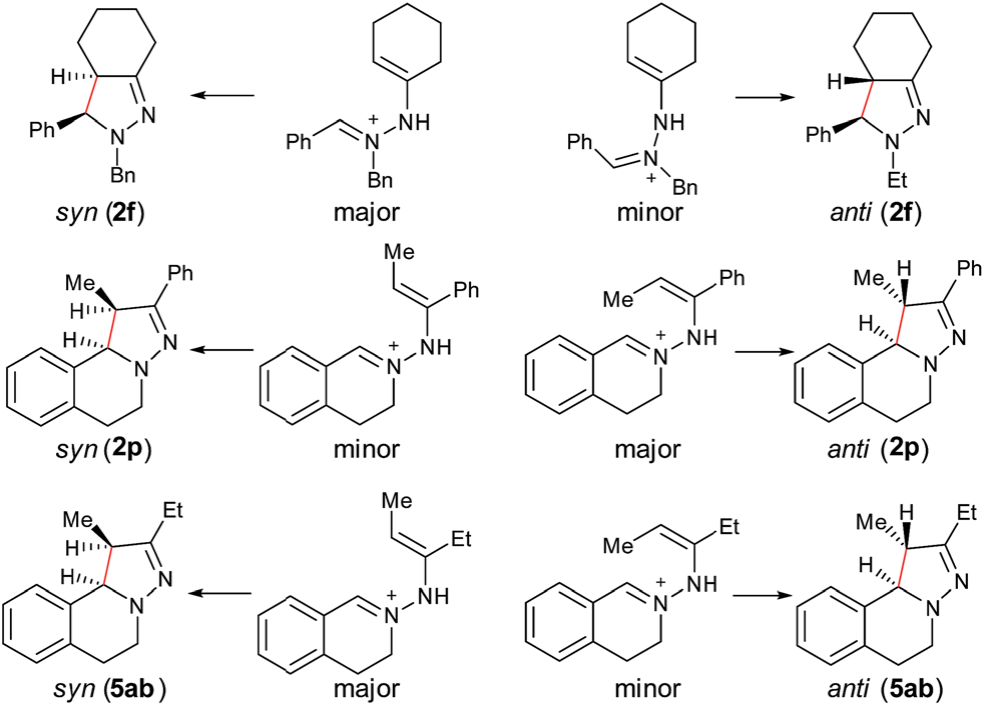
Interpretation of stereoselectivity of **2f**, **2p**, and **5ab**.

Furthermore, computational studies of three more representative substrates were also carried out to account for the diastereoselective outcome (Scheme 2). For **2f**, two enamine-type iminium ion intermediates are formed in the cyclization process, resulting in two different diastereoisomers. Based on the calculation (see ESI†), the (*E*)-isomer is more stable, resulting in *syn*-**2f** as the major product *via* a 5-center/6-electron disrotatory cyclization process. In the case of **2p**, the intermediate with the methyl and phenyl groups on the opposite side is the major isomer, and thus the *anti*-isomer becomes the major product. For **5ab**, the intermediate with two alkyl groups on the same side becomes the major isomers, and thus the *syn*-**5ab** is the major product. Compared with **5ab**, the diastereoselectivity in **5ac** is lower because the ratio of the major isomer is decreased due to the stronger steric effect by the isopropyl group. The combined results from experimental and computational studies suggest that the diastereoselectivity of products is possibly determined by the relative stability of the enamine-type iminium ion intermediates *via* a 5-center/6-electron disrotatory cyclization process.

## Conclusions

In summary, a highly diastereoselective aerobic intramolecular dehydrogenative cyclization of *N*,*N*-disubstituted hydrazones with good functional group tolerance was developed using a copper-catalyzed double sp^3^ C–H bond functionalization process. The observed diastereoselectivity was rationalized by a 5-center/6-electron disrotatory cyclization mechanism, which was supported by computational studies. As the first example of the copper-catalyzed aerobic diastereoselective intramolecular cyclization of amines *via* an iminium ion intermediate, this transformation provides a straightforward, environmentally friendly and atom efficient access to pyrazolines with up to five fused-ring systems. The enantioselective version of this transformation is currently under study in our laboratory.

## Abbreviations

CCR2: CC chemokine receptor 2
CCL2: CC chemokine ligand 2
CCR5: CC chemokine receptor 5
TLC: Thin layer chromatography

## Supplementary Material

Supplementary information
