# Supplementary material for: Copper-catalyzed diastereoselective aerobic intramolecular dehydrogenative coupling of hydrazones via sp3 C–H functionalization
Source: Chem Sci. 2015 Jul 14;6(10):5882–90. doi: 10.1039/c5sc01736j (PMC5950562; doi:10.1039/c5sc01736j)
Supplement: Supplementary file 1 [file SC-006-C5SC01736J-s001.pdf]

Electronic Supplementary Material (ESI) for Chemical Science.
This journal is © The Royal Society of Chemistry 2015

Copper-Catalyzed Diastereoselective Aerobic Intramolecular Dehydrogenative Coupling of Hydrazones via sp^3 C-H Functionalization

Xuesong Wu, Mian Wang, Guangwu Zhang, Yan Zhao, Jianyi Wang,* and Haibo Ge*

Table of Contents

General Information..........S2
Structures of Starting Materials..........S3
General Procedure for the Preparation of *N,N*-Disubstituted Hydrazones 1a-g, 1i-j, 1o..........S4
General Procedure for the Preparation of *N,N*-Disubstituted Hydrazones 1l, 1p, 4a-h, 4j-l, 4n-z, 4aa, 4ab, 4ac..........S5
Procedure for the Preparation of Hydrazines 4i and 4m..........S6
General Procedure for the Dehydrogenative Cyclization Reactions..........S6
Analytical Data for Starting Materials..........S7
Analytical Data for Products..........S20
Scheme S1. Proposed mechanism of stereoselectivity of 2a..........S42
Figure S1. Optimized geometries in 5-center/6-electron cyclization generating 2a..........S43
Scheme S2. Proposed mechanism of stereoselectivity of 2f..........S43
Figure S2. Optimized geometries in 5-center/6-electron cyclization generating 2f..........S44
Figure S3. Free energy profiles in 5-center/6-electron cyclization process generating 2f in Scheme 3 in the gas phase. Free energies in the solution phase are given in parentheses...........S44
Scheme S3. Proposed mechanism of stereoselectivity of 2p..........S45
Figure S5. Free energy profiles in 5-center/6-electron cyclization generating 2p in Scheme 3 in the gas phase. Free energies in the solution phase are given in parentheses...........S46
Scheme S4. Proposed mechanism of stereoselectivity of 5a..........S46
Scheme S5 Proposed mechanism of stereoselectivity of 5ab..........S47
Figure S8. Free energy profiles of the reaction generating 5ab in scheme 3 in the gas phase. Free energies in the solution phase are given in parentheses...........S47
Figure S9. HOMOs of TS1a, TS1b and TS2b generating 5ab..........S48
Table S1. Electronic energies (E*elec*), Zero-point energies (E_{zp}), Gibbs free energies (G*gas*), and solvent free energies (ΔG*sol*) of all stationary points. The E*elec* , G*gas* , and E_{zp} energies are in a.u. and ΔG*sol* are in kcal/mol...........S49
References..........S50
^{1}H and ^{13}C NMR Spectra..........S51
2D-NOESY..........S259

General Information

^{1}H and ^{13}C NMR spectra were recorded on a Bruker 500 MHz NMR Fourier transform spectrometer (500 MHz and 125 MHz, respectively) using tetramethylsilane as an internal reference, and chemical shifts (δ) and coupling constants (J) were expressed in ppm and Hz, respectively. Infrared spectra were obtained using a Thermo Nicolet IR 330 spectrometer. Mass (MS) analysis were abtained using Agilent 1100 series LC/MSD system with Electrospray Ionization (ESI). All the solvents and commercially available reagents were purchased from commercial sources and used directly. Hydrazone **1h**, **1k**, **1m** and **1n** were prepared according to literature procedures.[1] 1,4-Dioxaspiro[4.5]decan-8-one was prepared from cyclohexane-1,4-dione according to literature procedures.[2]

The relative configuration of **2a-g**, **2i-m**, **2o-p**, **5a**, **5w1**, **5y1**, **5aa1** and **5ab** was assigned by NOESY analysis, and that of the rest of products was assigned by analogy.

Structures of Starting Materials

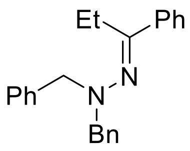

1a

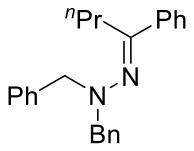

1b

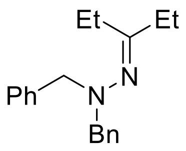

1c

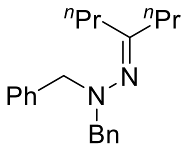

1d

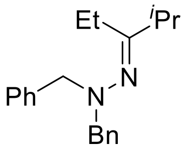

1e

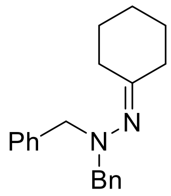

1f

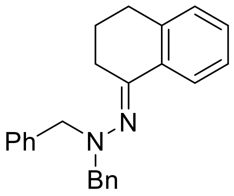

1g

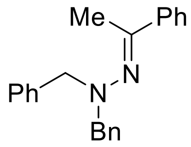

1h

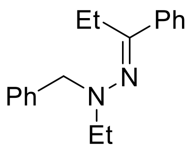

1i

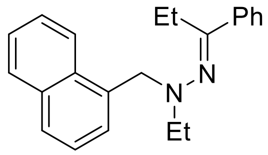

1j

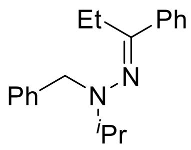

1k

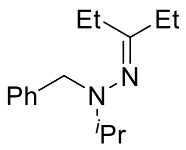

1l

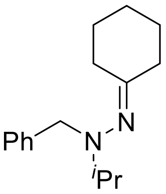

1m

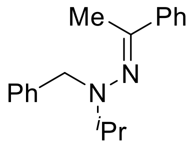

1n

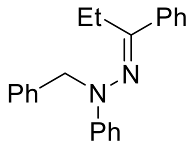

1o

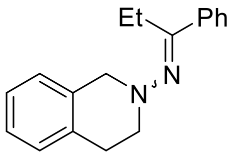

1p

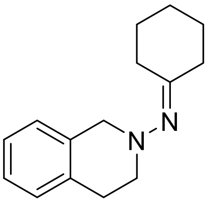

4a

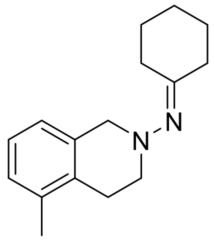

4b

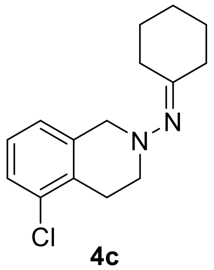

4c

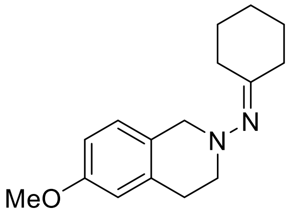

4d

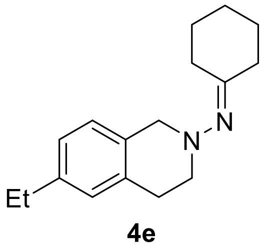

4e

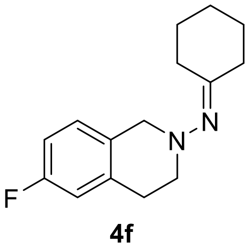

4f

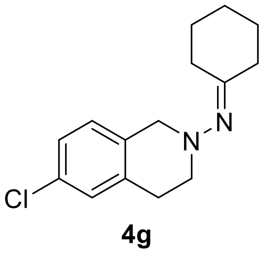

4g

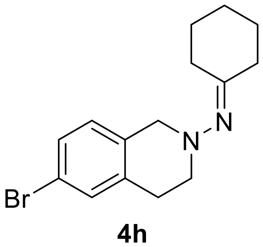

4h

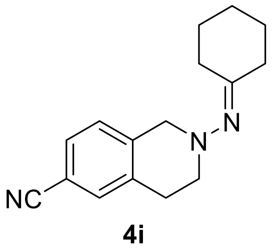

4i

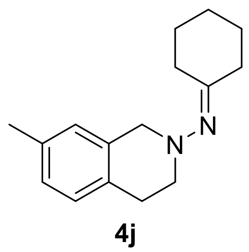

4j

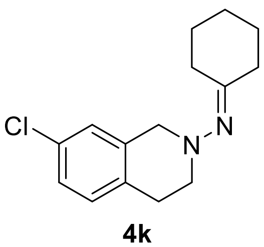

4k

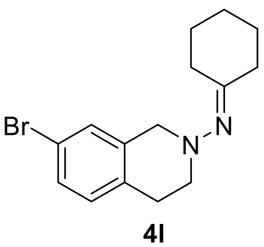

4l

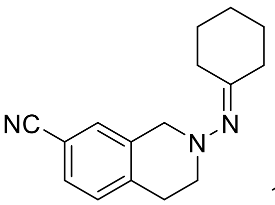

4m

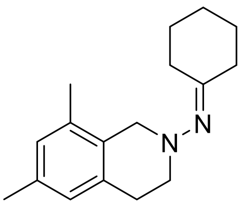

4n

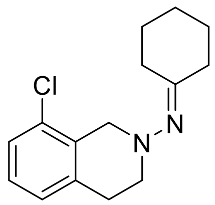

4o

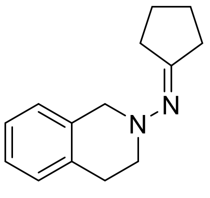

4p

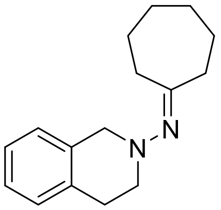

4q

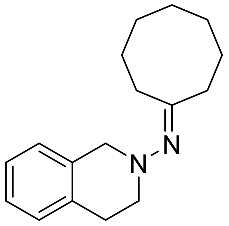

4r

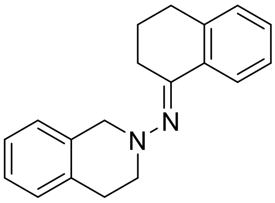

4s

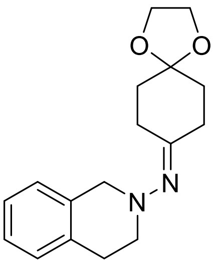

4t

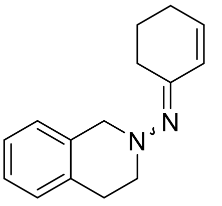

4u

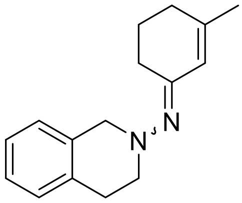

4v

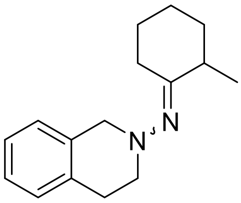

4w

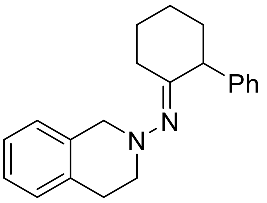

4x

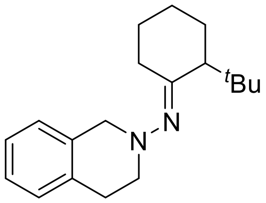

4y

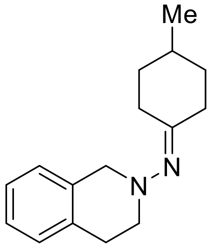

4z

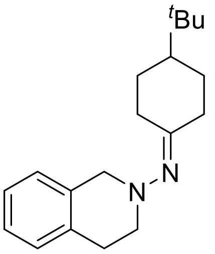

4aa

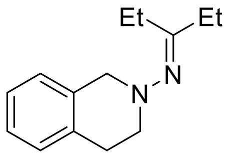

4ab

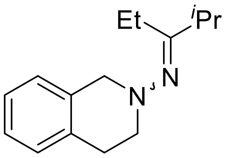

4ac

General Procedure for the Preparation of *N,N*-Disubstituted Hydrazones 1a-g, 1i-j, 1o

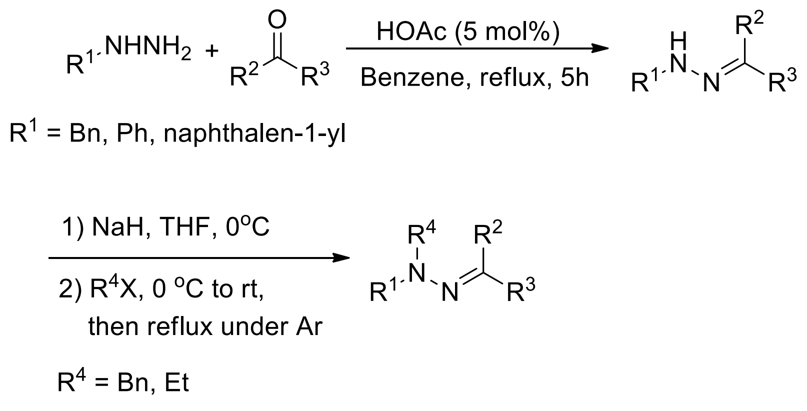

A 100 mL three-necked flask was charged with 30 mL anhydrous benzene, ketone (10 mmol), hydrazine (15 mmol) and acetic acid (28.6 µL, 0.50 mmol) under Ar. The reaction mixture was then refluxed for 5 h. After removal of solvent, the hydrazone, which was usually obtained in nearly quantitative yield, was used directly for the next step without further purification.

To a suspension of NaH (95%, 1.2 g, 48 mmol) in dry THF (20 mL), the solution of hydrazone (5.0 mmol in 5.0 mL dry THF) was added slowly at 0 °C under Ar. The mixture was stirred for 15 min, and then benzyl bromide or alkyl iodide (7.5 mmol) was added dropwise. After stirring at room temperature for 1.5 h, the reaction mixture was refluxed for 8 h. The reaction mixture was cooled to the room temperature, and then the solvent was removed under reduced pressure. The residue was slowly diluted with water (15 mL), extracted with ether (25 mL x 3), and dried over Na_2SO_4. After removal of the solvent, the residue was purified by flash chromatography column on silica gel (gradient eluent of EtOAc/hexanes: 1:100 ~ 1:20, v/v) to yield the product **1** as a yellow oil.

General Procedure for the Preparation of *N,N*-Disubstituted Hydrazones 1l, 1p, 4a-h, 4j-l, 4n-z, 4aa, 4ab, 4ac

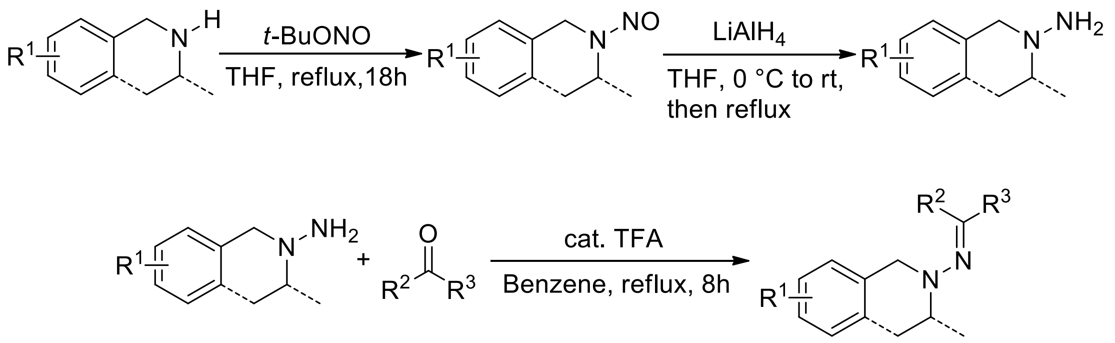

To a solution of 1,2,3,4-tetrahydroisoquinoline or *N*-benzylpropan-2-amine (6.0 mmol) in THF (4.0 mL) was added *tert*-butyl nitrite (1.07 mL, 0.93 g, 9.0 mmol) under Ar. The mixture was heated under reflux for 20 h. Then the solvent and the excess of the *tert*-butyl nitrite were removed in vacuo. The residual oil was dissolved in THF (3.0 mL), and then added dropwise to an ice-cooled, stirred suspension of $LiAlH_4$ (0.32 g, 8.4 mmol) in THF (9.0 mL). After the addition, the suspension was stirred at room temperature for 1 h and then refluxed for 2 h. After cooled to 0 °C, the reaction mixture was added water (1.3 mL) dropwise, the precipitated salts were filtered and washed with THF. The combined filtrate was dried over anhydrous sodium sulfate, and concentrated under reduced pressure to give crude hydrazine as a yellow oil, which was used directly for the next step without further purification.

To a solution of crude hydrazine (5.0 mmol) in anhydrous benzene (6.0 mL) were added ketone (5.0 mmol) and one drop trifluoroacetic acid (no trifluoroacetic acid for **4u** and **4v**) under Ar. The mixture was heated to reflux with Dean-Stark apparatus for 8-16h, and then cooled to room temperature, dried over anhydrous sodium sulfate, filtered and concentrated under reduced pressure. The residue was purified by flash column chromatography on silica gel, eluting with

EtOAc/ Et_3N /hexane (1:1:100, v/v), to afford corresponding *N*,*N*-disubstituted hydrazone.

Procedure for the Preparation of Hydrazines 4i and 4m

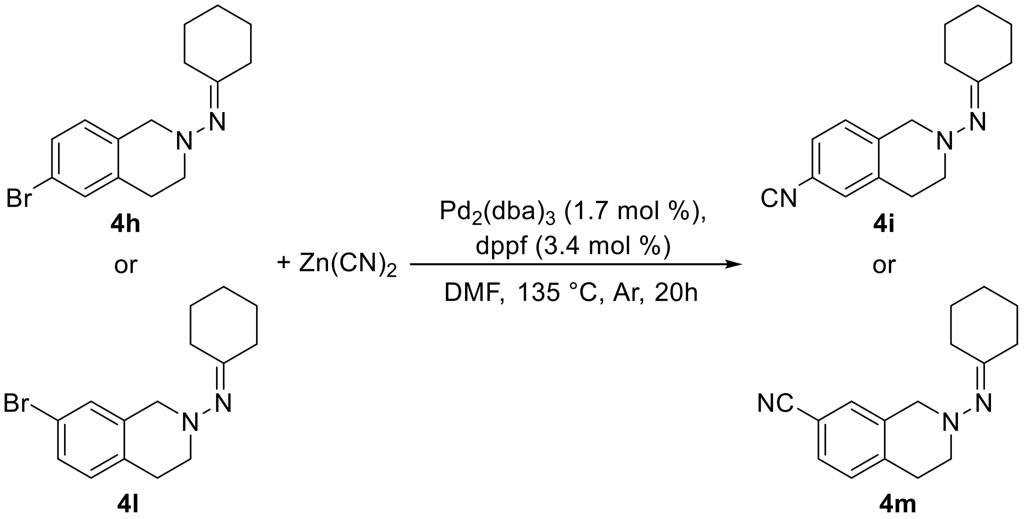

A 50 mL Schlenk tube was charged with $Pd_2(dba)_3$ (9.2 mg, 0.010 mmol), dppf (11.0 mg, 0.020 mmol), and $Zn(CN)_2$ (84.6 mg, 0.72 mmol). Then hydrazine **4h** or **4l** (0.60 mmol) in DMF (6.0 mL) was added, and the vial was degased and filled with argon. After stirring at 135 °C for 20 h, the reaction mixture was cooled to room temperature, diluted with EtOAc (30 mL) and filtered through a pad of Celite. The filtrate was washed with water (3 × 30 mL) to remove the DMF. The organic phase was dried over anhydrous sodium sulfate, filtered and concentrated under reduced pressure. The residue was purified by flash column chromatography on silica gel, eluting with EtOAc/ Et_3N /hexane (1:1:100, v/v), to afford hydrazine **4i** or **4m**.

General Procedure for the Dehydrogenative Cyclization Reactions

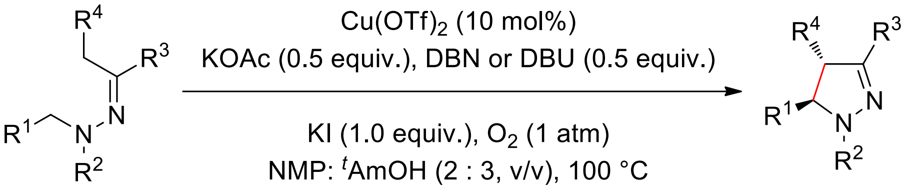

1 or **4** **2** or **5**

A 50 mL Schlenk tube was charged with hydrazone **1** or **4** (0.30 mmol), $Cu(OTf)_2$ (10.9 mg, 0.030 mmol, 10 mol %), KOAc (14.7 mg, 0.15 mmol), DBN (18.6 mg, 0.15 mmol) or DBU (22.8 mg, 0.15 mmol), KI (49.8 mg, 0.30 mmol), NMP (0.80 mL) and ^{t}AmOH (1.20 mL). The vial was evacuated and filled with O_2 (1 atm), and stirred rigorously at 100 °C for 2-12h (monitored by GC and TLC). The mixture was cooled to room temperature, diluted with EtOAc (20 mL). The organic layer was washed with brine (2 × 10 mL), dried over anhydrous sodium sulfate, and concentrated under reduced pressure. The residue was purified by flash column chromatography on silica gel, eluting with EtOAc/hexane (1:100 ~ 1:2, v/v), to afford corresponding product **2** or **5**.

Analytical Data for Starting Materials

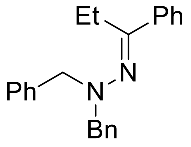

1a

Compound **1a**, yellow solid, yield: 79% (two steps from ketone). ^{1}H NMR (500 MHz, $CDCl_3$, a mixture of (Z/E) isomers in ratio 93:7, the minor one is marked with an *) δ 0.55 (t, J = 7.6 Hz, 3H), 2.61 (q, J = 7.6 Hz, 2H), 3.92 (s, 4H), 7.19-7.24 (m, 2H), 7.27-7.33 (m, 7H), 7.37 (d, J = 7.4 Hz, 4H), 7.42 (d, J = 7.2 Hz, 2H); 0.97* (t, J = 7.5 Hz, 3H), 2.47* (q, J = 7.5 Hz, 1H), 3.70* (s, 4H), 7.10-7.45* (m, 15H); ^{13}C NMR (125 MHz, $CDCl_3$) δ 11.0, 22.5, 62.6, 127.1, 127.2, 128.2, 128.4, 129.3, 129.8, 137.9, 138.9, 174.4; IR (neat) ν 2968, 2935, 2828, 1612, 1494, 1453, 1318, 1028, 950, 737, 696; Ms (ESI): m/z = 329.2 $[M+H]^+$.

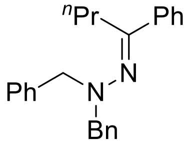

1b

Compound **1b**, yellow solid, yield: 81% (two steps from ketone). ^{1}H NMR (500 MHz, $CDCl_3$, a mixture of (Z/E) isomers in ratio 89:11, the minor one is marked with an *) δ 0.65 (t, J = 7.4 Hz, 3H), 0.92-1.01 (m, 2H), 2.56-2.61 (m, 2H), 3.91 (s, 4H), 7.18-7.24 (m, 3H), 7.27-7.32 (m, 6H), 7.35-7.42 (m, 6H); 0.75* (t, J = 7.4 Hz, 3H), 0.97-1.02* (m, 2H), 2.42-2.46* (m, 2H), 3.69* (s, 4H), 7.15-7.55* (m, 15H); ^{13}C NMR (125 MHz, $CDCl_3$) δ 14.1, 19.9, 31.3, 62.5, 127.0, 127.1, 128.2, 128.4, 129.2, 129.8, 138.3, 128.9, 173.3; 13.5*, 19.8*, 30.7*, 61.6*, 126.9*, 127.0*, 128.0*, 128.5*, 128.7*, 129.7*, 138.0*, 138.8*, 173.3*; IR (neat) ν 2959, 2930, 2871, 1603, 1494, 1453, 1317, 1028, 947, 751, 696; Ms (ESI): m/z = 343.2 $[M+H]^+$.

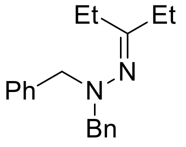

1c

Compound **1c**, colorless oil, yield: 63% (two steps from ketone). ^{1}H NMR (500 MHz, $CDCl_3$) δ 0.58 (t, J = 7.7 Hz, 3H), 0.90 (t, J = 7.5 Hz, 3H), 2.08 (q, J = 7.6 Hz, 2H), 2.15 (q, J = 7.7 Hz, 2H), 3.77 (s, 4H), 7.18-7.22 (m, 2H), 7.24-7.28 (m, 4H), 7.31 (d, J = 7.5 Hz, 4H); ^{13}C NMR (125 MHz, $CDCl_3$) δ 10.3, 11.8, 23.4, 28.4, 62.4, 126.9, 128.1, 129.8, 139.0, 178.8; IR (neat) ν 2969, 2934, 2827, 1635, 1494, 1454, 1374, 1028, 946, 745, 698; Ms (ESI): m/z = 281.2 $[M+H]^+$.

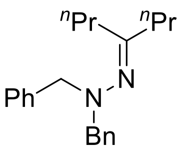

1d

Compound **1d**, colorless oil, yield: 65% (two steps from ketone). ^{1}H NMR (500 MHz, $CDCl_3$) δ 0.71-0.77 (m, 6H), 0.98-1.08 (m, 2H), 1.29-1.38 (m, 2H), 2.03-2.07 (m, 2H), 2.15-2.20 (m, 2H), 3.76 (s, 4H), 7.17-7.22 (m, 2H), 7.24-7.29 (m, 4H), 7.32 (d, J = 7.7 Hz, 4H); ^{13}C NMR (125 MHz, $CDCl_3$) δ 13.9, 14.5, 19.4, 20.6, 32.6, 37.9, 62.4, 127.0, 128.1, 129.7, 139.0, 176.9; IR (neat) ν 2959, 2930, 2871, 1634, 1494, 1453, 1348, 1028, 746, 697; Ms (ESI): m/z = 309.2 $[M+H]^+$.

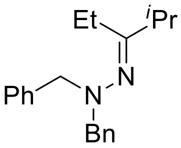

1e

Compound **1e**, colorless oil, yield: 60% (two steps from ketone). ^{1}H NMR (500 MHz, $CDCl_3$) δ 0.37 (d, J = 6.6 Hz, 6H), 0.98 (t, J = 7.8 Hz, 3H), 1.98 (q, J = 7.8 Hz, 2H), 3.50-3.59 (m, 1H), 3.77 (s, 4H), 7.17-7.22 (m, 2H), 7.24-7.28 (m, 4H), 7.31 (d, J = 7.6 Hz, 4H); ^{13}C NMR (125 MHz, $CDCl_3$) δ 12.9, 18.9, 23.7, 29.5, 62.8, 127.0, 128.1, 130.1, 139.1, 181.7; IR (neat) ν 2968, 2933, 2826, 1633, 1495, 1454, 1362, 1028, 946, 745, 698; Ms (ESI): m/z = 295.2 $[M+H]^+$.

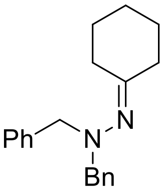

1f

Compound **1f**, colorless oil, yield: 71% (two steps from ketone). ^{1}H NMR (500 MHz, $CDCl_3$) δ 0.93-0.99 (m, 2H), 1.30-1.36 (m, 2H), 1.38-1.44 (m, 2H), 2.06-2.10 (m, 2H), 2.15-2.20 (m, 2H), 3.81 (s, 4H), 7.17-7.22 (m, 2H), 7.24-7.28 (m, 4H), 7.31 (d, J = 7.6 Hz, 4H); ^{13}C NMR (125 MHz, $CDCl_3$) δ 25.9, 26.1, 27.4, 29.6, 35.7, 62.4, 126.9, 128.1, 129.8, 139.0, 176.0; IR (neat) ν 2930, 2856, 1639, 1494, 1452, 1347, 1134, 1028, 745, 697; Ms (ESI): m/z = 293.2 $[M+H]^+$.

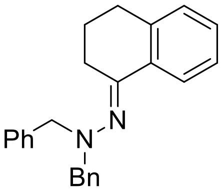

1g

Compound **1g**, yellow solid, yield: 83% (two steps from ketone). ^{1}H NMR (500 MHz, $CDCl_3$) δ 1.57-1.65 (m, 2H), 2.64-2.71 (m, 4H), 3.75-4.05 (m, 4H), 7.05 (d, J = 7.4 Hz, 1H), 7.10-7.30 (m, 8H), 7.33-7.37 (m, 4H), 8.14-8.18 (m, 1H); ^{13}C NMR (125 MHz, $CDCl_3$) δ 22.4, 28.1, 29.9, 61.8, 125.6, 126.2, 126.9, 128.2, 128.6, 129.4, 129.7, 133.0, 139.1, 140.3, 165.9; IR (neat) ν 2933, 2864,

2832, 1616, 1494, 1452, 1349, 1324, 1300, 1027, 952, 752, 730, 697; Ms (ESI): *m*/*z* = 341.2 $[M+H]^+$.

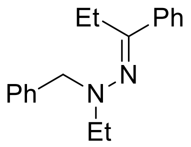

1i

Compound **1i**, yellow oil, yield: 67% (two steps from ketone). ^{1}H NMR (500 MHz, $CDCl_3$, a mixture of (Z/E) isomers in ratio 94:6, the minor one is marked with an *) δ 0.79 (t, *J* = 7.6 Hz, 3H), 1.03 (t, *J* = 7.2 Hz, 3H), 2.77-2.86 (m, 4H), 3.83 (s, 2H), 7.19-7.24 (m, 1H), 7.26-7.37 (m, 7H), 7.56-7.59 (m, 2H); 0.96* (t, *J* = 7.1 Hz, 3H), 1.08* (t, *J* = 7.0 Hz, 3H), 2.53-2.65* (m, 4H), 3.59* (s, 2H), 6.90-6.94* (m, 2H), 7.13-7.46* (m, 7H), 7.48-7.53* (m, 1H); ^{13}C NMR (125 MHz, $CDCl_3$) δ 11.3, 13.5, 22.6, 52.3, 63.1, 127.0, 127.2, 128.2, 128.4, 129.3, 129.8, 138.0, 138.9, 173.8; IR (neat) ν 2970, 2934, 2828, 1611, 1497, 1445, 1378, 1112, 1028, 916, 736, 695; Ms (ESI): *m*/*z* = 267.2 $[M+H]^+$.

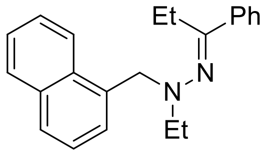

1j

Compound **1j**, yellow oil, yield: 64% (two steps from ketone). ^{1}H NMR (500 MHz, $CDCl_3$) δ 0.33 (t, *J* = 7.6 Hz, 3H), 1.10 (t, *J* = 7.2 Hz, 3H), 2.54 (q, *J* = 7.6 Hz, 2H), 2.96 (q, *J* = 7.2 Hz, 2H), 4.30 (s, 2H), 7.26-7.33 (m, 3H), 7.34-7.39 (m, 1H), 7.43-7.53 (m, 5H), 7.72 (d, *J* = 8.2 Hz, 1H), 7.81 (d, *J* = 8.1 Hz, 1H), 8.34 (d, *J* = 8.4 Hz, 1H); ^{13}C NMR (125 MHz, $CDCl_3$) δ 10.9, 13.5, 22.5, 53.0, 60.8, 124.7, 125.3, 125.5, 125.7, 127.2, 127.9, 128.1, 128.3, 128.5, 129.2, 132.8, 133.8, 134.7, 137.9, 174.7; IR (neat) ν 2957, 2925, 2852, 1652, 1496, 1464, 1362, 1049, 765, 753, 699, 668; Ms (ESI): *m*/*z* = 317.2 $[M+H]^+$.

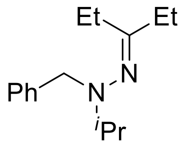

1l

Compound **1l**, colorless oil, yield: 72% (two steps from ketone). ^{1}H NMR (500 MHz, $CDCl_3$) δ 0.67 (t, *J* = 7.7 Hz, 3H), 0.97 (t, *J* = 7.6 Hz, 3H), 1.07 (d, *J* = 6.4 Hz, 6H), 2.13-2.24 (m, 4H), 2.84-2.93 (m, 1H), 3.74 (s, 2H), 7.15-7.19 (m, 1H), 7.21-7.27 (m, 4H); ^{13}C NMR (125 MHz, $CDCl_3$) δ 10.3, 11.9, 19.8, 23.3, 28.4, 55.5, 58.4, 126.6, 127.9, 130.0, 139.8, 178.4; IR (neat) ν 2967, 2934, 1635, 1456, 1375, 1168, 746, 697; Ms (ESI): *m*/*z* = 233.2 $[M+H]^+$.

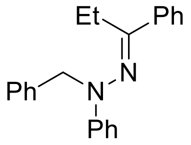

1o

Compound **1o**, yellow oil, yield: 80% (two steps from ketone). ^{1}H NMR (500 MHz, $CDCl_3$) δ 0.78 (t, J = 7.6 Hz, 3H), 2.67 (q, J = 7.6 Hz, 2H), 4.71 (s, 2H), 6.87-6.91 (m, 1H), 6.95 (d, J = 8.4 Hz, 2H), 7.20-7.41 (m, 10H), 7.72-7.75 (m, 2H); ^{13}C NMR (125 MHz, $CDCl_3$) δ 11.1, 22.9, 62.4, 117.0, 120.8, 127.0, 127.3, 128.3, 128.4, 128.5, 129.0, 129.8, 137.1, 139.0, 151.4, 173.7; IR (neat) v 2971, 2935, 2876, 1595, 1493, 1452, 1319, 1198, 1028, 916, 753, 694; Ms (ESI): m/z = 315.2 $[M+H]^+$.

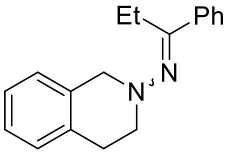

1p

Compound **1p**, yellow oil, yield: 66% (three steps from 1,2,3,4-tetrahydroisoquinoline). ^{1}H NMR (500 MHz, $CDCl_3$, a mixture of (Z/E) isomers in ratio 91:9, the minor one is marked with an *) δ 1.10 (t, J = 7.6 Hz, 3H), 2.99 (q, J = 7.6 Hz, 2H), 3.05-3.12 (m, 4H), 3.99 (s, 2H), 7.07-7.10 (m, 1H), 7.14-7.21 (m, 3H), 7.38-7.42 (m, 3H), 7.69-7.74 (m, 2H); 1.07* (t, J = 7.5 Hz, 3H), 2.61* (q, J = 7.5 Hz, 2H), 2.72* (t, J = 5.8 Hz, 2H), 2.93* (t, J = 5.9 Hz, 2H), 3.92* (s, 2H), 6.92-6.97* (m, 1H), 7.01-7.05* (m, 1H), 7.27-7.32* (m, 1H), 7.33-7.37* (m, 2H), 7.46-7.49* (m, 2H), 7.69-7.74* (m, 2H); ^{13}C NMR (125 MHz, $CDCl_3$) δ 12.2, 22.2, 29.0, 54.0, 58.0, 125.9, 126.4, 127.0, 127.3, 128.5, 128.6, 129.5, 134.3, 134.6, 137.8, 171.2; 11.9*, 28.8*, 32.4*, 52.6*, 57.3*, 125.7*, 126.1*, 126.9*, 127.3*, 128.3*, 128.3*, 128.4*, 134.5*, 134.9*, 137.4*, 165.7*; IR (neat) v 2966, 2929, 2805, 1607, 1496, 1455, 1365, 1017, 938, 770, 744, 694 cm^{-1}; Ms (ESI): m/z = 265.2 $[M+H]^+$.

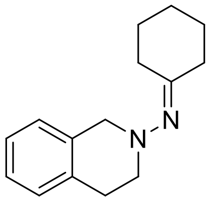

4a

Compound **4a**, pale yellow solid, yield: 60% (three steps from 1,2,3,4-tetrahydroisoquinoline). ^{1}H NMR (500 MHz, $CDCl_3$) δ 1.61-1.77 (m, 6H), 2.32 (t, J = 6.5 Hz, 2H), 2.59 (t, J = 5.9 Hz, 2H), 2.93-3.03 (m, 4H), 3.86 (s, 2H), 7.04 (d, J = 6.9 Hz, 1H), 7.10-7.18 (m, 3H); ^{13}C NMR (125 MHz, $CDCl_3$) δ 26.2, 26.9, 27.7, 29.0, 29.1, 36.2, 53.6, 57.7, 125.8, 126.4, 127.0, 128.5, 134.2, 134.6, 171.8; IR (neat) v 2928, 2855, 2803, 1636, 1497, 1468, 1367, 1023, 940, 744 cm^{-1}; Ms (ESI): m/z = 229.2 $[M+H]^+$.

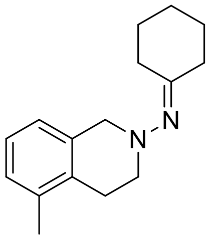

4b

Compound **4b**, yellow oil, yield: 51% (three steps from 1,2,3,4-tetrahydroisoquinoline). ^{1}H NMR (500 MHz, $CDCl_3$) δ 1.61-1.78 (m, 6H), 2.23 (s, 3H), 2.31 (t, J = 6.3 Hz, 2H), 2.59 (t, J = 5.7 Hz, 2H), 2.86 (t, J = 6.0 Hz, 2H), 2.99 (t, J = 6.0 Hz, 2H), 3.84 (s, 2H), 6.90 (d, J = 7.4 Hz, 1H), 7.00-7.08 (m, 2H); ^{13}C NMR (125 MHz, $CDCl_3$) δ 19.2, 26.2, 26.8, 27.1, 27.7, 29.0, 36.2, 53.8, 58.2, 124.7, 125.7, 127.8, 132.8, 134.5, 136.3, 171.7; IR (neat) ν 2926, 2855, 2807, 1635, 1503, 1452, 1366, 1024, 943, 760, 724 cm^{-1}; Ms (ESI): m/z = 243.2 $[M+H]^{+}$.

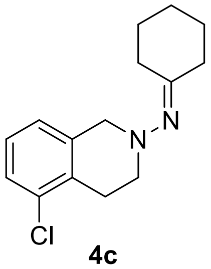

4c

Compound **4c**, yellow oil, yield: 63% (three steps from 1,2,3,4-tetrahydroisoquinoline). ^{1}H NMR (500 MHz, $CDCl_3$) δ 1.61-1.77 (m, 6H), 2.30 (t, J = 6.3 Hz, 2H), 2.56 (t, J = 5.8 Hz, 2H), 2.90-2.96 (m, 4H), 3.80 (s, 2H), 7.01-7.06 (m, 2H), 7.11 (dd, J = 8.2, 1.6 Hz, 1H); ^{13}C NMR (125 MHz, $CDCl_3$) δ 26.1, 26.8, 27.6, 28.4, 29.0, 36.1, 53.4, 57.4, 126.5, 126.8, 129.9, 131.4, 132.7, 136.5, 172.1; IR (neat) ν 2928, 2855, 2811, 1636, 1486, 1427, 1362, 1088, 1022, 940, 890, 801cm^{-1}; Ms (ESI): m/z = 263.1 $[M+H]^{+}$.

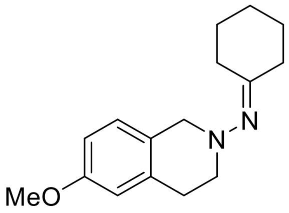

4d

Compound **4d**, yellow solid, yield: 45% (three steps from 1,2,3,4-tetrahydroisoquinoline). ^{1}H NMR (500 MHz, $CDCl_3$) δ 1.60-1.68 (m, 4H), 1.70-1.76 (m, 2H), 2.29-2.33 (m, 2H), 2.56-2.60 (m, 2H), 2.91-2.94 (m, 2H), 2.96-3.00 (m, 2H), 3.77-3.79 (m, 5H), 6.66 (d, J = 2.5 Hz, 1H), 6.71 (dd, J = 8.4, 2.7 Hz, 1H), 6.96 (d, J = 8.4 Hz, 1H); ^{13}C NMR (125 MHz, $CDCl_3$) δ 26.2, 26.9, 27.7, 29.0, 29.3, 36.2, 53.6, 55.4, 57.3, 112.2, 113.2, 126.8, 127.9, 135.4, 158.2, 171.6; IR (neat) ν 2930, 2855, 2832, 1615, 1505, 1448, 1270, 1158, 1038, 945, 810 cm^{-1}; Ms (ESI): m/z = 259.2 $[M+H]^{+}$.

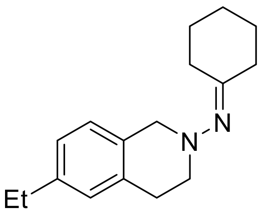

4e

Compound **4e**, yellow oil, yield: 55% (three steps from 1,2,3,4-tetrahydroisoquinoline). ^{1}H NMR (500 MHz, $CDCl_3$) δ 1.22 (t, J = 7.6 Hz, 3H), 1.60-1.77 (m, 6H), 2.28-2.33 (m, 2H), 2.56-2.62 (m, 4H), 2.92-3.02 (m, 4H), 3.81 (s, 2H), 6.94-7.00 (m, 3H); ^{13}C NMR (125 MHz, $CDCl_3$) δ 15.9, 26.2, 26.8, 27.7, 28.7, 29.0, 29.1, 36.2, 53.7, 57.6, 125.5, 126.9, 127.9, 131.8, 134.0, 142.4, 171.6; IR (neat) ν 2929, 2856, 2809, 1636, 1506, 1448, 1366, 1023, 943, 880, 819 cm^{-1}; Ms (ESI): m/z = 257.2 [M+H]$^{+}$.

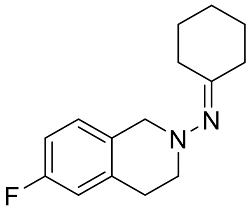

4f

Compound **4f**, yellow oil, yield: 64% (three steps from 1,2,3,4-tetrahydroisoquinoline). ^{1}H NMR (500 MHz, $CDCl_3$) δ 1.61-1.78 (m, 6H), 2.31 (t, J = 6.5 Hz, 2H), 2.57 (t, J = 6.1 Hz, 2H), 2.90-3.01 (m, 4H), 3.80 (s, 2H), 6.80-6.86 (m, 2H), 6.97-7.02 (m, 1H); ^{13}C NMR (125 MHz, $CDCl_3$) δ 26.1, 26.9, 27.6, 29.0, 29.1, 36.2, 53.2, 57.3, 113.0 ($^{2}J_{CF}$ = 21.4 Hz), 114.8 ($^{2}J_{CF}$ = 20.6 Hz), 128.3 ($^{3}J_{CF}$ = 7.9 Hz), 130.2, 136.3, 160.1 ($^{1}J_{CF}$ = 242Hz), 171.9; IR (neat) ν 2928, 2855, 2812, 1617, 1501, 1448, 1367, 1238, 1141, 922, 864, 807 cm^{-1}; Ms (ESI): m/z = 247.2 [M+H]$^{+}$.

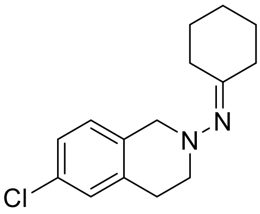

4g

Compound **4g**, yellow oil, yield: 59% (three steps from 1,2,3,4-tetrahydroisoquinoline). ^{1}H NMR (500 MHz, $CDCl_3$) δ 1.61-1.77 (m, 6H), 2.31 (t, J = 6.5 Hz, 2H), 2.57 (t, J = 6.0 Hz, 2H), 2.90-2.99 (m, 4H), 3.80 (s, 2H), 6.97 (d, J = 8.0 Hz, 1H), 7.08-7.13 (m, 2H); ^{13}C NMR (125 MHz, $CDCl_3$) δ 26.1, 26.9, 27.6, 28.8, 29.0, 36.1, 53.2, 57.3, 126.0, 126.2, 128.3, 128.4, 131.9, 136.1, 172.1; IR (neat) ν 2927, 2854, 2813, 1645, 1489, 1448, 1365, 1088, 1022, 942, 877, 808 cm^{-1}; Ms (ESI): m/z = 263.1 [M+H]$^{+}$.

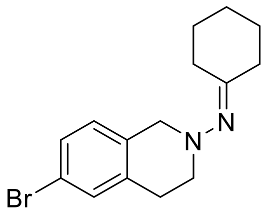

4h

Compound **4h**, yellow oil, yield: 48% (three steps from 1,2,3,4-tetrahydroisoquinoline). ^{1}H NMR (500 MHz, $CDCl_3$) δ 1.59-1.76 (m, 6H), 2.30 (t, J = 6.3 Hz, 2H), 2.56 (t, J = 5.7 Hz, 2H), 2.89-2.99 (m, 4H), 3.78 (s, 2H), 6.91 (d, J = 8.1 Hz, 1H), 7.22-7.26 (m, 2H); ^{13}C NMR (125 MHz, $CDCl_3$) δ 26.1, 26.9, 27.6, 28.8, 29.0, 36.1, 53.1, 57.3, 128.6, 128.6, 128.9, 131.3, 133.6, 136.6, 172.1; IR (neat) ν 2926, 2854, 2813, 1636, 1485, 1447, 1364, 1078, 1022, 941, 866, 806 cm^{-1}; Ms (ESI): m/z = 307.1 $[M+H]^+$.

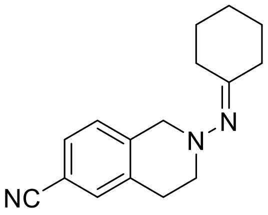

4i

Compound **4i**, yellow oil, yield: 81% (from **4g**). ^{1}H NMR (500 MHz, $CDCl_3$) δ 1.60-1.78 (m, 6H), 2.28-2.32 (m, 2H), 2.54-2.58 (m, 2H), 2.92-3.03 (m, 4H), 3.89 (s, 2H), 7.13 (d, J = 7.8 Hz, 1H), 7.39-7.43 (m, 2H); ^{13}C NMR (125 MHz, $CDCl_3$) δ 26.1, 26.9, 27.6, 28.5, 29.0, 36.1, 52.9, 57.6, 110.2, 119.2, 127.9, 129.4, 132.3, 135.8, 140.3, 172.5; IR (neat) ν 2928, 2854, 2815, 2227, 1636, 1499, 1447, 1372, 1080, 1026, 947, 908, 821 cm^{-1}; Ms (ESI): m/z = 254.2 $[M+H]^+$.

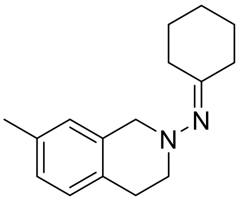

4j

Compound **4j**, yellow oil, yield: 55% (three steps from 1,2,3,4-tetrahydroisoquinoline). ^{1}H NMR (500 MHz, $CDCl_3$) δ 1.58-1.77 (m, 6H), 2.28-2.33 (m, 5H), 2.55-2.60 (m, 2H), 2.91-2.97 (m, 4H), 3.81 (s, 2H), 6.86 (s, 1H), 6.96 (d, J = 7.8 Hz, 1H), 7.01 (d, J = 7.7 Hz, 1H); ^{13}C NMR (125 MHz, $CDCl_3$) δ 21.1, 26.2, 26.9, 27.7, 28.6, 29.0, 36.2, 53.8, 57.8, 127.3, 127.5, 128.4, 131.6, 134.4, 137.3, 171.7; IR (neat) ν 2923, 2854, 2812, 1636, 1506, 1447, 1362, 1083, 1022, 940, 872, 801 cm^{-1}; Ms (ESI): m/z = 243.2 $[M+H]^+$.

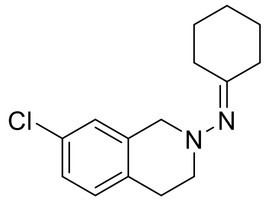

4k

Compound **4k**, yellow oil, yield: 62% (three steps from 1,2,3,4-tetrahydroisoquinoline). ^{1}H NMR (500 MHz, $CDCl_3$) δ 1.58-1.77 (m, 6H), 2.31 (t, J = 6.5 Hz, 2H), 2.57 (t, J = 6.0 Hz, 2H), 2.90-2.97 (m, 4H), 3.80 (s, 2H), 7.01-7.06 (m, 2H), 7.11 (d, J = 8.2 Hz, 1H); ^{13}C NMR (125 MHz, $CDCl_3$) δ 26.1, 26.9, 27.6, 28.4, 29.0, 36.1, 53.4, 57.4, 126.5, 126.8, 129.9, 131.4, 132.7, 136.5, 172.1; IR (neat) ν 2927, 2854, 1636, 1486, 1448, 1361, 1088, 1022, 940, 890, 801 cm^{-1}; Ms (ESI): m/z = 263.1 [M+H]$^+$.

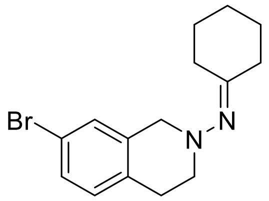

4l

Compound **4l**, yellow oil, yield: 50% (three steps from 1,2,3,4-tetrahydroisoquinoline). ^{1}H NMR (500 MHz, $CDCl_3$) δ 1.59-1.77 (m, 6H), 2.31 (t, J = 6.3 Hz, 2H), 2.56 (t, J = 6.0 Hz, 2H), 2.93 (brs, 4H), 3.80 (s, 2H), 6.99 (d, J = 8.2 Hz, 1H), 7.19 (s, 1H), 7.25 (d, J = 8.2 Hz, 1H); ^{13}C NMR (125 MHz, $CDCl_3$) δ 26.1, 26.9, 27.6, 28.4, 29.0, 36.1, 53.3, 57.3, 119.4, 129.4, 129.8, 130.2, 133.3, 136.9, 172.1; IR (neat) ν 2926, 2854, 1635, 1483, 1447, 1362, 1021, 940, 878, 798 cm^{-1}; Ms (ESI): m/z = 307.1 [M+H]$^+$.

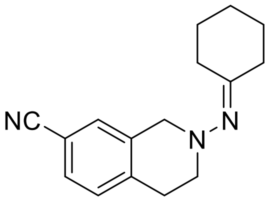

4m

Compound **4m**, yellow oil, yield: 84% (from **4l**). ^{1}H NMR (500 MHz, $CDCl_3$) δ 1.60-1.77 (m, 6H), 2.31 (t, J = 6.0 Hz, 2H), 2.56 (t, J = 6.0 Hz, 2H), 2.95 (t, J = 5.9 Hz, 2H), 3.03 (t, J = 5.9 Hz, 2H), 3.84 (s, 2H), 7.21 (d, J = 7.9 Hz, 1H), 7.34 (s, 1H), 7.41 (d, J = 7.9 Hz, 1H); ^{13}C NMR (125 MHz, $CDCl_3$) δ 26.1, 26.8, 27.6, 29.0, 29.1, 36.1, 52.8, 57.1, 109.7, 119.2, 129.4, 129.9, 130.8, 136.1, 140.1, 172.6; IR (neat) ν 2929, 2855, 2227, 1636, 1498, 1448, 1363, 1024, 941, 834, 808 cm^{-1}; Ms (ESI): m/z = 254.2 [M+H]$^+$.

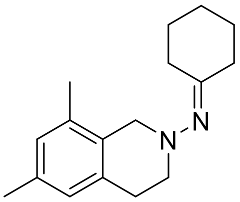

4n

Compound **4n**, yellow oil, yield: 51% (three steps from 1,2,3,4-tetrahydroisoquinoline). ^{1}H NMR (500 MHz, $CDCl_3$) δ 1.62-1.79 (m, 6H), 2.17 (s, 3H), 2.27 (s, 3H), 2.31-2.36 (m, 2H), 2.57-2.62 (m, 2H), 2.87-2.99 (m, 4H), 3.77 (s, 2H), 6.80-6.83 (m, 2H); ^{13}C NMR (125 MHz, $CDCl_3$) δ 18.7, 21.0, 26.2, 26.9, 27.6, 29.0, 29.4, 36.2, 53.2, 55.7, 126.7, 128.3, 130.1, 133.9, 134.9, 135.5, 171.7; IR (neat) *v* 2928, 2856, 2809, 1635, 1447, 1363, 1009, 964, 897, 848 cm^{-1}; Ms (ESI): *m*/*z* = 257.2 $[M+H]^+$.

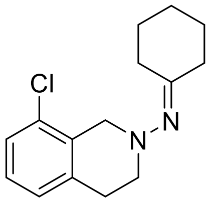

4o

Compound **4o**, yellow oil, yield: 59% (three steps from 1,2,3,4-tetrahydroisoquinoline). ^{1}H NMR (500 MHz, $CDCl_3$) δ 1.59-1.78 (m, 6H), 2.33 (t, *J* = 6.5 Hz, 2H), 2.58 (t, *J* = 5.9 Hz, 2H), 2.94 (t, *J* = 5.8 Hz, 2H), 3.00 (t, *J* = 5.5 Hz, 2H), 3.86 (s, 2H), 7.03 (d, *J* = 7.6 Hz, 1H), 7.07-7.11 (m, 1H), 7.18 (d, *J* = 7.8 Hz, 1H); ^{13}C NMR (125 MHz, $CDCl_3$) δ 26.1, 26.9, 27.7, 29.0, 29.2, 36.1, 52.9, 55.4, 126.7, 127.0, 127.1, 132.6, 132.7, 136.8, 172.2; IR (neat) *v* 2927, 2854, 2818, 1636, 1447, 1363, 1180, 1026, 956, 769 cm^{-1}; Ms (ESI): *m*/*z* = 263.1 $[M+H]^+$.

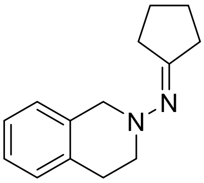

4p

Compound **4p**, yellow oil, yield: 67% (three steps from 1,2,3,4-tetrahydroisoquinoline). ^{1}H NMR (500 MHz, $CDCl_3$) δ 1.73-1.83 (m, 4H), 2.41-2.51 (m, 4H), 2.97-3.06 (m, 4H), 3.92 (s, 2H), 7.04 (d, *J* = 7.5 Hz, 1H), 7.10-7.17 (m, 3H); ^{13}C NMR (125 MHz, $CDCl_3$) δ 24.3, 25.0, 29.0, 29.8, 33.7, 53.2, 57.1, 125.8, 126.4, 127.0, 128.5, 134.2, 134.6, 177.8; IR (neat) *v* 2959, 2887, 2804, 1653, 1497, 1452, 1366, 1026, 939, 744 cm^{-1}; Ms (ESI): *m*/*z* = 215.2 $[M+H]^+$.

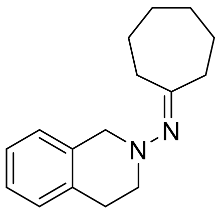

4q

Compound **4q**, yellow oil, yield: 62% (three steps from 1,2,3,4-tetrahydroisoquinoline). ^{1}H NMR (500 MHz, $CDCl_3$) δ 1.53-1.72 (m, 8H), 2.46-2.50 (m, 2H), 2.70-2.74 (m, 2H), 2.92-3.02 (m, 4H), 3.83 (s, 2H), 7.04 (d, J = 7.1 Hz, 1H), 7.10-7.17 (m, 3H); ^{13}C NMR (125 MHz, $CDCl_3$) δ 25.2, 27.4, 29.1, 30.1, 30.6, 31.3, 37.2, 53.2, 57.3, 125.8, 126.4, 127.0, 128.6, 134.3, 134.7, 176.0; IR (neat) v 2925, 2855, 2823, 1635, 1587, 1454, 1366, 1017, 939, 744 cm^{-1}; Ms (ESI): m/z = 243.2 $[M+H]^+$.

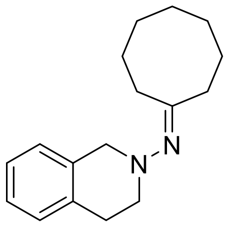

4r

Compound **4r**, yellow oil, yield: 59% (three steps from 1,2,3,4-tetrahydroisoquinoline). ^{1}H NMR (500 MHz, $CDCl_3$) δ 1.43-1.58 (m, 6H), 1.76-1.84 (m, 4H), 2.38-2.42 (m, 2H), 2.55-2.58 (m, 2H), 2.92 (t, J = 5.9 Hz, 2H), 3.01 (t, J = 5.9 Hz, 2H), 3.80 (s, 2H), 7.05 (d, J = 7.2 Hz, 1H), 7.11-7.17 (m, 3H); ^{13}C NMR (125 MHz, $CDCl_3$) δ 24.7, 25.3, 26.9, 27.0, 27.6, 29.1, 30.0, 35.7, 52.9, 57.2, 125.8, 126.3, 127.0, 128.6, 134.4, 134.8, 178.0; IR (neat) v 2926, 2854, 2804, 1634, 1497, 1455, 1367, 1022, 940, 743 cm^{-1}; Ms (ESI): m/z = 257.2 $[M+H]^+$.

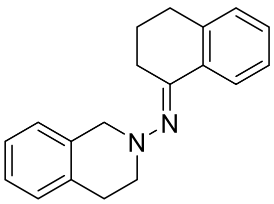

4s

Compound **4s**, yellow solid, yield: 67% (three steps from 1,2,3,4-tetrahydroisoquinoline). ^{1}H NMR (500 MHz, $CDCl_3$) δ 1.90-1.97 (m, 2H), 2.85-2.94 (m, 4H), 3.04-3.15 (m, 4H), 4.06 (s, 2H), 7.08 (d, J = 7.0 Hz, 1H), 7.13-7.24 (m, 5H), 7.27-7.31 (m, 1H), 8.21 (d, J = 7.9 Hz, 1H); ^{13}C NMR (125 MHz, $CDCl_3$) δ 22.8, 27.6, 28.9, 30.0, 53.3, 57.5, 125.3, 125.9, 126.3, 126.4, 127.0, 128.6, 128.8, 129.7, 133.1, 134.4, 134.7, 140.1, 162.7; IR (neat) v 2930, 2830, 2805, 1612, 1585, 1452, 1366, 1020, 939, 762, 746, 730 cm^{-1}; Ms (ESI): m/z = 277.2 $[M+H]^+$.

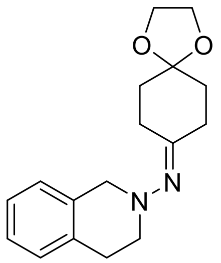

4t

Compound **4t**, white solid, yield: 62% (three steps from 1,2,3,4-tetrahydroisoquinoline). ^{1}H NMR (500 MHz, $CDCl_3$) δ 1.79 (t, J = 6.8 Hz, 2H), 1.88 (t, J = 6.7 Hz, 2H), 2.51 (t, J = 6.8 Hz, 2H), 2.76 (t, J = 6.8 Hz, 2H), 2.94-3.02 (m, 4H), 3.86 (s, 2H), 3.99 (brs, 4H), 7.03 (d, J = 7.4 Hz, 1H), 7.10-7.17 (m, 3H); ^{13}C NMR (125 MHz, $CDCl_3$) δ 32.6, 34.0, 34.1, 34.8, 38.3, 53.6, 57.6, 64.6 (2C), 108.1, 125.8, 126.4, 128.5, 134.1, 134.4, 169.2; IR (neat) ν 2953, 2885, 2805, 1717, 1635, 1496, 1436, 1363, 1124, 1087, 1031, 940, 908, 747 cm^{-1}; Ms (ESI): m/z = 287.2 [M+H]$^+$.

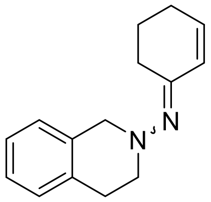

4u

Compound **4u**, yellow oil, yield: 50% (three steps from 1,2,3,4-tetrahydroisoquinoline). ^{1}H NMR (500 MHz, $CDCl_3$, a mixture of (Z/E) isomers in ratio 65:35, the minor one is marked with an *) δ 1.77-1.83 (m, 2H), 2.21-2.29 (m, 2H), 2.71 (t, J = 6.5 Hz, 2H), 2.99-3.06 (m, 4H), 3.93 (s, 2H), 6.22 (d, J = 10.1 Hz, 1H), 6.31-6.36 (m, 1H), 7.02-7.07 (m, 1H), 7.11-7.18 (m, 3H); 1.86-1.92* (m, 2H), 2.21-2.29* (m, 2H), 2.49* (t, J = 6.5 Hz, 2H), 2.99-3.06* (m, 4H), 3.91* (s, 2H) 6.33-6.38* (m, 1H), 6.81* (d, J = 10.3 Hz, 1H), 7.02-7.07* (m, 1H), 7.11-7.18* (m, 3H); ^{13}C NMR (125 MHz, $CDCl_3$) δ 22.0, 25.7, 26.4, 29.0, 53.2, 57.3, 125.8, 126.4, 127.0, 128.5, 128.6, 134.2, 134.5, 138.3, 164.9; 22.7*, 26.6*, 29.0*, 31.9*, 54.0*, 58.1*, 120.6*, 125.8*, 126.4*, 126.9*, 128.5*, 134.3*, 134.6*, 140.8*, 163.1*; IR (neat) ν 3023, 2926, 2826, 2804, 1625, 1584, 1497, 1454, 1365, 1026, 938, 744 cm^{-1}; Ms (ESI): m/z = 227.2 [M+H]$^+$.

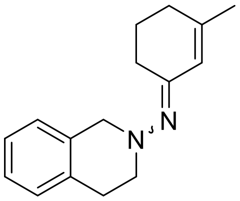

4v

Compound **4v**, yellow oil, yield: 57% (three steps from 1,2,3,4-tetrahydroisoquinoline). ^{1}H NMR (500 MHz, $CDCl_3$, a mixture of (Z/E) isomers in ratio 74:26, the minor one is marked with an *) δ 1.76-1.89 (m, 5H), 2.13-2.18 (m, 2H), 2.65 (t, J = 6.5 Hz, 2H), 2.98-3.02 (m, 4H), 3.90 (s, 2H), 6.04 (s, 1H), 7.02-7.06 (m, 1H), 7.10-7.17 (m, 3H); 1.76-1.89* (m, 5H), 2.17-2.21* (m, 2H), 2.41*

(t, J = 6.5 Hz, 2H), 3.01-3.04* (m, 4H), 3.89* (s, 2H), 6.62* (s, 1H), 7.02-7.06* (m, 1H), 7.10-7.17* (m, 3H); ^{13}C NMR (125 MHz, $CDCl_3$) δ 22.2, 24.3, 25.7, 29.0, 30.9, 53.3, 57.4, 124.1, 125.8, 126.4, 127.0, 128.6, 134.3, 134.6, 148.5, 165.9; 22.8*, 24.6*, 29.1*, 31.4*, 31.8*, 53.9*, 58.0*, 116.8*, 125.8*, 126.4*, 127.0*, 128.6*, 134.4*, 134.8*, 151.6*, 163.8*; IR (neat) ν 2926, 2823, 2804, 1635, 1586, 1455, 1366, 1017, 939, 744 cm^{-1}; Ms (ESI): m/z = 241.2 [M+H]$^+$.

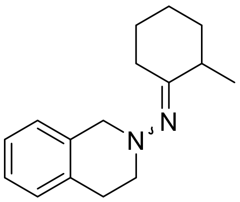

4w

Compound **4w**, yellow oil, yield: 56% (three steps from 1,2,3,4-tetrahydroisoquinoline). ^{1}H NMR (500 MHz, $CDCl_3$, a mixture of (Z/E) isomers in ratio 75:25, the minor one is marked with an *) δ 1.15 (d, J = 7.0 Hz, 3H), 1.45-1.80 (m, 5H), 1.83-1.90 (m, 1H), 2.42-2.50 (m, 2H), 2.66-2.73 (m, 1H), 2.90-3.03 (m, 4H), 3.83 (s, 2H), 7.02-7.06 (m, 1H), 7.10-7.17 (m, 3H); 1.16* (d, J = 7.0 Hz, 3H), 1.45-1.80* (m, 5H), 2.24-2.30* (m, 1H), 2.35-2.42* (m, 1H), 2.42-2.50* (m, 2H), 2.90-3.03* (m, 4H), 3.85* (s, 2H), 7.02-7.17* (m, 4H); ^{13}C NMR (125 MHz, $CDCl_3$) δ 18.0, 23.4, 27.0, 27.2, 29.1, 35.1, 39.1, 53.7, 57.8, 125.8, 126.3, 127.0, 128.5, 134.3, 134.8, 174.2; 17.3*, 21.2*, 28.1*, 29.1*, 30.8*, 32.0*, 33.1*, 54.0*, 58.1*, 125.8*, 126.0*, 126.4*, 128.6*, 134.2*, 134.7*, 177.0*; IR (neat) ν 2926, 2854, 2804, 1635, 1455, 1367, 1022, 940, 743 cm^{-1}; Ms (ESI): m/z = 243.2 [M+H]$^+$.

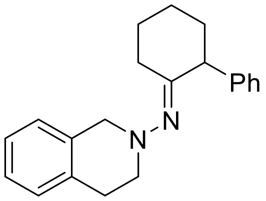

4x

Compound **4x**, yellow solid, yield: 55% (three steps from 1,2,3,4-tetrahydroisoquinoline). ^{1}H NMR (500 MHz, $CDCl_3$) δ 1.55-1.84 (m, 5H), 1.95-2.04 (m, 1H), 2.08-2.16 (m, 1H), 2.40-2.48 (m, 1H), 3.00-3.09 (m, 4H), 3.76-3.79 (m, 1H), 3.89-3.97 (m, 2H), 7.06 (d, J = 7.3 Hz, 1H), 7.11-7.17 (m, 3H), 7.19-7.25 (m, 1H), 7.31-7.34 (m, 4H); ^{13}C NMR (125 MHz, $CDCl_3$) δ 22.3, 26.9, 27.2, 29.0, 31.3, 48.1, 53.7, 57.8, 125.8, 126.2, 126.4, 127.0, 127.7, 128.5, 128.6, 134.2, 134.6, 141.1, 172.5; IR (neat) ν 2931, 2858, 2805, 1715, 1634, 1496, 1447, 1366, 1022, 939, 746, 698 cm^{-1}; Ms (ESI): m/z = 305.2 [M+H]$^+$.

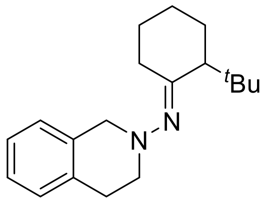

4y

Compound **4y**, yellow oil, yield: 52% (three steps from 1,2,3,4-tetrahydroisoquinoline). ^{1}H NMR (500 MHz, $CDCl_3$) δ 1.04 (s, 9H), 1.45-1.53 (m, 1H), 1.57- 1.88 (m, 5H), 2.13 (t, J = 6.2 Hz, 1H), 2.41-2.47 (m, 1H), 2.76-2.82 (m, 1H), 2.95-3.03 (m, 4H), 3.82-3.92 (m, 2H), 7.04 (d, J = 7.1 Hz, 1H), 7.10-7.17 (m, 3H); ^{13}C NMR (125 MHz, $CDCl_3$) δ 24.3, 26.9, 28.3, 28.9, 29.4 (2C), 33.8, 53.5, 53.8, 58.0, 125.7, 126.3, 127.0, 128.5, 134.3, 134.8, 172.9; IR (neat) v 2930, 2861, 2804, 1635, 1454, 1365, 1021, 940, 743 cm^{-1}; Ms (ESI): m/z = 285.3 $[M+H]^+$.

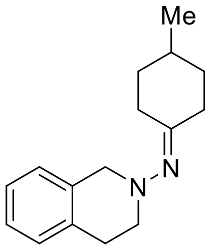

4z

Compound **4z**, yellow oil, yield: 62% (three steps from 1,2,3,4-tetrahydroisoquinoline). ^{1}H NMR (500 MHz, $CDCl_3$) δ 0.96 (d, J = 6.6 Hz, 3H), 1.10-1.35 (m, 2H), 1.66-1.76 (m, 1H), 1.81-1.95 (m, 3H), 2.22 (td, J = 13.5, 5.0 Hz, 1H), 2.42-2.49 (m, 1H), 2.93-3.02 (m, 4H), 3.26-3.32 (m, 1H), 3.80-3.90 (m, 2H), 7.04 (d, J = 7.6 Hz, 1H), 7.10-7.17 (m, 3H); ^{13}C NMR (125 MHz, $CDCl_3$) δ 21.6, 28.1, 29.0, 32.2, 34.8, 35.4, 35.6, 53.6, 57.8, 125.8, 126.4, 127.0, 128.6, 134.2, 134.6, 171.6; IR (neat) v 2923, 2852, 2805, 1636, 1497, 1455, 1367, 1023, 939, 743 cm^{-1}; Ms (ESI): m/z = 243.2 $[M+H]^+$.

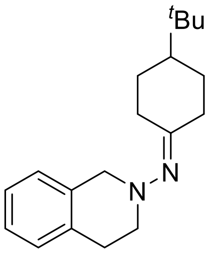

4aa

Compound **4aa**, white solid, yield: 60% (three steps from 1,2,3,4-tetrahydroisoquinoline). ^{1}H NMR (500 MHz, $CDCl_3$) δ 0.89 (s, 9H), 1.13-1.36 (m, 3H), 1.80 (td, J = 13.7, 5.4 Hz, 1H), 1.91-2.04 (m, 2H), 2.18 (td, J = 13.5, 5.1 Hz, 1H), 2.51 (dd, J = 13.9, 2.4 Hz, 1H), 2.92-3.03 (m, 4H), 3.41 (dd, J = 14.1, 1.6 Hz, 1H), 3.80-3.90 (m, 2H), 7.04 (d, J = 7.3 Hz, 1H), 7.10-7.17 (m, 3H); ^{13}C NMR (125 MHz, $CDCl_3$) δ 27.5, 27.7, 28.3, 28.6, 29.1, 32.6, 35.9, 47.8, 53.7, 57.8, 125.8, 126.4, 127.0, 128.5, 134.2, 134.6, 171.7; IR (neat) v 2950, 2865, 2804, 1636. 1496, 1455, 1365,

1025, 940, 743 cm^{-1}; Ms (ESI): *m*/*z* = 285.2 [M+H]$^+$.

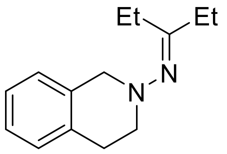

4ab

Compound **4ab**, yellow oil, yield: 64% (three steps from 1,2,3,4-tetrahydroisoquinoline). ^{1}H NMR (500 MHz, $CDCl_3$) δ 1.07-1.16 (m, 6H), 2.31 (q, *J* = 7.5 Hz, 2H), 2.52 (q, *J* = 7.6 Hz, 2H), 2.92 (t, *J* = 6.0 Hz, 2H), 3.01 (t, *J* = 5.9 Hz, 2H), 3.81 (s, 2H), 7.04 (d, *J* = 7.1 Hz, 1H), 7.10-7.17 (m, 3H); ^{13}C NMR (125 MHz, $CDCl_3$) δ 11.3, 11.8, 23.0, 29.1 (2C), 53.7, 57.8, 125.8, 126.3, 127.0, 128.5, 134.3, 134.8, 176.1; IR (neat) *v* 2969, 2932, 2804, 1635, 1497, 1456, 1366, 1020, 938, 744 cm^{-1}; Ms (ESI): *m*/*z* = 217.2 [M+H]$^+$.

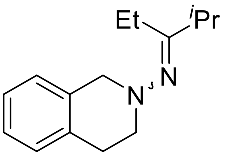

4ac

Compound **4ac**, yellow oil, yield: 60% (three steps from 1,2,3,4-tetrahydroisoquinoline). ^{1}H NMR (500 MHz, $CDCl_3$, a mixture of (Z/E) isomers in ratio 77:23, the minor one is marked with an *) δ 1.05-1.18 (m, 9H), 2.43 (q, *J* = 7.5 Hz, 2H), 2.56-2.65 (m, 1H), 2.87-2.92 (m, 2H), 2.98-3.03 (m, 2H), 3.78 (s, 2H), 7.04 (d, *J* = 6.6 Hz, 1H), 7.11-7.18 (m, 3H); 1.05-1.18* (m, 9H), 2.26* (q, *J* = 7.5 Hz, 2H), 2.45-2.50* (m, 1H), 2.87-2.92* (m, 2H), 2.98-3.03* (m, 2H), 3.78* (s, 2H), 7.04* (d, *J* = 6.6 Hz, 1H), 7.11-7.18* (m, 3H); ^{13}C NMR (125 MHz, $CDCl_3$) δ 12.1, 20.6, 22.2, 29.1, 35.0, 53.6, 57.8, 125.7, 126.3, 126.9, 128.5, 134.4, 134.9, 179.5; 12.7*, 18.5*, 20.1*, 24.6*, 29.1*, 53.9*, 58.0*, 125.7*, 126.3*, 127.0*, 128.5*, 134.4*, 134.9*, 179.2*; IR (neat) *v* 2964, 2928, 2804, 1635, 1456, 1364, 1018, 939, 742 cm^{-1}; Ms (ESI): *m*/*z* = 231.2 [M+H]$^+$.

Analytical Data for Products

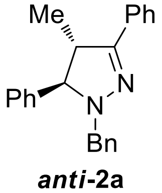

***anti*-2a**

Compound **2a**, yellow oil, yield: 86% (*anti* : *syn* = 6.8 : 1). ***anti*-2a**: ^{1}H NMR (500 MHz, $CDCl_3$) δ 1.20 (dd, *J* = 6.9, 0.7 Hz, 3H), 3.35-3.46 (m, 1H), 3.79 (d, *J* = 11.7 Hz, 1H), 3.96 (d, *J* = 14.3 Hz, 1H), 4.53 (d, *J* = 14.3 Hz, 1H), 7.21-7.26 (m, 1H), 7.27-7.39 (m, 10H), 7.41 (d, *J* = 7.8 Hz, 2H), 7.58 (d, *J* = 8.0 Hz, 2H); ^{13}C NMR (125 MHz, $CDCl_3$) δ 16.7, 50.2, 56.7, 76.8, 126.4, 127.3, 127.9, 128.0, 128.2, 128.3, 128.5, 128.8, 129.6, 133.3, 137.0, 140.6, 152.7; IR (neat) *v* 2960, 2926, 1494, 1455, 1349, 1051, 758, 697; Ms (ESI): *m*/*z* = 327.2 [M+H]$^+$. The relative configuration was

assigned by NOESY analysis of this compound. NOEs between the aromatic proton H_c of ***anti*-2a** and H_b, H_a and H_d were observed. Additionally, there was no observable NOE between H_c and H_d. These results indicate that H_a and H_b of ***anti*-2a** have the *anti* relationship.

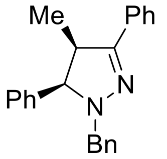

***syn*-2a**

***syn*-2a**: ^{1}H NMR (500 MHz, $CDCl_3$) δ 0.78 (d, J = 7.3 Hz, 3H), 3.36-3.44 (m, 1H), 3.97 (d, J = 14.2 Hz, 1H), 4.31 (d, J = 9.4 Hz, 1H), 4.58 (d, J = 14.2 Hz, 1H), 7.22-7.26 (m, 1H), 7.27-7.48 (m, 12H), 7.69 (d, J = 7.3 Hz, 2H); ^{13}C NMR (125 MHz, $CDCl_3$) δ 13.0, 44.5, 56.3, 72.7, 126.2, 127.3, 127.7, 128.2, 128.5, 128.6, 128.6, 128.6, 129.9, 132.6, 136.6, 136.7, 154.8; IR (neat) ν 2969, 2927, 1495, 1455, 1375, 1169, 1028, 761, 699; Ms (ESI): m/z = 327.2 $[M+H]^+$. The relative configuration was assigned by NOESY analysis of this compound. NOE between the aromatic proton H_c of ***syn*-2a** and H_d was observed. Additionally, there were no observable NOEs between H_a and H_d, or H_b and H_c. Comparing with ***anti*-2a**, these results indicate that H_a and H_b of ***syn*-2a** have the *syn* relationship.

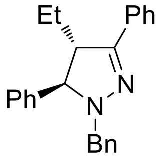

***anti*-2b**

Compound **2b**, yellow oil, yield: 83% (*anti* : *syn* = 7.6 : 1). ***anti*-2b**: ^{1}H NMR (500 MHz, $CDCl_3$) δ 0.67 (t, J = 7.5 Hz, 3H), 1.60-1.69 (m, 2H), 3.44-3.49 (m, 1H), 3.98 (d, J = 14.4 Hz, 1H), 4.09 (d, J = 9.4 Hz, 1H), 4.62 (d, J = 14.4 Hz, 1H), 7.21-7.39 (m, 13H), 7.59 (d, J = 7.7 Hz, 2H); ^{13}C NMR (125 MHz, $CDCl_3$) δ 10.4, 23.5, 56.3, 56.5, 72.0, 126.1, 127.3, 127.8, 127.9, 128.1, 128.2, 128.6, 128.8, 129.5, 133.5, 137.2, 141.9, 150.6; IR (neat) ν 2961, 2927, 1494, 1455, 1357, 1166, 1064, 758, 697; Ms (ESI): m/z = 341.2 $[M+H]^+$. The relative configuration was assigned by NOESY analysis of this compound. NOEs between the aromatic proton H_c of ***anti*-2b** and H_b, H_a and H_d, H_a and H_e were observed. Additionally, there was no observable NOE between H_c and H_d. These results indicate that H_a and H_b of ***anti*-2b** have the *anti* relationship.

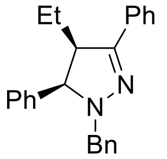

***syn*-2b**

***syn*-2b**: ^{1}H NMR (500 MHz, $CDCl_3$) δ 0.42 (t, J = 7.5 Hz, 3H), 1.25-1.33 (m, 1H), 1.41-1.51 (m, 1H), 3.26-3.31 (m, 1H), 3.96 (d, J = 14.2 Hz, 1H), 4.35 (d, J = 9.6 Hz, 1H), 4.56 (d, J = 14.3 Hz, 1H), 7.22-7.41 (m, 11H), 7.44-7.50 (m, 2H), 7.67 (d, J = 7.7 Hz, 2H); ^{13}C NMR (125 MHz, $CDCl_3$) δ 12.2, 20.3, 51.2, 56.4, 72.7, 125.7, 126.4, 127.2, 127.7, 128.2, 128.5, 128.6, 128.6, 129.8, 131.4, 136.6, 138.0, 155.2; IR (neat) ν 2958, 2927, 1495, 1456, 1361, 1049, 765, 699; Ms (ESI): m/z =

341.2 [M+H]+. The relative configuration was assigned by NOESY analysis of this compound. There were no observable NOEs between H_a and H_d, or H_b and H_c. Comparing with ***anti*-2b**, these results indicate that H_a and H_b of ***syn*-2b** have the *syn* relationship.

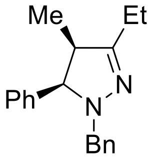

***syn*-2c**

Compound **2c**, yellow oil, yield: 67% (*syn* : *anti* = 3.6 : 1). ***syn*-2c**: ^{1}H NMR (500 MHz, $CDCl_3$) δ 0.66 (d, J = 7.4 Hz, 3H), 1.16 (t, J = 7.5 Hz, 3H), 2.17-2.26 (m, 1H), 2.33-2.42 (m, 1H), 2.84-2.91 (m, 1H), 3.80 (d, J = 14.2 Hz, 1H), 4.09 (d, J = 9.5 Hz, 1H), 4.34 (d, J = 14.2 Hz, 1H), 7.20-7.25 (m, 1H), 7.26-7.31 (m, 5H), 7.32-7.39 (m, 4H); ^{13}C NMR (125 MHz, $CDCl_3$) δ 11.5, 12.4, 22.5, 46.6, 56.9, 72.7, 127.1, 127.4, 128.1, 128.4, 128.5, 129.8, 137.3, 137.4, 160.5; IR (neat) ν 2968, 2928, 1494, 1456, 1374, 1029, 750, 700; Ms (ESI): m/z = 279.2 [M+H]+. The relative configuration was assigned by NOESY analysis of this compound. NOE between the aromatic proton H_c of ***syn*-2c** and H_d was observed. Additionally, there were no observable NOEs between H_a and H_d, or H_b and H_c. These results indicate that H_a and H_b of ***syn*-2c** have the *syn* relationship.

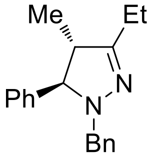

***anti*-2c**

***anti*-2c**: ^{1}H NMR (500 MHz, $CDCl_3$) δ 1.05 (d, J = 6.9 Hz, 3H), 1.13 (t, J = 7.5 Hz, 3H), 2.14-2.23 (m, 1H), 2.29-2.38 (m, 1H), 2.78-2.87 (m, 1H), 3.45 (d, J = 13.5 Hz, 1H), 3.84 (d, J = 14.0 Hz, 1H), 4.20 (d, J = 14.1 Hz, 1H), 7.17-7.23 (m, 1H), 7.24-7.30 (m, 5H), 7.31-7.35 (m, 2H), 7.41 (d, J = 7.7 Hz, 2H); ^{13}C NMR (125 MHz, $CDCl_3$) δ 10.9, 14.3, 21.9, 51.7, 57.8, 77.9, 127.1, 127.8, 128.0, 128.1, 128.6, 129.7, 137.4, 140.6, 158.7; IR (neat) ν 2966, 2932, 1494, 1454, 1375, 1154, 1027, 757, 700; Ms (ESI): m/z = 279.2 [M+H]+. The relative configuration was assigned by NOESY analysis of this compound. NOEs between the aromatic proton H_c of ***anti*-2c** and H_b, H_a and H_d were observed. Additionally, there was no observable NOE between H_c and H_d. Comparing with ***syn*-2c**, these results indicate that H_a and H_b of ***anti*-2c** have the *anti* relationship.

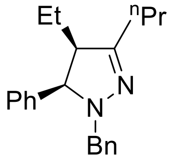

***syn*-2d**

Compound **2d**, yellow oil, yield: 71% (*syn* : *anti* = 2.5 : 1). ***syn*-2d**: ^{1}H NMR (500 MHz, $CDCl_3$) δ 0.53 (t, J = 7.5 Hz, 3H), 0.95 (t, J = 7.4 Hz, 3H), 1.18-1.27 (m, 2H), 1.50-1.60 (m, 1H), 1.60-1.70 (m, 1H), 2.16-2.24 (m, 1H), 2.30-2.38 (m, 1H), 2.69-2.74 (m, 1H), 3.77 (d, J = 14.2 Hz, 1H), 4.11 (d, J = 9.6 Hz, 1H), 4.37 (d, J = 14.2 Hz, 1H), 7.21-7.30 (m, 6H), 7.31-7.44 (m, 4H); ^{13}C NMR (125 MHz, $CDCl_3$) δ 12.2, 14.0, 20.1, 20.4, 32.0, 53.4, 56.8, 72.5, 127.0, 127.5, 128.1, 128.4,

128.6, 129.7, 137.2, 137.3, 158.7; IR (neat) *v* 2958, 2930, 1494, 1454, 1377, 1154, 1028, 742, 700; Ms (ESI): *m*/*z* = 307.2 $[M+H]^+$. The relative configuration was assigned by NOESY analysis of this compound. There were no observable NOEs between H_a and H_d, or H_b and H_c. Comparing with ***anti*-2d**, these results indicate that H_a and H_b of ***syn*-2d** have the *syn* relationship.

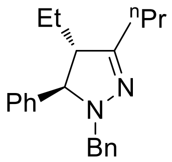

***anti*-2d**

***anti*-2d**: ^{1}H NMR (500 MHz, $CDCl_3$) δ 0.71 (t, *J* = 7.5 Hz, 3H), 0.97 (t, *J* = 7.4 Hz, 3H), 1.41-1.50 (m, 1H), 1.52-1.66 (m, 3H), 2.12-2.20 (m, 1H), 2.23-2.31 (m, 1H), 2.83-2.89 (m, 1H), 33.67 (d, *J* = 12.8 Hz, 1H), 3.84 (d, *J* = 14.2 Hz, 1H), 4.27 (d, *J* = 14.2 Hz, 1H), 7.17-7.28 (m, 6H), 7.29-7.33 (m, 2H), 7.37 (d, *J* = 7.8 Hz, 2H); ^{13}C NMR (125 MHz, $CDCl_3$) δ 11.0, 14.2, 20.1, 22.1, 30.9, 57.4, 58.1, 73.4, 127.1, 127.6, 128.0, 128.3, 128.6, 129.7, 137.2, 141.7, 155.8; IR (neat) *v* 2960, 2929, 1494, 1454, 1382, 1151, 1028, 756, 700; Ms (ESI): *m*/*z* = 307.2 $[M+H]^+$. The relative configuration was assigned by NOESY analysis of this compound. NOEs between the aromatic proton H_c of ***anti*-2d** and H_b, H_a and H_d were observed. Additionally, there was no observable NOE between H_c and H_d. These results indicate that H_a and H_b of ***anti*-2d** have the *anti* relationship.

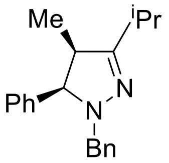

***syn*-2e**

Compound **2e**, yellow oil, yield: 64% (*syn* : *anti* = 1.8 : 1). ***syn*-2e**: ^{1}H NMR (500 MHz, $CDCl_3$) δ 0.66 (d, *J* = 7.3 Hz, 3H), 1.13 (d, *J* = 7.0 Hz, 3H), 1.22 (d, *J* = 6.8 Hz, 3H), 2.49-2.58 (m, 1H), 2.86-2.93 (m, 1H), 3.81 (d, *J* = 14.2 Hz, 1H), 4.02 (d, *J* = 9.4 Hz, 1H), 4.41 (d, *J* = 14.2 Hz, 1H), 7.19-7.30 (m, 7H), 7.31-7.38 (m, 3H); ^{13}C NMR (125 MHz, $CDCl_3$) δ 12.9, 20.3, 21.6, 28.8, 45.7, 56.6, 72.5, 127.1, 127.4, 128.0, 128.4, 128.5, 130.0, 137.0, 137.3, 163.8; IR (neat) *v* 2964, 2927, 1494, 1455, 1373, 1167, 1029, 751, 700; Ms (ESI): *m*/*z* = 293.2 $[M+H]^+$. The relative configuration was assigned by NOESY analysis of this compound. NOE between the aromatic proton H_c of ***syn*-2e** and H_d was observed. Additionally, there were no observable NOEs between H_a and H_d, or H_b and H_c. These results indicate that H_a and H_b of ***syn*-2e** have the *syn* relationship.

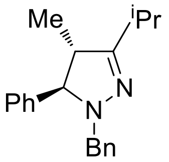

***anti*-2e**

***anti*-2e**: ^{1}H NMR (500 MHz, $CDCl_3$) δ 1.02 (d, *J* = 6.9 Hz, 3H), 1.09 (d, *J* = 7.0 Hz, 3H), 1.22 (d,

J = 6.7 Hz, 3H), 2.49-2.59 (m, 1H), 2.81-2.90 (m, 1H), 3.41 (d, J = 13.5 Hz, 1H), 3.85 (d, J = 14.1 Hz, 1H), 4.31 (d, J = 14.1 Hz, 1H), 7.18-7.29 (m, 6H), 7.30-7.35 (m, 2H), 7.38 (d, J = 7.4 Hz, 2H); ^{13}C NMR (125 MHz, $CDCl_3$) δ 6.72, 14.5, 19.5, 20.4, 28.1, 51.2, 57.5, 127.1, 127.7, 128.0, 128.1, 128.6, 130.0, 136.9, 140.6, 161.2; IR (neat) ν 2963, 2927, 1495, 1456, 1377, 1169, 1020, 757, 700; Ms (ESI): m/z = 293.2 $[M+H]^+$. The relative configuration was assigned by NOESY analysis of this compound. NOEs between the aromatic proton H_c of ***anti*-2e** and H_b, H_a and H_d were observed. Additionally, there was no observable NOE between H_c and H_d. Comparing with ***syn*-2e**, these results indicate that H_a and H_b of ***anti*-2e** have the *anti* relationship.

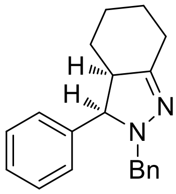

***syn*-2f**

Compound **2f**, yellow oil, yield: 85% (*syn* : *anti* = 5.7 : 1). ***syn*-2f**: ^{1}H NMR (500 MHz, $CDCl_3$) δ 1.06-1.40 (m, 4H), 1.63-1.70 (m, 1H), 1.86-1.93 (m, 1H), 2.12-2.20 (m, 1H), 2.62-2.68 (m, 1H), 2.81-2.88 (m, 1H), 3.89 (d, J = 14.2 Hz, 1H), 4.23 (d, J = 10.9 Hz, 1H), 4.37 (d, J = 14.2 Hz, 1H), 7.20-7.29 (m, 6H), 7.30-7.39 (m, 4H); ^{13}C NMR (125 MHz, $CDCl_3$) δ 25.3, 28.5, 29.1, 29.3, 52.9, 57.3, 70.2, 127.2, 127.3, 128.1, 128.2, 128.3, 129.7, 137.3, 137.9, 157.2; IR (neat) ν 2931, 2854, 1494, 1454, 1177, 1028, 750, 700; Ms (ESI): m/z = 291.2 $[M+H]^+$. The relative configuration was assigned by NOESY analysis of this compound. NOE between the aromatic proton H_c of ***syn*-2f** and H_d was observed. Additionally, there were no observable NOEs between H_a and H_d, or H_b and H_c. These results indicate that H_a and H_b of ***syn*-2f** have the *syn* relationship.

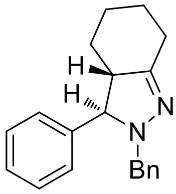

***anti*-2f**

***anti*-2f**: ^{1}H NMR (500 MHz, $CDCl_3$) δ 1.18-1.33 (m, 2H), 1.35-1.45 (m, 1H), 1.76-1.82 (m, 1H), 1.89-1.95 (m, 1H), 1.98-2.12 (m, 2H), 2.61-2.71 (m, 2H), 3.61 (d, J = 12.7 Hz, 1H), 3.84 (d, J = 13.9 Hz, 1H), 4.12 (d, J = 13.9 Hz, 1H), 7.18-7.22 (m, 1H), 7.24-7.35 (m, 7H), 7.42 (d, J = 7.6 Hz, 2H); ^{13}C NMR (125 MHz, $CDCl_3$) δ 24.6, 25.9, 27.8, 31.4, 55.1, 58.3, 77.4, 127.0, 127.6, 127.7, 128.2, 128.7, 129.3, 138.1, 140.1, 156.4; IR (neat) ν 2930, 2856, 1493, 1453, 1373, 1166, 1027, 756, 699; Ms (ESI): m/z = 291.2 $[M+H]^+$. The relative configuration was assigned by NOESY analysis of this compound. NOEs between the aromatic proton H_c of ***anti*-2f** and H_b, H_a and H_d were observed. Additionally, there was no observable NOE between H_c and H_d. Comparing with ***syn*-2f**, these results indicate that H_a and H_b of ***anti*-2f** have the *anti* relationship.

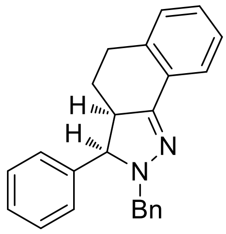

***syn*-2g**

Compound **2g**, yellow oil, yield: 80% (*syn* : *anti* = 3.5 : 1). ***syn*-2g**: ^{1}H NMR (500 MHz, $CDCl_3$) δ 1.22-1.33 (m, 1H), 1.55-1.63 (m, 1H), 2.72-2.78 (m, 1H), 2.81-2.90 (m, 1H), 3.49-3.55 (m, 1H), 4.14 (d, J = 14.1 Hz, 1H), 4.65 (d, J = 10.8 Hz, 1H), 4.77 (d, J = 14.2 Hz, 1H), 7.04-7.31 (m, 13H), 7.97 (d, J = 7.3 Hz, 1H); ^{13}C NMR (125 MHz, $CDCl_3$) δ 23.9, 29.9, 49.8, 55.5, 70.1, 124.3, 126.5, 127.2, 127.8, 128.3, 128.4 (2C), 128.5, 128.9, 129.1, 129.6, 132.7, 136.8, 137.8, 152.4; IR (neat) v 2931, 2849, 1474, 1454, 1359, 1029, 909, 763, 729, 700; Ms (ESI): m/z = 339.2 $[M+H]^+$. The relative configuration was assigned by NOESY analysis of this compound. There were no observable NOEs between H_a and H_d, or H_b and H_c. Comparing with ***anti*-2g**, these results indicate that H_a and H_b of ***syn*-2g** have the *syn* relationship.

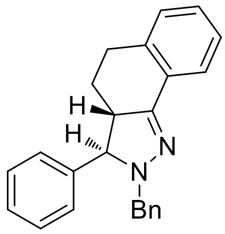

***anti*-2g**

***anti*-2g**: ^{1}H NMR (500 MHz, $CDCl_3$) δ 1.66-1.76 (m, 1H), 2.02-2.09 (m, 1H), 2.79-2.85 (m, 2H), 3.08-3.16 (m, 1H), 3.84 (d, J = 13.7 Hz, 1H), 3.97 (d, J = 14.2 Hz, 1H), 4.41 (d, J = 14.2 Hz, 1H), 7.08-7.13 (m, 1H), 7.18-7.40 (m, 11H), 7.48 (d, J = 7.7 Hz, 2H), 7.93-8.00 (m, 1H); ^{13}C NMR (125 MHz, $CDCl_3$) δ 26.7, 29.4, 54.5, 57.4, 76.7, 124.4, 126.7, 127.2, 127.9, 128.0, 128.2, 128.7, 128.8, 128.9, 129.0, 129.7, 137.3, 138.1, 140.0, 151.7; IR (neat) v 2928, 2836, 1494, 1453, 1371, 1027, 908, 759, 729, 700; Ms (ESI): m/z = 339.2 $[M+H]^+$. The relative configuration was assigned by NOESY analysis of this compound. NOEs between the aromatic proton H_c of ***anti*-2g** and H_b, H_a and H_d were observed. Comparing with ***syn*-2g**, these results indicate that H_a and H_b of ***anti*-2g** have the *anti* relationship.

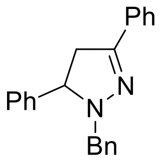

2h

Compound **2h**, yellow oil, yield: 90%. ^{1}H NMR (500 MHz, $CDCl_3$) δ 2.96 (m, 1H), 3.35 (dd, J = 16.0, 10.4 Hz, 1H), 3.96 (d, J = 14.3 Hz, 1H), 4.26 (dd, J = 14.0, 10.5 Hz, 1H), 4.53 (d, J = 14.3 Hz, 1H), 7.20-7.39 (m, 11H), 7.45 (d, J = 7.9 Hz, 2H), 7.62 (d, J = 8.1 Hz, 2H); ^{13}C NMR (125 MHz, $CDCl_3$) δ 43.1, 56.7, 69.1, 125.8, 127.3, 127.8, 127.9, 128.2, 128.5, 128.6, 128.8, 129.7, 133.3, 136.9, 140.8, 149.0; IR (neat) v 2923, 2840, 1494, 1454, 1362, 1059, 910, 758, 699; Ms

(ESI): m/z = 313.2 $[M+H]^+$.

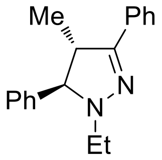

***anti*-2i**

Compound **2i**, yellow oil, yield: 70% (*anti* : *syn* = 8.4 : 1). ***anti*-2i**: ^{1}H NMR (500 MHz, $CDCl_3$) δ 1.20 (t, J = 7.1 Hz, 3H), 1.32 (d, J = 6.8 Hz, 3H), 2.91-2.99 (m, 1H), 3.02-3.11 (m, 1H), 3.35-3.43 (m, 1H), 3.83 (d, J = 12.0 Hz, 1H), 7.27-7.39 (m, 6H), 7.45 (d, J = 7.4 Hz, 2H), 7.59 (d, J = 7.5 Hz, 2H); ^{13}C NMR (125 MHz, $CDCl_3$) δ 12.9, 16.8, 48.1, 50.5, 78.7, 126.4, 127.8, 128.0, 128.2, 128.5, 128.8, 133.4, 141.2, 152.9; IR (neat) v 2966, 2930, 1496, 1455, 1363, 1166, 1031, 873, 754, 696; Ms (ESI): m/z = 265.2 $[M+H]^+$. The relative configuration was assigned by NOESY analysis of this compound. NOEs between the aromatic proton H_c of ***anti*-2i** and H_b, H_a and H_d were observed. Additionally, there was no observable NOE between H_c and H_d. These results indicate that H_a and H_b of ***anti*-2i** have the *anti* relationship.

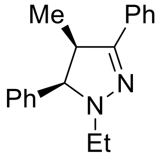

***syn*-2i**

***syn*-2i**: ^{1}H NMR (500 MHz, $CDCl_3$) δ 0.75 (d, J = 7.3 Hz, 3H), 1.29 (t, J = 7.1 Hz, 3H), 2.83-2.91 (m, 1H), 3.11-3.19 (m, 1H), 3.47-3.55 (m, 1H), 4.41 (d, J = 9.4 Hz, 1H), 7.28-7.33 (m, 2H), 7.34-7.40 (m, 4H), 7.43 (d, J = 7.5 Hz, 2H), 7.71 (d, J = 8.0 Hz, 2H); ^{13}C NMR (125 MHz, $CDCl_3$) δ 12.6, 12.8, 44.7, 47.8, 74.4, 126.2, 127.6, 128.3, 128.4, 128.5, 128.7, 132.6, 137.3, 154.9; IR (neat) v 2969, 2929, 1496, 1456, 1364, 1162, 972, 752, 693; Ms (ESI): m/z = 265.2 $[M+H]^+$. The relative configuration was assigned by NOESY analysis of this compound. NOE between the aromatic proton H_c of ***syn*-2i** and H_d was observed. Additionally, there were no observable NOEs between H_a and H_d, or H_b and H_c. Comparing with ***anti*-2i**, these results indicate that H_a and H_b of ***syn*-2i** have the *syn* relationship.

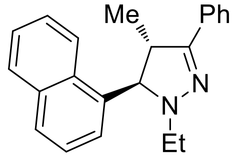

***anti*-2j**

Compound **2j**, yellow oil, yield: 68% (*anti* : *syn* = 9.0 : 1). ***anti*-2j**: ^{1}H NMR (500 MHz, $CDCl_3$) δ 1.17 (t, J = 7.1 Hz, 3H), 1.37 (d, J = 6.8 Hz, 3H), 3.03-3.16 (m, 2H), 3.72 (brs, 1H), 4.53 (brs, 1H), 7.28-7.32 (m, 1H), 7.34-7.39 (m, 2H), 7.44-7.52 (m, 3H), 7.60-7.70 (m, 3H), 7.83 (d, J = 8.2 Hz, 1H), 7.88-7.92 (m, 1H), 8.32-8.43 (m, 1H); ^{13}C NMR (125 MHz, $CDCl_3$) δ 12.9, 18.3, 27.6, 48.6, 72.6, 124.4, 125.6, 125.8, 125.9, 126.4, 128.2, 128.6, 128.7, 129.1, 129.2, 131.8, 133.4, 134.4, 134.5, 152.3; IR (neat) v 2967, 2928, 2853, 1456, 1378, 1175, 1034, 972, 797, 776, 763, 695; Ms (ESI): m/z = 315.2 $[M+H]^+$. The relative configuration was assigned by NOESY analysis of this

compound. NOEs between the aromatic proton H_c of ***anti*-2j** and H_b, H_a and H_d were observed. These results indicate that H_a and H_b of ***anti*-2j** have the *anti* relationship.

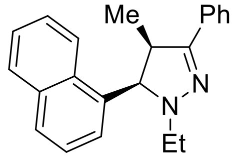

***syn*-2j**

***syn*-2j**: ^{1}H NMR (500 MHz, $CDCl_3$) δ 0.61 (d, J = 7.3 Hz, 3H), 1.38 (t, J = 7.1 Hz, 3H), 2.88-2.96 (m, 1H), 3.21-3.29 (m, 1H), 3.87-3.94 (m, 1H), 5.08 (d, J = 9.5 Hz, 1H), 7.30-7.35 (m, 1H), 7.36-7.41 (m, 2H), 7.51-7.59 (m, 3H), 7.78 (d, J = 7.7 Hz, 2H), 7.83 (d, J = 8.2 Hz, 1H), 7.88 (d, J = 7.1 Hz, 1H), 7.90-7.97 (m, 2H); ^{13}C NMR (125 MHz, $CDCl_3$) δ 12.7, 12.8, 43.3, 48.2, 70.8, 122.6, 125.7, 125.8 (2C), 126.3, 126.4, 127.9, 128.6, 128.7, 129.2, 131.8, 132.6, 132.9, 133.9, 154.5; IR (neat) ν 2969, 2929, 2852, 1448, 1374, 1163, 971, 801, 783, 764, 693; Ms (ESI): m/z = 315.2 $[M+H]^+$. The relative configuration was assigned by NOESY analysis of this compound. NOE between the aromatic proton H_c of ***syn*-2j** and H_d was observed. Additionally, there was no observable NOE between H_a and H_d. Comparing with ***anti*-2j**, these results indicate that H_a and H_b of ***syn*-2j** have the *syn* relationship.

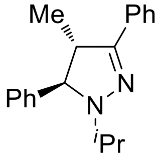

***anti*-2k**

Compound **2k**, yellow oil, yield: 67% (*anti* : *syn* = 3.1 : 1). ***anti*-2k**: ^{1}H NMR (500 MHz, $CDCl_3$) δ 1.09 (d, J = 6.4 Hz, 3H), 1.29 (d, J = 6.6 Hz, 3H), 1.33 (d, J = 6.9 Hz, 3H), 3.21 (m, 1H), 3.35 (m, 1H), 4.00 (d, J = 11.8 Hz, 1H), 7.27-7.39 (m, 6H), 7.45 (d, J = 7.8 Hz, 2H), 7.58 (d, J = 8.5 Hz, 2H); ^{13}C NMR (125 MHz, $CDCl_3$) δ 17.2, 18.0, 21.6, 50.5, 51.9, 75.6, 126.3, 127.6, 127.7, 127.9, 128.5, 128.7, 133.7, 142.5, 151.6; IR (neat) ν 2969, 2928, 1494, 1454, 1361, 1176, 1027, 979, 762, 696; Ms (ESI): m/z = 279.2 $[M+H]^+$. The relative configuration was assigned by NOESY analysis of this compound. NOEs between the aromatic proton H_c of ***anti*-2k** and H_b, H_a and H_d were observed. Additionally, there was no observable NOE between H_c and H_d. These results indicate that H_a and H_b of ***anti*-2k** have the *anti* relationship.

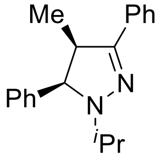

***syn*-2k**

***syn*-2k**: ^{1}H NMR (500 MHz, $CDCl_3$) δ 0.74 (d, J = 7.3 Hz, 3H), 1.02 (d, J = 6.4 Hz, 3H), 1.42 (d, J = 6.7 Hz, 3H), 3.13-3.22 (m, 1H), 3.44-3.52 (m, 1H), 4.62 (d, J = 9.7 Hz, 1H), 7.27-7.32 (m, 2H), 7.33-7.39 (m, 4H), 7.43 (d, J = 7.6 Hz, 2H), 7.70 (d, J = 8.2 Hz, 2H); ^{13}C NMR (125 MHz, $CDCl_3$) δ 13.1, 16.6, 22.2, 44.4, 50.8, 71.5, 126.0, 127.5, 128.1, 128.4, 128.5, 128.6, 133.0, 137.8, 153.6; IR (neat) ν 2971, 2929, 1494, 1455, 1375, 1173, 1121, 763, 693; Ms (ESI): m/z = 279.2 $[M+H]^+$.

The relative configuration was assigned by NOESY analysis of this compound. NOE between the aromatic proton H_c of ***syn*-2k** and H_d was observed. Additionally, there were no observable NOEs between H_a and H_d, or H_b and H_c. Comparing with ***anti*-2k**, these results indicate that H_a and H_b of ***syn*-2k** have the *syn* relationship.

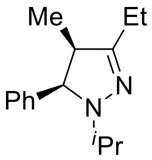

***syn*-2l**

Compound **2l**, yellow oil, yield: 72% (*syn* : *anti* = 2.3 : 1). ***syn*-2l**: ^{1}H NMR (500 MHz, $CDCl_3$) δ 0.62 (d, J = 7.4 Hz, 3H), 0.96 (d, J = 6.4 Hz, 3H), 1.17 (t, J = 7.5 Hz, 3H), 1.28 (d, J = 6.7 Hz, 3H), 2.19-2.28 (m, 1H), 2.35-2.44 (m, 1H), 2.93-3.07 (m, 2H), 4.36 (d, J = 9.9 Hz, 1H), 7.23-7.27 (m, 1H), 7.30-7.34 (m, 2H), 7.36 (d, J = 7.3 Hz, 2H); ^{13}C NMR (125 MHz, $CDCl_3$) δ 11.7, 12.7, 16.2, 22.1, 22.5, 46.5, 50.9, 70.7, 127.2, 128.3, 128.4, 138.7, 159.3; IR (neat) v 2969, 2931, 1494, 1453, 1376, 1170, 751, 702; Ms (ESI): m/z = 231.2 $[M+H]^+$. The relative configuration was assigned by NOESY analysis of this compound. NOE between the aromatic proton H_c of ***syn*-2l** and H_d was observed. Additionally, there were no observable NOEs between H_a and H_d, or H_b and H_c. These results indicate that H_a and H_b of ***syn*-2l** have the *syn* relationship.

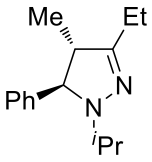

***anti*-2l**

***anti*-2l**: ^{1}H NMR (500 MHz, $CDCl_3$) δ 0.98 (d, J = 6.5 Hz, 3H), 1.11-1.15 (m, 6H), 1.17 (d, J = 6.7 Hz, 3H), 2.16-2.25 (m, 1H), 2.30-2.39 (m, 1H), 2.74-2.83 (m, 1H), 2.98-3.07 (m, 1H), 3.66 (d, J = 13.2 Hz, 1H), 7.25-7.29 (m, 1H), 7.31-7.35 (m, 2H), 7.42 (d, J = 7.5 Hz, 2H); ^{13}C NMR (125 MHz, $CDCl_3$) δ 11.0, 14.7, 17.3, 21.4, 22.0, 52.0, 52.1, 75.6, 127.5, 127.8, 128.6, 142.4, 157.5; IR (neat) v 2967, 2930, 1456, 1376, 1162, 995, 750, 700; Ms (ESI): m/z = 231.2 $[M+H]^+$. The relative configuration was assigned by NOESY analysis of this compound. NOEs between H_a and H_d of ***anti*-2l** was observed. Additionally, there was no observable NOE between H_c and H_d. Comparing with ***syn*-2l**, these results indicate that H_a and H_b of ***anti*-2l** have the *anti* relationship.

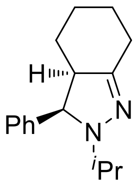

***syn*-2m**

Compound **2m**, yellow oil, yield: 82% (*syn* : *anti* = 3.8 : 1). ***syn*-2m**: ^{1}H NMR (500 MHz, $CDCl_3$) δ 0.95-1.05 (m, 4H), 1.06-1.12 (m, 1H), 1.17-1.27 (m, 4H), 1.29-1.39 (m, 1H), 1.63-1.68 (m, 1H), 1.85-1.91 (m, 1H), 2.14-2.22 (m, 1H), 2.64-2.70 (m, 1H), 2.90-2.97 (m, 1H), 3.09 (hept, J = 6.5 Hz, 1H), 4.46 (d, J = 11.3 Hz, 1H), 7.21-7.25 (m, 1H), 7.28-7.33 (m, 2H), 7.34-7.38 (m, 2H); ^{13}C

NMR (125 MHz, $CDCl_3$) δ 17.7, 21.7, 25.3, 28.1, 29.0, 29.7, 52.2, 52.4, 68.4, 126.9, 128.1 (2C), 139.6, 156.0; IR (neat) ν 2928, 2855, 1489, 1455, 1363, 1173, 992, 747, 698 cm^{-1}; Ms (ESI): *m/z* = 243.2 $[M+H]^+$. The relative configuration was assigned by NOESY analysis of this compound. NOEs between the aromatic proton H_{11} of ***syn*-2m** and H_{4a}, H_{4b} were observed. Additionally, there was no observable NOE between H_{11} and H_{3a}. These results indicate that H_3 and H_{3a} of ***syn*-2m** have the *syn* relationship.

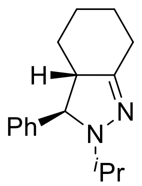

***anti*-2m**

***anti*-2m**: ^1H NMR (500 MHz, $CDCl_3$) δ 1.03 (d, J = 6.4 Hz, 3H), 1.19 (d, J = 6.7 Hz, 3H), 1.25-1.34 (m, 2H), 1.39-1.49 (m, 1H), 1.77-1.85 (m, 1H), 1.91-1.97 (m, 1H), 2.02-2.14 (m, 2H), 2.56-2.67 (m, 2H), 3.02 (hept, J = 6.6 Hz, 1H), 3.79 (d, J = 12.4 Hz, 1H), 7.26-7.29 (m, 1H), 7.30-7.35 (m, 2H), 7.37-7.41 (m, 2H); ^{13}C NMR (125 MHz, $CDCl_3$) δ 17.2, 21.8, 24.7, 26.2, 27.9, 31.7, 52.0, 55.6, 74.2, 127.3, 127.4, 128.6, 142.7, 155.1; IR (neat) ν 2929, 2856, 1494, 1452, 1362, 1166, 990, 748, 700 cm^{-1}; Ms (ESI): *m/z* = 243.2 $[M+H]^+$.

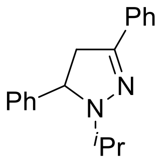

2n

Compound **2n**, yellow oil, yield: 89%.^1H NMR (500 MHz, $CDCl_3$) δ 0.78 (d, J = 7.3 Hz, 3H), 3.36-3.44 (m, 1H), 3.97 (d, J = 14.2 Hz, 1H), 4.31 (d, J = 9.4 Hz, 1H), 4.58 (d, J = 14.2 Hz, 1H), 7.22-7.26 (m, 1H), 7.27-7.48 (m, 12H), 7.69 (d, J = 7.3 Hz, 2H); ^{13}C NMR (125 MHz, $CDCl_3$) δ 13.0, 44.5, 56.3, 72.7, 126.2, 127.3, 127.7, 128.2, 128.5, 128.6, 128.6, 128.6, 129.9, 132.6, 136.6, 136.7, 154.8; IR (neat) ν 2969, 2927, 1495, 1455, 1375, 1169, 1028, 761, 699; Ms (ESI): *m/z* = 265.2 $[M+H]^+$.

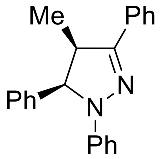

2o

Compound **2o**, yellow oil, yield: 76%. ^1H NMR (500 MHz, $CDCl_3$) δ 1.46 (d, J = 7.1 Hz, 3H), 3.45-3.52 (m, 1H), 4.86 (d, J = 4.5 Hz, 1H), 6.74-6.79 (m, 1H), 7.06 (d, J = 8.5 Hz, 2H), 7.16-7.20 (m, 2H), 7.24-7.27 (m, 3H), 7.28-7.33 (m, 3H), 7.35-7.40 (m, 2H), 7.73 (d, J = 8.1 Hz, 2H); ^{13}C NMR (125 MHz, $CDCl_3$) δ 19.6, 50.9, 72.6, 113.2, 119.0, 125.7, 126.2, 127.8, 128.5, 128.7, 129.1, 129.3, 132.3, 141.7, 144.5, 151.1; IR (neat) ν 2963, 2925, 1597, 1503, 1391, 1135, 1069, 763, 691; Ms (ESI): *m/z* = 313.2 $[M+H]^+$. The relative configuration was assigned by NOESY analysis of this compound. NOE between the aromatic proton H_c of **2o** and H_d was observed. Additionally,

there were no observable NOEs between H_a and H_d, or H_b and H_c. These results indicate that H_a and H_b of **2o** have the *syn* relationship.

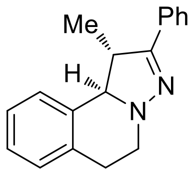

2p

Compound **2p**, yellow oil, yield: 84%. ^{1}H NMR (500 MHz, $CDCl_3$) δ 1.45 (d, J = 7.2 Hz, 3H), 2.51-2.56 (m, 1H), 3.02-3.11 (m, 1H), 3.34-3.42 (m, 1H), 3.55-3.61 (m, 1H), 4.15 (dd, J = 13.7, 4.8 Hz, 1H), 4.63 (brs, 1H), 7.00 (d, J = 7.6 Hz, 1H), 7.10-7.18 (m, 2H), 7.21-7.26 (m, 2H), 7.30-7.34 (m, 2H), 7.63 (d, J = 8.1 Hz, 2H); ^{13}C NMR (125 MHz, $CDCl_3$) δ 17.7, 26.0, 47.8, 49.6, 69.9, 126.2, 126.4, 126.5, 127.1, 128.5, 128.6, 128.9, 132.0, 135.0, 137.6, 156.5; IR (neat) ν 3059, 3020, 2965, 2929, 1494, 1451, 1360, 1159, 1078, 930, 860, 764, 747, 694 cm^{-1}; Ms (ESI): m/z = 263.2 $[M+H]^+$. The relative configuration was assigned by NOESY analysis of this compound. NOE between the aromatic proton H_{10} of **2p** and H_1 was observed. Additionally, there was no observable NOE between H_{10} and H_{11}. These results indicate that H_1 and H_{10b} of **2p** have the *anti* relationship.

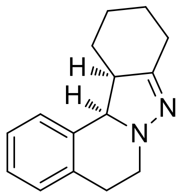

5a

Compound **5a**, yellow oil, yield: 85%. ^{1}H NMR (500 MHz, $CDCl_3$) δ 0.60-0.70 (m, 1H), 1.10-1.21 (m, 1H), 1.31-1.42 (m, 1H), 1.72-1.79 (m, 1H), 1.80-1.89 (m, 2H), 2.05-2.13 (m, 1H), 2.39 (d, J = 15.6 Hz, 1H), 2.64 (dd, J = 14.9, 4.4 Hz, 1H), 2.90-2.98 (m, 1H), 3.12-3.25 (m, 2H), 3.97 (ddd, J = 13.5, 4.2, 1.7 Hz, 1H), 5.03 (d, J = 11.3 Hz, 1H), 6.92 (d, J = 7.3 Hz, 1H), 7.04 (d, J = 7.2 Hz, 1H), 7.10-7.18 (m, 2H); ^{13}C NMR (125 MHz, $CDCl_3$) δ 24.9, 25.4, 25.6, 28.1, 29.8, 48.8, 50.7, 63.6, 125.9, 126.2, 128.5, 128.9, 134.4, 137.0, 159.2; IR (neat) ν 2931, 2854, 1489, 1448, 1358, 1074, 919, 836, 748 cm^{-1}; Ms (ESI): m/z = 227.2 $[M+H]^+$. The relative configuration was assigned by NOESY analysis of this compound. NOEs between the aromatic proton H_1 of **5a** and H_{12a}, H_{12b} were observed. Additionally, there was no observable NOE between H_1 and H_{13}, or H_{14} and H_{12a} or H_{12b}. These results indicate that H_{13} and H_{14} of **5a** have the *syn* relationship.

To further comfirm the relative stereochemistry of the products, the other diastereoisomer ***anti*-5a** was obtained by using $K_2S_2O_8$ as oxidant instead of O_2, and NOESY analysis of ***anti*-5a** was carried out. NOEs between the aromatic proton H_1 of ***anti*-5a** and H_{13}, H_{14} and H_{12a}, H_{12b} were observed. Comparing with **5a**, these results indicate that H_{13} and H_{14} of ***anti*-5a** have the *anti* relationship.

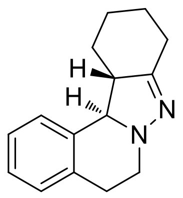

***anti*-5a**

Compound ***anti*-5a**, yellow oil. ^{1}H NMR (500 MHz, $CDCl_3$) δ 1.39-1.50 (m, 2H), 1.64-1.73 (m, 1H), 1.81-1.88 (m, 1H), 1.92-2.02 (m, 1H), 2.07-2.15 (m, 1H), 2.17-2.23 (m, 1H), 2.48-2.53 (m, 1H), 2.57-2.62 (m, 1H), 2.82-2.87 (m, 1H), 2.90-2.98 (m, 1H), 3.22-3.29 (m, 1H), 3.94 (ddd, *J* = 13.5, 4.9, 1.4 Hz, 1H), 4.49 (d, *J* = 3.4 Hz, 1H), 7.02-7.07 (m, 2H), 7.11-7.16 (m, 1H), 7.19-7.24 (m, 1H); ^{13}C NMR (125 MHz, $CDCl_3$) δ 25.6, 25.7, 28.7, 28.8, 33.8, 47.9, 58.4, 66.1, 126.2, 126.3, 127.1, 128.7, 135.3, 138.7, 160.2; IR (neat) ν 2927, 2853, 1489, 1448, 1363, 1163, 1077, 746 cm^{-1}; Ms (ESI): *m*/*z* = 227.2 $[M+H]^+$.

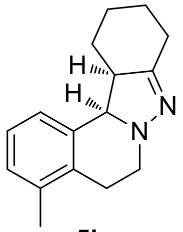

5b

Compound **5b**, yellow oil, yield: 82%. ^{1}H NMR (500 MHz, $CDCl_3$) δ 0.54-0.66 (m, 1H), 1.10-1.20 (m, 1H), 1.30-1.40 (m, 1H), 1.71-1.87 (m, 3H), 2.05-2.13 (m, 1H), 2.24 (s, 3H), 2.45 (d, J = 16.1 Hz, 1H), 2.60-2.75 (m, 2H), 3.12-3.24 (m, 2H), 3.99 (ddd, *J* = 13.7, 4.4, 1.6 Hz, 1H), 5.03 (d, *J* = 11.2 Hz, 1H), 6.77 (d, *J* = 7.5 Hz, 1H), 6.99-7.08 (m, 2H); ^{13}C NMR (125 MHz, $CDCl_3$) δ 19.4, 21.9, 24.9, 25.5, 28.1, 29.6, 48.5, 50.8, 63.8, 125.2, 126.2, 127.6, 134.2, 135.6, 135.8, 159.0; IR (neat) ν 2933, 2857, 1463, 1360, 1171, 927, 835, 757 cm^{-1}; Ms (ESI): *m*/*z* = 241.2 $[M+H]^+$.

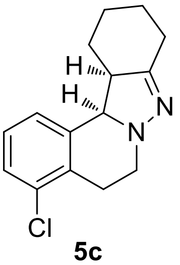

5c

Compound **5c**, yellow oil, yield: 89%. ^{1}H NMR (500 MHz, $CDCl_3$) δ 0.61-0.71 (m, 1H), 1.10-1.21 (m, 1H), 1.31-1.43 (m, 1H), 1.76-1.92 (m, 3H), 2.05-2.13 (m, 1H), 2.37 (d, *J* = 15.7 Hz, 1H), 2.63 (dd, *J* = 15.1, 4.3 Hz, 1H), 2.83-2.91 (m, 1H), 3.07-3.14 (m, 1H), 3.17-3.25 (m, 1H), 3.94-4.00 (m, 1H), 4.96 (d, *J* = 11.3 Hz, 1H), 6.91-6.93 (m, 1H), 6.98 (d, *J* = 8.2 Hz, 1H), 7.10 (dd, *J* = 8.1, 1.9 Hz, 1H); ^{13}C NMR (125 MHz, $CDCl_3$) δ 24.8, 24.9, 25.6, 28.0, 29.8, 48.7, 50.5, 63.4, 126.4, 128.2, 130.2, 131.5, 135.4, 136.3, 159.1; IR (neat) ν 2933, 2856, 1484, 1357, 1177, 1090, 810, 710cm^{-1}; Ms (ESI): *m*/*z* = 261.1 $[M+H]^+$.

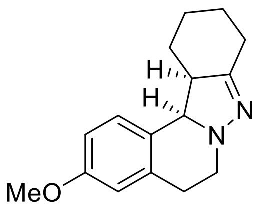

5d

Compound **5d**, yellow oil, yield: 70%. ^{1}H NMR (500 MHz, $CDCl_3$) δ 0.64-0.74 (m, 1H), 1.10-1.21 (m, 1H), 1.32-1.42 (m, 1H), 1.72-1.88 (m, 3H), 2.04-2.12 (m, 1H), 2.32-2.37 (m, 1H), 2.60-2.65 (m, 1H), 2.88-2.97 (m, 1H), 3.11-3.21 (m, 2H), 3.78 (s, 3H), 3.92-3.97 (m, 1H), 4.98 (d, *J* = 11.1 Hz, 1H), 6.58 (d, *J* = 2.4 Hz, 1H), 6.72-6.75 (m, 1H), 6.83 (d, *J* = 8.5 Hz, 1H); ^{13}C NMR (125 MHz, $CDCl_3$) δ 24.9, 25.6, 25.7, 28.1, 29.7, 48.7, 50.8, 55.3, 63.2, 112.5, 113.3, 126.3, 129.6, 138.2, 157.8, 159.1; IR (neat) *ν* 2932, 2855, 1609, 1500, 1457, 1264, 1041, 860, 836 cm^{-1}; Ms (ESI): *m*/*z* = 257.2 [M+H]$^+$.

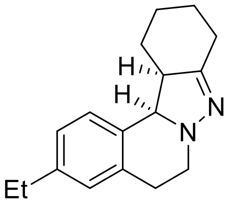

5e

Compound **5e**, yellow oil, yield: 75%. ^{1}H NMR (500 MHz, $CDCl_3$) δ 0.65-0.74 (m, 1H), 1.12-1.24 (m, 4H), 1.32-1.42 (m, 1H), 1.73-1.90 (m, 3H), 2.05-2.13 (m, 1H), 2.33-2.39 (m, 1H), 2.57-2.67 (m, 3H), 2.88-2.97 (m, 1H), 3.12-3.23 (m, 2H), 3.96 (ddd, *J* = 13.5, 4.4, 1.8 Hz, 1H), 5.00 (d, *J* = 11.2 Hz, 1H), 6.84 (d, *J* = 7.9 Hz, 1H), 6.88 (s, 1H), 7.00 (d, *J* = 7.8 Hz, 1H); ^{13}C NMR (125 MHz, $CDCl_3$) δ 15.6, 24.9, 25.5, 25.6, 28.1, 28.5, 29.7, 48.8, 50.7, 63.5, 125.6, 128.2, 128.5, 131.6, 136.8, 142.1, 159.0; IR (neat) *ν* 2931, 2856, 1498, 1446, 1358, 1074, 885, 838 cm^{-1}; Ms (ESI): *m*/*z* = 255.2 [M+H]$^+$.

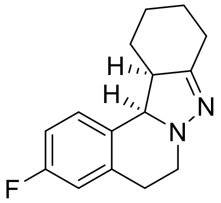

5f

Compound **5f**, yellow oil, yield: 92%. ^{1}H NMR (500 MHz, $CDCl_3$) δ 0.59-0.68 (m, 1H), 1.09-1.20 (m, 1H), 1.31-1.42 (m, 1H), 1.73-1.86 (m, 3H), 2.04-2.12 (m, 1H), 2.33-2.39 (m, 1H), 2.60-2.66 (m, 1H), 2.88-2.97 (m, 1H), 3.08-3.23 (m, 2H), 3.95 (ddd, *J* = 13.5, 4.3, 1.7 Hz, 1H), 4.98 (d, *J* = 11.2 Hz, 1H), 6.75 (d, *J* = 9.1 Hz, 1H), 6.84-6.89 (m, 2H); ^{13}C NMR (125 MHz, $CDCl_3$) δ 24.9, 25.5, 25.6, 28.0, 29.7, 48.4, 50.6, 63.2, 113.2 ($^2J_{CF}$ = 21.4 Hz), 115.2 ($^2J_{CF}$ = 20.4 Hz), 129.9 ($^4J_{CF}$ = 3.1 Hz), 130.0 ($^3J_{CF}$ = 8.1 Hz), 139.1 ($^3J_{CF}$ = 7.4 Hz), 159.0, 161.1 ($^1J_{CF}$ = 244 Hz); IR (neat) *ν* 2933, 2857, 1614, 1495, 1447, 1249, 1142, 865, 837 cm^{-1}; Ms (ESI): *m*/*z* = 245.2 [M+H]$^+$.

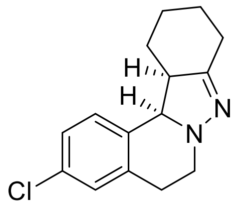

5g

Compound **5g**, yellow oil, yield: 90%. ^{1}H NMR (500 MHz, $CDCl_3$) δ 0.59-0.69 (m, 1H), 1.09-1.20 (m, 1H), 1.32-1.42 (m, 1H), 1.73-1.88 (m, 3H), 2.05-2.13 (m, 1H), 2.33-2.39 (m, 1H), 2.60-2.66 (m, 1H), 2.87-2.95 (m, 1H), 3.08-3.15 (m, 1H), 3.16-3.24 (m, 1H), 3.95 (ddd, *J* = 13.6, 4.3, 1.8 Hz, 1H), 4.98 (d, *J* = 11.2 Hz, 1H), 6.85 (d, *J* = 8.3 Hz, 1H), 7.05 (s, 1H), 7.13 (dd, *J* = 8.5, 1.8 Hz, 1H); ^{13}C NMR (125 MHz, $CDCl_3$) δ 24.9, 25.2, 25.6, 28.0, 29.8, 48.4, 50.6, 63.2, 126.2, 128.7, 129.8, 131.9, 132.9, 138.9, 159.3; IR (neat) ν 2934, 2857, 1481, 1447, 1357, 1264, 1094, 908, 867, 730 cm^{-1}; Ms (ESI): *m*/*z* = 261.1 $[M+H]^+$.

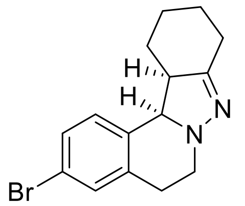

5h

Compound **5h**, yellow oil, yield: 87%. ^{1}H NMR (500 MHz, $CDCl_3$) δ 0.59-0.70 (m, 1H), 1.09-1.20 (m, 1H), 1.31-1.42 (m, 1H), 1.72-1.88 (m, 3H), 2.04-2.13 (m, 1H), 2.32-2.38 (m, 1H), 2.59-2.66 (m, 1H), 2.87-2.95 (m, 1H), 3.07-3.25 (m, 2H), 3.95 (ddd, *J* = 13.6, 4.3, 1.7 Hz, 1H), 4.96 (d, *J* = 11.2 Hz, 1H), 6.79 (d, *J* = 8.2 Hz, 1H), 7.21 (s, 1H), 7.27 (d, *J* = 8.3 Hz, 1H); ^{13}C NMR (125 MHz, $CDCl_3$) δ 24.9, 25.2, 25.5, 28.0, 29.8, 48.4, 50.5, 63.3, 120.0, 129.1, 130.2, 131.6, 133.5, 139.3, 159.1; IR (neat) ν 2932, 2856, 1478, 1446, 1357, 1084, 861, 838 cm^{-1}; Ms (ESI): *m*/*z* = 305.1 $[M+H]^+$.

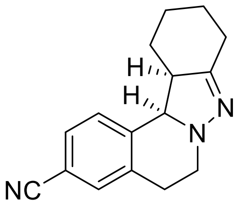

5i

Compound **5i**, yellow oil, yield: 90%. ^{1}H NMR (500 MHz, $CDCl_3$) δ 0.51-0.61 (m, 1H), 1.09-1.20 (m, 1H), 1.33-1.43 (m, 1H), 1.74-1,90 (m, 3H), 2.05-2.14 (m, 1H), 2.40-2.47 (m, 1H), 2.61-2.67 (m, 1H), 2.90-2.99 (m, 1H), 3.08-3.17 (m, 1H), 3.22-3.30 (m, 1H), 4.00 (ddd, *J* = 13.7, 4.3, 1.8 Hz, 1H), 5.02 (d, *J* = 11.4 Hz, 1H), 7.03 (d, *J* = 8.0 Hz, 1H), 7.36 (s, 1H), 7.44 (d, *J* = 8.0 Hz, 1H); ^{13}C NMR (125 MHz, $CDCl_3$) δ 24.8, 25.1, 25.5, 28.0, 30.0, 48.3, 50.5, 63.6, 110.2, 119.0, 129.3, 129.4, 132.6, 138.5, 140.5, 159.3; IR (neat) ν 2934, 2857, 2227, 1491, 1446, 1358, 1074, 895, 839, 729 cm^{-1}; Ms (ESI): *m*/*z* = 252.1 $[M+H]^+$.

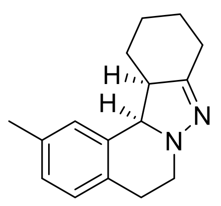

5j

Compound **5j**, yellow oil, yield: 85%. ^{1}H NMR (500 MHz, $CDCl_3$) δ 0.61-0.71 (m, 1H), 1.11-1.21 (m, 1H), 1.32-1.43 (m, 1H), 1.73-1.89 (m, 3H), 2.05-2.13 (m, 1H), 2.30 (s, 3H), 2.33-2.38 (m, 1H), 2.60-2.66 (m, 1H), 2.84-2.93 (m, 1H), 3.10-3.24 (m, 2H), 3.96 (ddd, *J* = 13.5, 4.1, 1.7 Hz, 1H), 4.98 (d, *J* = 11.3 Hz, 1H), 6.74 (s, 1H), 6.94 (brs, 2H); ^{13}C NMR (125 MHz, $CDCl_3$) δ 21.3, 25.0, 25.1, 25.7, 28.1, 29.9, 49.0, 50.7, 63.6, 127.0, 128.7, 129.0, 133.9, 134.3, 135.3, 159.0; IR (neat) ν 2930, 2855, 1502, 1446, 1357, 1073, 836, 809 cm^{-1}; Ms (ESI): *m*/*z* = 241.2 $[M+H]^{+}$.

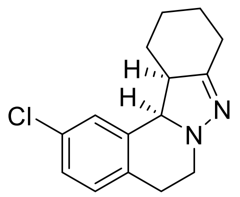

5k

Compound **5k**, yellow oil, yield: 91%. ^{1}H NMR (500 MHz, $CDCl_3$) δ 0.61-0.71 (m, 1H), 1.10-1.21 (m, 1H), 1.33-1.43 (m, 1H), 1.76-1.92 (m, 3H), 2.05-2.13 (m, 1H), 2.34-2.41 (m, 1H), 2.60-2.67 (m, 1H), 2.81-2.91 (m, 1H), 3.06-3.14 (m, 1H), 3.17-3.25 (m, 1H), 3.97 (ddd, *J* = 13.6, 4.3, 1.7 Hz, 1H), 4.96 (d, *J* = 11.3 Hz, 1H), 6.92 (d, *J* = 1.8 Hz, 1H), 6.98 (d, *J* = 8.2 Hz, 1H), 7.10 (dd, *J* = 8.2, 2.1 Hz, 1H); ^{13}C NMR (125 MHz, $CDCl_3$) δ 24.8, 24.9, 25.6, 28.0, 29.8, 48.6, 50.5, 63.4, 126.4, 128.2, 130.2, 131.5, 135.4, 136.3, 159.1; IR (neat) ν 2965, 2929, 1494, 1451, 1360, 1159, 1078, 930, 860 cm^{-1}; Ms (ESI): *m*/*z* = 261.1 $[M+H]^{+}$.

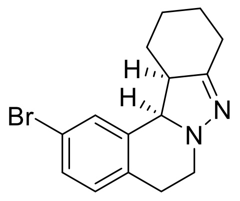

5l

Compound **5l**, yellow oil, yield: 86%. ^{1}H NMR (500 MHz, $CDCl_3$) δ 0.60-0.70 (m, 1H), 1.10-1.21 (m, 1H), 1.33-1.43 (m, 1H), 1.73-1.92 (m, 3H), 2.05-2.14 (m, 1H), 2.33-2.39 (m, 1H), 2.60-2.67 (m, 1H), 2.80-2.89 (m, 1H), 3.06-3.25 (m, 2H), 3.97 (ddd, *J* = 13.6, 4.2, 1.5 Hz, 1H), 4.96 (d, *J* = 11.3 Hz, 1H), 6.93 (d, *J* = 8.1 Hz, 1H), 7.07 (s, 1H), 7.23-7.26 (m, 1H); ^{13}C NMR (125 MHz, $CDCl_3$) δ 24.9 (2C), 25.6, 28.0, 29.8, 48.6, 50.6, 63.3, 119.4, 129.3, 130.5, 131.2, 136.0, 136.7, 159.2; IR (neat) ν 2932, 2855, 1481, 1446, 1357, 1176, 1075, 922, 836, 807cm^{-1}; Ms (ESI): *m*/*z* = 305.1 $[M+H]^{+}$.

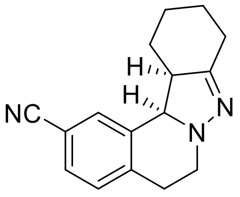

5m

Compound **5m**, yellow oil, yield: 89%. ^{1}H NMR (500 MHz, $CDCl_3$) δ 0.51-0.61 (m, 1H), 1.06-1.18 (m, 1H), 1.33-1.44 (m, 1H), 1.76-1.91 (m, 3H), 2.05-2.14 (m, 1H), 2.43-2.48 (m, 1H), 2.60-2.66 (m, 1H), 2.93-3.01 (m, 1H), 3.09-3.16 (m, 1H), 3.21-3.28 (m, 1H), 4.00 (ddd, *J* = 13.6, 4.3, 1.4 Hz, 1H), 4.99 (d, *J* = 11.2 Hz, 1H), 7.15 (d, *J* = 7.9 Hz, 1H), 7.23 (s, 1H), 7.40 (d, *J* = 7.9 Hz, 1H); ^{13}C NMR (125 MHz, $CDCl_3$) δ 24.8, 25.5, 25.6, 27.9, 29.9, 48.2, 50.4, 63.2, 110.0, 119.0, 129.6, 129.9, 132.2, 136.1, 142.8, 159.2; IR (neat) ν 2934, 2857, 2227, 1494, 1447, 1358, 1169, 1074, 913, 837, 729 cm^{-1}; Ms (ESI): *m*/*z* = 252.1 $[M+H]^+$.

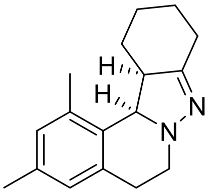

5n

Compound **5n**, yellow oil, yield: 71%. ^{1}H NMR (500 MHz, $CDCl_3$) δ 0.44-0.53 (m, 1H), 1.19-1.42 (m, 3H), 1.62-1.69 (m, 1H), 1.84-1.90 (m, 1H), 2.09-2.21 (m, 4H), 2.26 (s, 3H), 2.33-2.38 (m, 1H), 2.63-2.69 (m, 1H), 2.71-2.79 (m, 1H), 2.99-3.06 (m, 1H), 3.21-3.29 (m, 1H), 3.90 (ddd, *J* = 13.4, 3.7, 2.2 Hz, 1H), 5.16 (d, *J* = 12.0 Hz, 1H), 6.74 (s, 1H), 6.84 (s, 1H); ^{13}C NMR (125 MHz, $CDCl_3$) δ 19.5, 21.0, 25.1, 26.2, 27.0, 28.6, 30.2, 49.4, 51.3, 60.6, 127.0, 129.3, 130.6, 134.9, 135.6, 137.6, 160.2; IR (neat) ν 2929, 2856, 1445, 1358, 1182, 1070, 849, 735 cm^{-1}; Ms (ESI): *m*/*z* = 255.2 $[M+H]^+$.

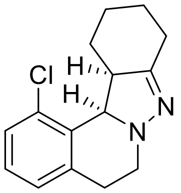

5o

Compound **5o**, yellow oil, yield: 79%. ^{1}H NMR (500 MHz, $CDCl_3$) δ 0.42-0.52 (m, 1H), 1.20-1.28 (m, 1H), 1.34-1.45 (m, 1H), 1.64-1.92 (m, 3H), 2.09-2.17 (m, 1H), 2.39-2.45 (m, 1H), 2.62-2.68 (m, 1H), 2.76-2.84 (m, 1H), 3.00-3.07 (m, 1H), 3.33-3.41 (m, 1H), 3.95 (ddd, *J* = 13.6, 3.7, 2.0 Hz, 1H), 5.33 (d, *J* = 11.9 Hz, 1H), 6.99 (d, *J* = 7.5 Hz, 1H), 7.07-7.11 (m, 1H), 7.22 (d, *J* = 7.9 Hz, 1H); ^{13}C NMR (125 MHz, $CDCl_3$) δ 25.0, 25.8, 26.9, 28.5, 29.8, 49.1, 51.5, 61.3, 127.2, 127.3, 127.7, 133.0, 133.3, 140.0, 160.5; IR (neat) ν 2931, 2855, 1457, 1443, 1358, 1187, 1071, 955, 825, 781 cm^{-1}; Ms (ESI): *m*/*z* = 261.1 $[M+H]^+$.

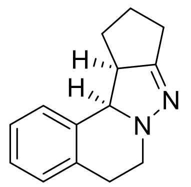

5p

Compound **5p**, yellow oil, yield: 62%. ^{1}H NMR (500 MHz, $CDCl_3$) δ 1.02-1.13 (m, 1H), 1.86-1.93 (m, 1H), 1.96-2.08 (m, 2H), 2.12-2.21 (m, 1H), 2.28-2.36 (m, 1H), 2.37-2.43 (m, 1H), 3.06-3.15 (m, 1H), 3.16-3.23 (m, 1H), 3.58-3.66 (m, 1H), 3.97-4.03 (m, 1H), 5.01 (d, *J* = 10.3 Hz, 1H), 6.84 (d, *J* = 7.5 Hz, 1H), 7.04 (d, *J* = 7.5 Hz, 1H), 7.10-7.19 (m, 2H); ^{13}C NMR (125 MHz, $CDCl_3$) δ 22.6, 25.1, 26.8, 27.6, 48.9, 56.7, 66.2, 126.1, 126.2, 128.3, 129.1, 134.1, 136.6, 169.5; IR (neat) ν 2956, 2869, 1490, 1449, 1133, 1066, 916, 748 cm^{-1}; Ms (ESI): *m*/*z* = 213.1 $[M+H]^+$.

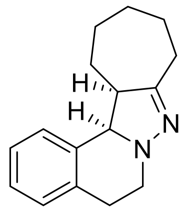

5q

Compound **5q**, yellow oil, yield: 82%. ^{1}H NMR (500 MHz, $CDCl_3$) δ 1.27-1.37 (m, 3H), 1.42-1.75 (m, 4H), 1.86-1.96 (m, 1H), 2.40-2.56 (m, 3H), 3.03-3.12 (m, 2H), 3.35-3.42 (m, 1H), 3.89-3.96 (m, 1H), 4.79 (d, *J* = 10.0 Hz, 1H), 7.03-7.08 (m, 2H), 7.12-7.18 (m, 2H); ^{13}C NMR (125 MHz, $CDCl_3$) δ 26.4, 26.7, 27.2, 28.8, 30.5, 30.9, 48.9, 53.4, 66.8, 125.8, 126.3, 128.3, 129.1, 134.0, 137.1, 163.0; IR (neat) ν 2924, 2851, 1489, 1449, 1363, 1142, 1071, 926, 747 cm^{-1}; Ms (ESI): *m*/*z* = 241.2 $[M+H]^+$.

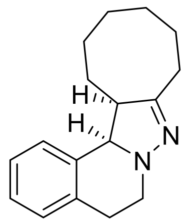

5r

Compound **5r**, yellow oil, yield: 71%. ^{1}H NMR (500 MHz, $CDCl_3$) δ 1.20-1.35 (m, 3H), 1.40-1.59 (m, 4H), 1.65-1.88 (m, 3H), 2.38-2.45 (m, 1H), 2.58-2.65 (m, 2H), 3.00-3.12 (m, 2H), 3.26-3.32 (m, 1H), 3.86-3.94 (m, 1H), 4.60 (d, *J* = 9.8 Hz, 1H), 7.04-7.10 (m, 2H), 7.12-7.18 (m, 2H); ^{13}C NMR (125 MHz, $CDCl_3$) δ 25.2, 25.4, 25.8, 26.1, 26.9, 28.0, 29.2, 49.4, 50.9, 67.8, 125.8, 126.4, 127.8, 129.0, 134.3, 137.0, 163.0; IR (neat) ν 2925, 2853, 1490, 1457, 1358, 1073, 1017, 926, 746 cm^{-1}; Ms (ESI): *m*/*z* = 255.2 $[M+H]^+$.

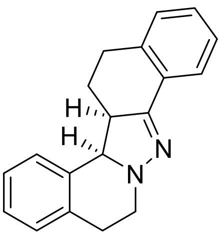

5s

Compound **5s**, yellow oil, yield: 78%. ^{1}H NMR (500 MHz, $CDCl_3$) δ 1.28-1.38 (m, 1H), 2.22-2.28 (m, 1H), 2.42-2.48 (m, 1H), 2.83-2.90 (m, 1H), 2.93-3.02 (m, 1H), 3.09-3.17 (m, 1H), 3.29-3.36 (m, 1H), 3.57-3.64 (m, 1H), 4.17 (dd, *J* = 13.6, 4.6 Hz, 1H), 5.13 (d, *J* = 10.4 Hz, 1H), 7.02 (d, *J* = 7.6 Hz, 2H), 7.08-7.14 (m, 2H), 7.15-7.22 (m, 3H), 7.92 (d, *J* = 7.8 Hz, 1H); ^{13}C NMR (125 MHz, $CDCl_3$) δ 25.3, 26.1, 30.0, 48.8, 49.2, 65.5, 124.3, 126.0, 126.4, 126.6, 128.0, 128.7, 128.8, 129.0, 129.2, 133.7, 136.7, 137.7, 154.6; IR (neat) *v* 2932, 2867, 1491, 1461, 1365, 1073, 919, 751, 729 cm^{-1}; Ms (ESI): *m*/*z* = 275.2 $[M+H]^+$.

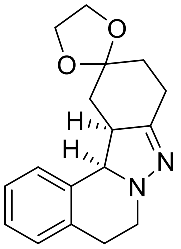

5t

Compound **5t**, yellow oil, yield: 75%. ^{1}H NMR (500 MHz, $CDCl_3$) δ 1.04 (t, *J* = 12.9 Hz, 1H), 1.45-1.53 (m, 1H), 1.79-1.89 (m, 2H), 2.37-2.45 (m, 2H), 2.63 (dd, *J* = 15.1, 5.0 Hz, 1H), 2.91-2.99 (m, 1H), 3.13-3.20 (m, 1H), 3.53-3.61 (m, 1H), 3.91-4.02 (m, 5H), 5.06 (d, *J* = 11.4 Hz, 1H), 6.87 (d, *J* = 7.4 Hz, 1H), 7.04 (d, *J* = 7.3 Hz, 1H), 7.10-7.18 (m, 2H); ^{13}C NMR (125 MHz, $CDCl_3$) δ 24.5, 25.5, 33.3, 37.7, 48.5, 48.9, 64.3, 64.6, 64.8, 109.0, 126.3, 126.4, 128.4, 129.1, 133.9, 137.0, 156.6; IR (neat) *v* 2954, 2930, 2888, 1490, 1455, 1351, 1264, 1116, 1056, 1029, 927, 749 cm^{-1}; Ms (ESI): *m*/*z* = 285.2 $[M+H]^+$.

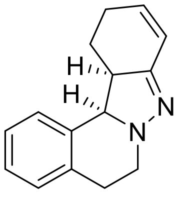

5u

Compound **5u**, yellow oil, yield: 78%. ^{1}H NMR (500 MHz, $CDCl_3$) δ 1.14-1.24 (m, 1H), 2.10-2.16 (m, 1H), 2.26-2.32 (m, 2H), 2.39-2.45 (m, 1H), 3.03-3.12 (m, 1H), 3.23-3.31 (m, 1H), 3.37-3.44 (m, 1H), 4.05 (dd, *J* = 13.6, 4.7 Hz, 1H), 4.99 (d, *J* = 10.3 Hz, 1H), 6.02-6.07 (m, 1H), 6.31 (d, *J* = 10.2 Hz, 1H), 6.97 (d, *J* = 7.4 Hz, 1H), 7.04 (d, *J* = 7.4 Hz, 1H), 7.11-7.19 (m, 2H); ^{13}C NMR (125 MHz, $CDCl_3$) δ 24.8, 25.8, 25.9, 47.9, 48.4, 64.9, 121.2, 125.9, 126.4, 128.0, 129.2, 133.7, 135.2, 136.7, 155.5; IR (neat) *v* 3034, 2930, 2870, 1490, 1449, 1351, 1201, 1132, 1073, 920, 852, 750 cm^{-1}; Ms (ESI): *m*/*z* = 225.1 $[M+H]^+$.

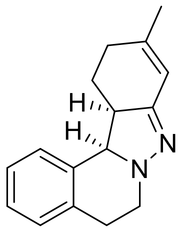

5v

Compound **5v**, yellow oil, yield: 71%. ^{1}H NMR (500 MHz, $CDCl_3$) δ 1.15-1.25 (m, 1H), 1.79 (s, 3H), 2.07-2.17 (m, 2H), 2.26-2.42 (m, 2H), 3.04-3.12 (m, 1H), 3.21-3.37 (m, 2H), 4.03 (ddd, *J* = 13.5, 4.6, 1.2 Hz, 1H), 4.96 (d, *J* = 10.2 Hz, 1H), 6.08 (s, 1H), 6.96 (d, *J* = 7.4 Hz, 1H), 7.03 (d, *J* = 7.5 Hz, 1H), 7.10-7.18 (m, 2H); ^{13}C NMR (125 MHz, $CDCl_3$) δ 24.0, 24.9, 25.6, 31.2, 47.6, 48.5, 64.6, 116.9, 125.9, 126.3, 128.1, 129.2, 133.8, 136.7, 145.4, 156.6; IR (neat) *v* 3018, 2929, 2868, 1489, 1449, 1385, 1073, 921, 851, 749 cm^{-1}; Ms (ESI): *m*/*z* = 239.2 [M+H]$^{+}$.

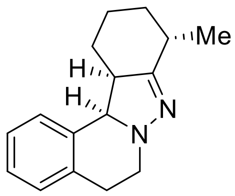

5w1

Compound **5w1**, yellow oil, yield: 61%. ^{1}H NMR (500 MHz, $CDCl_3$) δ 0.59-0.69 (m, 1H), 1.15 (d, *J* = 7.3 Hz, 3H), 1.39-1.47 (m, 1H), 1.51-1.66 (m, 3H), 1.80-1.87 (m, 1H), 2.37-2.42 (m, 1H), 2.84-2.98 (m, 2H), 3.12-3.19 (m, 1H), 3.36-3.43 (m, 1H), 3.97 (ddd, *J* = 13.5, 4.3, 1.8 Hz, 1H), 5.01 (d, *J* = 11.3 Hz, 1H), 6.93 (d, *J* = 7.2 Hz, 1H), 7.04 (d, *J* = 7.2 Hz, 1H), 7.11-7.19 (m, 2H); ^{13}C NMR (125 MHz, $CDCl_3$) δ 18.7, 19.8, 25.5, 30.2, 31.2, 32.0, 47.4, 48.8, 63.6, 125.9, 126.2, 128.6, 128.9, 134.4, 137.0, 162.8; IR (neat) *v* 2926, 2854, 1489, 1456, 1374, 1261, 1074, 1024, 804, 746 cm^{-1}; Ms (ESI): *m*/*z* = 241.2 [M+H]$^{+}$. The relative configuration was determined by NOESY analysis of this compound. NOEs between proton H_{13} of **5w1** and H_{15} was observed.

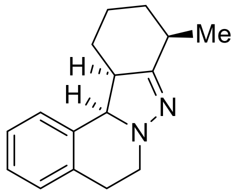

5w2

Compound **5w2**, yellow oil, yield: 17%. ^{1}H NMR (500 MHz, $CDCl_3$) δ 0.58-0.68 (m, 1H), 0.87-0.95 (m, 1H), 1.19 (d, *J* = 6.5 Hz, 3H), 1.38-1.48 (m, 1H), 1.71-1.87 (m, 3H), 2.21-2.28 (m, 1H), 2.35-2.41 (m, 1H), 2.89-2.97 (m, 1H), 3.12-3.26 (m, 2H), 4.02 (ddd, *J* = 13.5, 4.2, 1.9 Hz, 1H), 5.03 (d, *J* = 11.3 Hz, 1H), 6.93 (d, *J* = 7.2 Hz, 1H), 7.04 (d, *J* = 6.5 Hz, 1H), 7.10-7.18 (m, 2H); ^{13}C NMR (125 MHz, $CDCl_3$) δ 17.4, 25.1, 25.3, 30.1, 34.6, 34.9, 48.9, 50.9, 63.8, 125.9, 126.1, 128.6, 128.8, 134.7, 137.2, 162.9; IR (neat) *v* 2927, 2853, 1489, 1448, 1374, 1184, 1063, 861, 747 cm^{-1}; Ms (ESI): *m*/*z* = 241.2 [M+H]$^{+}$.

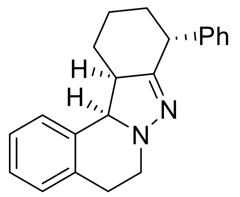

5x1

Compound **5x1**, yellow oil, yield: 71%. ^{1}H NMR (500 MHz, $CDCl_3$) δ 0.70-0.81 (m, 1H), 1.46-1.71 (m, 3H), 1.73-1.85 (m, 1H), 2.34-2.40 (m, 1H), 2.44-2.50 (m, 1H), 2.97-3.06 (m, 1H), 3.20-3.27 (m, 1H), 3.33-3.40 (m, 1H), 4.03-4.09 (m, 2H), 5.09 (d, J = 11.4 Hz, 1H), 6.92 (d, J = 6.6 Hz, 1H), 7.08 (d, J = 6.5 Hz, 1H), 7.14-7.24 (m, 3H), 7.28-7.35 (m, 4H); ^{13}C NMR (125 MHz, $CDCl_3$) δ 20.5, 25.7, 30.0, 30.2, 40.6, 48.7, 48.9, 63.8, 126.0, 126.3 (2C), 127.4, 128.5, 128.6, 128.9, 134.3, 137.0, 140.9, 159.9; IR (neat) ν 3058, 3023, 2933, 2860, 1494, 1447, 1355, 1183, 1075, 919, 748, 732, 698 cm^{-1}; Ms (ESI): m/z = 303.2 [M+H]$^+$.

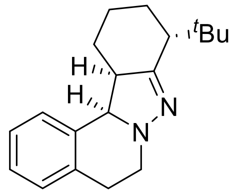

5y1

Compound **5y1**, yellow oil, yield: 55%. ^{1}H NMR (500 MHz, $CDCl_3$) δ 0.56-0.66 (m, 1H), 1.01 (s, 9H), 1.31-1.40 (m, 1H), 1.46-1.53 (m, 1H), 1.54-1.64 (m, 1H), 1.72-1.78 (m, 1H), 1.96-2.02 (m, 1H), 2.40-2.46 (m, 2H), 2.87-2.95 (m, 1H), 3.18 (ddd, J = 13.5, 12.2, 2.7 Hz, 1H), 3.32-3.40 (m, 1H), 3.99 (ddd, J = 13.4, 4.2, 2.2 Hz, 1H), 5.08 (d, J = 11.5 Hz, 1H), 6.95 (d, J = 7.2 Hz, 1H), 7.05 (d, J = 7.3 Hz, 1H), 7.10-7.18 (m, 2H); ^{13}C NMR (125 MHz, $CDCl_3$) δ 22.5, 25.9, 26.4, 29.9, 30.3, 34.8, 45.9, 49.0, 50.3, 64.0, 126.0, 126.2, 128.5, 128.8, 134.7, 137.2, 160.7; IR (neat) ν 2942, 2865, 1491, 1451, 1364, 1183, 1076, 923, 747 cm^{-1}; Ms (ESI): m/z = 283.2 [M+H]$^+$. The relative configuration was determined by NOESY analysis of this compound. NOEs between the proton H_{13} of **5y1** and H_{15} was observed.

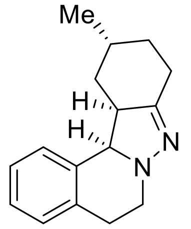

5z1

Compound **5z1**, yellow oil, yield: 43%. ^{1}H NMR (500 MHz, $CDCl_3$) δ 0.87-0.95 (m, 1H), 1.07 (d, J = 7.3 Hz, 3H), 1.39-1.48 (m, 1H), 1.60-1.71 (m, 2H), 2.00-2.10 (m, 1H), 2.28-2.43 (m, 2H), 2.47 (dd, J = 15.4, 4.3 Hz, 1H), 2.91-2.99 (m, 1H), 3.13-3.20 (m, 1H), 3.40-3.48 (m, 1H), 3.98 (dd, J = 13.6, 2.7 Hz, 1H), 5.03 (d, J = 11.2 Hz, 1H), 6.91 (d, J = 7.2 Hz, 1H), 7.04 (d, J = 6.2 Hz, 1H), 7.10-7.18 (m, 2H); ^{13}C NMR (125 MHz, $CDCl_3$) δ 16.7, 23.0, 25.4, 27.4, 30.6, 34.9, 45.5, 48.9, 63.8, 125.9, 126.2, 128.6, 128.9, 134.3, 137.1, 159.6; IR (neat) ν 2925, 2854, 1490, 1455, 1348, 1154, 1074, 854, 748 cm^{-1}; Ms (ESI): m/z = 241.2 [M+H]$^+$.

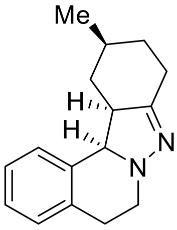

5z2

Compound **5z2**, yellow oil, yield: 38%. ^{1}H NMR (500 MHz, $CDCl_3$) δ 0.37-0.46 (m, 1H), 0.80-0.95 (m, 4H), 1.55-1.82 (m, 3H), 2.10-2.19 (m, 1H), 2.37-2.43 (m, 1H), 2.62 (dd, *J* = 15.1, 4.4 Hz, 1H), 2.91-2.99 (m, 1H), 3.12-3.20 (m, 1H), 3.25-3.32 (m, 1H), 3.97 (ddd, *J* = 13.5, 4.2, 1.6 Hz, 1H), 5.02 (d, *J* = 11.3 Hz, 1H), 6.93 (d, *J* = 7.4 Hz, 1H), 7.05 (d, *J* = 7.3 Hz, 1H), 7.11-7.19 (m, 2H); ^{13}C NMR (125 MHz, $CDCl_3$) δ 22.1, 25.4, 27.5, 31.7, 33.9, 37.9, 48.9, 50.6, 63.9, 126.0, 126.2, 128.6, 128.9, 134.4, 136.9, 159.1; IR (neat) ν 2926, 2855, 1490, 1456, 1357, 1181, 1072, 854, 746 cm^{-1}; Ms (ESI): *m*/*z* = 241.2 [M+H]$^+$.

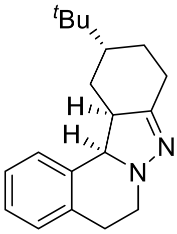

5aa1

Compound **5aa1**, yellow oil, yield: 42%. ^{1}H NMR (500 MHz, $CDCl_3$) δ 0.73 (s, 9H), 0.95-1.02 (m, 1H), 1.31-1.38 (m, 1H), 1.42-1.50 (m, 1H), 1.61-1.71 (m, 2H), 2.30-2.42 (m, 2H), 2.46-2.53 (m, 1H), 2.93-3.01 (m, 1H), 3.10-3.18 (m, 1H), 3.44-3.51 (m, 1H), 3.96 (ddd, *J* = 13.4, 4.2, 1.9 Hz, 1H), 4.99 (d, *J* = 11.2 Hz, 1H), 6.96 (d, *J* = 7.5 Hz, 1H), 7.04 (d, *J* = 7.8 Hz, 1H), 7.10-7.19 (m, 2H); ^{13}C NMR (125 MHz, $CDCl_3$) δ 23.1, 24.4, 25.6, 25.8, 27.6, 33.1, 42.5, 47.5, 49.2, 65.6, 126.0, 126.2, 128.8, 129.0, 133.7, 137.3, 160.9; IR (neat) ν 2956, 2867, 1489, 1456, 1364, 1187, 1071, 922, 747 cm^{-1}; Ms (ESI): *m*/*z* = 283.2 [M+H]$^+$. The relative configuration was determined by NOESY analysis of this compound. NOEs between proton H_{13} of **5aa1** and H_{15} was observed.

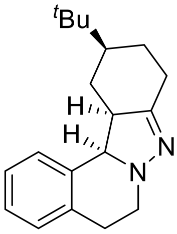

5aa2

Compound **5aa2**, yellow oil, yield: 30%. ^{1}H NMR (500 MHz, $CDCl_3$) δ 0.38-0.47 (m, 1H), 0.78 (s, 9H), 0.88-0.97 (m, 1H), 1.87-1.95 (m, 2H), 1.96-2.15 (m, 2H), 2.37-2.43 (m, 1H), 2.66-2.70 (m, 1H), 2.92-3.01 (m, 1H), 3.13-3.28 (m, 2H), 3.97 (ddd, *J* = 13.5, 4.3, 1.8 Hz, 1H), 5.03 (d, *J* = 11.1 Hz, 1H), 6.94 (d, *J* = 7.4 Hz, 1H), 7.04 (d, *J* = 6.1 Hz, 1H), 7.10-7.19 (m, 2H); ^{13}C NMR (125 MHz, $CDCl_3$) δ 25.4, 26.7, 27.8 (2C), 30.8, 32.7, 47.0, 48.8, 51.0, 64.2, 125.9, 126.2, 128.5, 128.9,

134.3, 136.9, 159.5; IR (neat) *ν* 2953, 2867, 1506, 1456, 1365, 1148, 1074, 925, 747 cm^{-1}; Ms (ESI): *m*/*z* = 283.2 $[M+H]^+$.

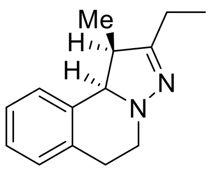

***syn*-5ab**

Compound **5ab**, yellow oil, yield: 64% (*syn* : *anti* = 5.6 : 1). ***syn*-5ab**: ^{1}H NMR (500 MHz, $CDCl_3$) δ 0.98 (d, *J* = 7.5 Hz, 3H), 1.13 (t, *J* = 7.5 Hz, 3H), 2.13-2.22 (m, 1H), 2.26-2.36 (m, 1H), 2.45-2.51 (m, 1H), 2.97-3.05 (m, 1H), 3.08-3.15 (m, 1H), 3.33-3.41 (m, 1H), 3.94 (ddd, *J* = 12.6, 4.6, 2.0 Hz, 1H), 4.79 (d, *J* = 10.2 Hz, 1H), 7.01 (d, *J* = 6.9 Hz, 1H), 7.06 (d, *J* = 7.1 Hz, 1H), 7.12-7.19 (m, 2H); ^{13}C NMR (125 MHz, $CDCl_3$) δ 11.2, 13.0, 22.0, 26.5, 45.9, 49.0, 65.8, 125.7, 126.3, 128.5, 129.0, 133.7, 137.1, 162.6; IR (neat) *ν* 2968, 2932, 1490, 1455, 1375, 1067, 929, 851, 748 cm^{-1}; Ms (ESI): *m*/*z* = 215.2 $[M+H]^+$. The relative configuration was assigned by NOESY analysis of this compound. NOE between the aromatic proton H_{10} of ***syn*-5ab** and H_{11} was observed. Additionally, there was no observable NOE between H_{10} and H_1. These results indicate that H_1 and H_{10b} of ***syn*-5ab** have the *syn* relationship.

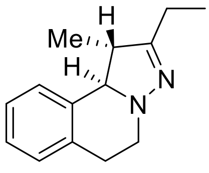

***anti*-5ab**

***anti*-5ab**: ^{1}H NMR (500 MHz, $CDCl_3$) δ 1.05 (t, *J* = 7.5 Hz, 3H), 1.35 (d, *J* = 7.3 Hz, 3H), 2.11-2.20 (m, 1H), 2.25-2.34 (m, 1H), 2.43-2.48 (m, 1H), 2.95-3.05 (m, 2H), 3.19-3.26 (m, 1H), 3.99 (dd, *J* = 13.7, 5.0 Hz, 1H), 4.42 (brs, 1H), 7.02 (d, *J* = 7.6 Hz, 1H), 7.06 (d, *J* = 7.7 Hz, 1H), 7.11-7.15 (m, 1H), 7.19-7.23 (m, 1H); ^{13}C NMR (125 MHz, $CDCl_3$) δ 11.2, 17.1, 22.1, 25.0, 47.6, 52.5, 68.9, 126.3, 126.4, 127.0, 128.8, 135.2, 138.0, 162.1; IR (neat) *ν* 2964, 2927, 1489, 1455, 1374, 1076, 924, 855, 746 cm^{-1}; Ms (ESI): *m*/*z* = 215.2 $[M+H]^+$. The relative configuration was assigned by NOESY analysis of this compound. NOE between the aromatic proton H_{10} of ***anti*-5ab** and H_1 was observed. Additionally, there was no observable NOE between H_{10} and H_{11}. These results indicate that H_1 and H_{10b} of ***anti*-5ab** have the *anti* relationship.

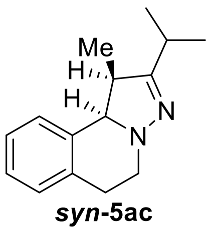

***syn*-5ac**

Compound **5ac**, yellow oil, yield: 70% (*syn* : *anti* = 3.3 : 1). ***syn*-5ac**: ^{1}H NMR (500 MHz, $CDCl_3$) δ 0.98 (d, *J* = 7.4 Hz, 3H), 1.12 (d, *J* = 7.0 Hz, 3H), 1.18 (d, *J* = 6.7 Hz, 3H), 2.48-2.54 (m, 2H),

2.99-3.13 (m, 2H), 3.37-3.44 (m, 1H), 3.91-3.96 (m, 1H), 4.70 (d, J = 10.0 Hz, 1H), 7.01 (d, J = 7.0 Hz, 1H), 7.07 (d, J = 7.0 Hz, 1H), 7.12-7.19 (m, 2H); ^{13}C NMR (125 MHz, $CDCl_3$) δ 12.9, 19.9, 20.9, 26.8, 28.1, 44.9, 49.1, 66.2, 125.7, 126.3, 128.4, 129.0, 133.8, 137.1, 166.2; IR (neat) ν 2965, 2929, 1491, 1455, 1360, 1073, 929, 747 cm^{-1}; Ms (ESI): m/z = 229.2 [M+H]$^+$.

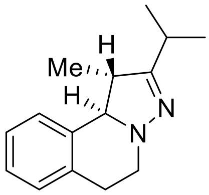

***anti*-5ac**

***anti*-5ac**: ^{1}H NMR (500 MHz, $CDCl_3$) δ 0.98 (d, J = 7.0 Hz, 3H), 1.11 (d, J = 6.8 Hz, 3H), 1.36 (d, J = 7.3 Hz, 3H), 2.41-2.55 (m, 2H), 2.96-3.08 (m, 2H), 3.18-3.25 (m, 1H), 3.99 (dd, J =13.9, 4.8 Hz, 1H), 4.40 (brs, 1H), 7.01 (d, J = 7.6 Hz, 1H), 7.06 (d, J = 7.7 Hz, 1H), 7.10-7.14 (m, 1H), 7.18-7.22 (m, 1H); ^{13}C NMR (125 MHz, $CDCl_3$) δ 17.5, 20.3, 21.3, 25.0, 28.5, 47.6, 51.2, 69.3, 126.2, 126.4, 126.9, 128.7, 135.2, 137.7, 165.7; IR (neat) ν 2962, 2927, 1489, 1456, 1362, 1076, 927, 745 cm^{-1}; Ms (ESI): m/z = 229.2 [M+H]$^+$.

Scheme S1. Proposed mechanism of stereoselectivity of 2a

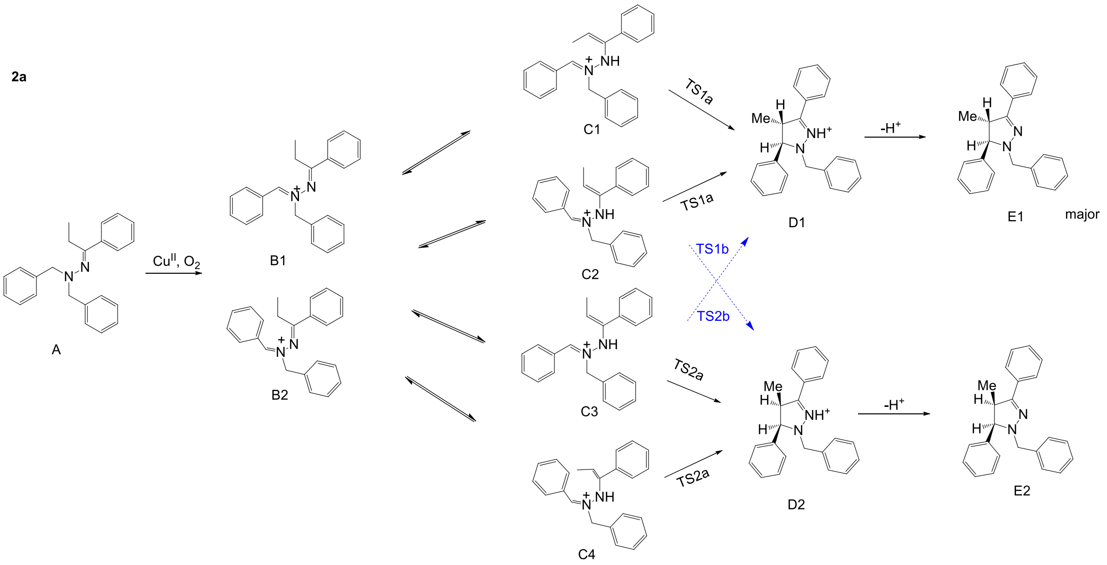

Figure S1. Optimized geometries in 5-center/6-electron cyclization generating 2a

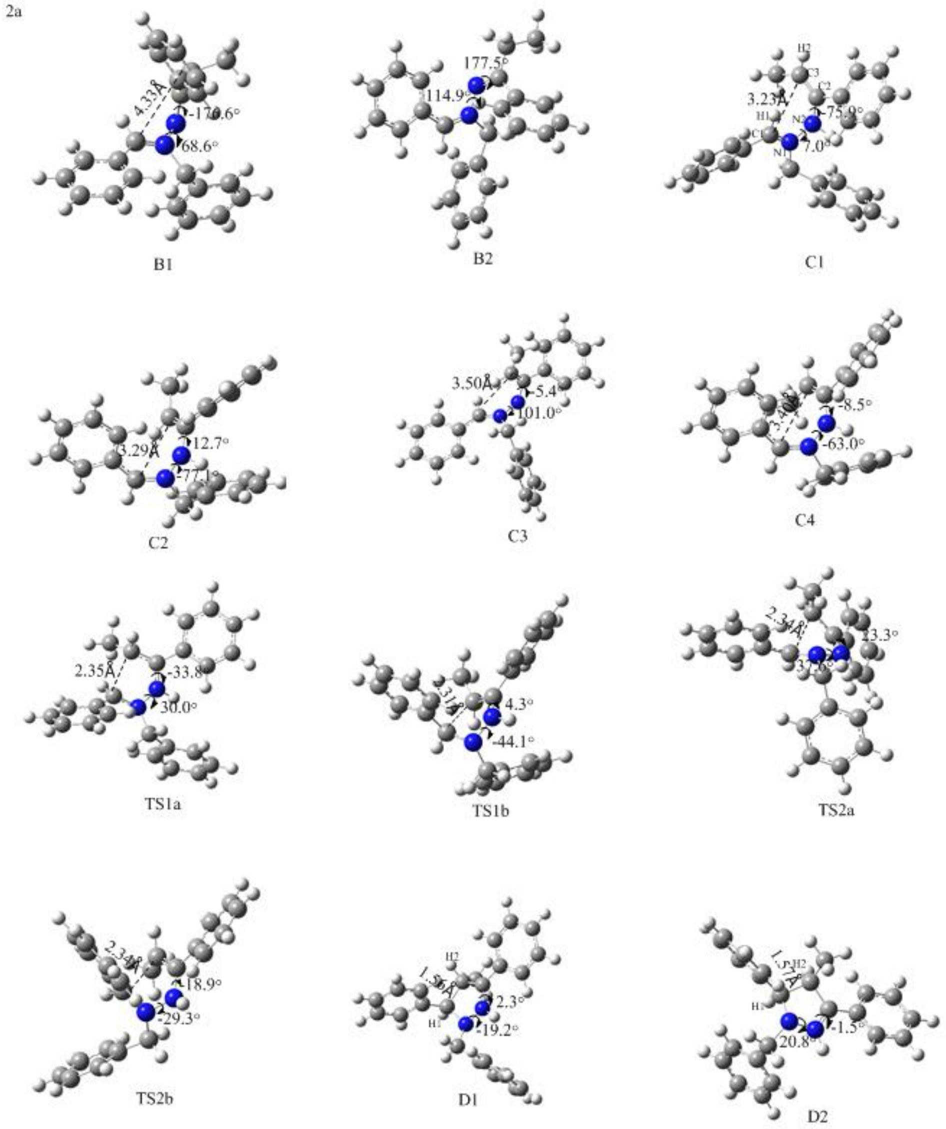

Scheme S2. Proposed mechanism of stereoselectivity of 2f

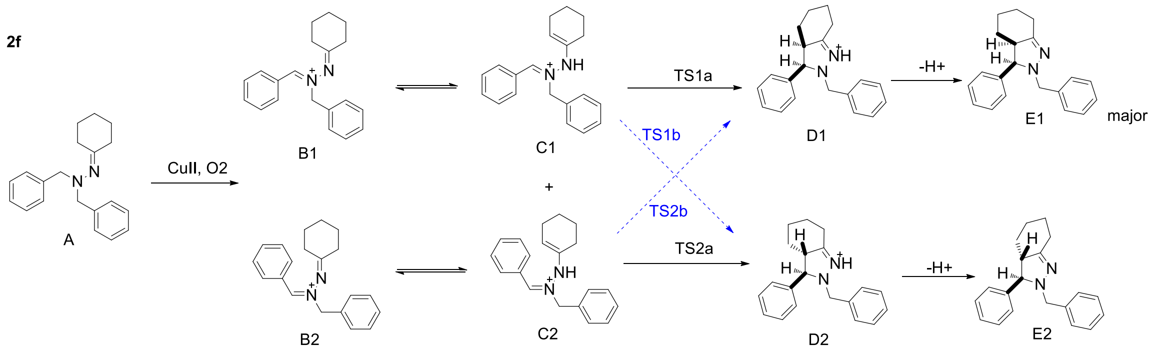

Figure S2. Optimized geometries in 5-center/6-electron cyclization generating 2f

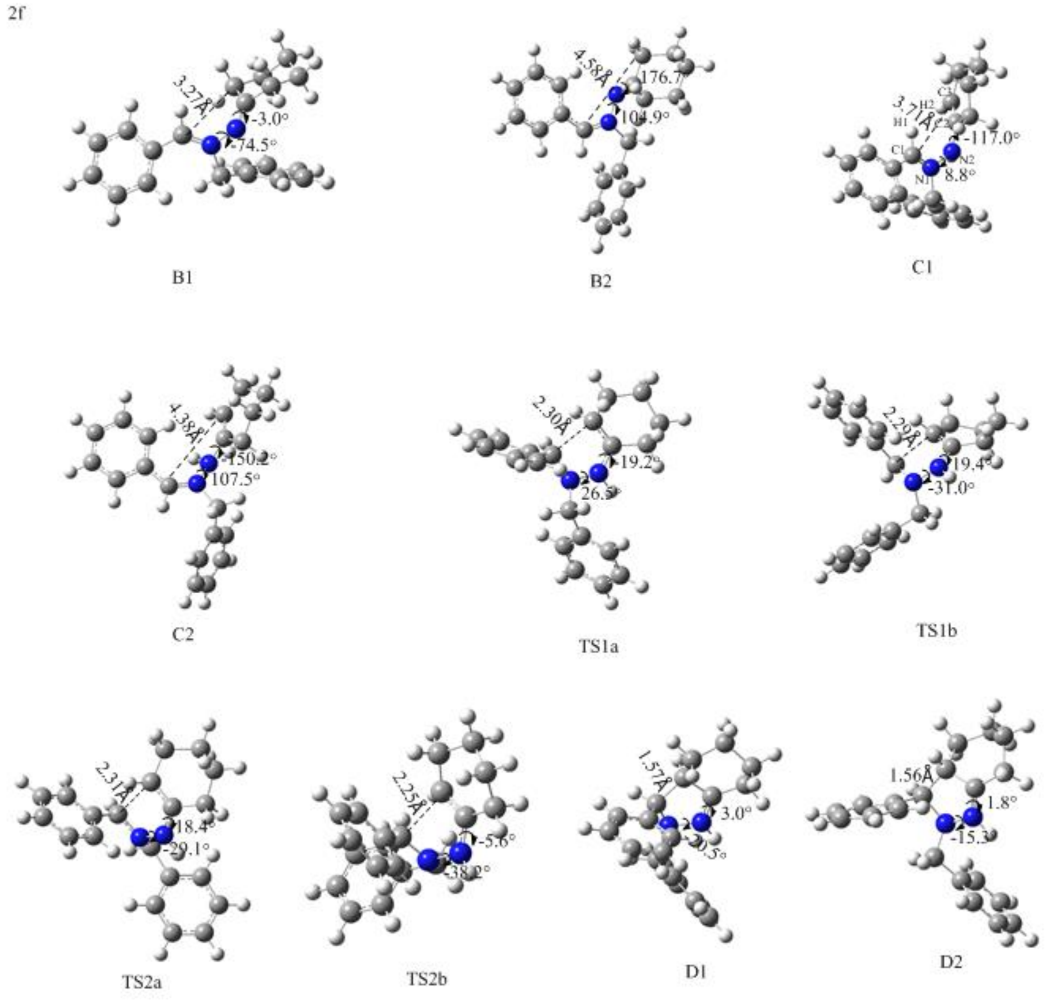

Figure S3. Free energy profiles in 5-center/6-electron cyclization process generating 2f in Scheme 3 in the gas phase. Free energies in the solution phase are given in parentheses.

C1 preferentially goes through disrotatory process (**TS1a**) to give **D1** (to be exothermic by 8.9 kcal/mol with a free energy barrier of 24.0 kcal/mol), rather than conrotatory process (**TS1b**) to afford **D2**. Also, **C2** is more likely to go through disrotatory process (**TS2a**) to give **D2**, rather than conrotatory process (**TS2b**) to afford **D1**. Compared with **C2**, **C1** overcome less free energy barrier, determining the stereoselectivity of this reaction. The overall selective picture is the same as that captured from gas-phase calculations. These are in good agreement with the experimental finding.

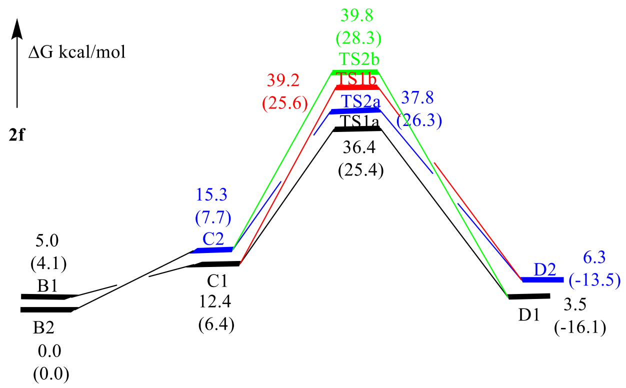

Scheme S3. Proposed mechanism of stereoselectivity of 2p

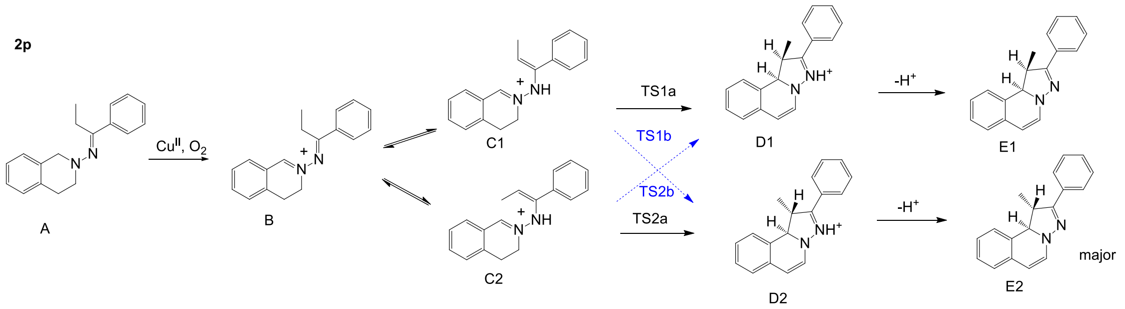

Figure S4. Optimized geometries in 5-center/6-electron cyclization generating 2p.

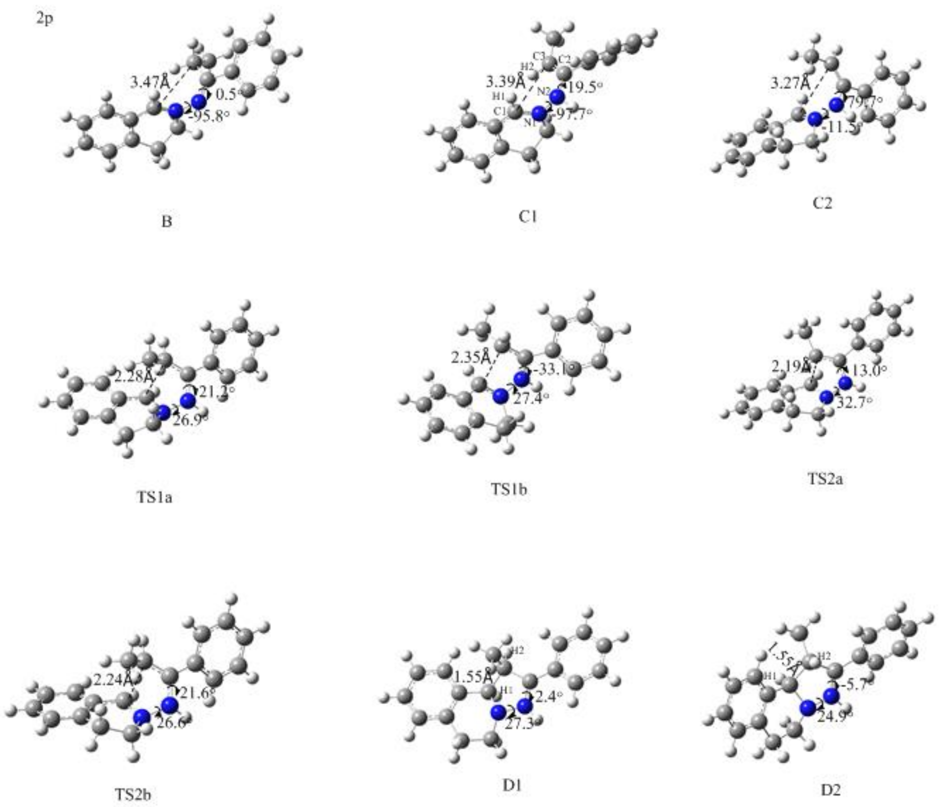

Figure S5. Free energy profiles in 5-center/6-electron cyclization generating 2p in Scheme 3 in the gas phase. Free energies in the solution phase are given in parentheses.

C2 prefers to undergo disrotatory process (**TS2a**) to give **D2** (to be exothermic by 3.2 kcal/mol with a free energy barrier of 26.4 kcal/mol), rather than conrotatory process (**TS2b**) to afford **D1**. However, **C1** is more likely undergo conrotatory process (**TS1b**) to afford **D2**, rather than disrotatory process (**TS1a**) to give **D1**. Compared with **C1**, **C2** overcome less energy barrier, determining the stereoselectivity of this reaction. The overall selective picture is the same as that captured from gas-phase calculations. These are in good agreement with the experimental finding.

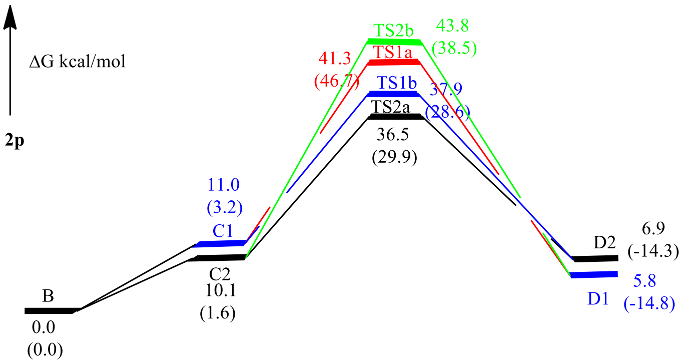

Scheme S4. Proposed mechanism of stereoselectivity of 5a

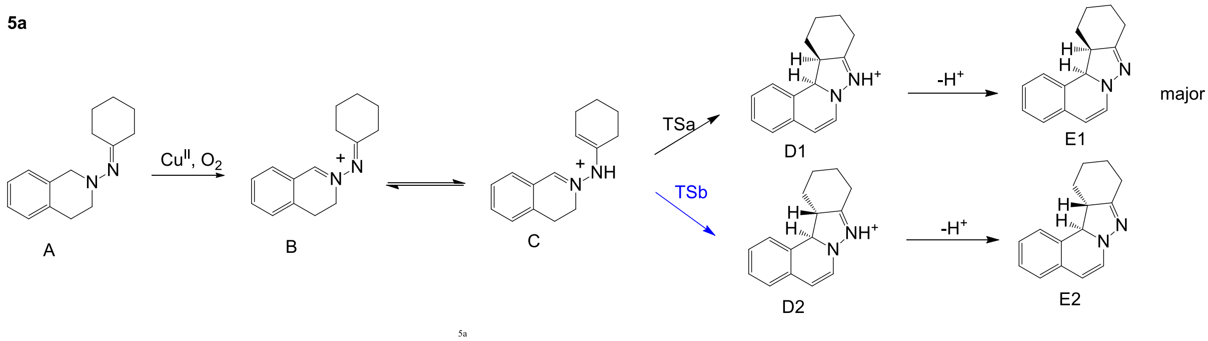

Figure S6. Optimized geometries in 5-center/6-electron cyclization generating 5a

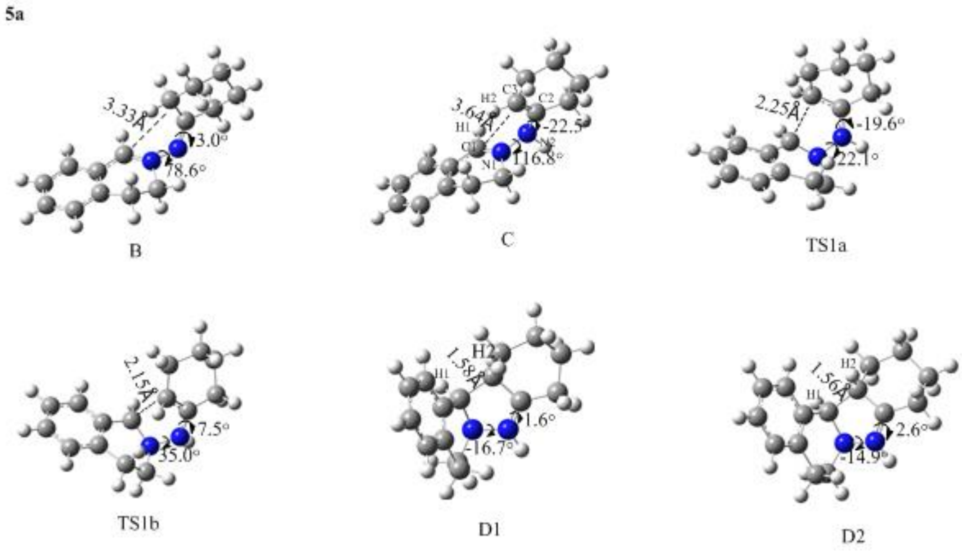

Scheme S5 Proposed mechanism of stereoselectivity of 5ab

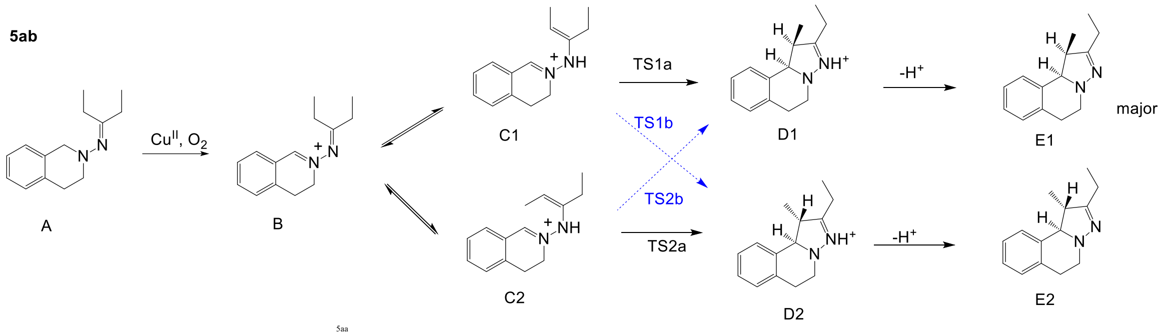

Figure S7. Optimized geometries in 5-center/6-electron cyclization generating 5ab

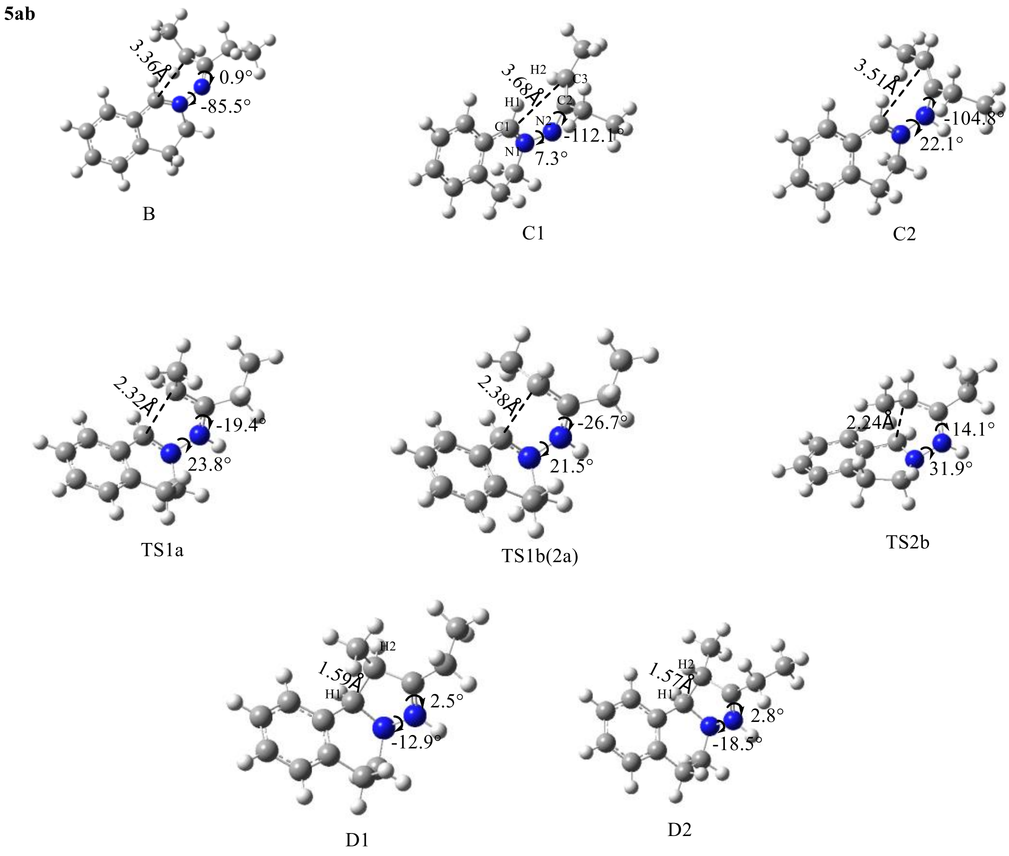

Figure S8. Free energy profiles of the reaction generating 5ab in scheme 3 in the gas phase. Free energies in the solution phase are given in parentheses.

C1 tends to undergo disrotatory process (**TS1a**) to give **D1** (to be exothermic by 2.8 kcal/mol with a free energy barrier of 25.7 kcal/mol), rather than conrotatory process (**TS1b**) to afford **D2**. Also, **C2** is more likely to undergo disrotatory process (**TS2a**) to give **D2**, rather than conrotatory process (**TS2b**) to afford **D1**. However, compared with **C2**, **C1** overcome less free energy barrier, determining the stereoselectivity of this reaction. The overall stereoselective picture is the same as that captured from gas-phase calculations. These are in good agreement with the experimental finding.

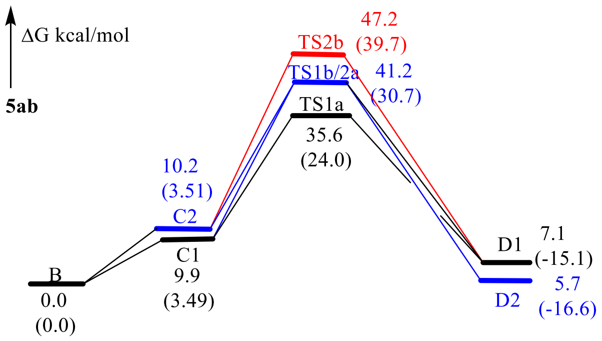

Figure S9. HOMOs of TS1a, TS1b and TS2b generating 5ab

To better understand this point, HOMOs of **TS1a, TS1b(=TS2a) and TS2b** were compared. Seen from **Figure S4**, the under lobe of orbital of C atom in C=N$^+$ bond overlaps with the under lobe of orbital of the terminal C atom in C=C bond, and C=N$^+$-NH-C=C and CH_3 group constitutes a delocalized system in **TS1a**. In **TS1b** the under lobe of orbital of C atom in C=N$^+$ bond overlaps with the under lobe of orbital of the terminal C atom in C=C bond, but the delocalized system is only consisted of C=N$^+$-NH-C=C. The delocalized system in **TS1b** is smaller than **TS1a,** suggesting that **TS1b** is less stable and more difficult to overcome than **TS1a**. Compared with the orbital in **TS2a**, the C=N$^+$-NH-C=C in **TS2b** do not constitute a complete delocalized orbital, and an orbital nodal surface is found between NH and the terminal C atom in C=C bond, also suggesting that **TS2b** is less stable and more difficult to cross than **TS2a**. Therefore, **C1 (C2)** goes through disrotatory process to form **D1(D2)**, instead of conrotatory process to afford **D2 (D1).**

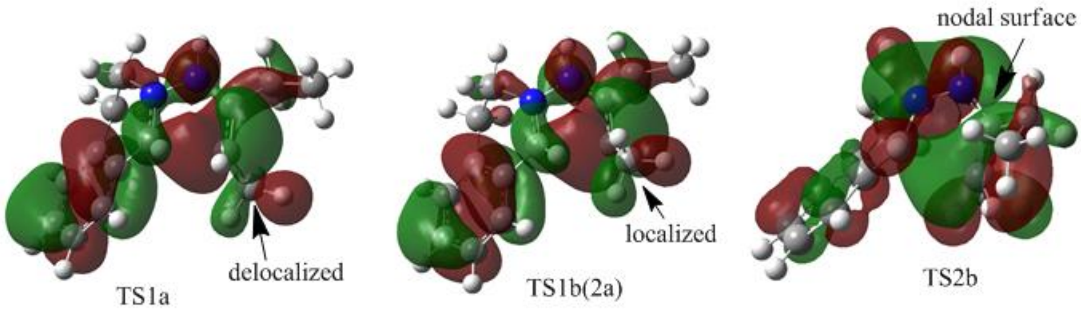

Table S1. Electronic energies (E*elec*), Zero-point energies (E_{zp}), Gibbs free energies (G*gas*), and solvent free energies (ΔG*sol*) of all stationary points. The E*elec* , G*gas* , and E_{zp} energies are in a.u. and ΔG*sol* are in kcal/mol.

	E_{elec}	E_{ZP}	G_{gas}	ΔG
2a				
B1-2a	-999.6081	0.40334	-999.2607	-63.18
B2-2a	-999.6158	0.4038	-999.2682	-60.1
C1-2a	-999.5989	0.40406	-999.249	-67.41
C2-2a	-999.5977	0.40426	-999.2476	-71.21
C3-2a	-999.5956	0.40417	-999.2455	-55.85
C4-2a	-999.5928	0.40432	-999.2414	-56.12
TS1a-2a	-999.5613	0.40355	-999.2105	-70.43
TS1b-2a	-999.5546	0.40168	-999.2052	-70.16
TS2a-2a	-999.5488	0.40232	-999.1988	-67.9
TS2b-2a	-999.5439	0.4021	-999.1942	-69.51
D1-2a	-999.627	0.40628	-999.2735	-55.54
D2-2a	-999.6245	0.40615	-999.2715	-57.8
2f				
B1-2f	-885.29183	0.388509	-884.953336	-63.0
B2-2f	-885.30011	0.388854	-884.961256	-62.09
C1-2f	-885.28027	0.388834	-884.941425	-68.18
C2-2f	-885.27445	0.388801	-884.936888	-69.65
TS1a-2f	-885.24287	0.388384	-884.903197	-73.14
TS1b-2f	-885.23667	0.387067	-884.898725	-75.68
TS2a-2f	-885.2391	0.387225	-884.900972	-73.59
TS2b-2f	-1770.1337	0.386677	-884.897871	-73.56
D1-2f	-885.29821	0.390767	-884.955698	-81.63
D2-2f	-885.29216	0.390498	-884.951221	-81.92
2p				
B-2p	-806.6697	0.33059	-806.3859	-59.29
C1-2p	-806.6522	0.33052	-806.3684	-67.09
C2-2p	-806.6543	0.33066	-806.3698	-67.79
TS1a-2p	-806.6061	0.3299	-806.32	-64.49
TS1b-2p	-806.6114	0.33025	-806.3254	-68.69
D2-2p	-806.6637	0.33265	-806.3749	-80.47
TS2a-2p	-806.613	0.32932	-806.3277	-65.91
TS2b-2p	-806.6022	0.33	-806.3161	-64.57
D1-2p	-806.6656	0.33285	-806.3767	-79.91
D2-2p	-806.6637	0.33265	-806.3749	-80.47
5a				
B-5a	-692.3511	0.31511	-692.0784	-60.21
C1-5a	-692.3341	0.31535	-692.061	-67.36

TS1a-5a	-692.2921	0.31463	-692.018	-72.35
TS1b-5a	-692.2895	0.31412	-692.0154	-69.41
D1-5a	-692.3348	0.31741	-692.0581	-82.61
D2-5a	-692.3455	0.3176	-692.0679	-82.23
5ab				
B-5ab	-654.2375	0.3059	-653.9758	-59.18
C1-5ab	-654.2225	0.30594	-653.96	-65.57
C2-5ab	-654.222	0.30597	-653.9596	-65.84
TS1a-5ab	-654.1824	0.30528	-653.919	-70.83
TS1b/2a-5ab	-654.1739	0.30558	-653.9101	-69.7
TS2b-5ab	-654.1644	0.30532	-653.9006	-66.62
D1-5ab	-654.2322	0.30879	-653.9645	-81.36
D2-5ab	-654.2336	0.3085	-653.9667	-81.47

References

(1) G. Zhang, Y. Zhao and H. Ge, *Angew. Chem., Int., Ed.*, 2013, **52**, 2559.

(2) D. Fischer and E. A. Theodorakis, *Eur. J. Org. Chem.*, 2007, 4193.

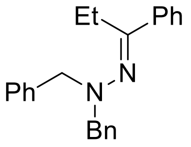

1a

^1H NMR (500 MHz, $CDCl_3$)

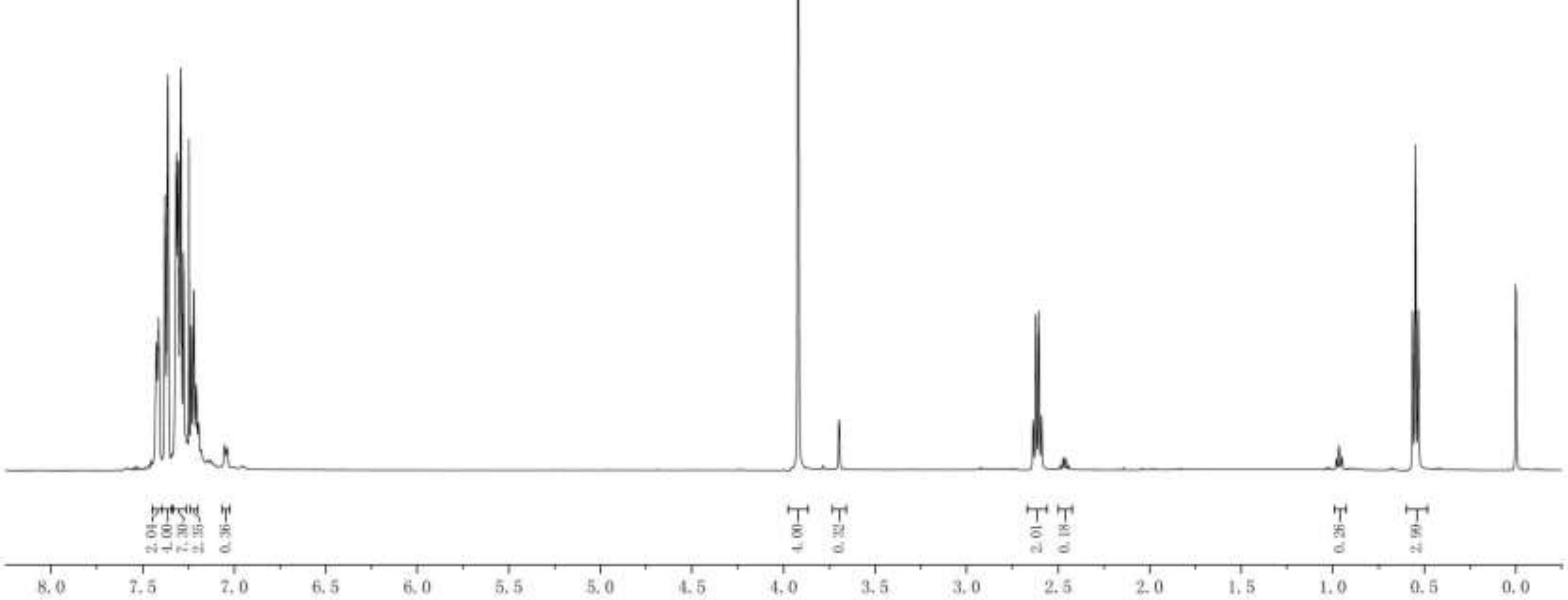

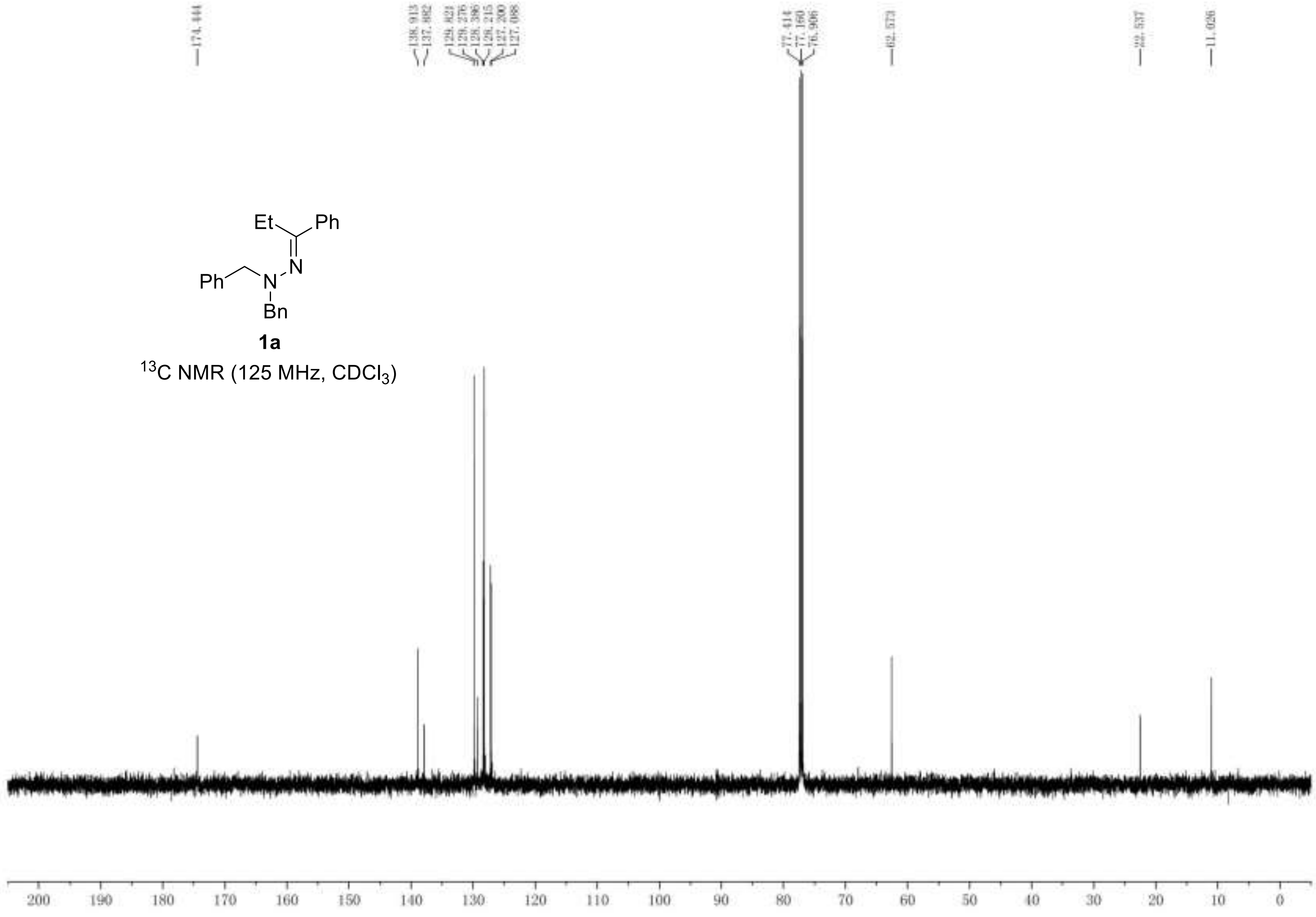

1a

^{13}C NMR (125 MHz, $CDCl_3$)

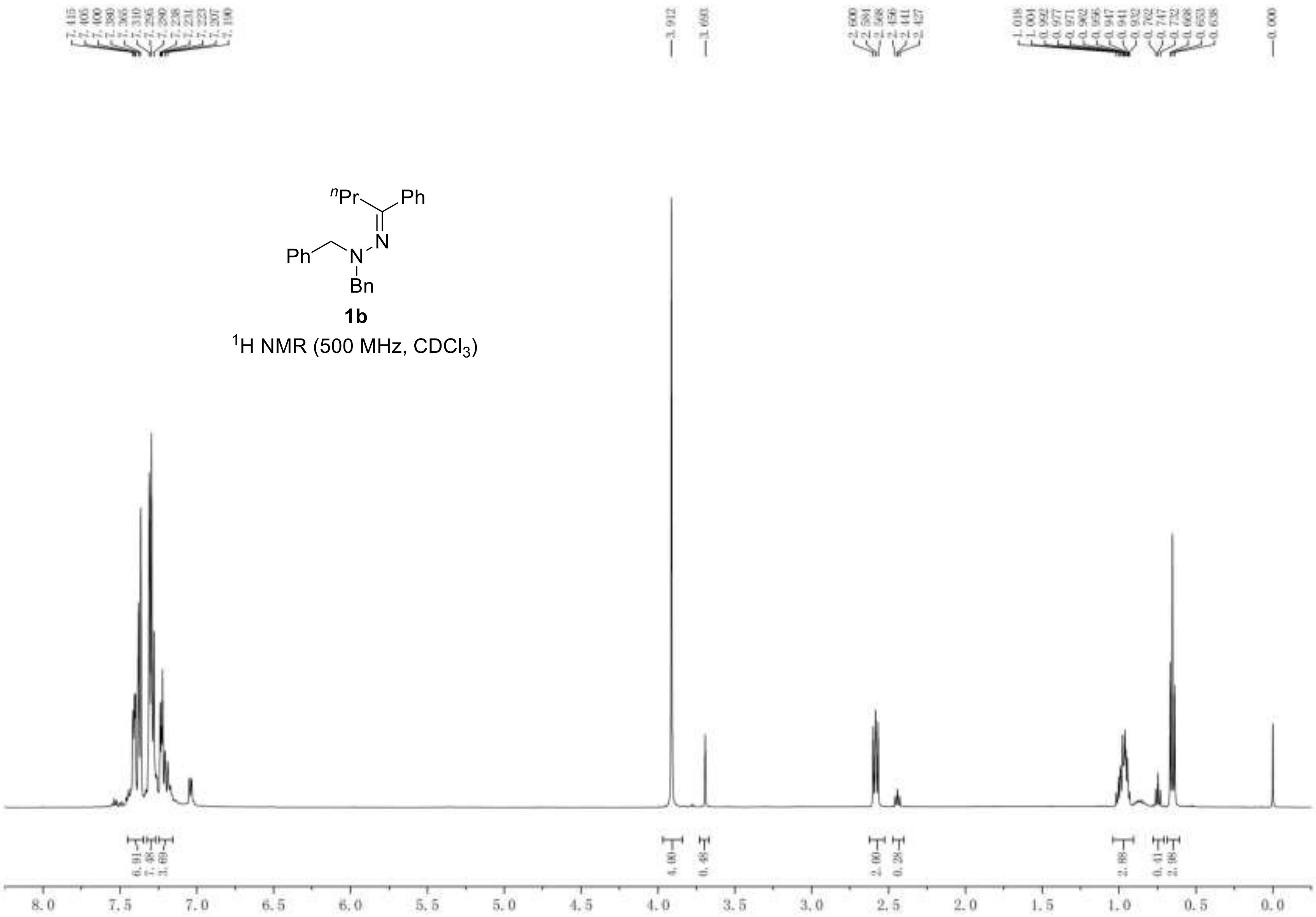

1b

^1H NMR (500 MHz, $CDCl_3$)

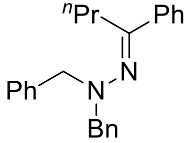

1b

^{13}C NMR (125 MHz, $CDCl_3$)

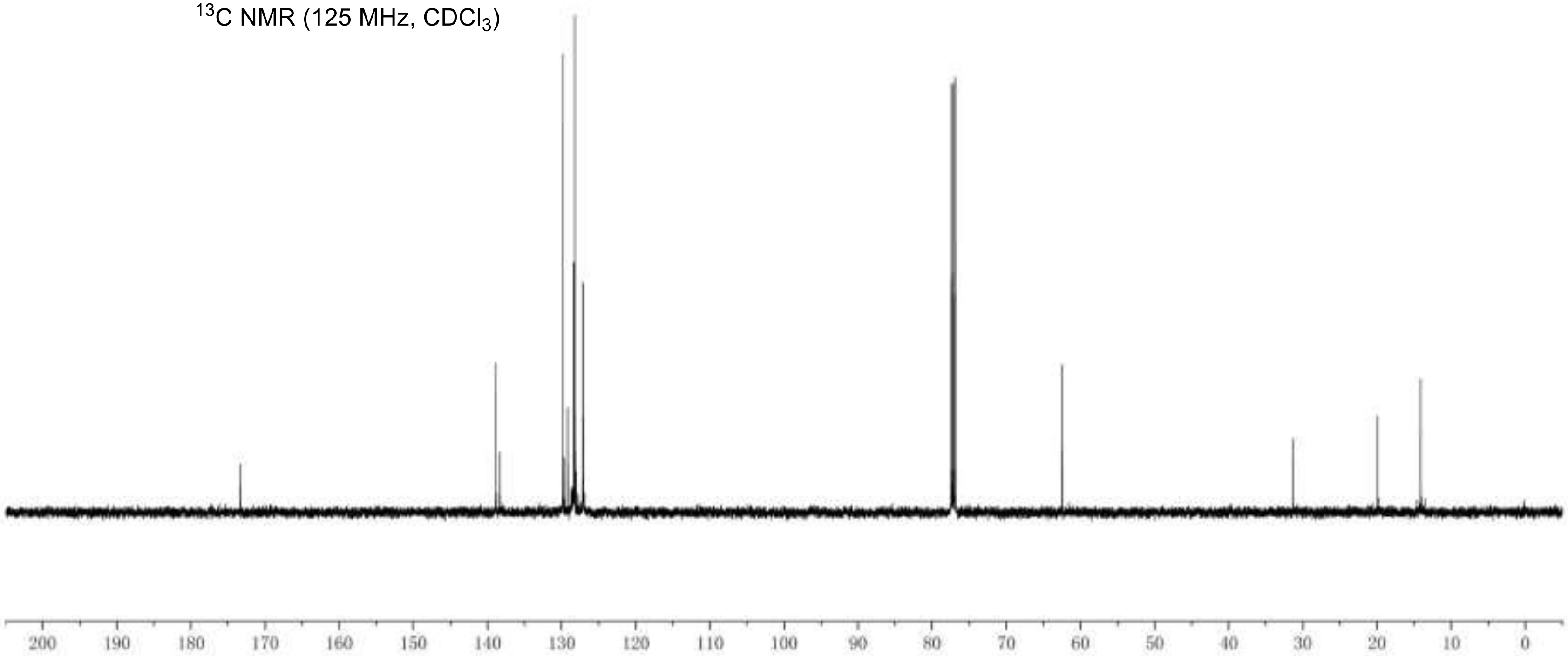

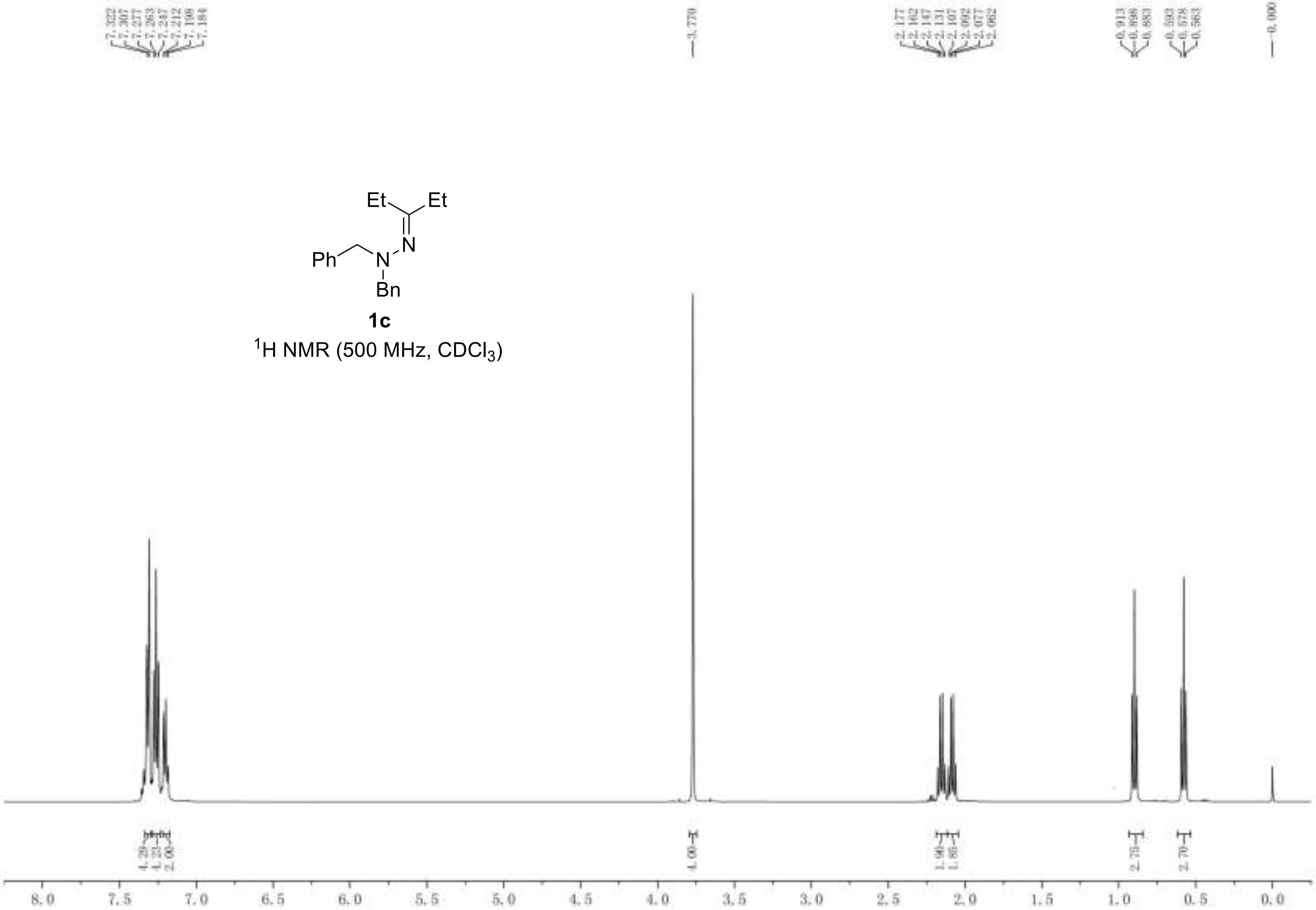

1c

^{1}H NMR (500 MHz, $CDCl_3$)

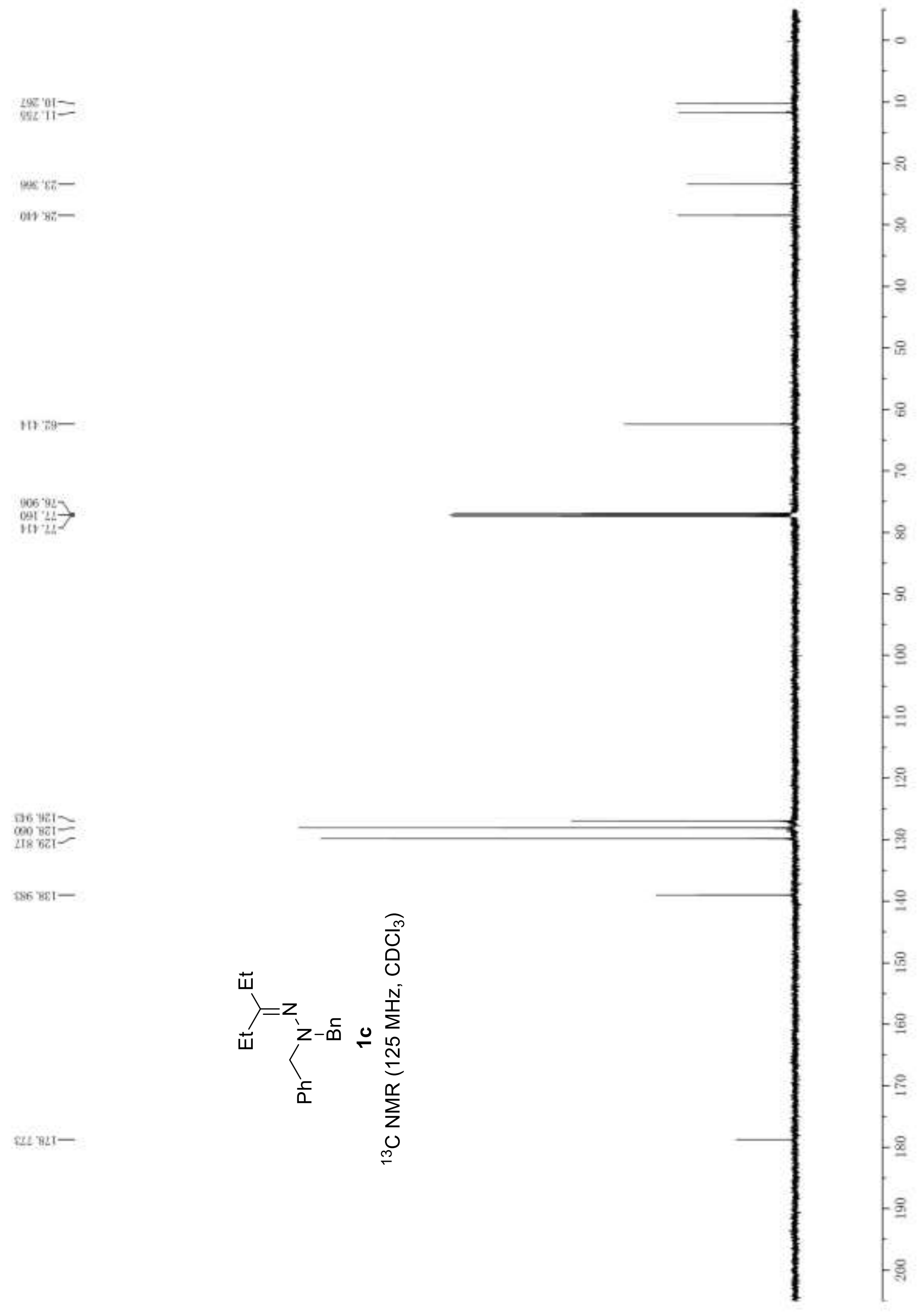

1c

^{13}C NMR (125 MHz, $CDCl_3$)

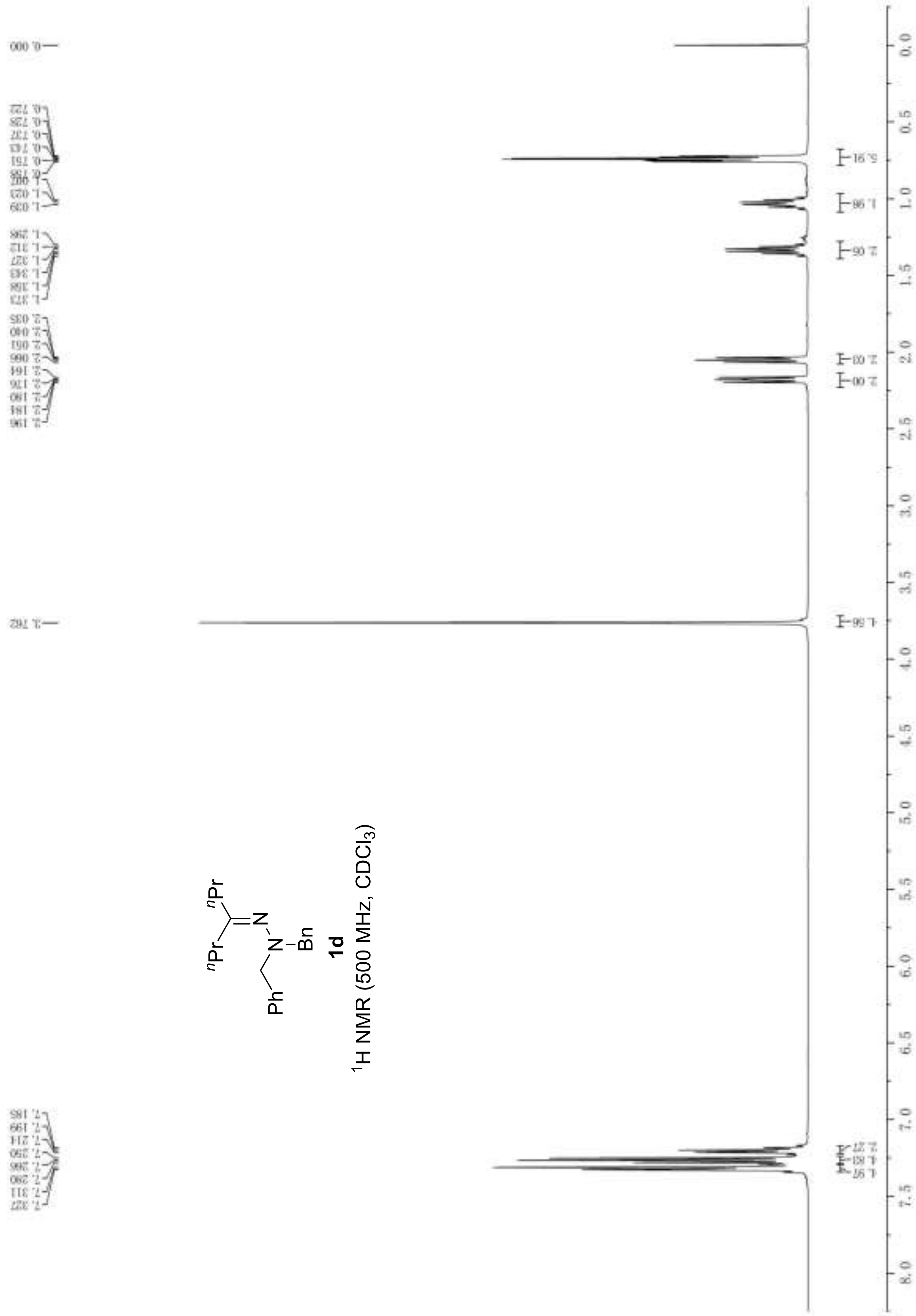
nPr
nPr
N
N
Bn
Ph
1d
1H NMR (500 MHz, CDCl3)

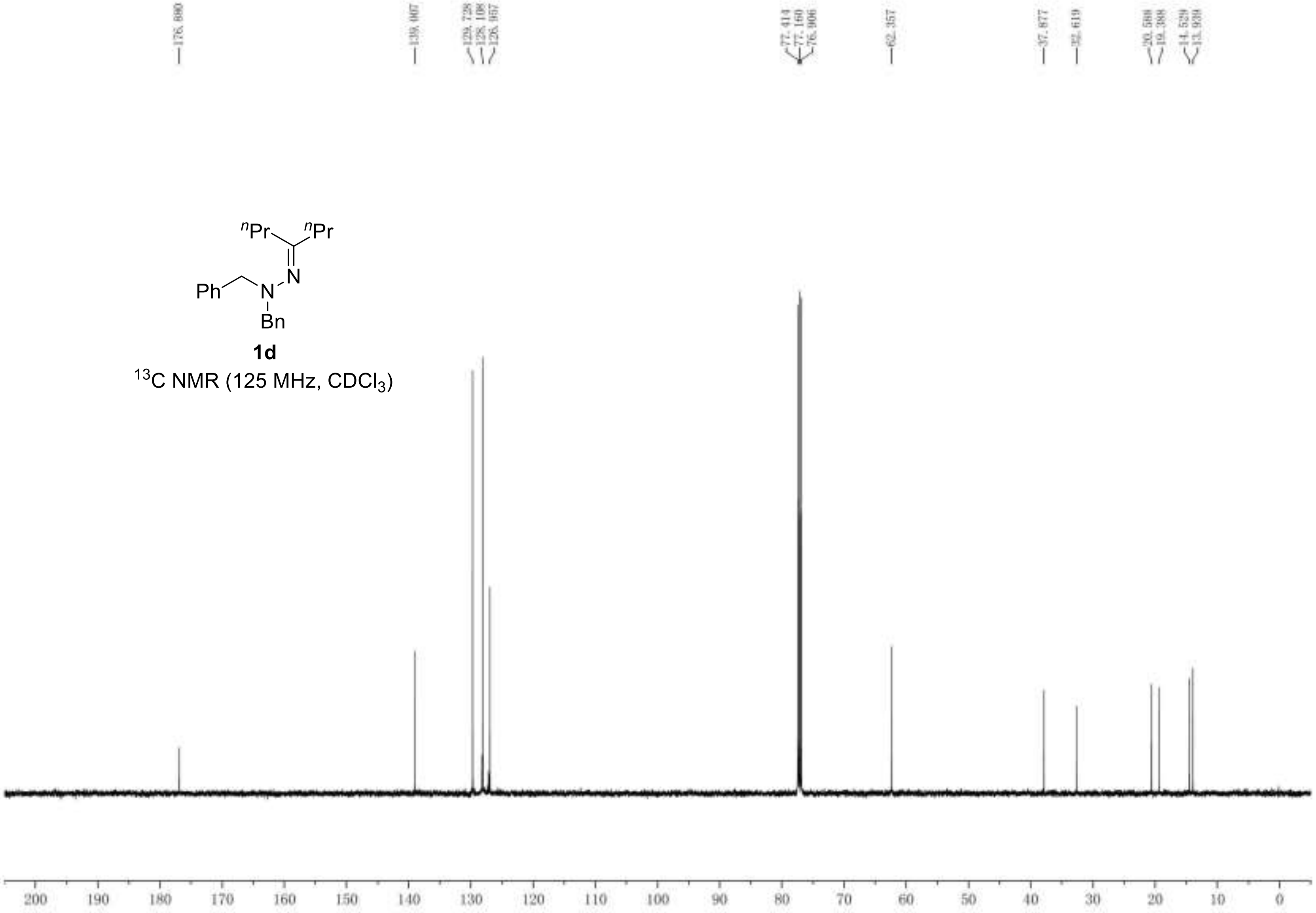

1d

^{13}C NMR (125 MHz, $CDCl_3$)

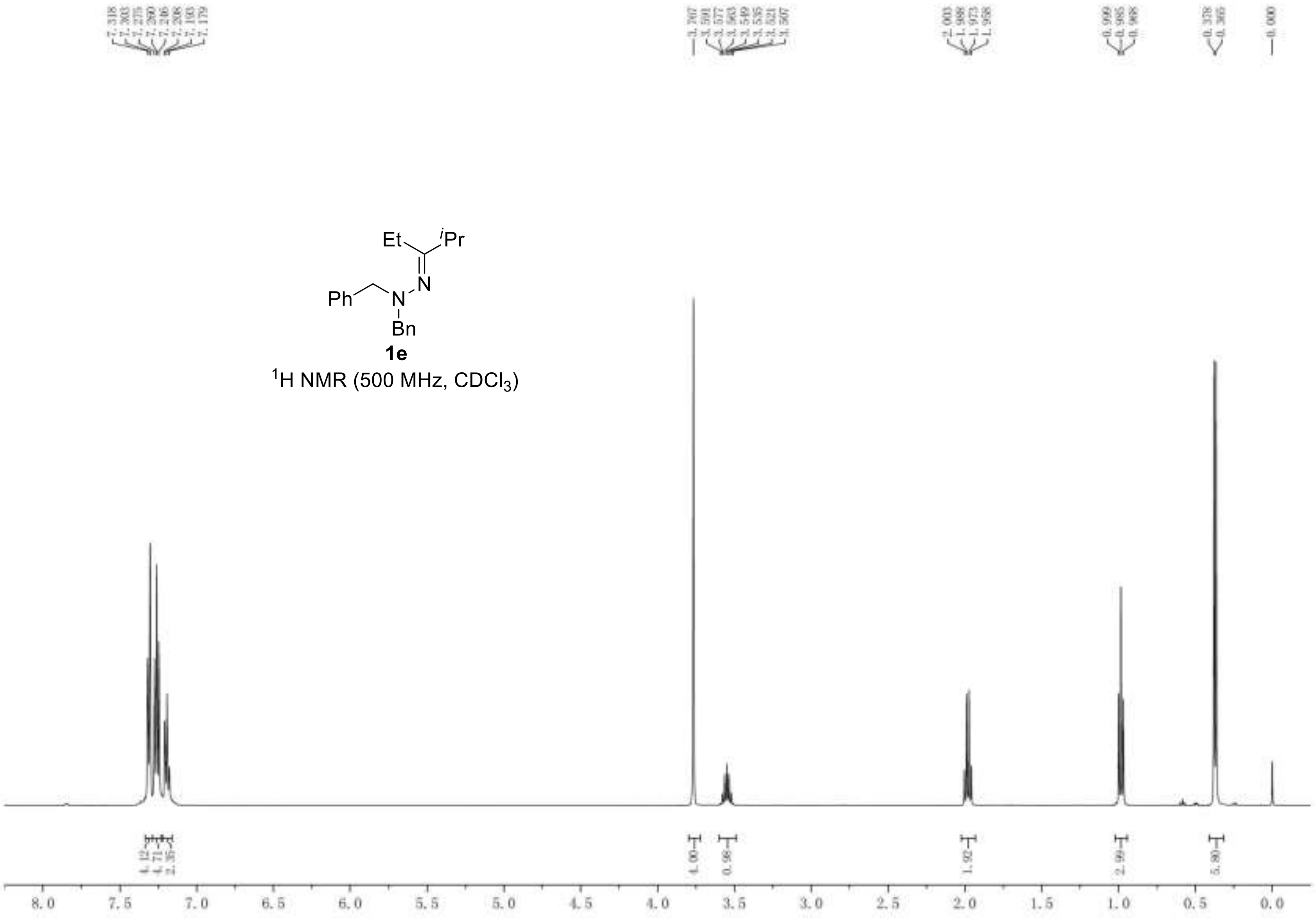

1e

^{1}H NMR (500 MHz, $CDCl_3$)

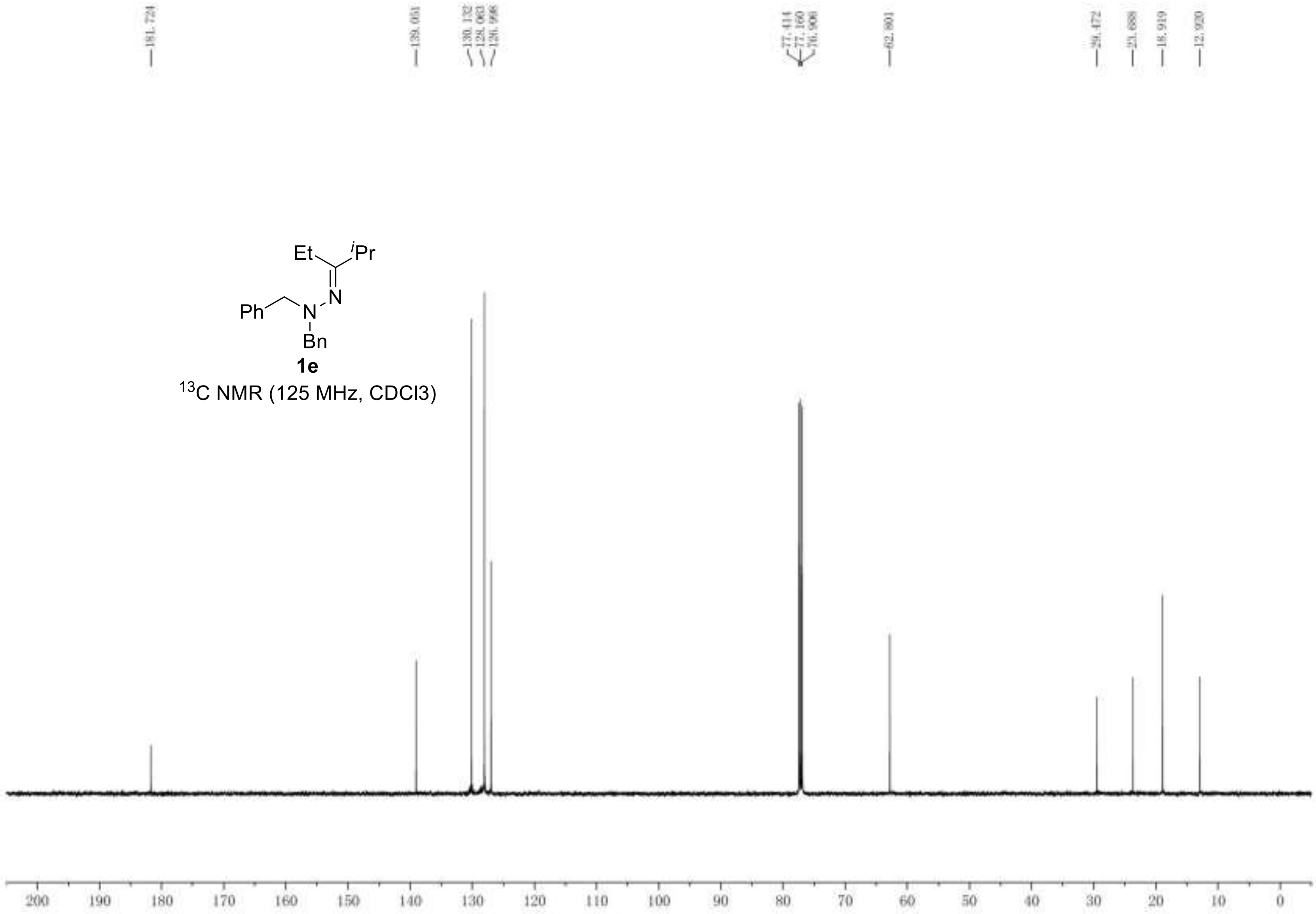

1e

^{13}C NMR (125 MHz, CDCl3)

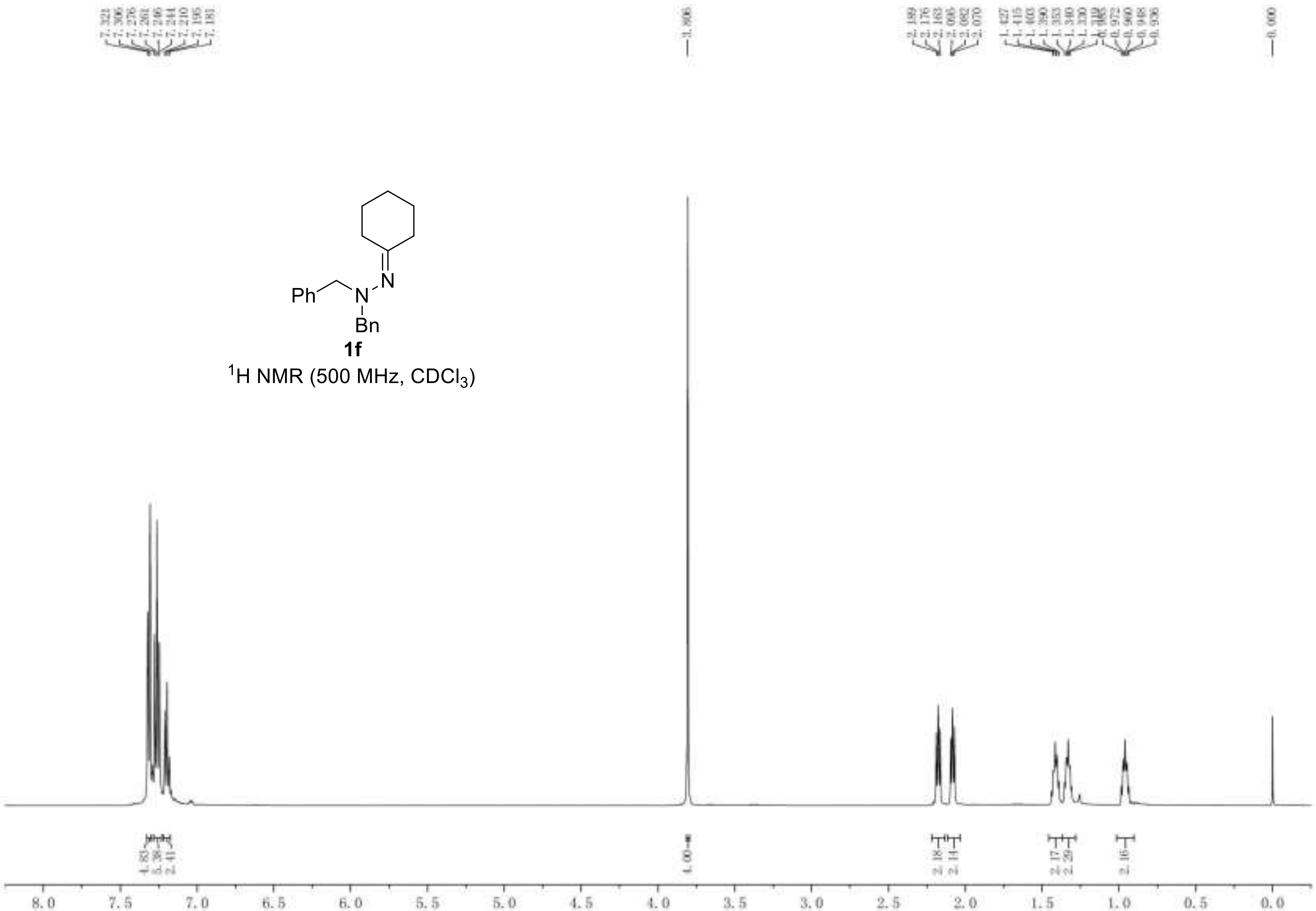

1f

^{1}H NMR (500 MHz, CDCl$_3$)

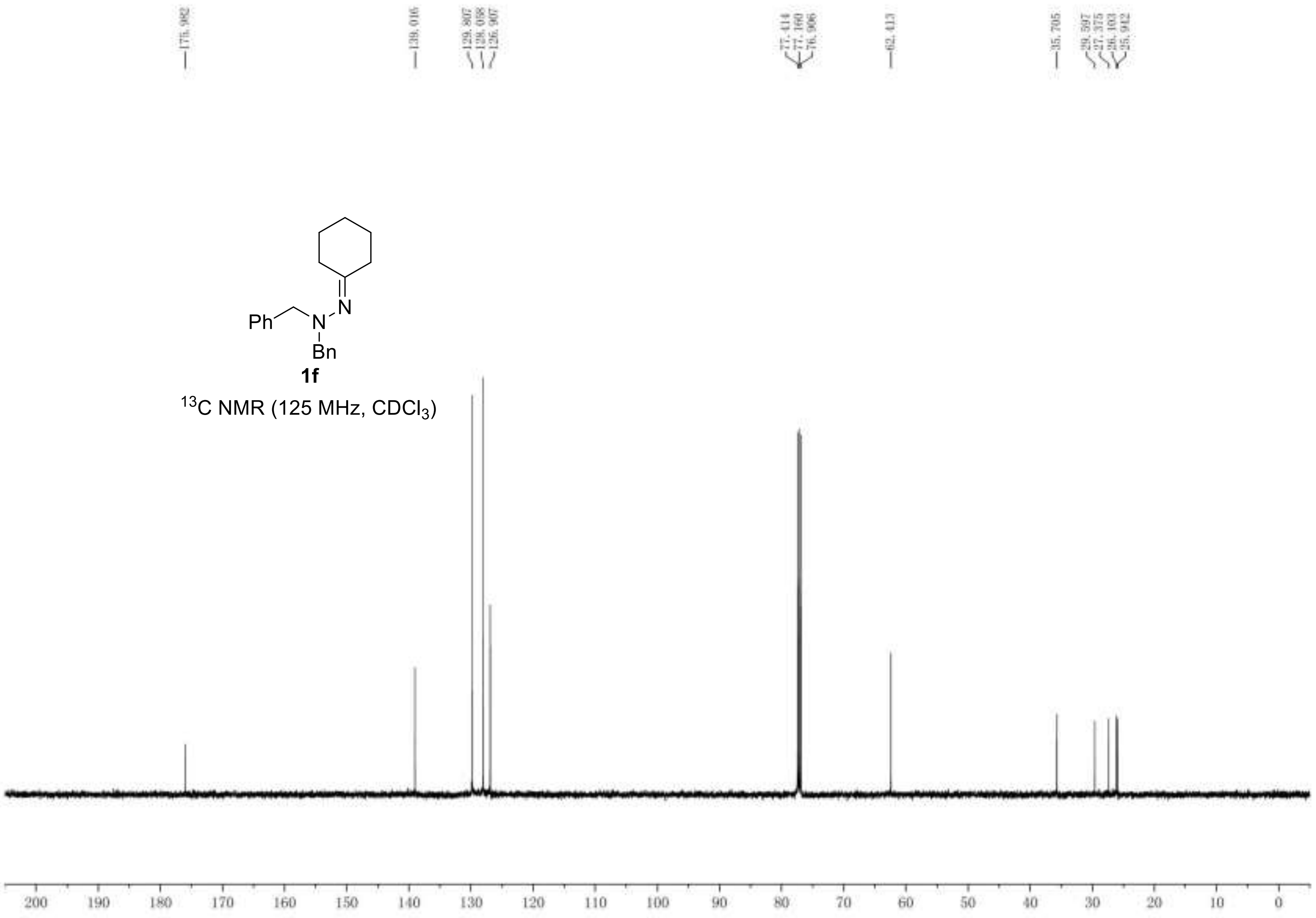

1f

^{13}C NMR (125 MHz, $CDCl_3$)

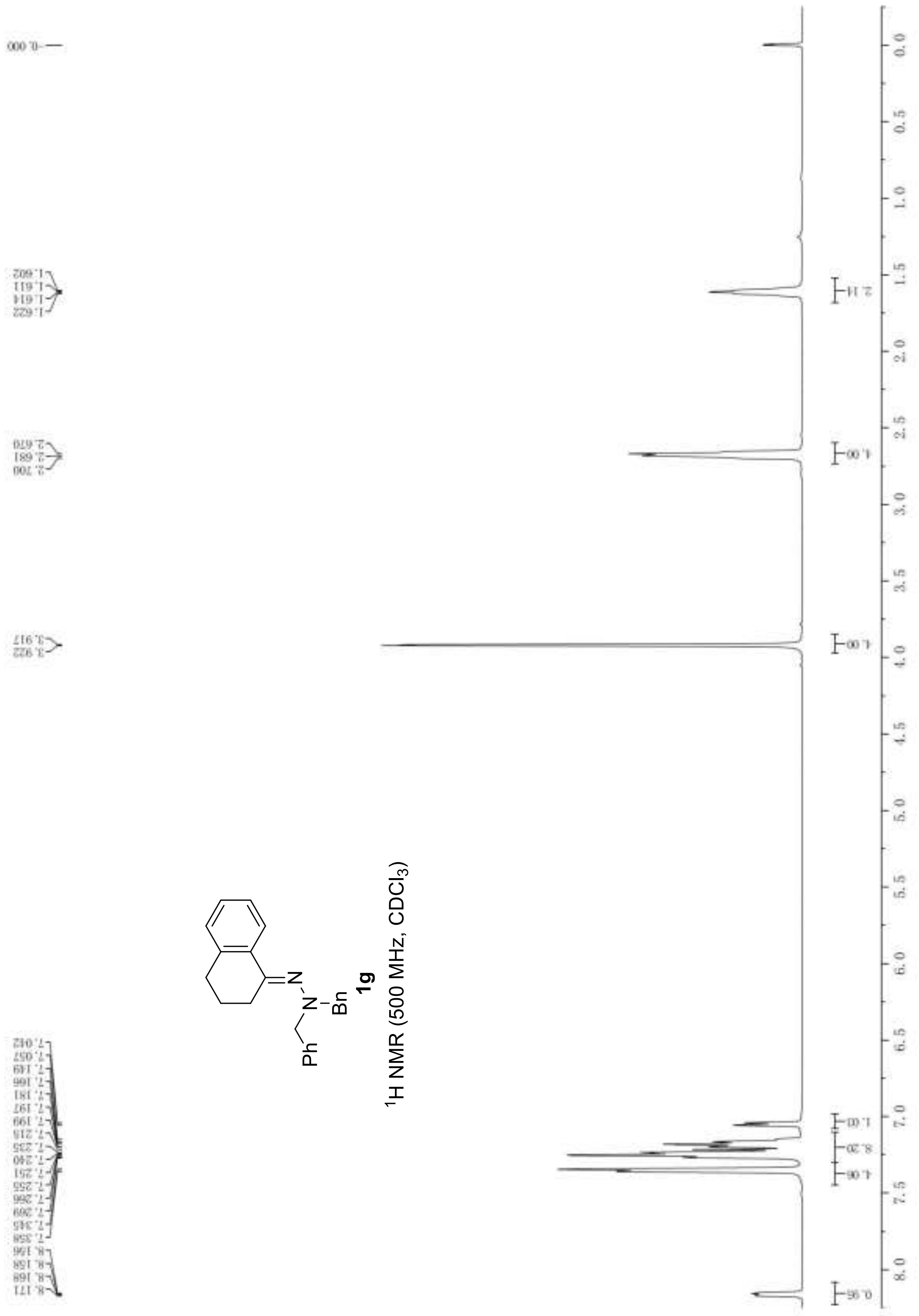

1g

^{1}H NMR (500 MHz, $CDCl_3$)

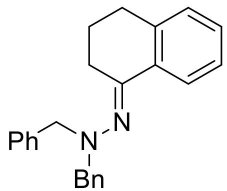

1g

^{13}C NMR (125 MHz, $CDCl_3$)

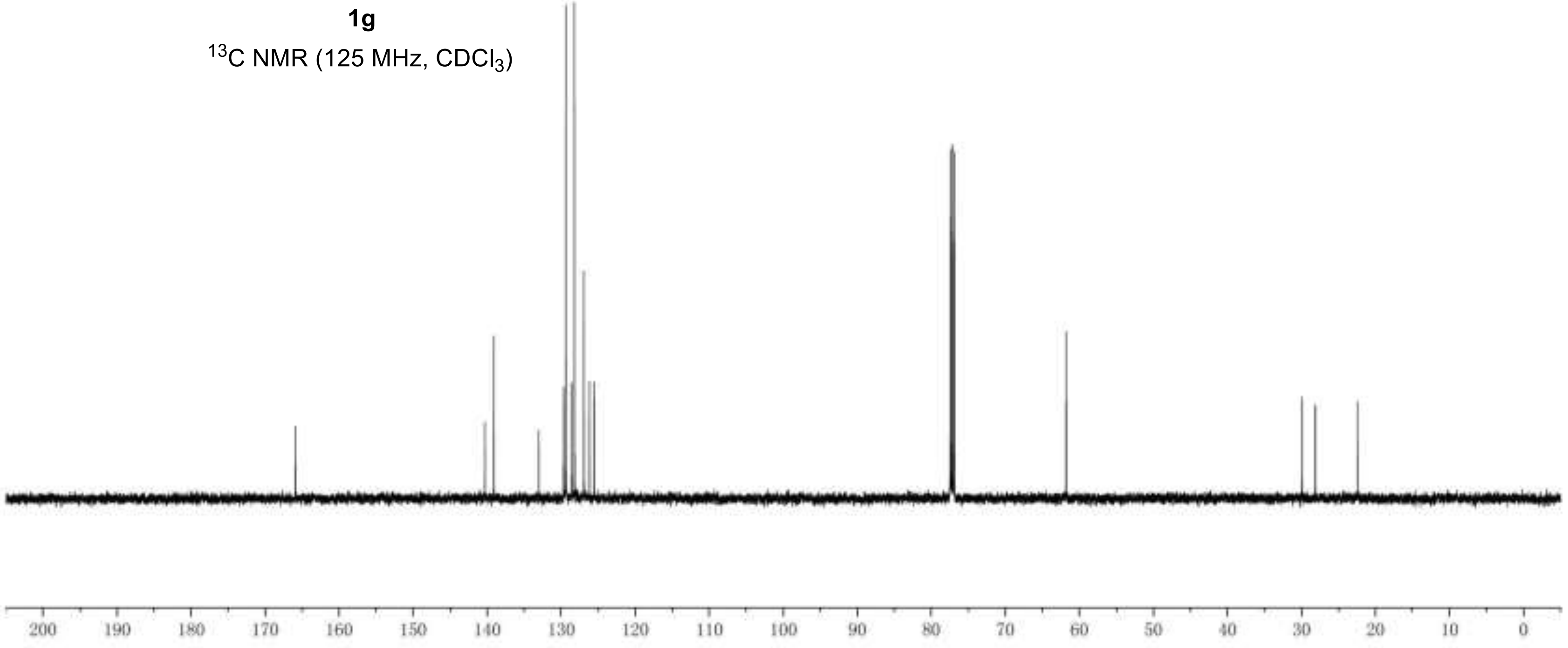

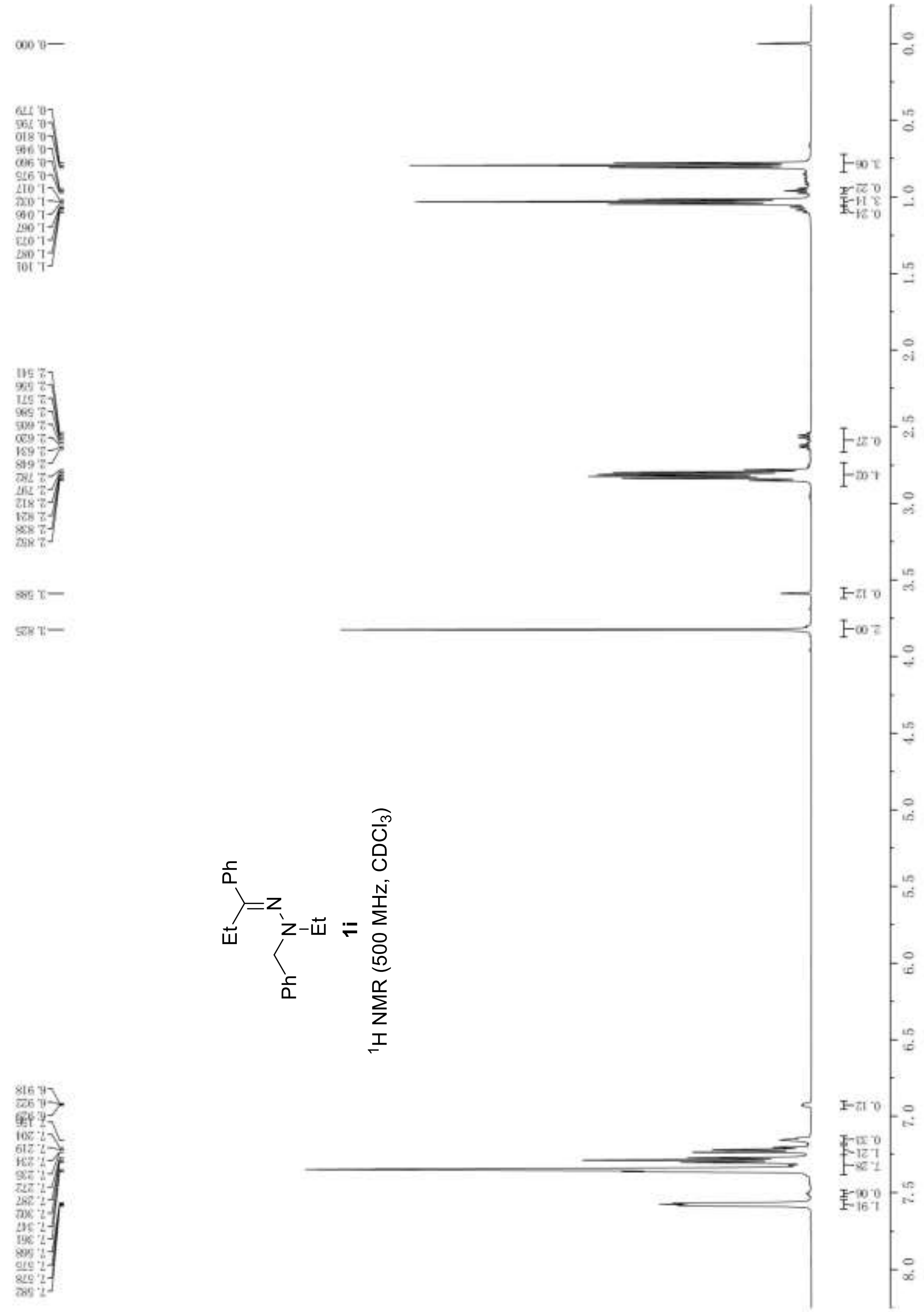

1i

^{1}H NMR (500 MHz, $CDCl_3$)

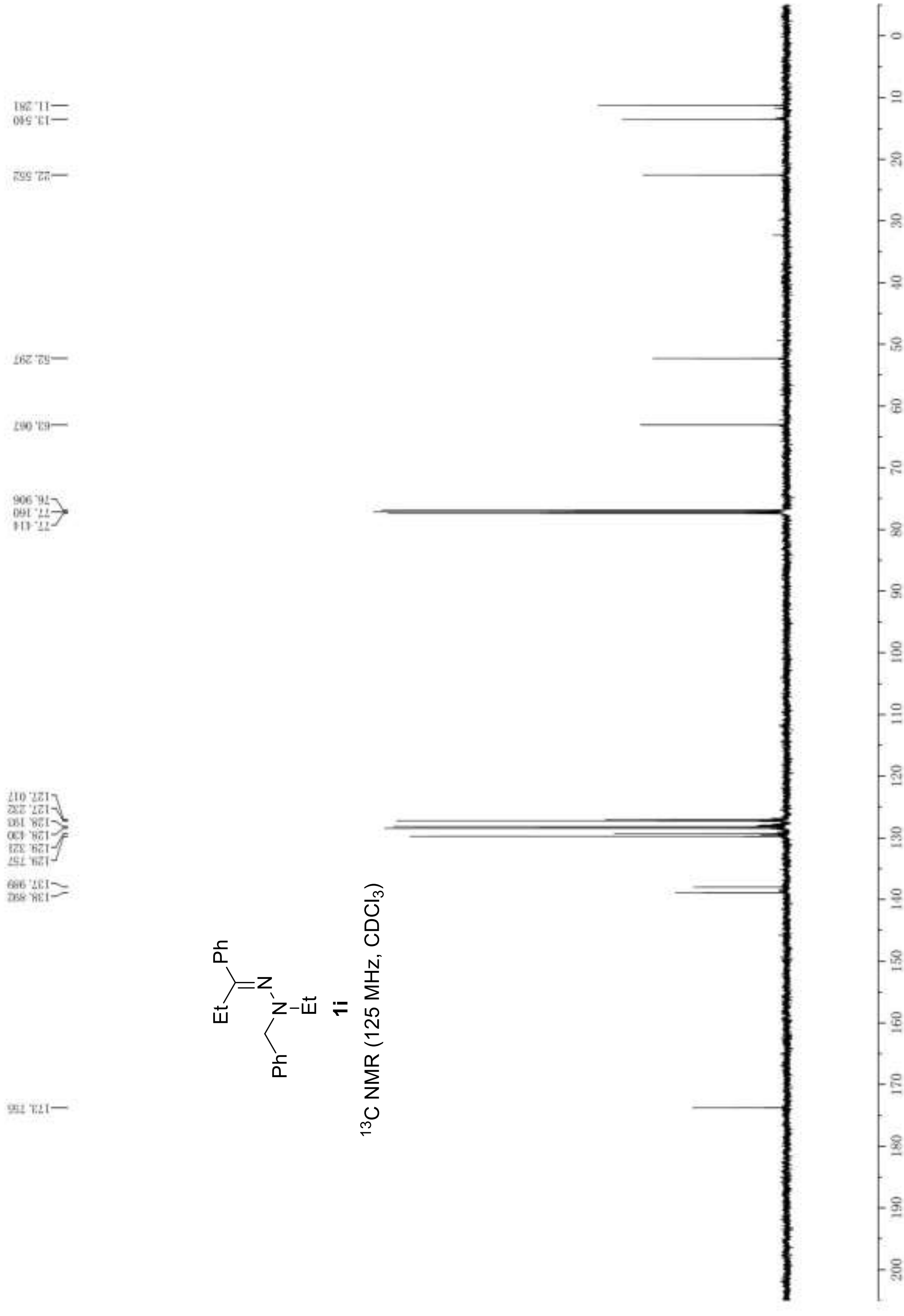

1i

^{13}C NMR (125 MHz, $CDCl_3$)

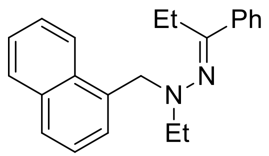

1j

^{1}H NMR (500 MHz, $CDCl_3$)

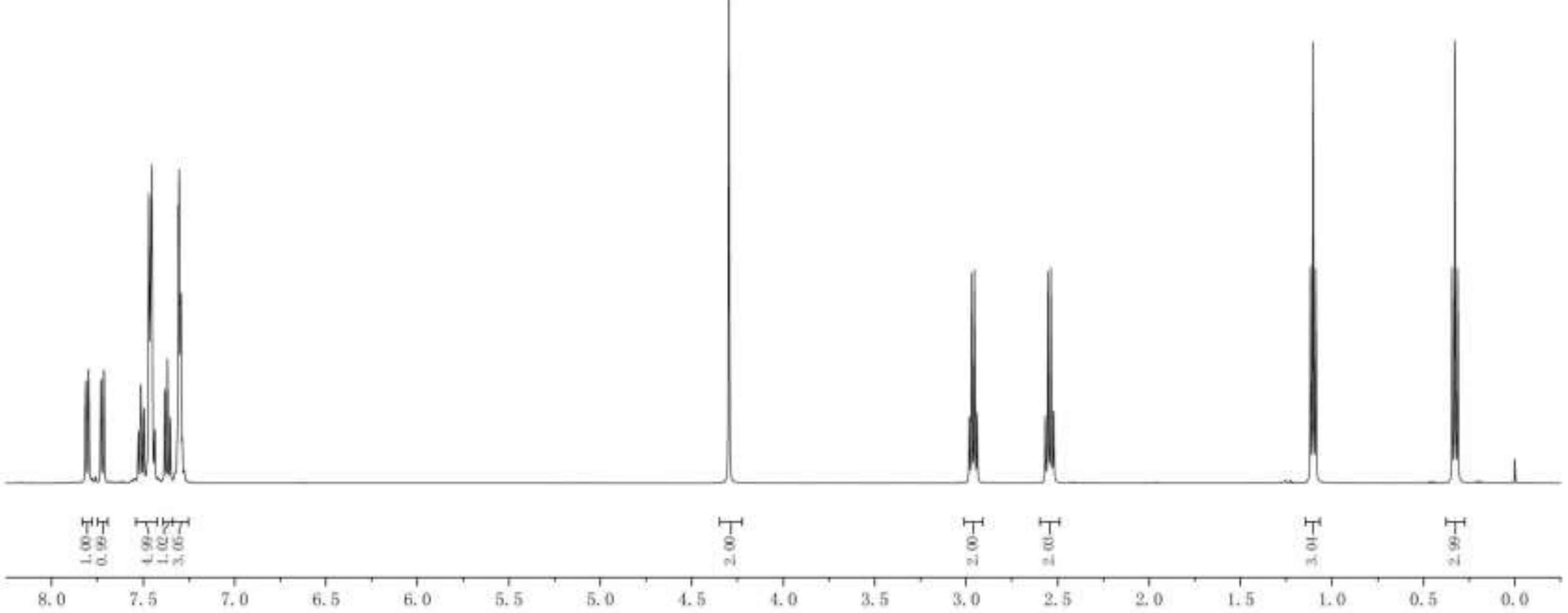

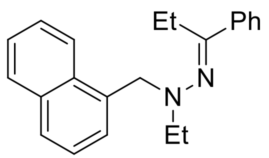

1j

^{13}C NMR (125 MHz, $CDCl_3$)

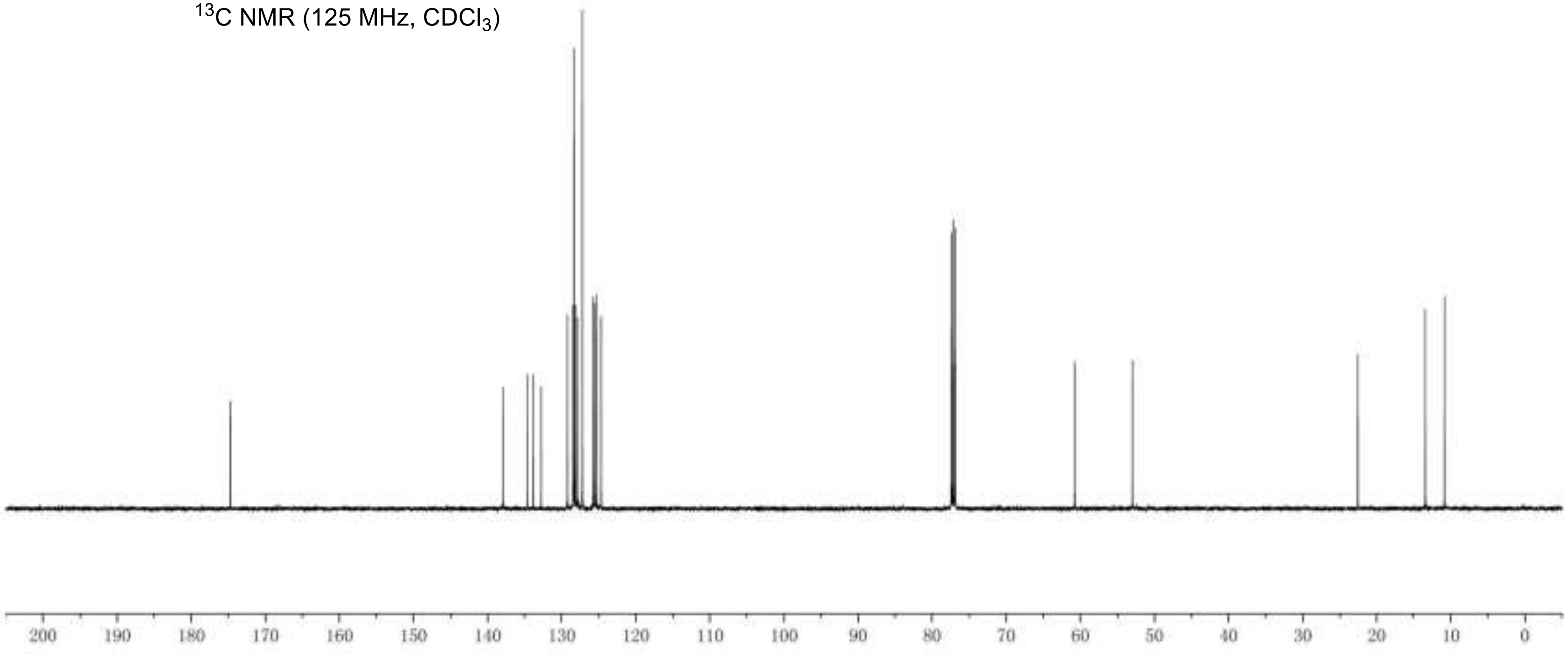

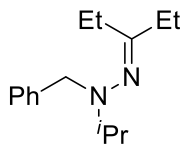

1l

^{1}H NMR (500 MHz, $CDCl_3$)

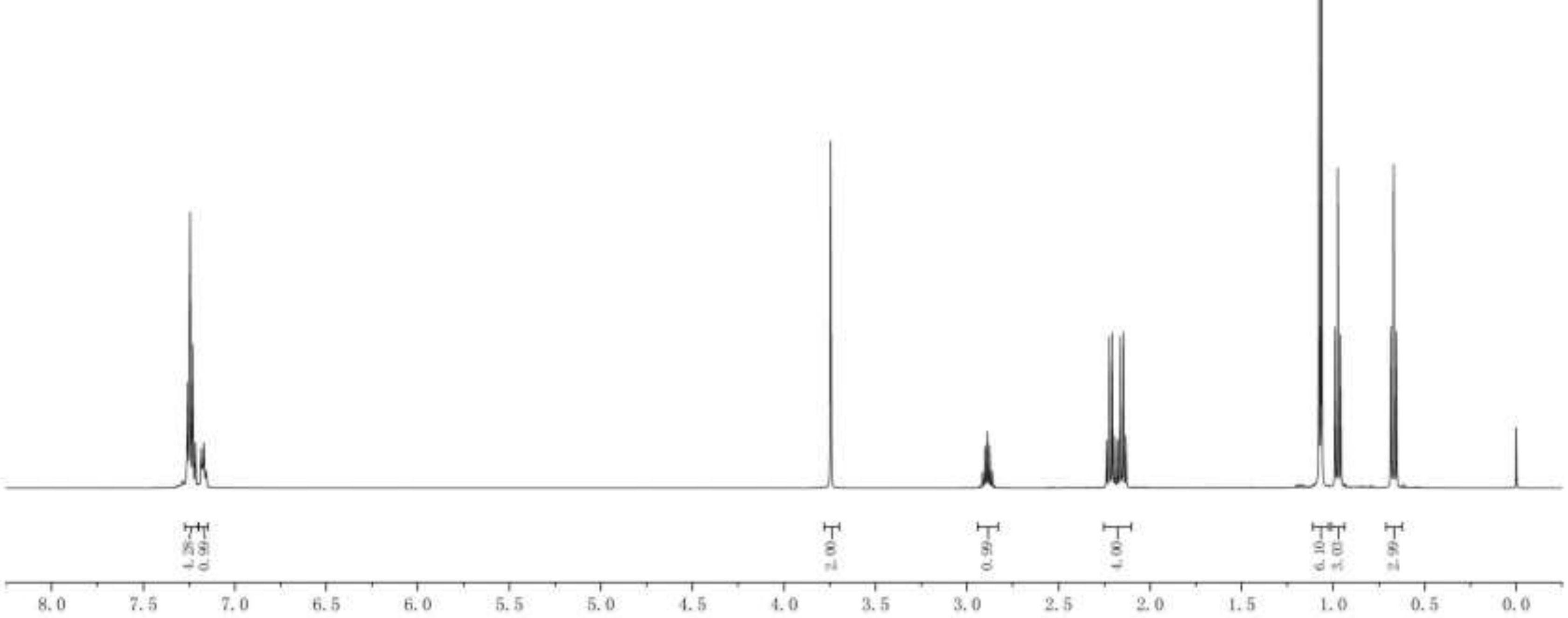

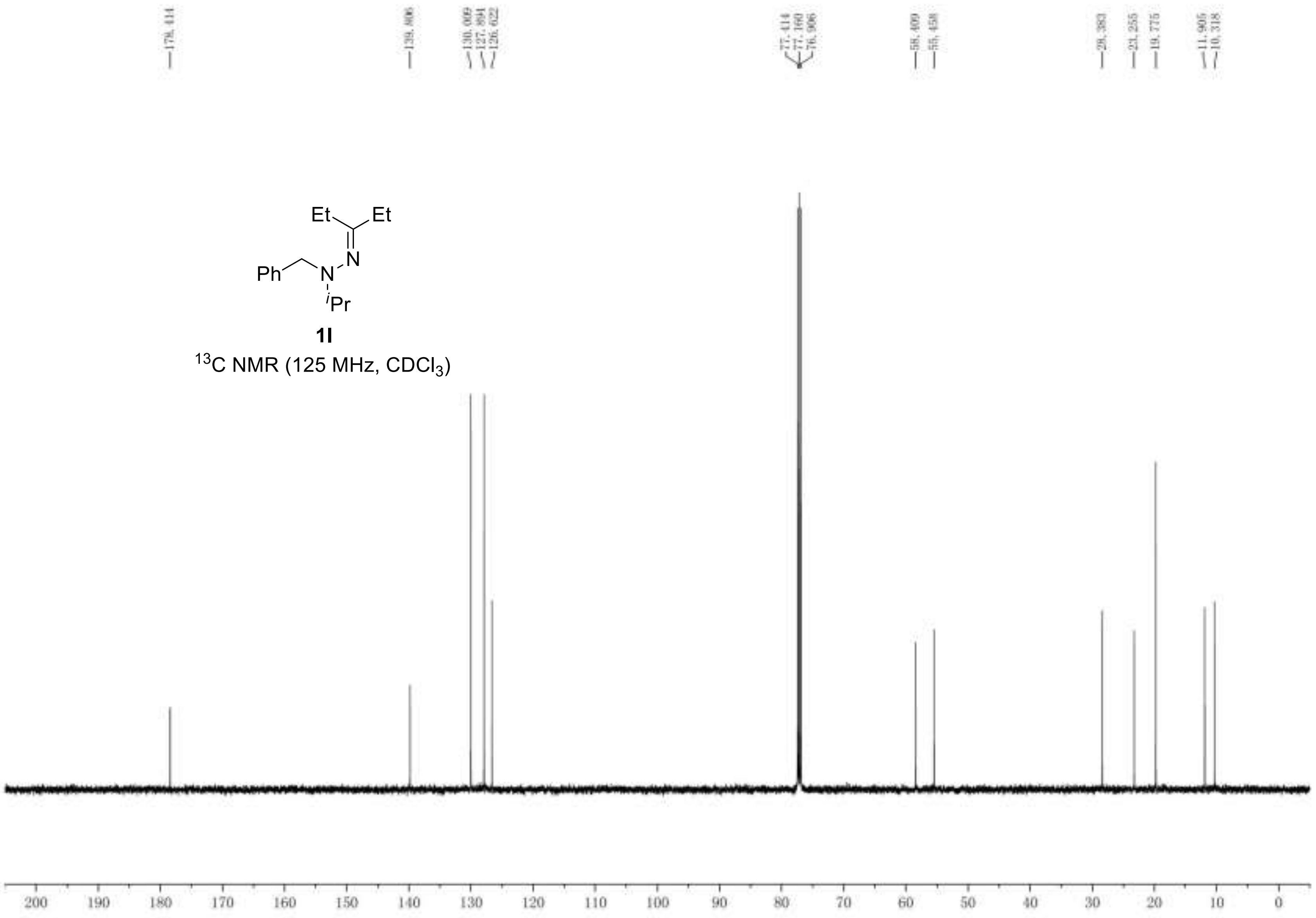

1l

^{13}C NMR (125 MHz, $CDCl_3$)

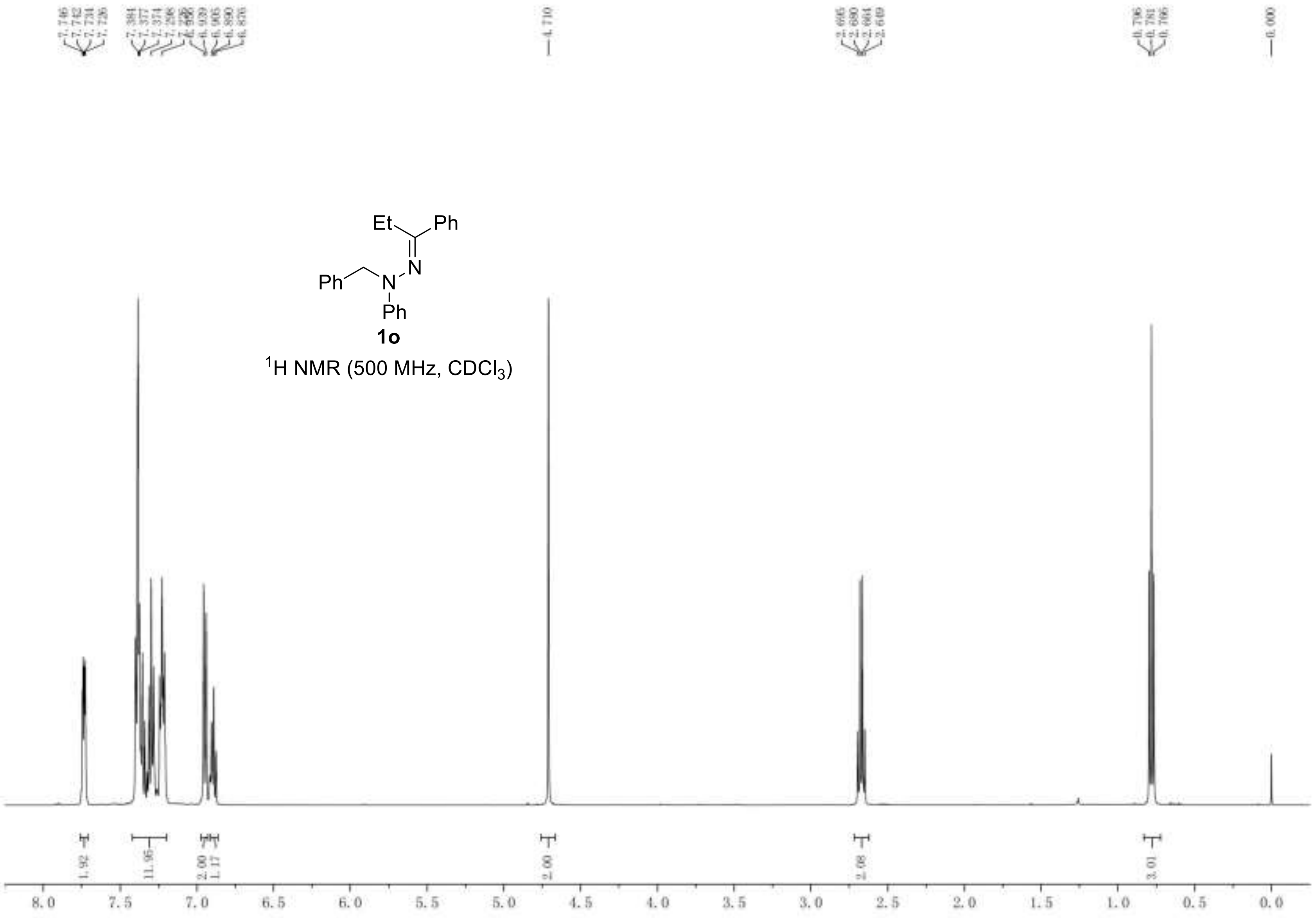

1o

^{1}H NMR (500 MHz, $CDCl_3$)

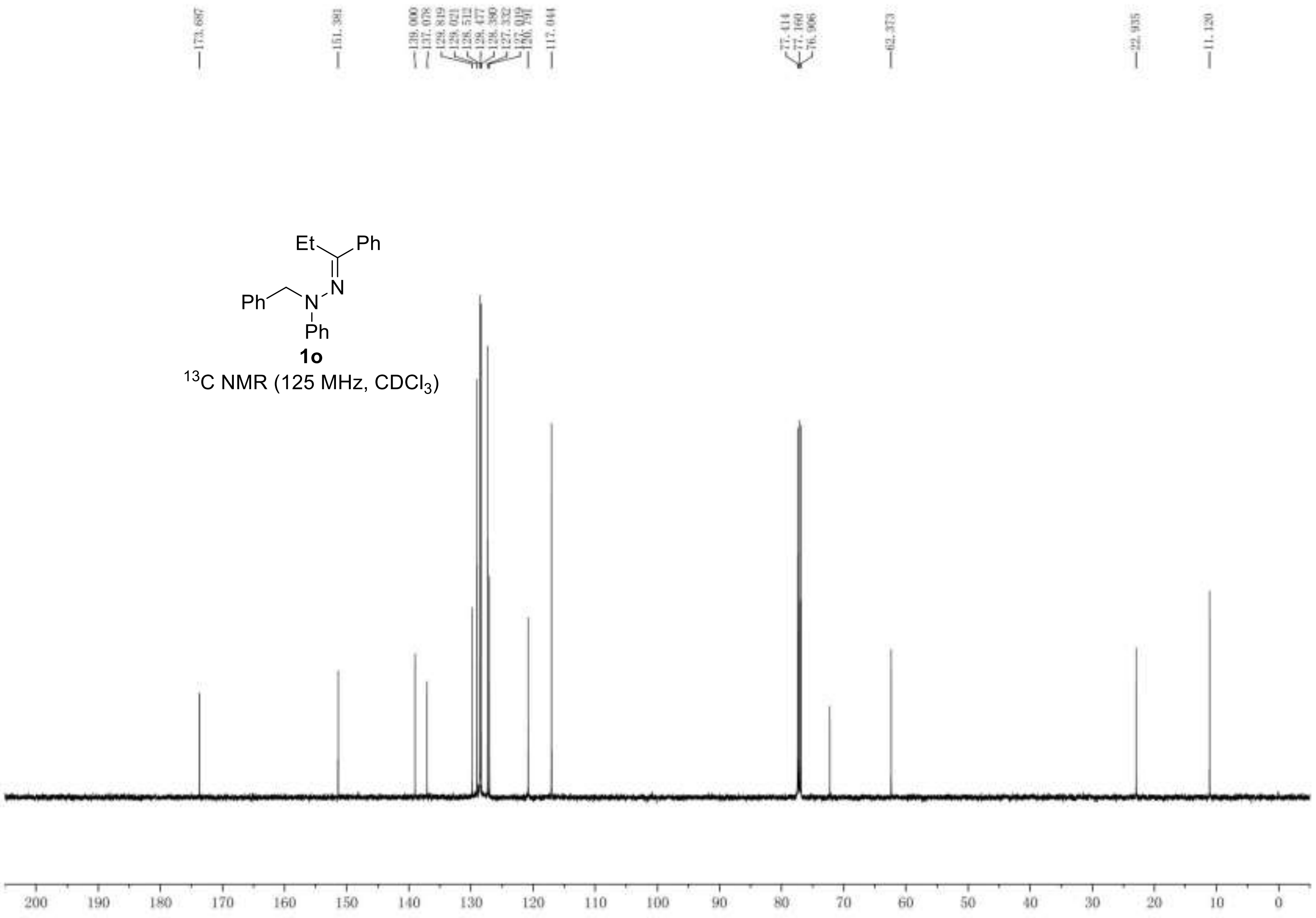

1o

^{13}C NMR (125 MHz, $CDCl_3$)

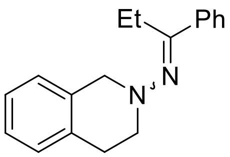

1p

^{1}H NMR (500 MHz, $CDCl_3$)

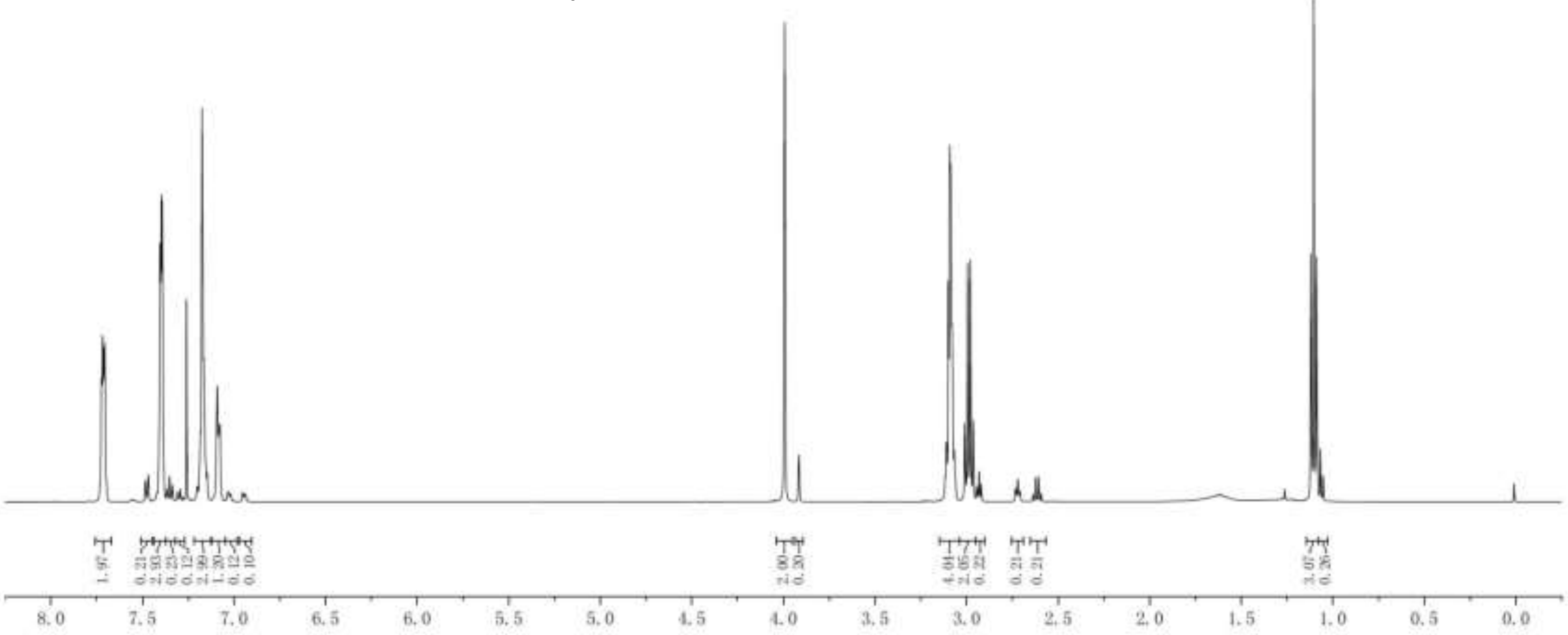

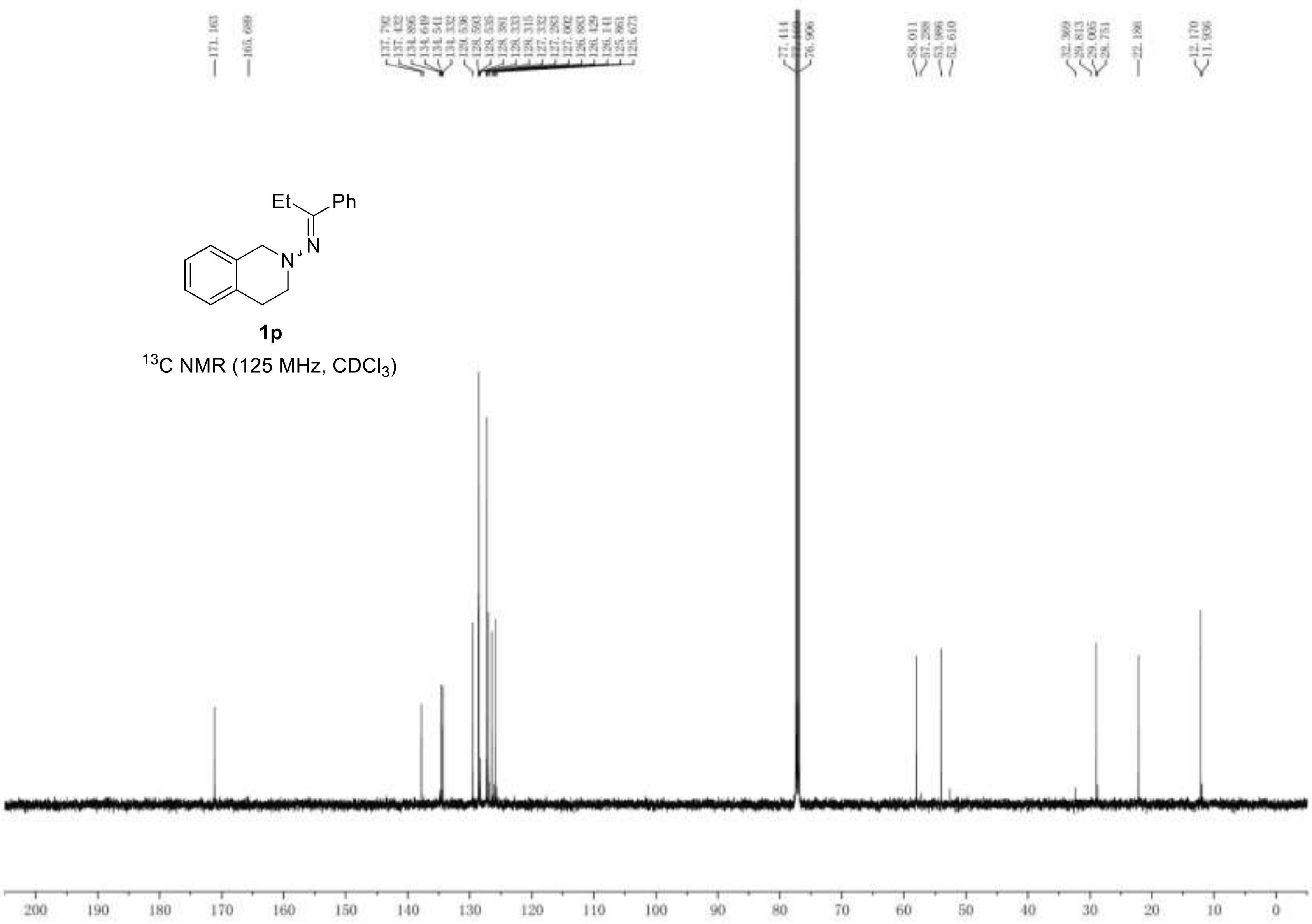

1p

^{13}C NMR (125 MHz, $CDCl_3$)

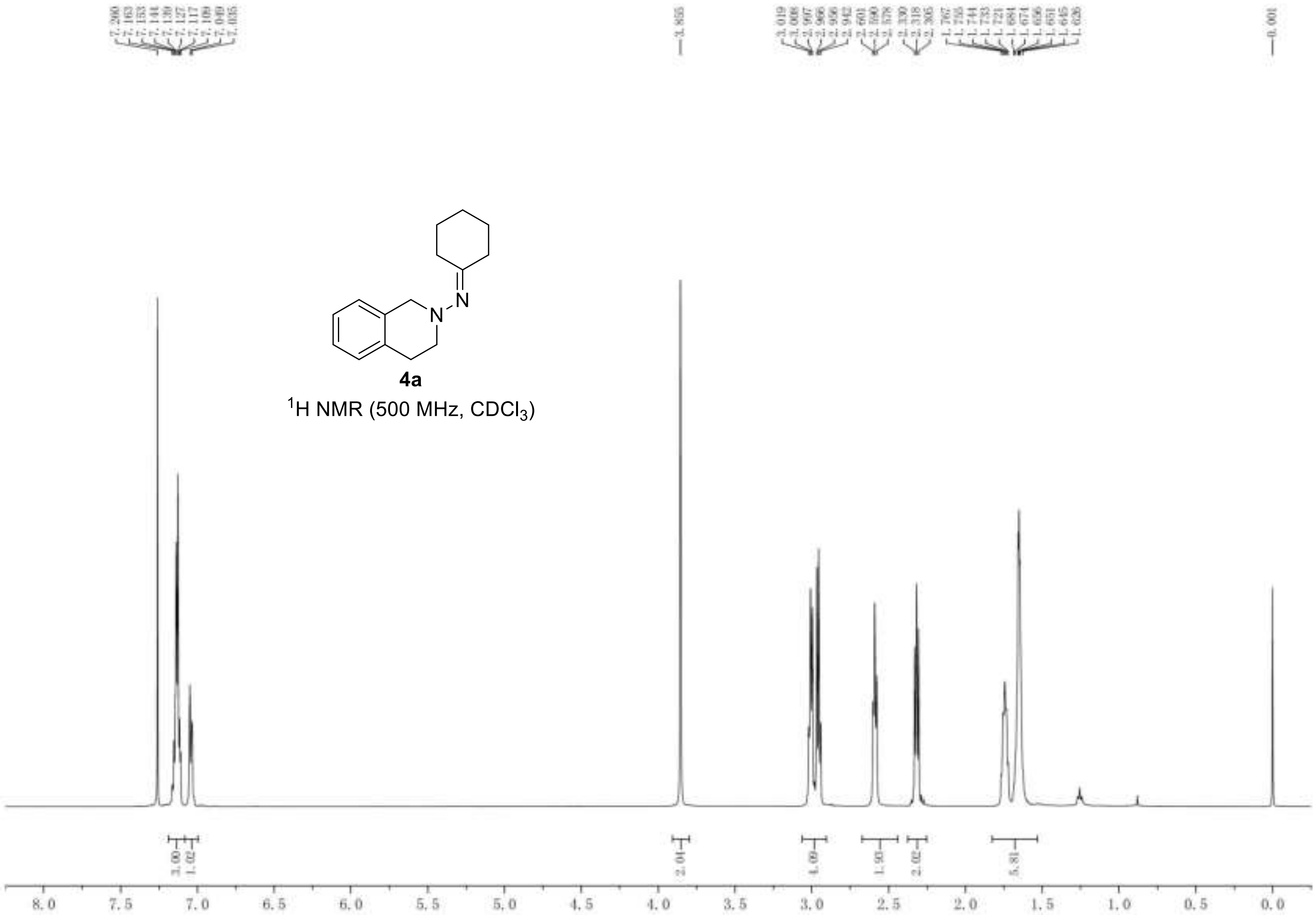

4a

^1H NMR (500 MHz, $CDCl_3$)

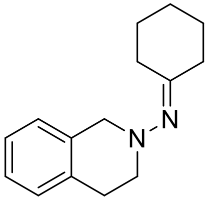

4a

^{13}C NMR (125 MHz, $CDCl_3$)

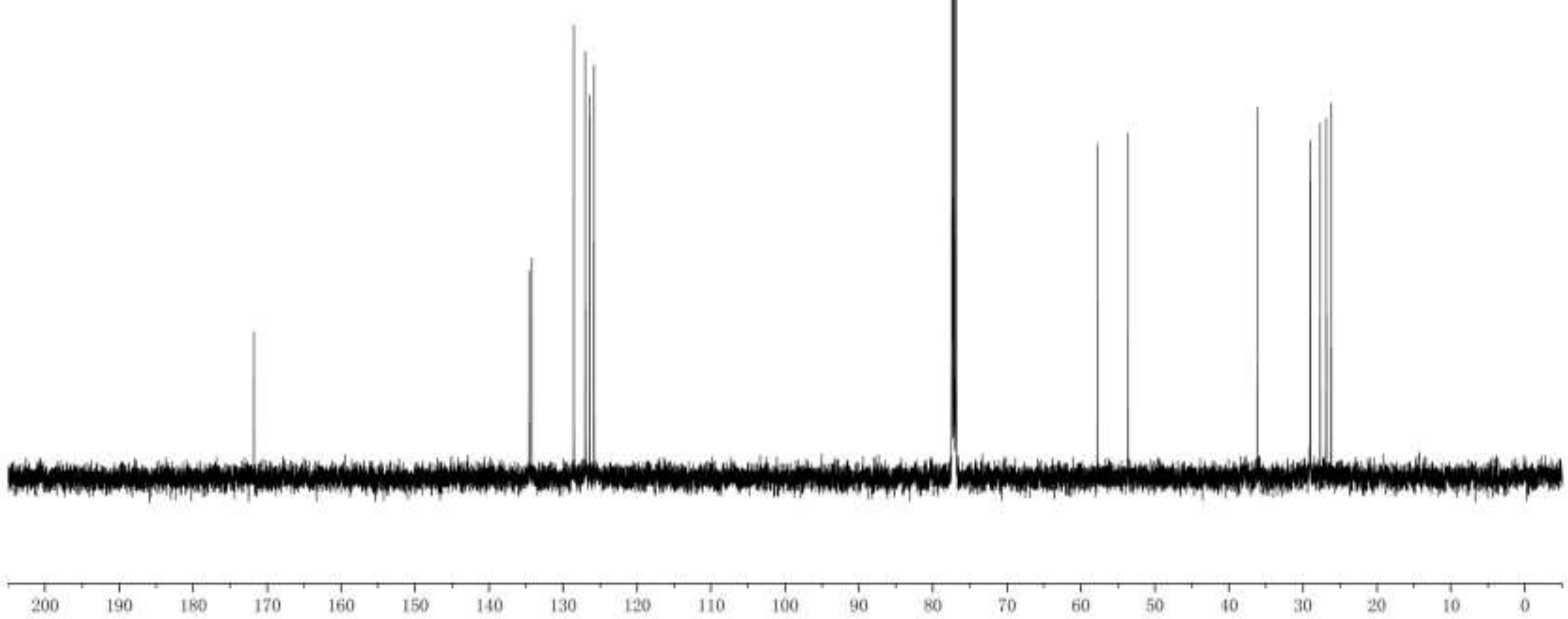

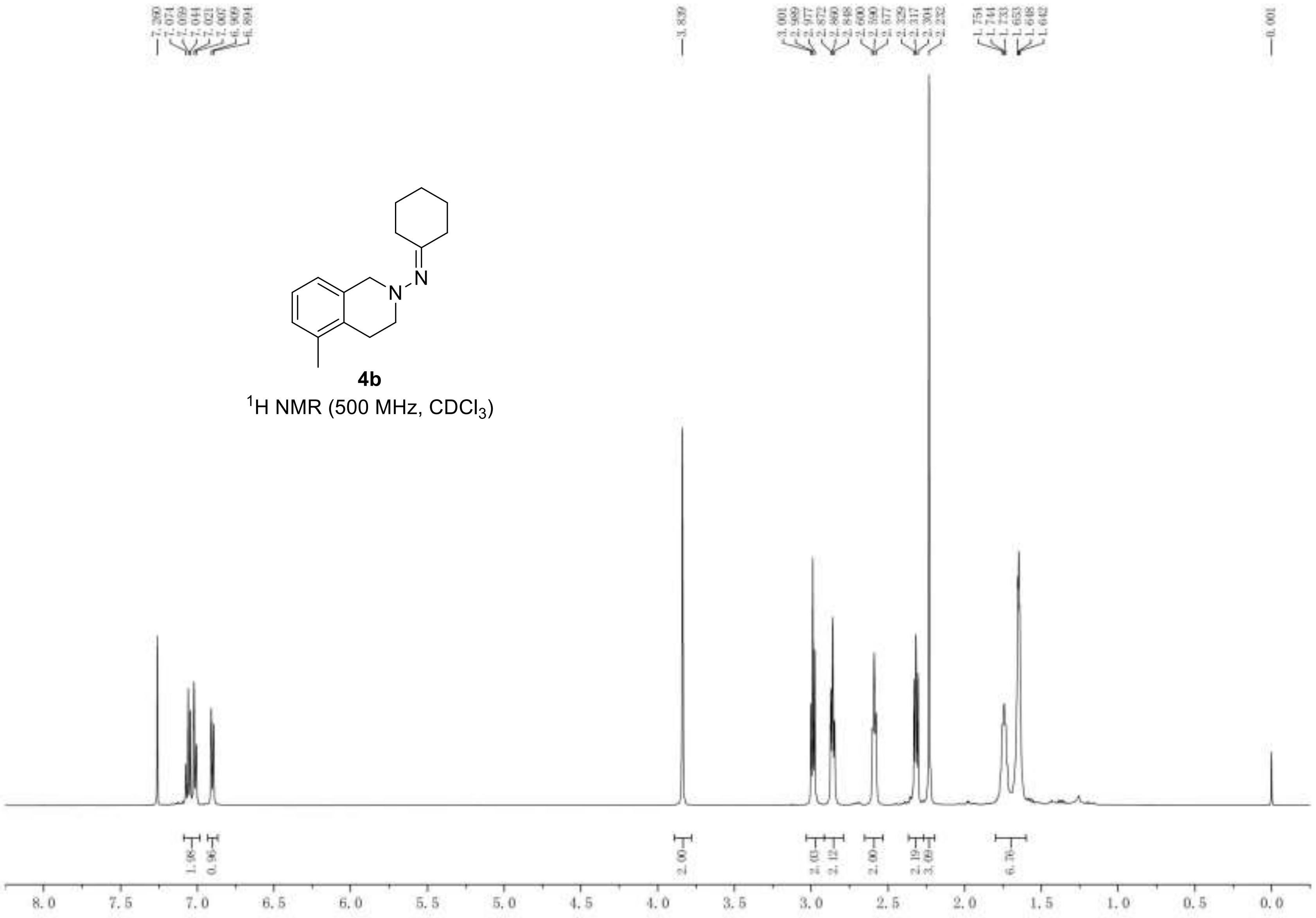

4b

^{1}H NMR (500 MHz, $CDCl_3$)

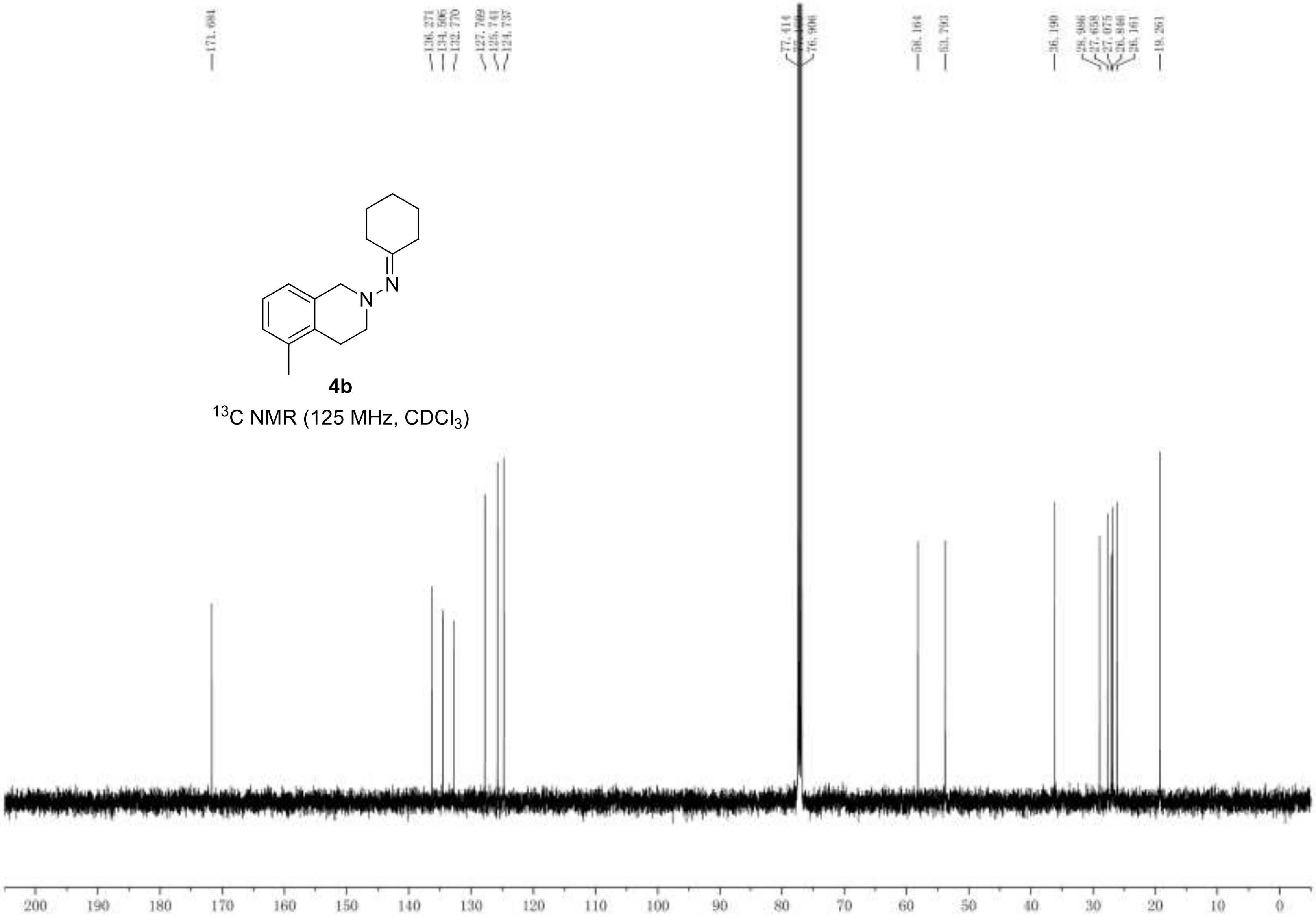

4b

^{13}C NMR (125 MHz, $CDCl_3$)

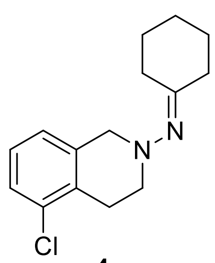

4c

^{1}H NMR (500 MHz, $CDCl_3$)

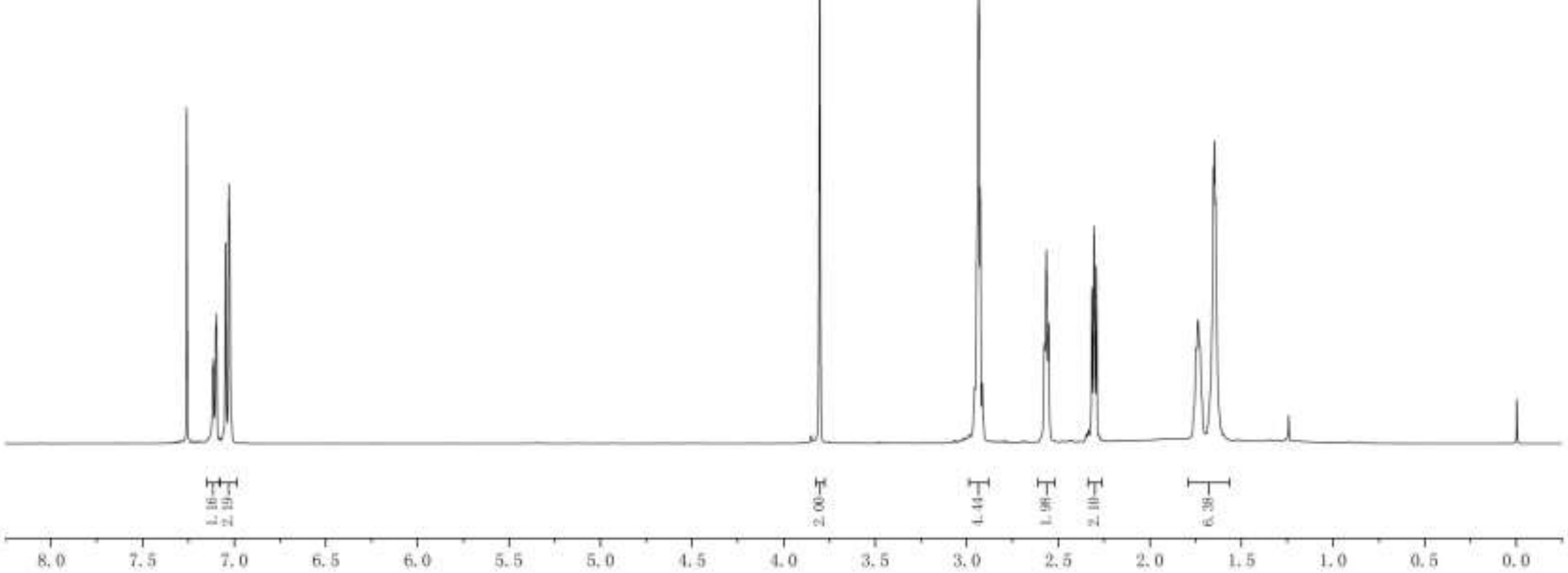

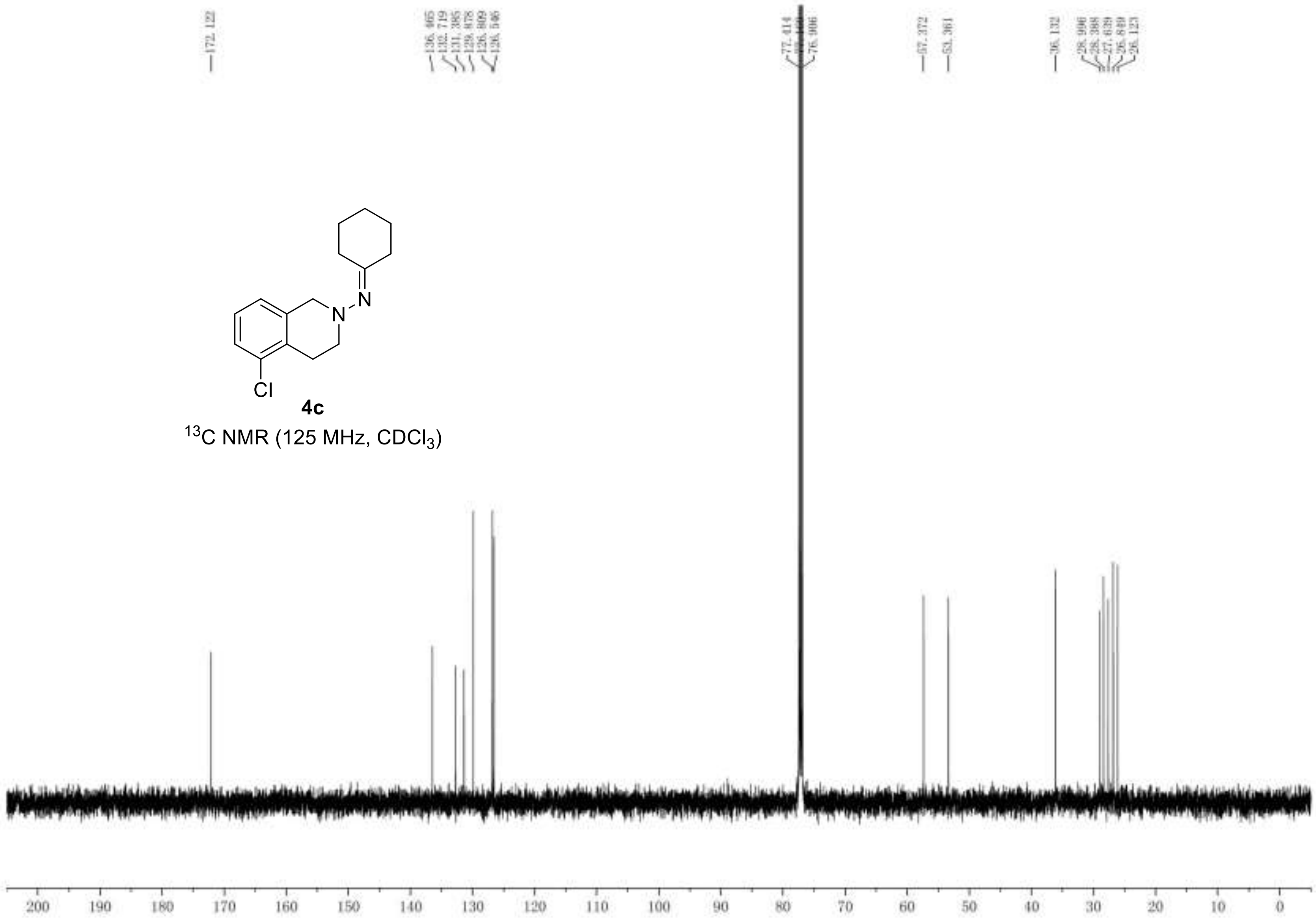

4c

^{13}C NMR (125 MHz, $CDCl_3$)

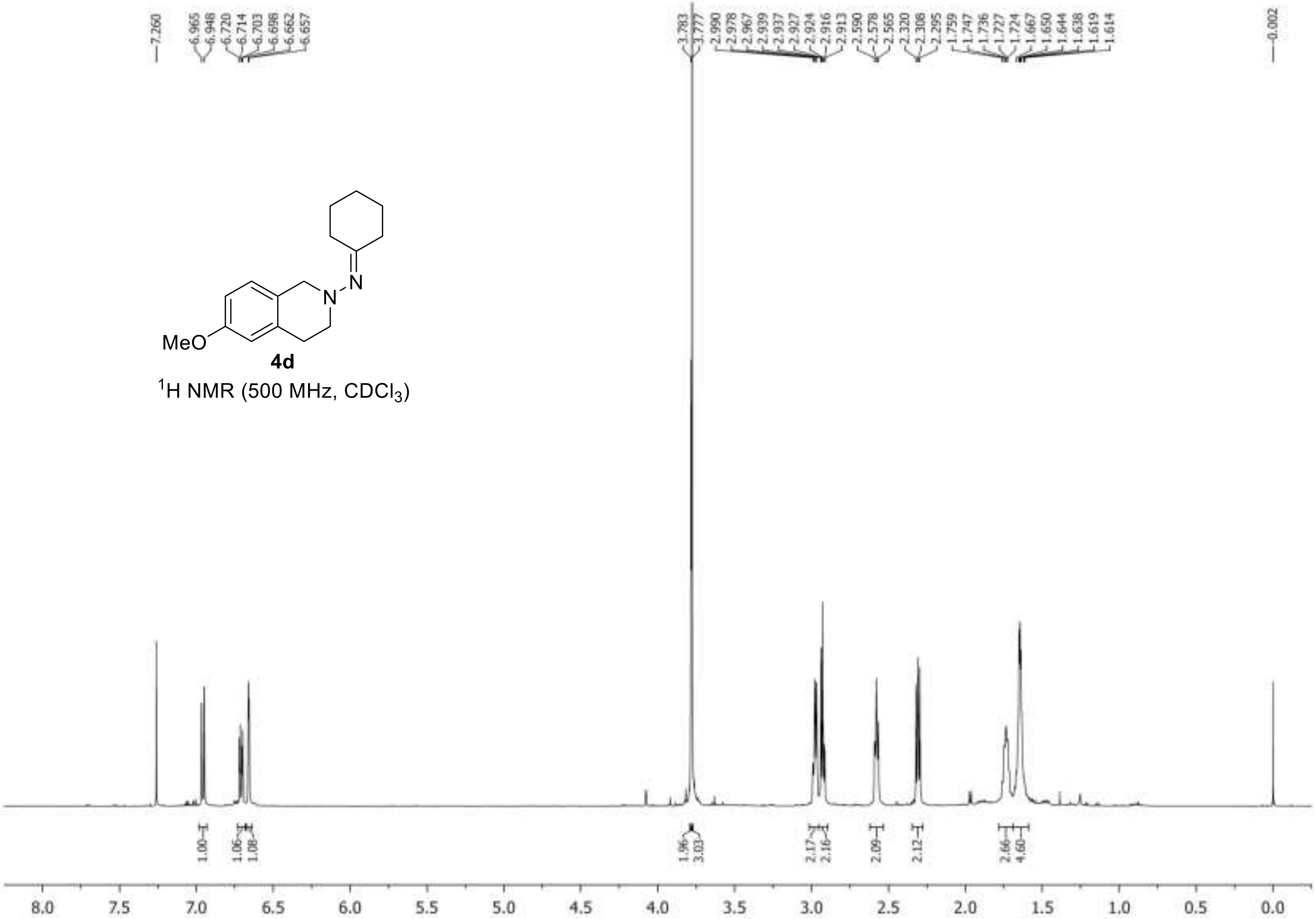

4d

^{1}H NMR (500 MHz, $CDCl_3$)

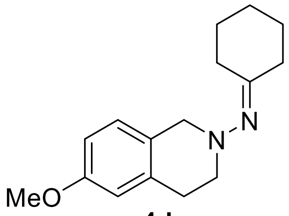

4d

^{13}C NMR (125 MHz, $CDCl_3$)

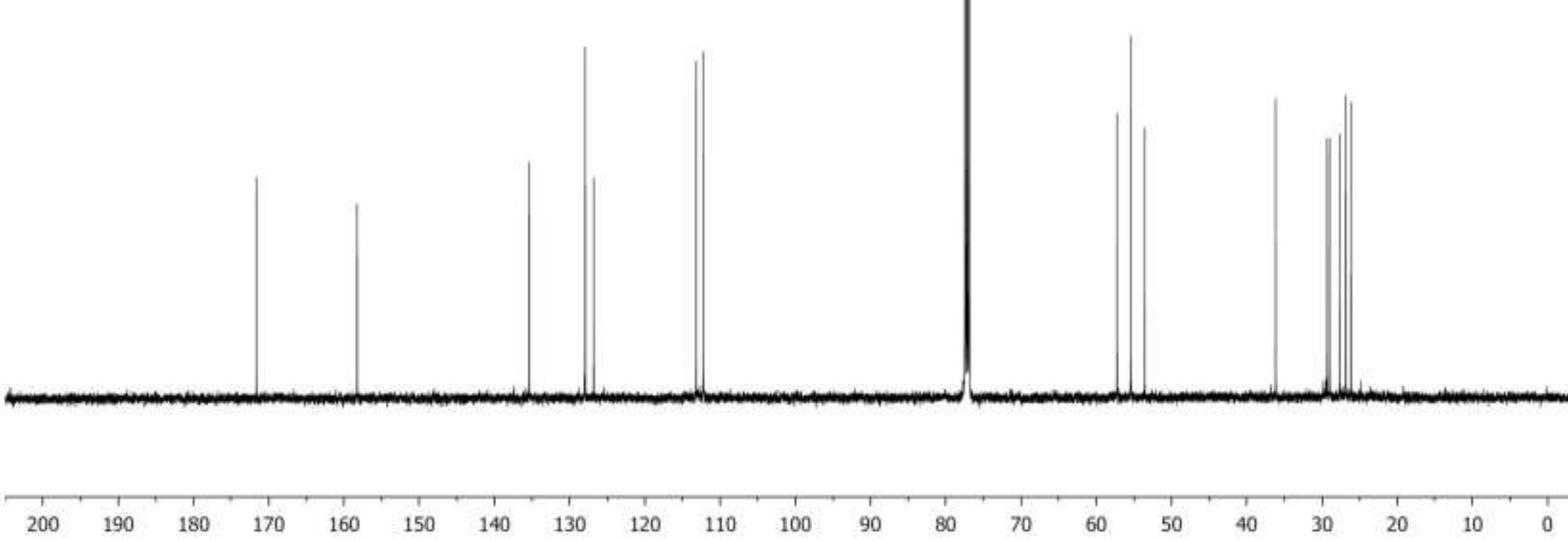

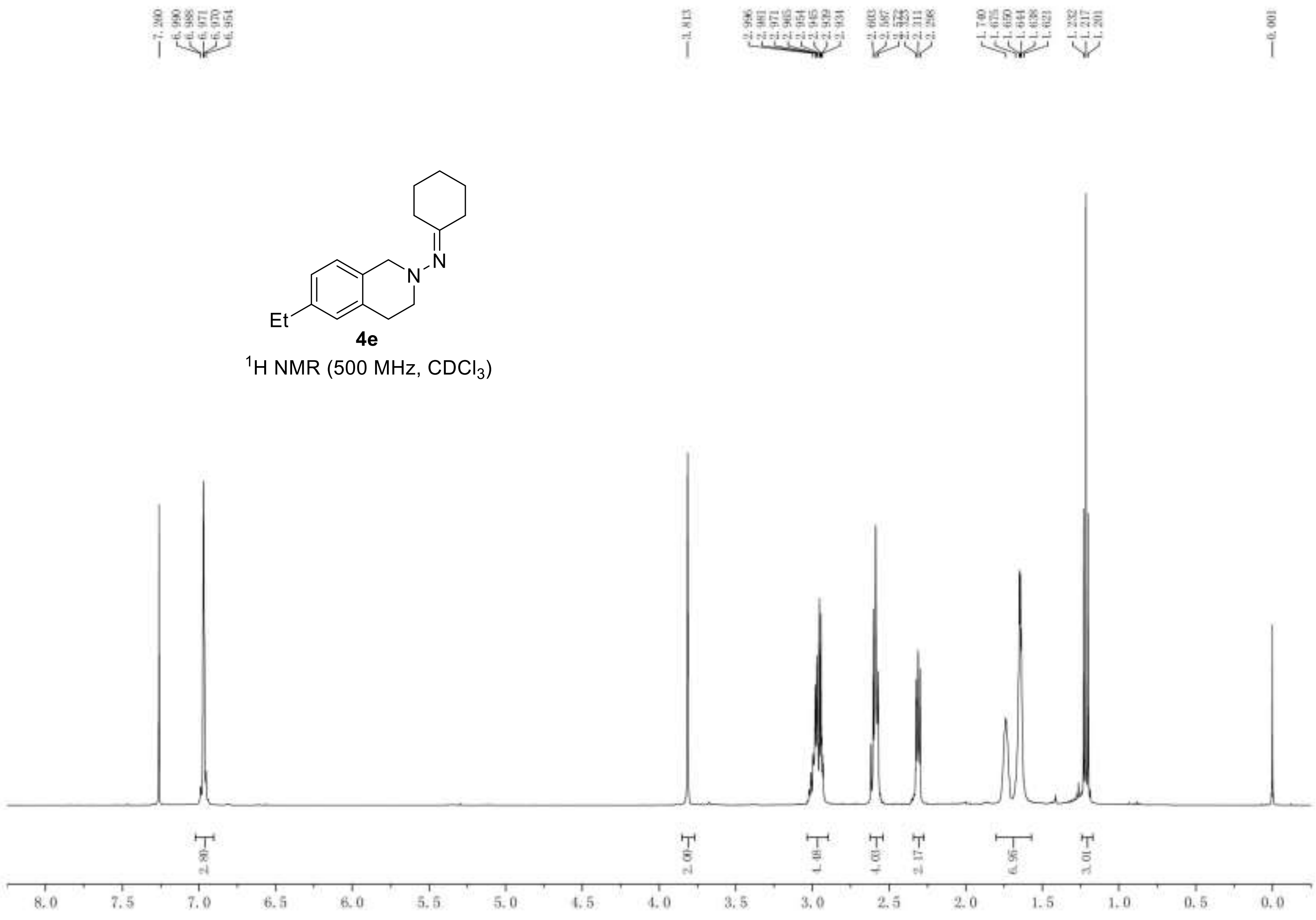

4e

^{1}H NMR (500 MHz, $CDCl_3$)

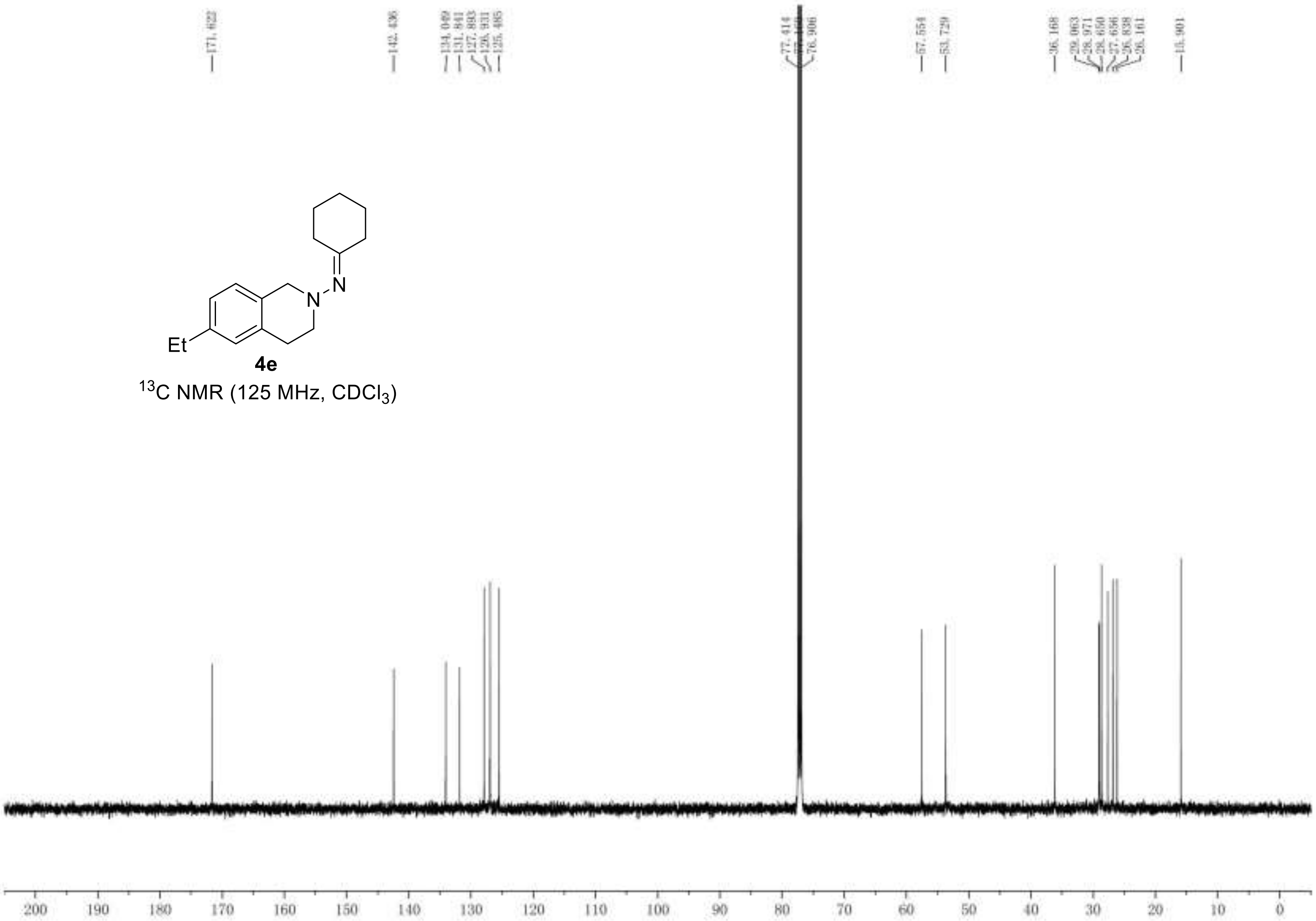

4e

^{13}C NMR (125 MHz, $CDCl_3$)

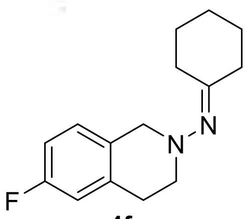

4f

^{1}H NMR (500 MHz, $CDCl_3$)

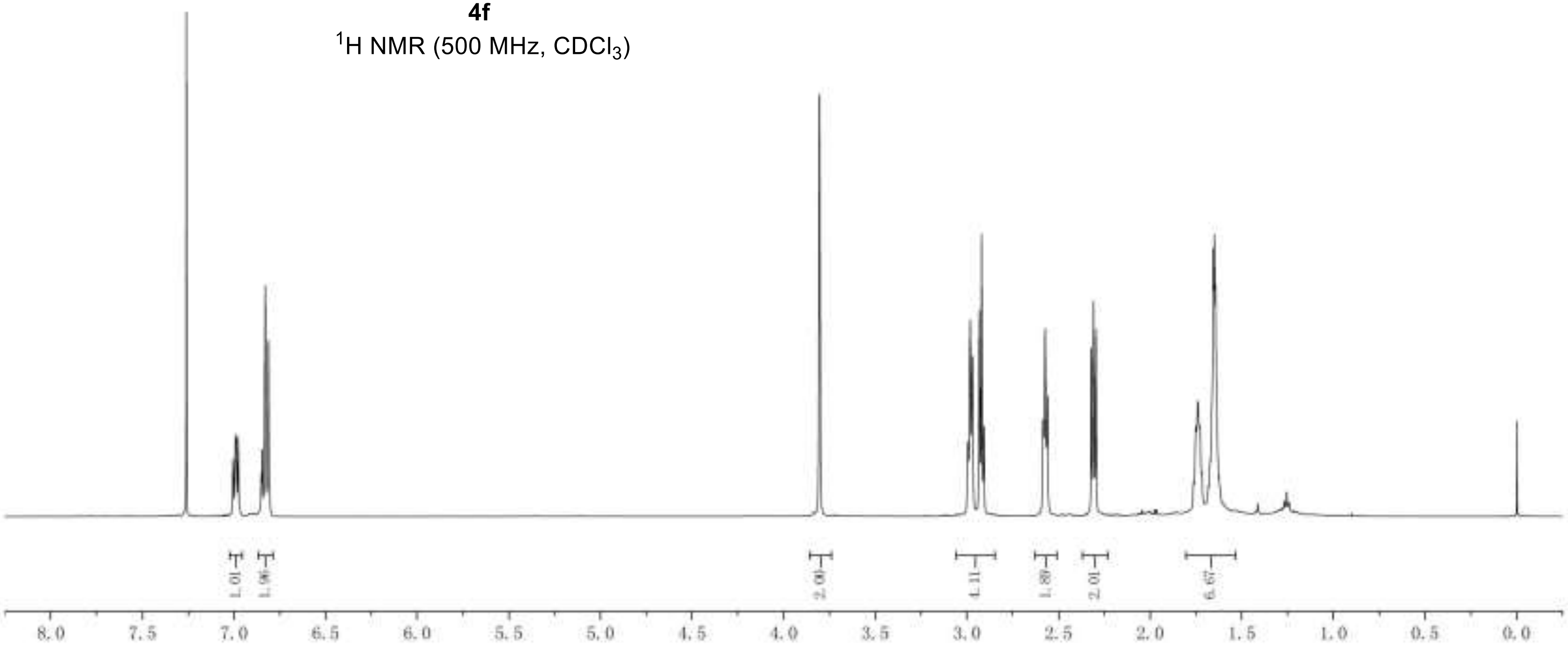

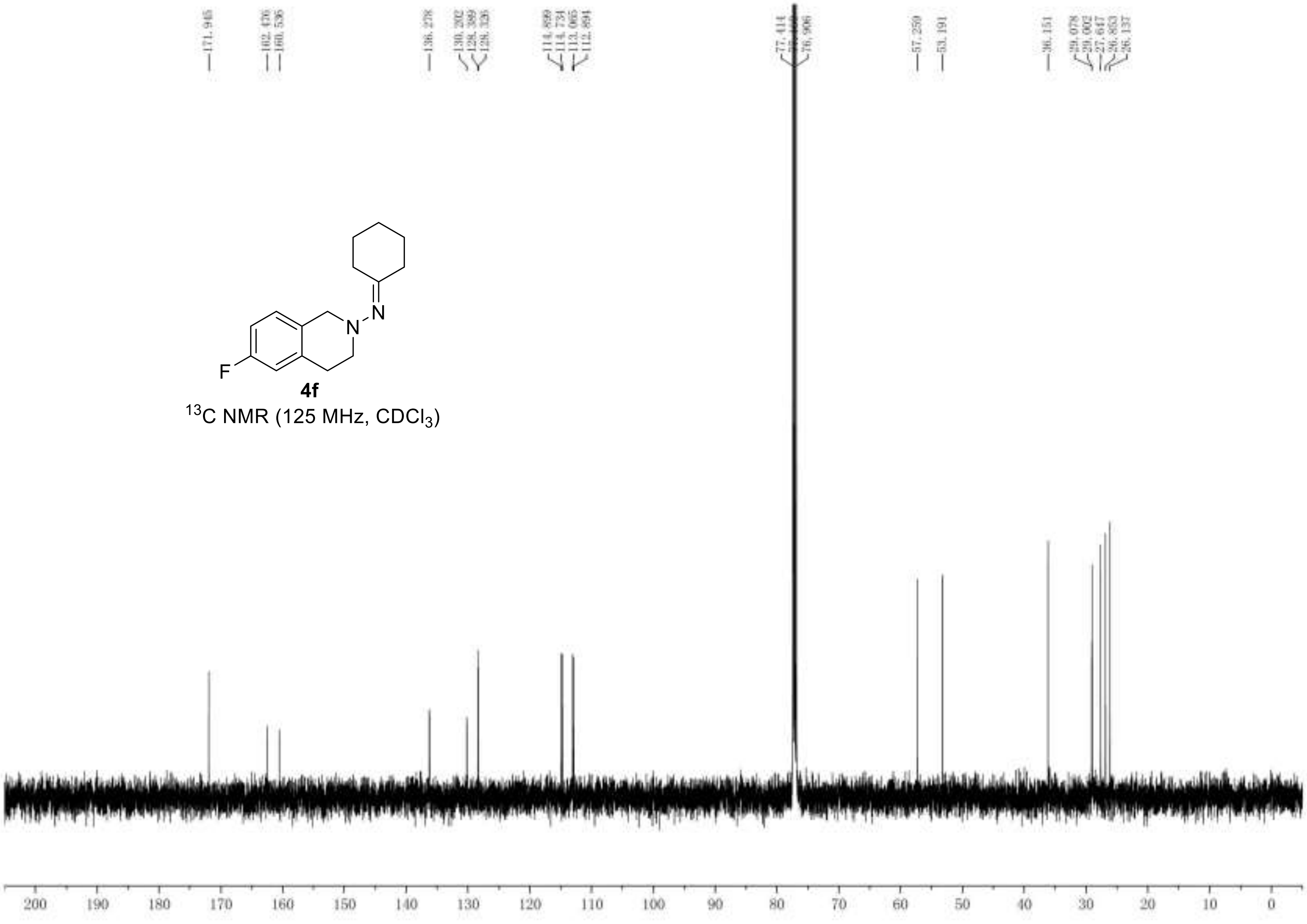

4f

^{13}C NMR (125 MHz, $CDCl_3$)

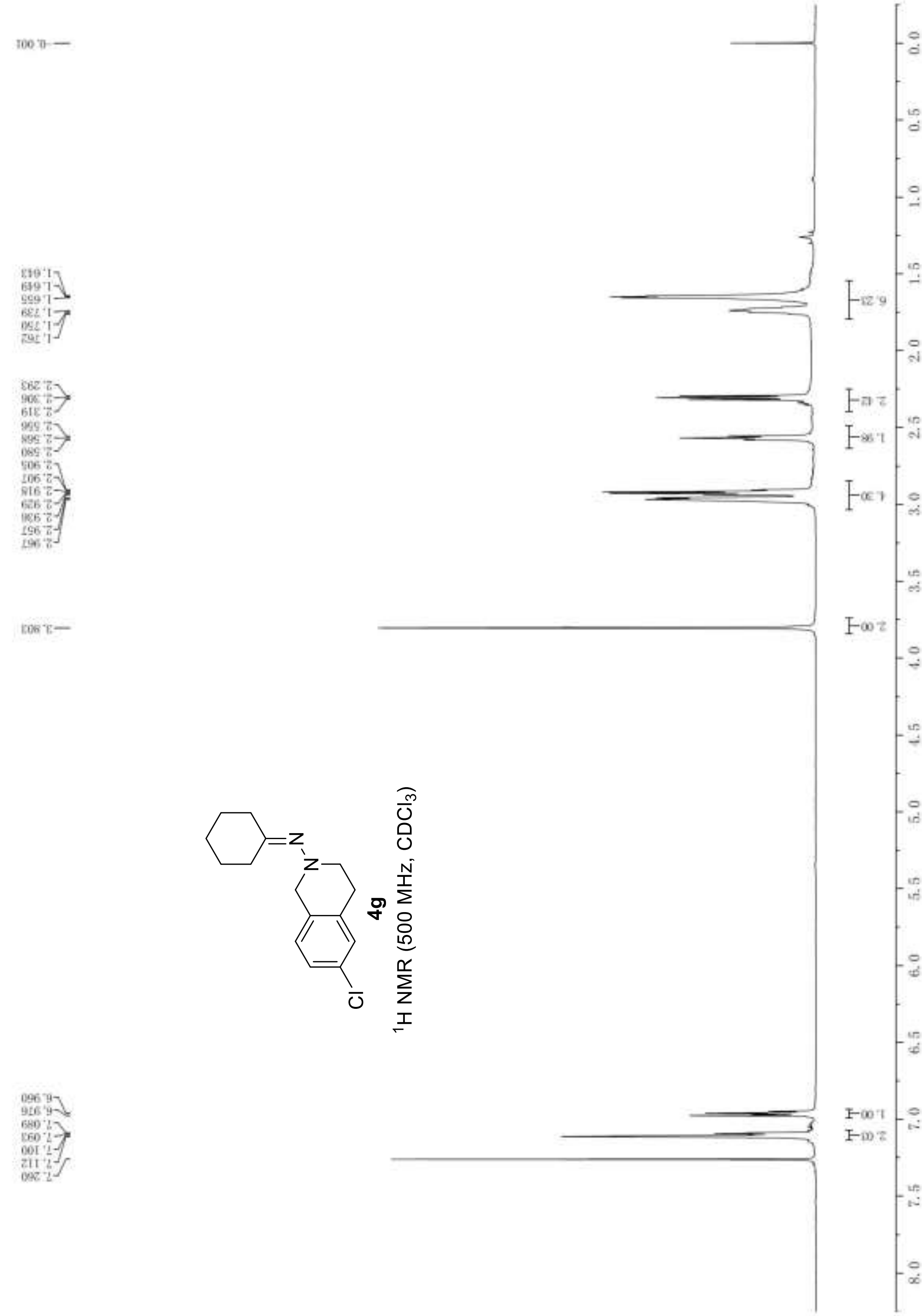

4g

^{1}H NMR (500 MHz, $CDCl_3$)

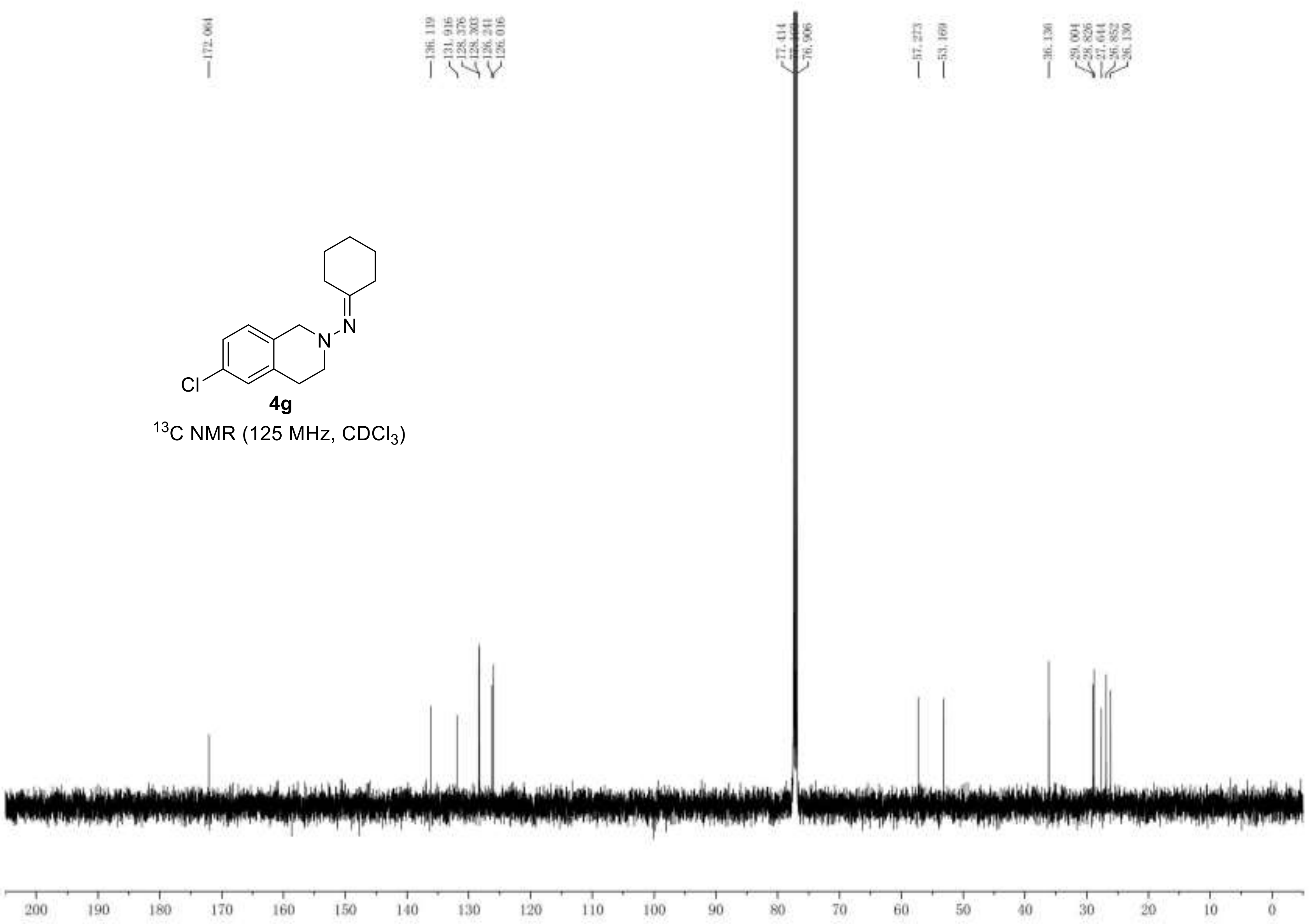

4g

^{13}C NMR (125 MHz, $CDCl_3$)

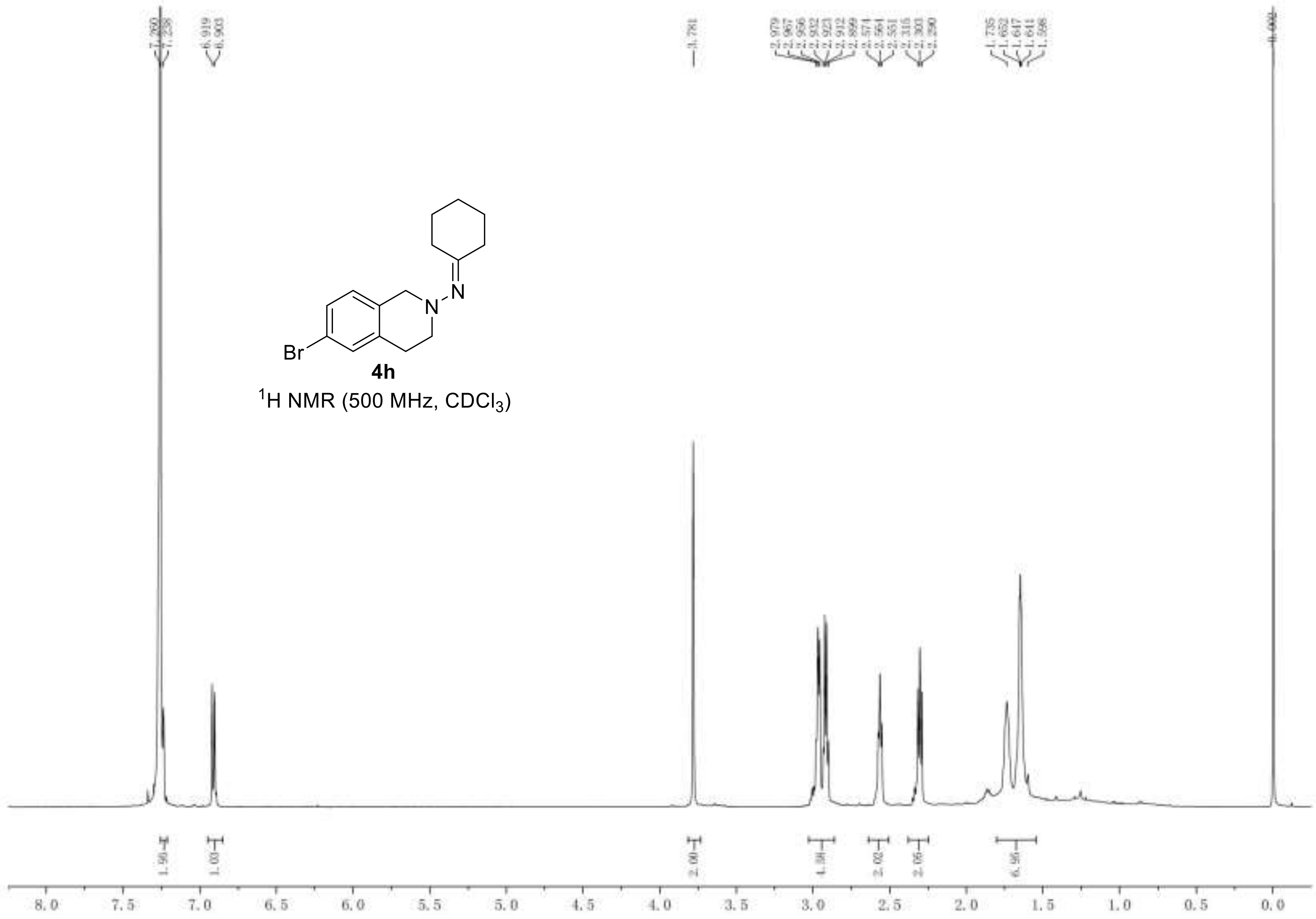

4h

^{1}H NMR (500 MHz, $CDCl_3$)

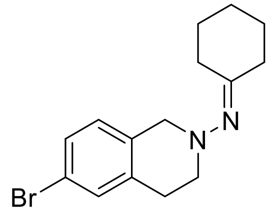

4h

^{13}C NMR (125 MHz, $CDCl_3$)

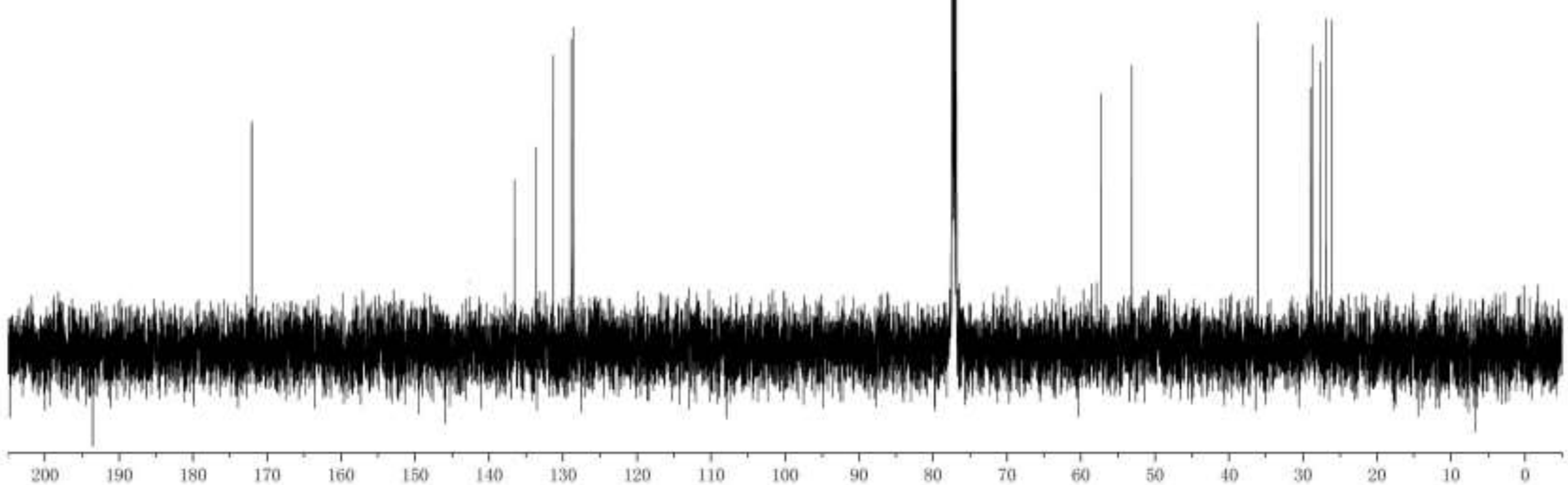

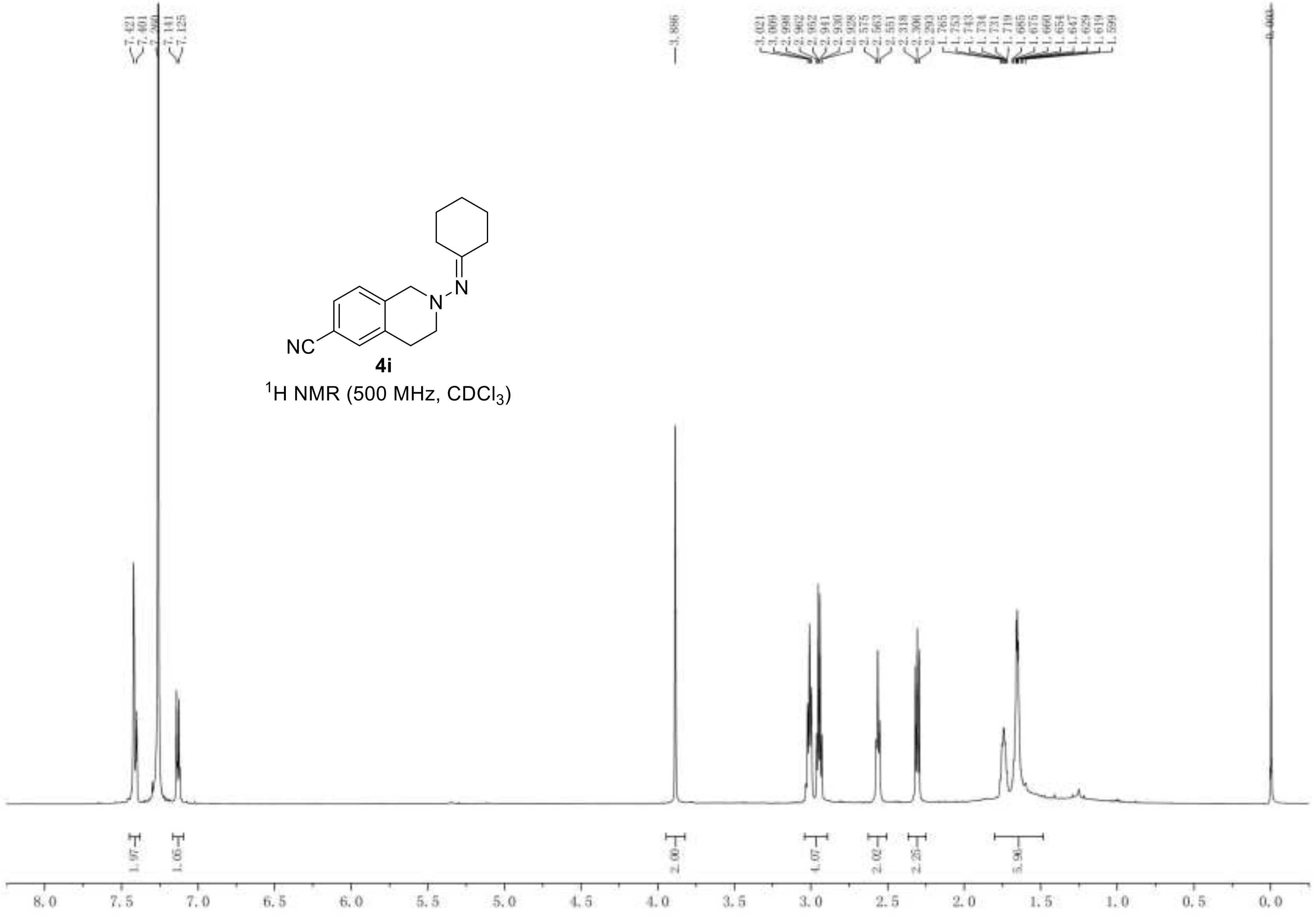

4i

^{1}H NMR (500 MHz, $CDCl_3$)

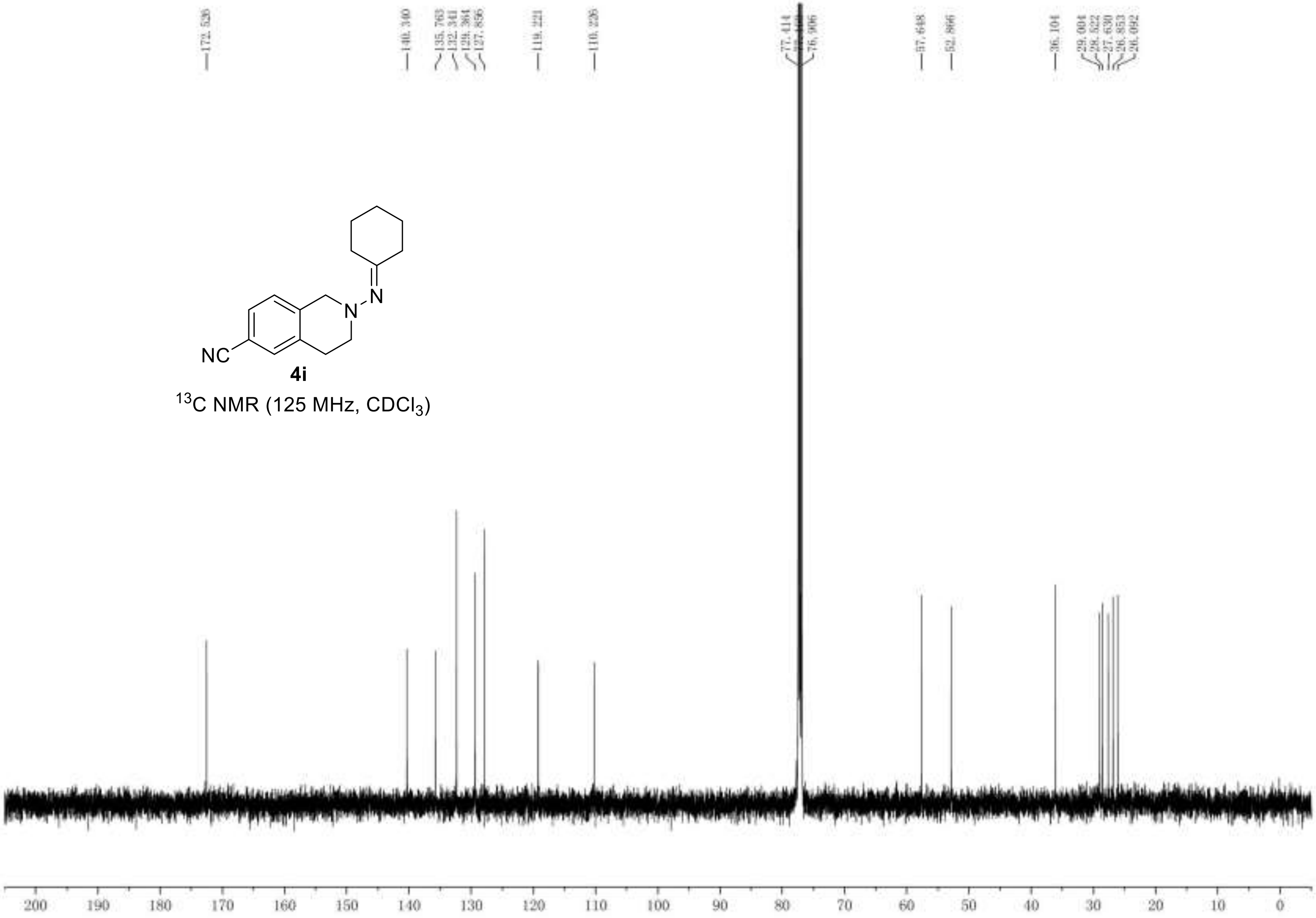

4i

^{13}C NMR (125 MHz, $CDCl_3$)

4j

^{1}H NMR (500 MHz, $CDCl_3$)

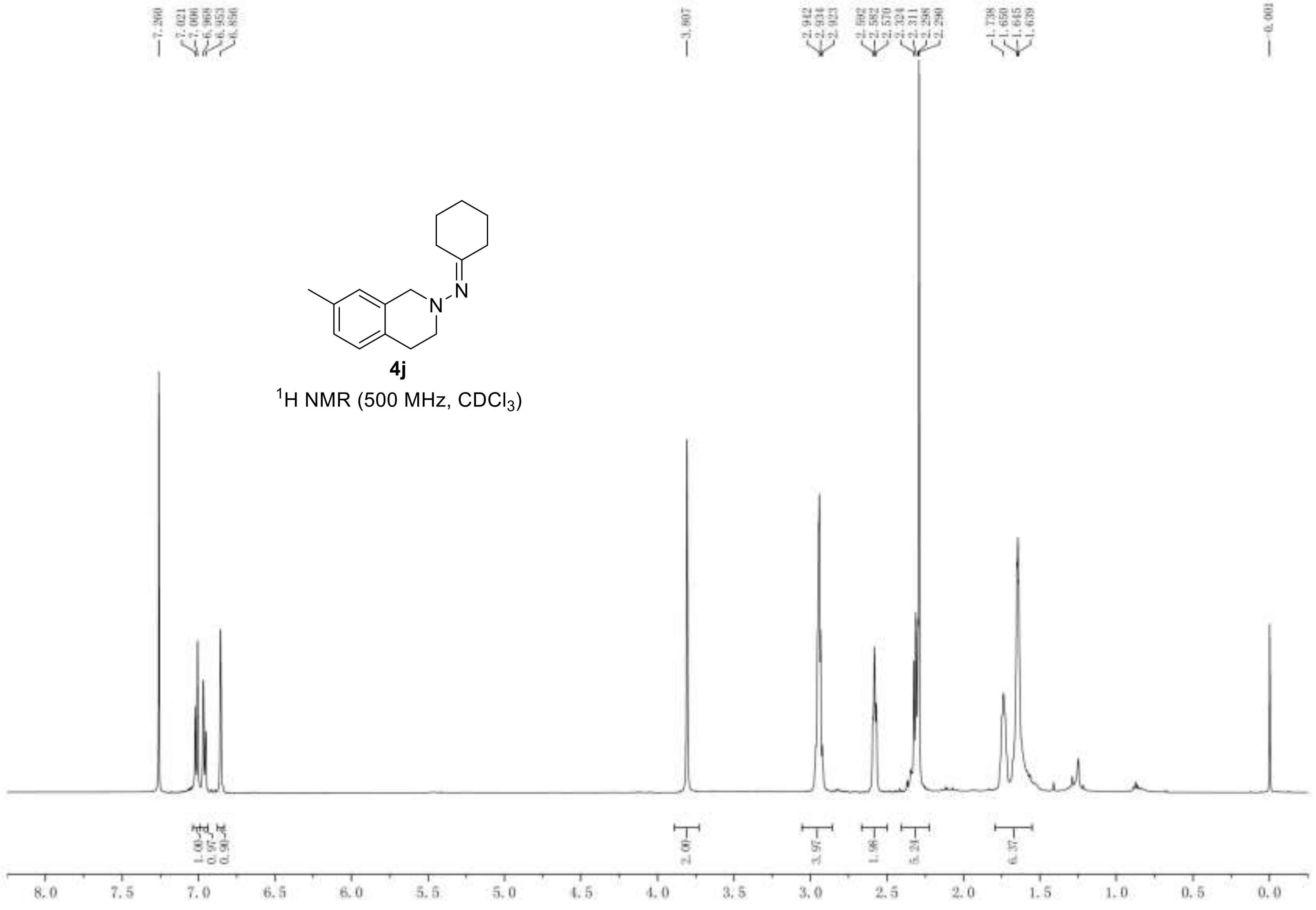

4j

^{13}C NMR (125 MHz, $CDCl_3$)

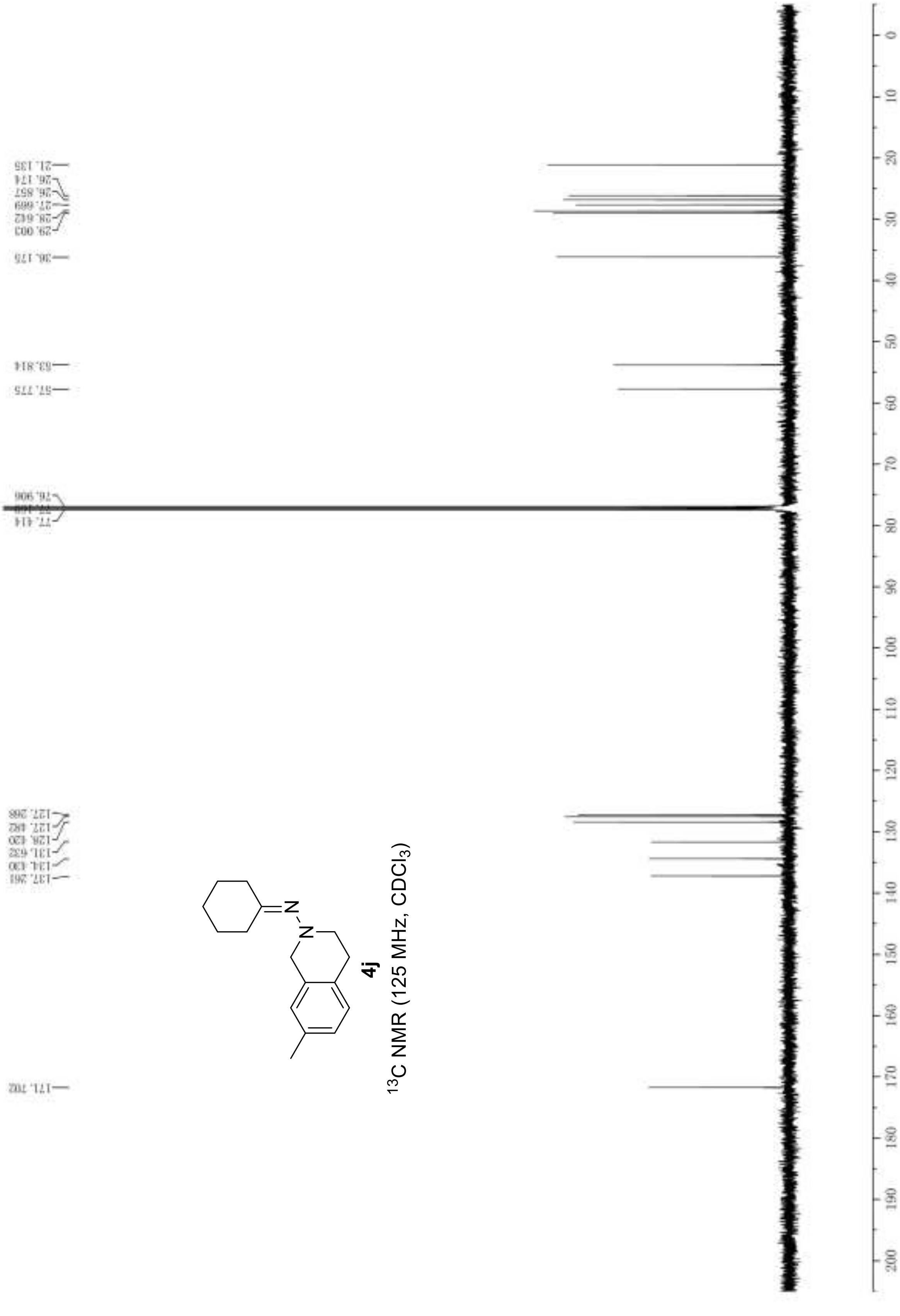

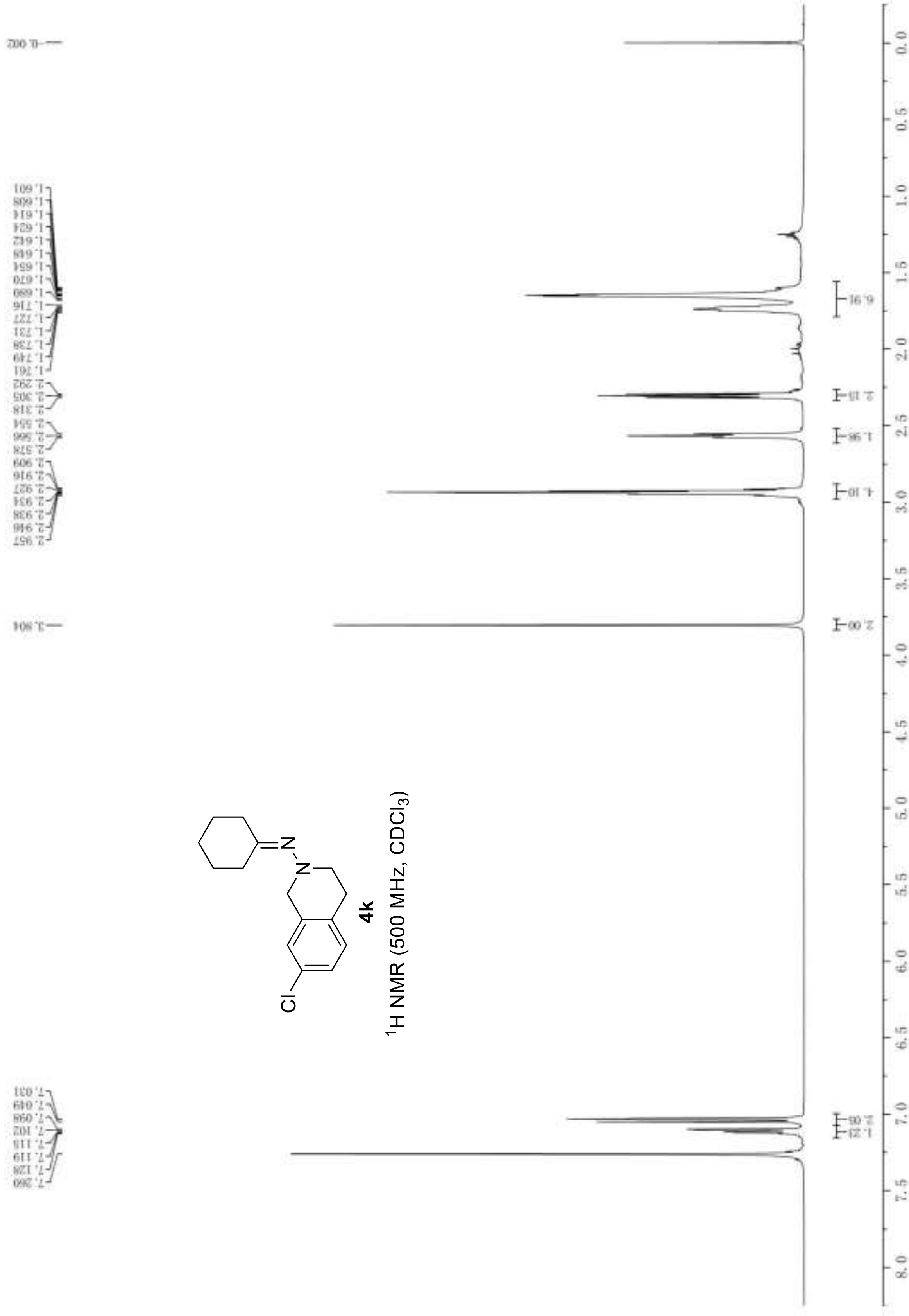

4k

^{1}H NMR (500 MHz, $CDCl_3$)

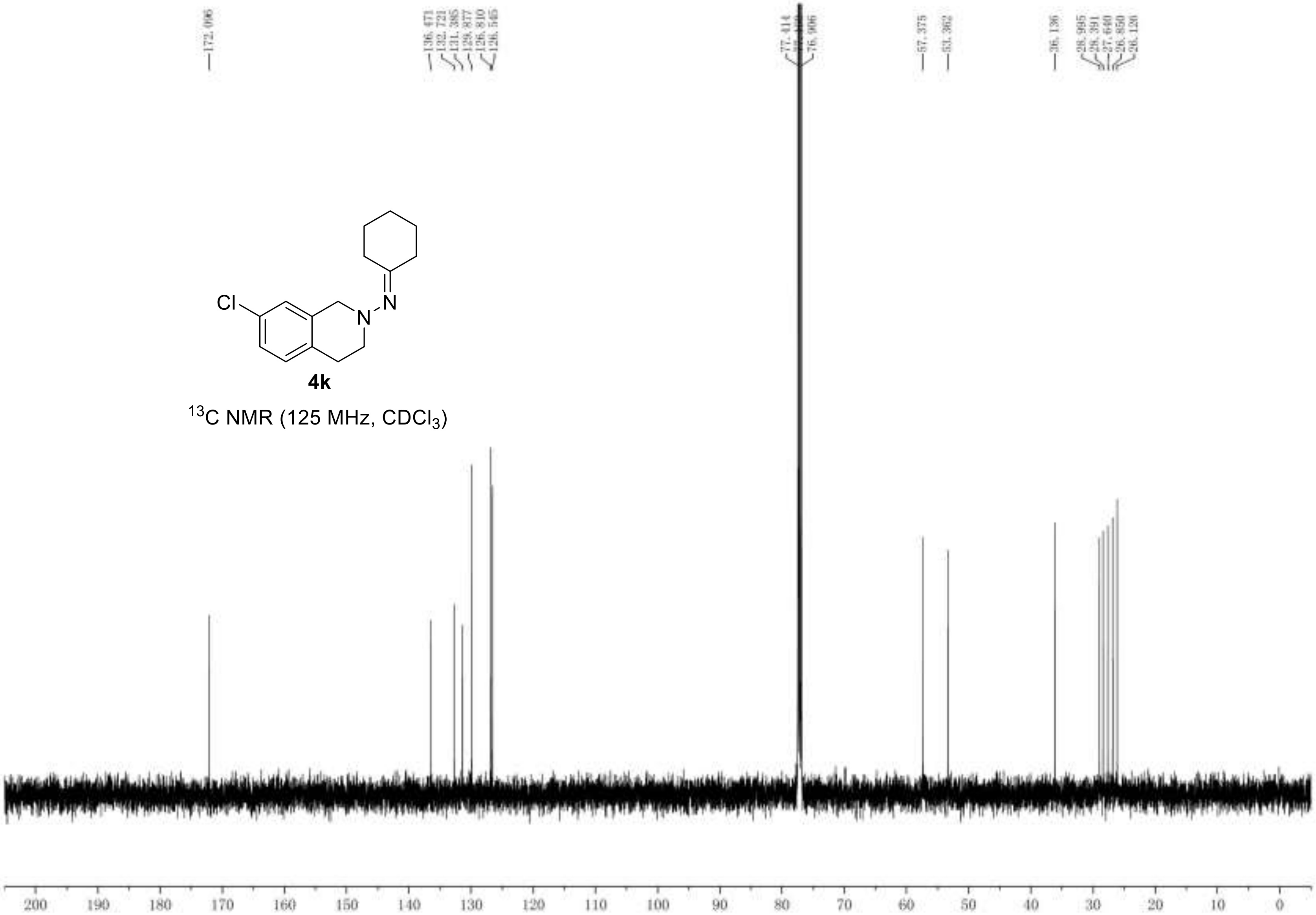

4k

^{13}C NMR (125 MHz, $CDCl_3$)

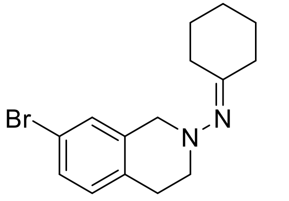

4l

^{1}H NMR (500 MHz, $CDCl_3$)

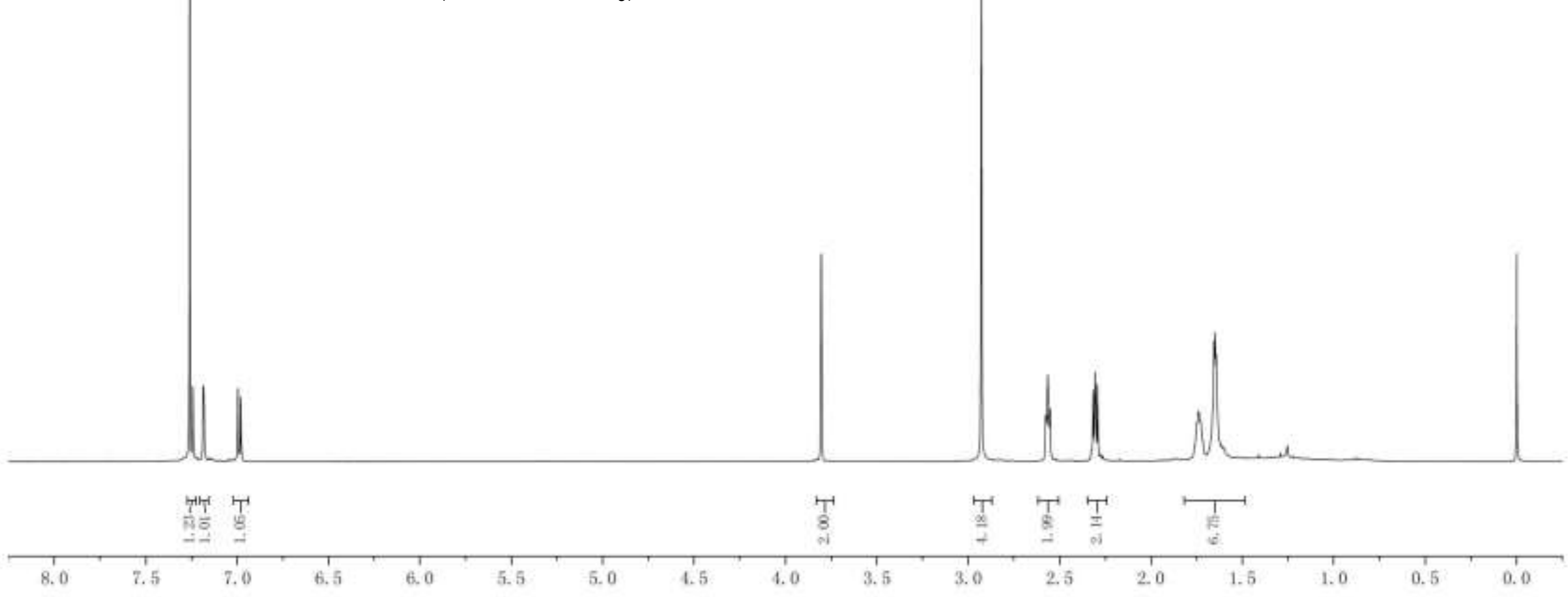

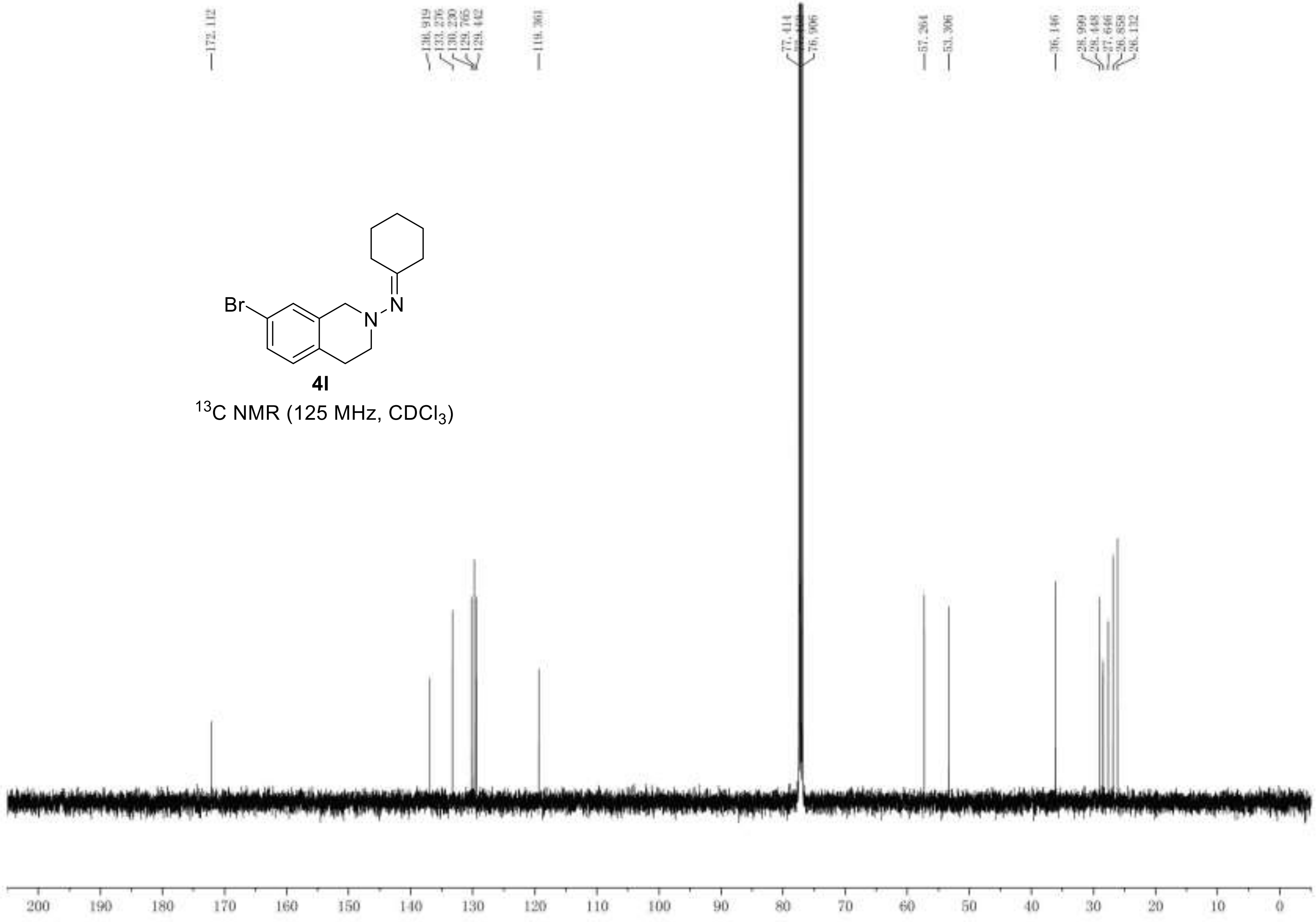

4l

^{13}C NMR (125 MHz, $CDCl_3$)

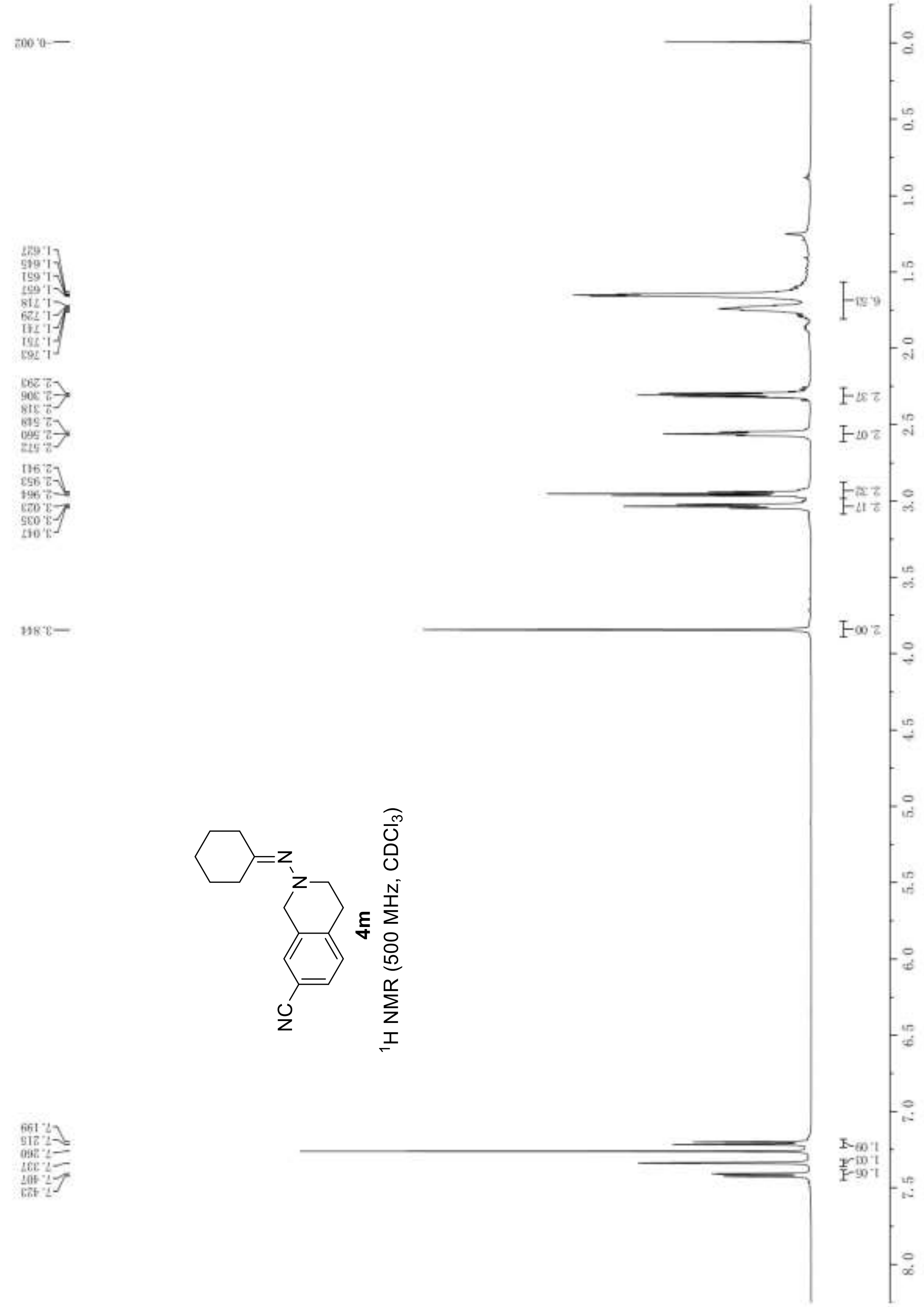

NC
N
N
4m
1H NMR (500 MHz, CDCl3)
-0.002
1.627
1.645
1.651
1.657
1.718
1.729
1.741
1.751
1.763
2.293
2.306
2.318
2.548
2.560
2.572
2.941
2.953
2.964
3.023
3.035
3.047
3.844
7.199
7.215
7.260
7.337
7.407
7.423

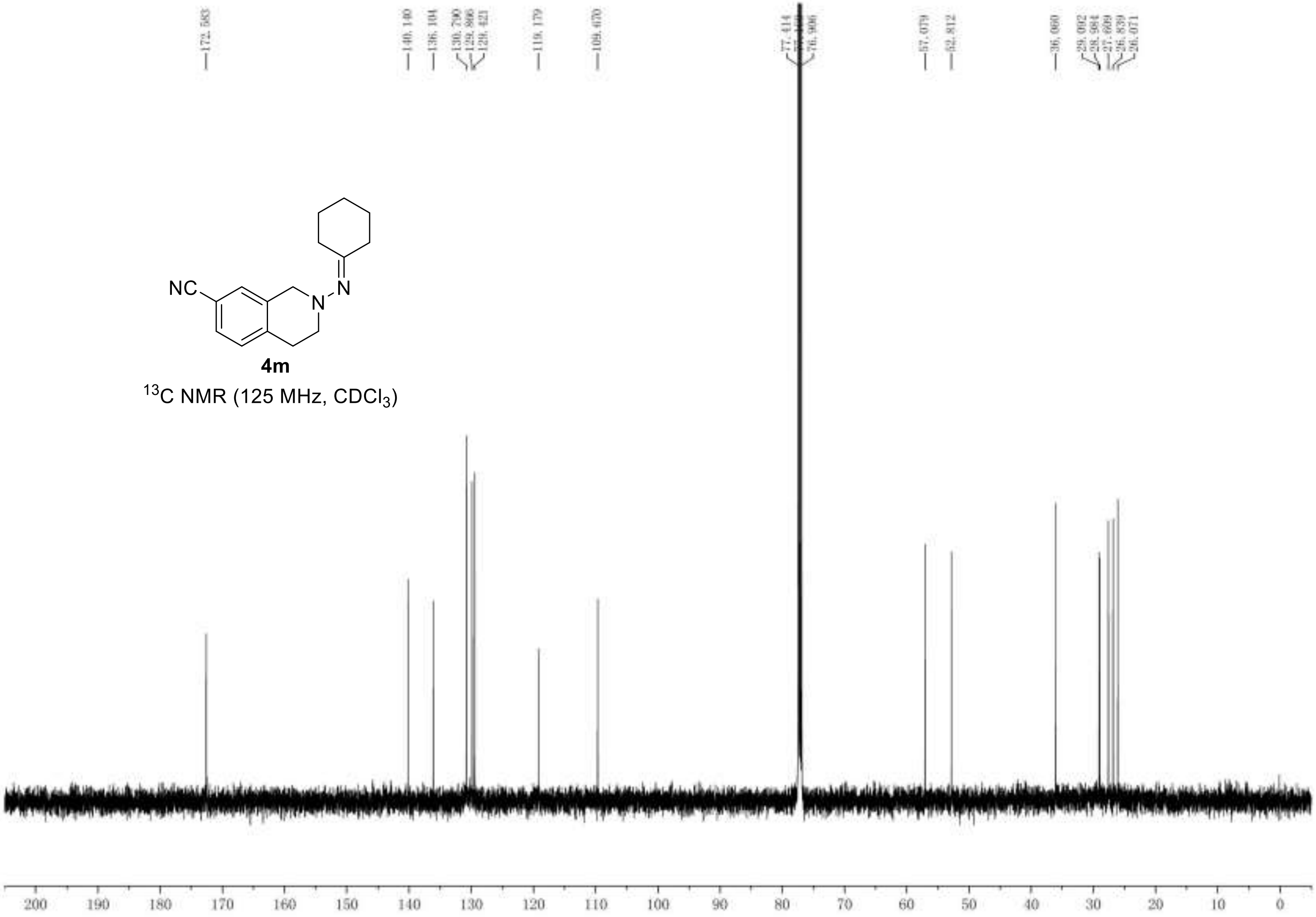

4m

^{13}C NMR (125 MHz, $CDCl_3$)

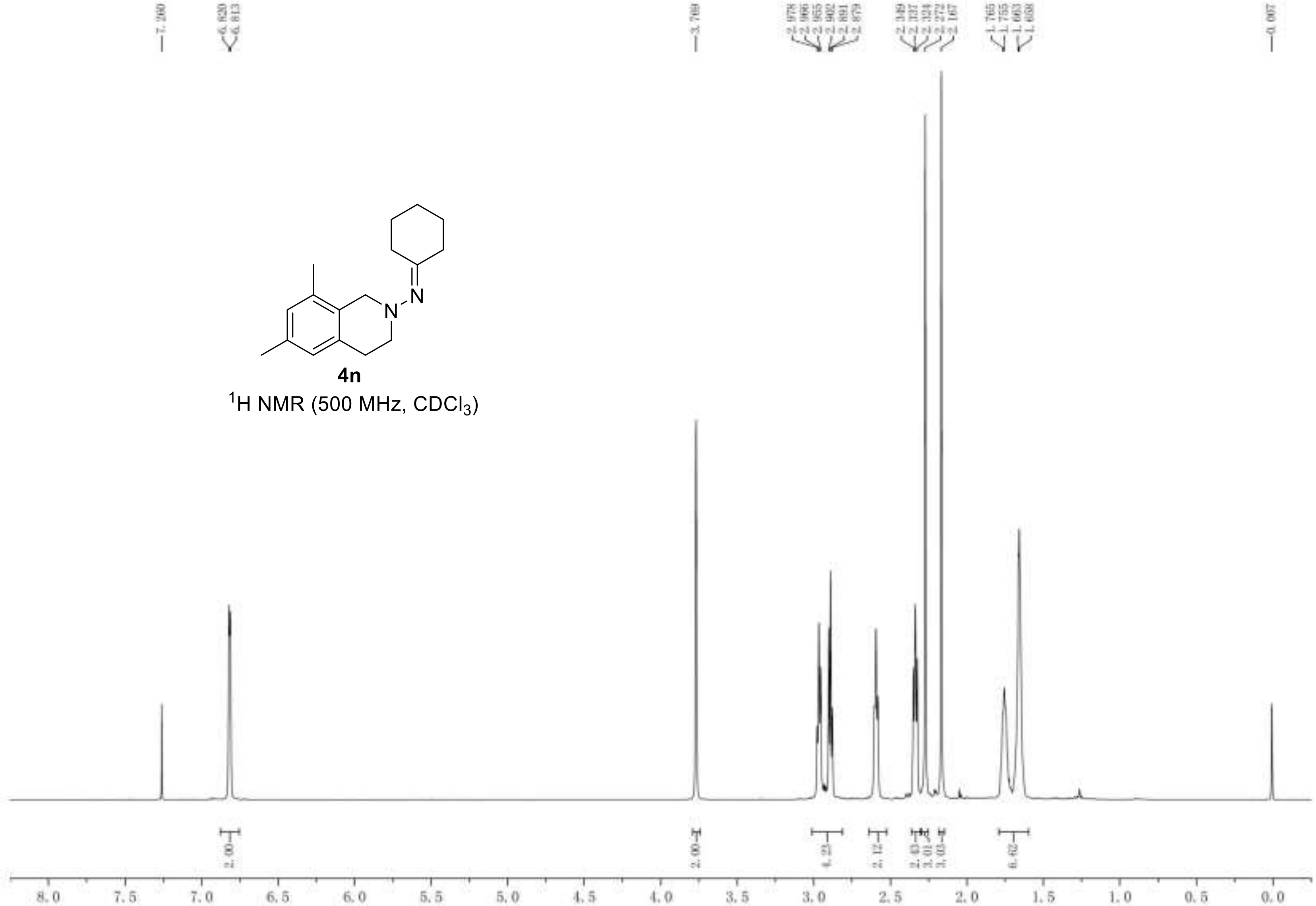

4n

^1H NMR (500 MHz, $CDCl_3$)

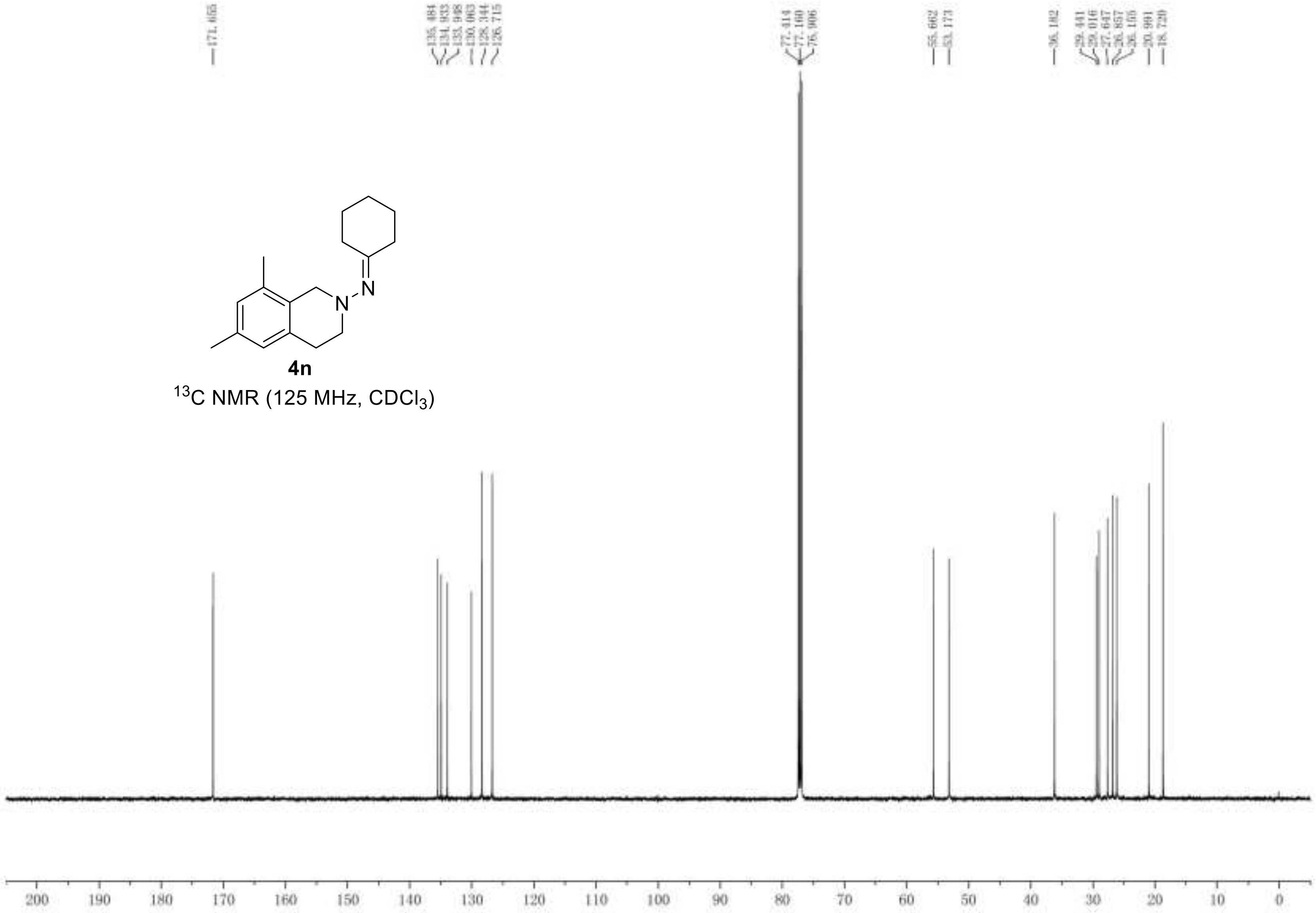

4n

^{13}C NMR (125 MHz, $CDCl_3$)

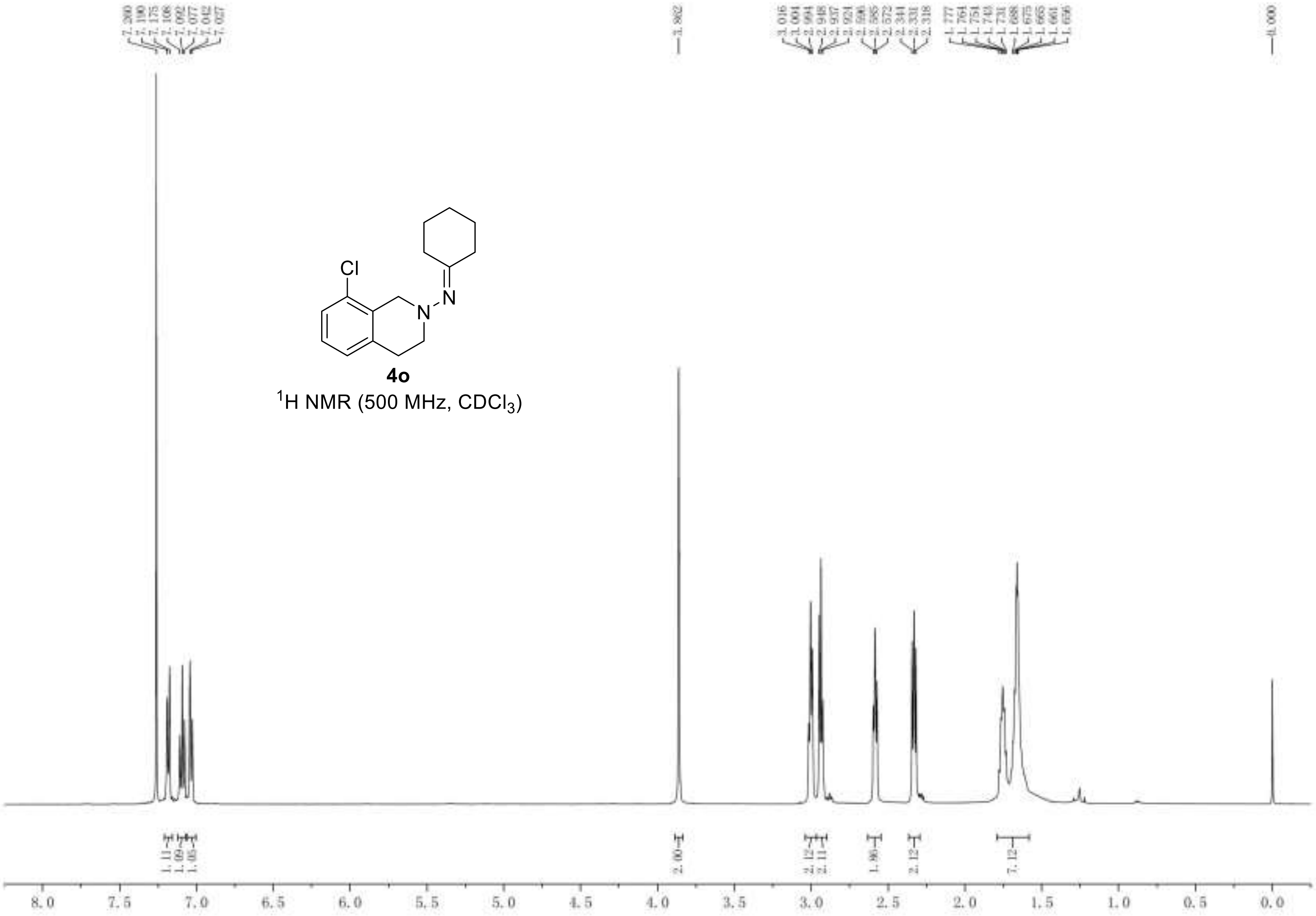

4o

^{1}H NMR (500 MHz, $CDCl_3$)

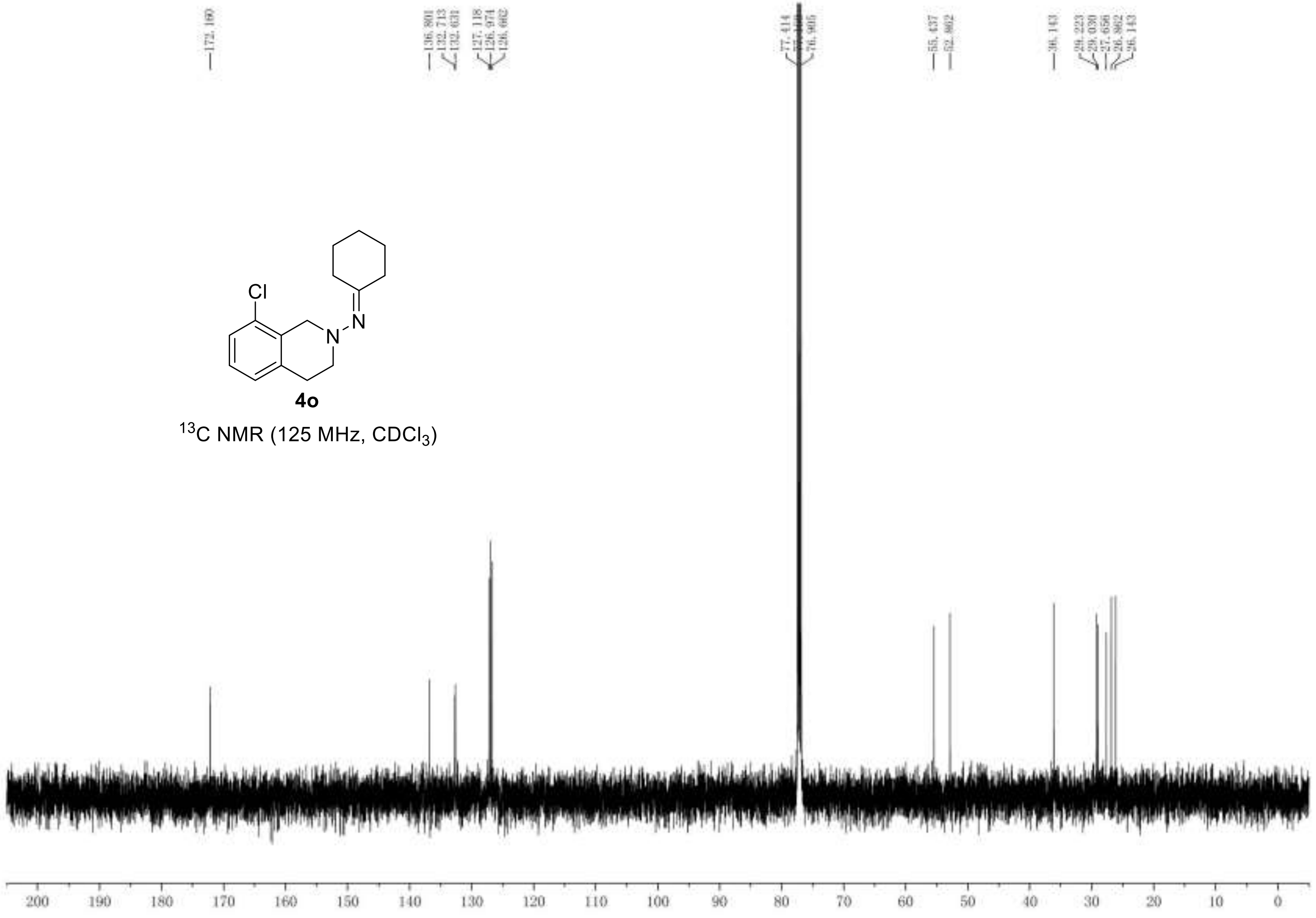

4o

^{13}C NMR (125 MHz, $CDCl_3$)

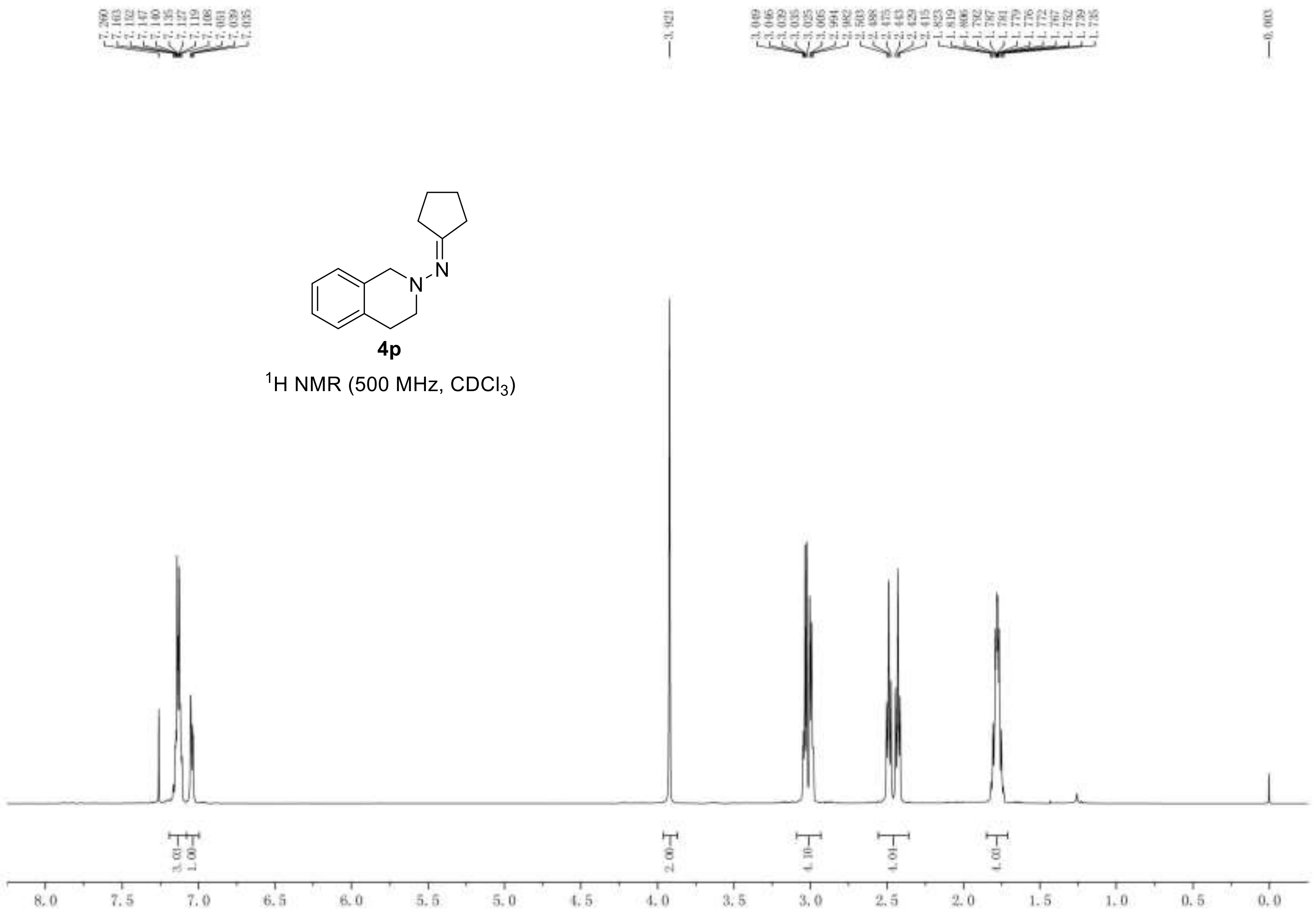

4p

^1H NMR (500 MHz, $CDCl_3$)

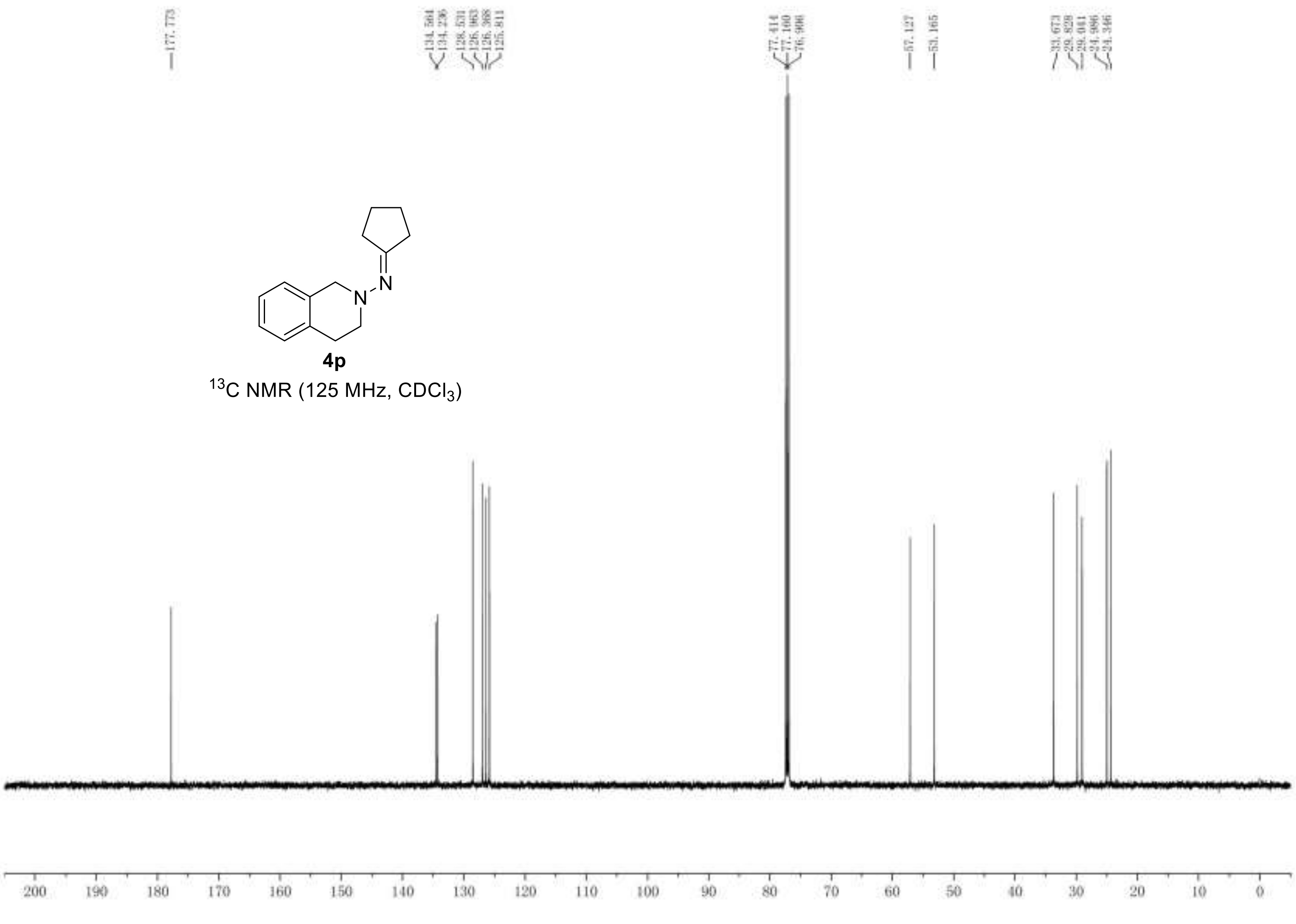

4p

^{13}C NMR (125 MHz, $CDCl_3$)

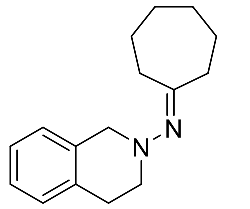

4q

^{1}H NMR (500 MHz, $CDCl_3$)

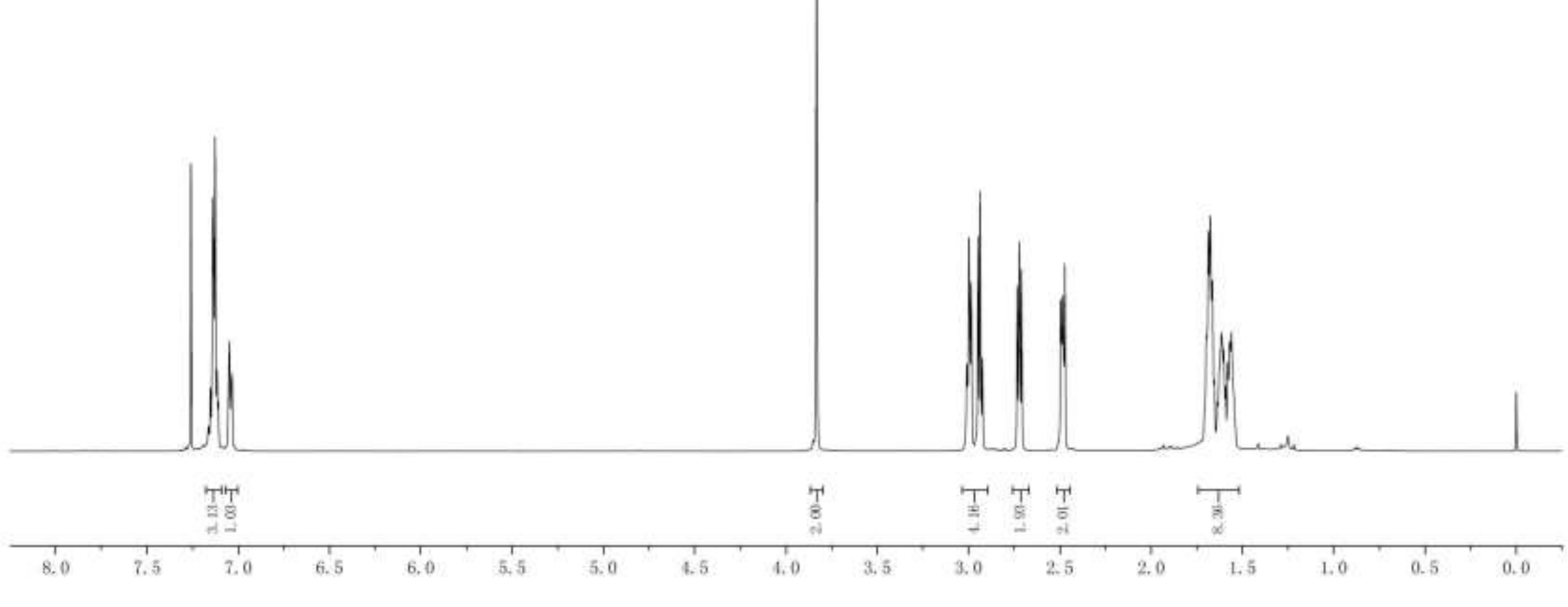

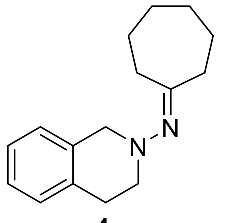

4q

^{13}C NMR (125 MHz, $CDCl_3$)

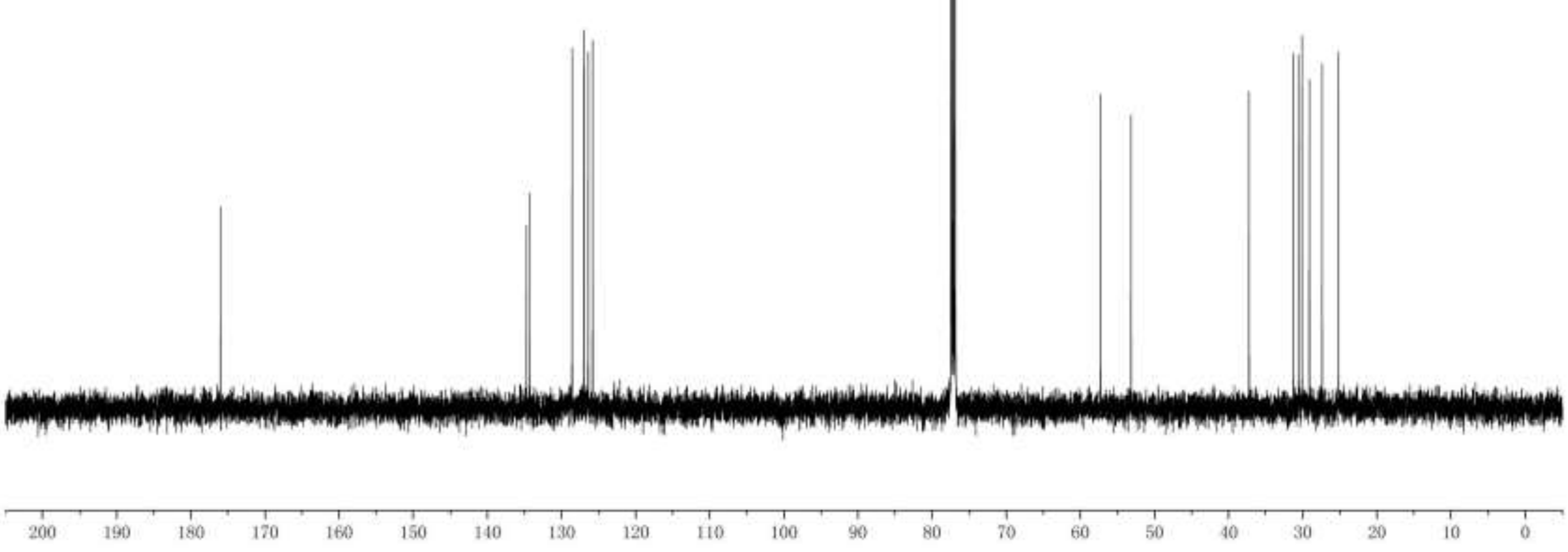

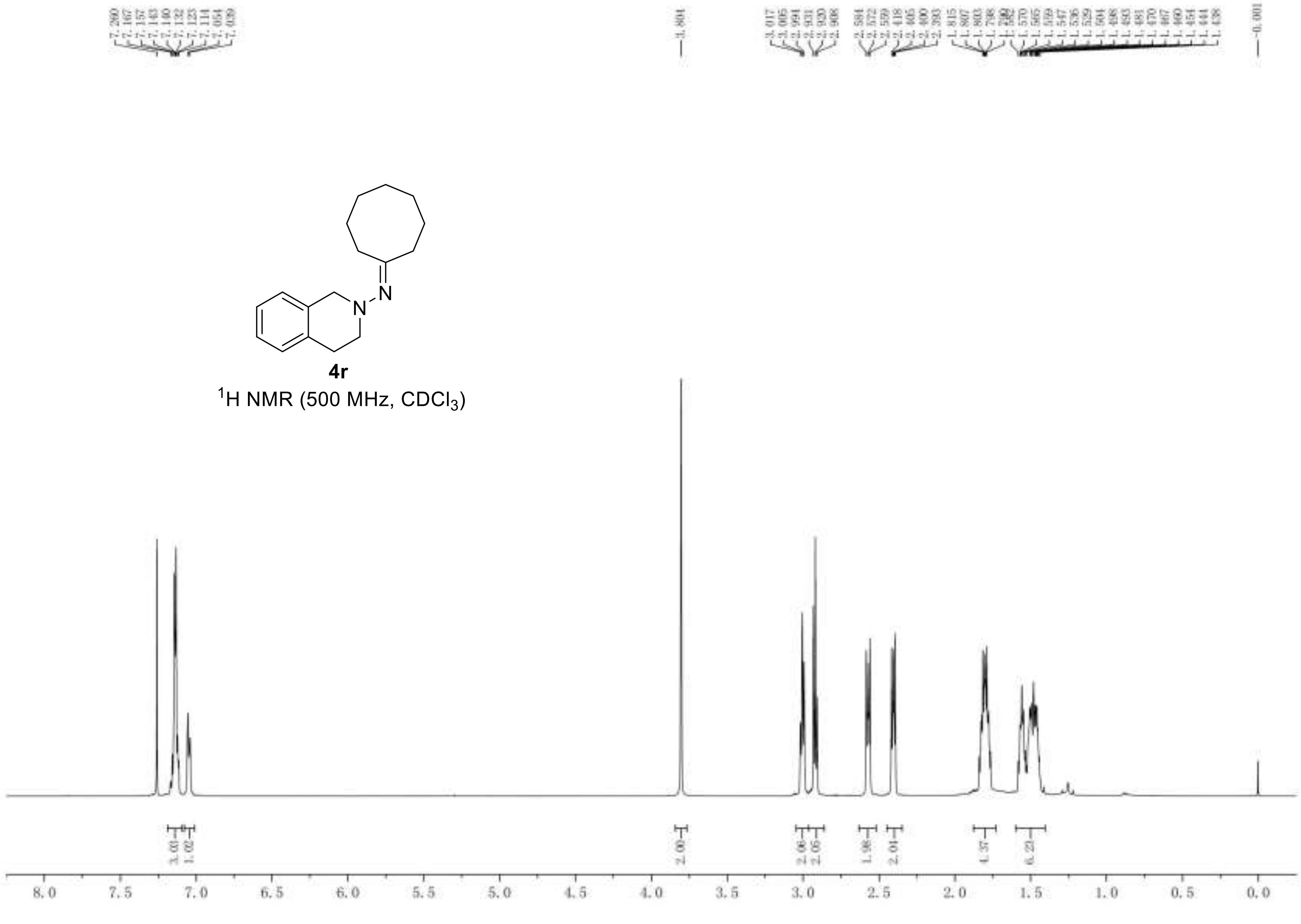

4r

^{1}H NMR (500 MHz, $CDCl_3$)

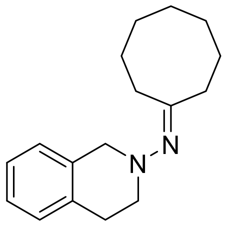

4r

^{13}C NMR (125 MHz, $CDCl_3$)

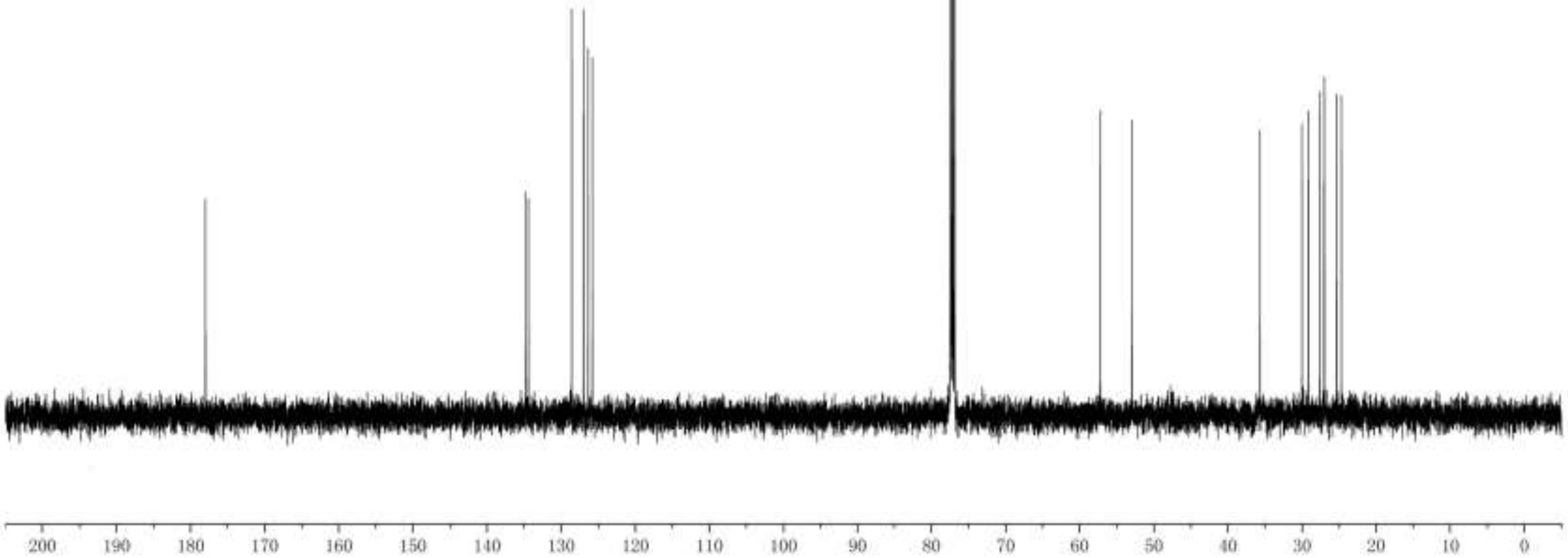

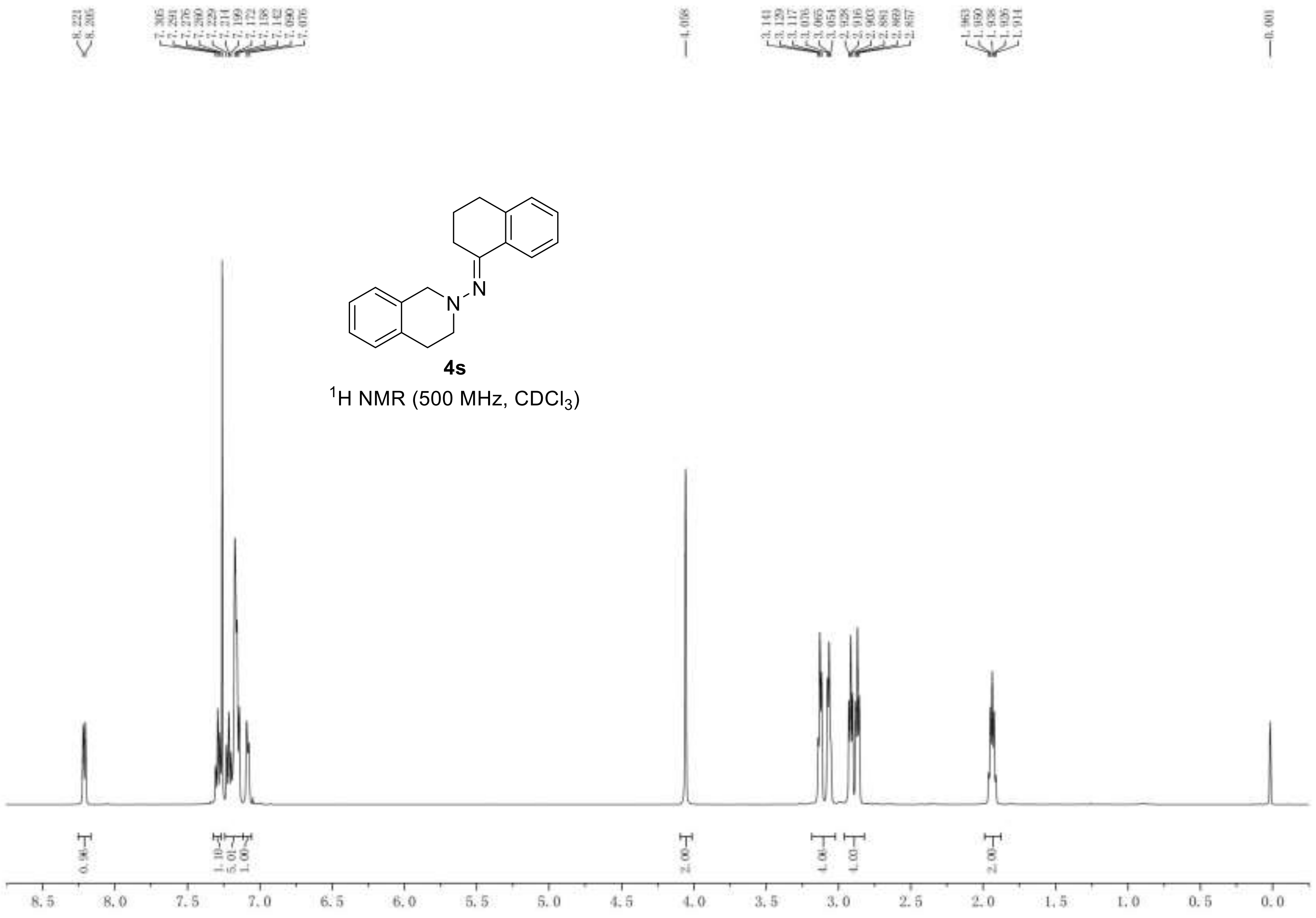
4s
^{1}H NMR (500 MHz, CDCl$_3$)

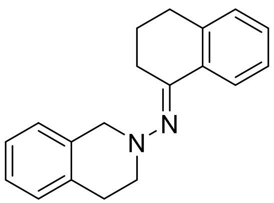

4s

^{13}C NMR (125 MHz, $CDCl_3$)

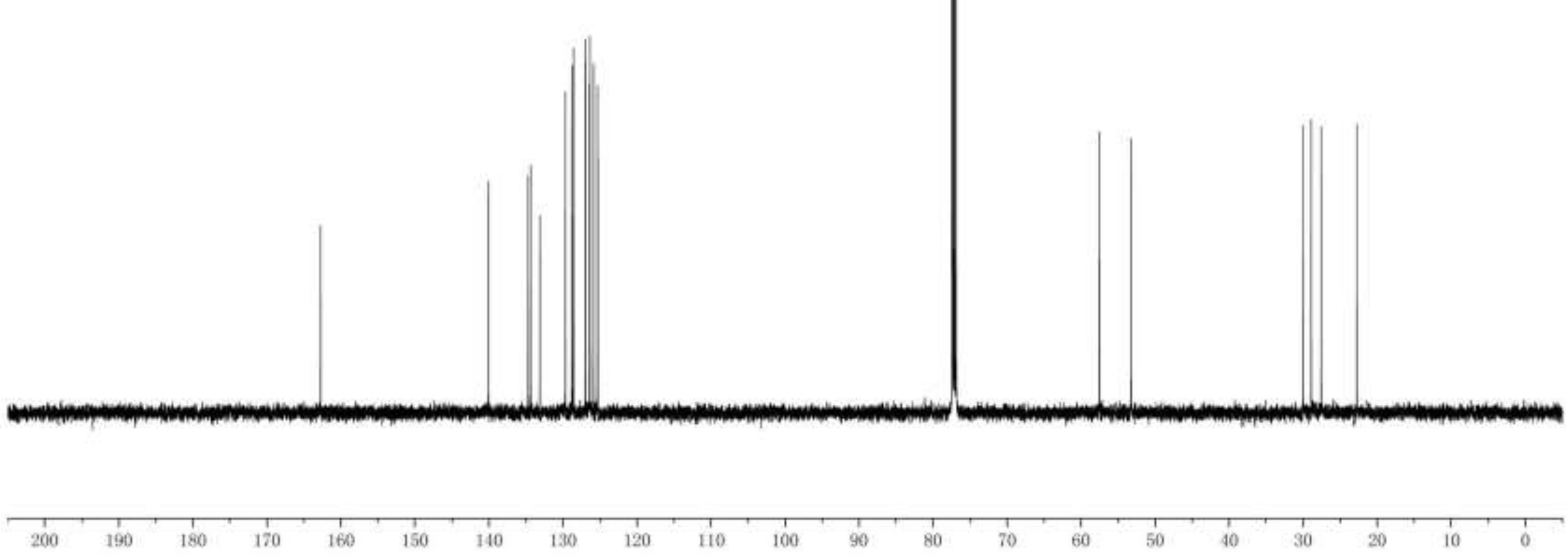

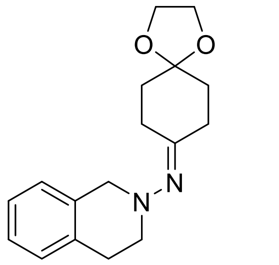

4t

^{1}H NMR (500 MHz, $CDCl_3$)

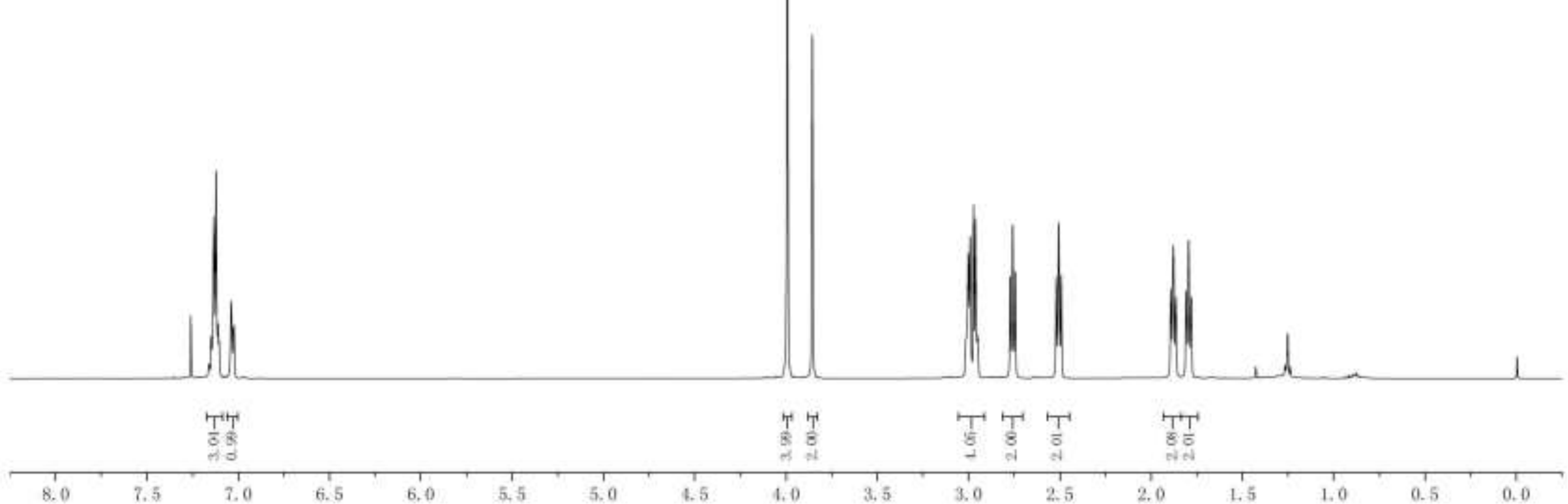

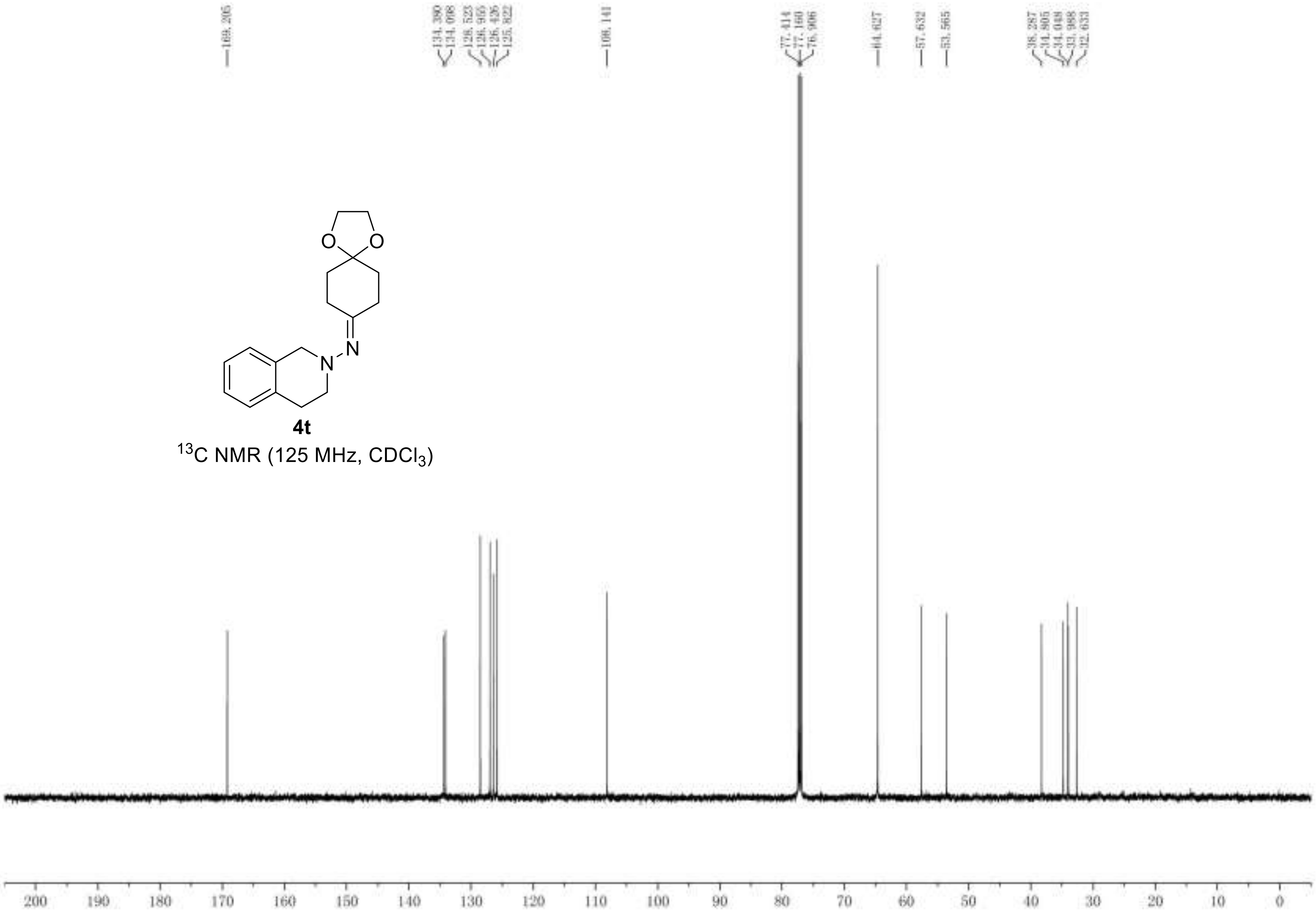

4t

^{13}C NMR (125 MHz, $CDCl_3$)

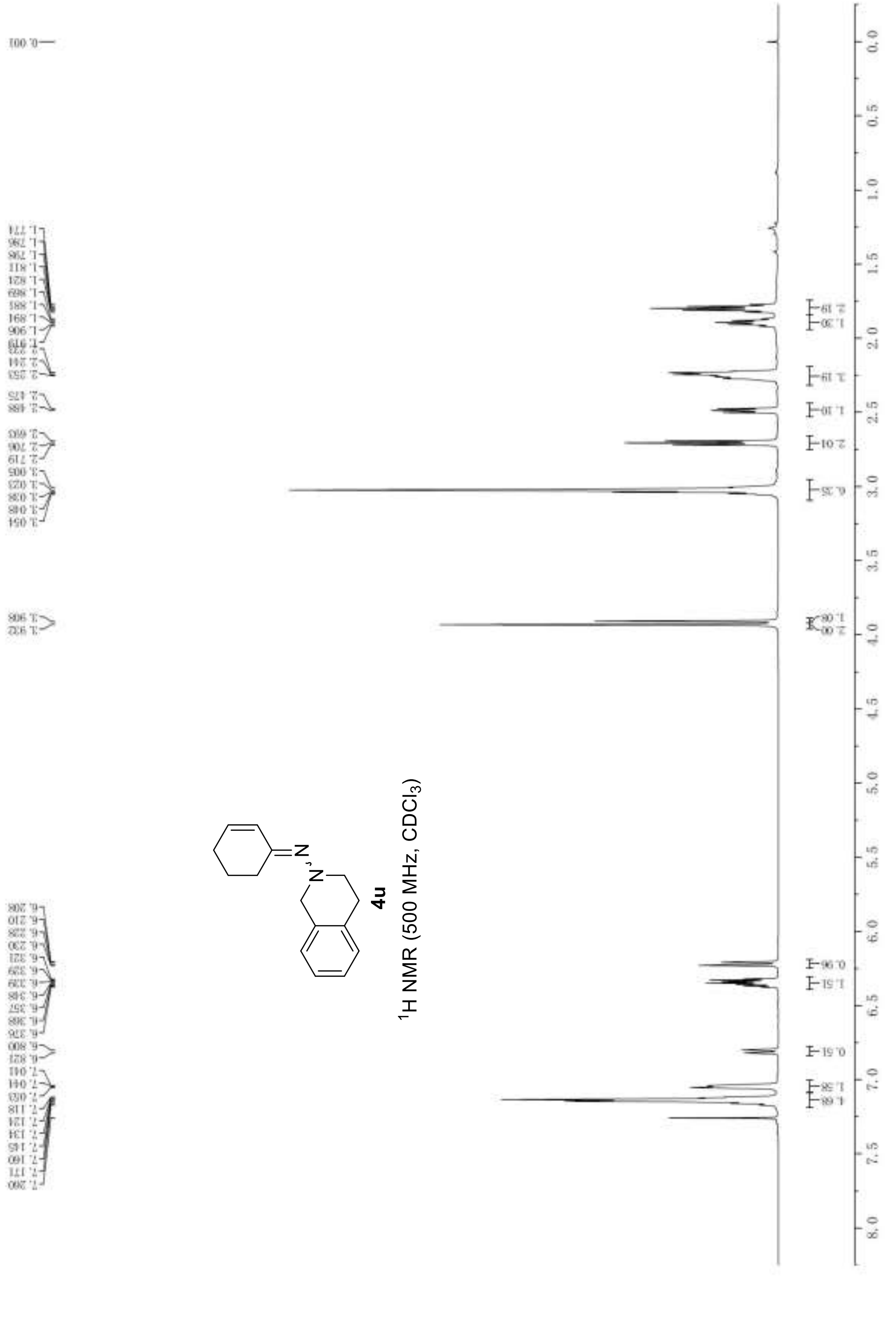

4u

^1H NMR (500 MHz, $CDCl_3$)

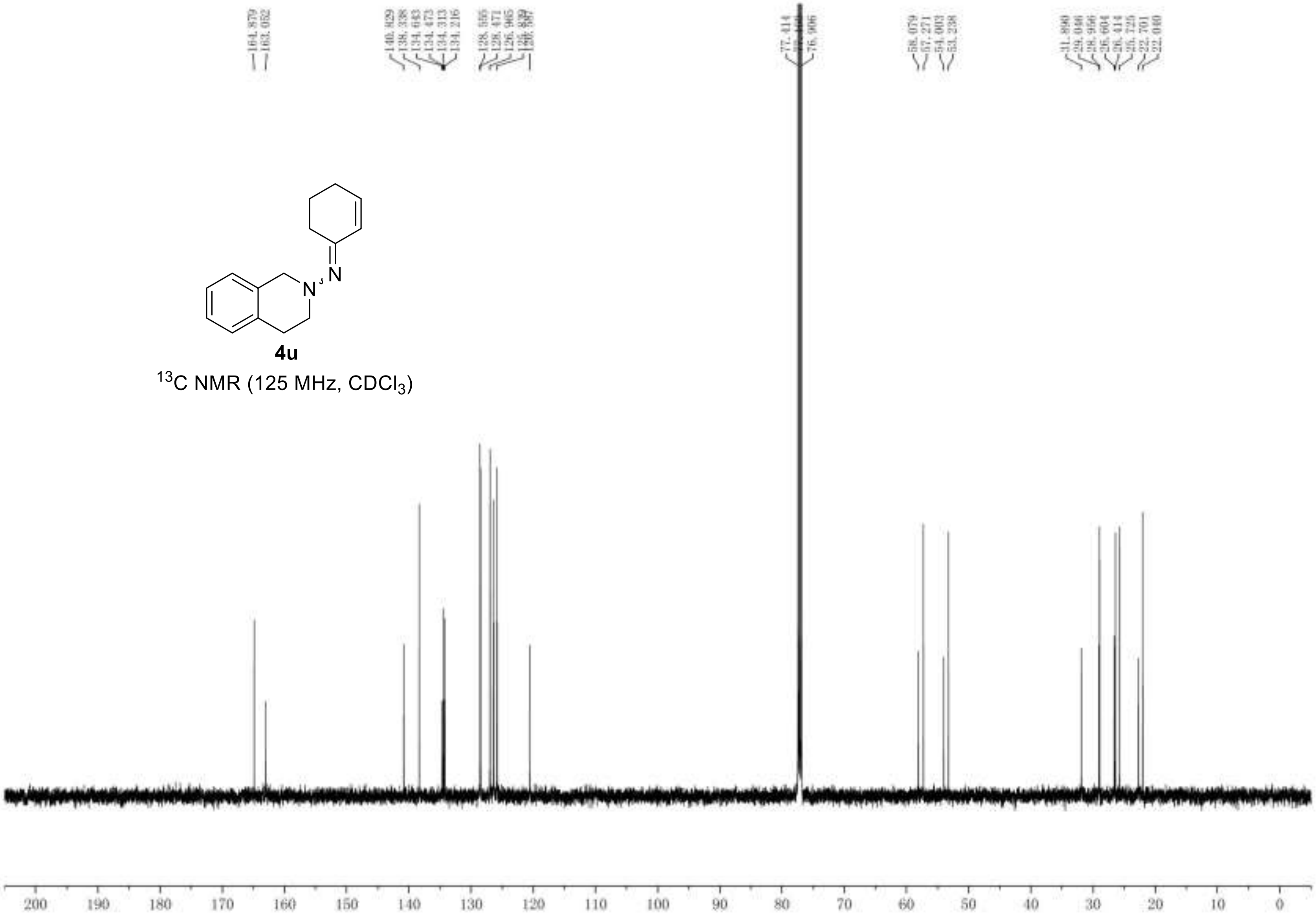

4u

^{13}C NMR (125 MHz, $CDCl_3$)

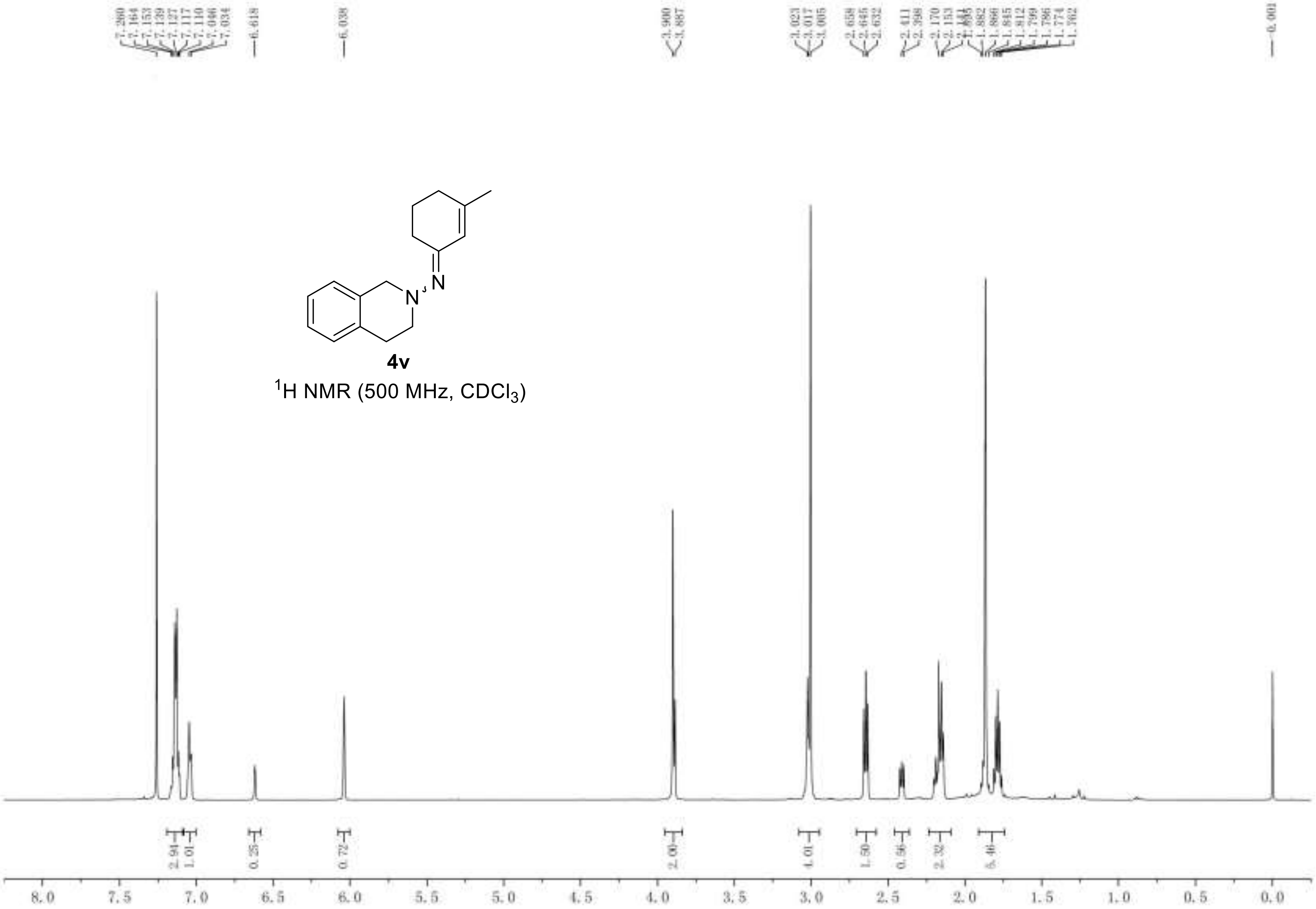

4v

^{1}H NMR (500 MHz, $CDCl_3$)

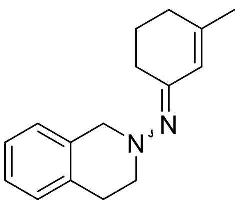

4v

^{13}C NMR (125 MHz, $CDCl_3$)

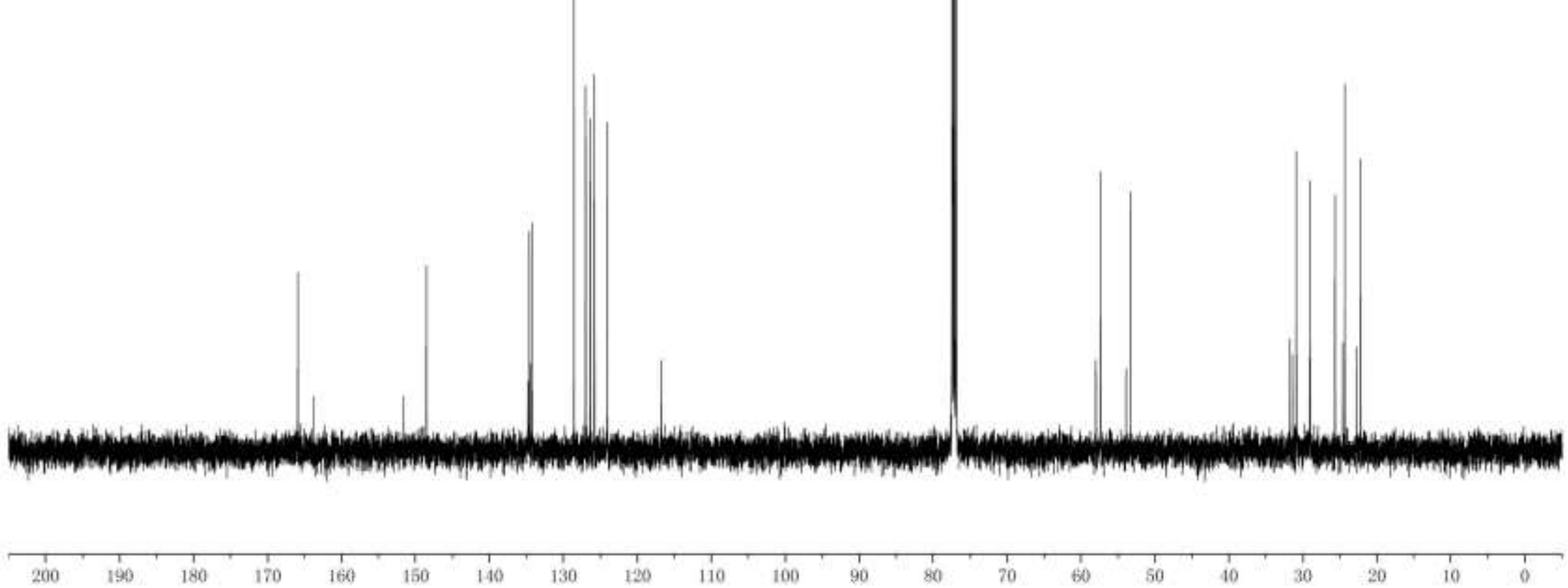

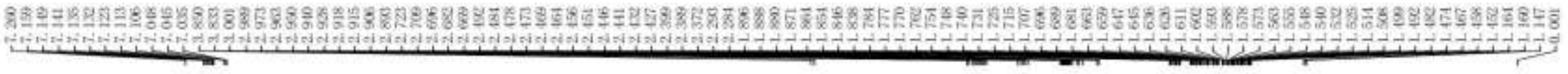

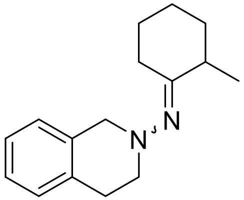

4w

^{1}H NMR (500 MHz, $CDCl_3$)

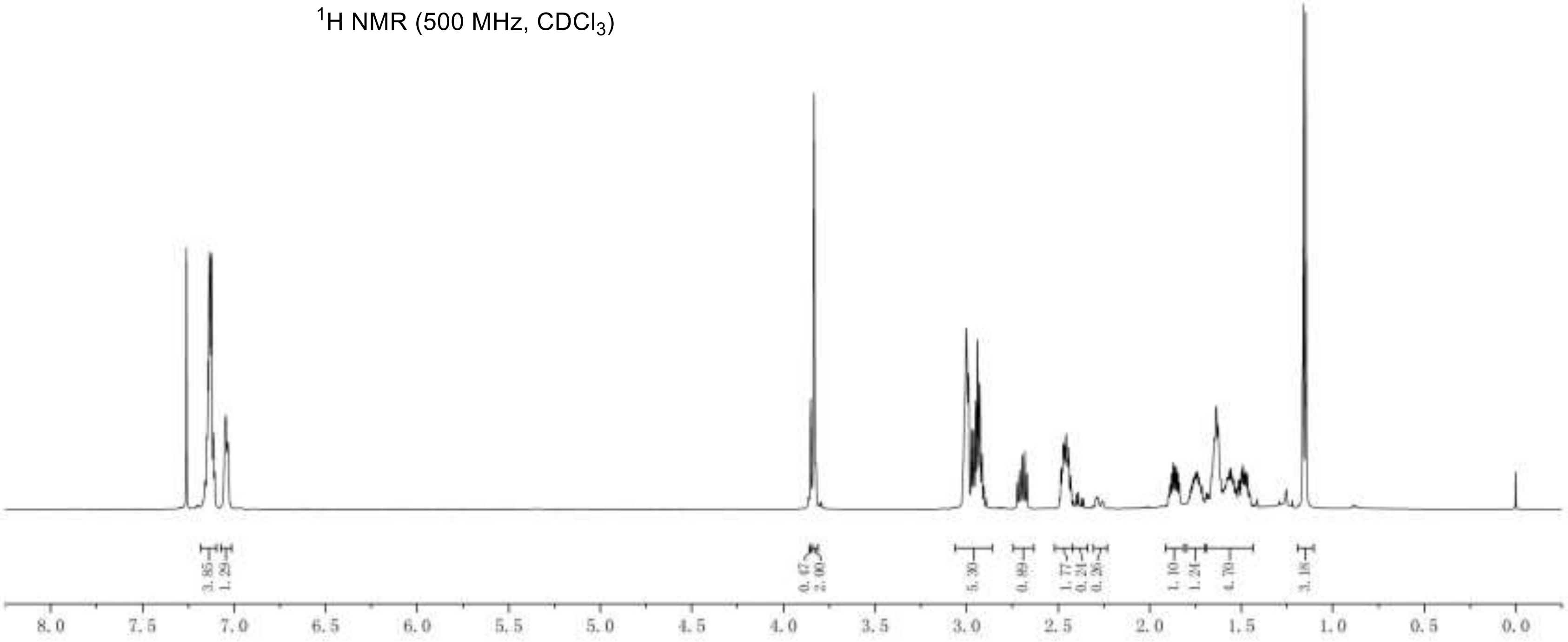

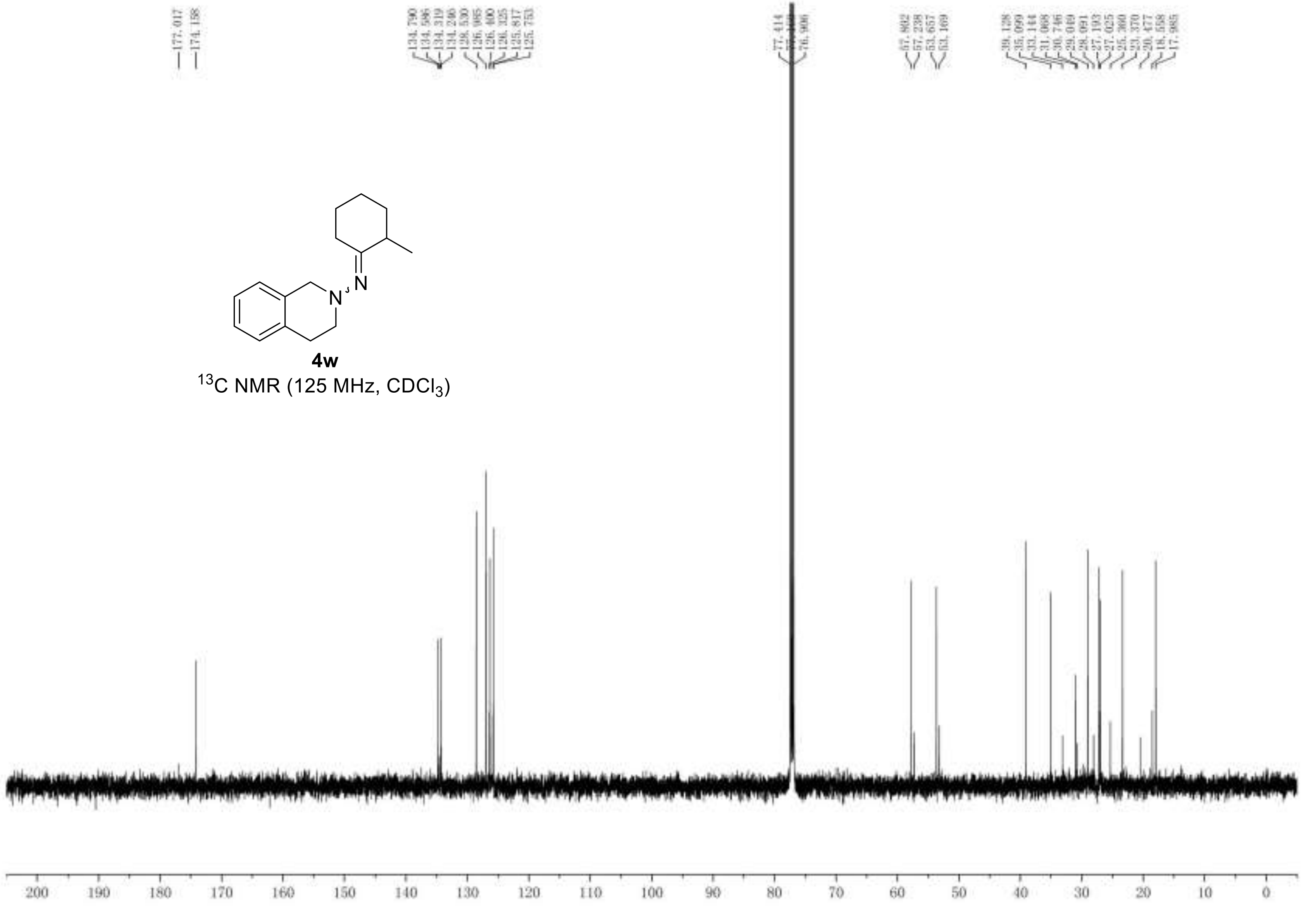

4w
^{13}C NMR (125 MHz, $CDCl_3$)

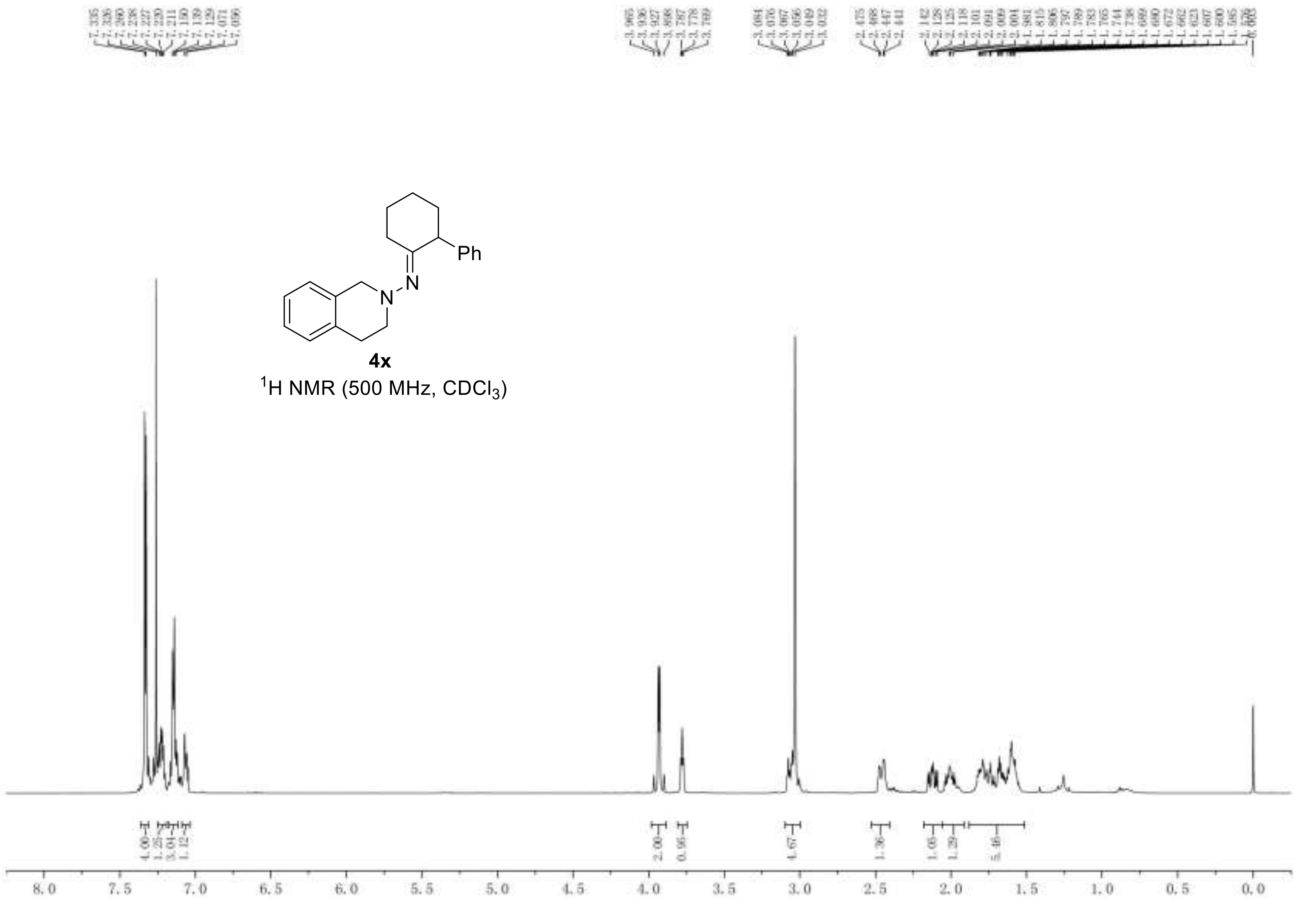

4x

^1H NMR (500 MHz, $CDCl_3$)

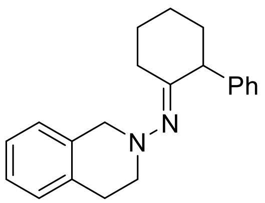

4x

^{13}C NMR (125 MHz, $CDCl_3$)

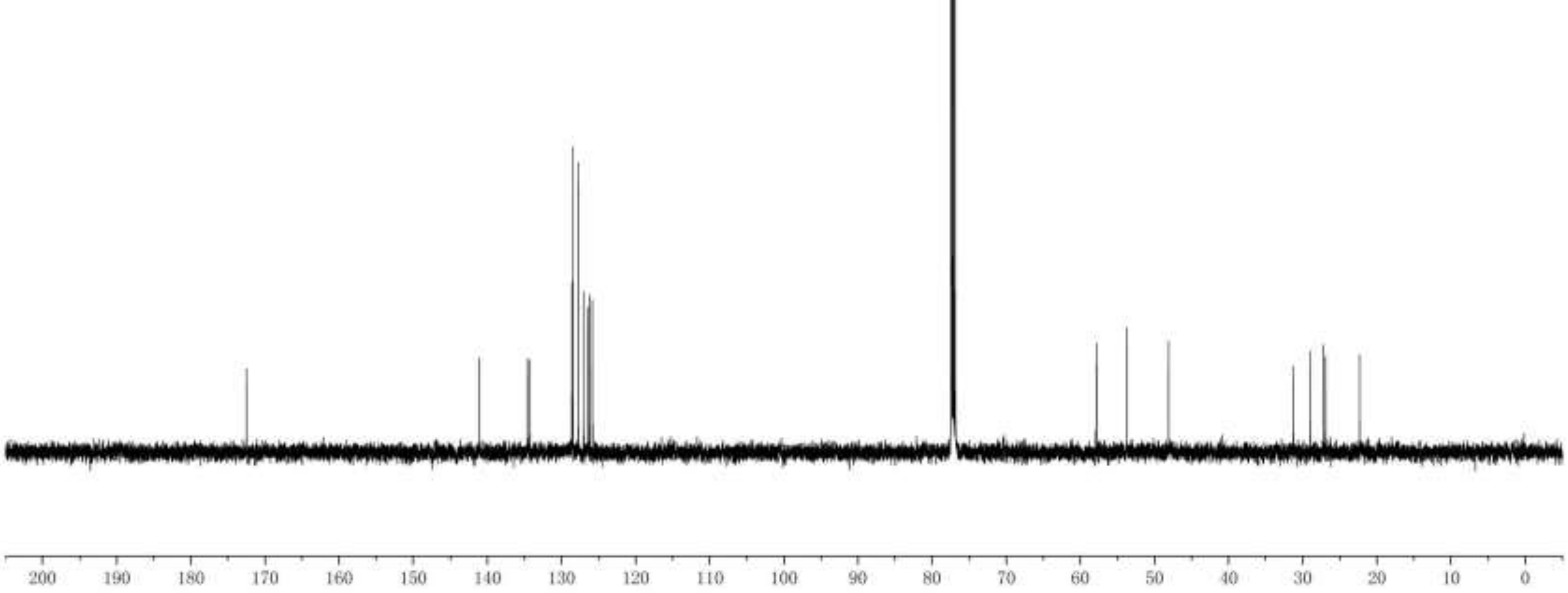

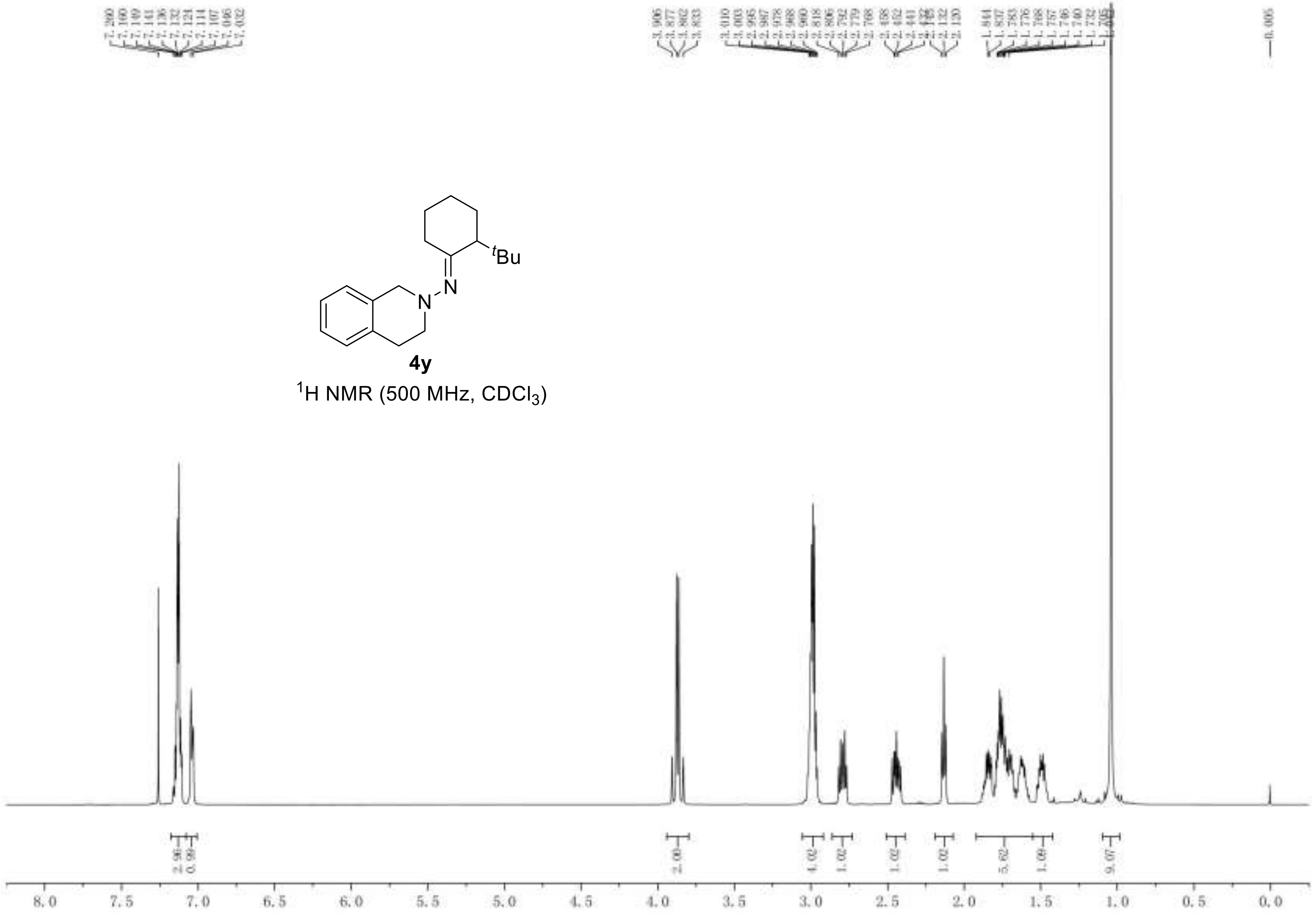

4y

^{1}H NMR (500 MHz, $CDCl_3$)

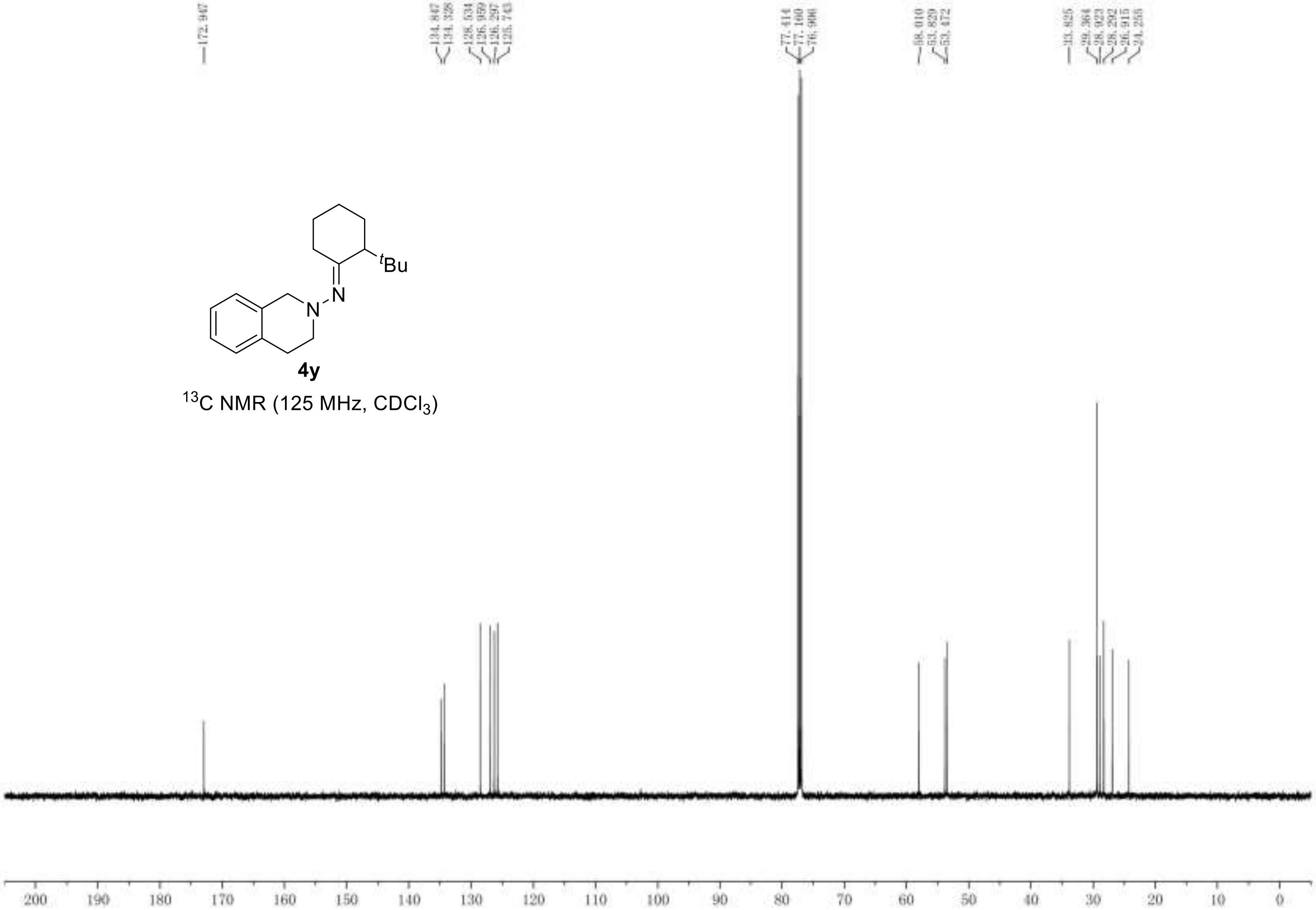

4y

^{13}C NMR (125 MHz, $CDCl_3$)

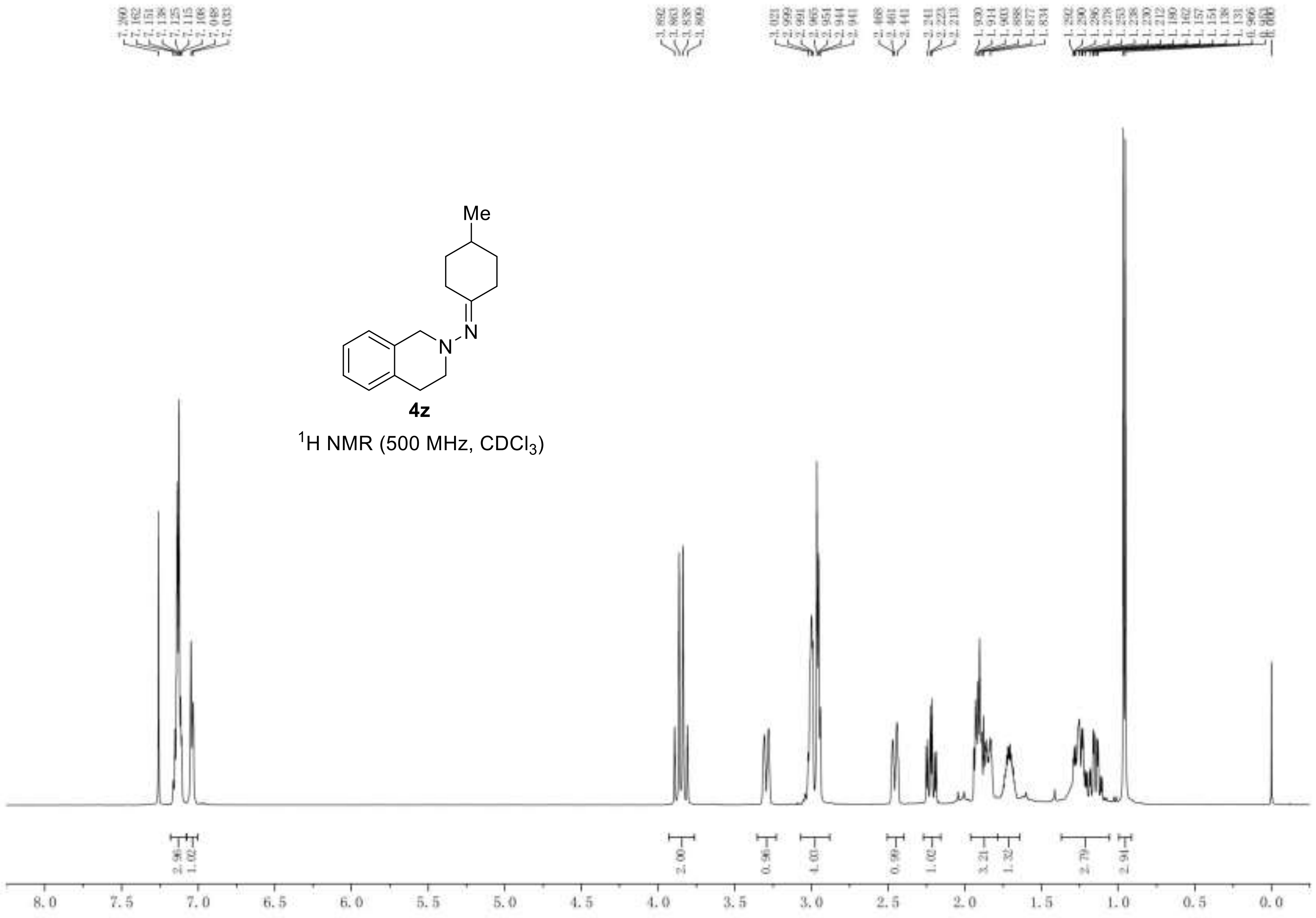

4z

^1H NMR (500 MHz, $CDCl_3$)

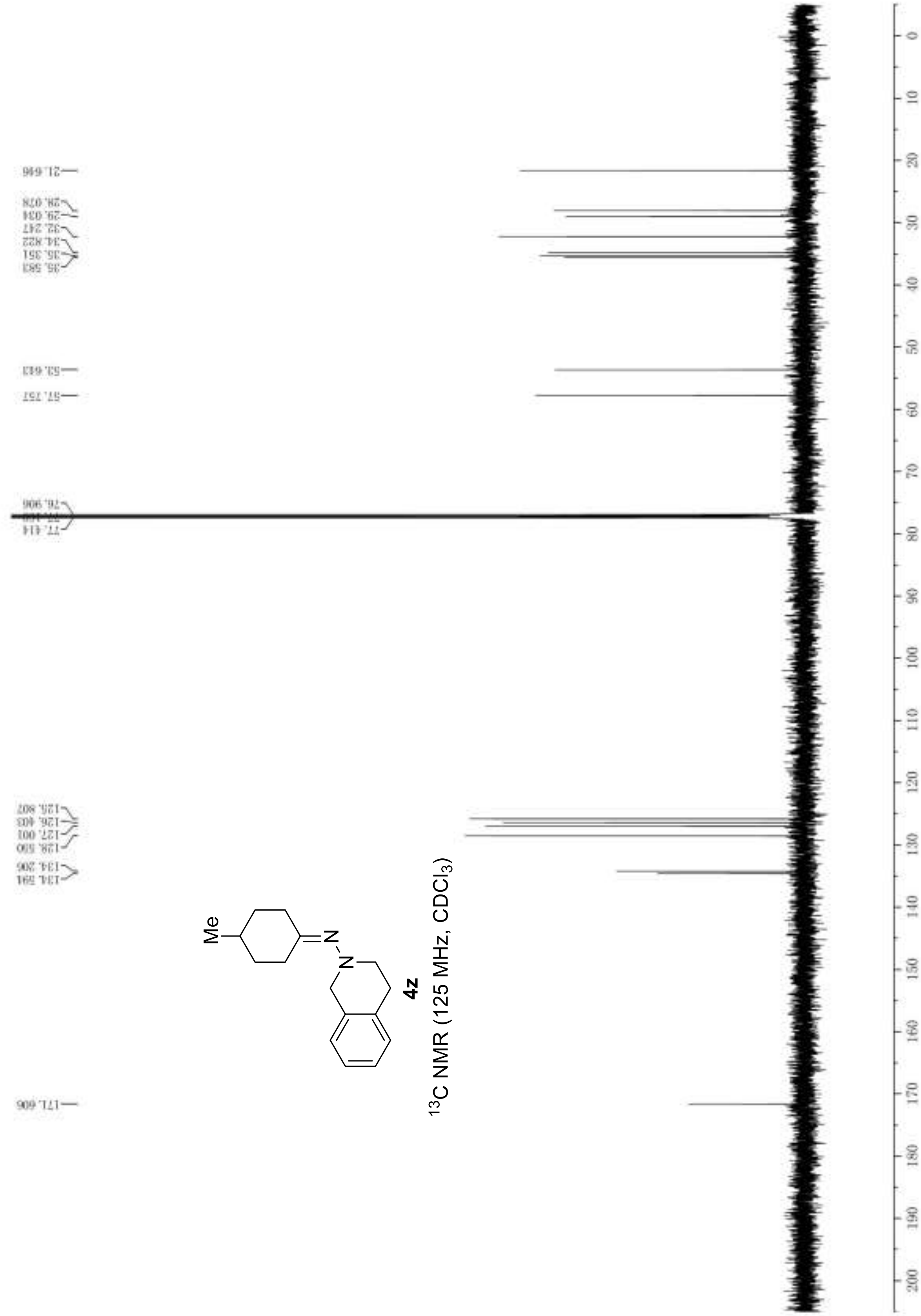
21.646
28.078
29.034
32.247
34.822
35.351
35.583
53.643
57.757
76.906
77.414
125.807
126.403
127.001
128.590
134.206
134.594
171.606
Me
N
N
4z
13C NMR (125 MHz, CDCl3)
0
10
20
30
40
50
60
70
80
90
100
110
120
130
140
150
160
170
180
190
200

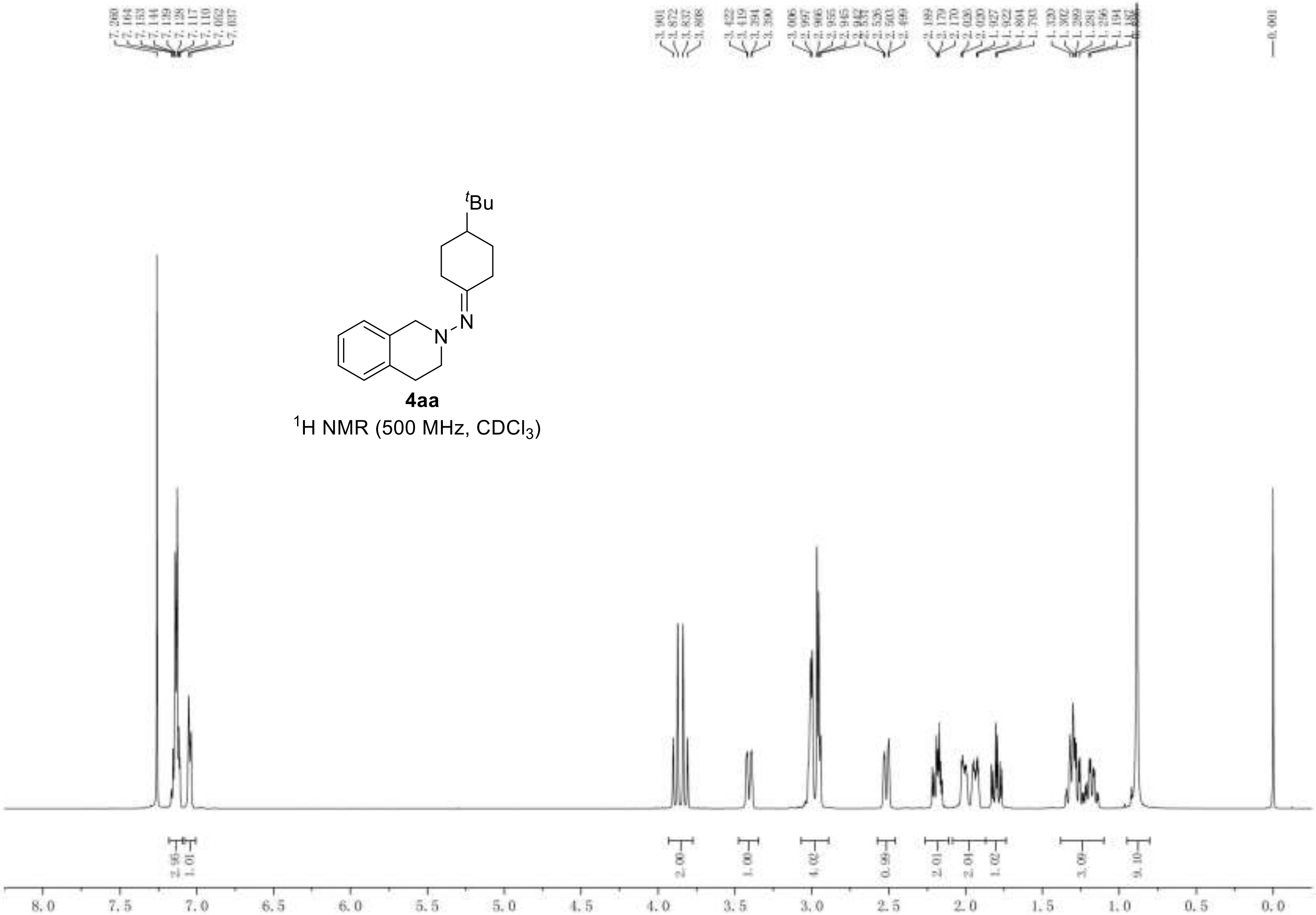

4aa

^1H NMR (500 MHz, $CDCl_3$)

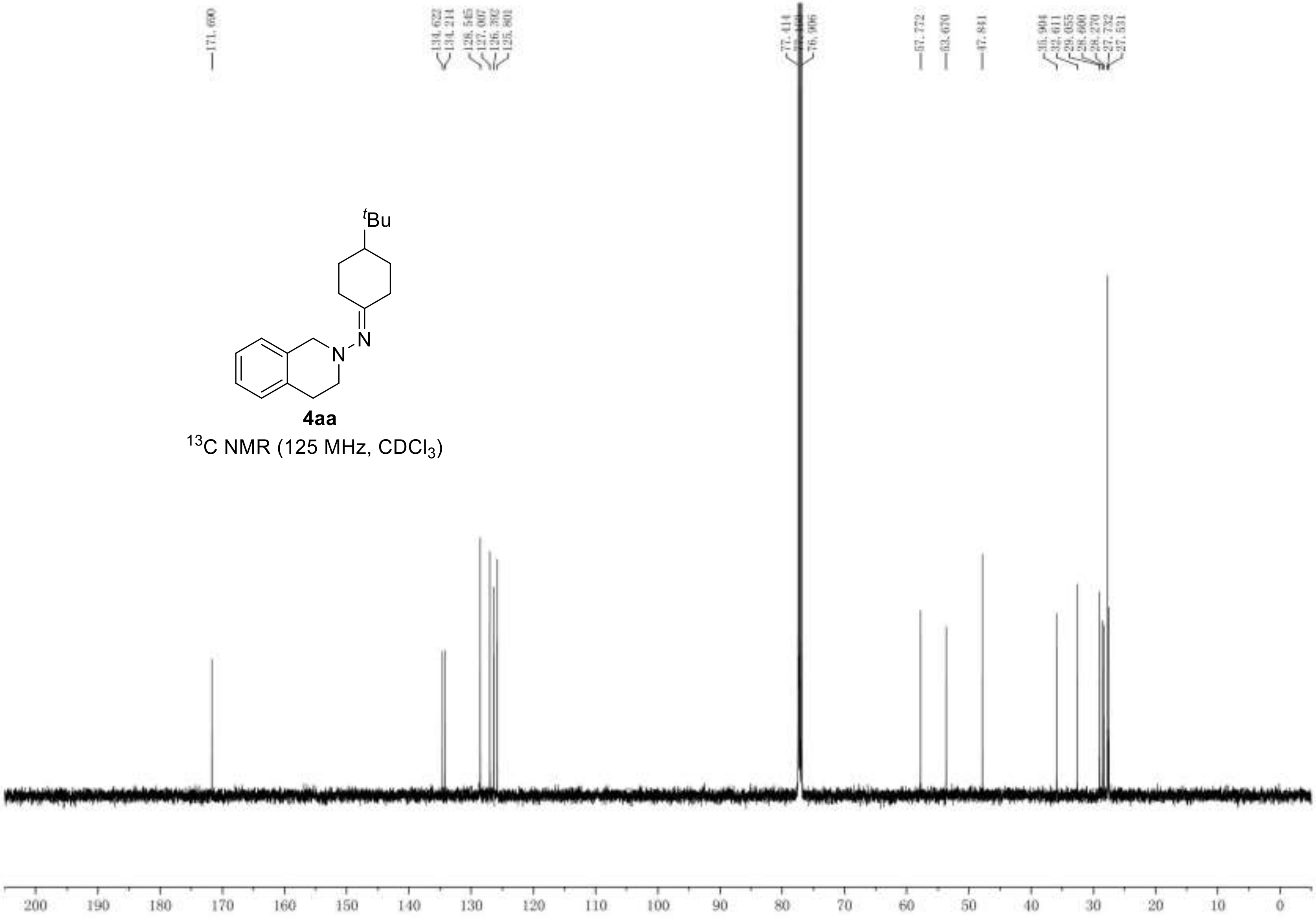

4aa

^{13}C NMR (125 MHz, $CDCl_3$)

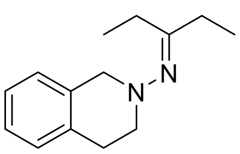

4ab

^{1}H NMR (500 MHz, $CDCl_3$)

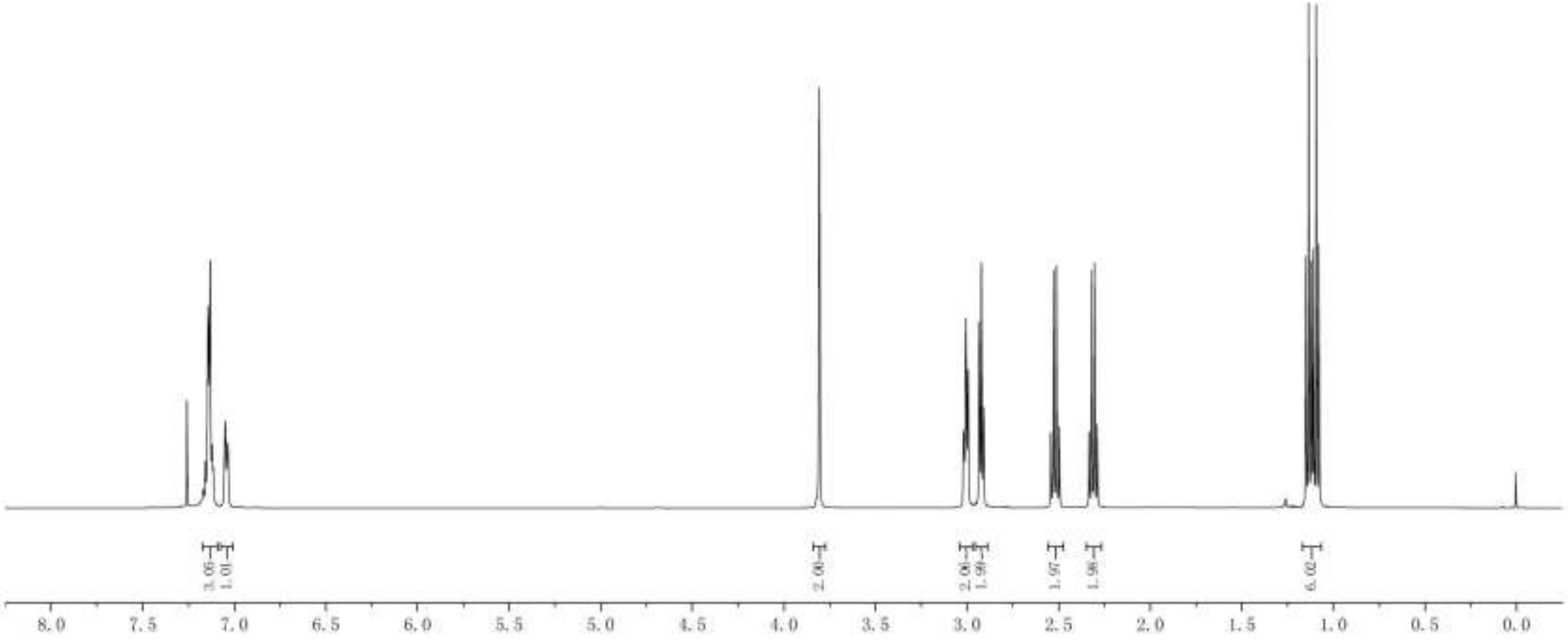

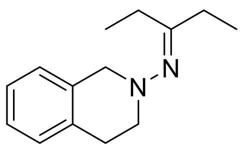

4ab

^{13}C NMR (125 MHz, $CDCl_3$)

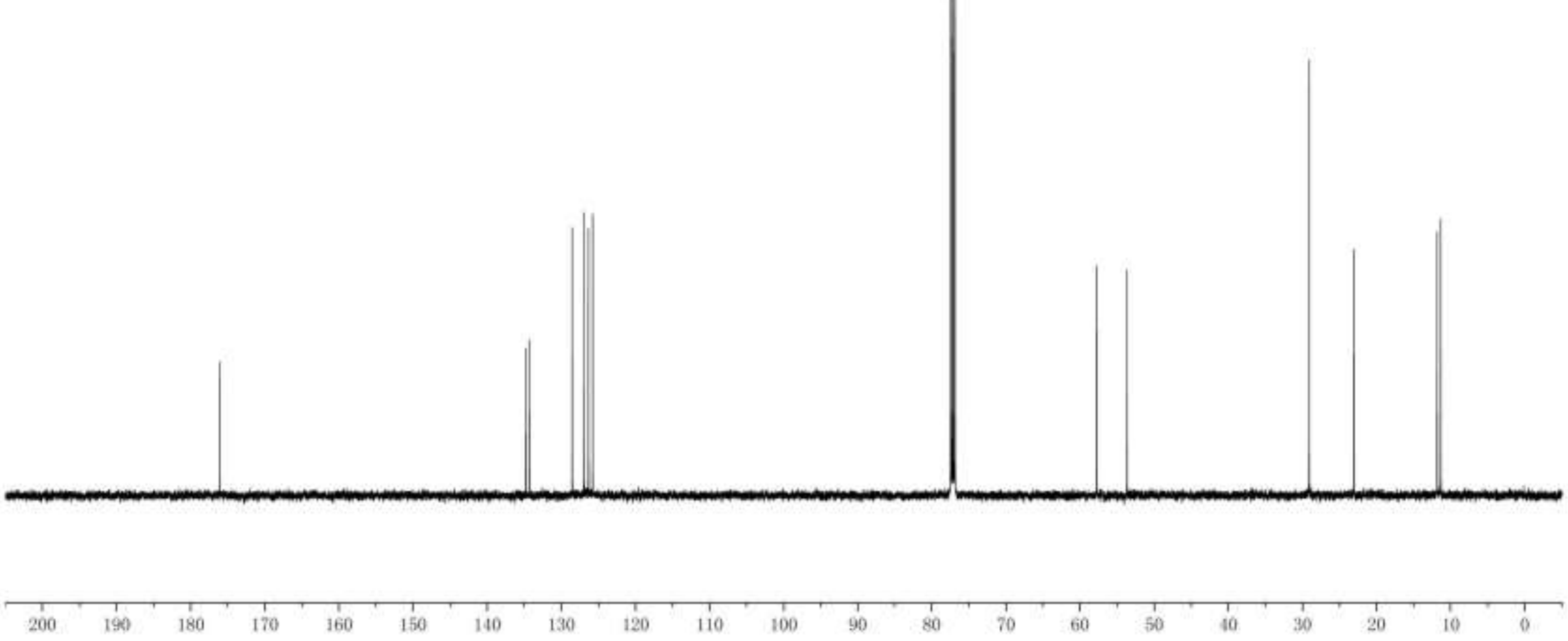

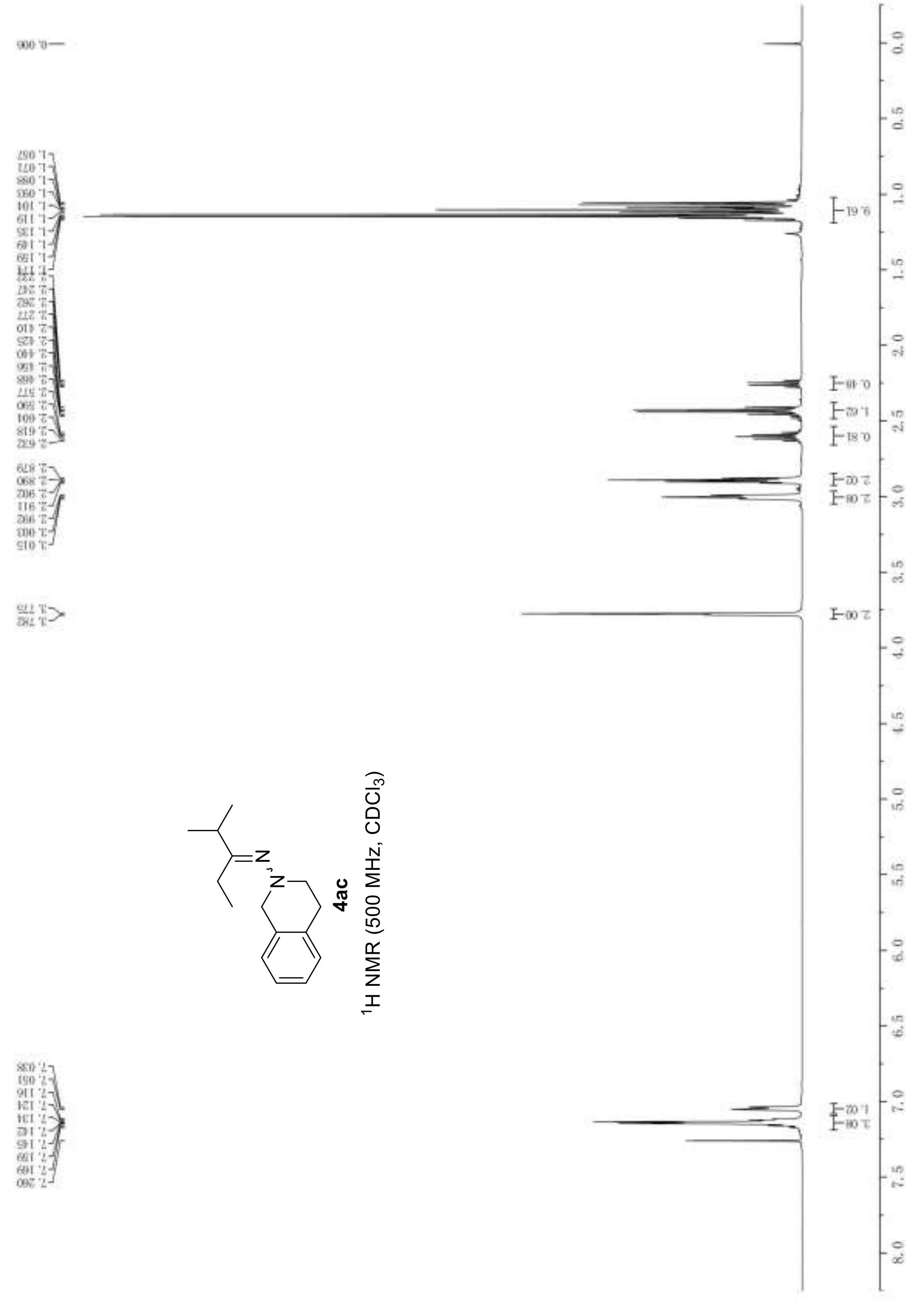

4ac

^{1}H NMR (500 MHz, $CDCl_3$)

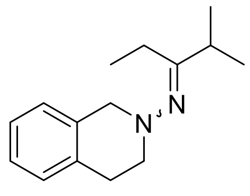

4ac

^{13}C NMR (125 MHz, $CDCl_3$)

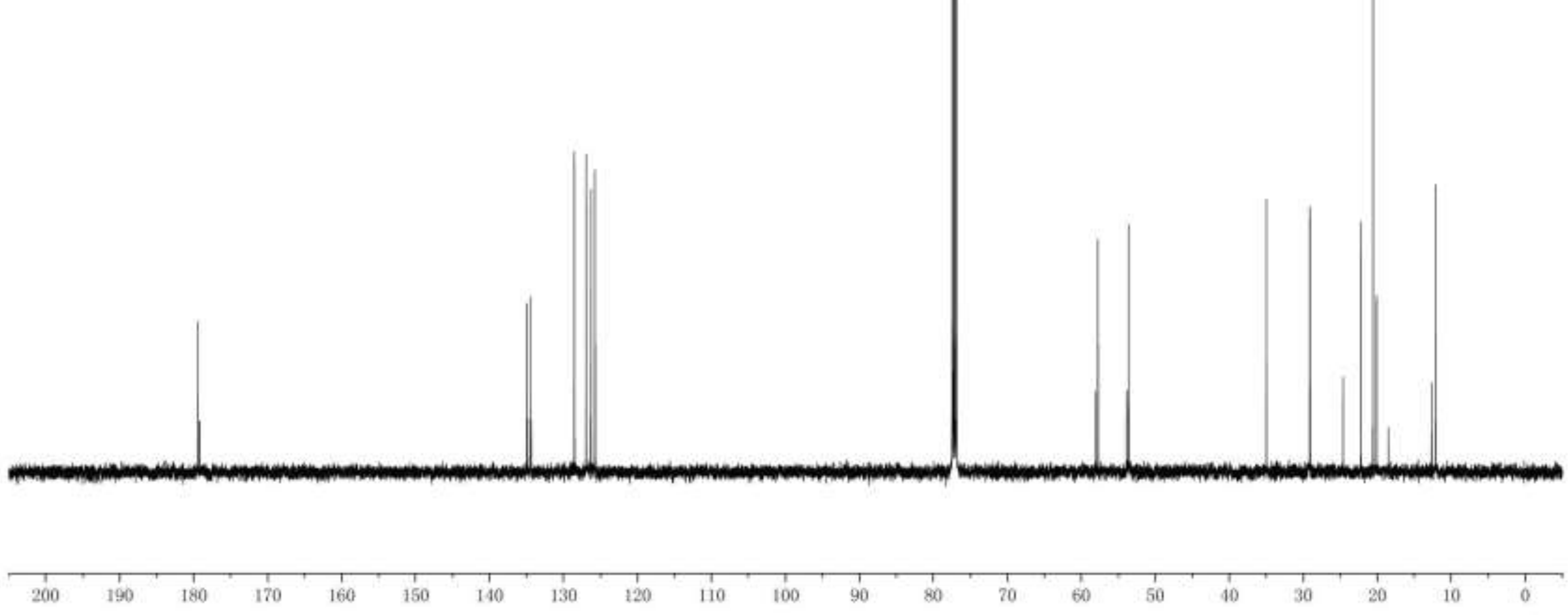

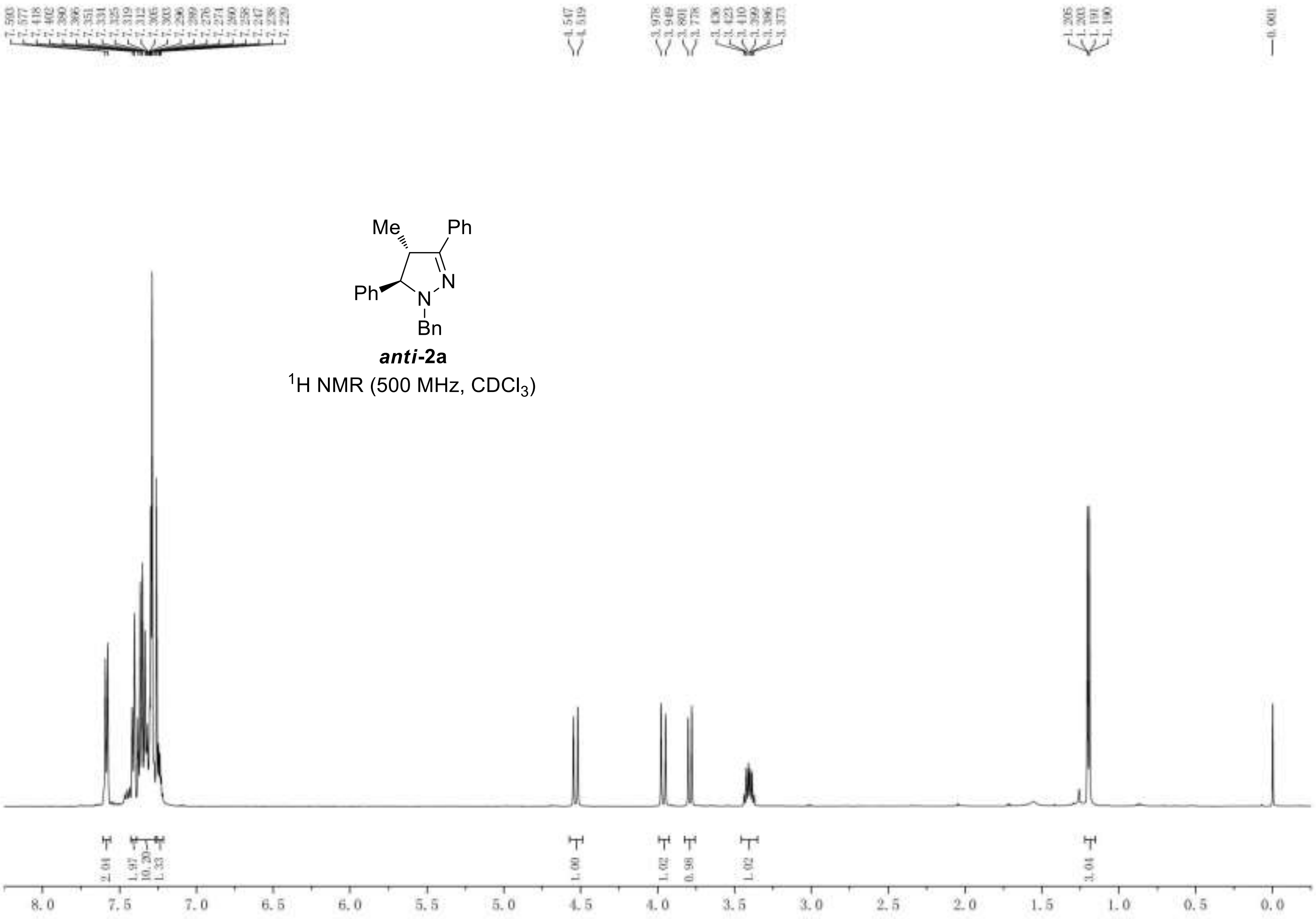

***anti*-2a**

^{1}H NMR (500 MHz, $CDCl_3$)

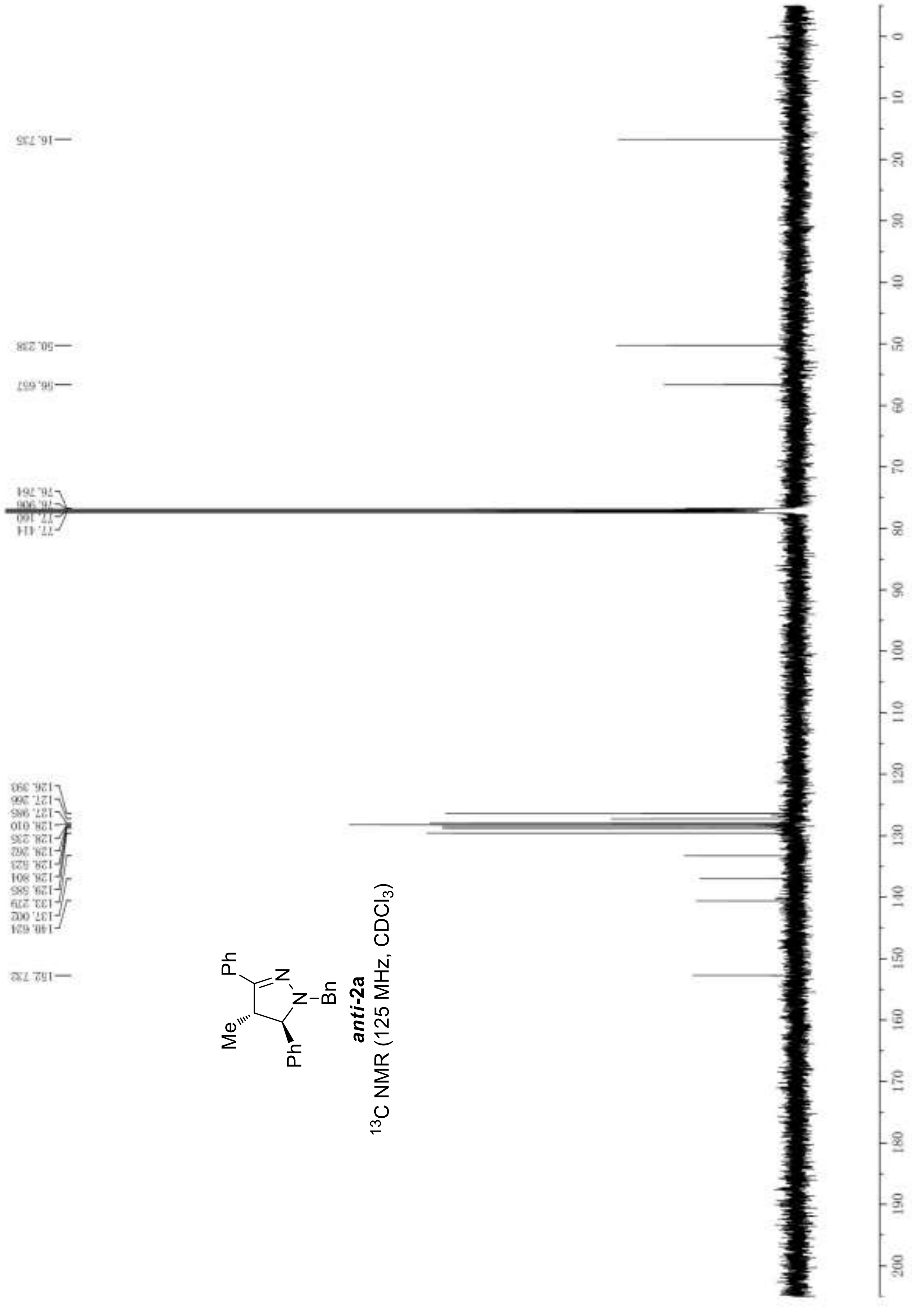

***anti*-2a**

^{13}C NMR (125 MHz, $CDCl_3$)

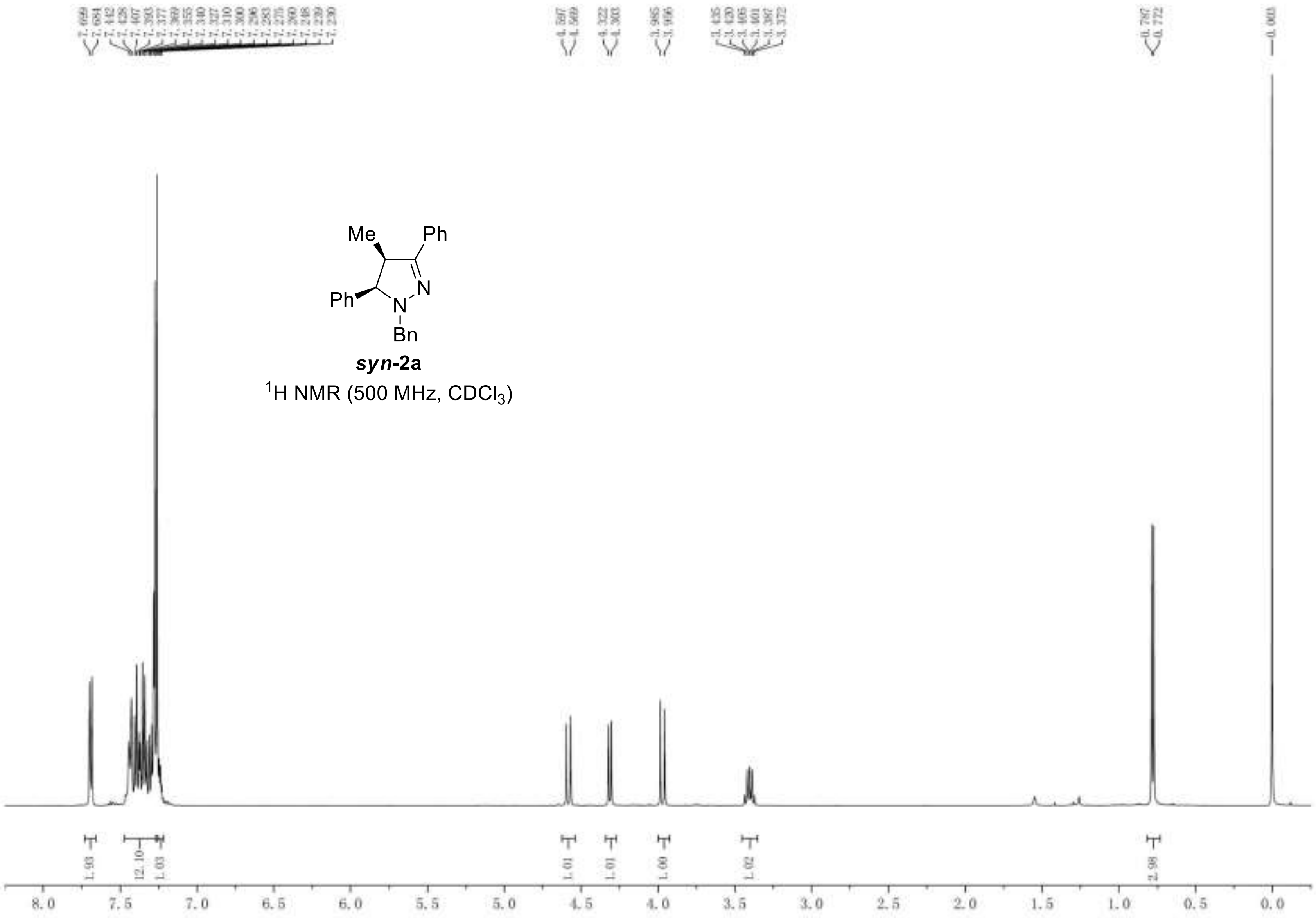

***syn*-2a**

^1H NMR (500 MHz, $CDCl_3$)

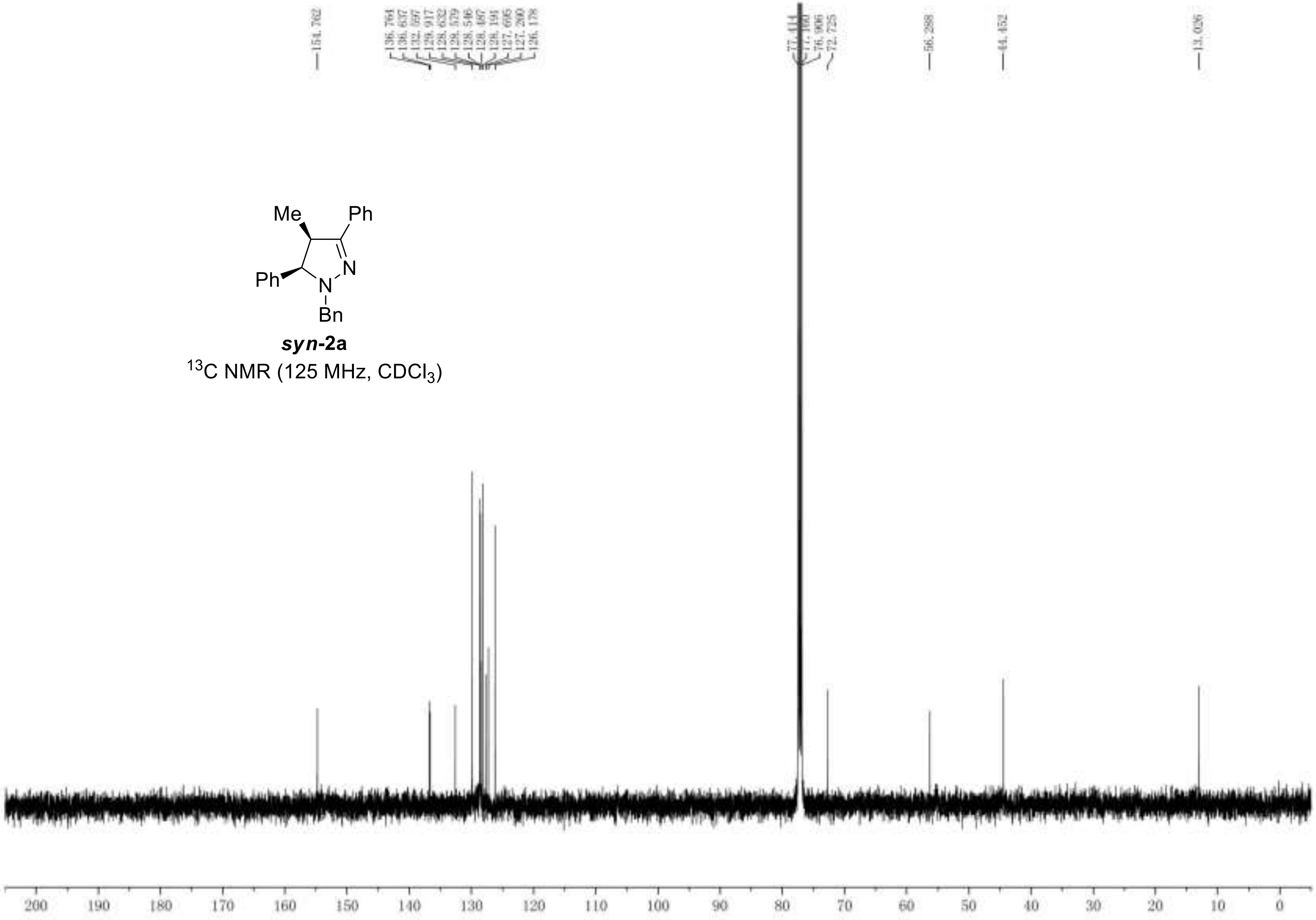
Me
Ph
Ph
N
N
Bn
syn-2a
13C NMR (125 MHz, CDCl3)
154.762
136.764
136.637
132.597
129.917
128.632
128.579
128.546
128.487
128.191
127.695
127.290
126.178
77.414
77.160
76.906
72.725
56.288
44.452
13.026
200
190
180
170
160
150
140
130
120
110
100
90
80
70
60
50
40
30
20
10
0

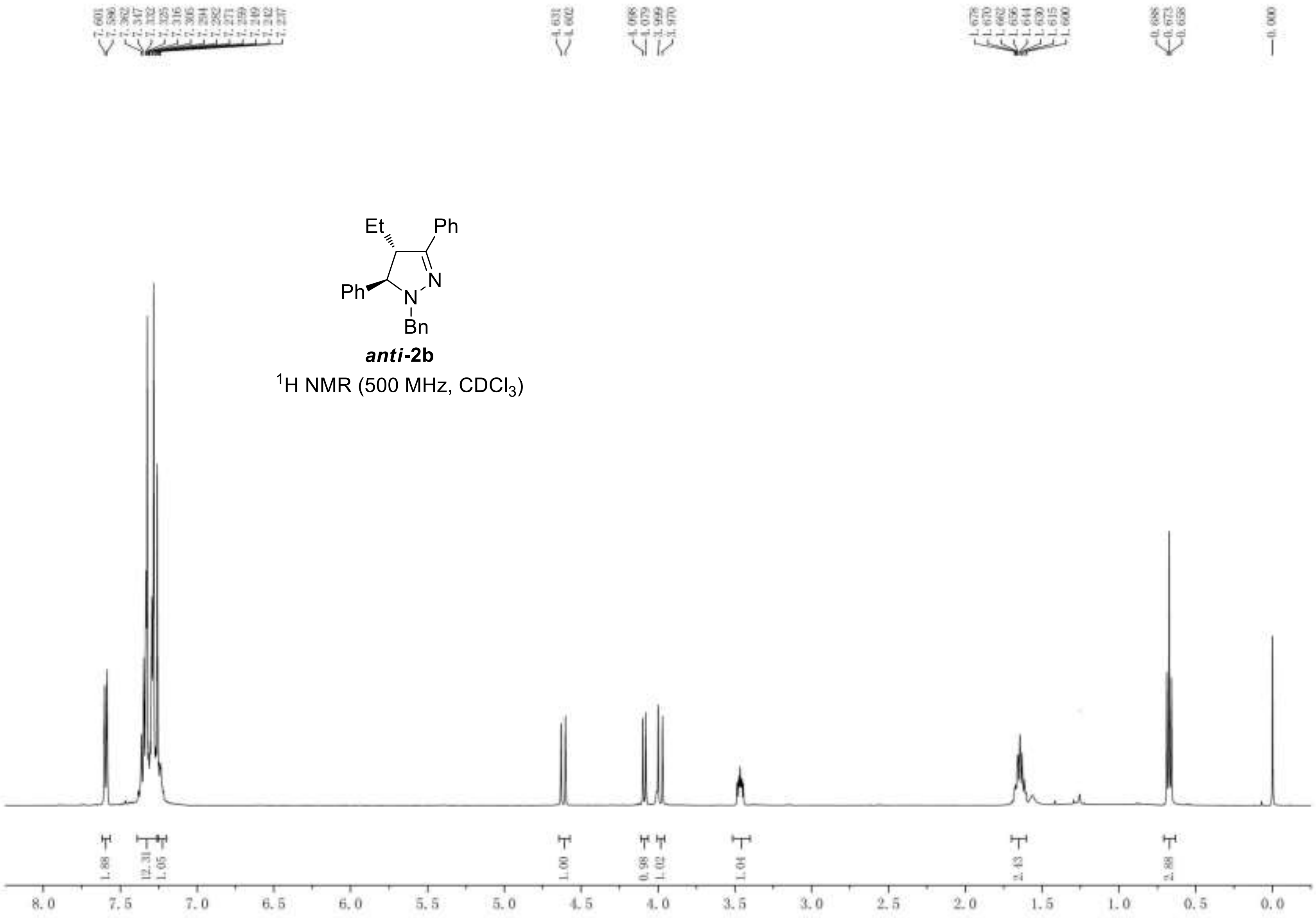

***anti*-2b**

^1H NMR (500 MHz, $CDCl_3$)

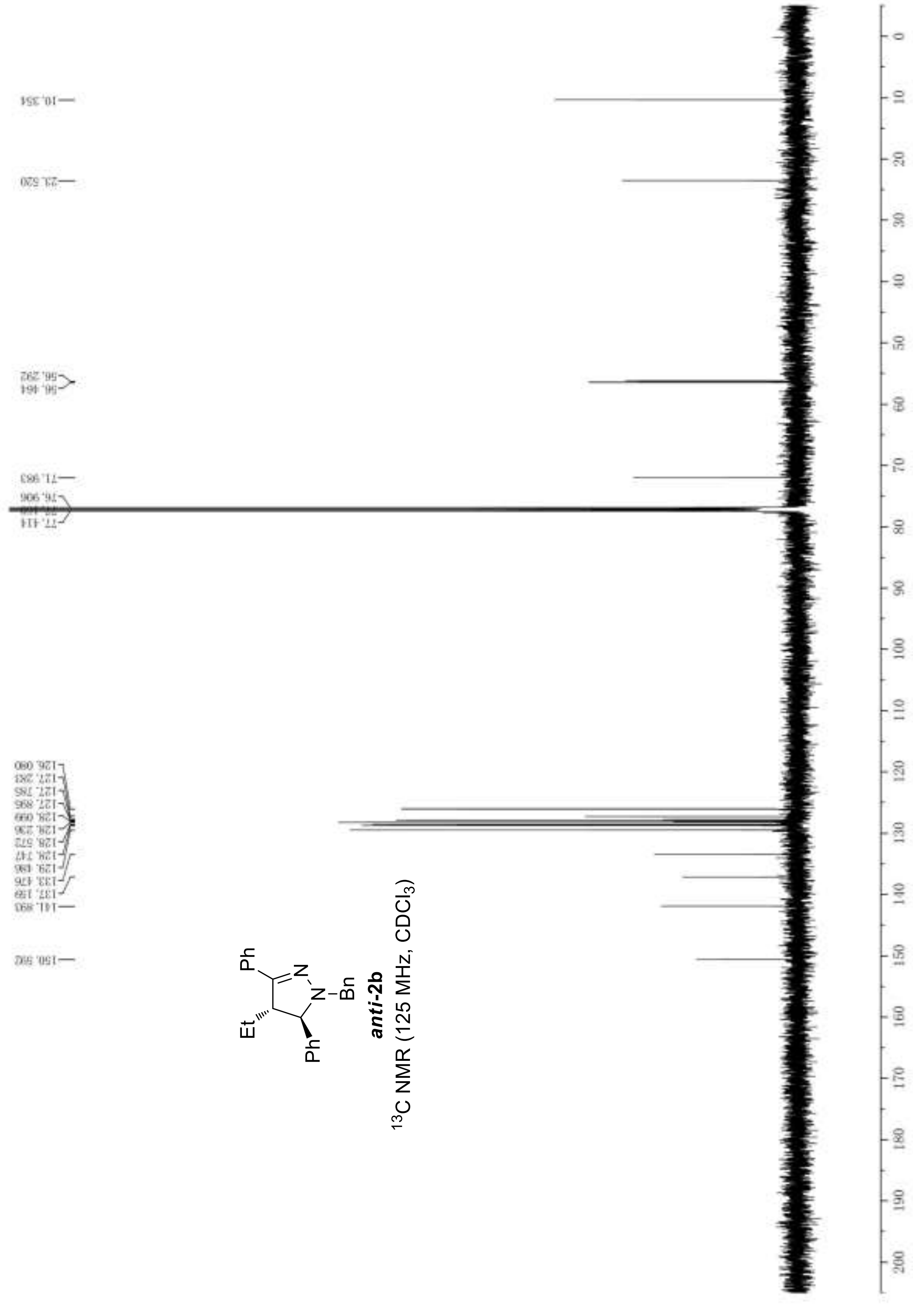

***anti*-2b**

^{13}C NMR (125 MHz, $CDCl_3$)

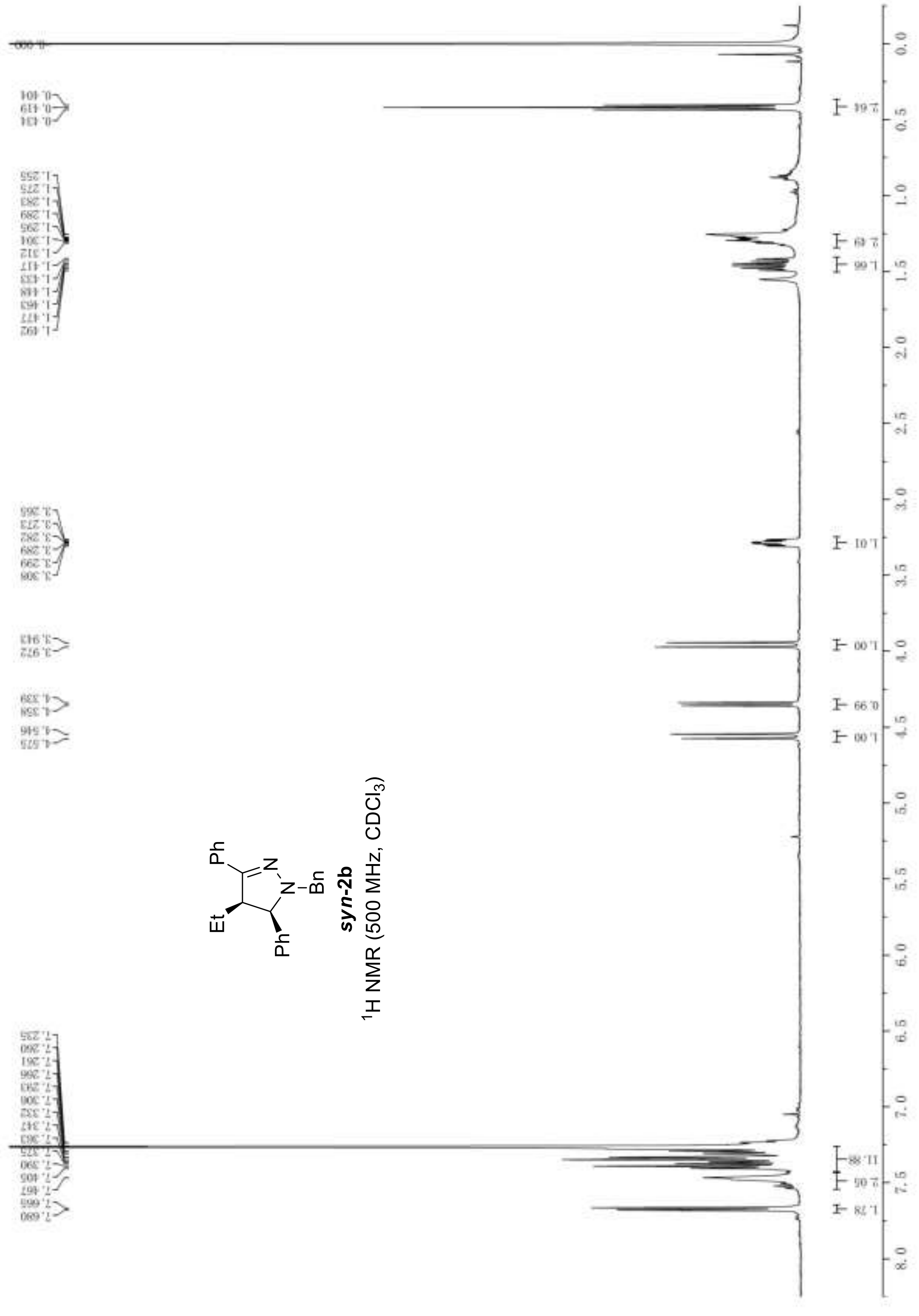

***syn*-2b**

^{1}H NMR (500 MHz, $CDCl_3$)

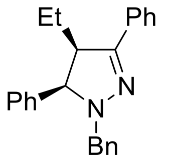

syn-**2b**

^{13}C NMR (125 MHz, $CDCl_3$)

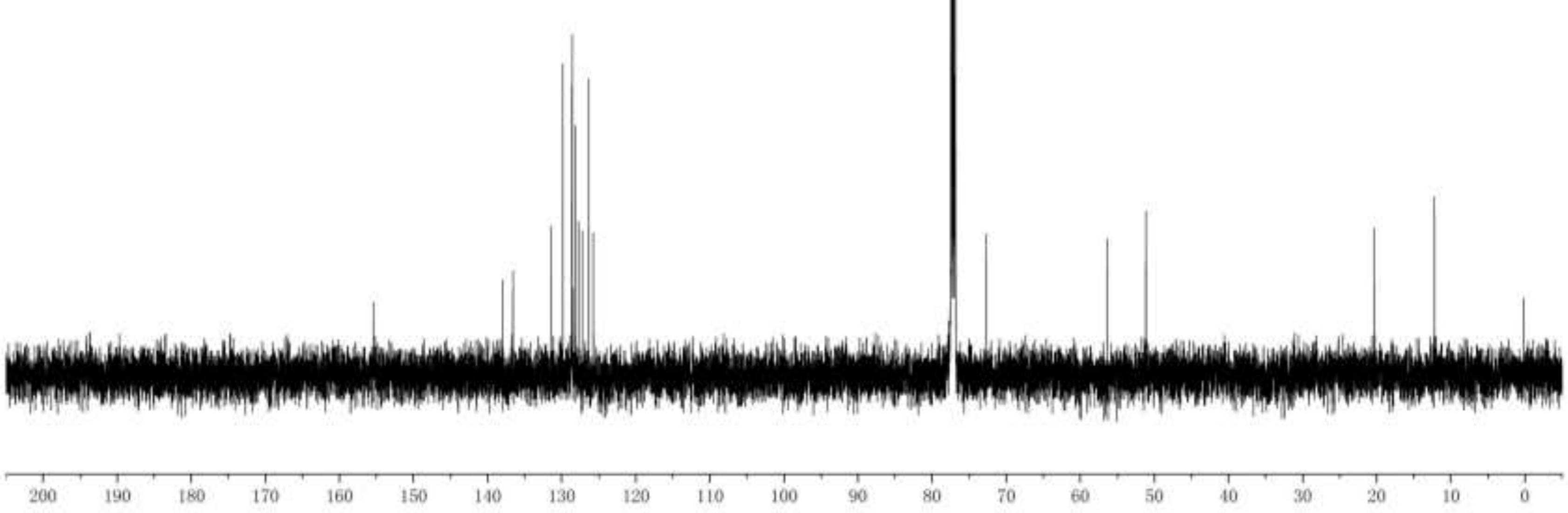

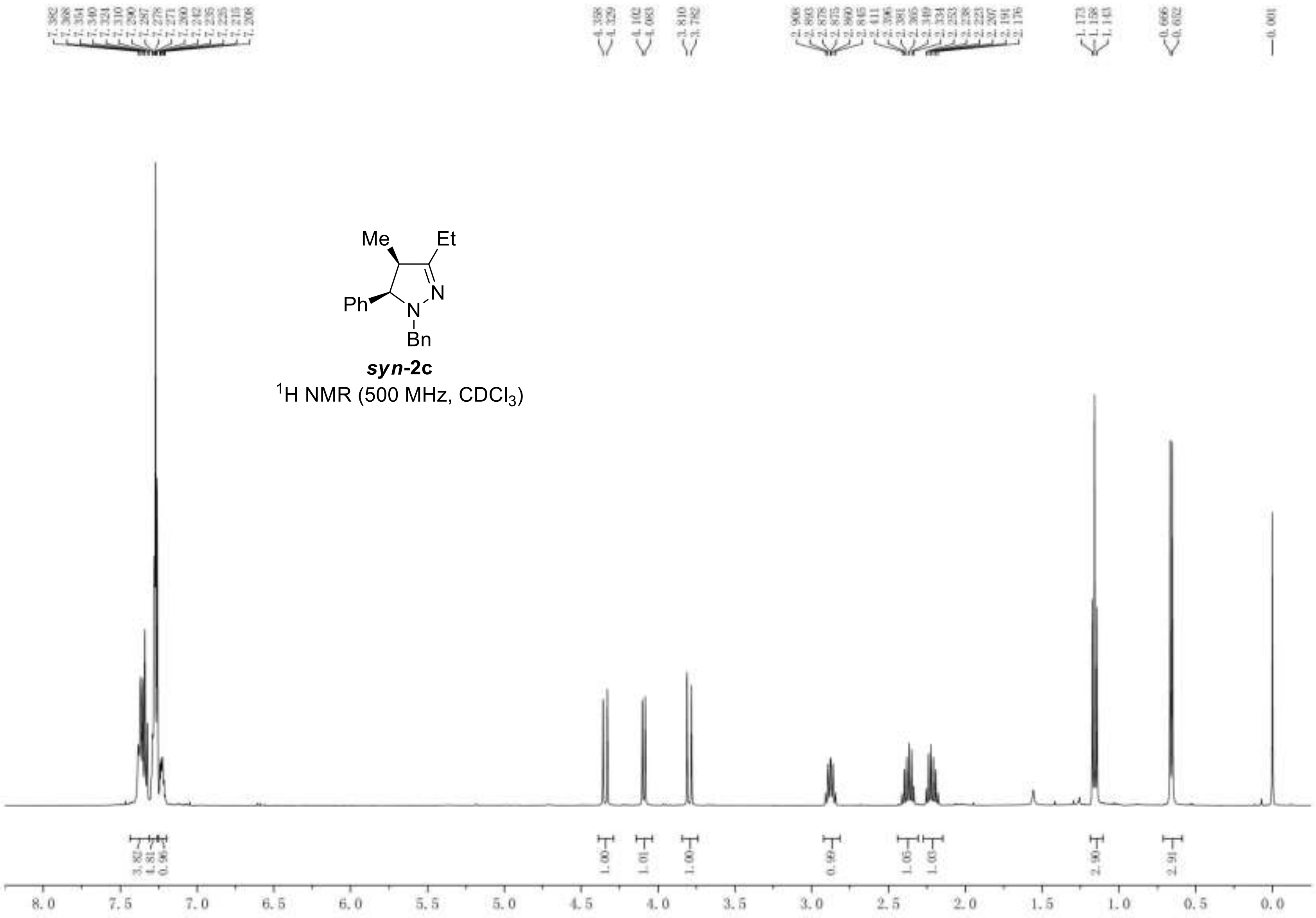

***syn*-2c**

^{1}H NMR (500 MHz, $CDCl_3$)

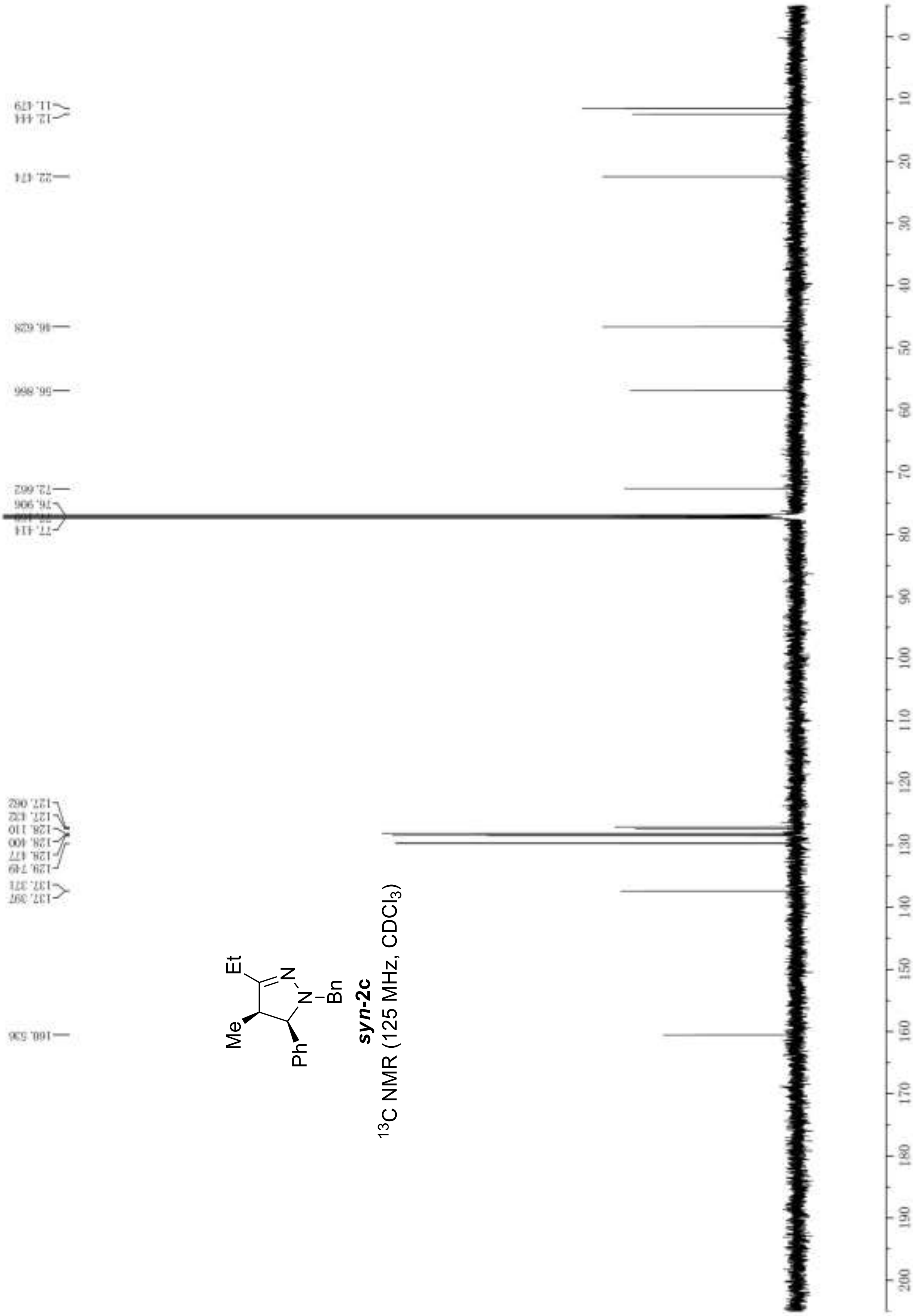

***syn*-2c**

^{13}C NMR (125 MHz, $CDCl_3$)

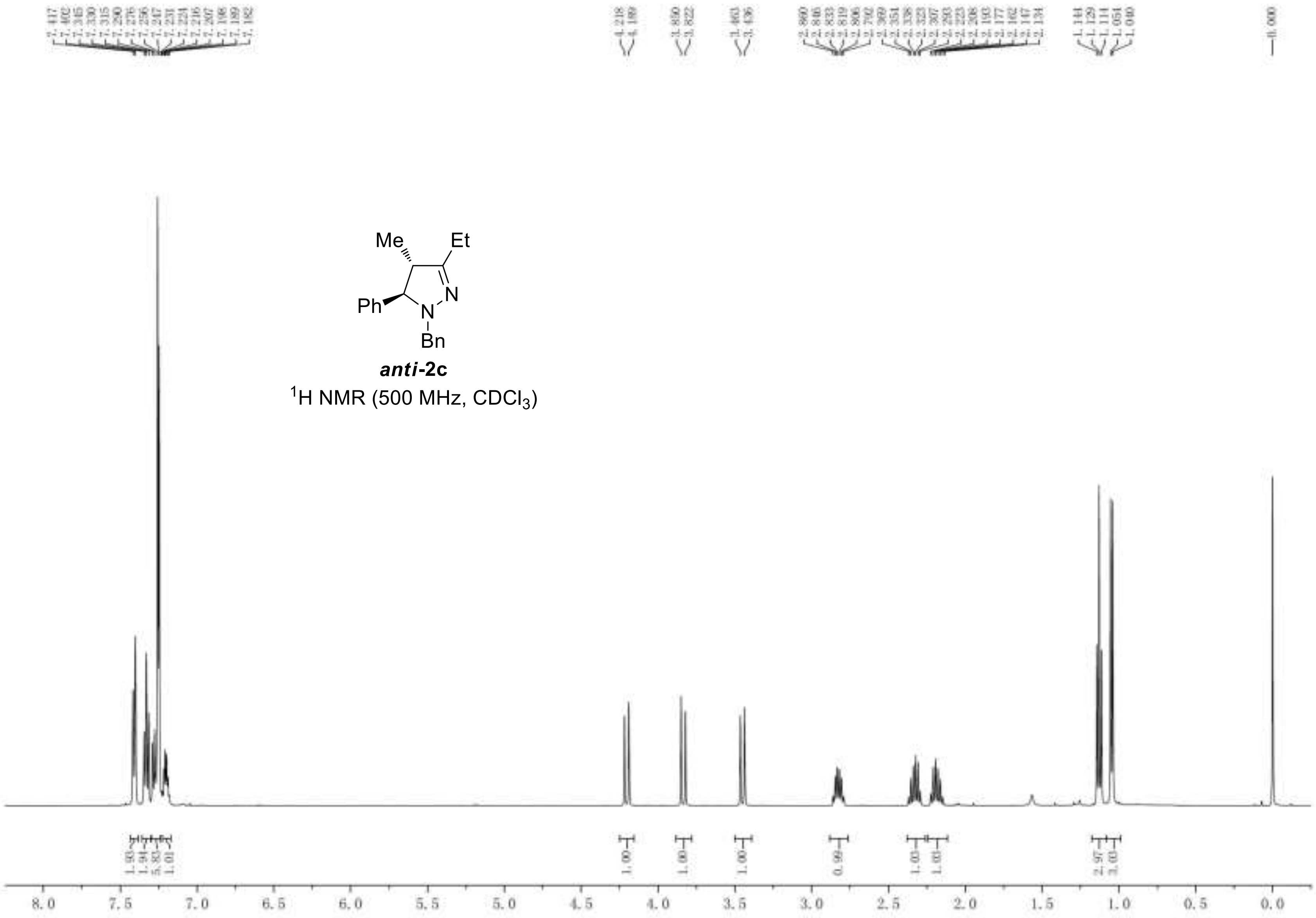

***anti*-2c**

^1H NMR (500 MHz, $CDCl_3$)

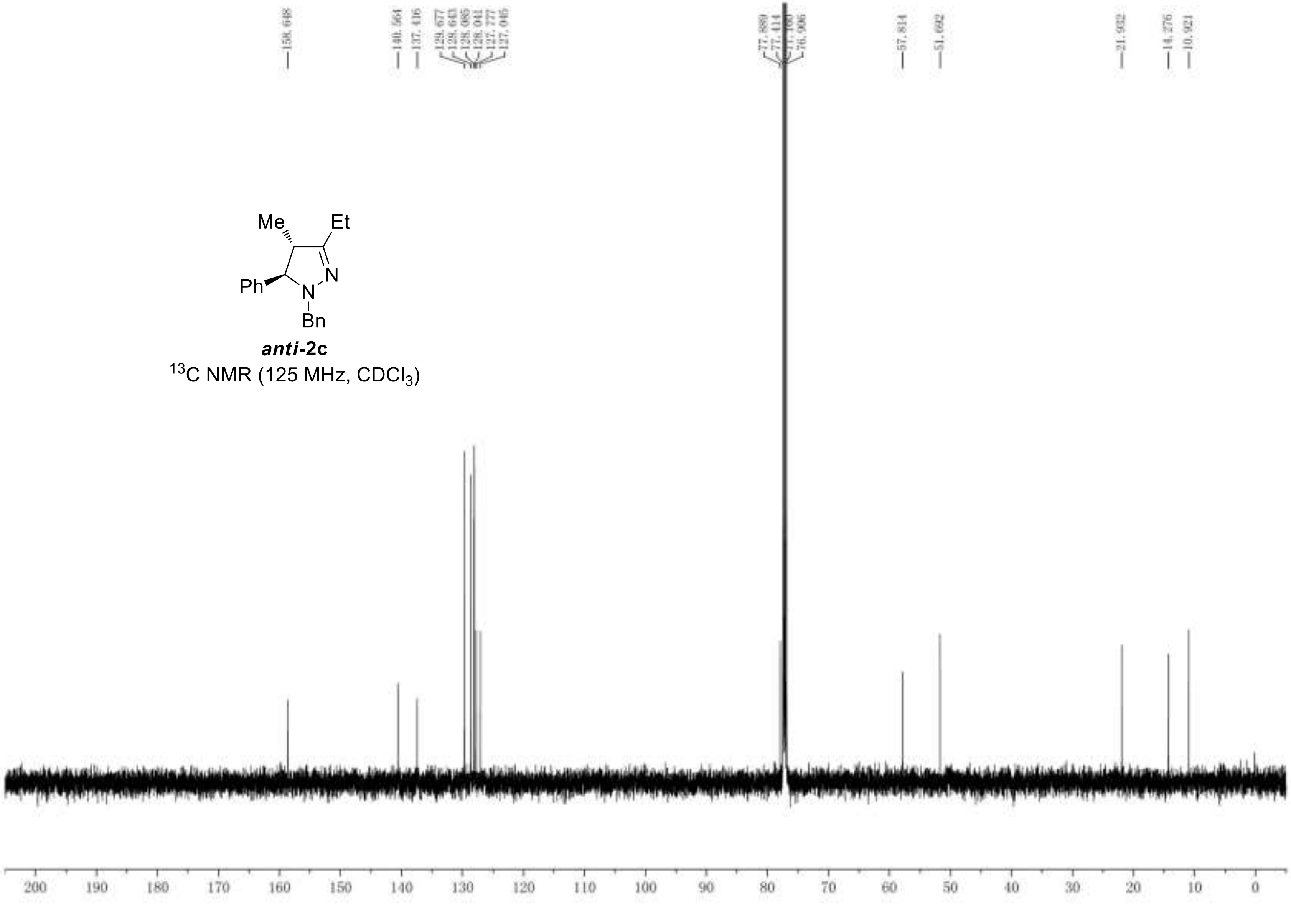

***anti*-2c**

^{13}C NMR (125 MHz, $CDCl_3$)

syn-**2d**

^{1}H NMR (500 MHz, $CDCl_3$)

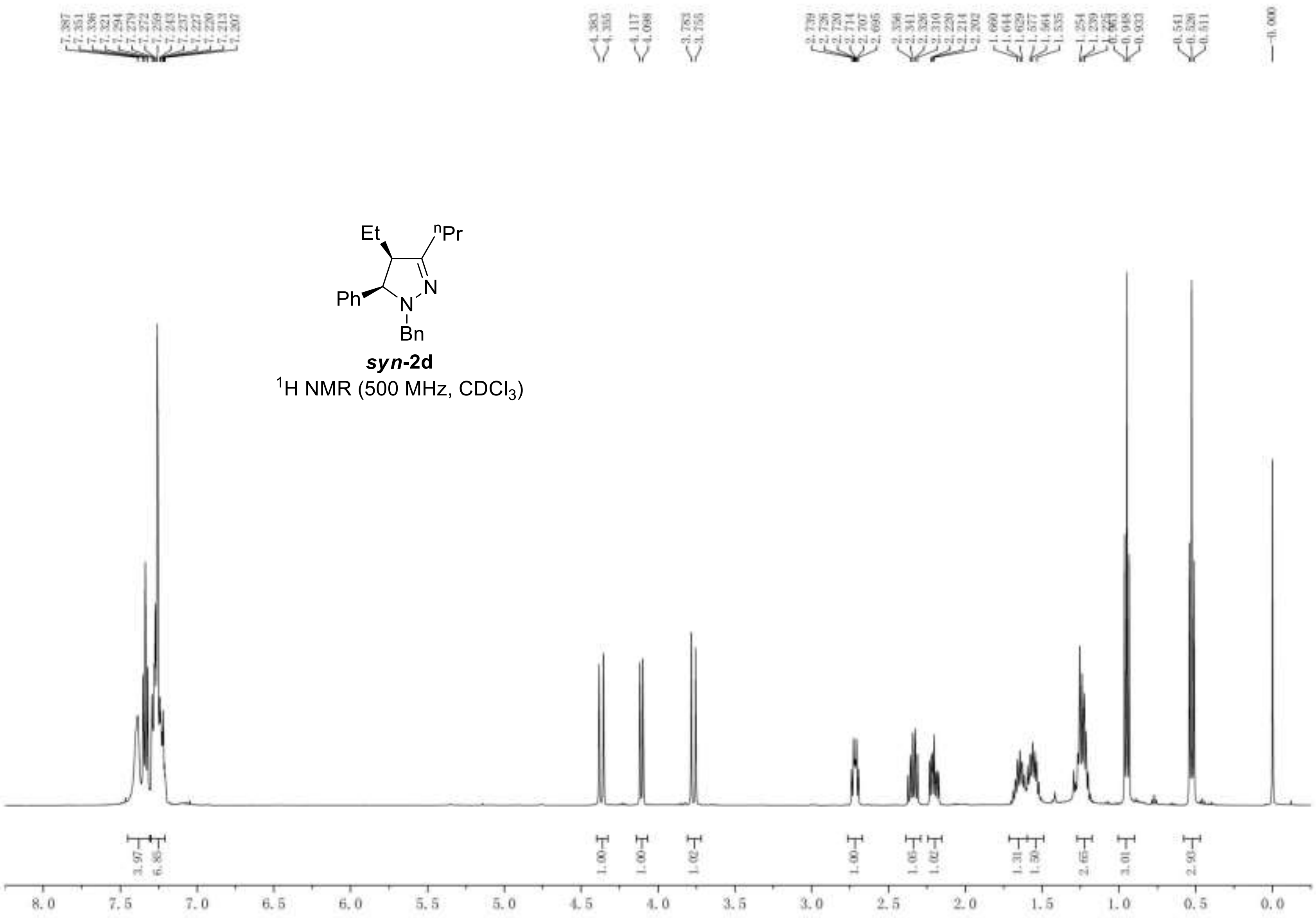

***syn*-2d**

^{13}C NMR (125 MHz, $CDCl_3$)

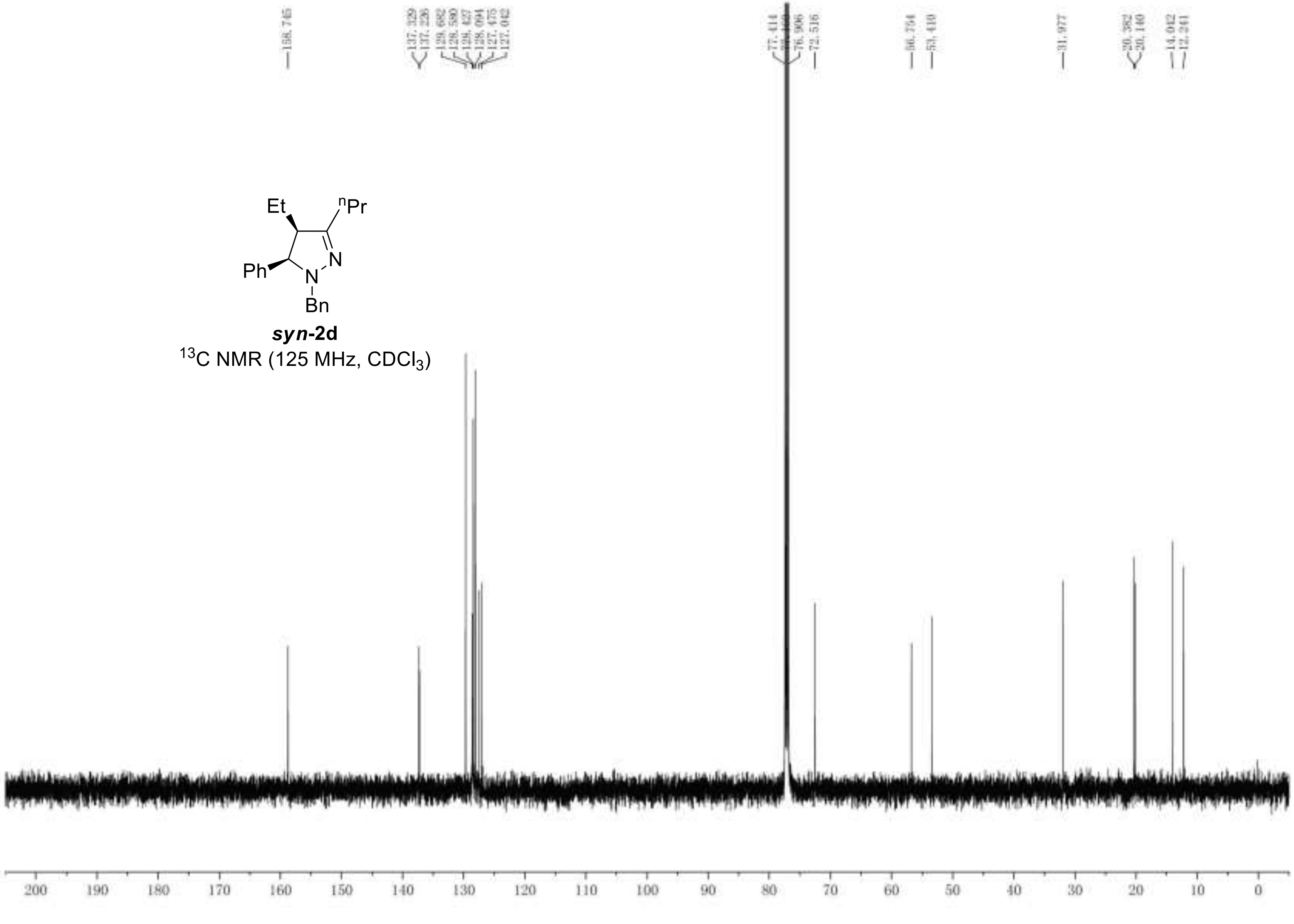

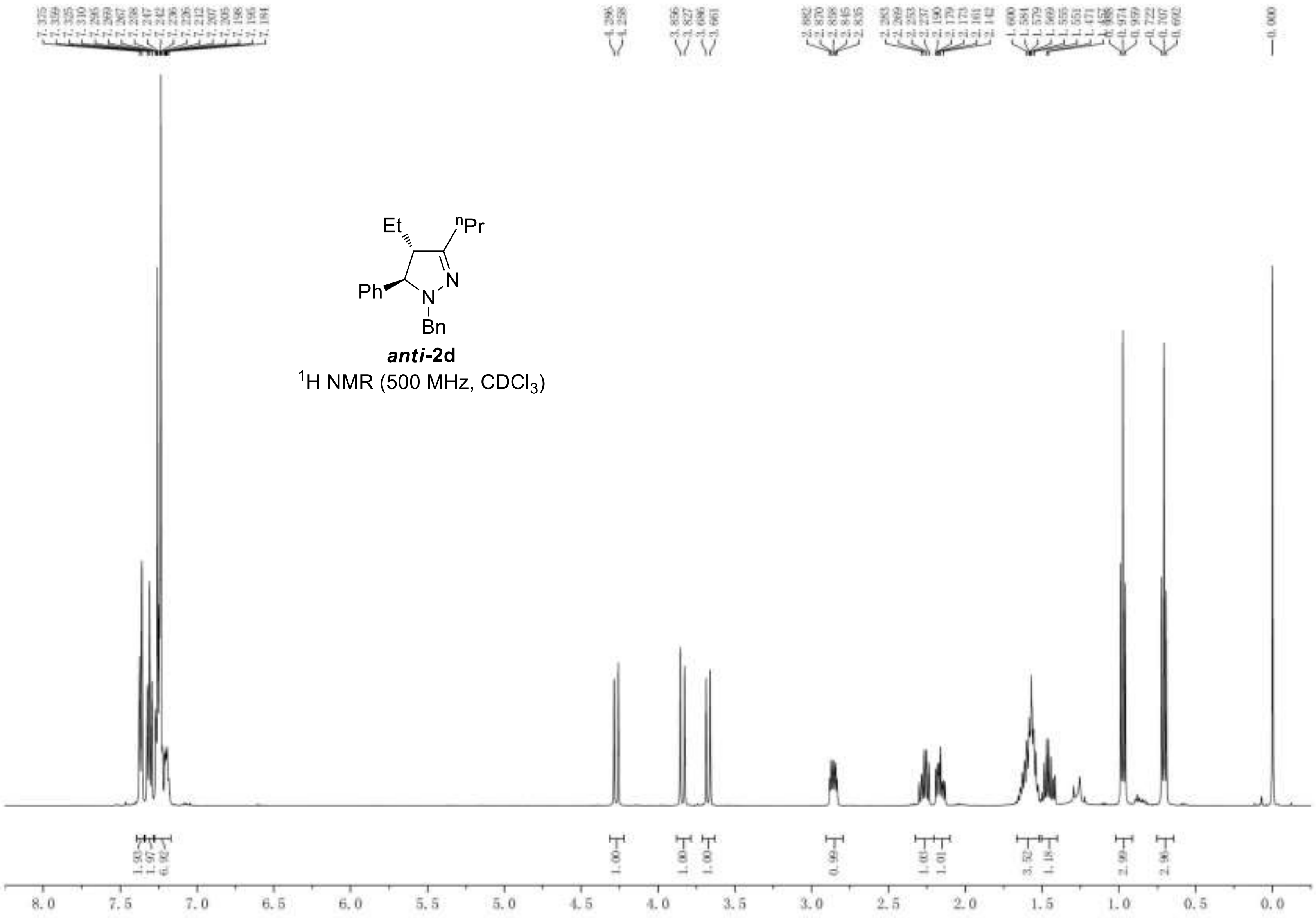

***anti*-2d**

^{1}H NMR (500 MHz, $CDCl_3$)

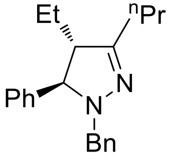

***anti*-2d**

^{13}C NMR (125 MHz, $CDCl_3$)

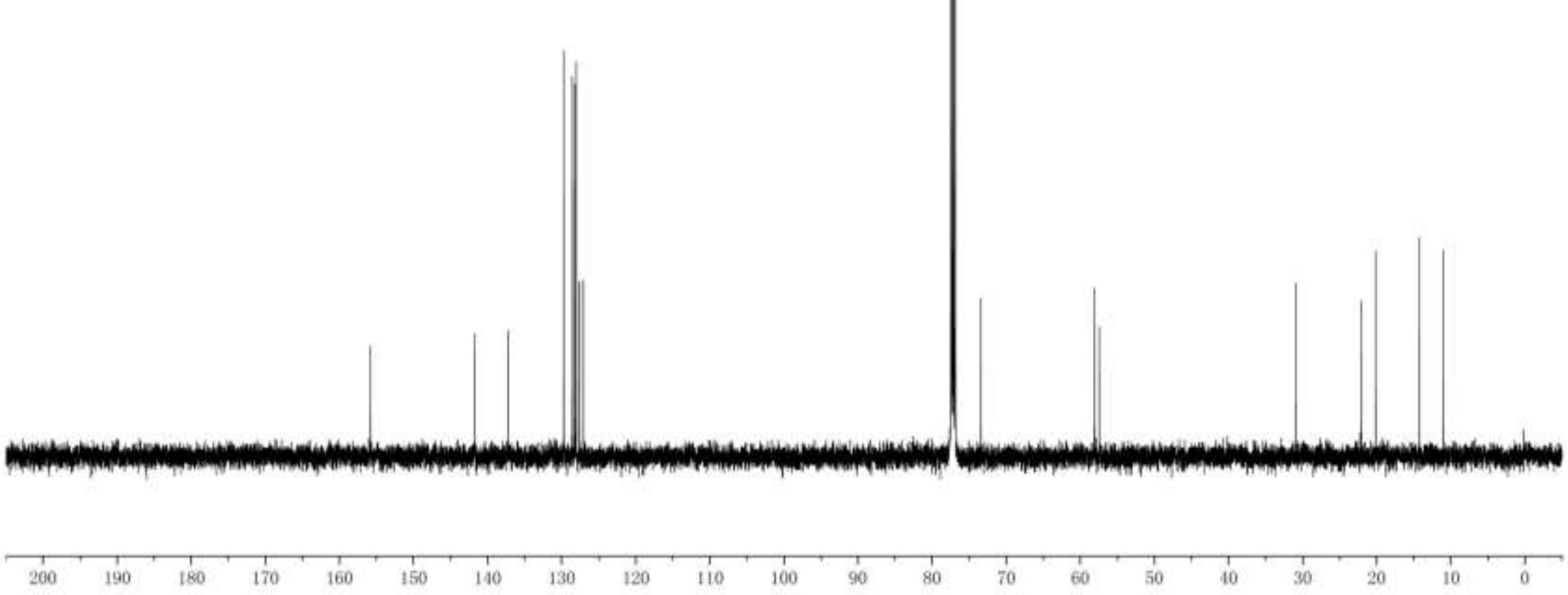

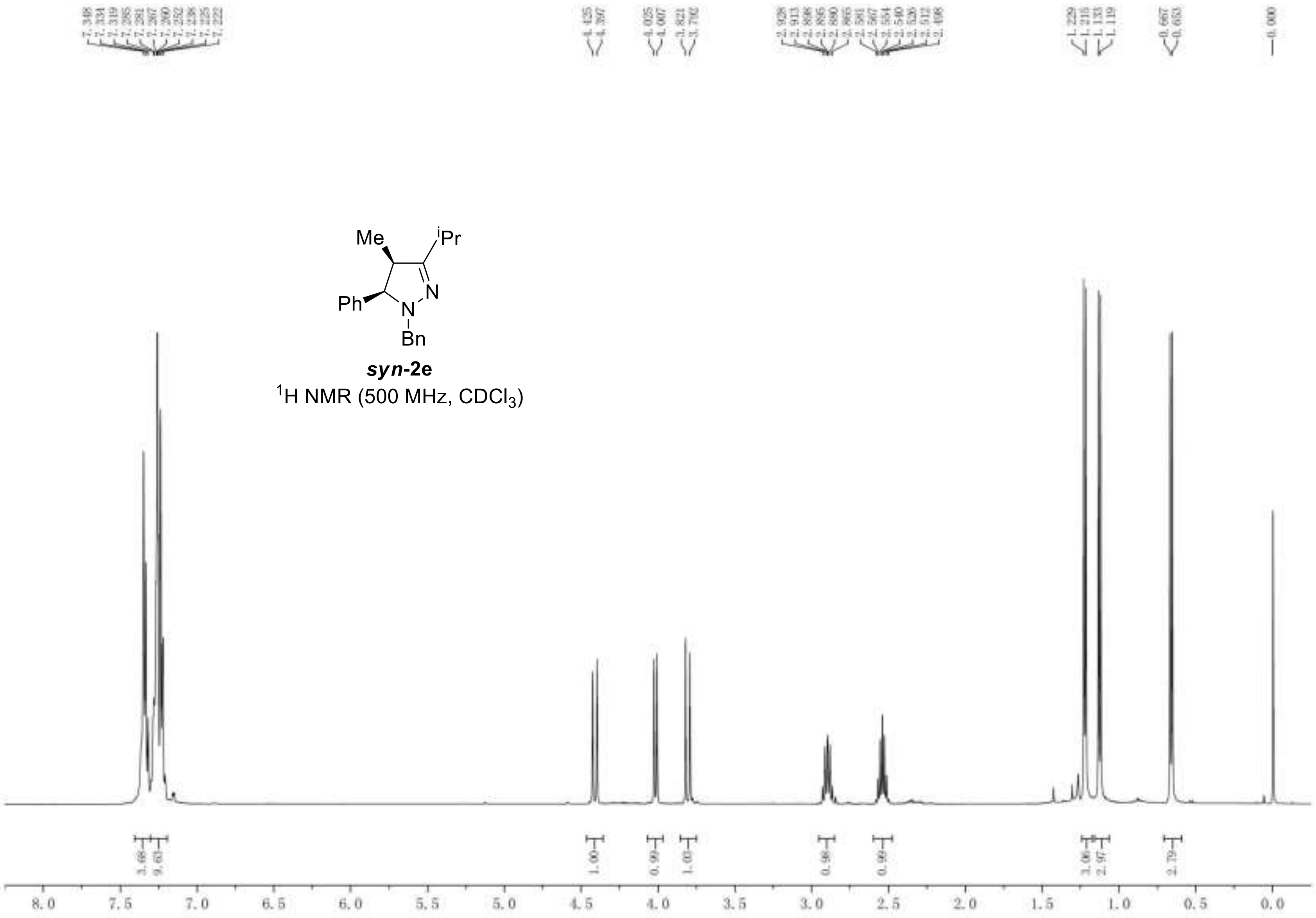

***syn*-2e**

^{1}H NMR (500 MHz, $CDCl_3$)

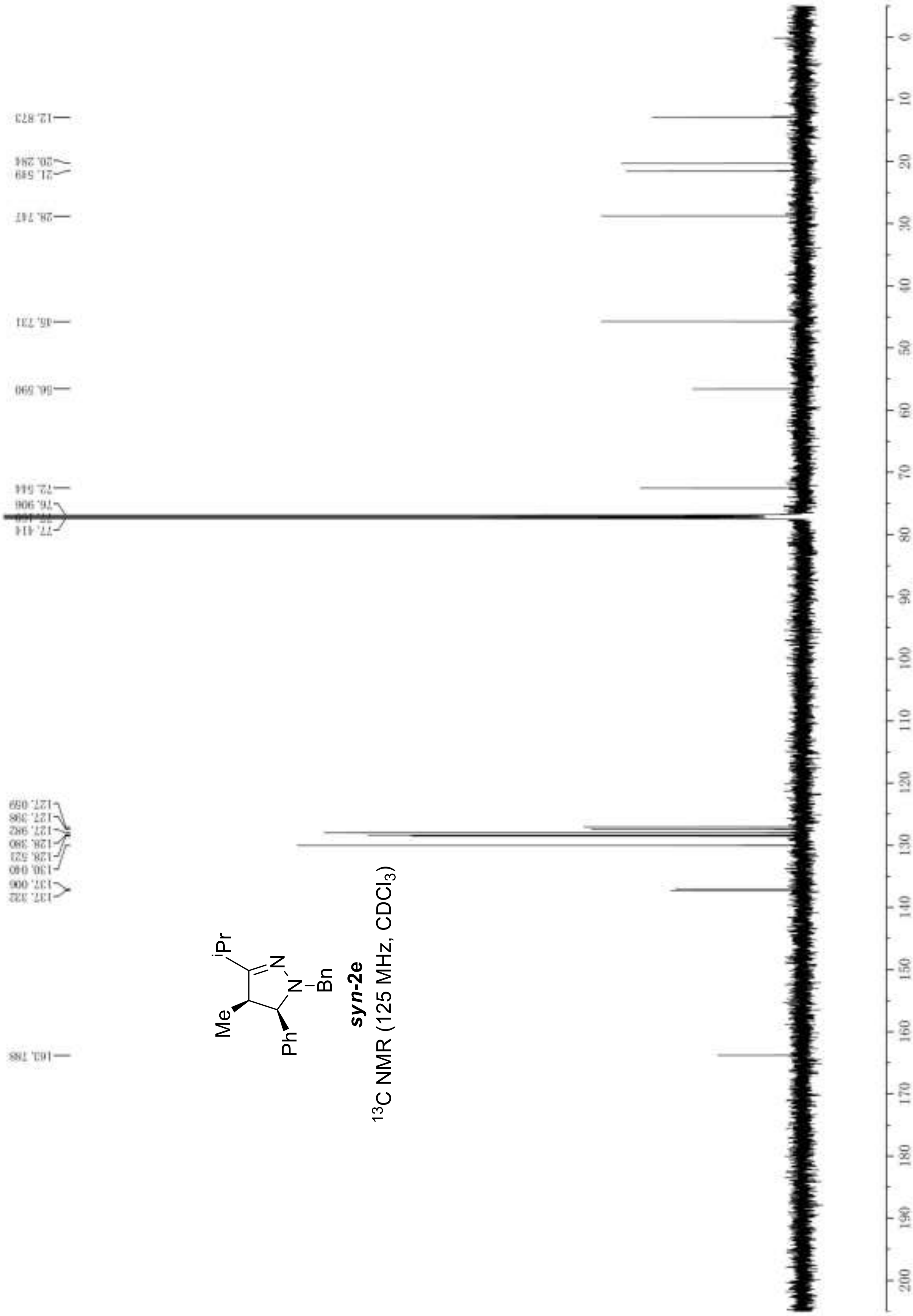

163.788
137.332
137.006
130.040
128.521
128.380
127.982
127.398
127.059
77.414
77.160
76.906
72.544
56.590
45.731
28.747
21.549
20.284
12.873
iPr
N
N
Bn
Me
Ph
syn-2e
^{13}C NMR (125 MHz, $CDCl_3$)

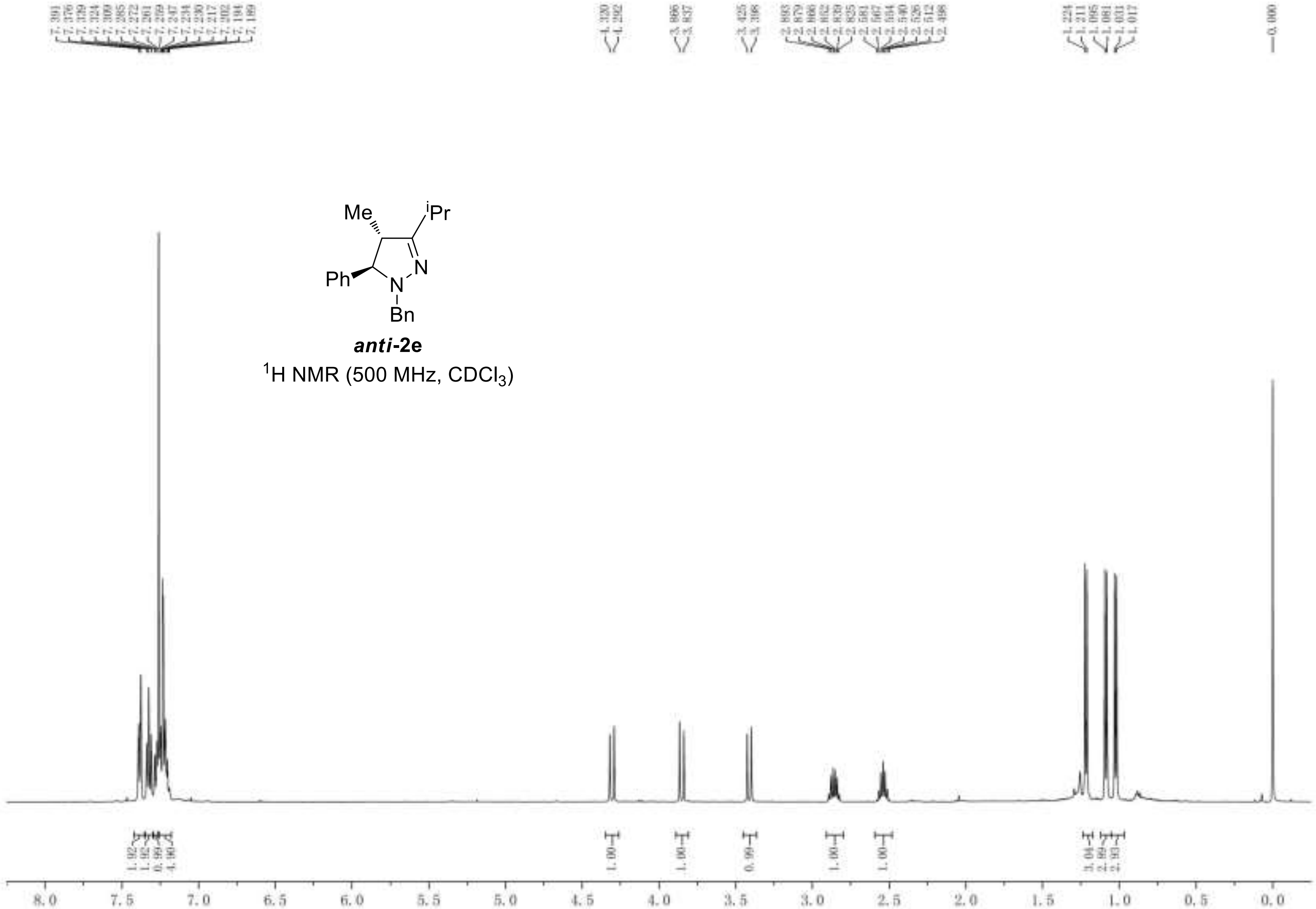

***anti*-2e**

^{1}H NMR (500 MHz, $CDCl_3$)

***anti*-2e**

^{13}C NMR (125 MHz, $CDCl_3$)

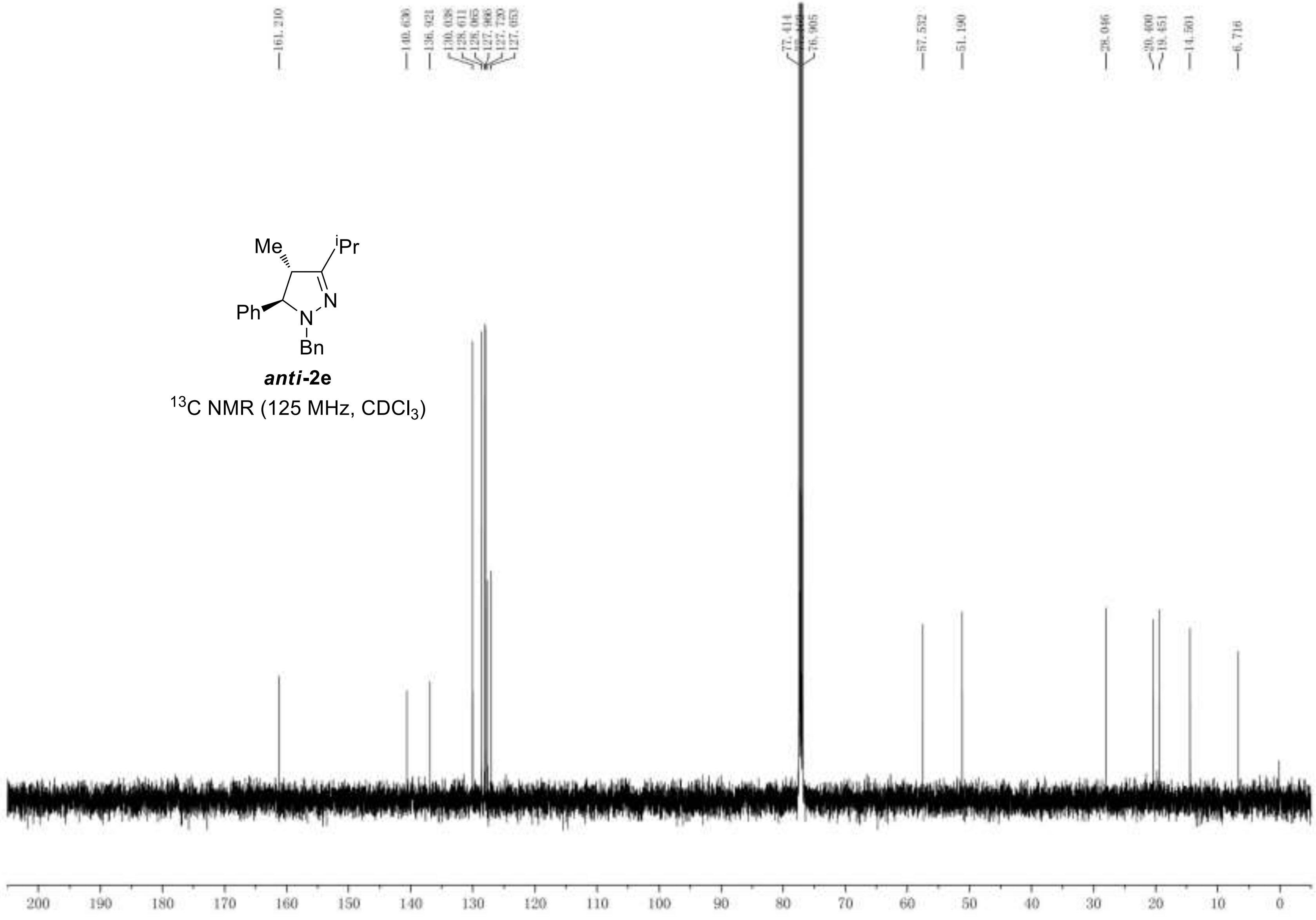

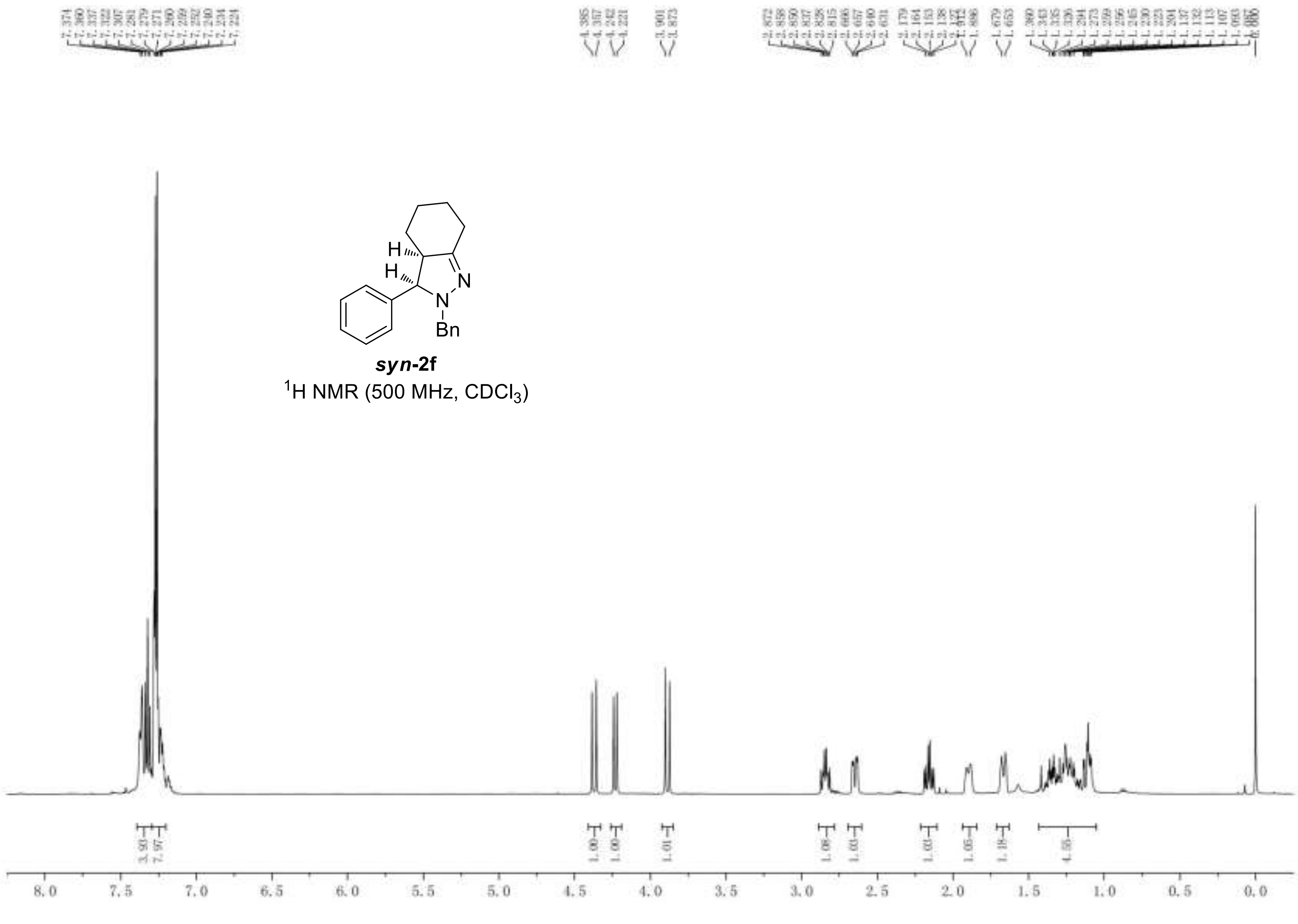

***syn*-2f**

^{1}H NMR (500 MHz, $CDCl_3$)

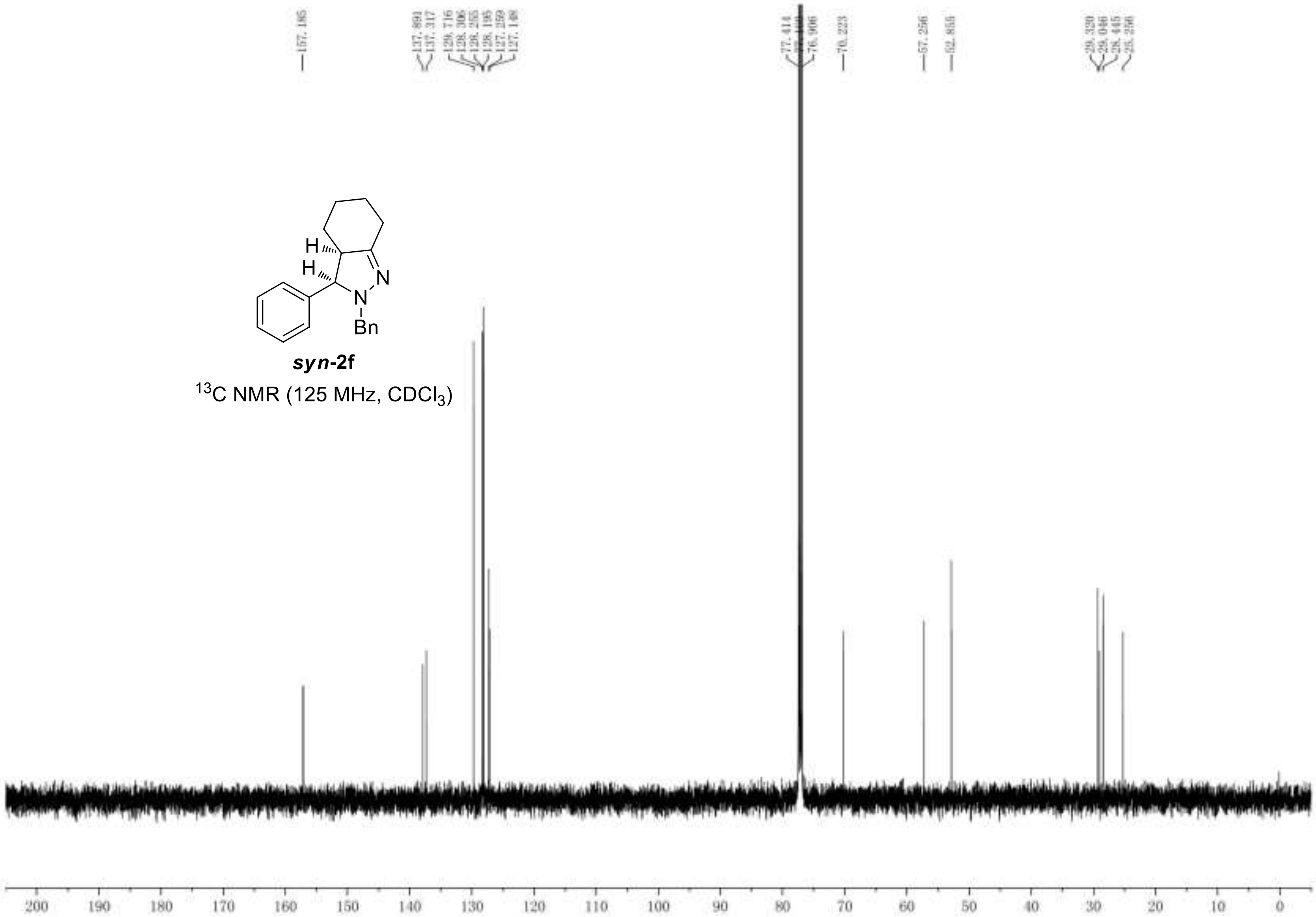

***syn*-2f**

^{13}C NMR (125 MHz, $CDCl_3$)

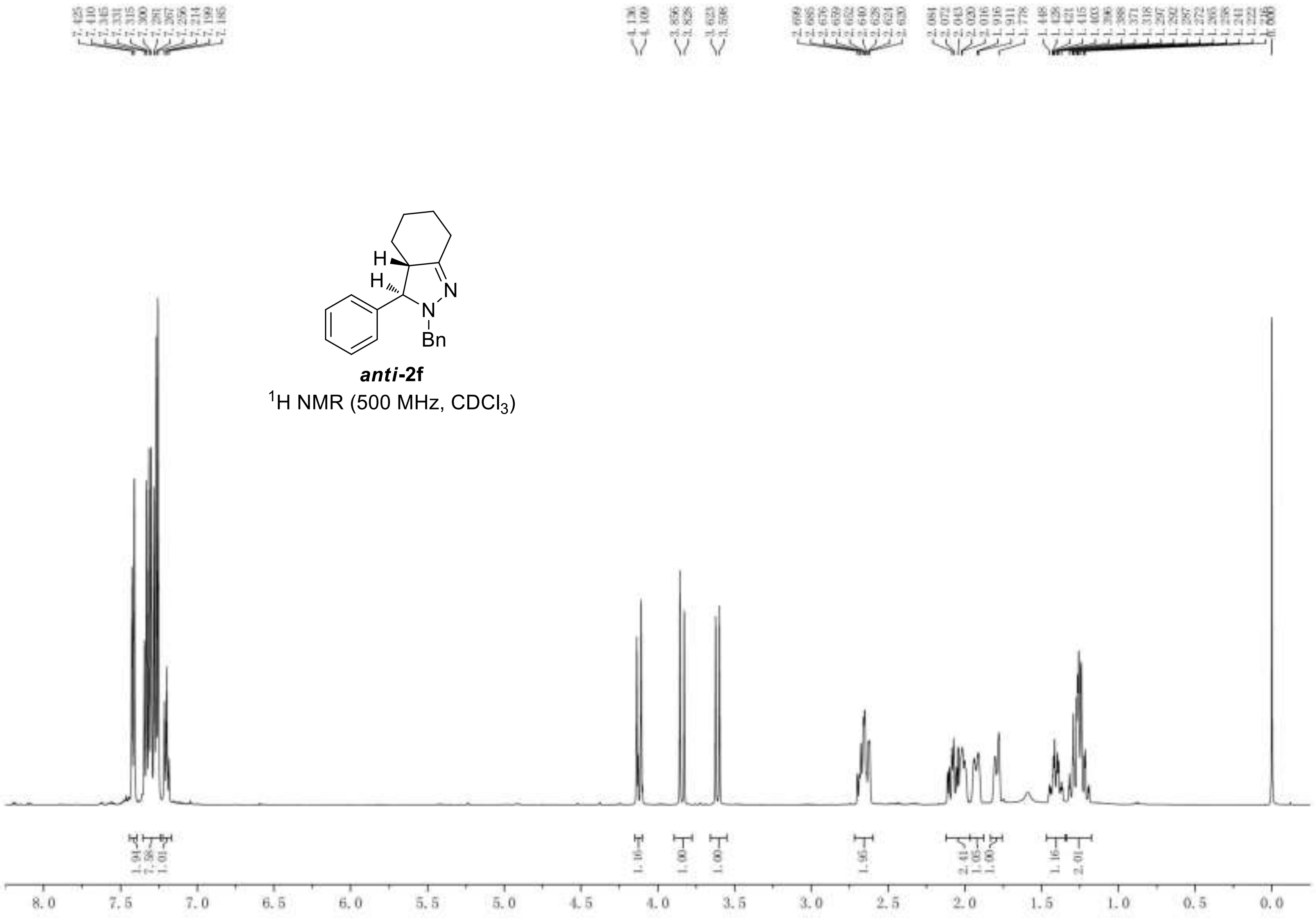

***anti*-2f**

^{1}H NMR (500 MHz, $CDCl_3$)

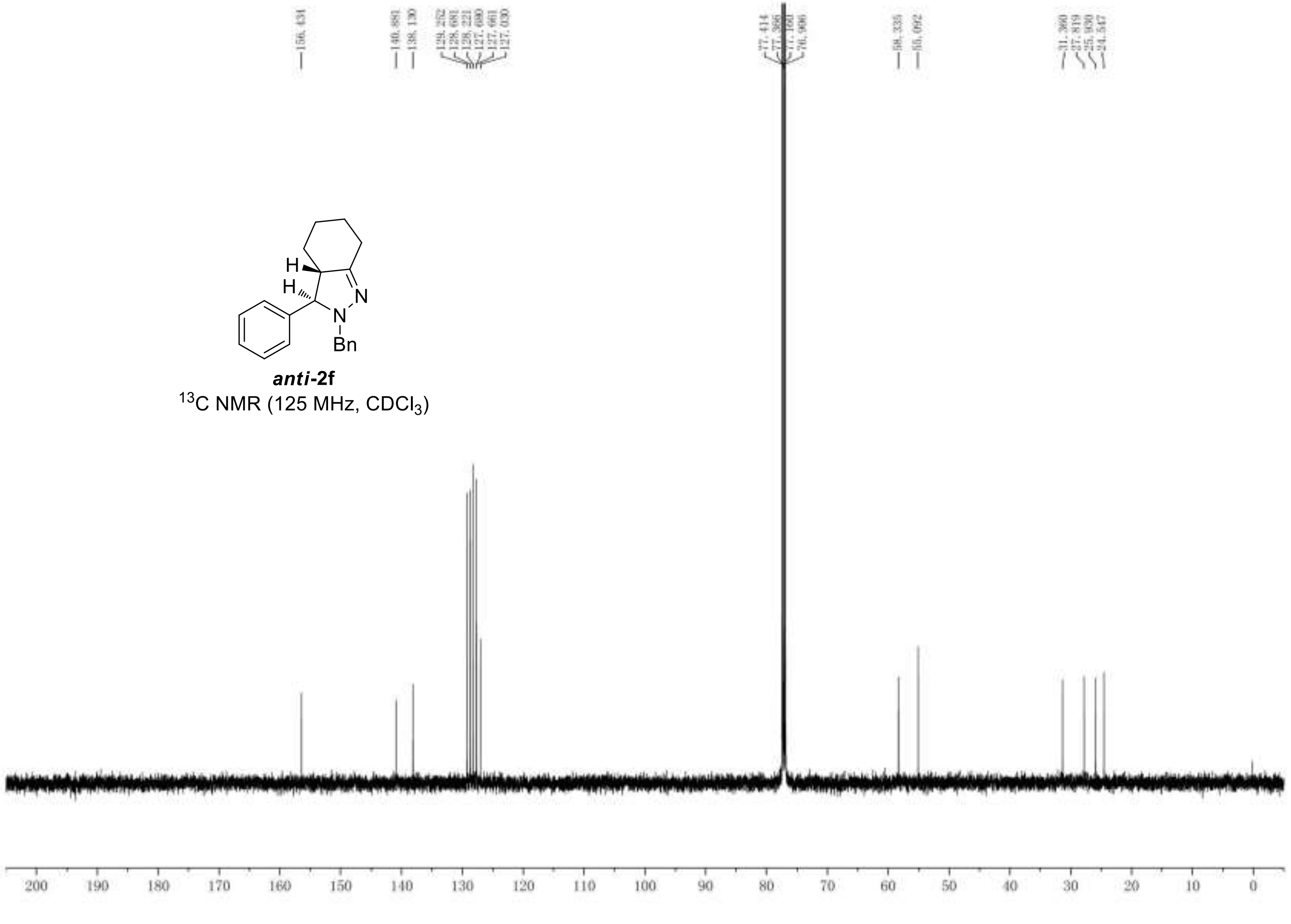

***anti*-2f**

^{13}C NMR (125 MHz, $CDCl_3$)

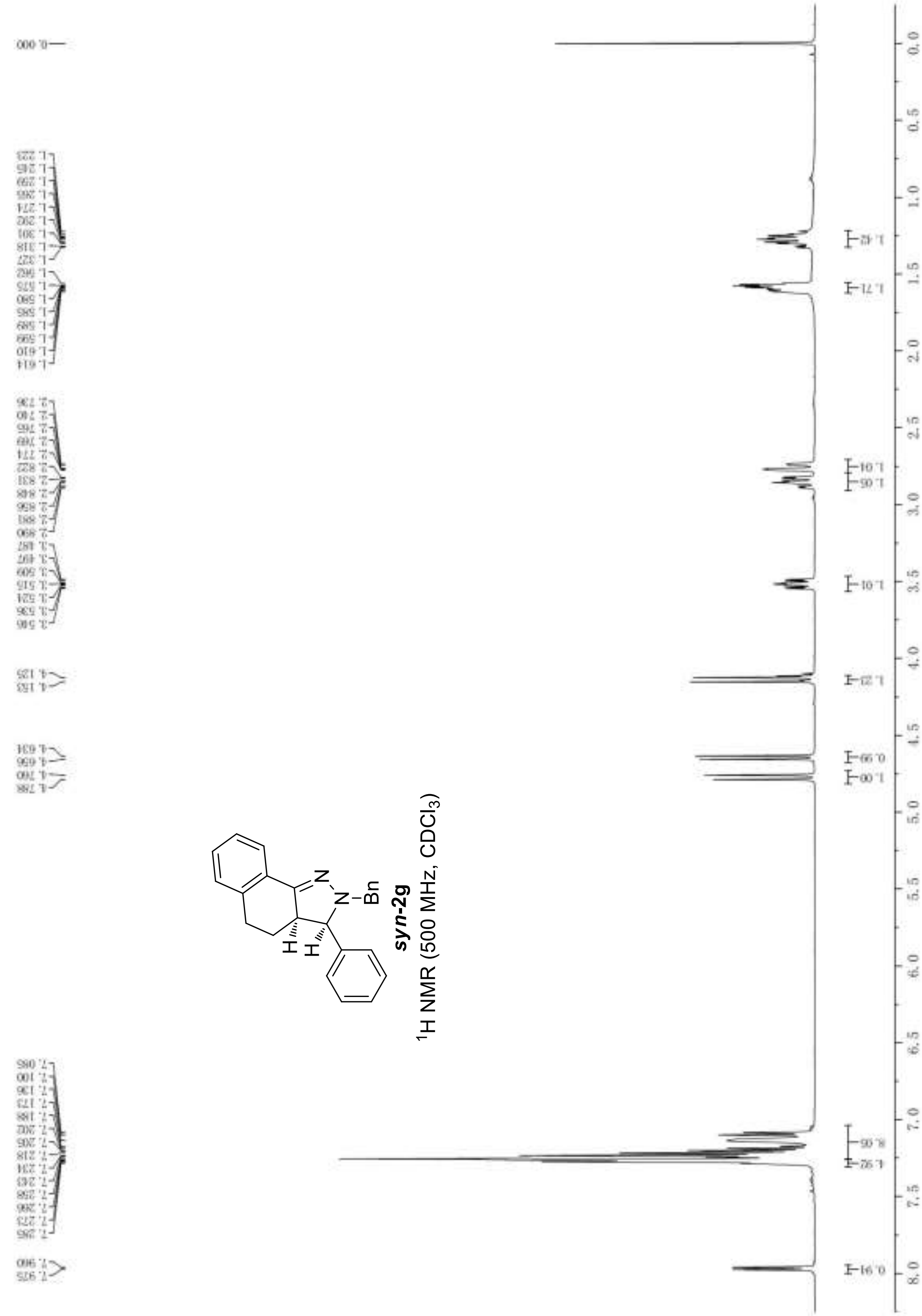

***syn*-2g**
^1H NMR (500 MHz, $CDCl_3$)

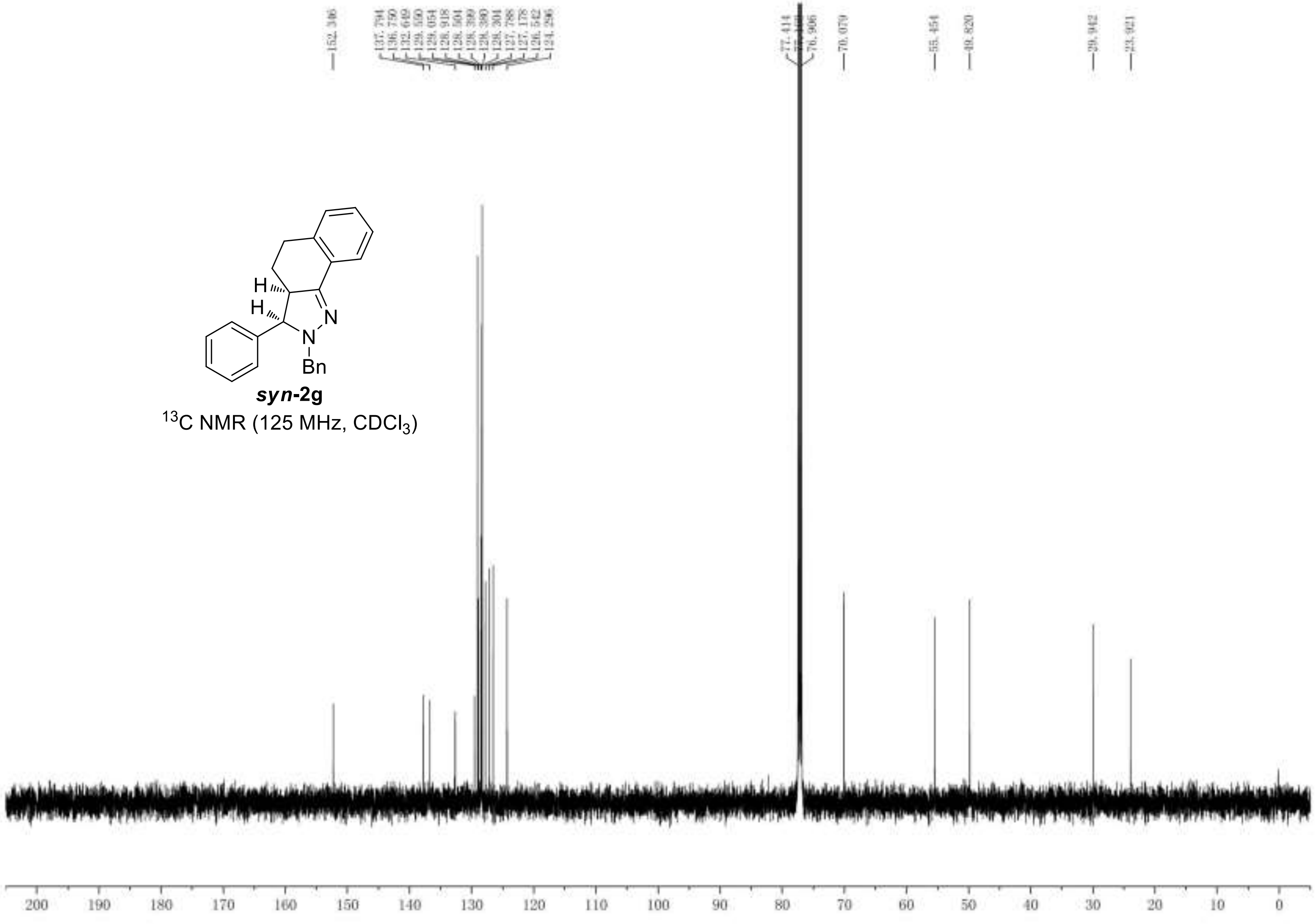

***syn*-2g**

^{13}C NMR (125 MHz, $CDCl_3$)

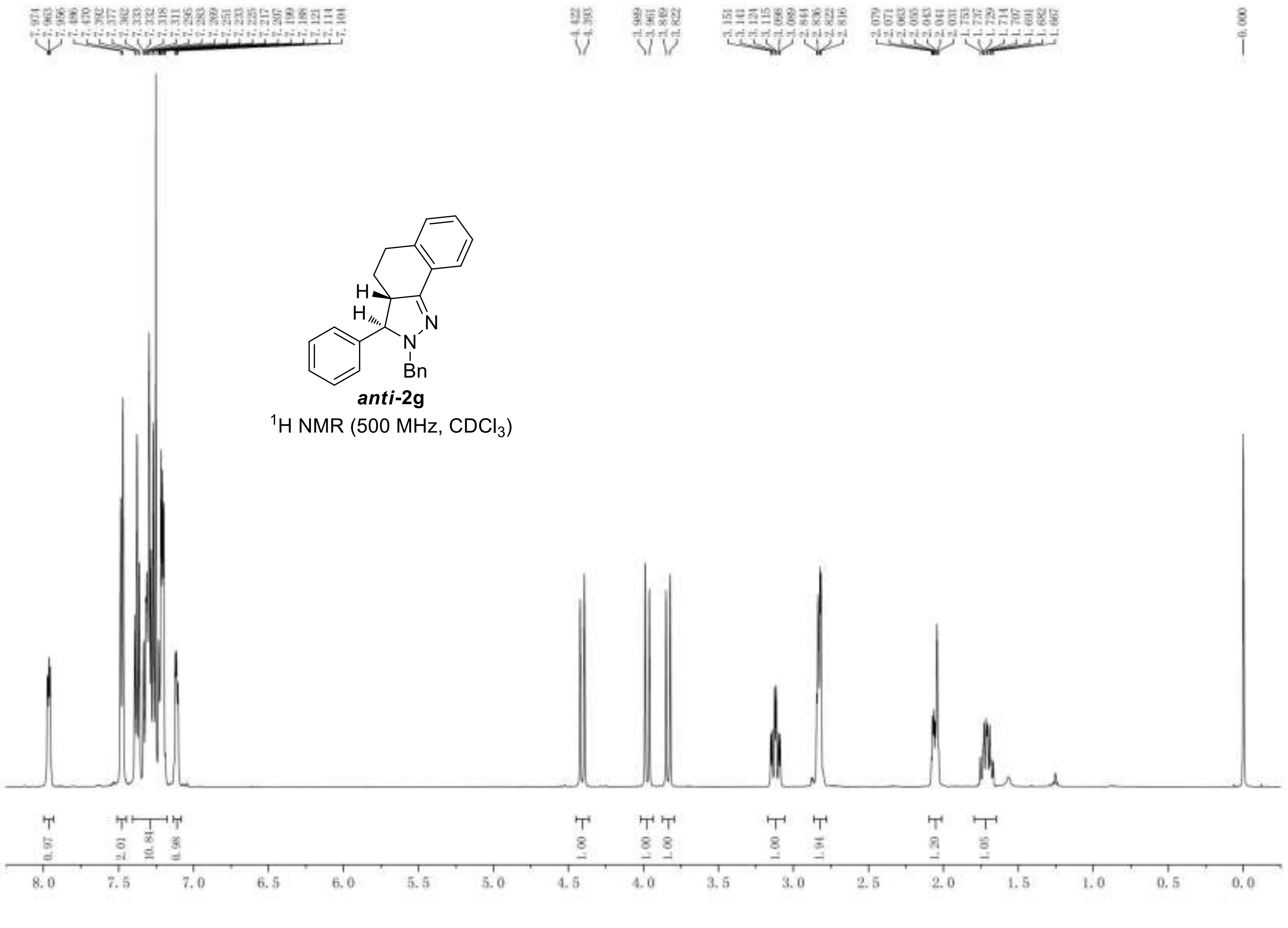

***anti*-2g**
^{1}H NMR (500 MHz, $CDCl_3$)

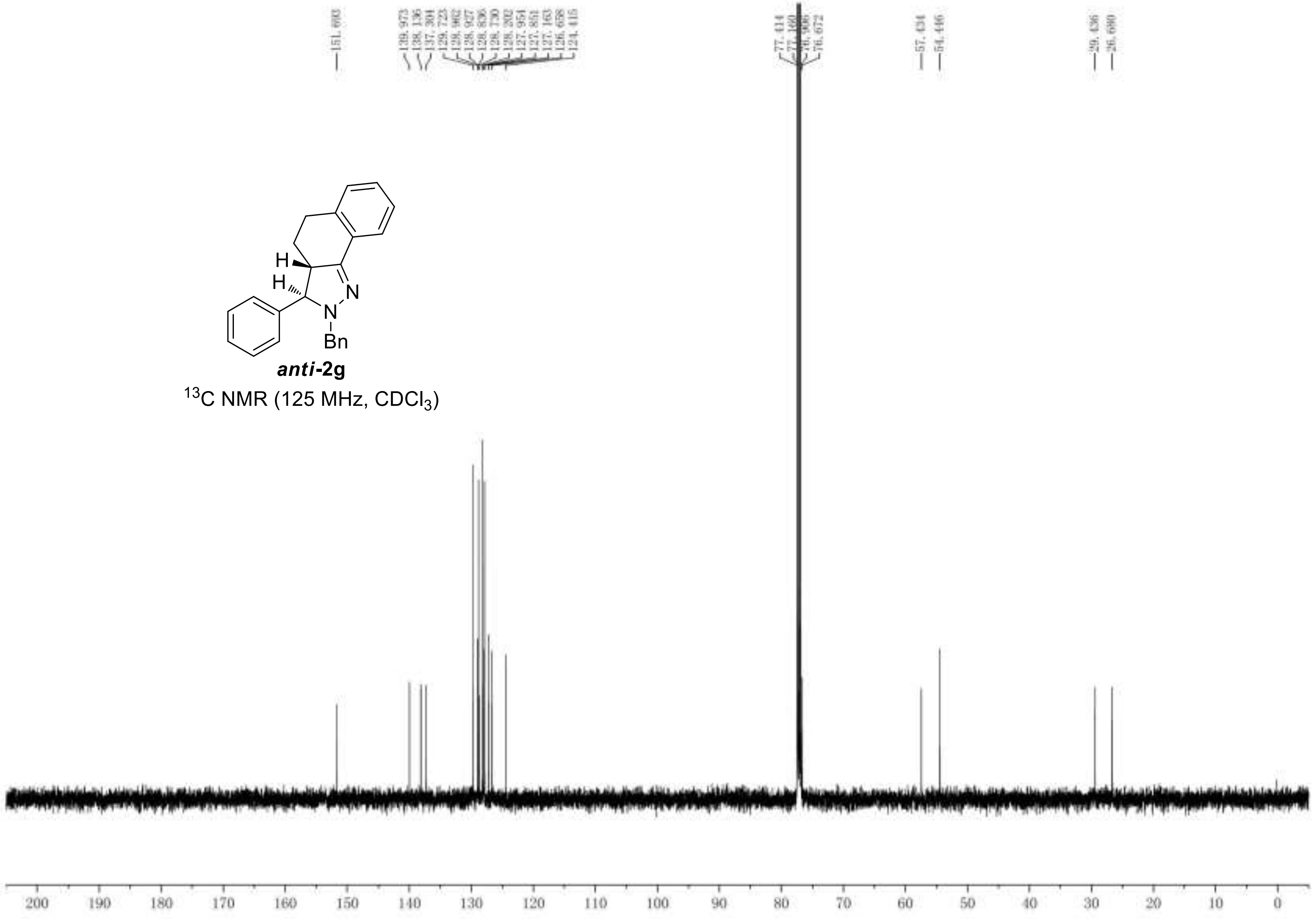

***anti*-2g**

^{13}C NMR (125 MHz, $CDCl_3$)

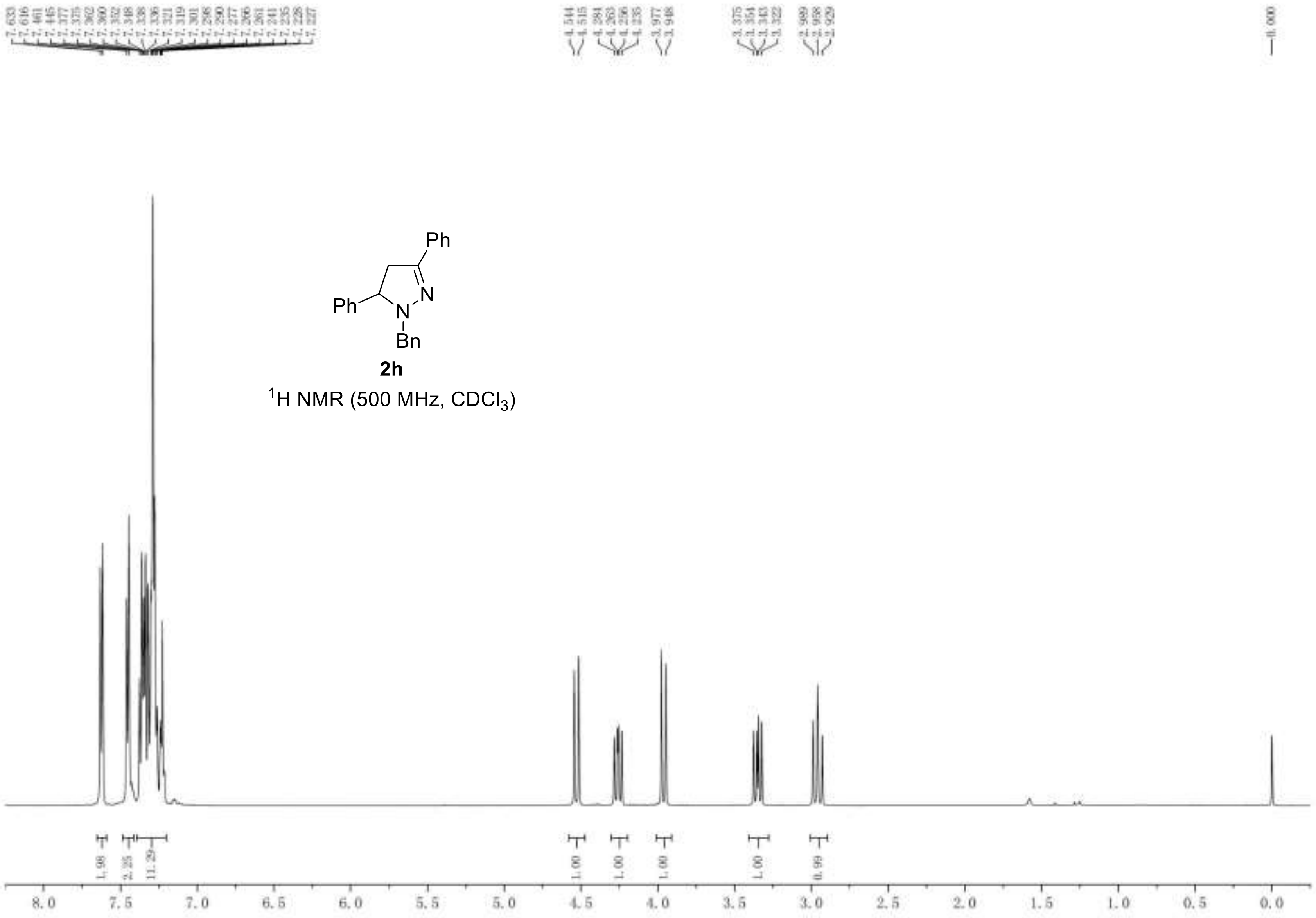

2h

^{1}H NMR (500 MHz, $CDCl_3$)

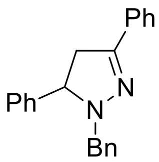

2h

^{13}C NMR (125 MHz, $CDCl_3$)

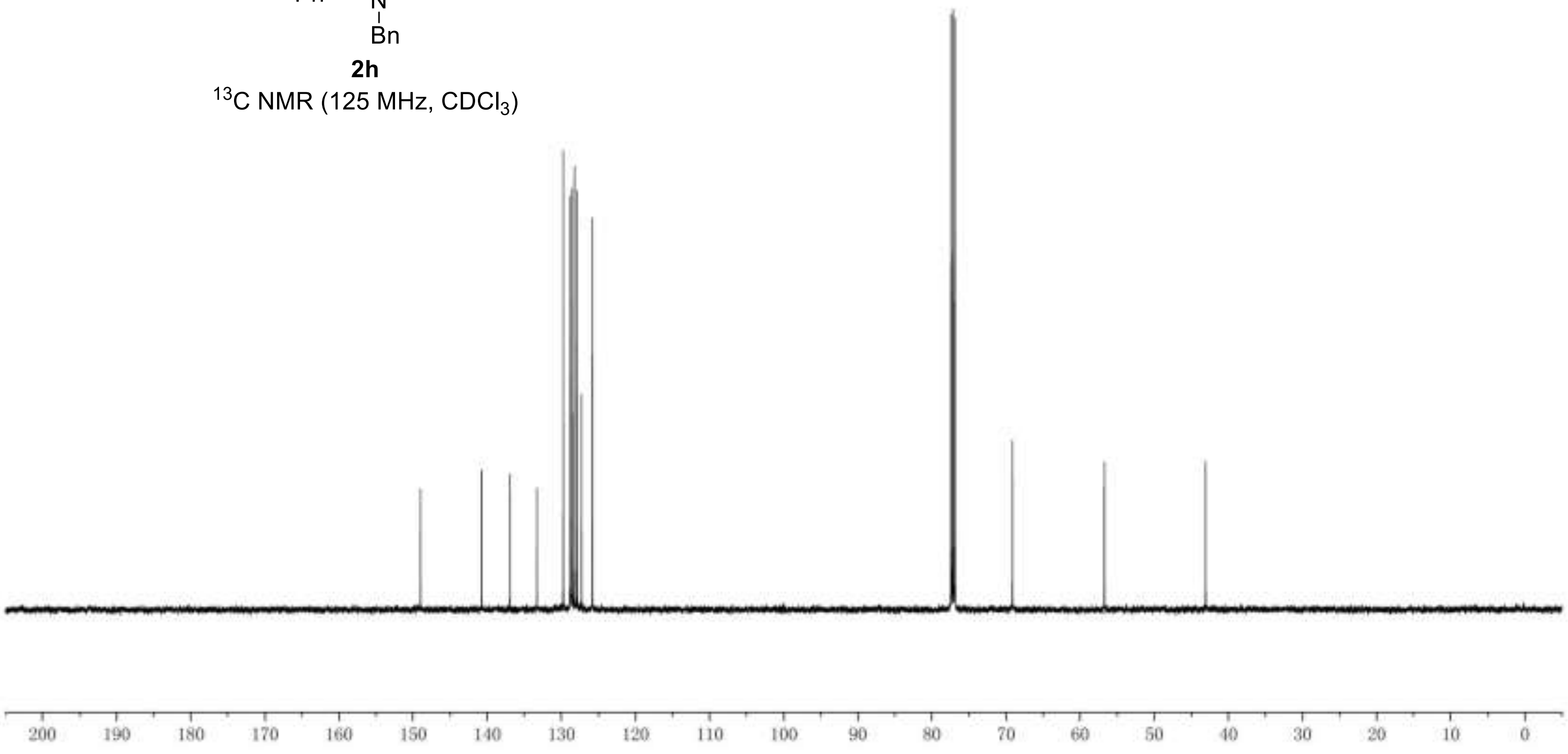

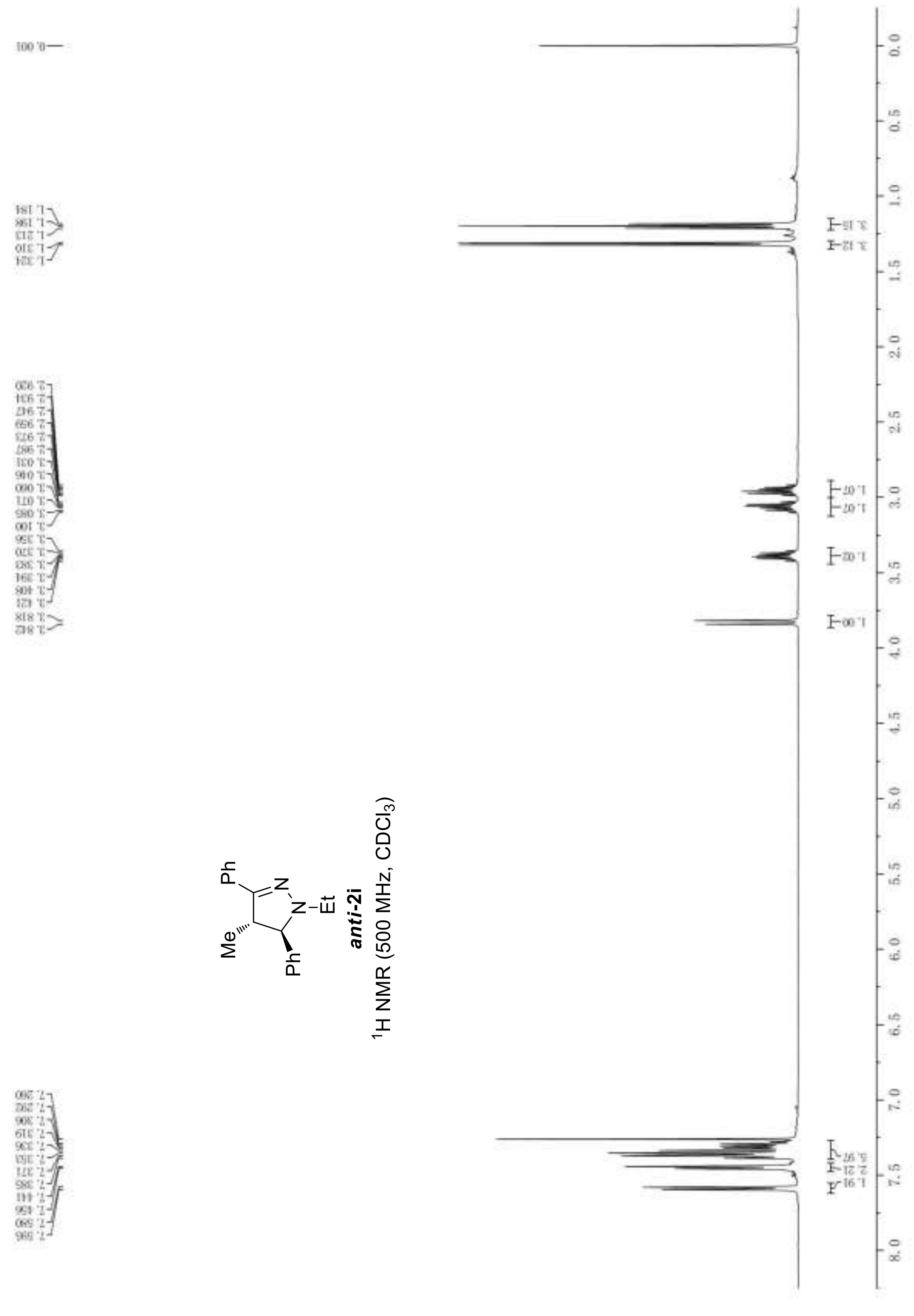

***anti*-2i**

^{1}H NMR (500 MHz, $CDCl_3$)

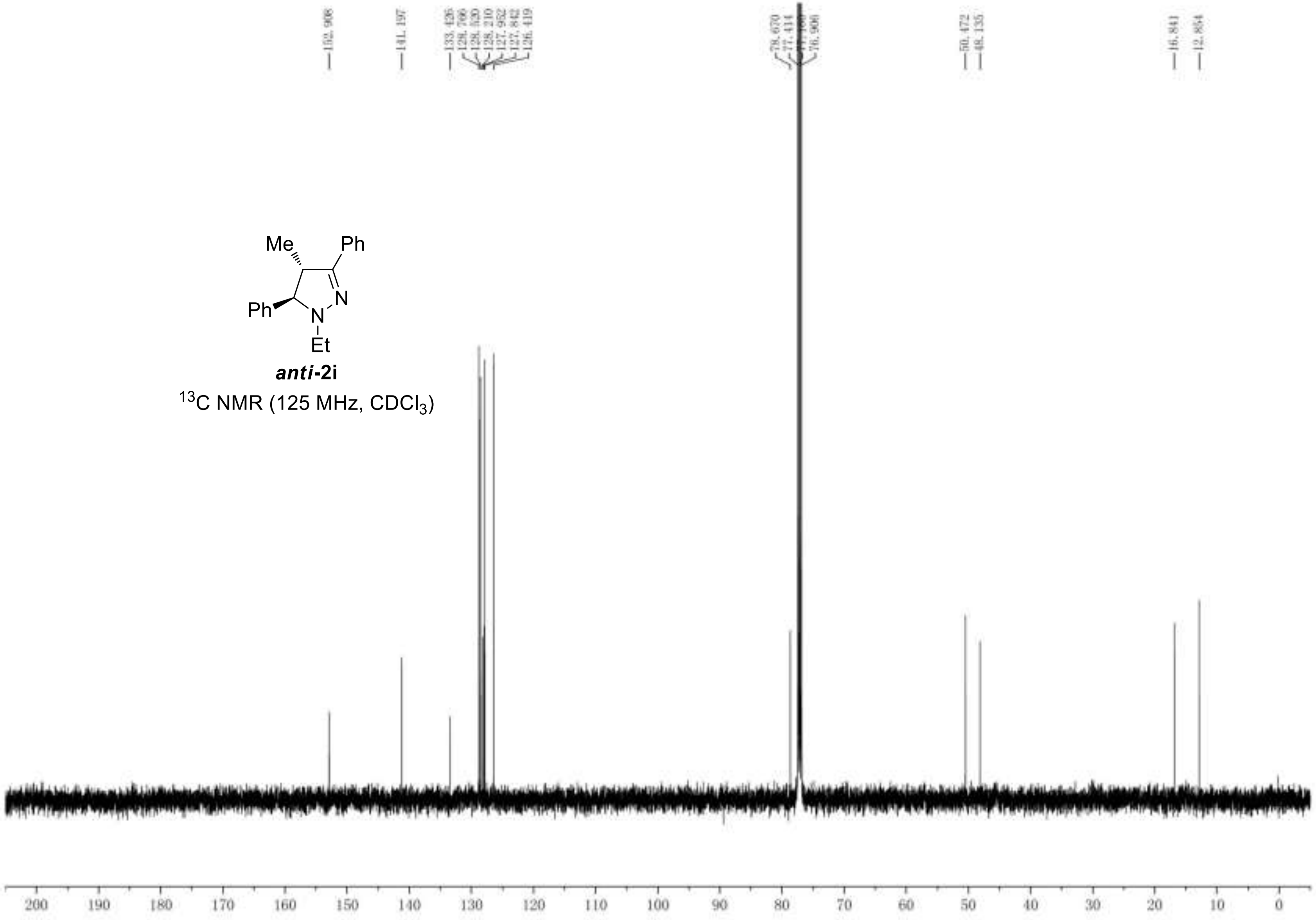

***anti*-2i**

^{13}C NMR (125 MHz, $CDCl_3$)

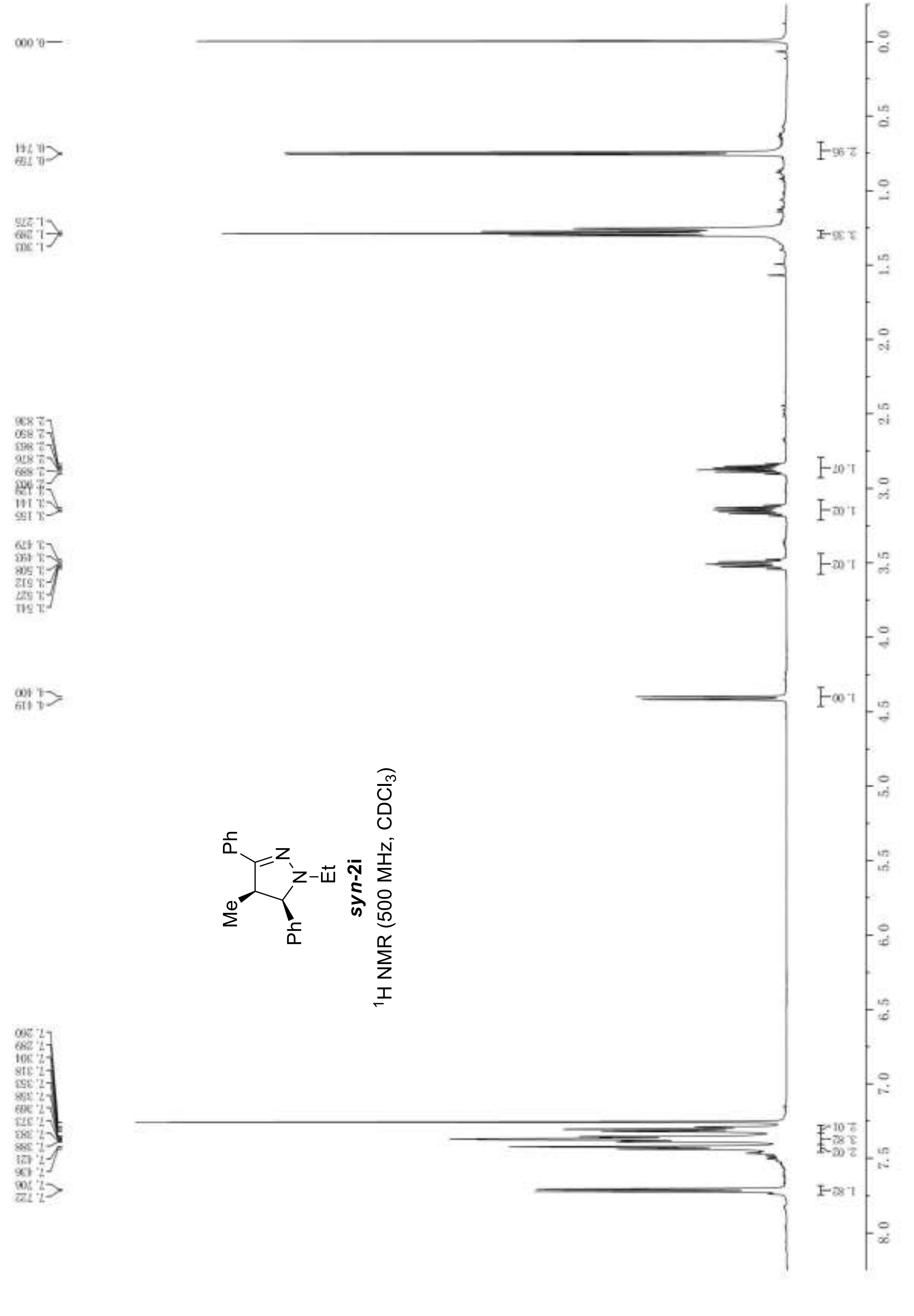

syn-**2i**

^{1}H NMR (500 MHz, $CDCl_3$)

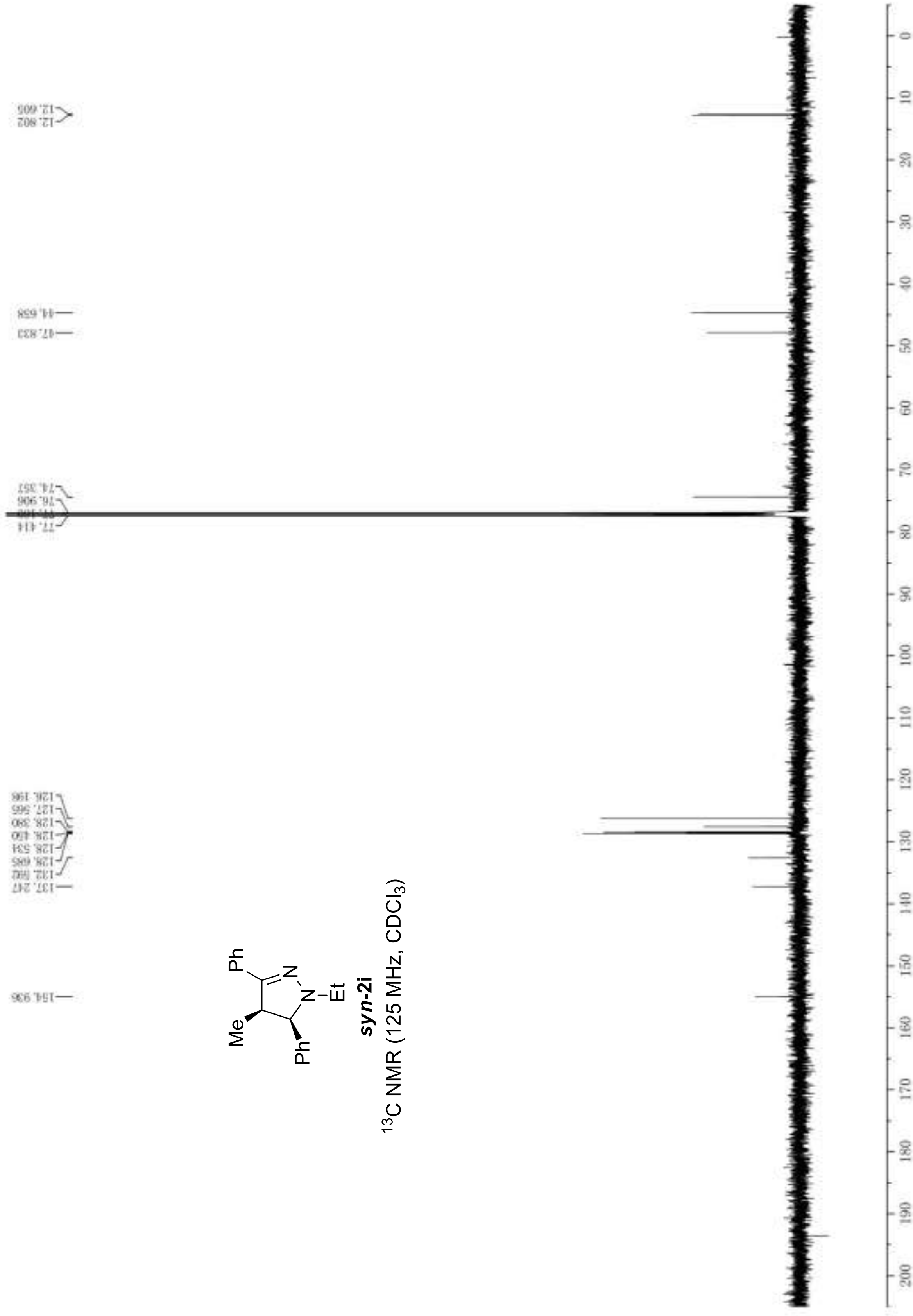

***syn*-2i**

^{13}C NMR (125 MHz, $CDCl_3$)

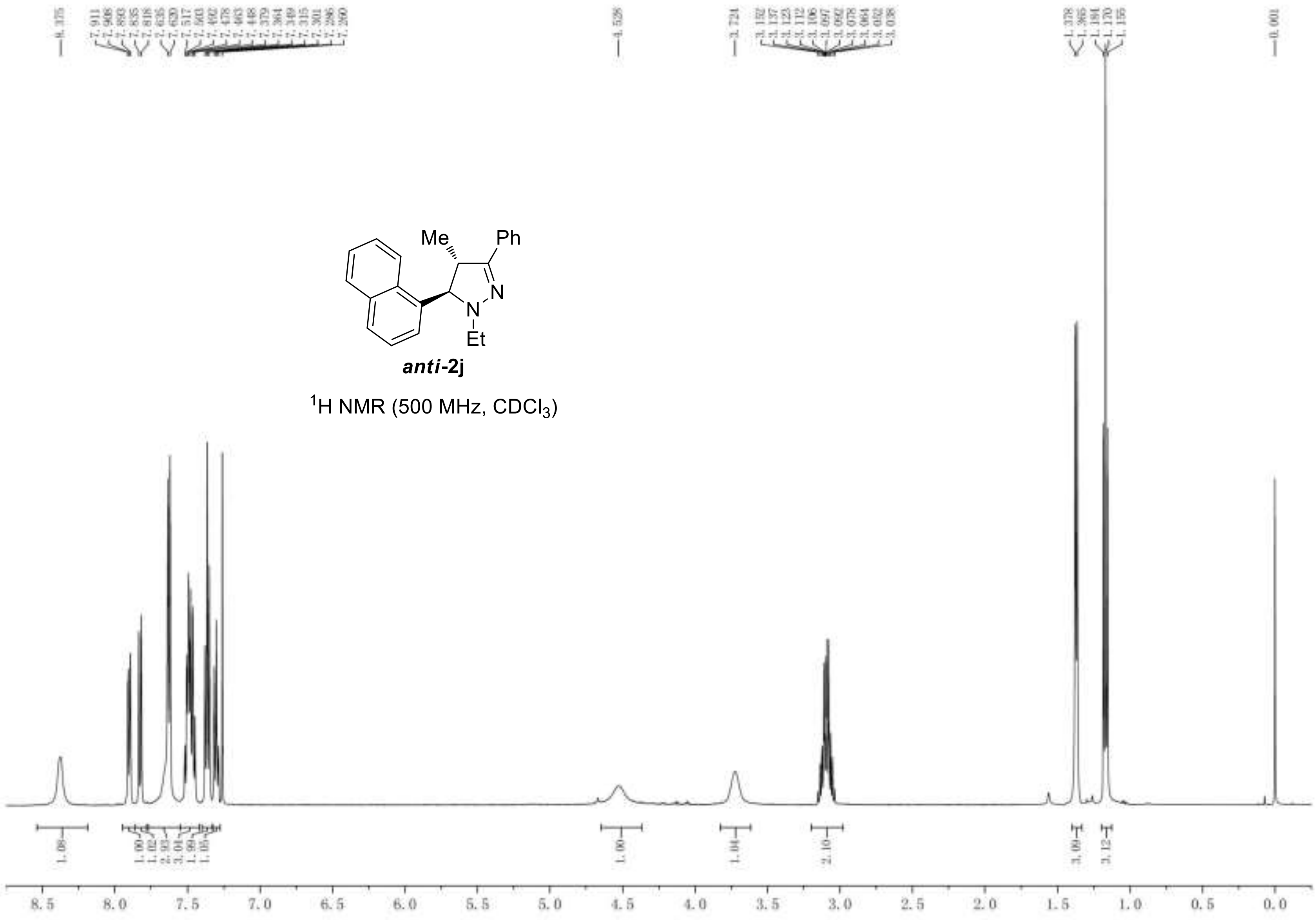

***anti*-2j**

^{1}H NMR (500 MHz, $CDCl_3$)

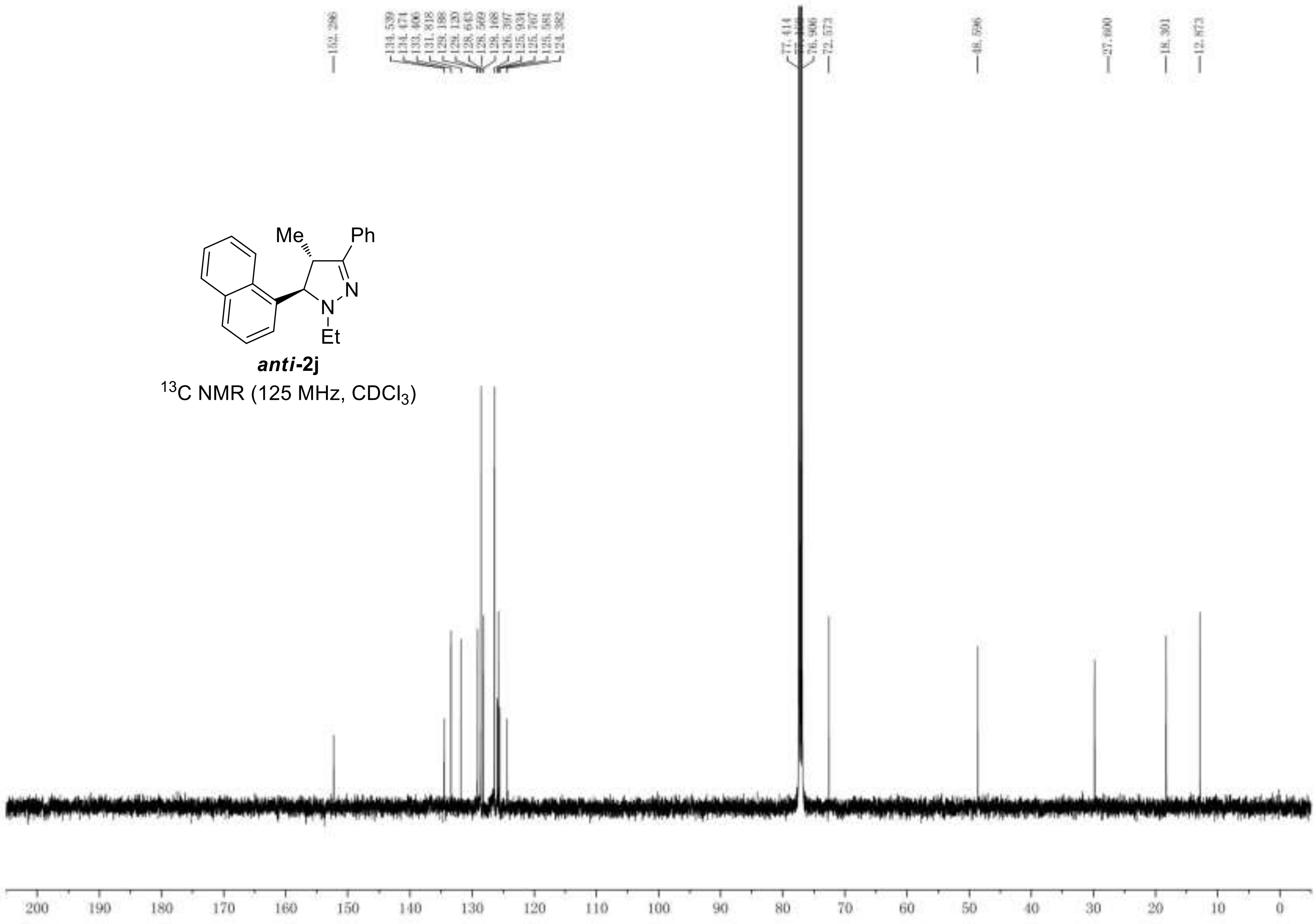

anti-**2j**

^{13}C NMR (125 MHz, $CDCl_3$)

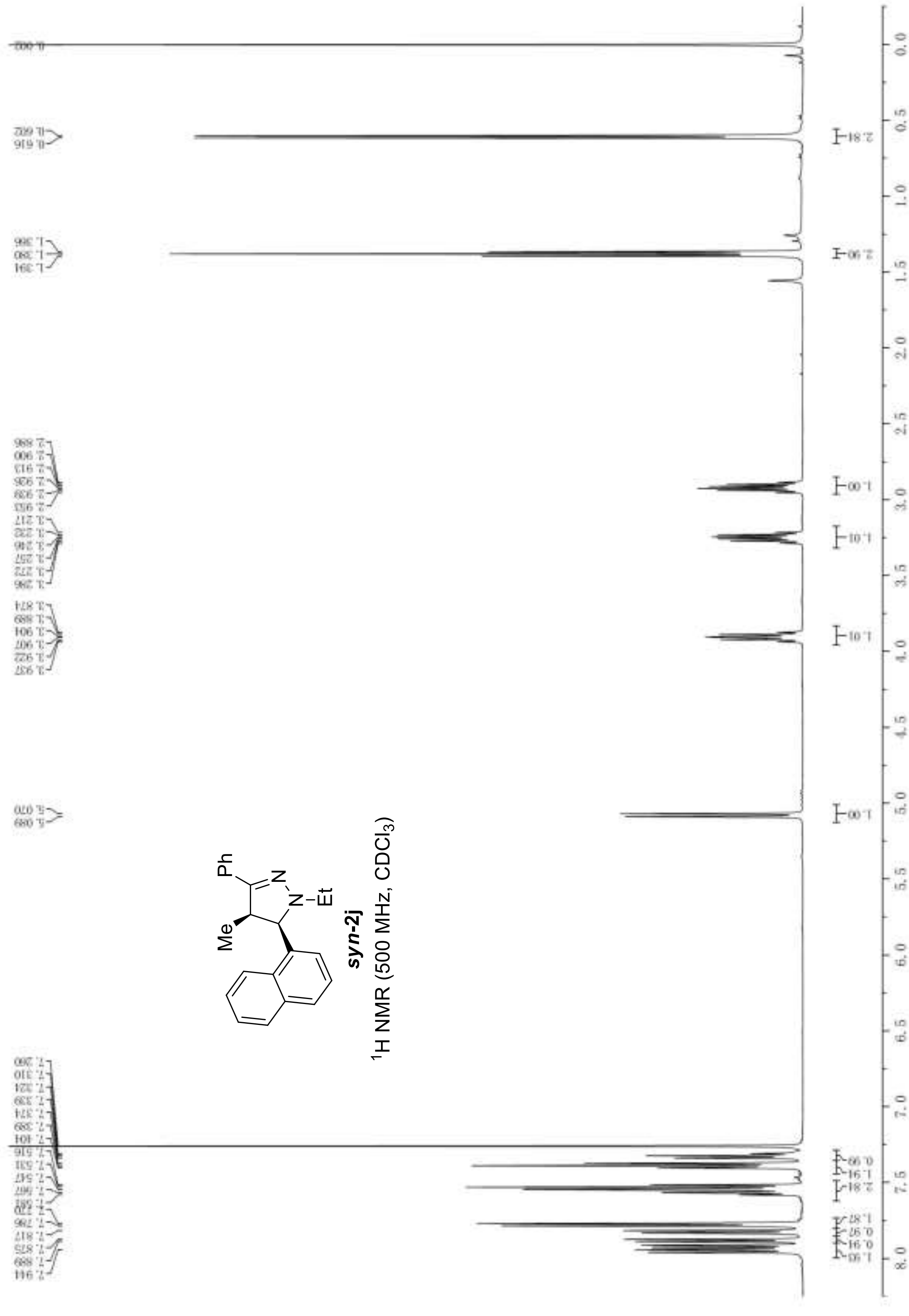
Ph
N
N–Et
Me
syn-2j
1H NMR (500 MHz, CDCl3)

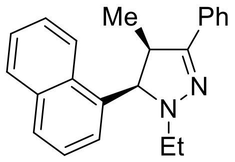

***syn*-2j**

^{13}C NMR (125 MHz, $CDCl_3$)

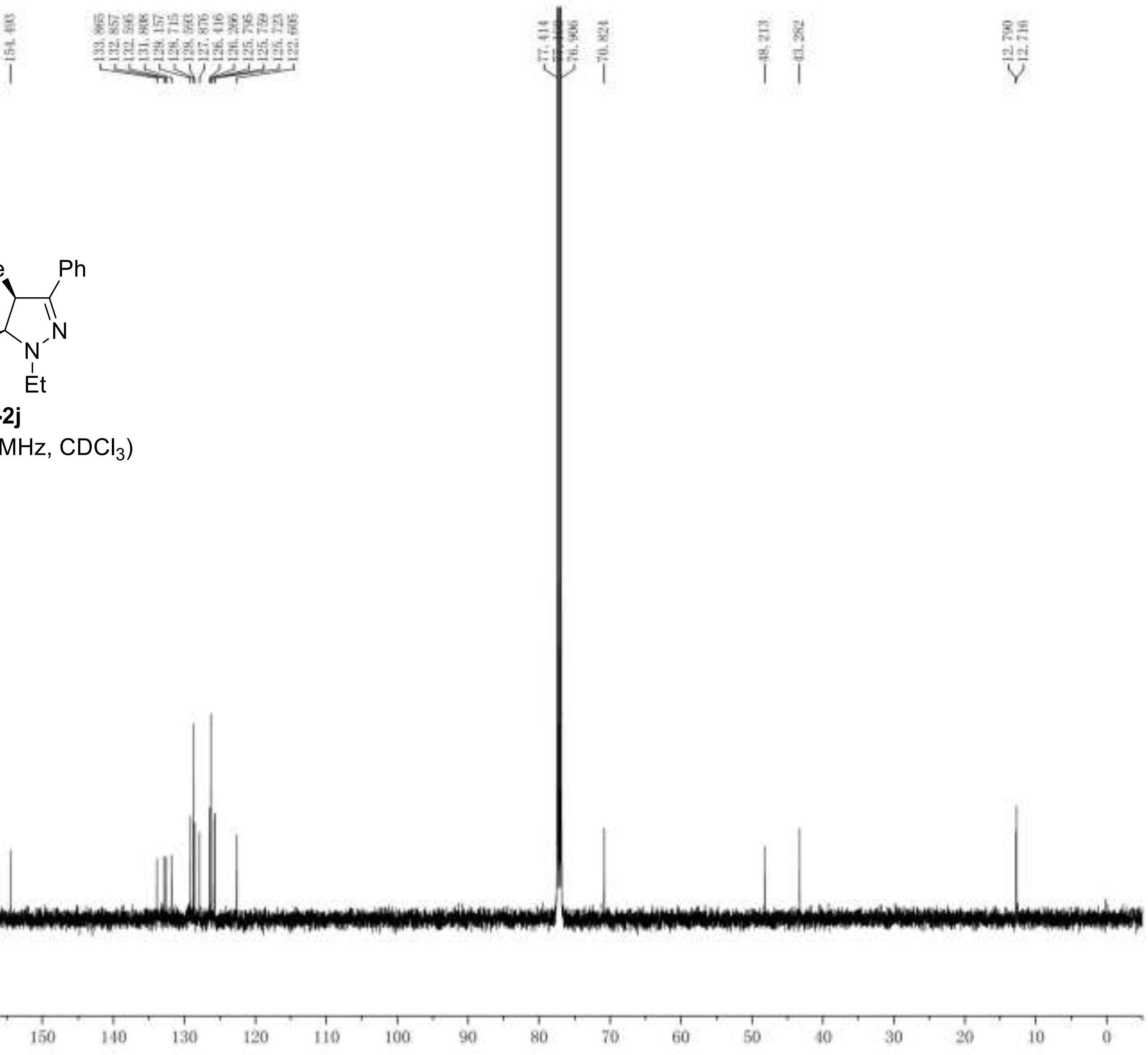

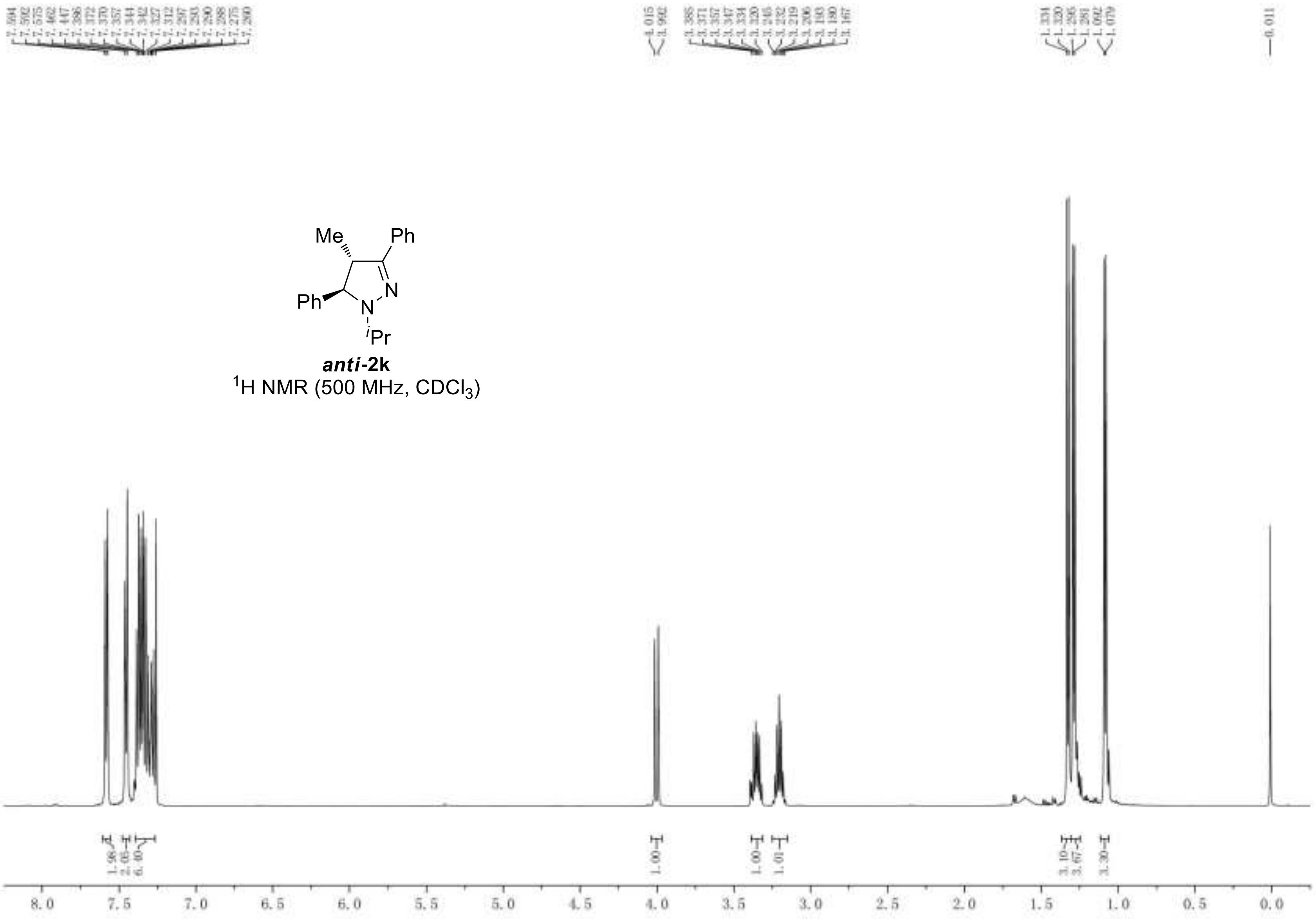

***anti*-2k**

^1H NMR (500 MHz, $CDCl_3$)

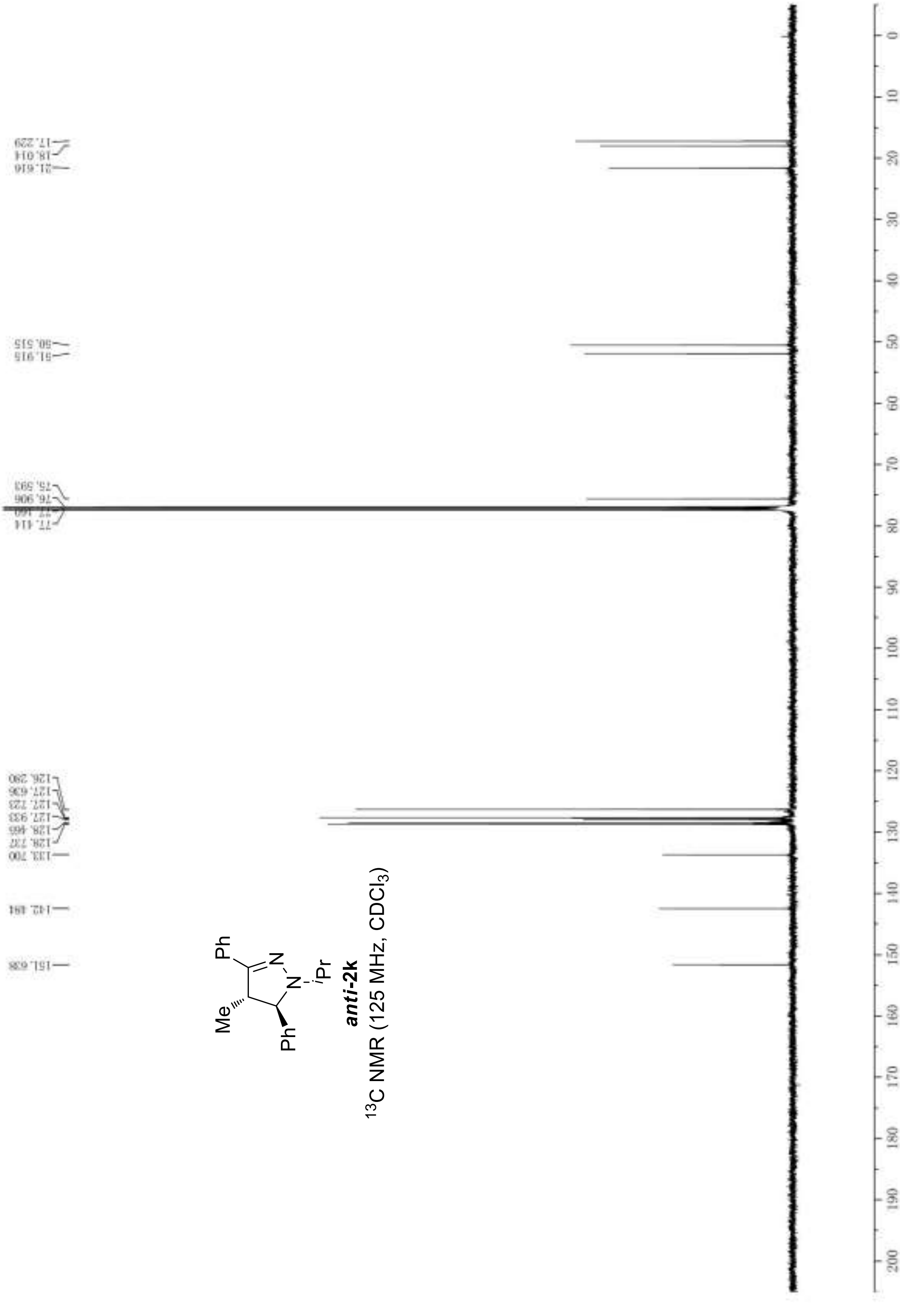

***anti*-2k**
^{13}C NMR (125 MHz, $CDCl_3$)

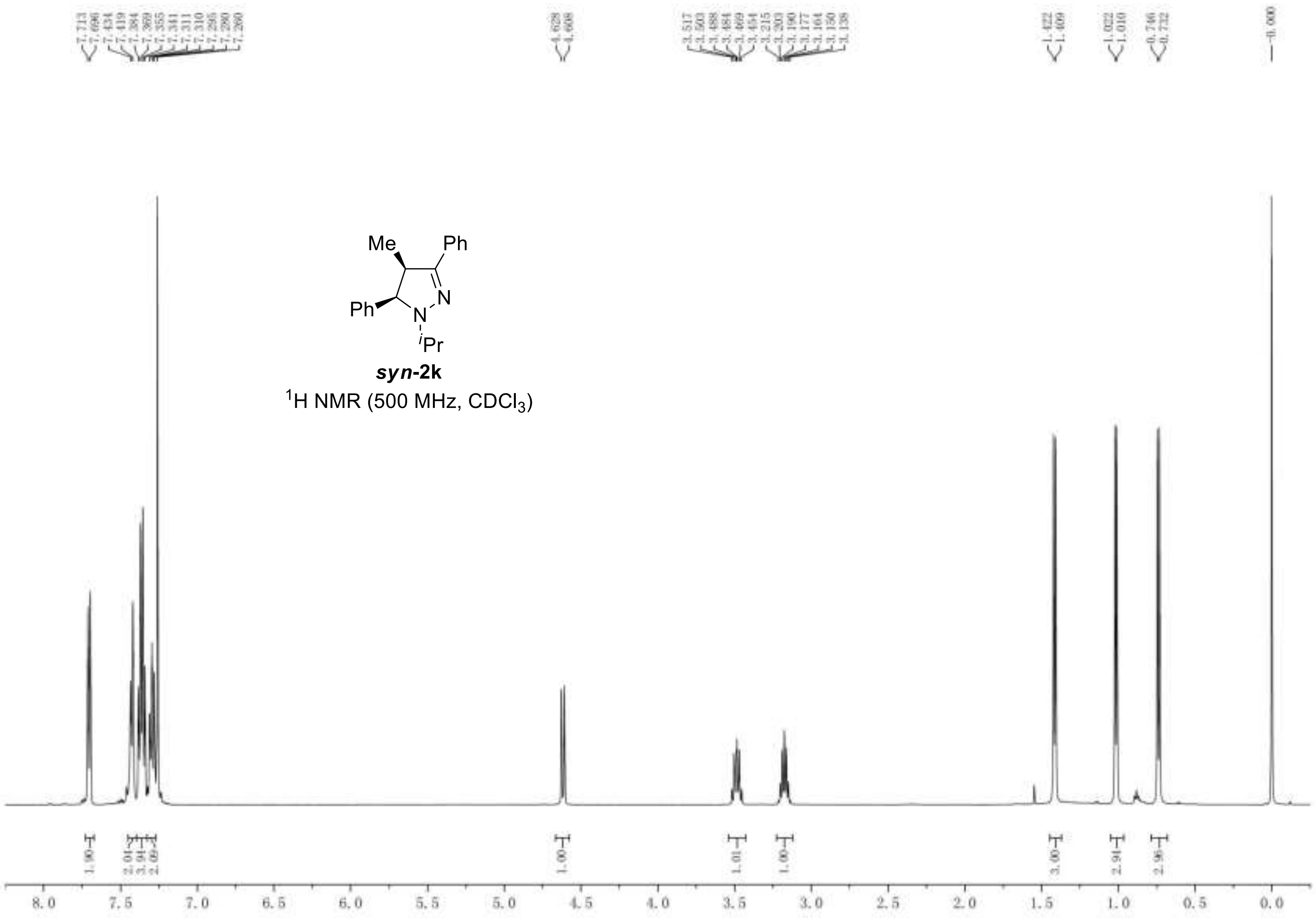

***syn*-2k**

^1H NMR (500 MHz, $CDCl_3$)

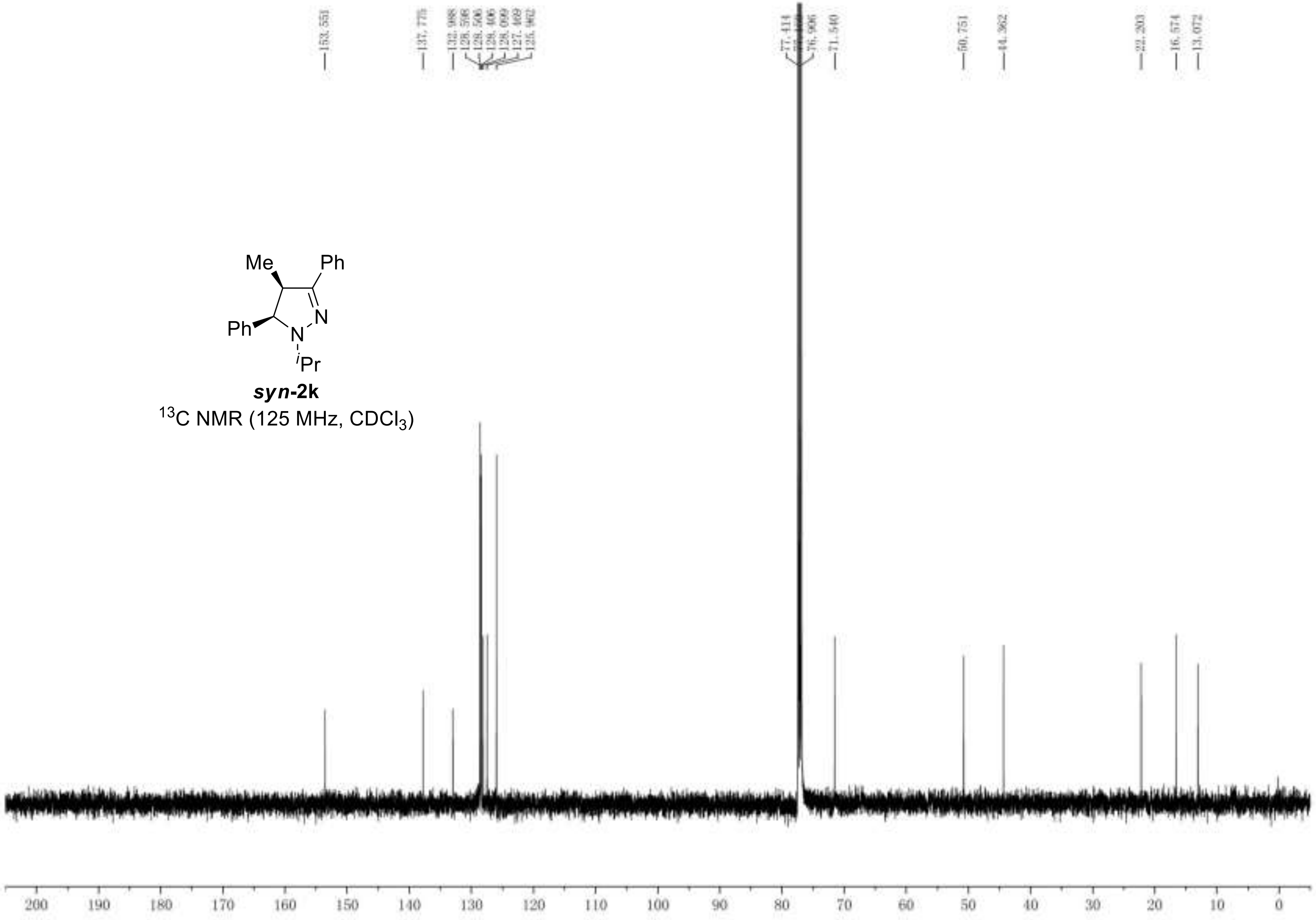

***syn*-2k**

^{13}C NMR (125 MHz, $CDCl_3$)

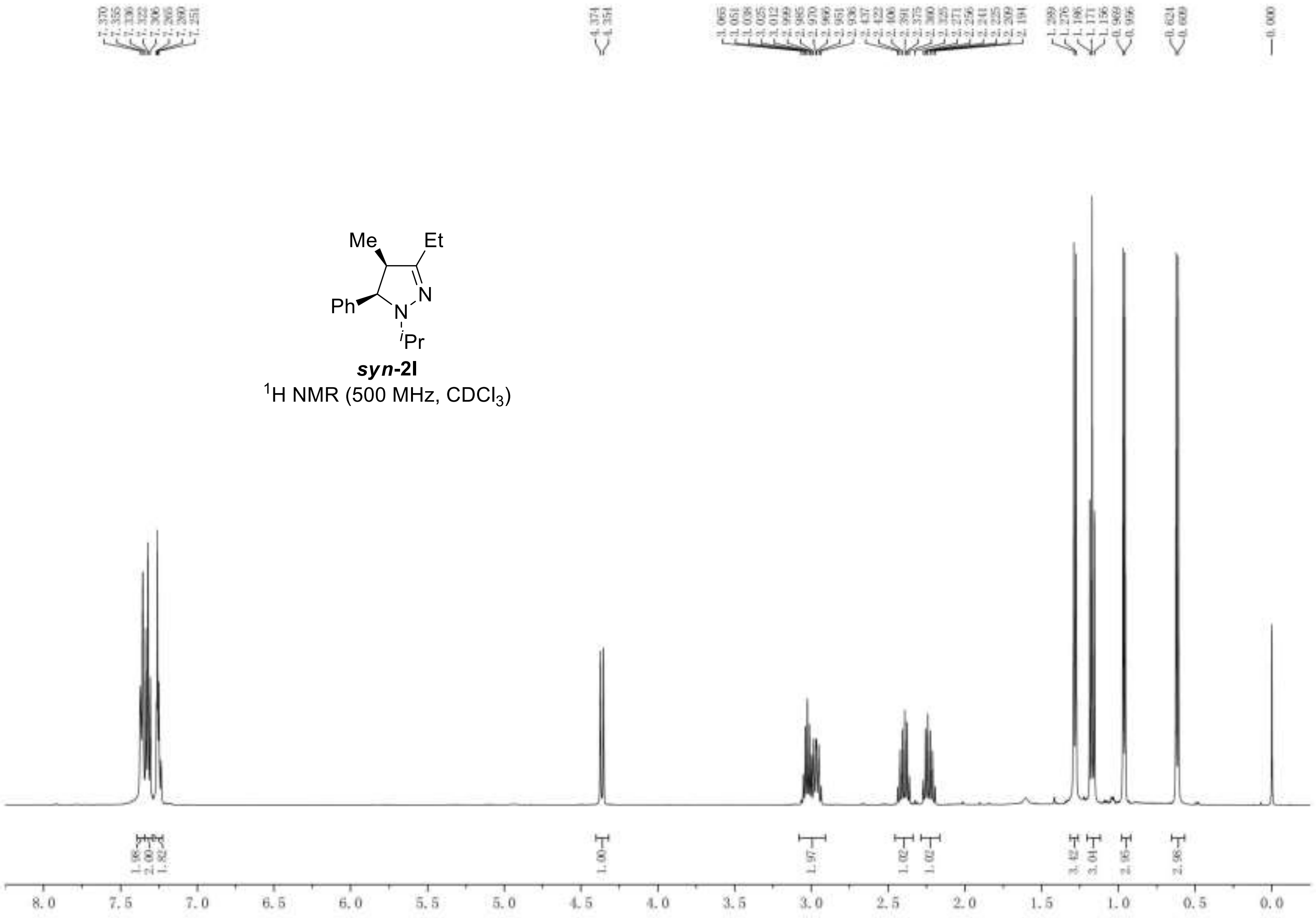

***syn*-2l**

^{1}H NMR (500 MHz, $CDCl_3$)

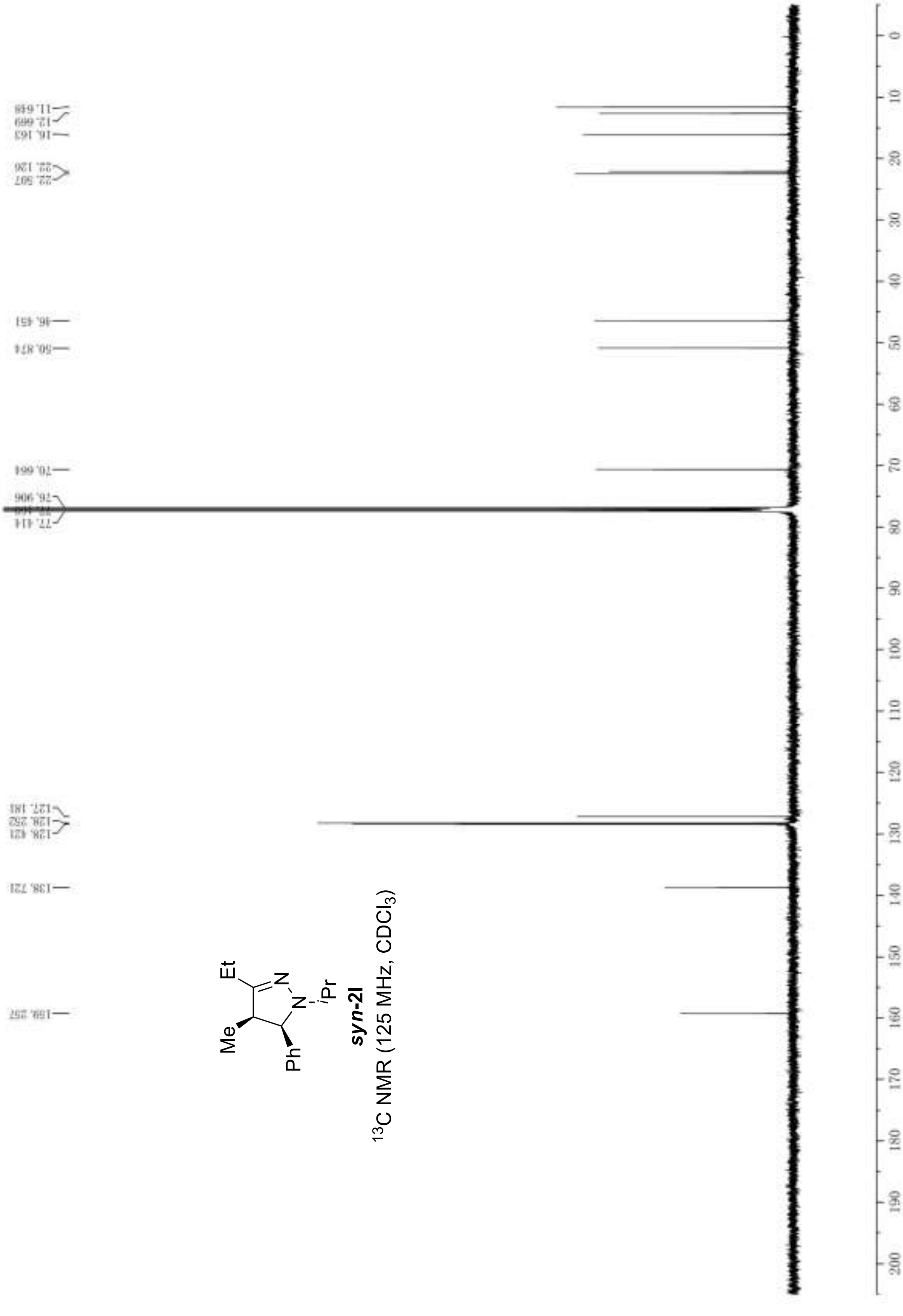

11.648
12.669
16.163
22.126
22.507
46.451
50.874
70.664
76.906
77.414
127.181
128.252
128.421
138.721
159.257
Me
Et
N
N
Ph
iPr
syn-2l
13C NMR (125 MHz, CDCl3)

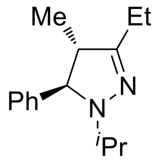

***anti*-2l**

^{1}H NMR (500 MHz, $CDCl_3$)

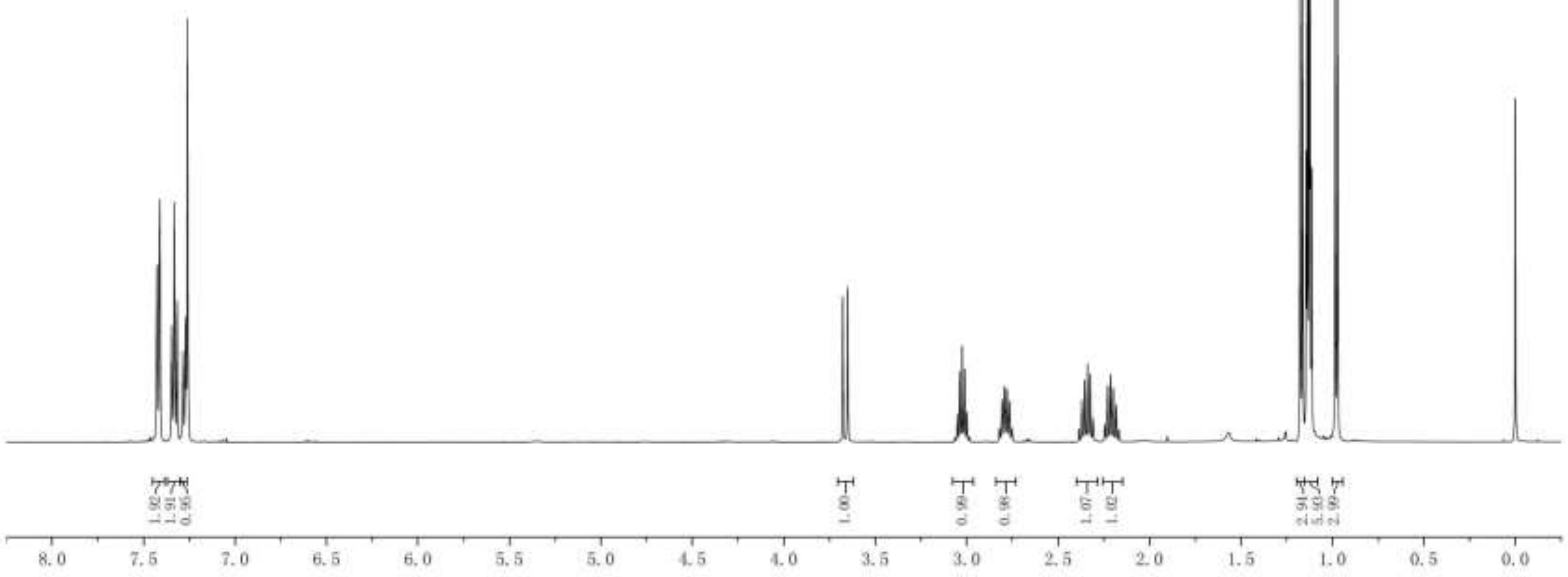

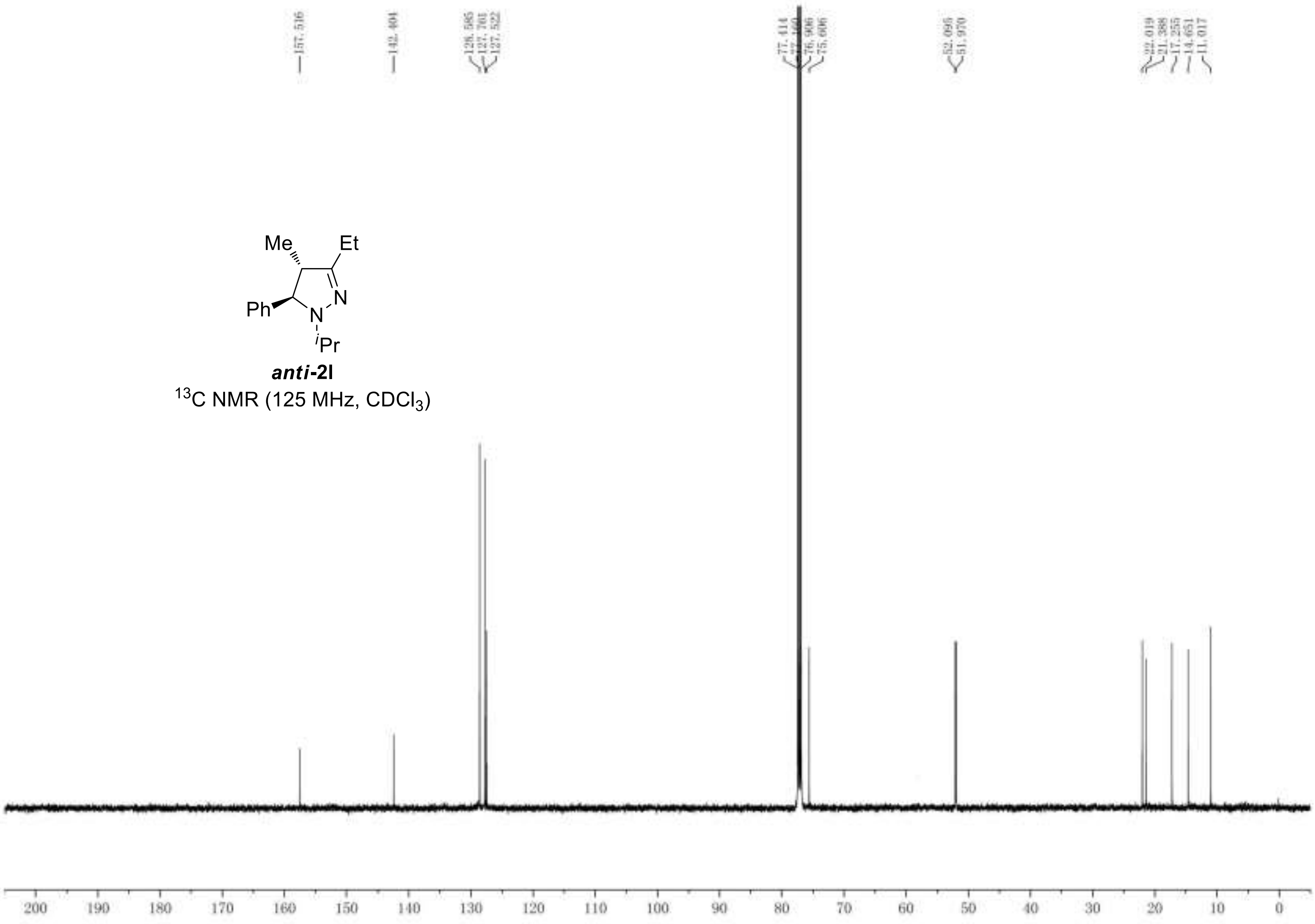

***anti*-2l**

^{13}C NMR (125 MHz, $CDCl_3$)

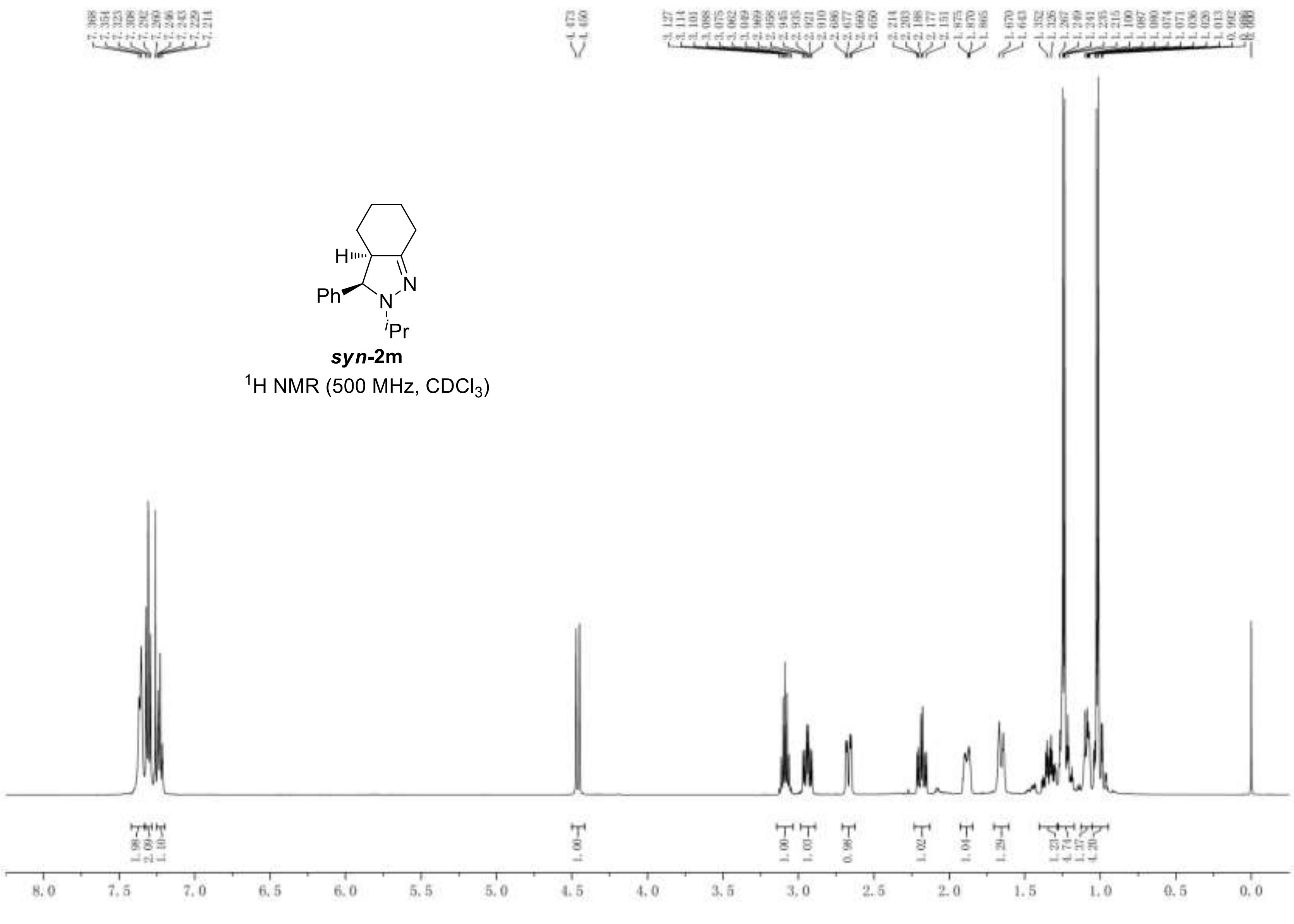

***syn*-2m**
^{1}H NMR (500 MHz, $CDCl_3$)

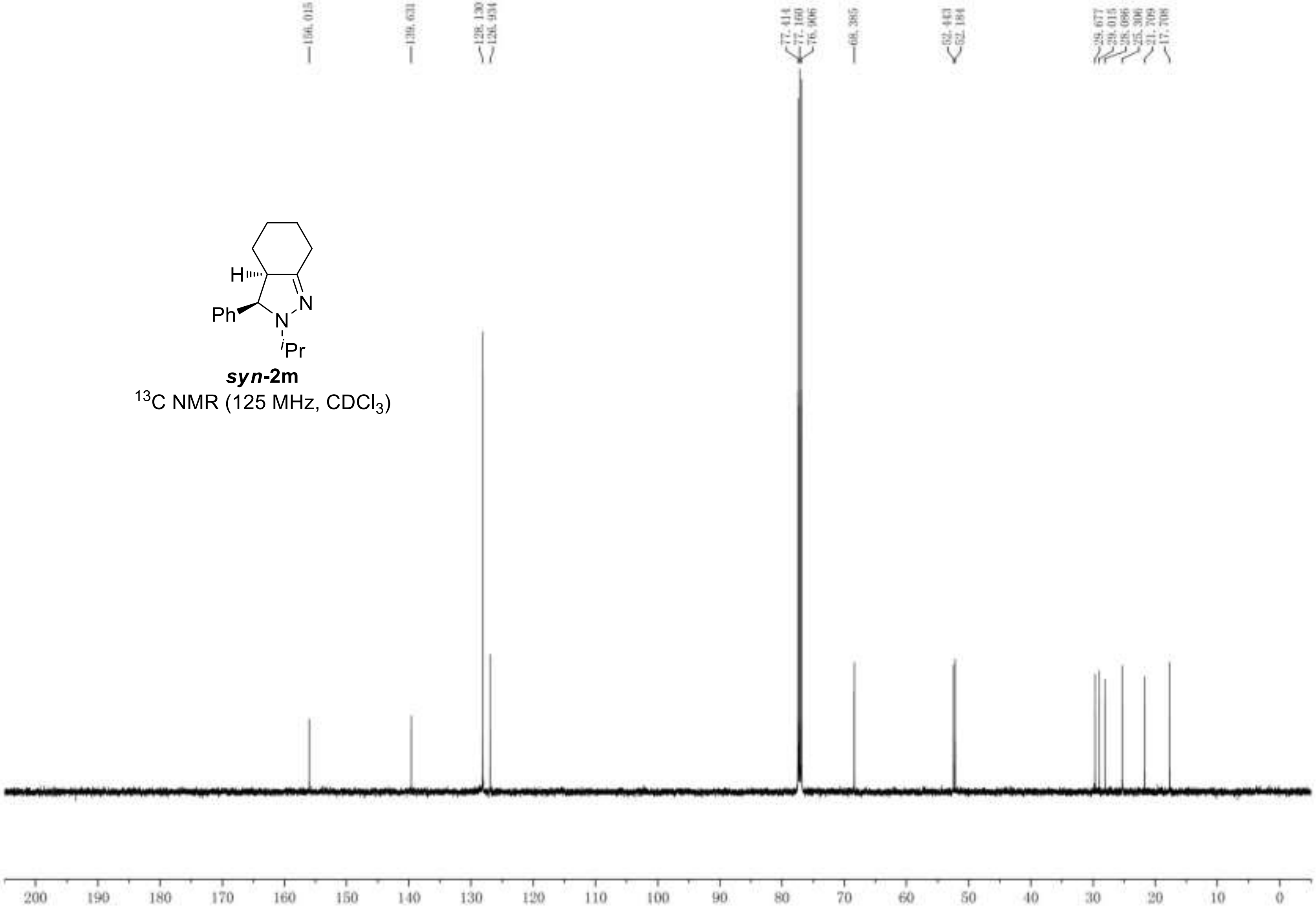

***syn*-2m**

^{13}C NMR (125 MHz, $CDCl_3$)

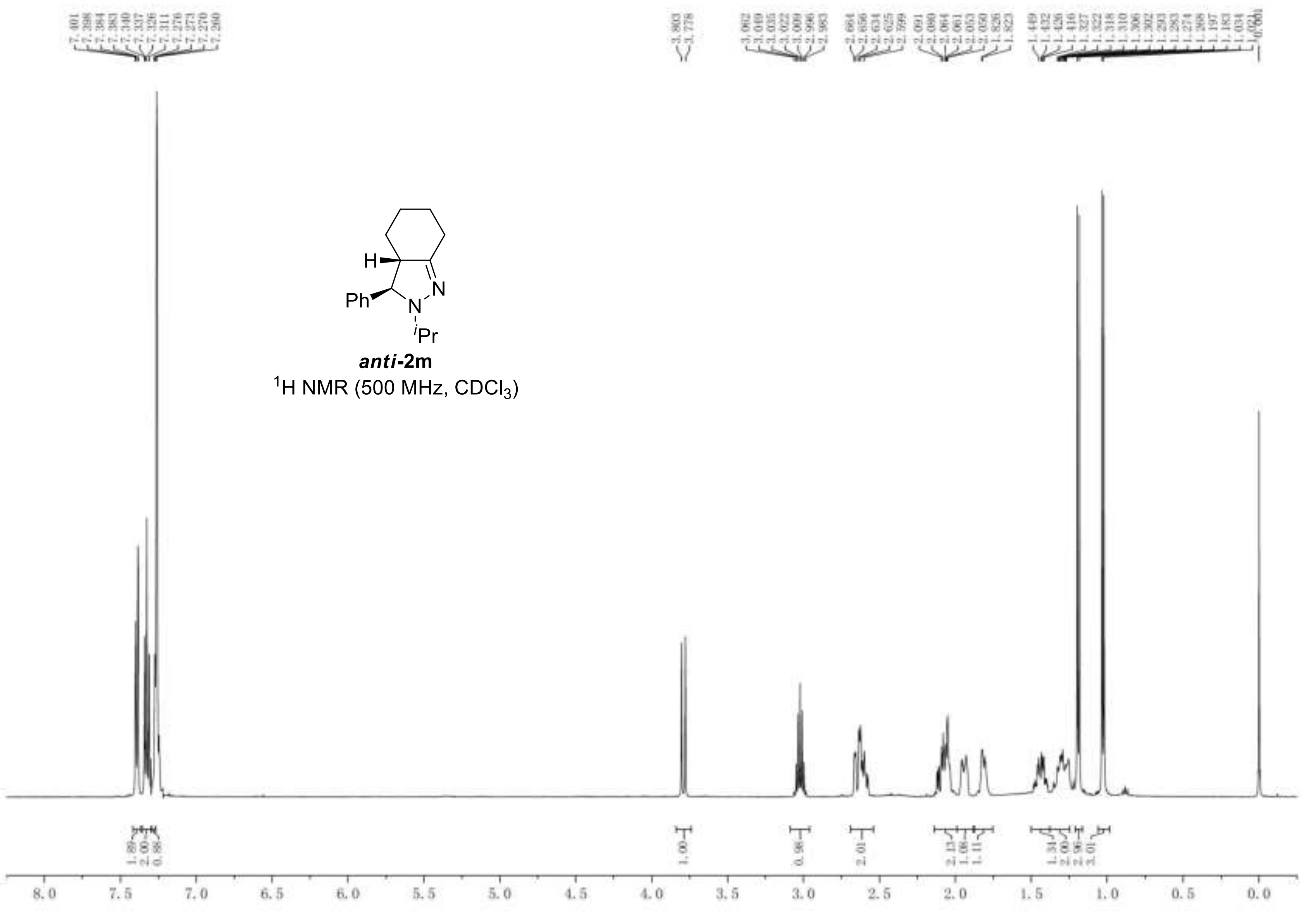

***anti*-2m**

^1H NMR (500 MHz, $CDCl_3$)

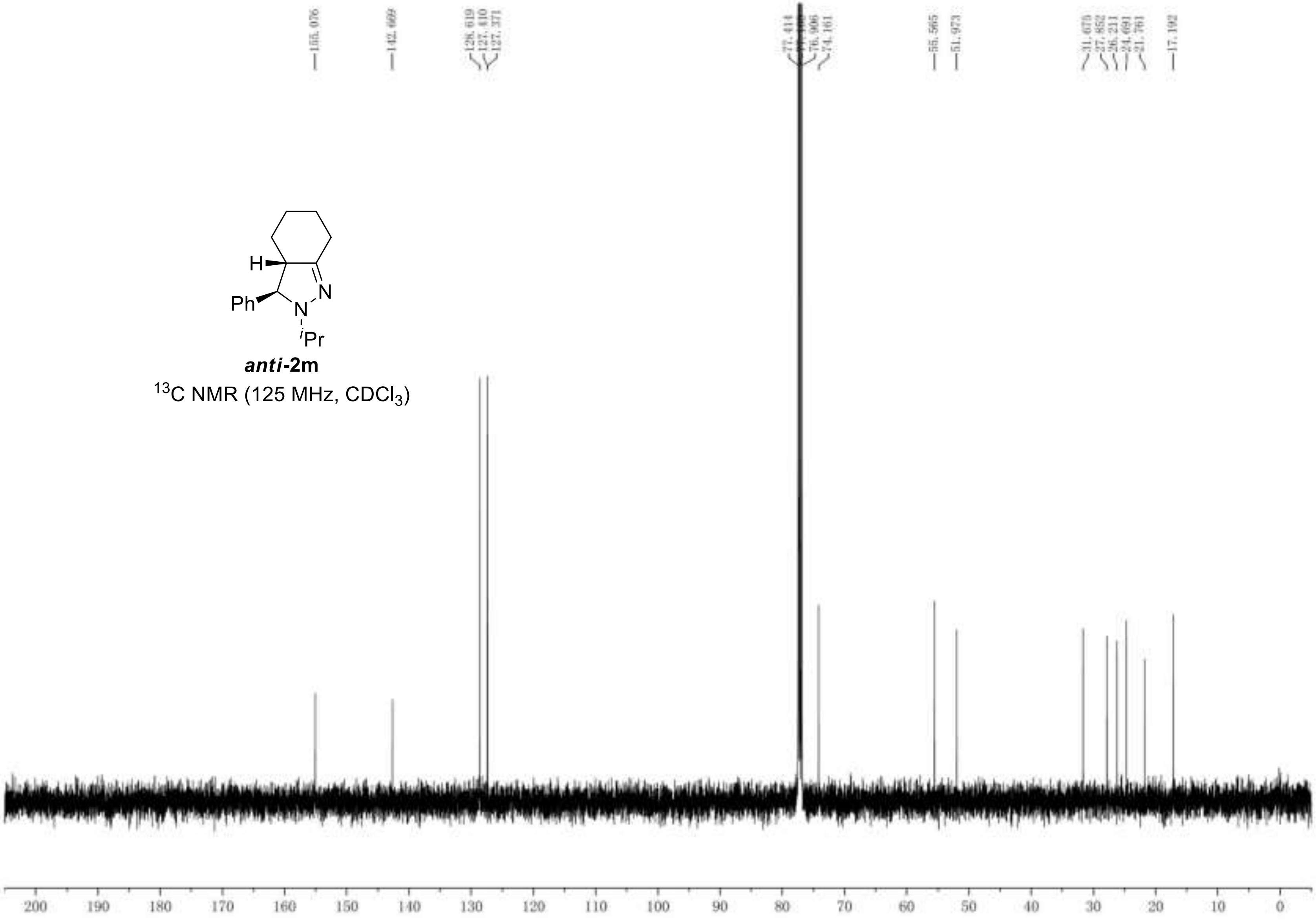

***anti*-2m**

^{13}C NMR (125 MHz, $CDCl_3$)

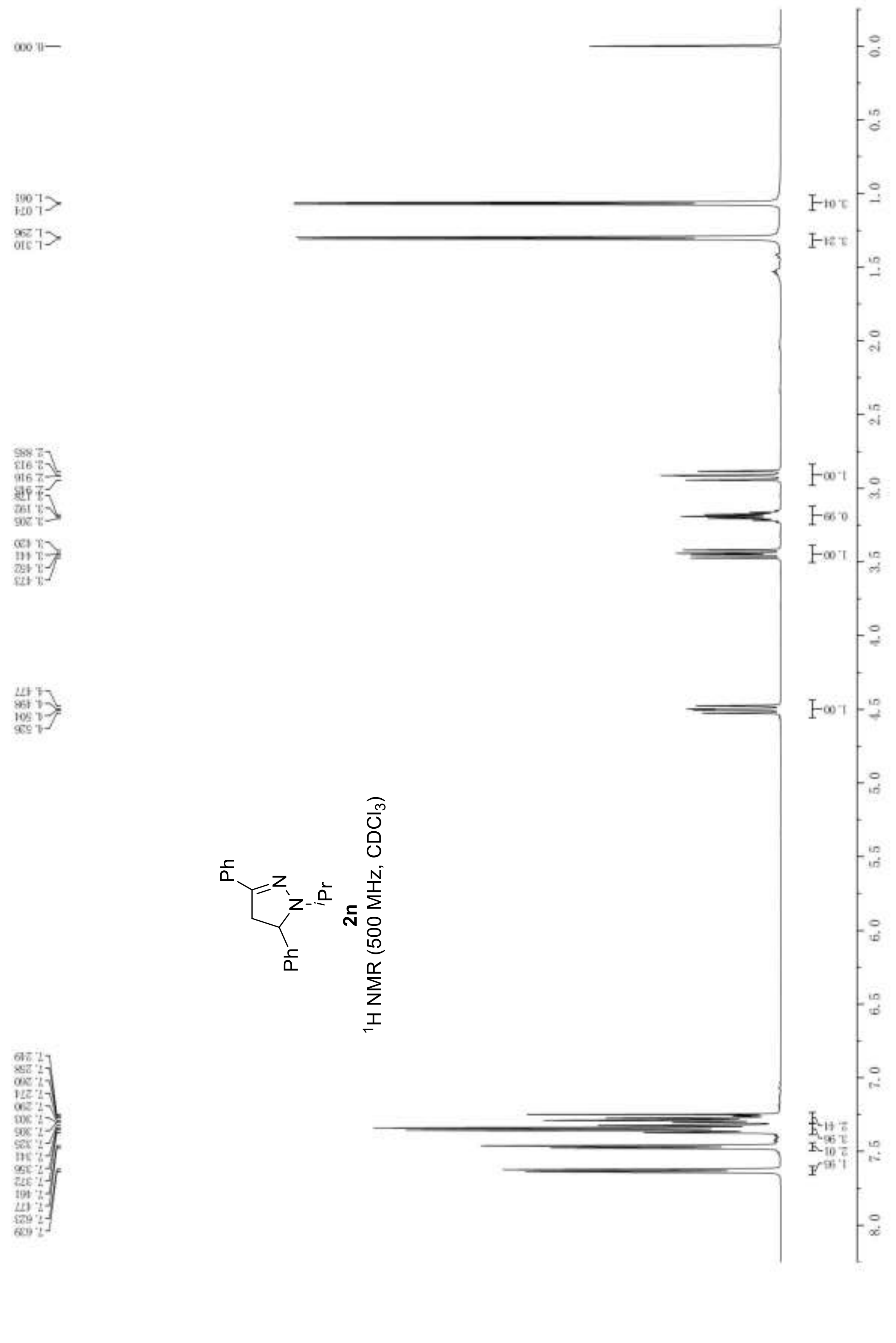

2n

^{1}H NMR (500 MHz, $CDCl_3$)

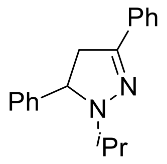

2n

^{13}C NMR (125 MHz, $CDCl_3$)

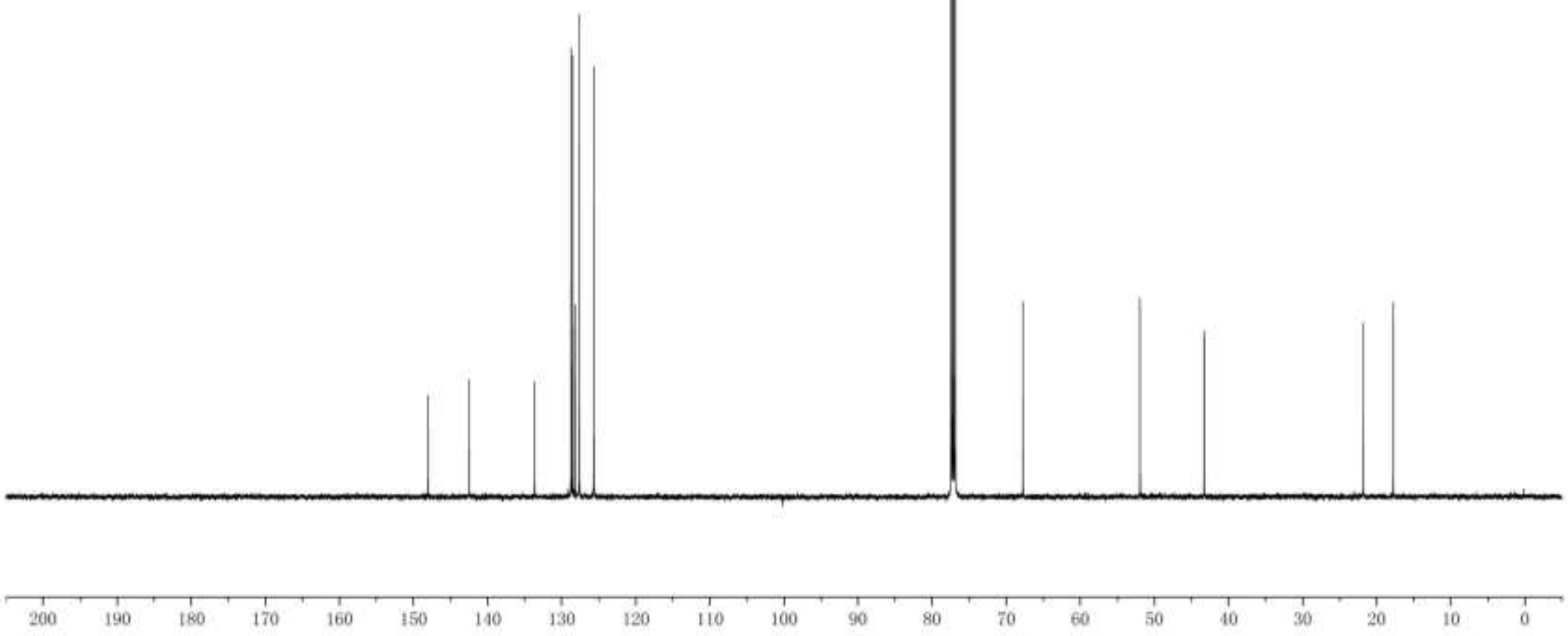

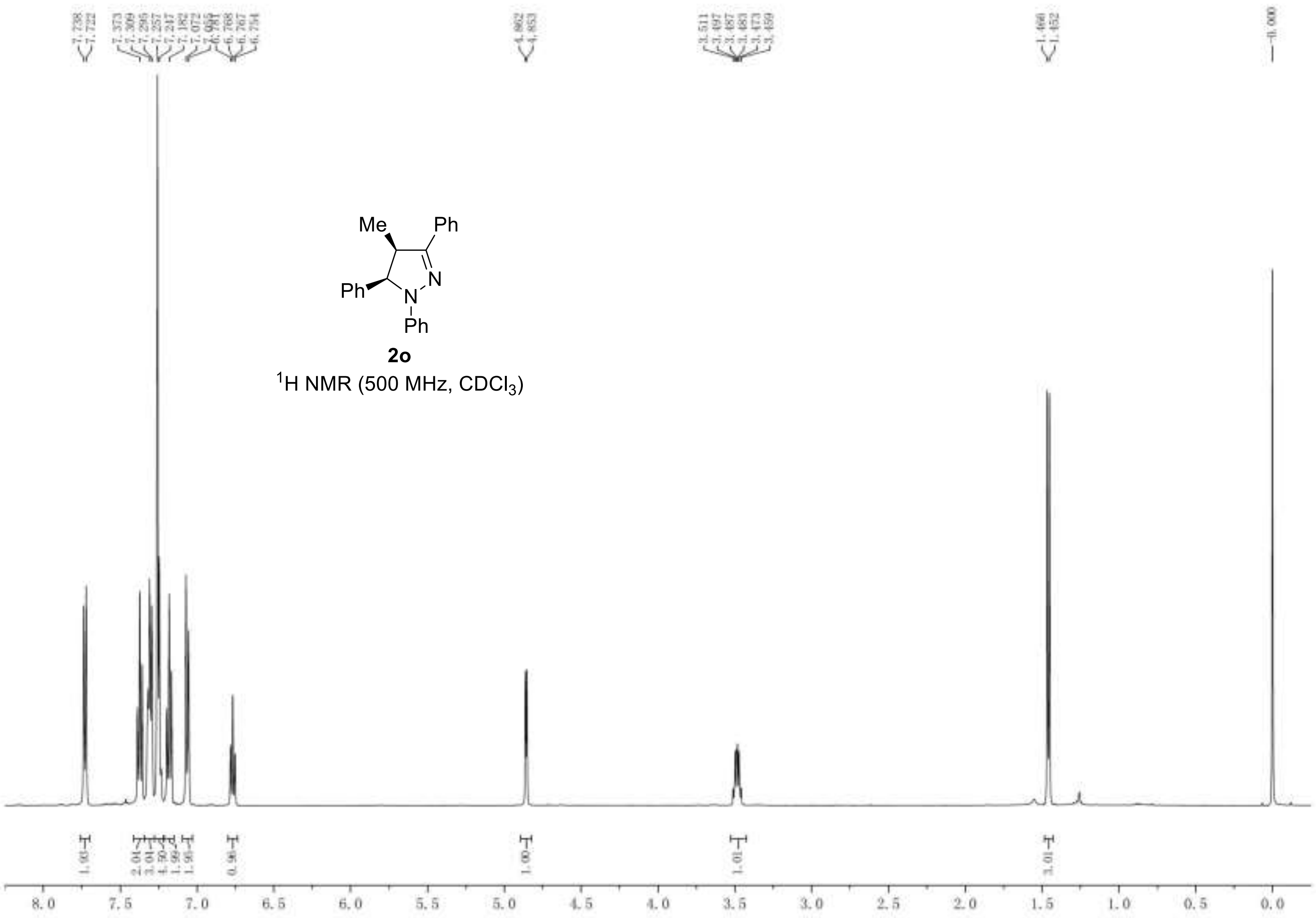

2o

^1H NMR (500 MHz, $CDCl_3$)

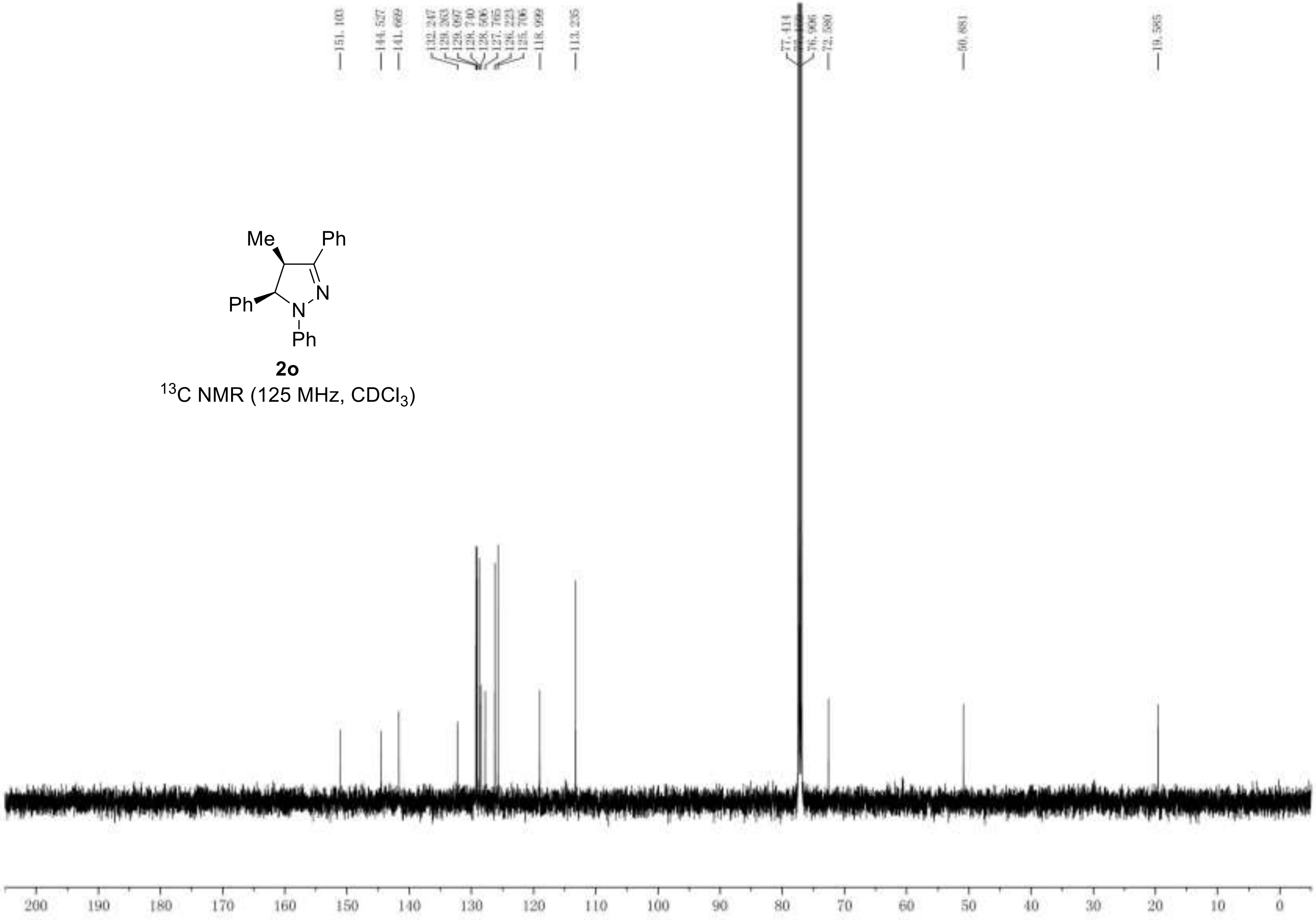

2o

^{13}C NMR (125 MHz, $CDCl_3$)

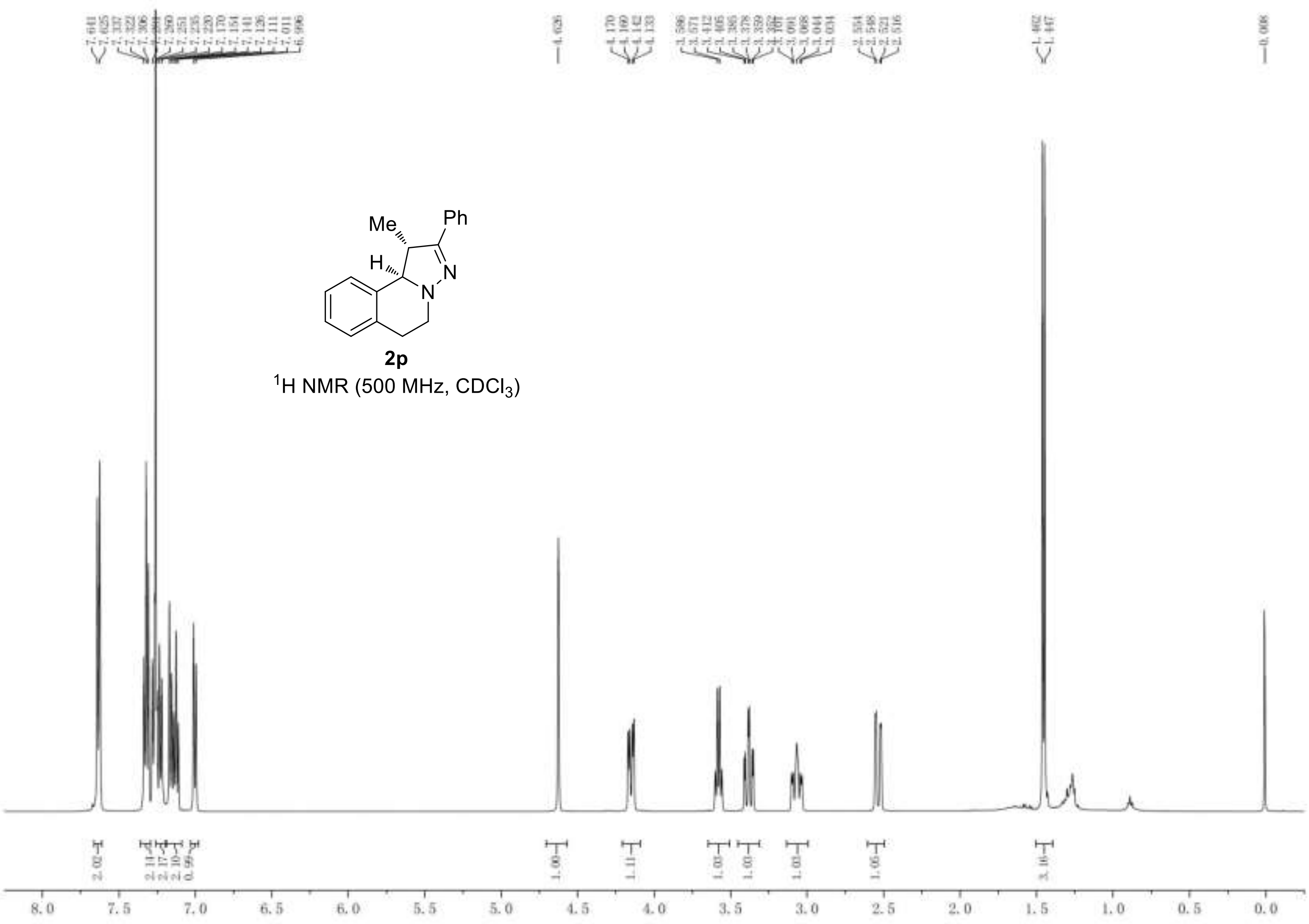

2p

^{1}H NMR (500 MHz, $CDCl_3$)

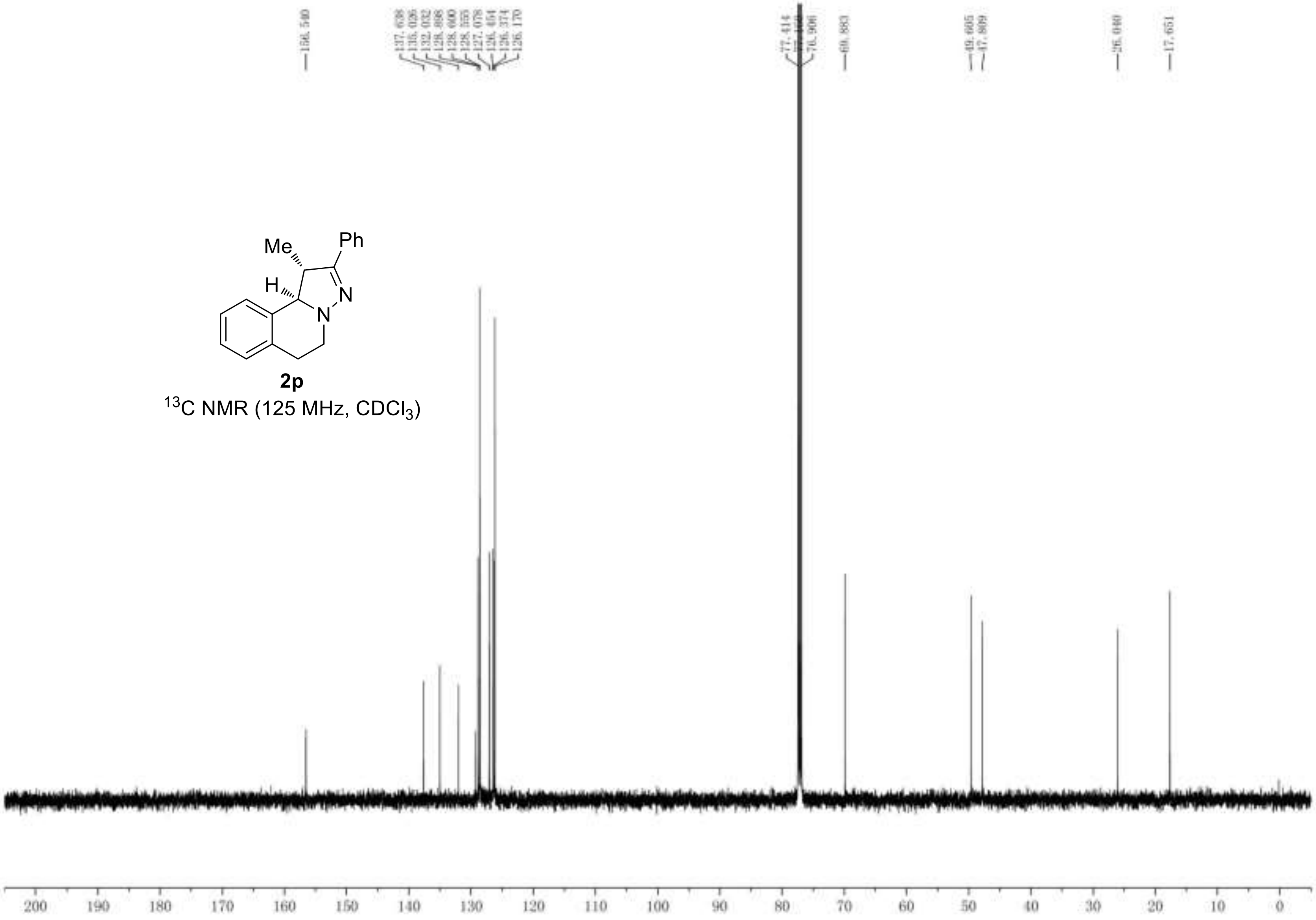
Me
Ph
H
N
N
2p
^{13}C NMR (125 MHz, $CDCl_3$)
156.540
137.638
135.026
132.032
128.898
128.600
128.555
127.078
126.454
126.374
126.170
77.414
76.906
69.883
49.605
47.809
26.040
17.651
200
190
180
170
160
150
140
130
120
110
100
90
80
70
60
50
40
30
20
10
0

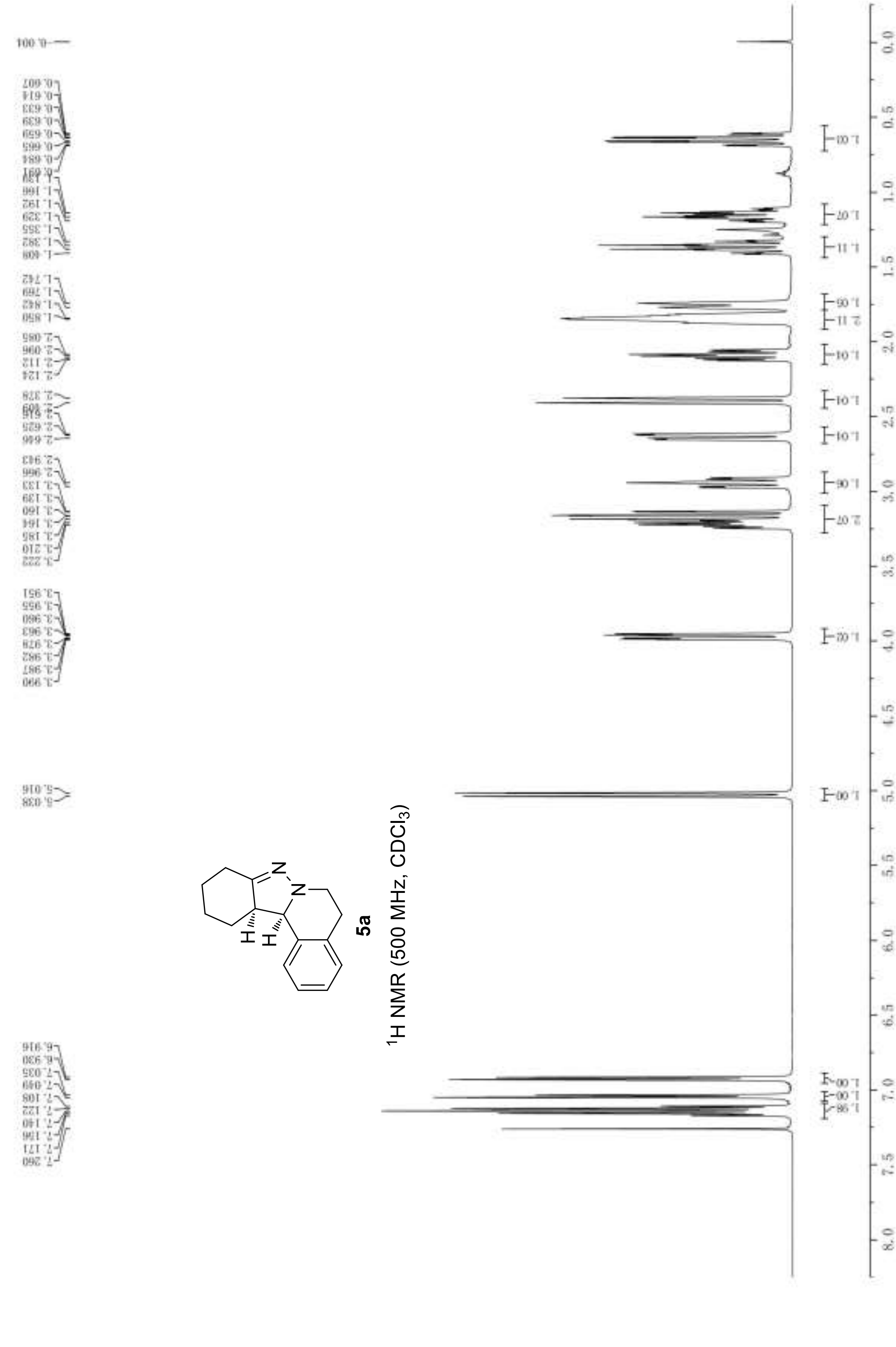
5a
1H NMR (500 MHz, CDCl3)

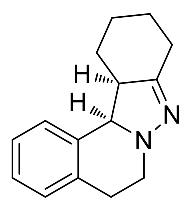

5a

^{13}C NMR (125 MHz, $CDCl_3$)

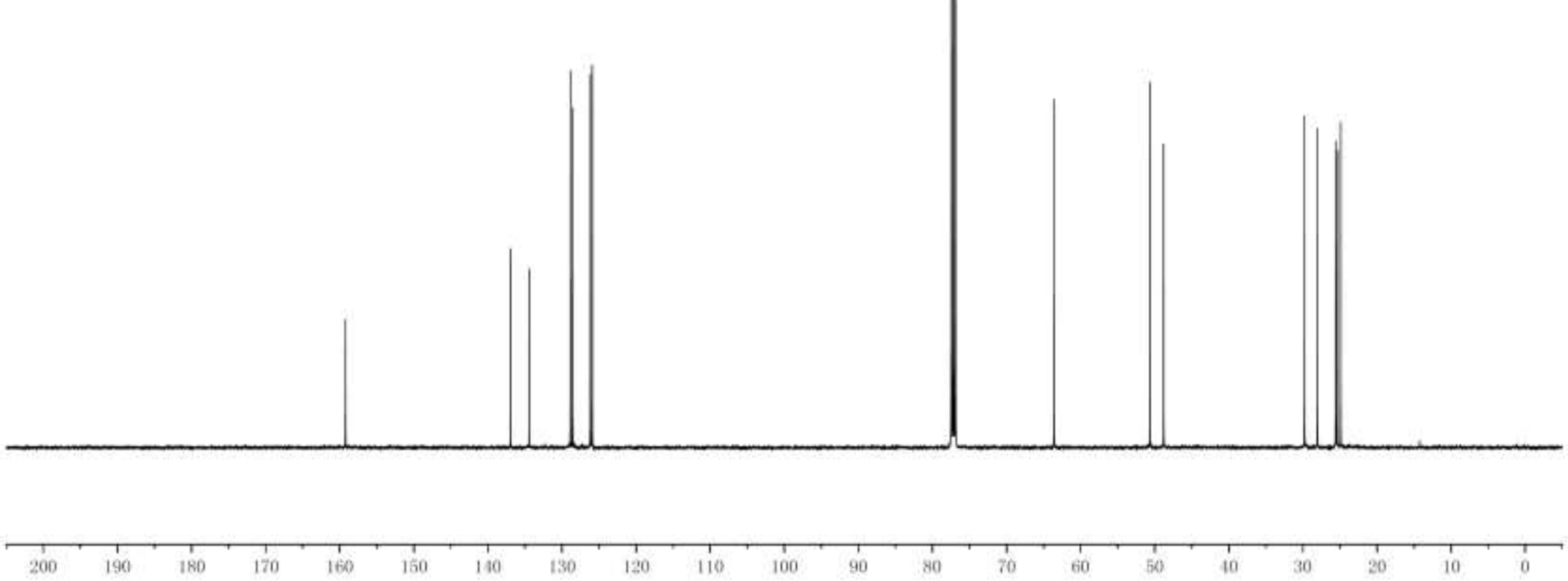

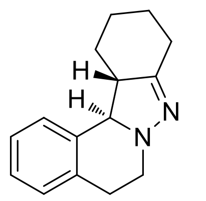

***anti*-5a**

^1H NMR (500 MHz, $CDCl_3$)

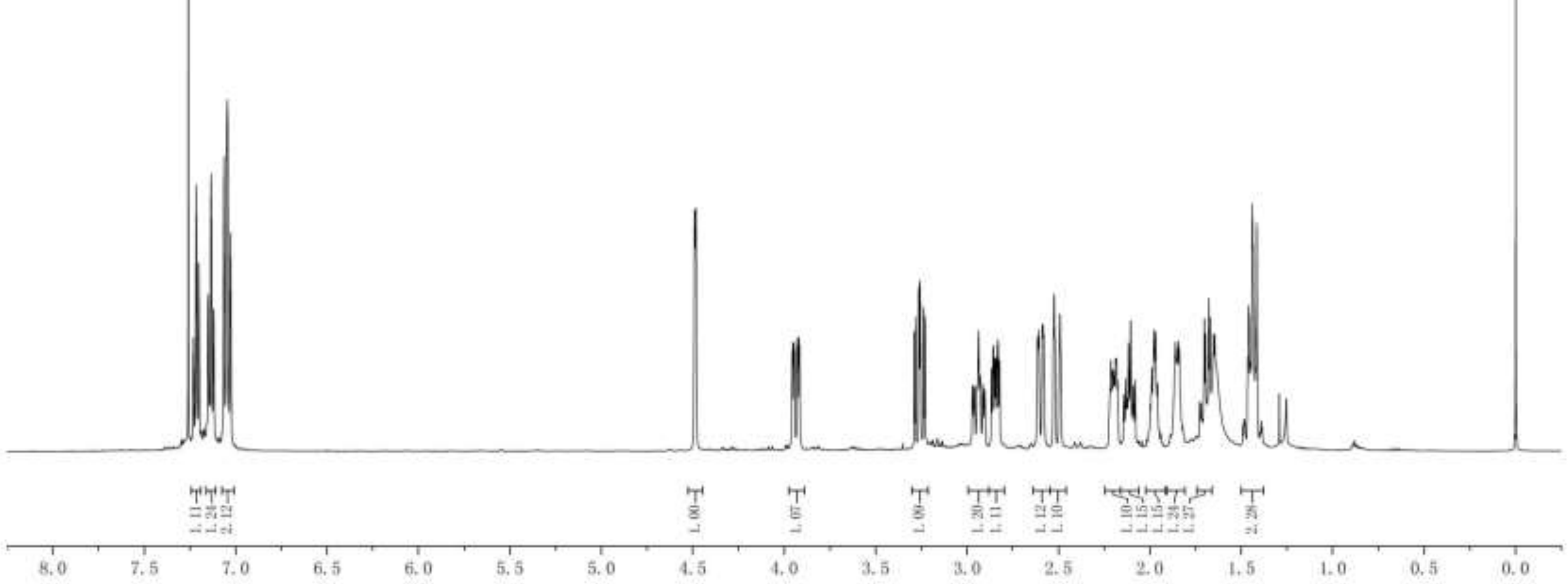

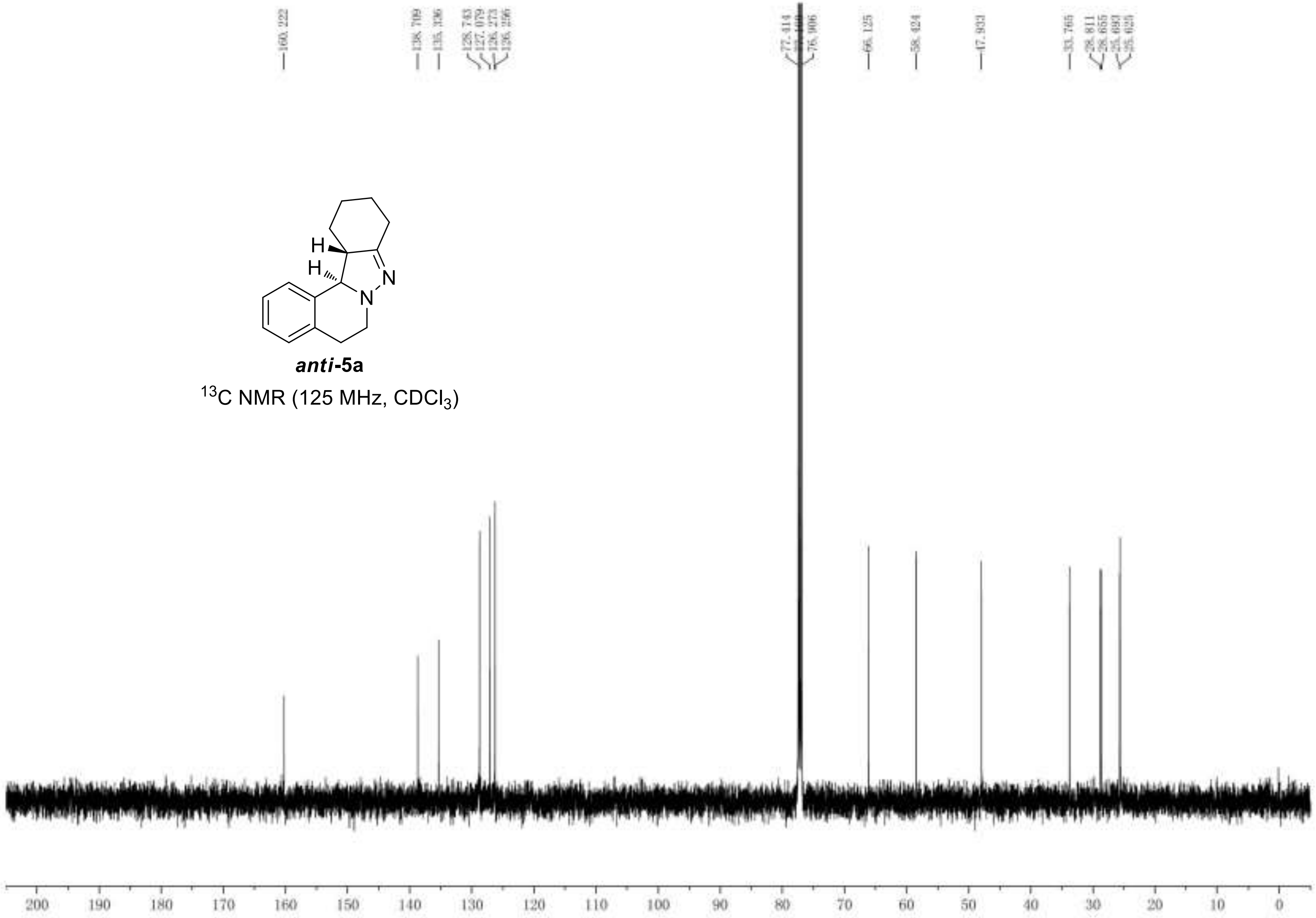

***anti*-5a**

^{13}C NMR (125 MHz, $CDCl_3$)

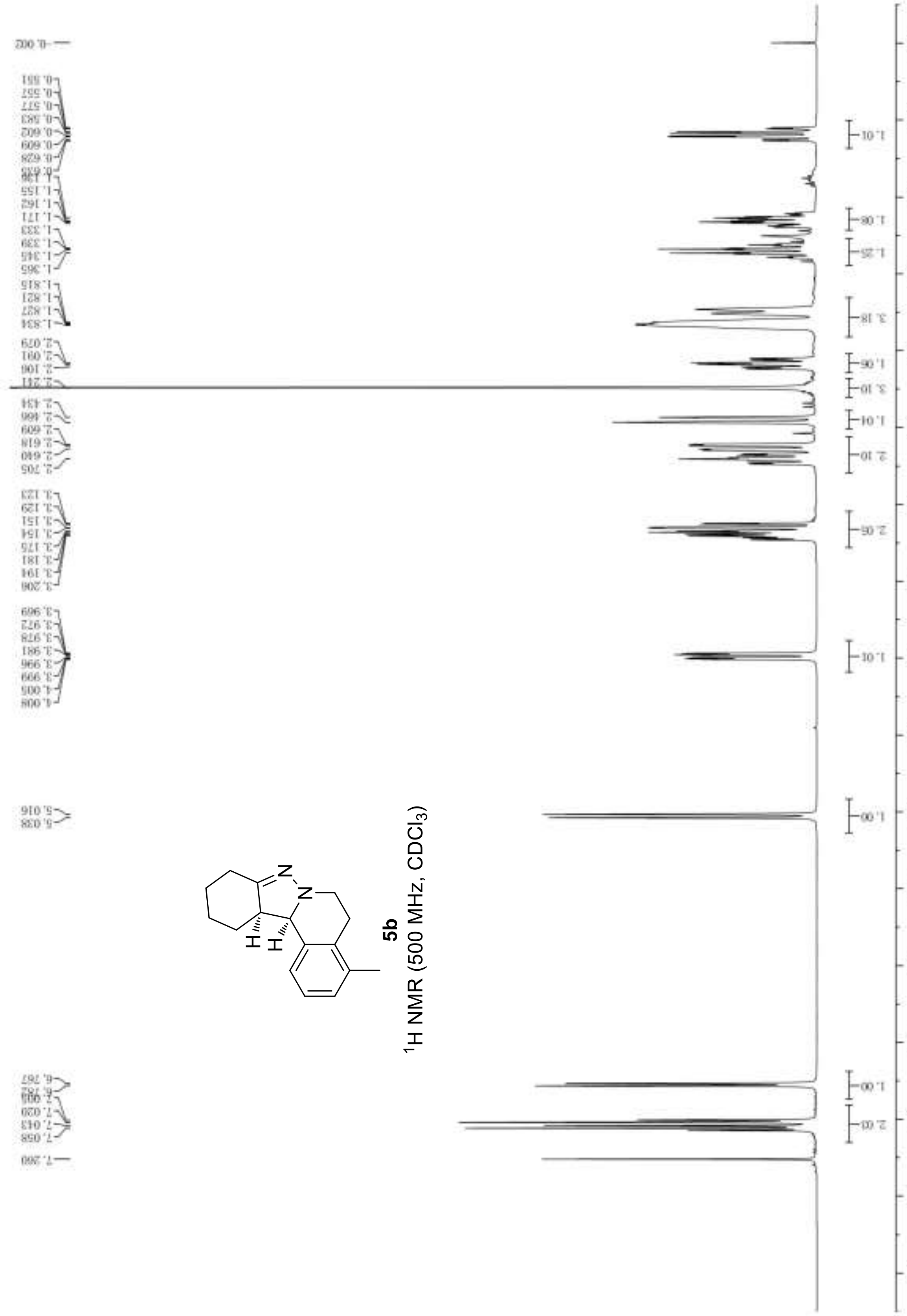

5b

^1H NMR (500 MHz, $CDCl_3$)

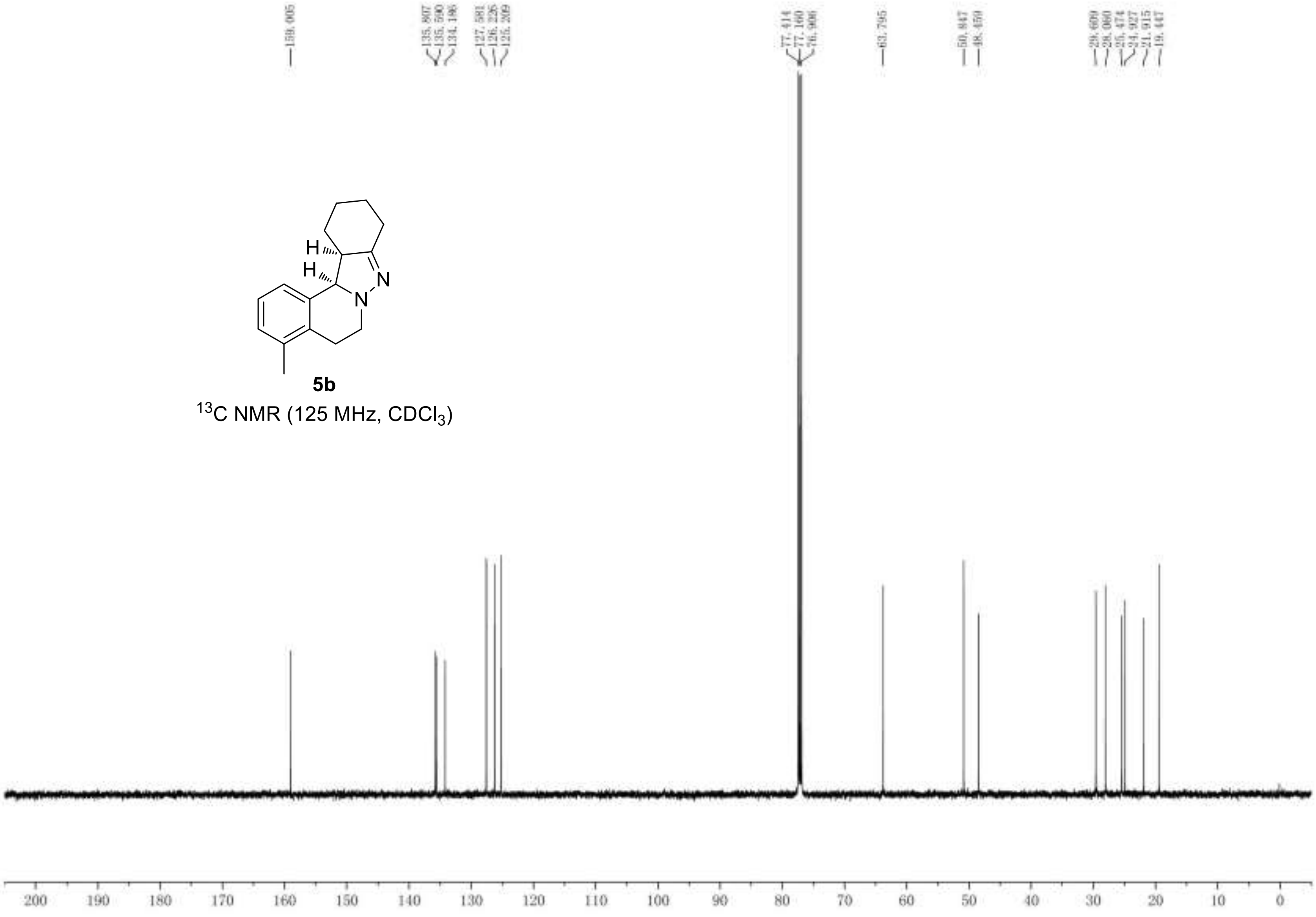

5b

^{13}C NMR (125 MHz, $CDCl_3$)

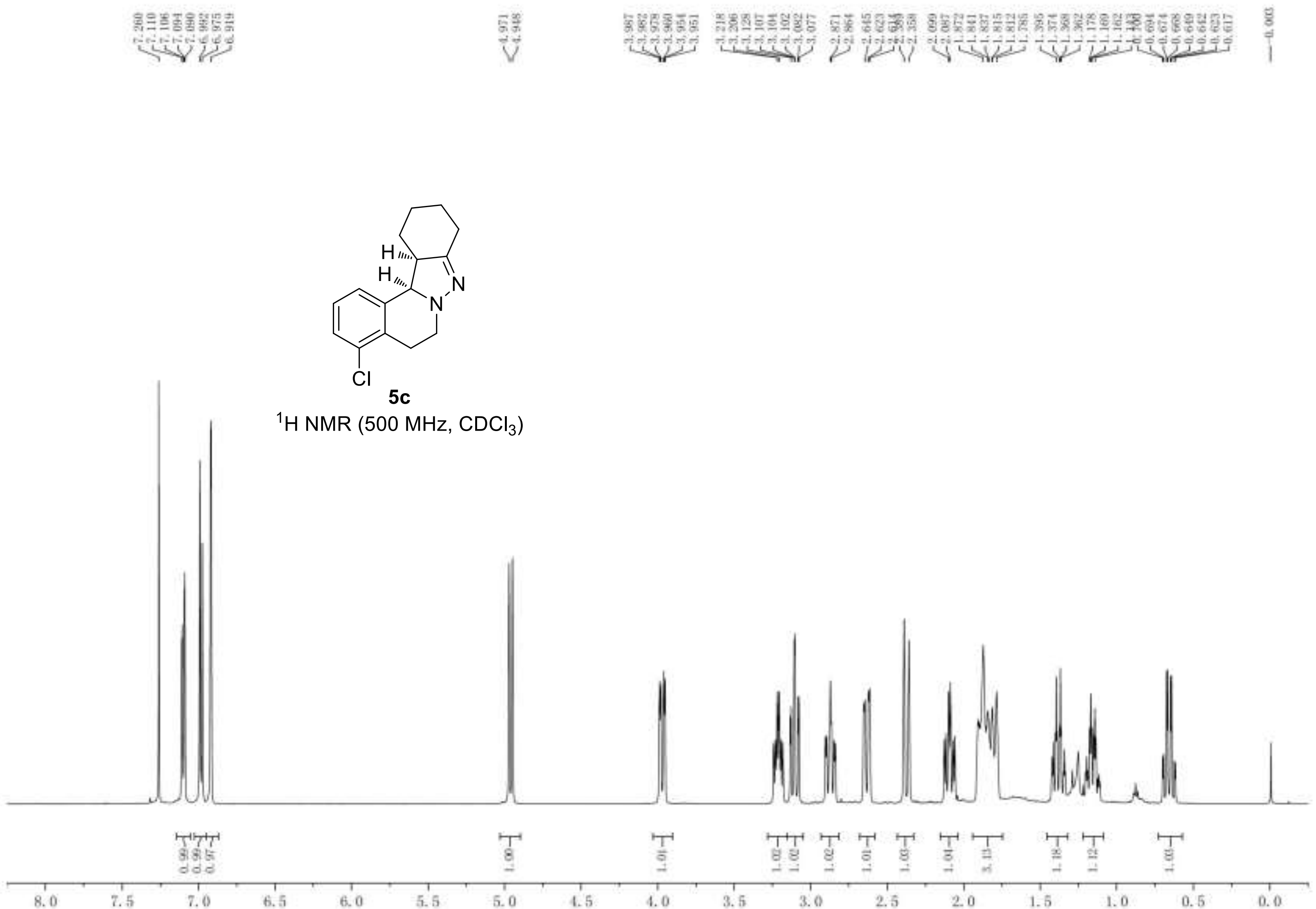

5c

^1H NMR (500 MHz, $CDCl_3$)

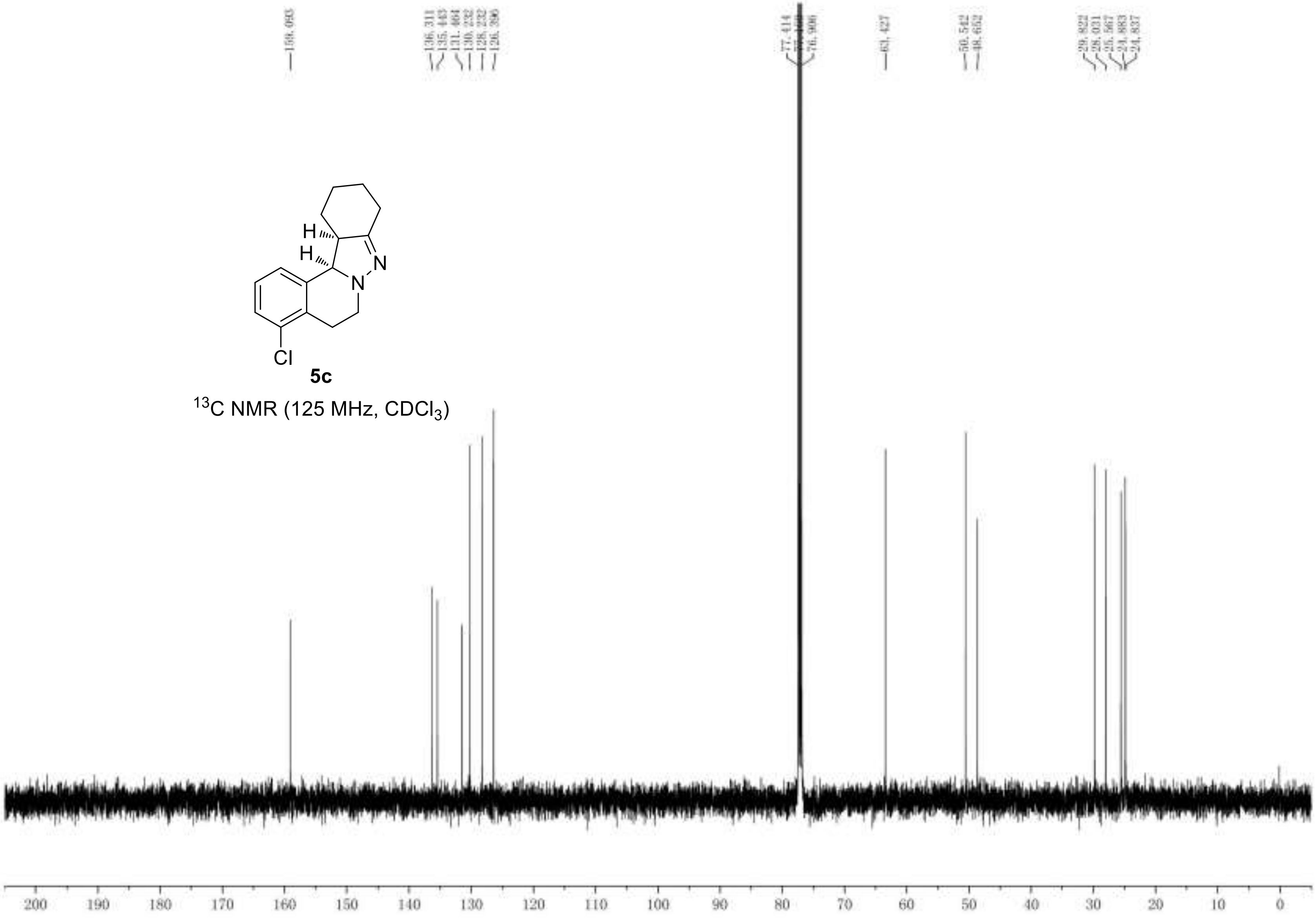

5c

^{13}C NMR (125 MHz, $CDCl_3$)

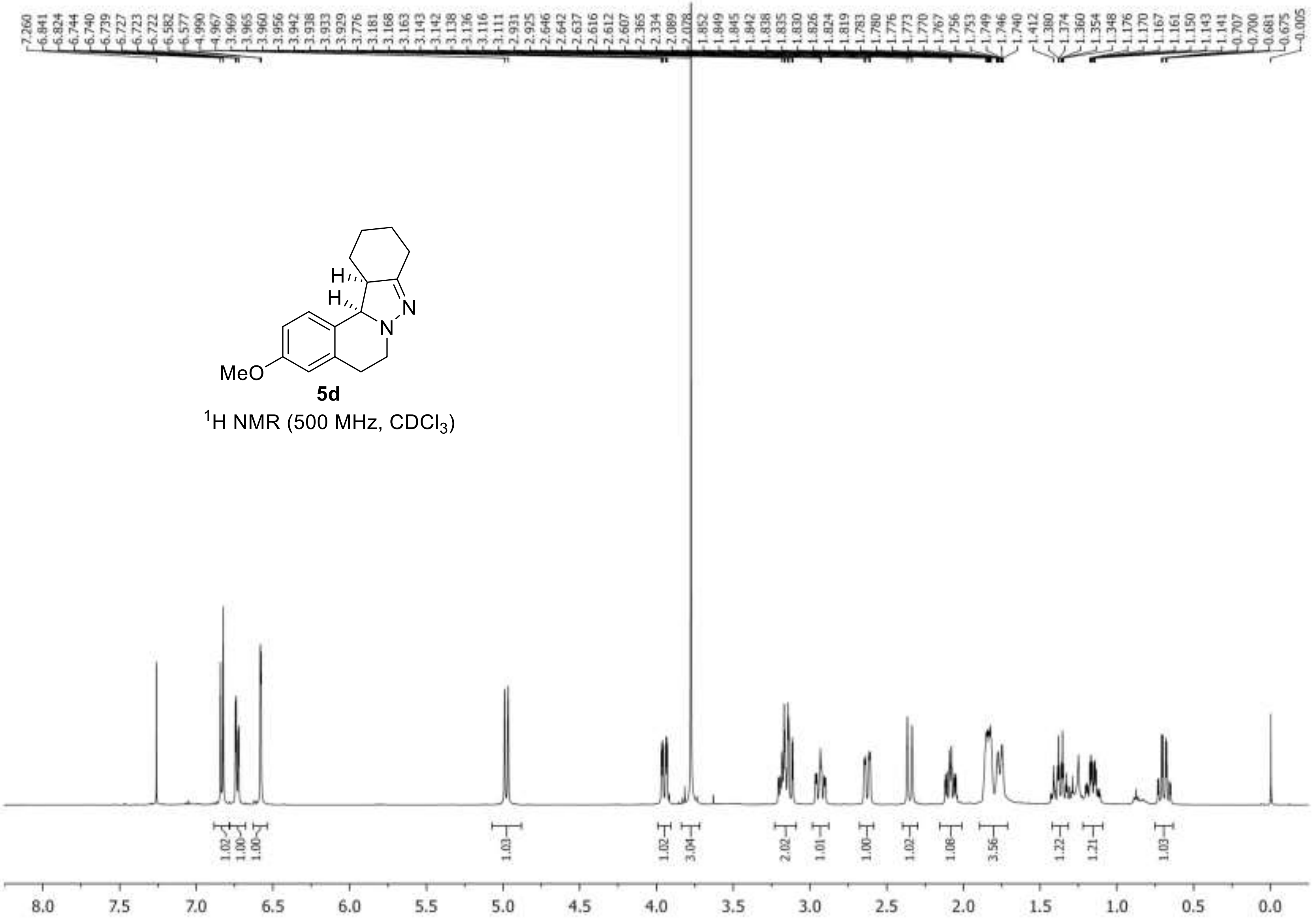

5d

^1H NMR (500 MHz, $CDCl_3$)

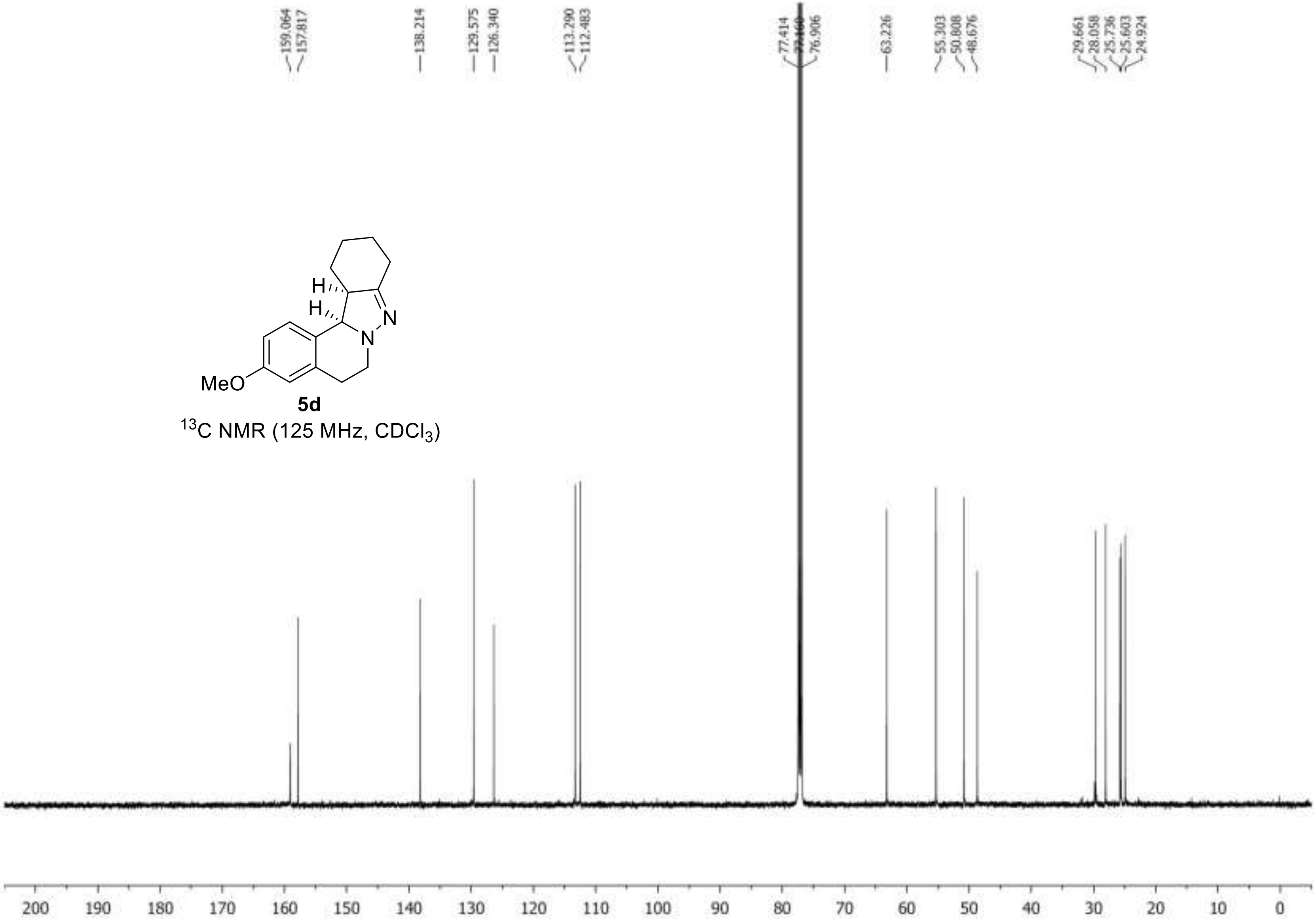

5d

^{13}C NMR (125 MHz, $CDCl_3$)

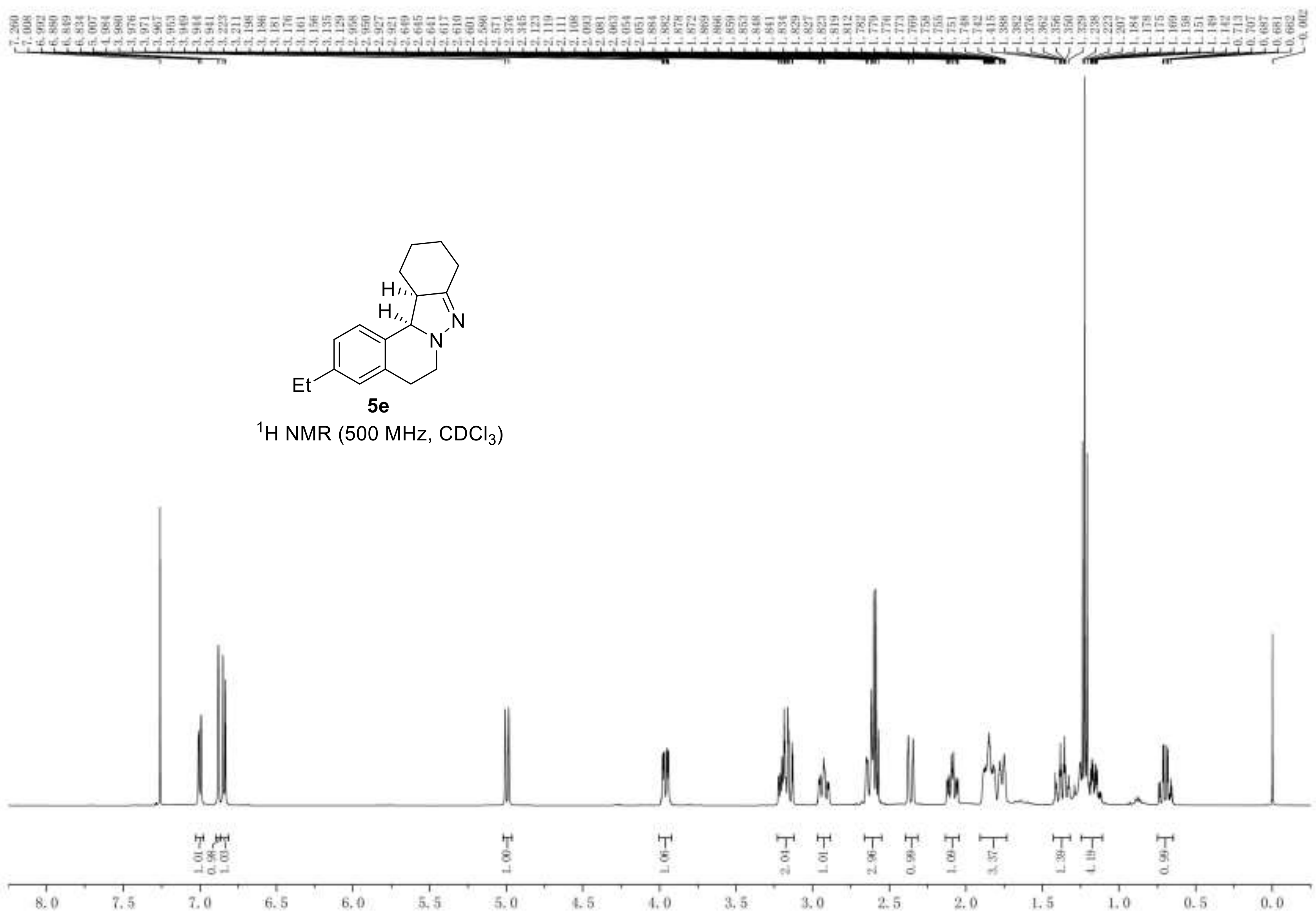

5e

^{1}H NMR (500 MHz, $CDCl_3$)

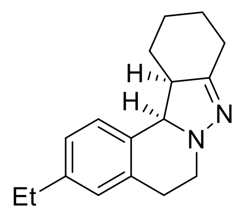

5e

^{13}C NMR (125 MHz, $CDCl_3$)

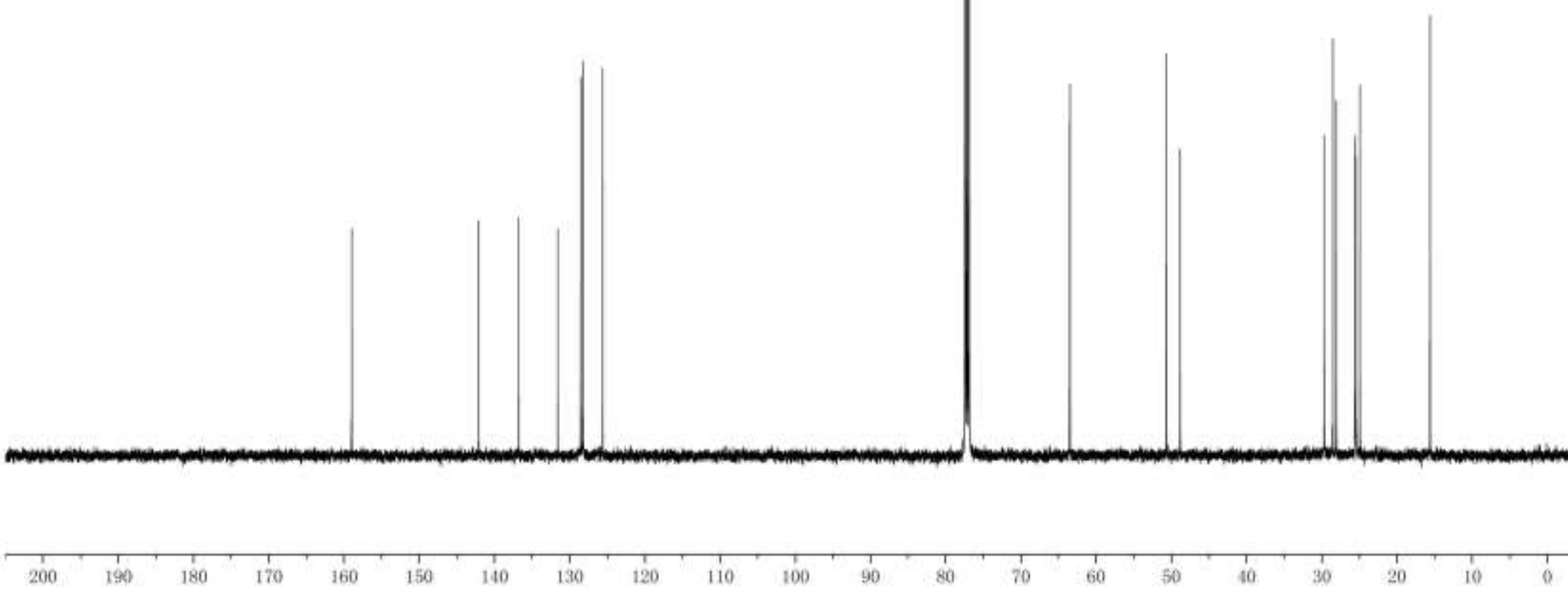

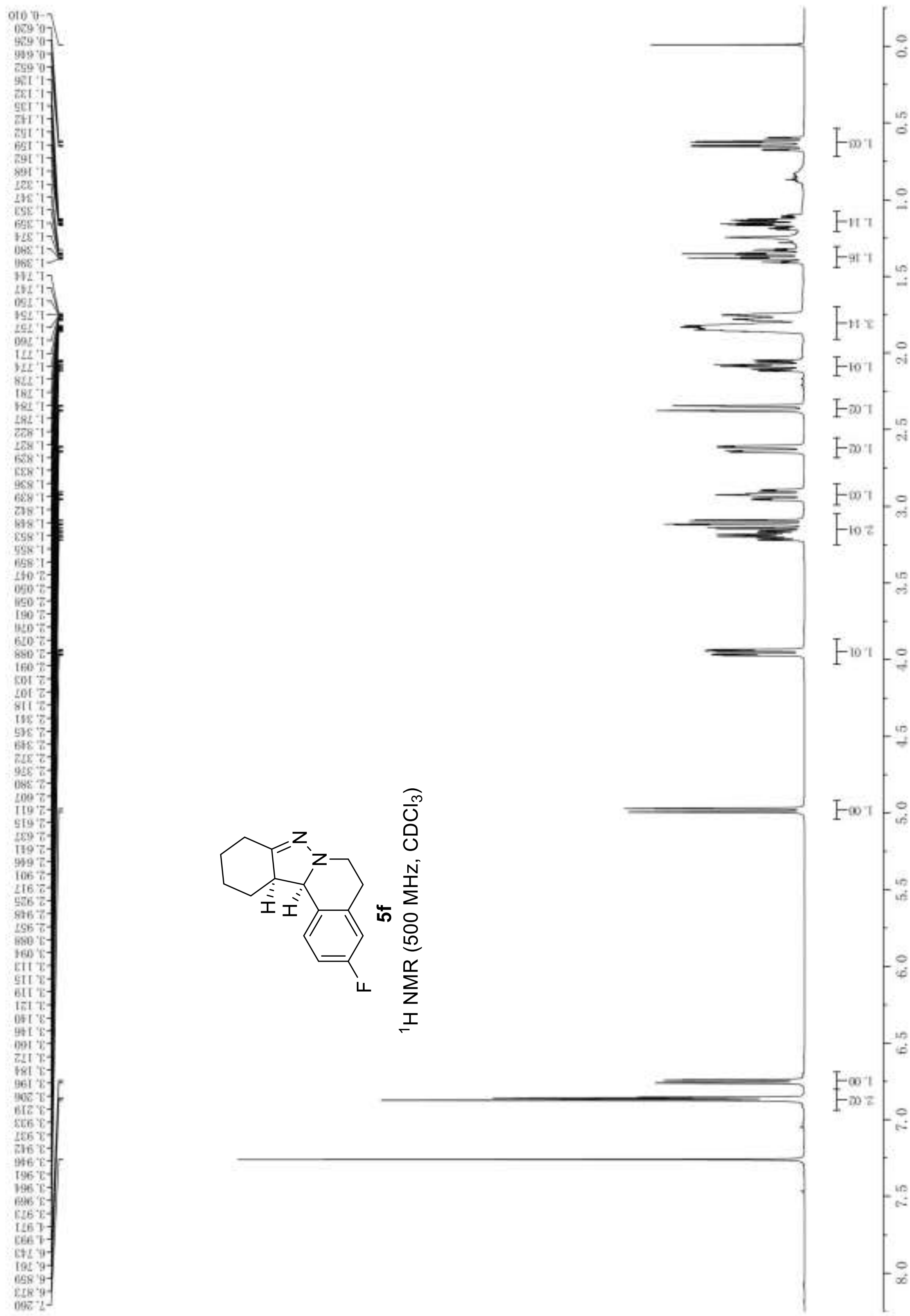

5f

^{1}H NMR (500 MHz, $CDCl_3$)

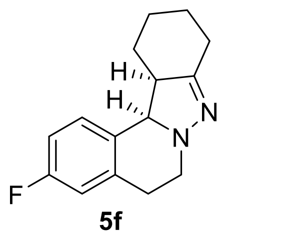

5f

^{13}C NMR (125 MHz, $CDCl_3$)

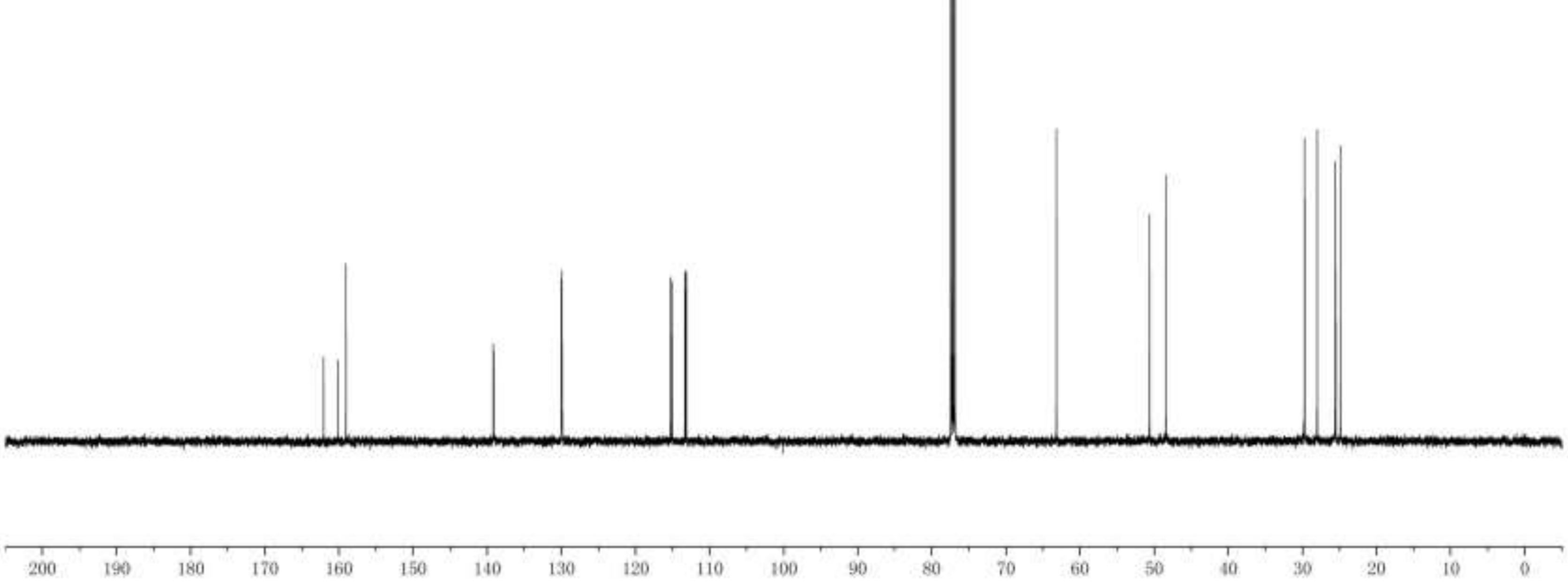

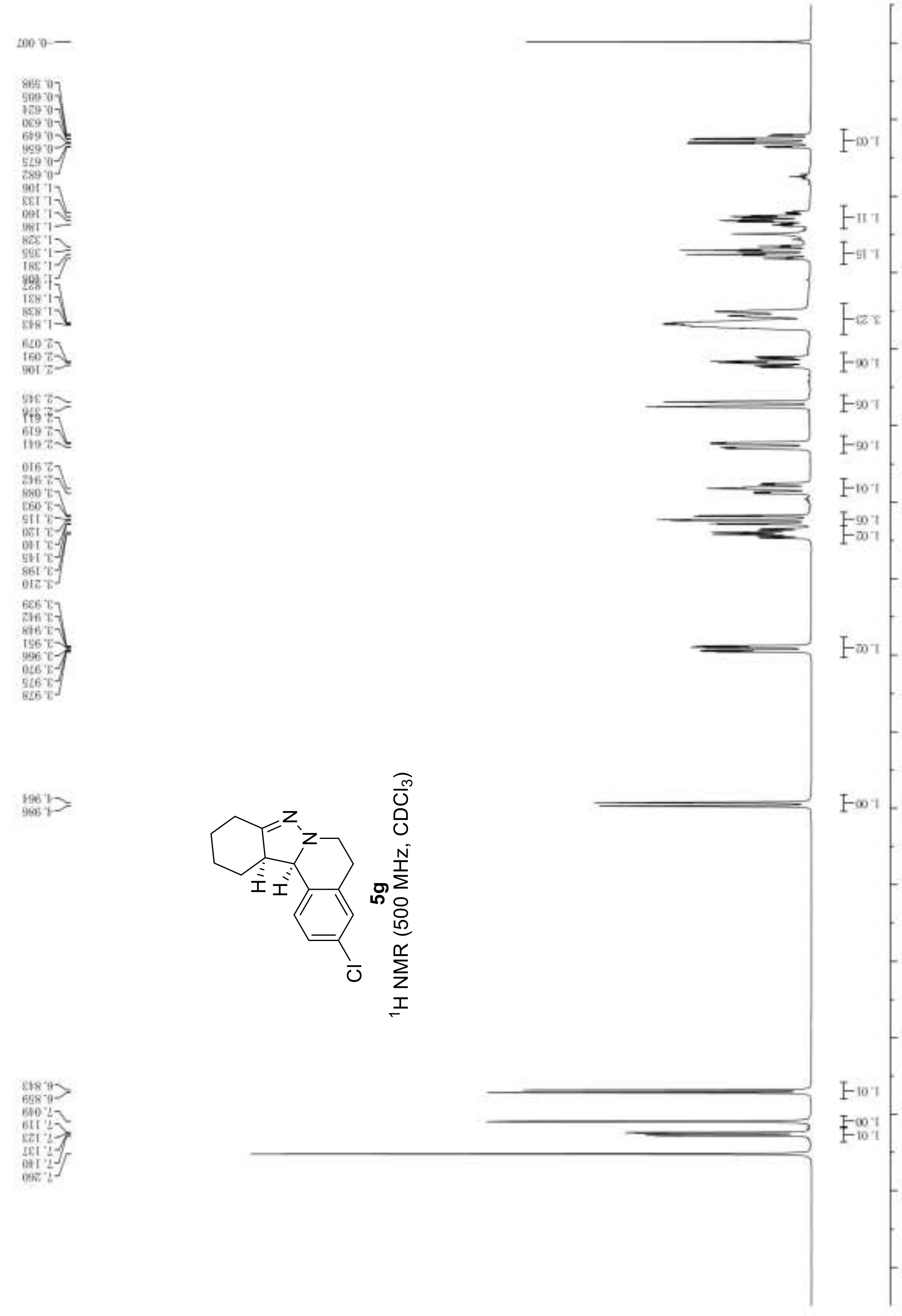

Cl
N
N
H
H
5g
^{1}H NMR (500 MHz, CDCl$_3$)

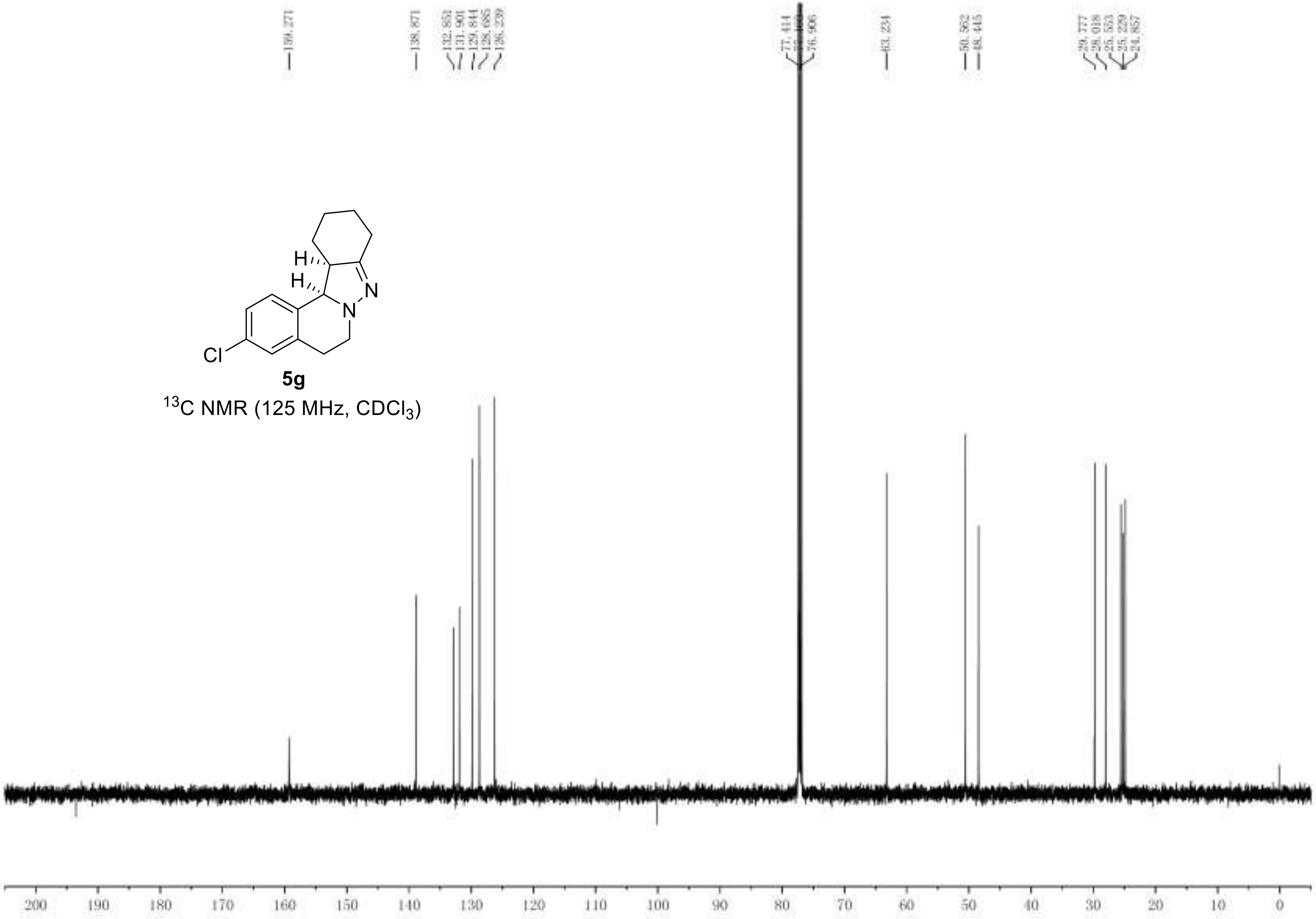

5g

^{13}C NMR (125 MHz, $CDCl_3$)

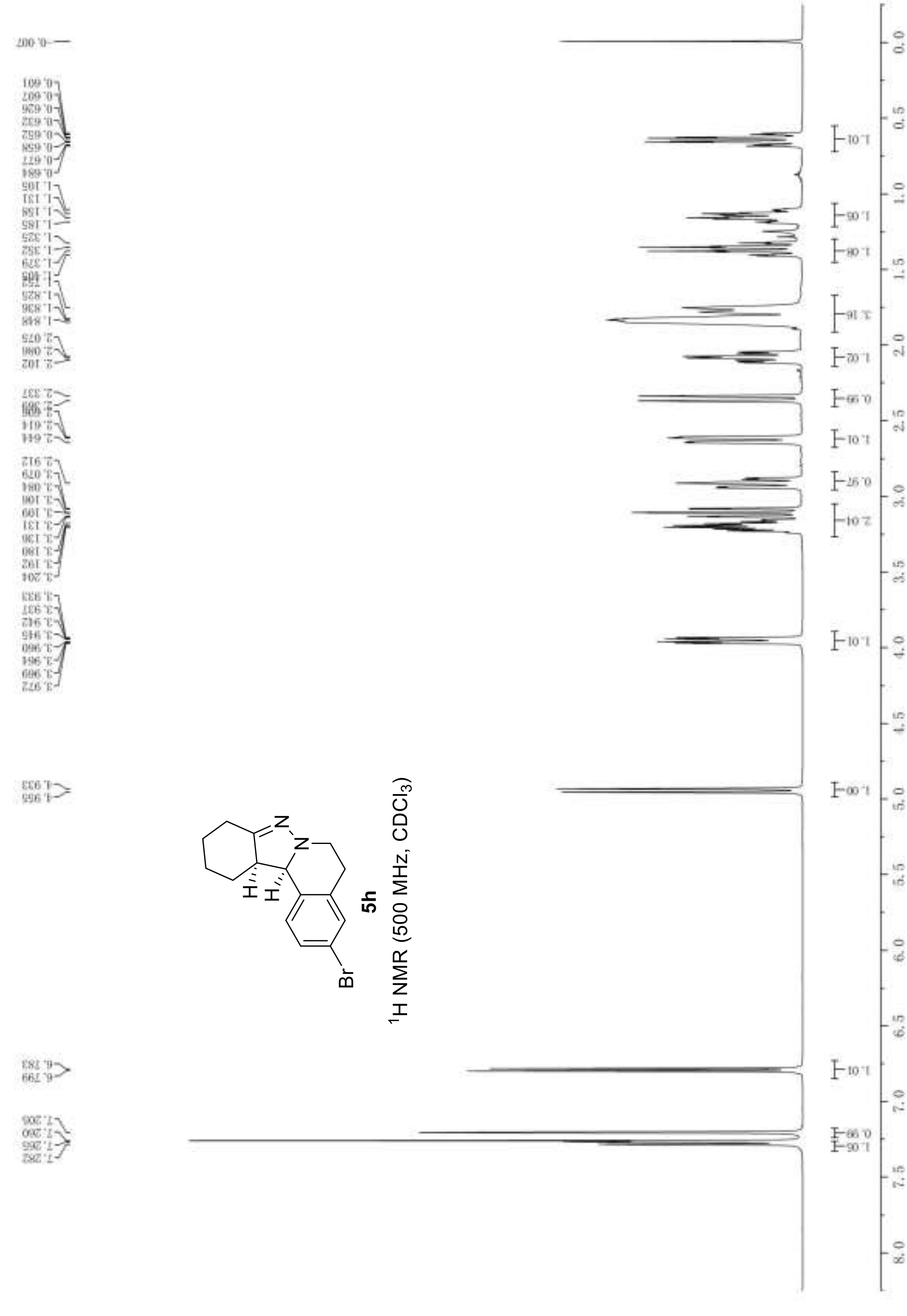

5h

^1H NMR (500 MHz, $CDCl_3$)

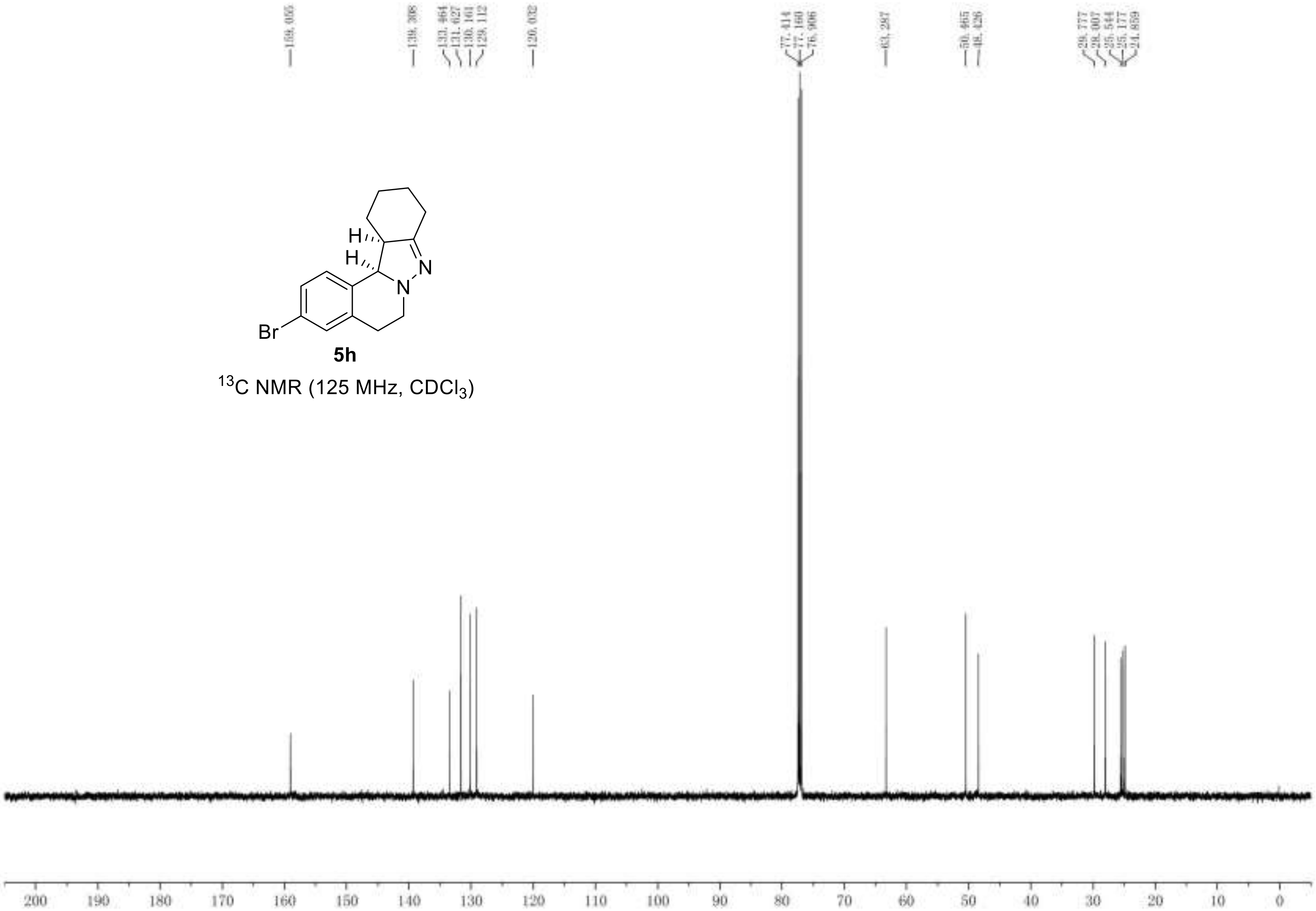

5h

^{13}C NMR (125 MHz, $CDCl_3$)

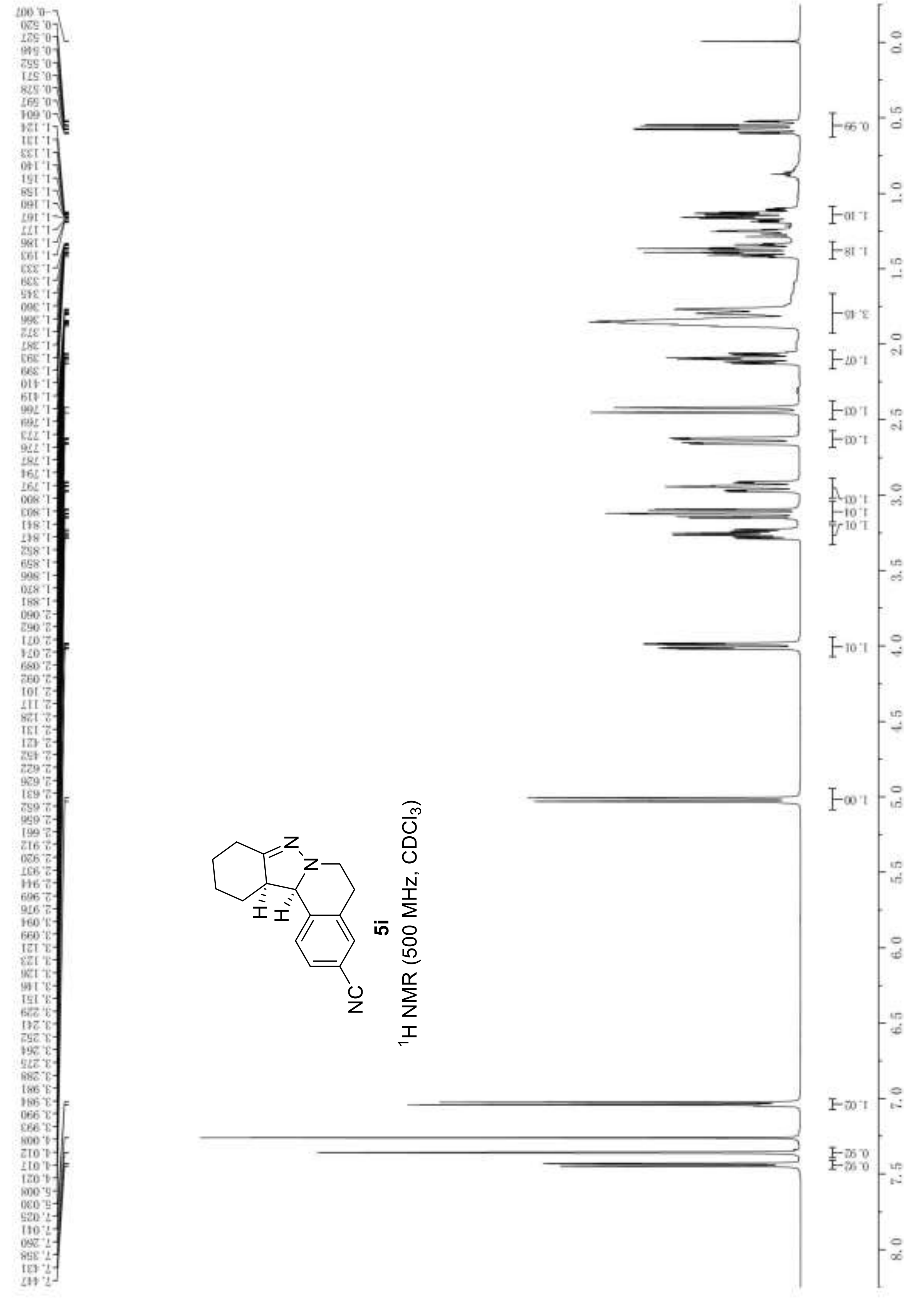
5i
1H NMR (500 MHz, CDCl3)

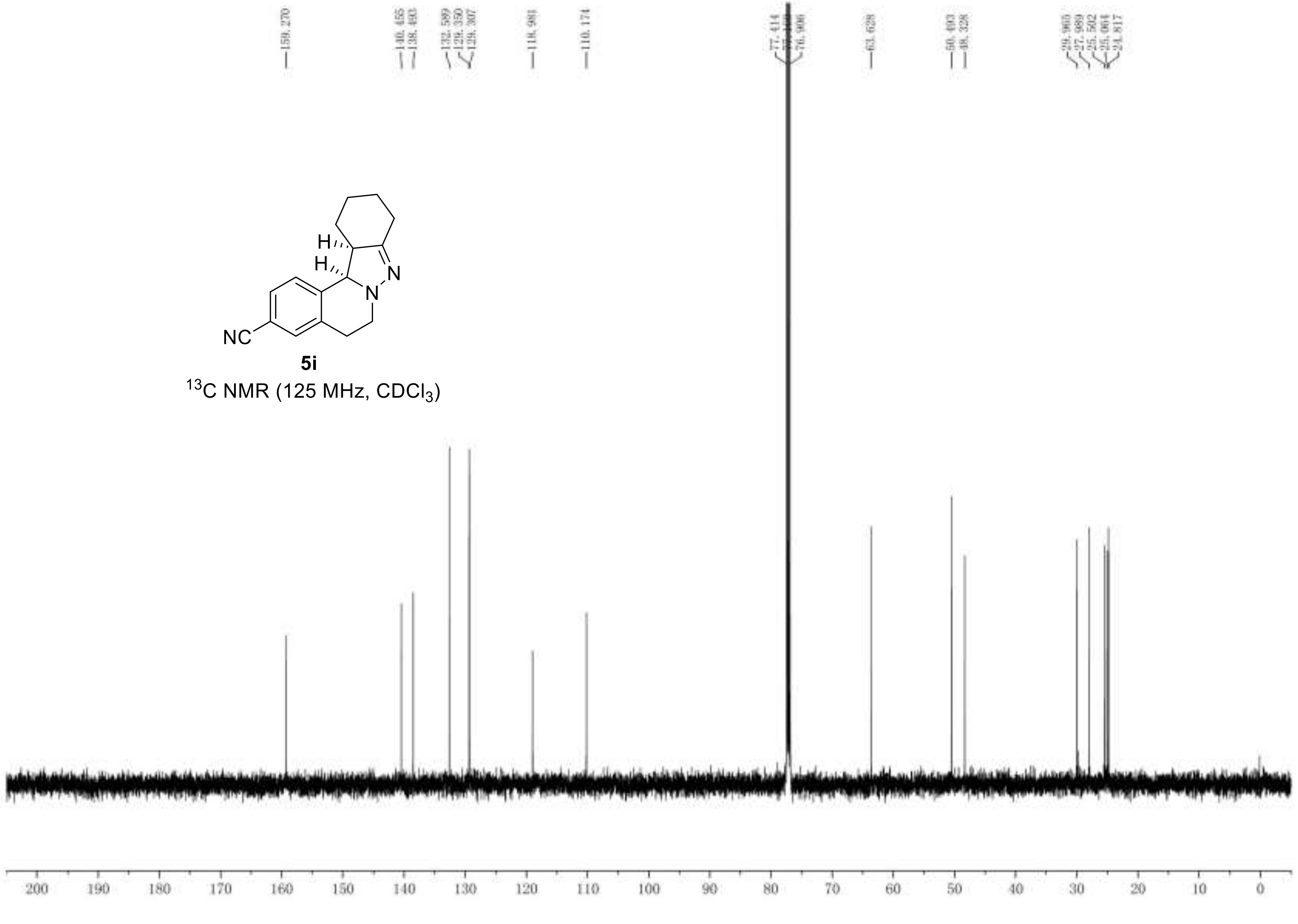

5i

^{13}C NMR (125 MHz, $CDCl_3$)

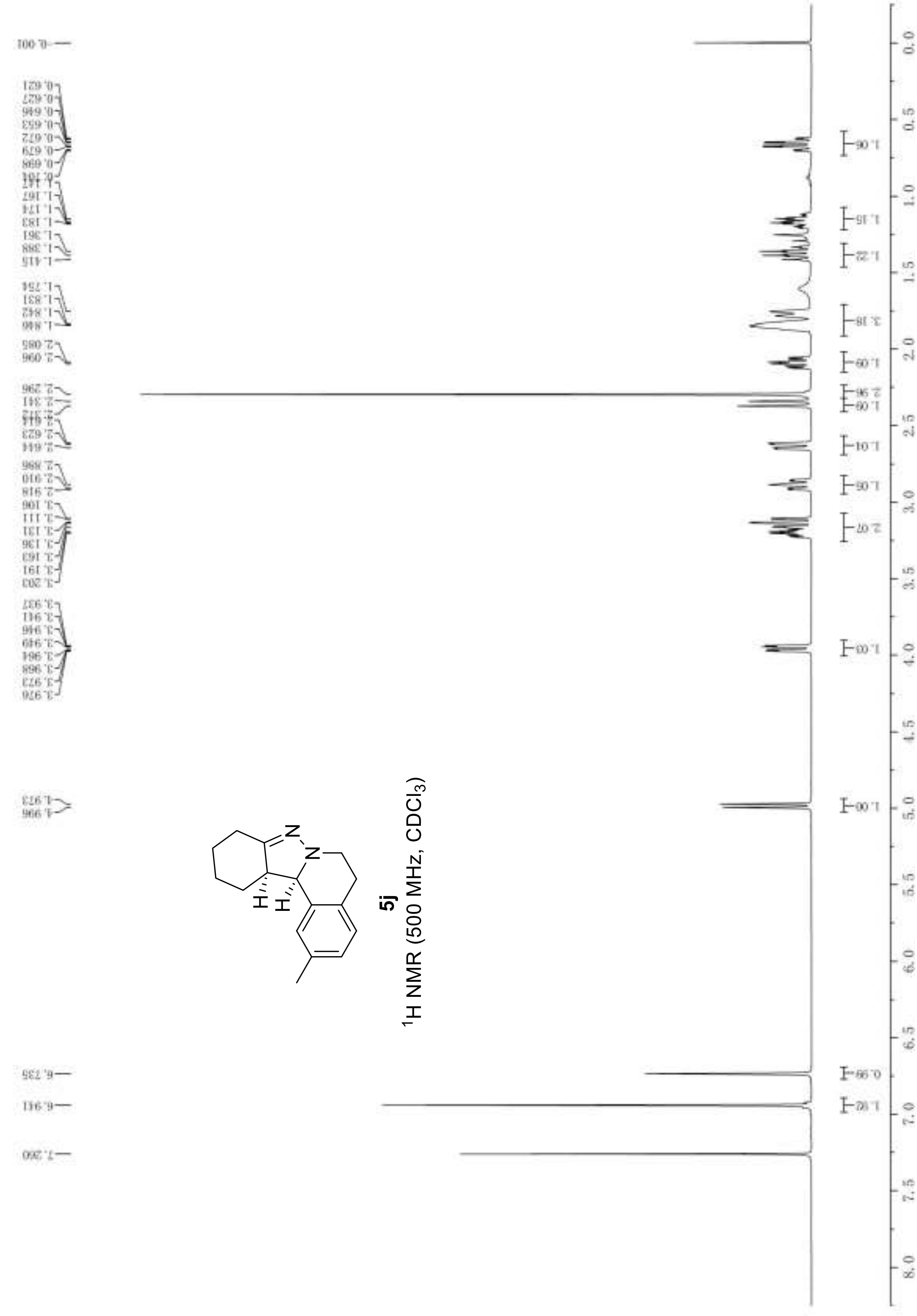

5j

^1H NMR (500 MHz, $CDCl_3$)

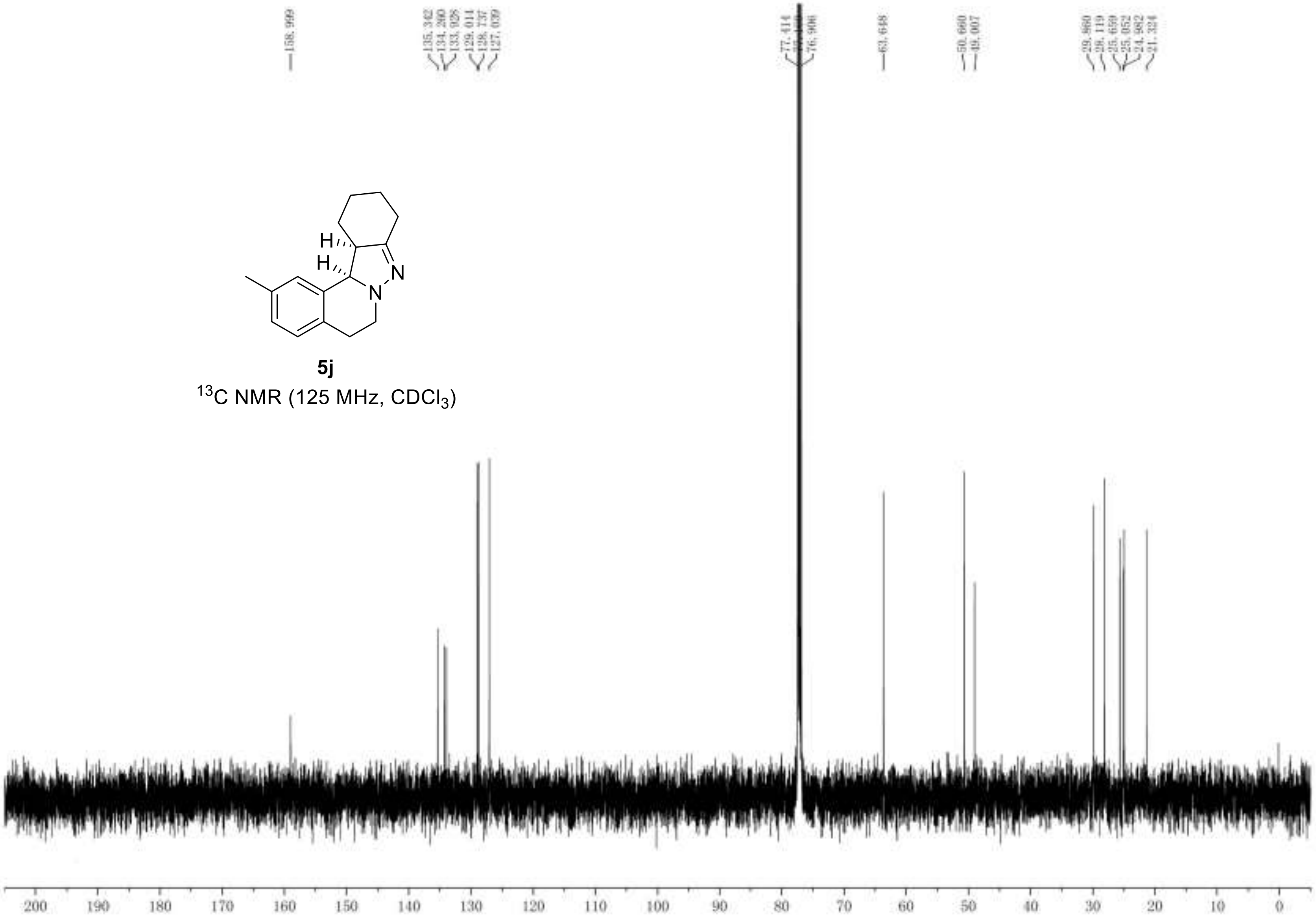

5j

^{13}C NMR (125 MHz, $CDCl_3$)

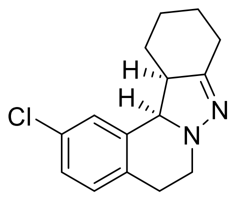

5k

^1H NMR (500 MHz, $CDCl_3$)

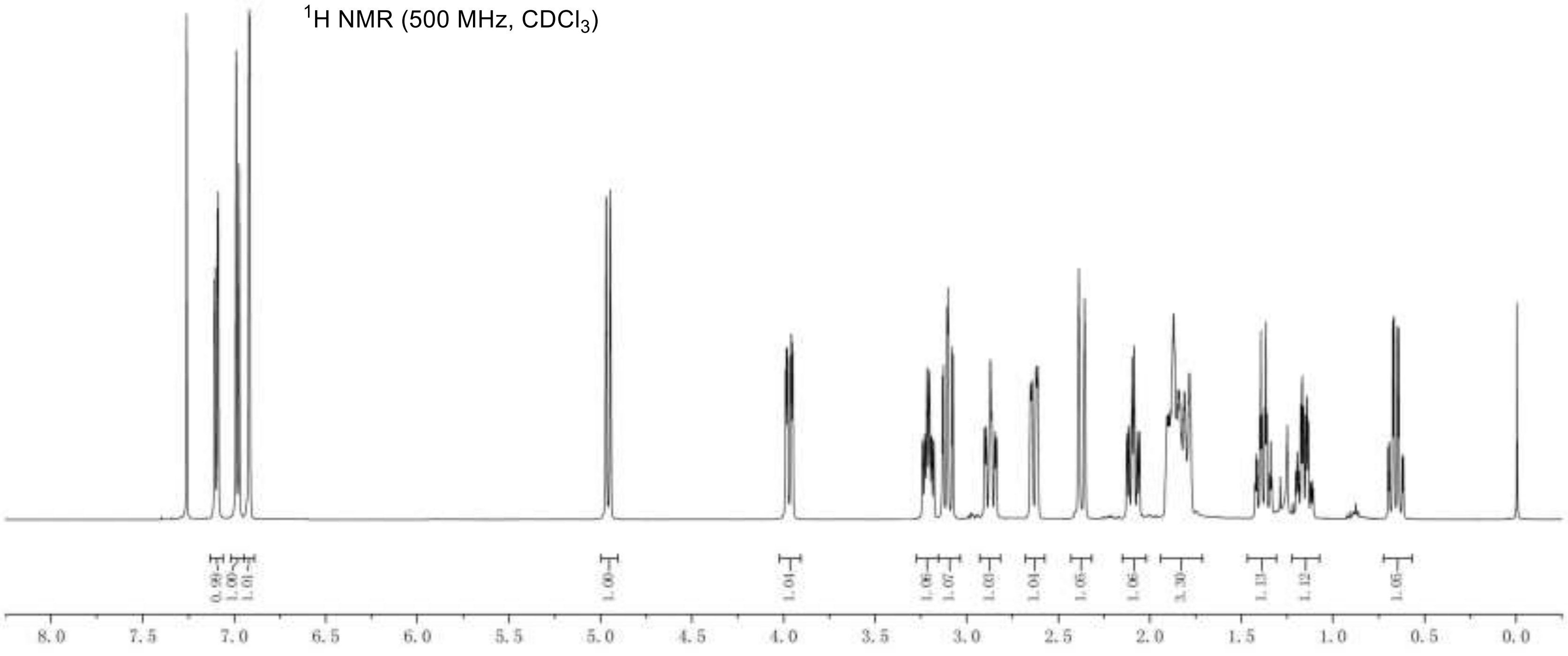

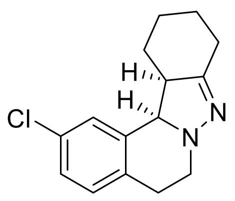

5k

^{13}C NMR (125 MHz, $CDCl_3$)

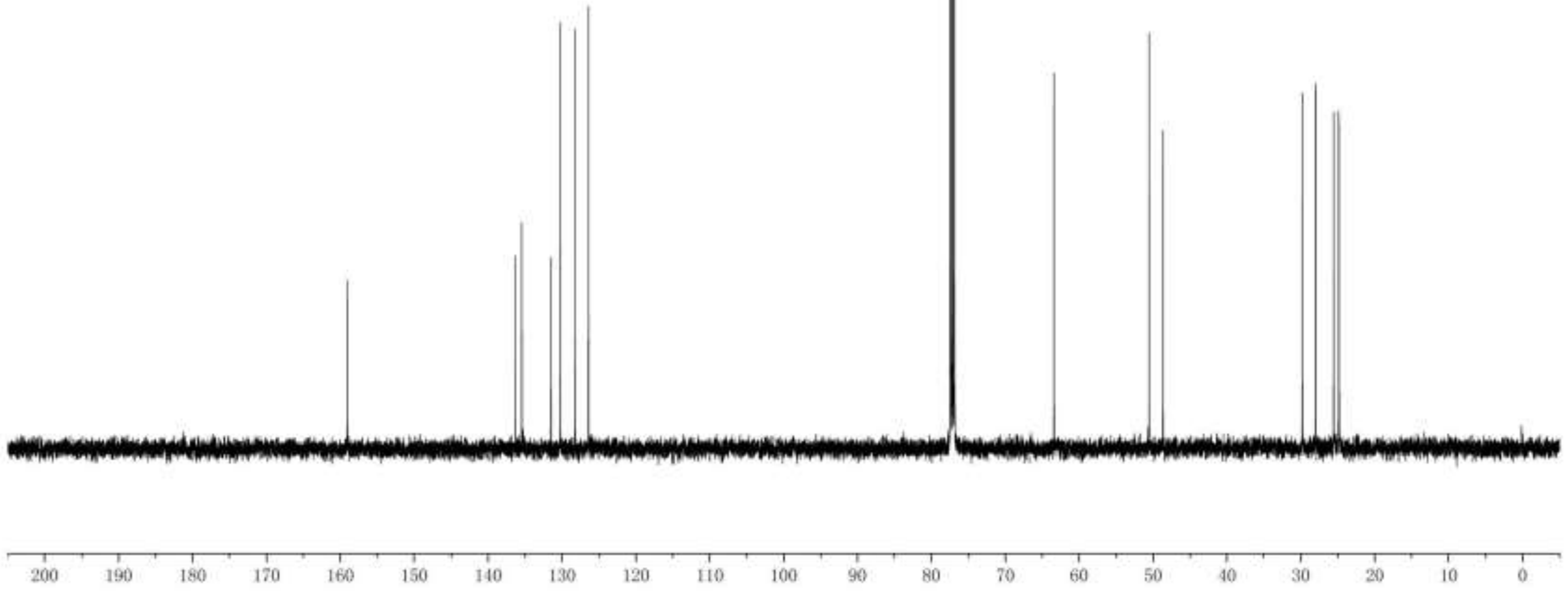

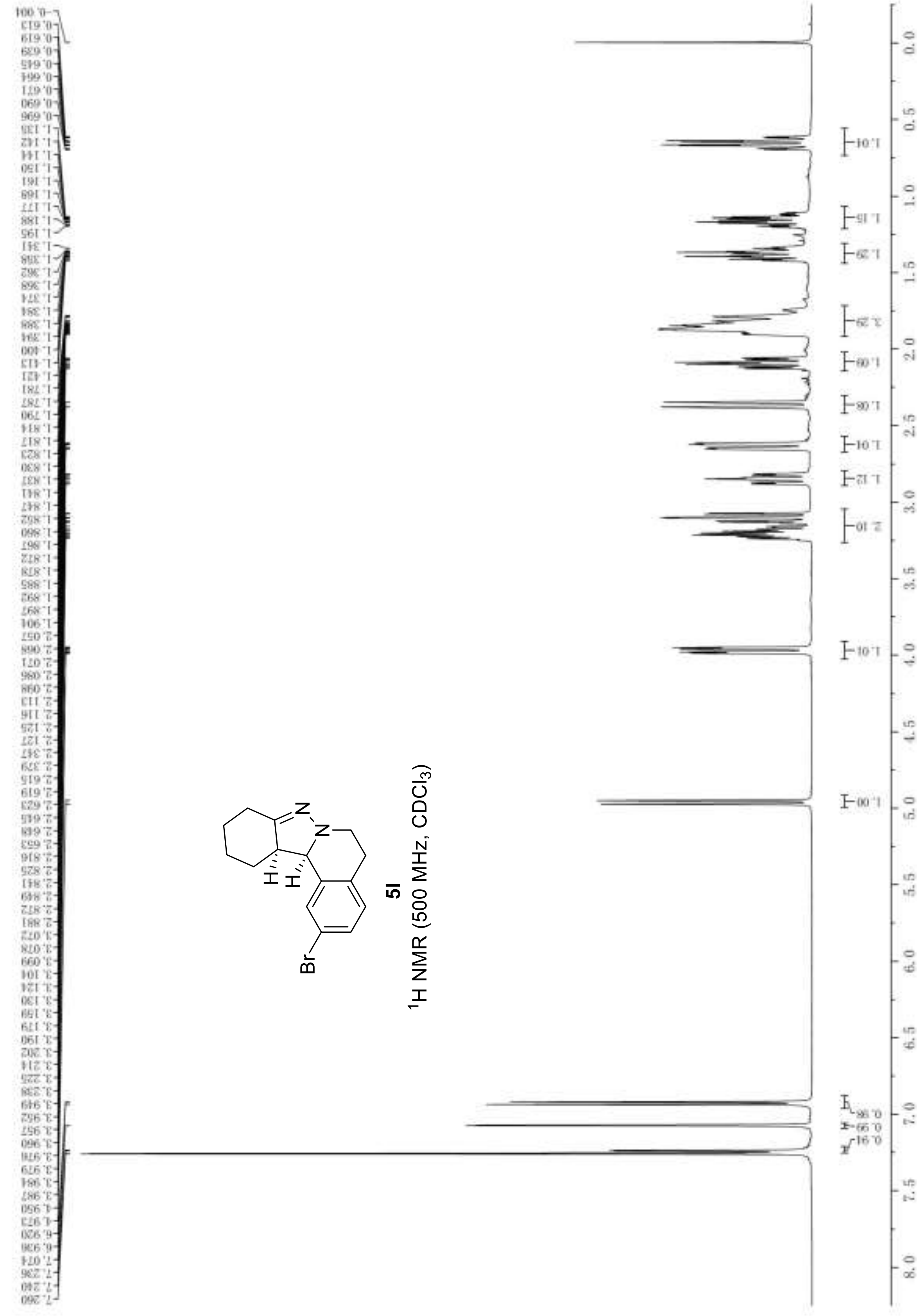
5l
1H NMR (500 MHz, CDCl3)

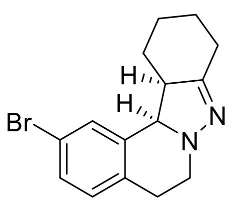

5l

^{13}C NMR (125 MHz, $CDCl_3$)

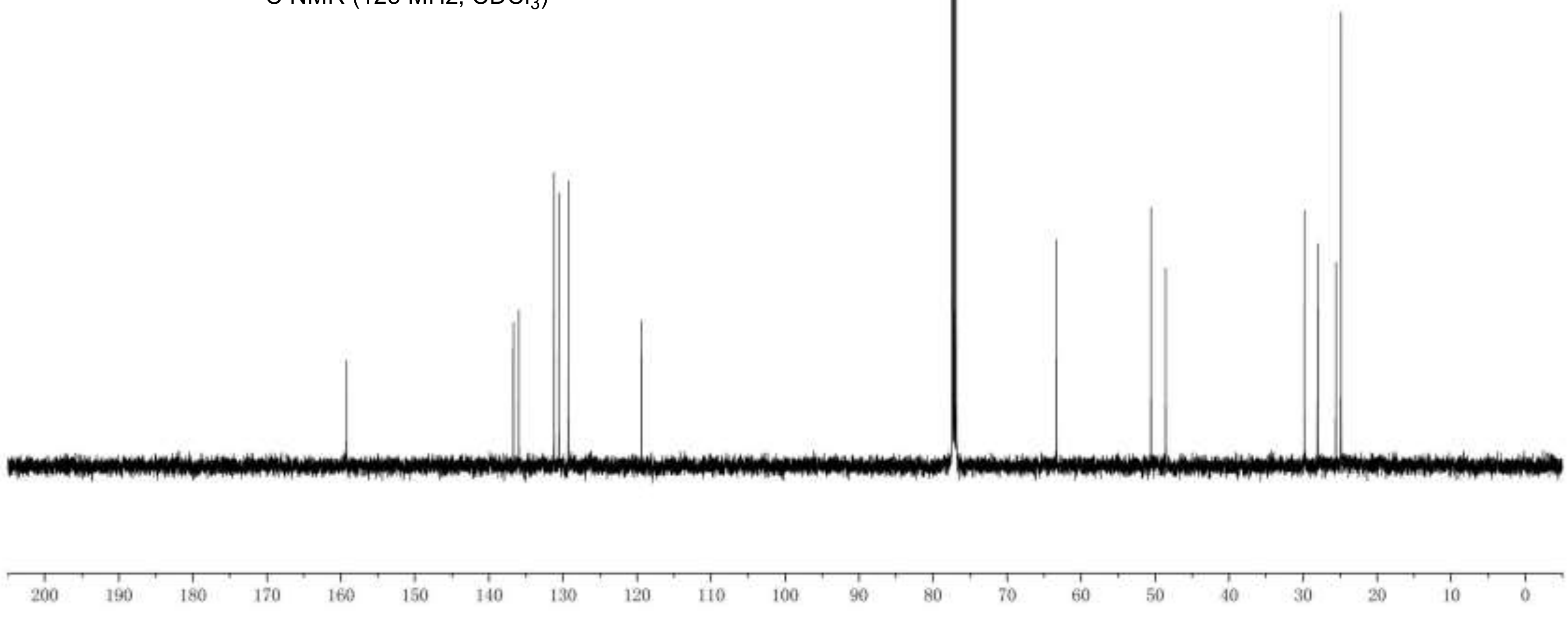

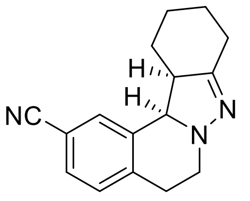

5m

^1H NMR (500 MHz, $CDCl_3$)

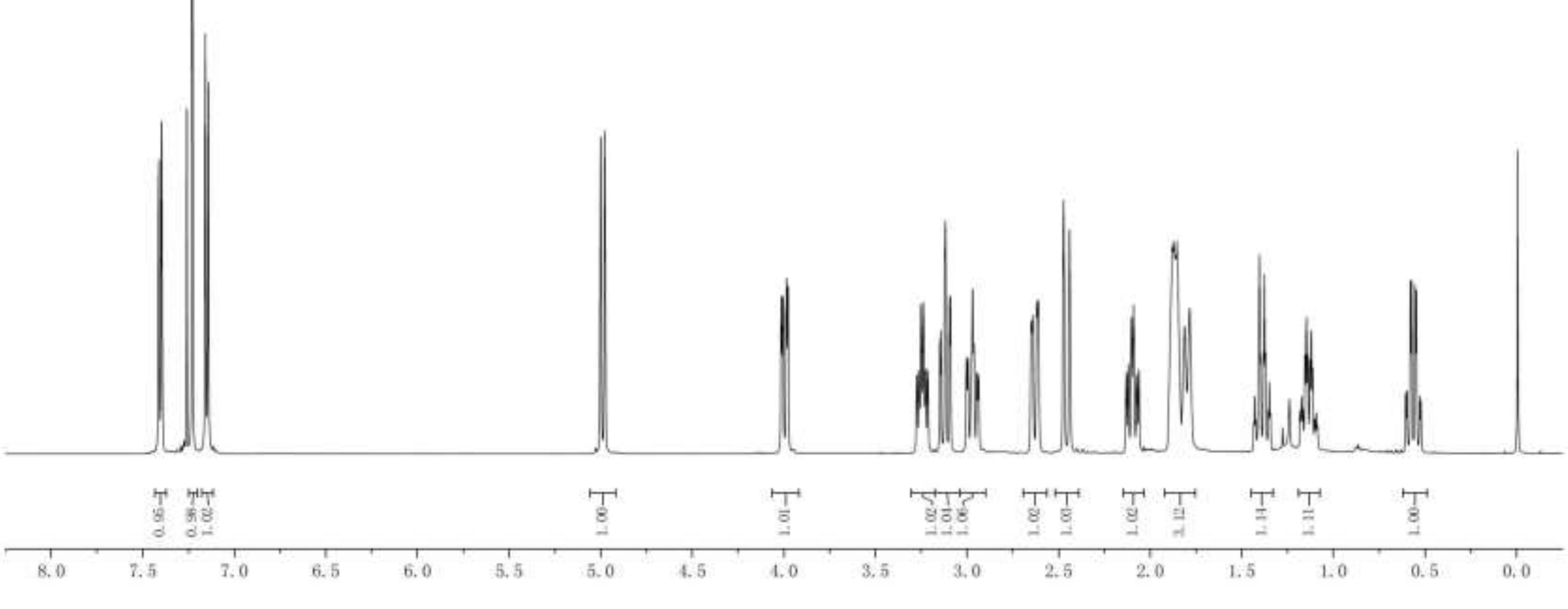

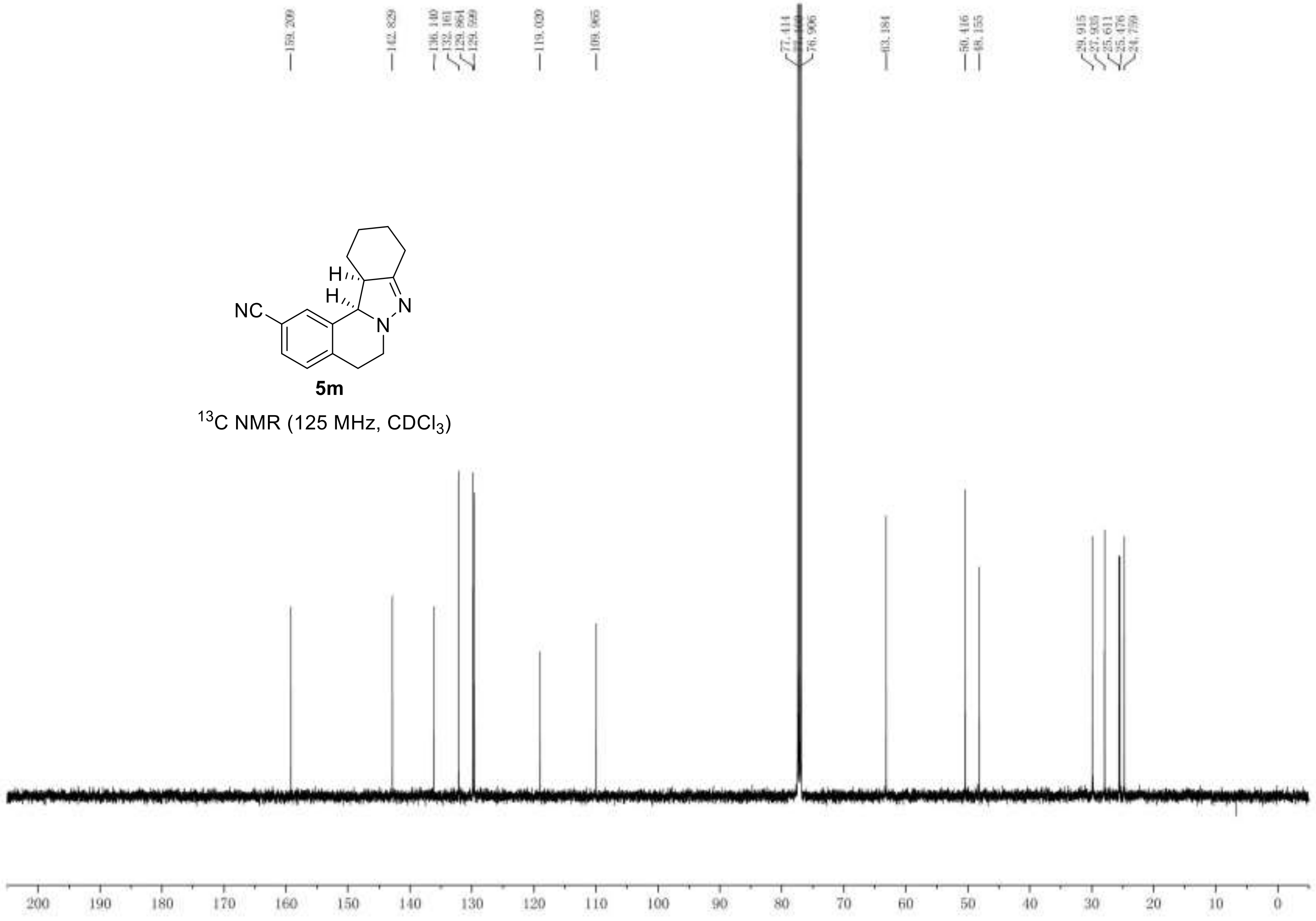

5m

^{13}C NMR (125 MHz, $CDCl_3$)

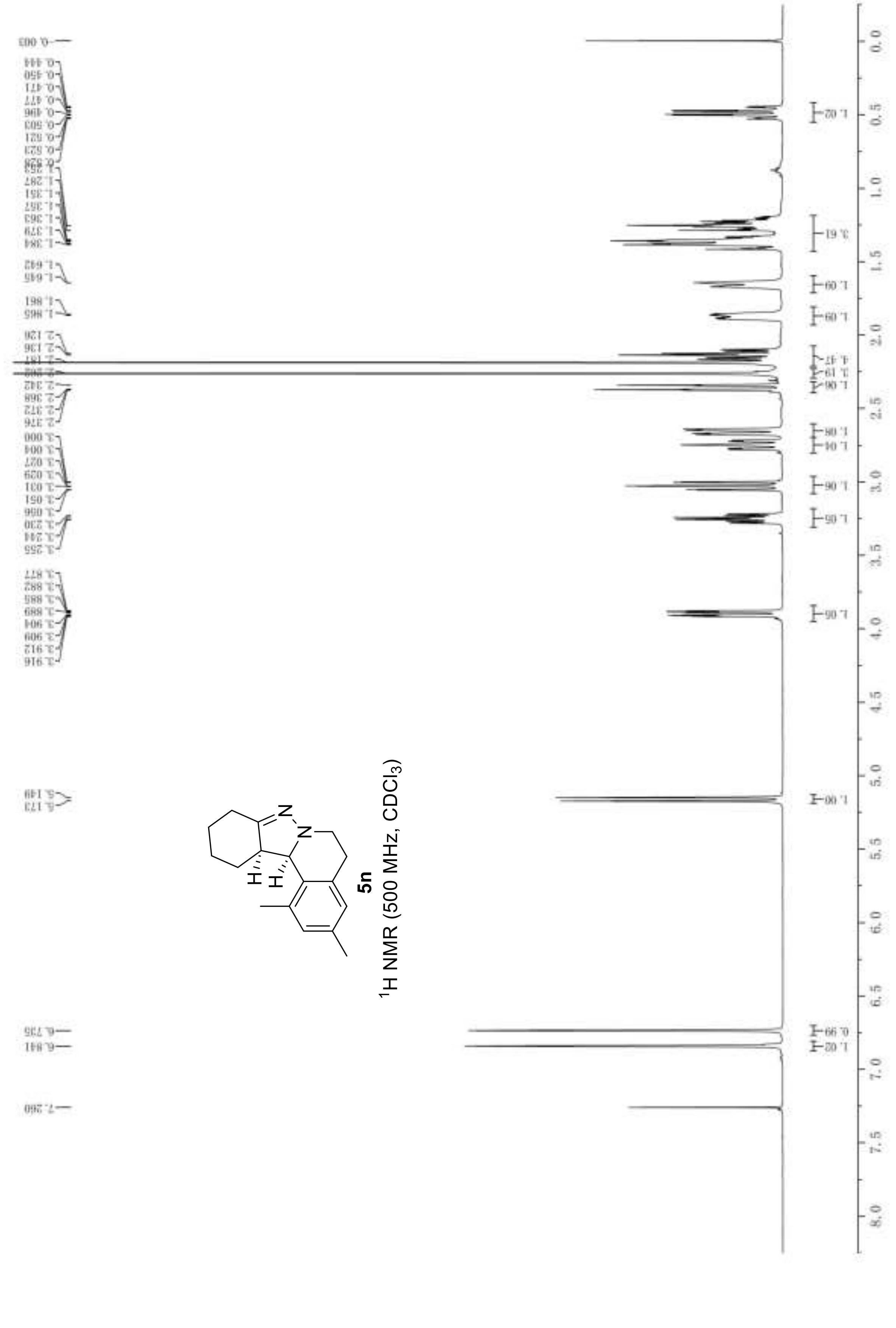
5n
1H NMR (500 MHz, CDCl3)

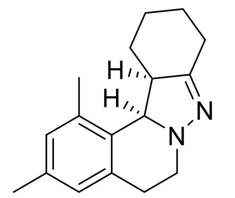

5n

^{13}C NMR (125 MHz, $CDCl_3$)

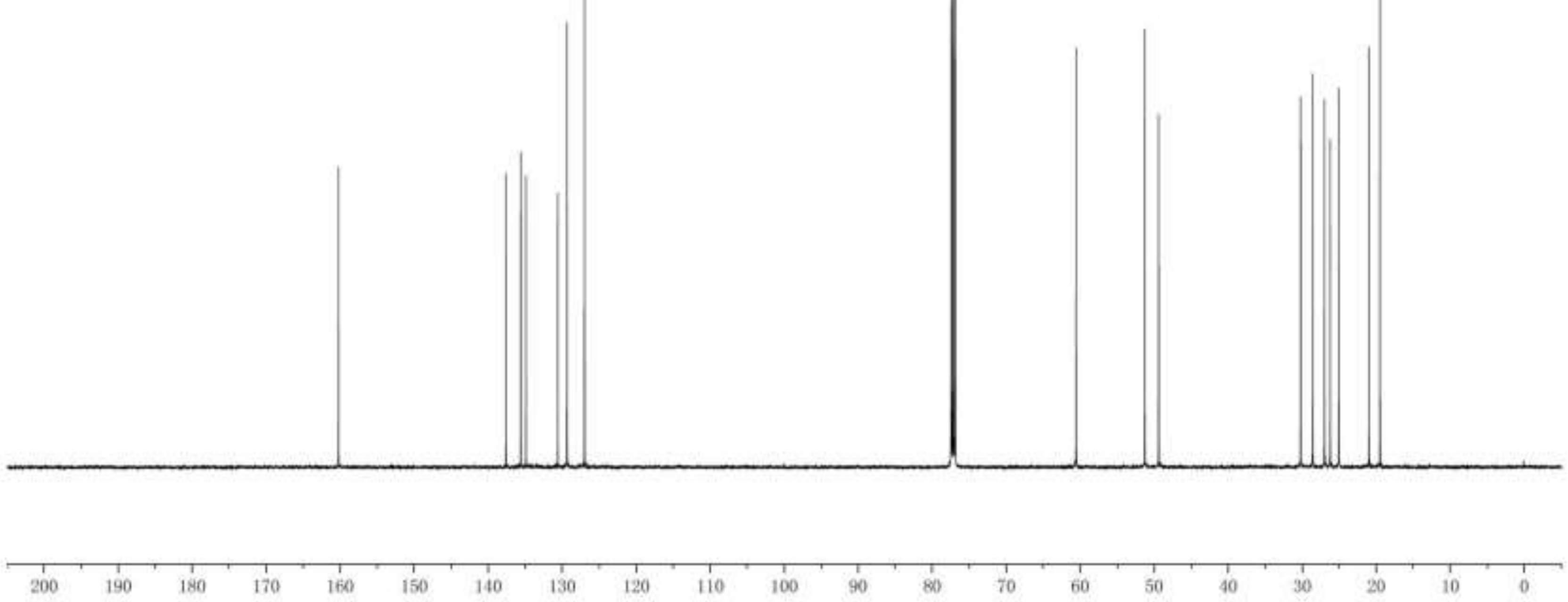

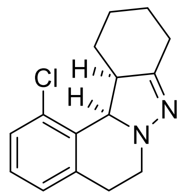

5o

^{1}H NMR (500 MHz, CDCl$_3$)

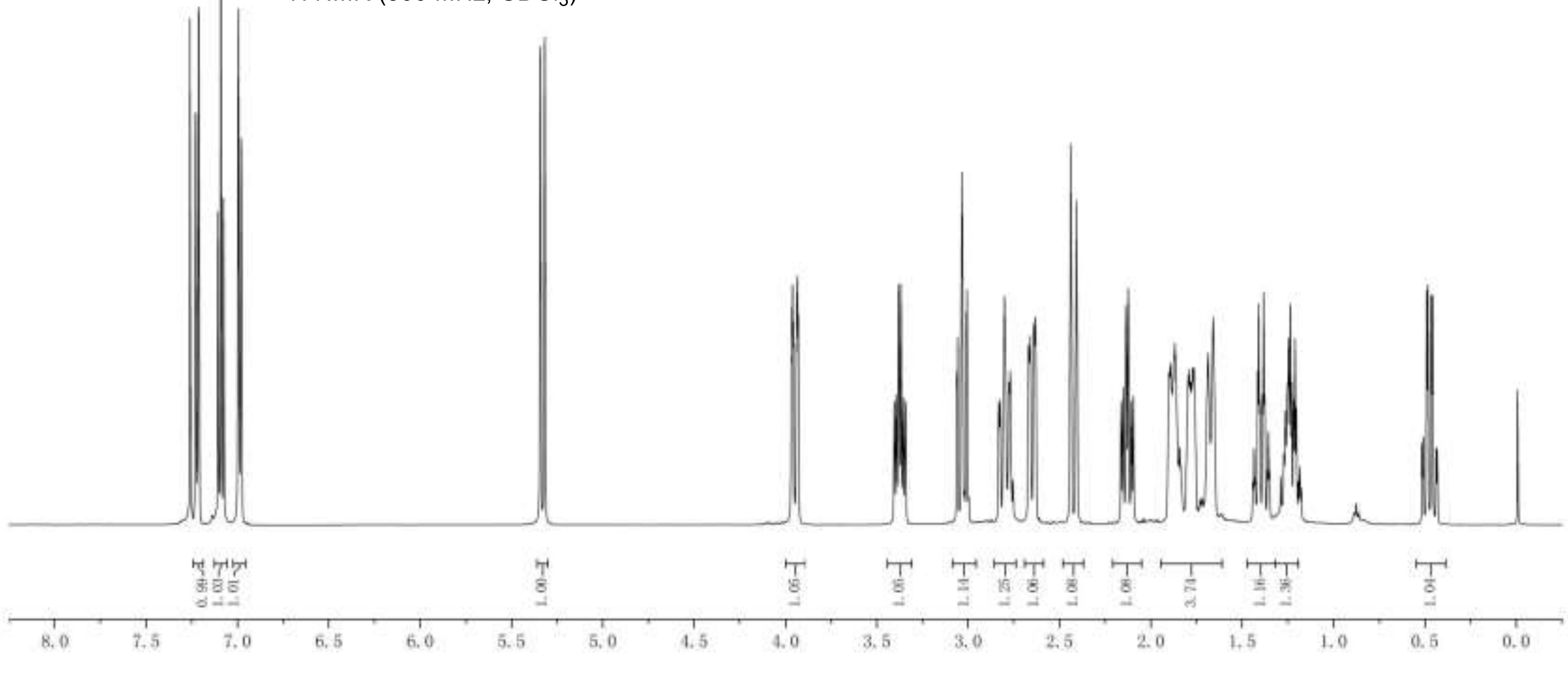

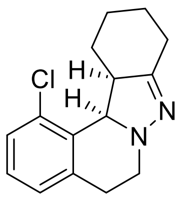

5o

^{13}C NMR (125 MHz, $CDCl_3$)

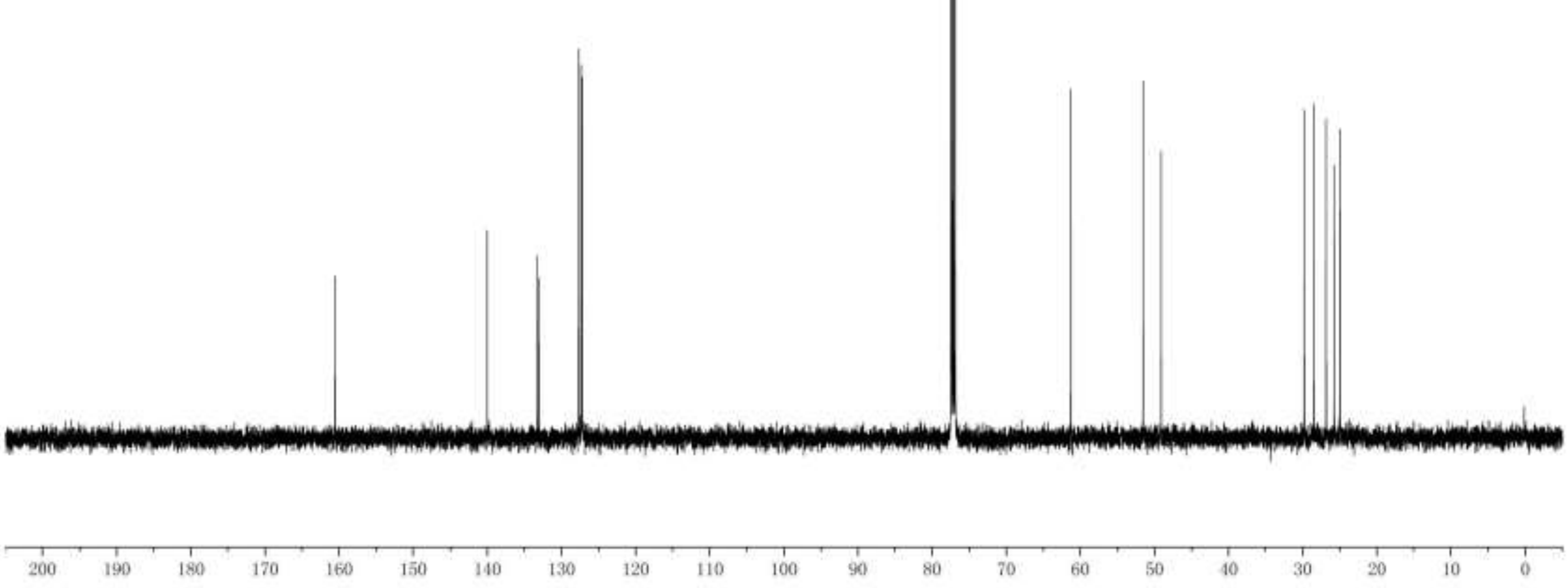

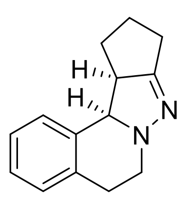

5p

^{1}H NMR (500 MHz, $CDCl_3$)

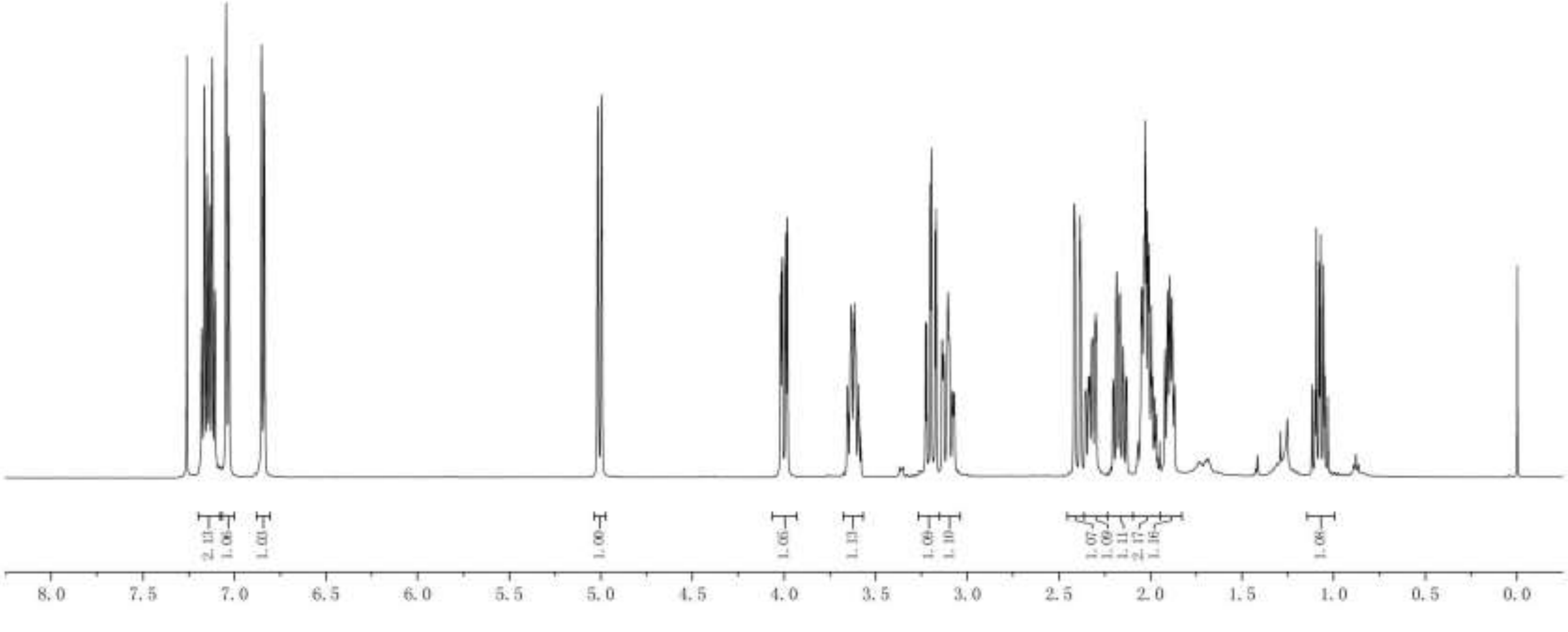

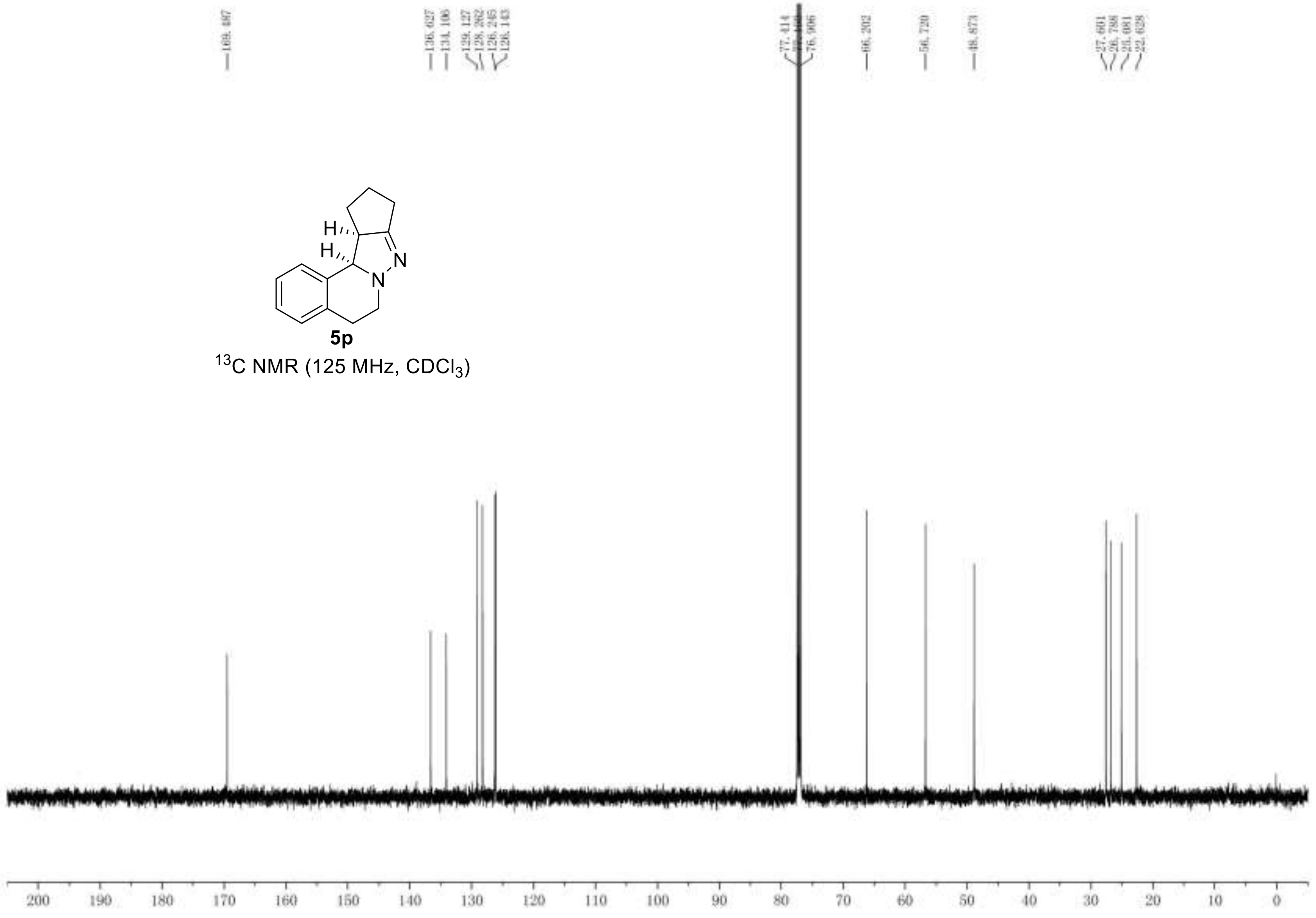

5p

^{13}C NMR (125 MHz, $CDCl_3$)

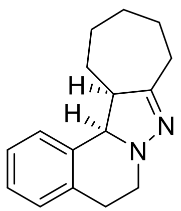

5q

^{1}H NMR (500 MHz, $CDCl_3$)

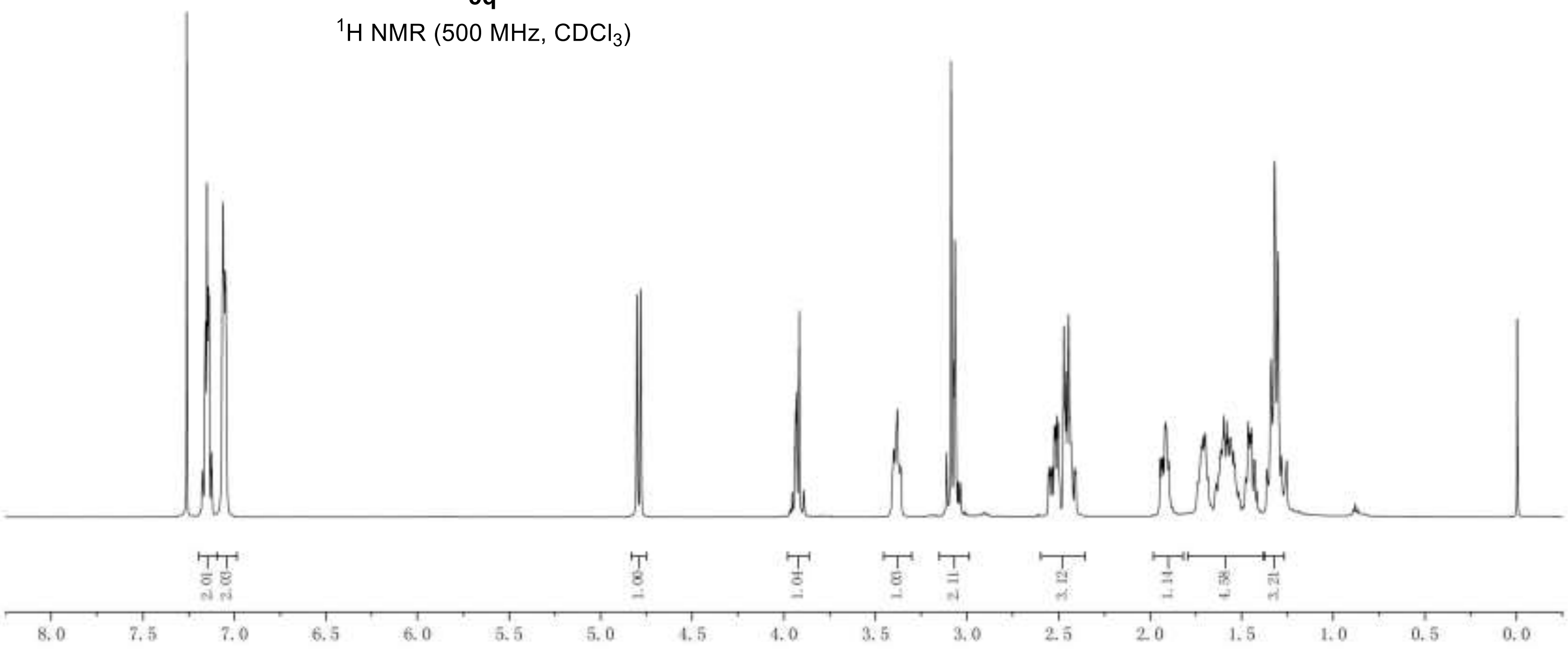

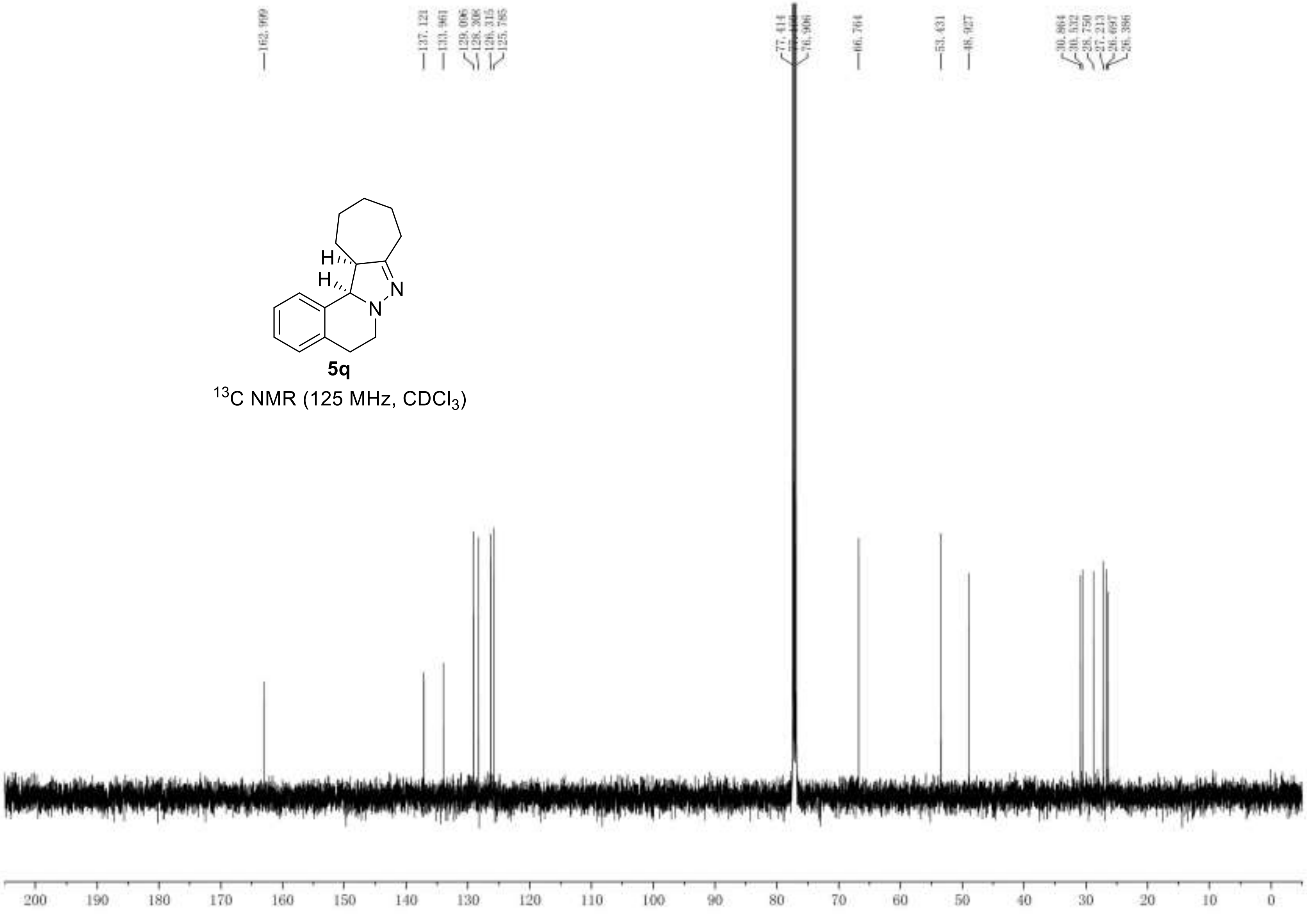

5q

^{13}C NMR (125 MHz, $CDCl_3$)

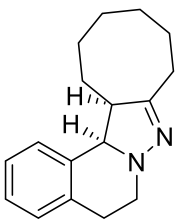

5r

^1H NMR (500 MHz, $CDCl_3$)

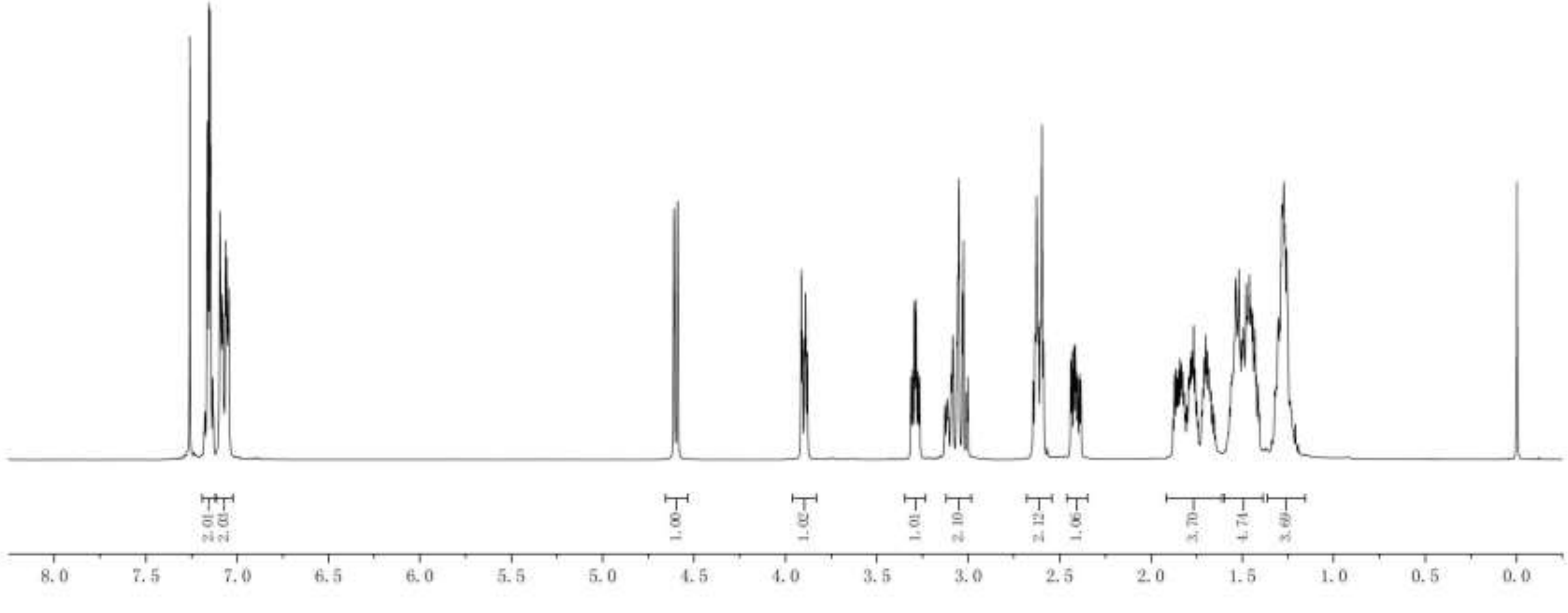

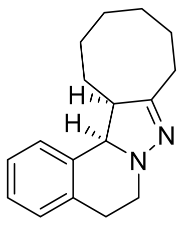

5r

^{13}C NMR (125 MHz, $CDCl_3$)

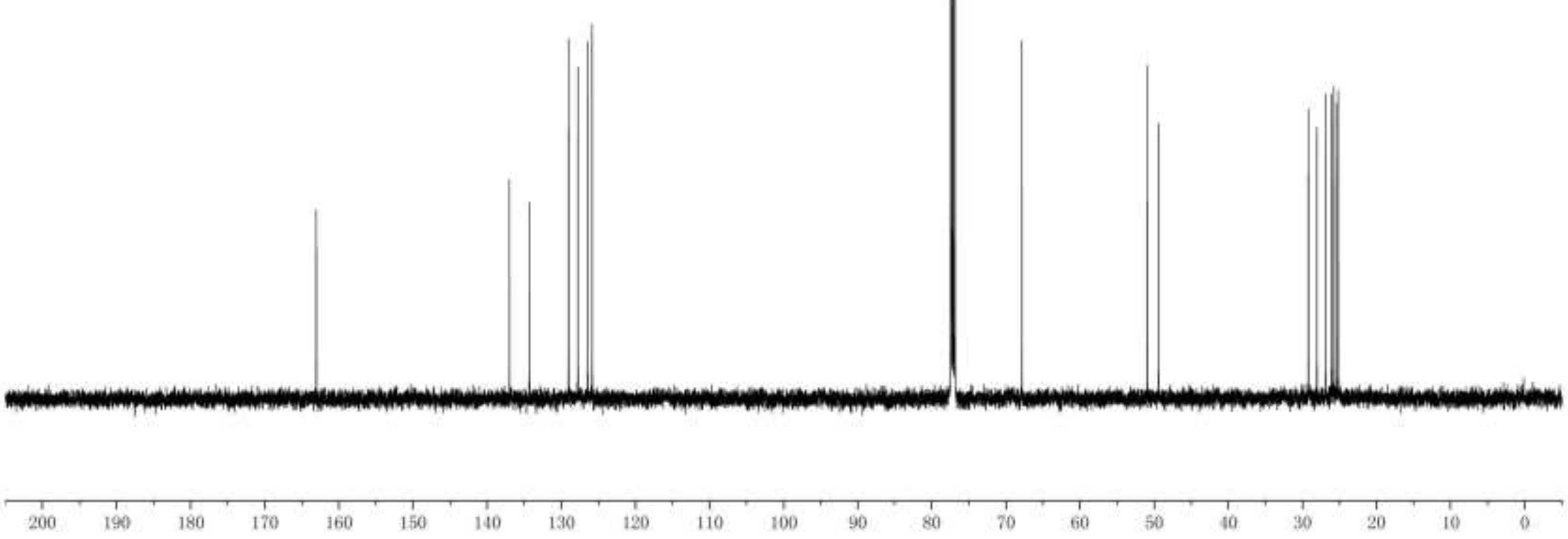

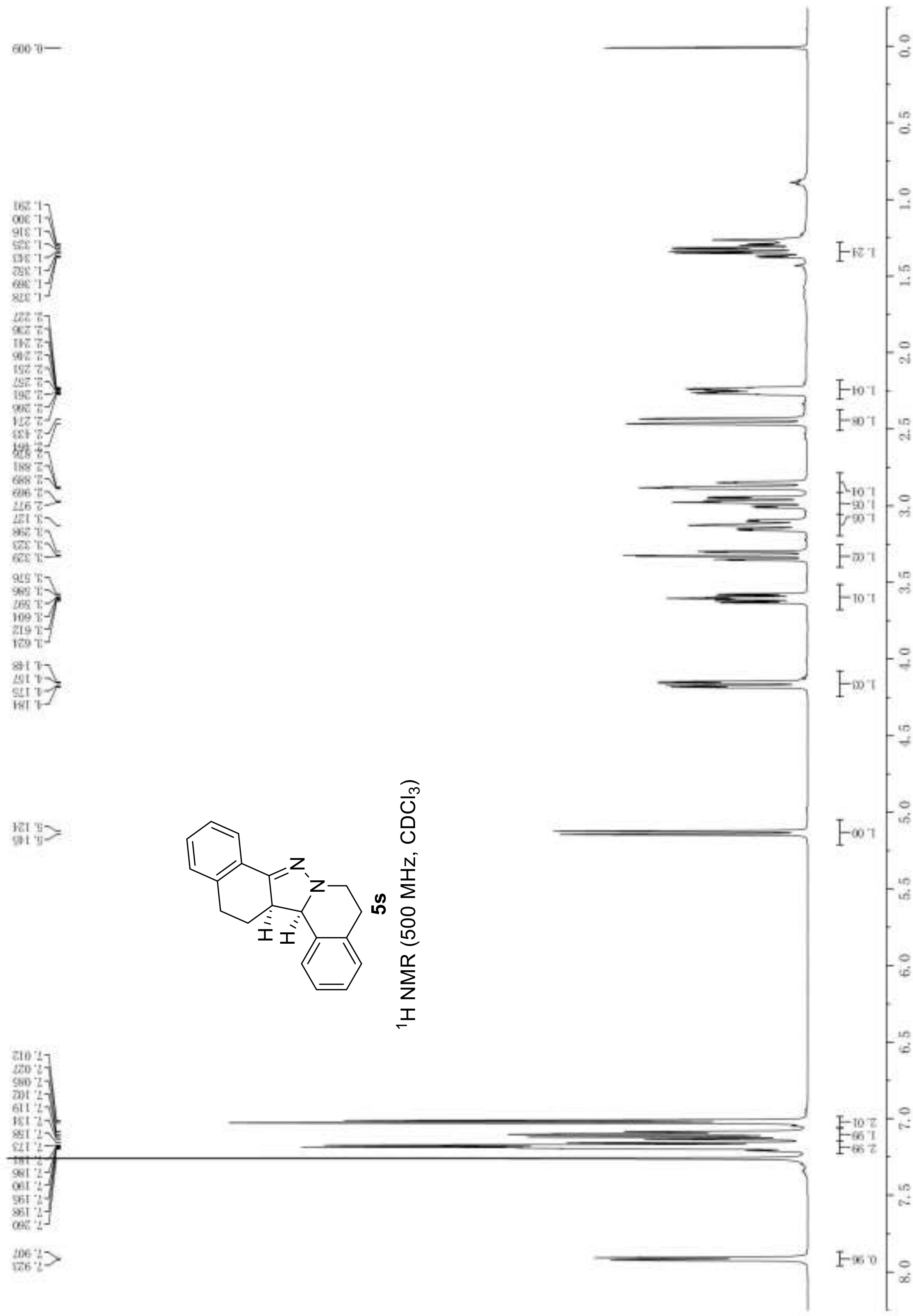

5s

^{1}H NMR (500 MHz, $CDCl_3$)

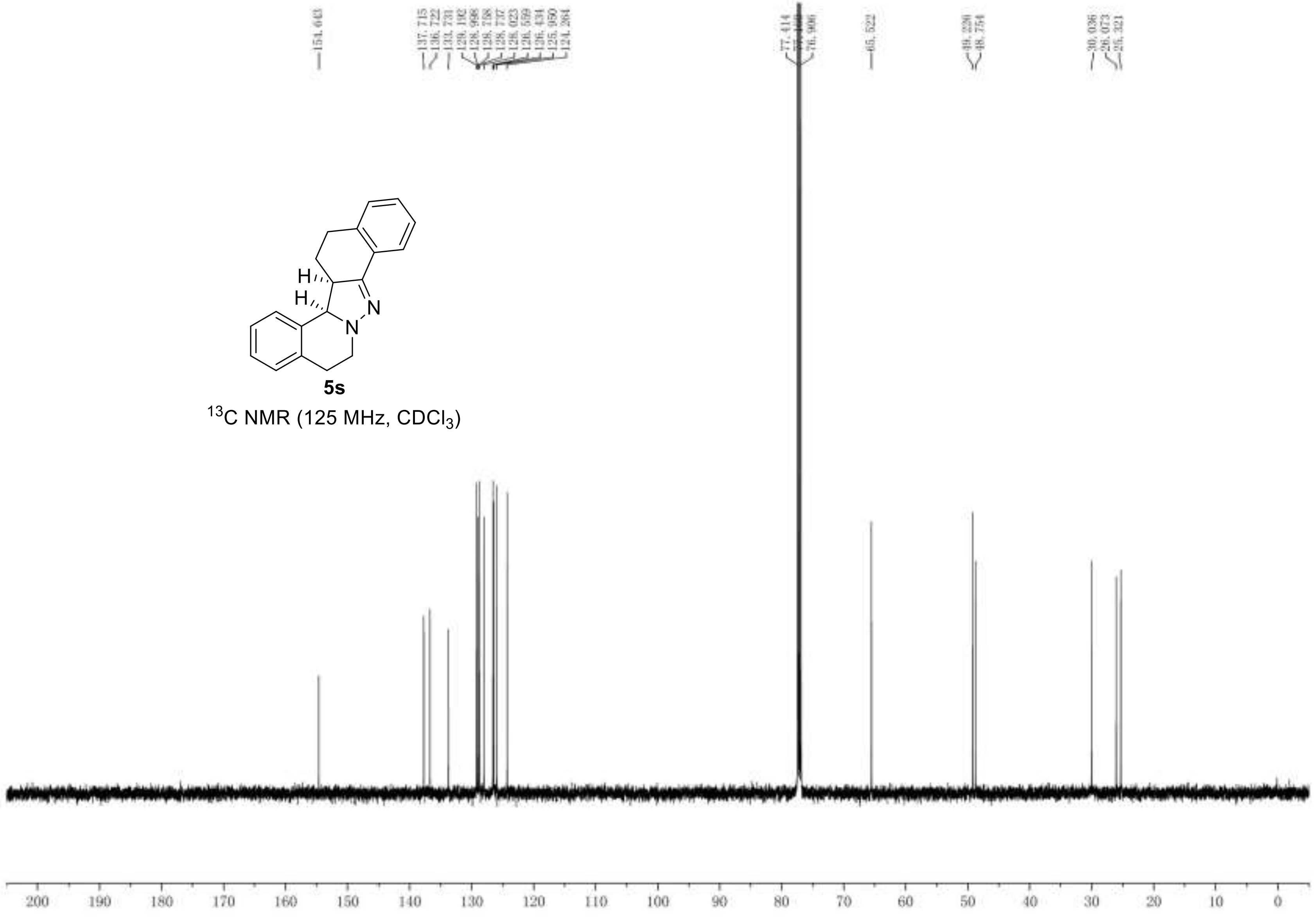

5s

^{13}C NMR (125 MHz, $CDCl_3$)

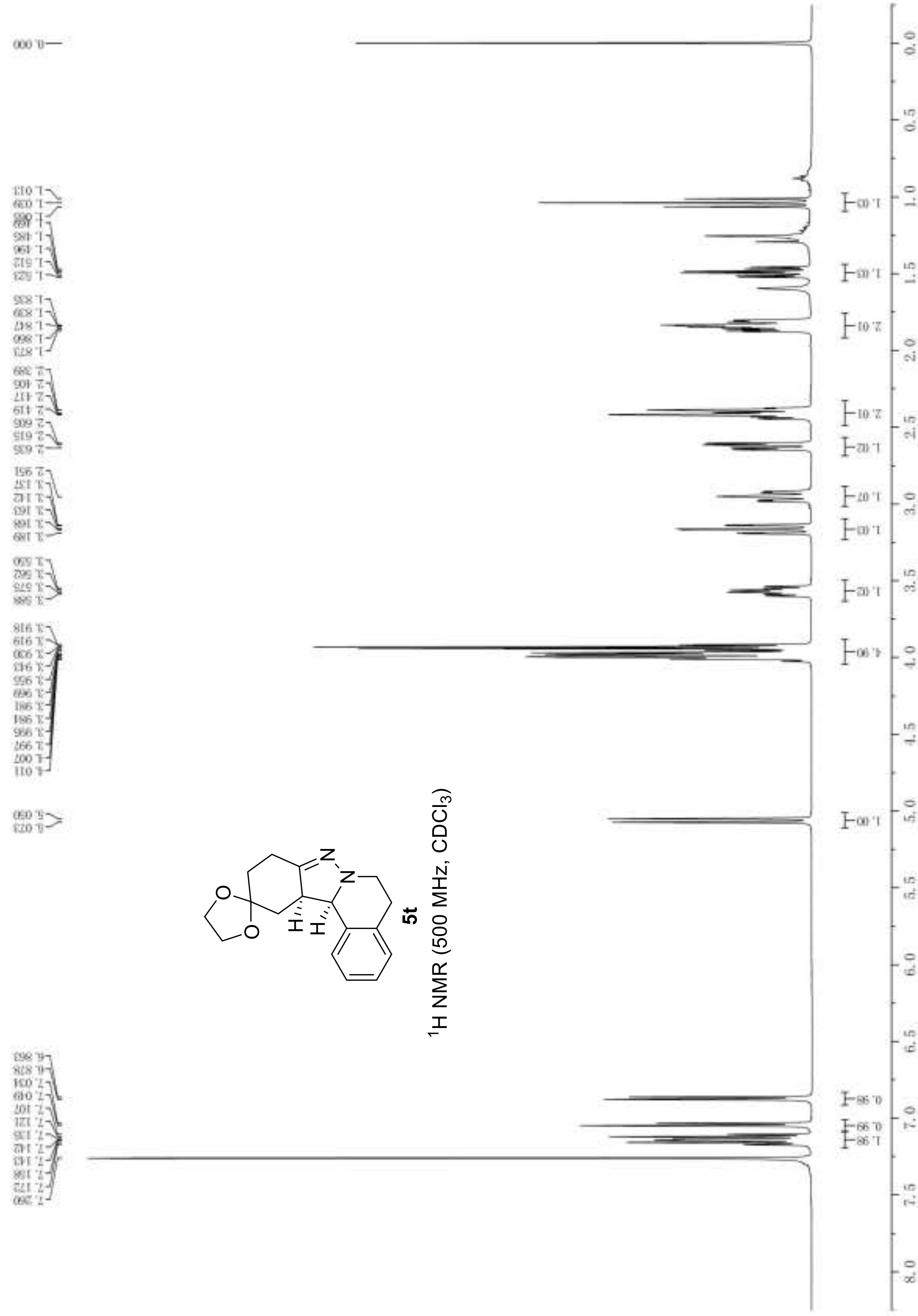

5t

^{1}H NMR (500 MHz, $CDCl_3$)

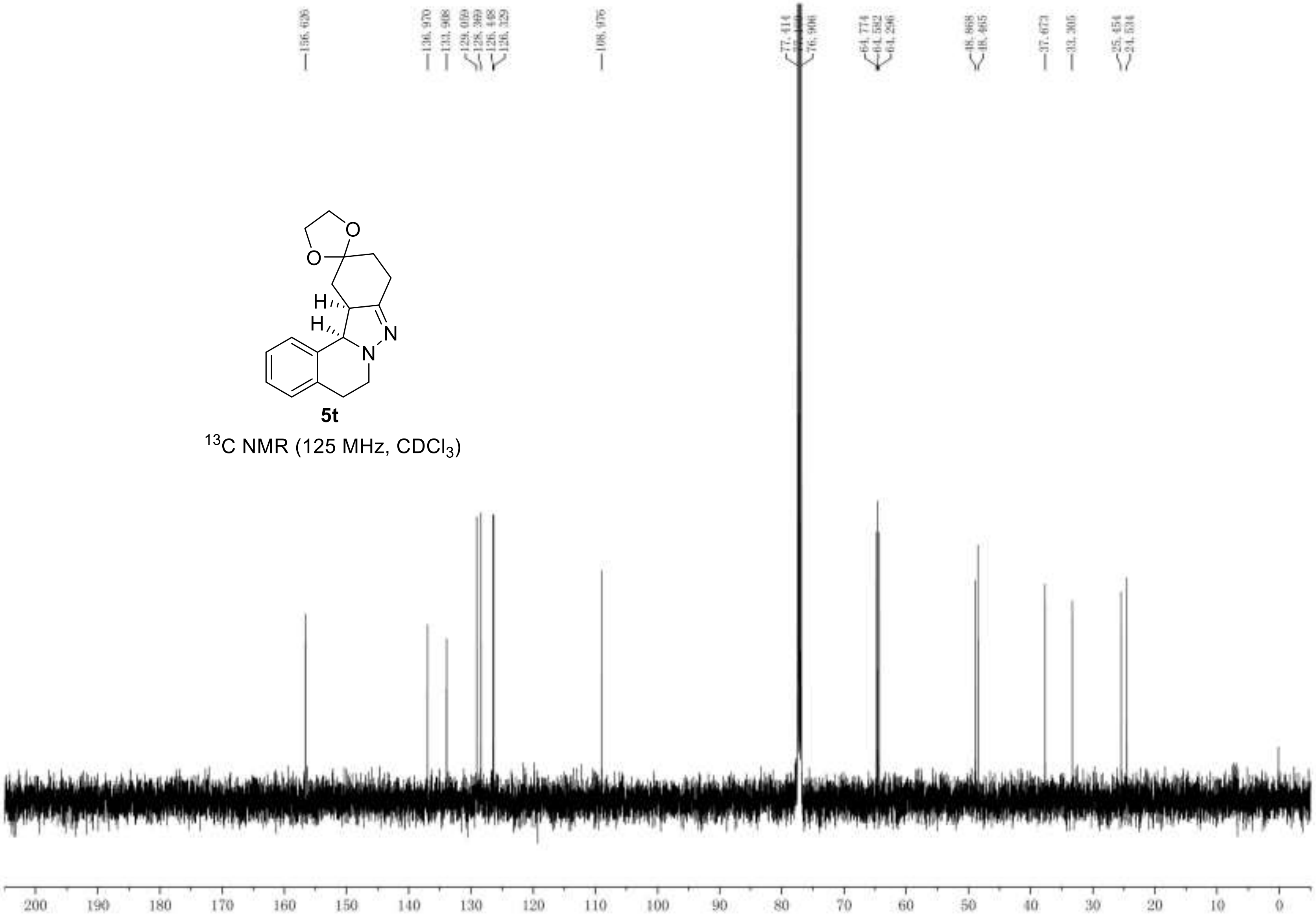

5t

^{13}C NMR (125 MHz, $CDCl_3$)

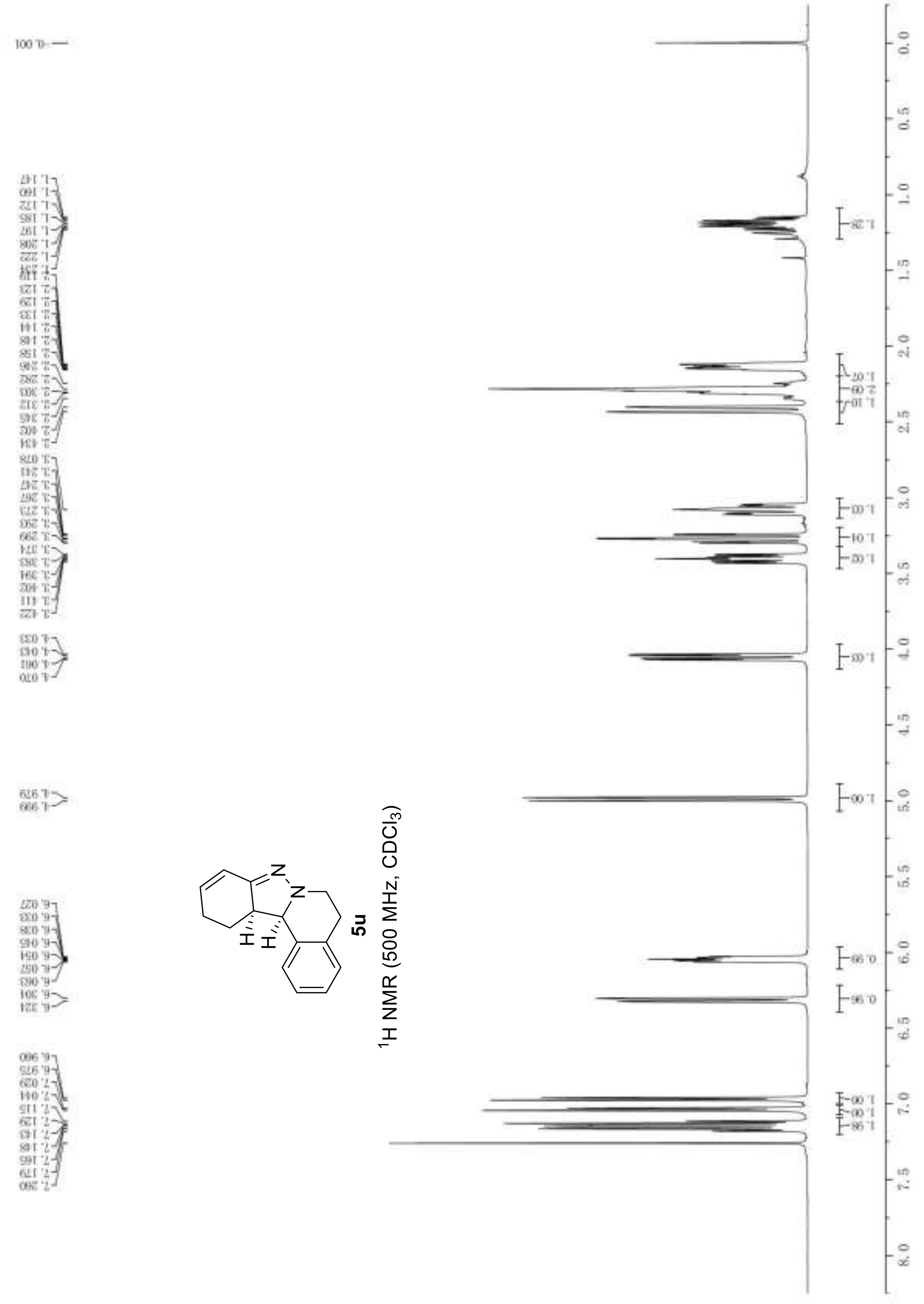
5u
1H NMR (500 MHz, CDCl3)

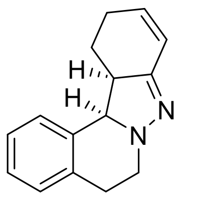

5u

^{13}C NMR (125 MHz, $CDCl_3$)

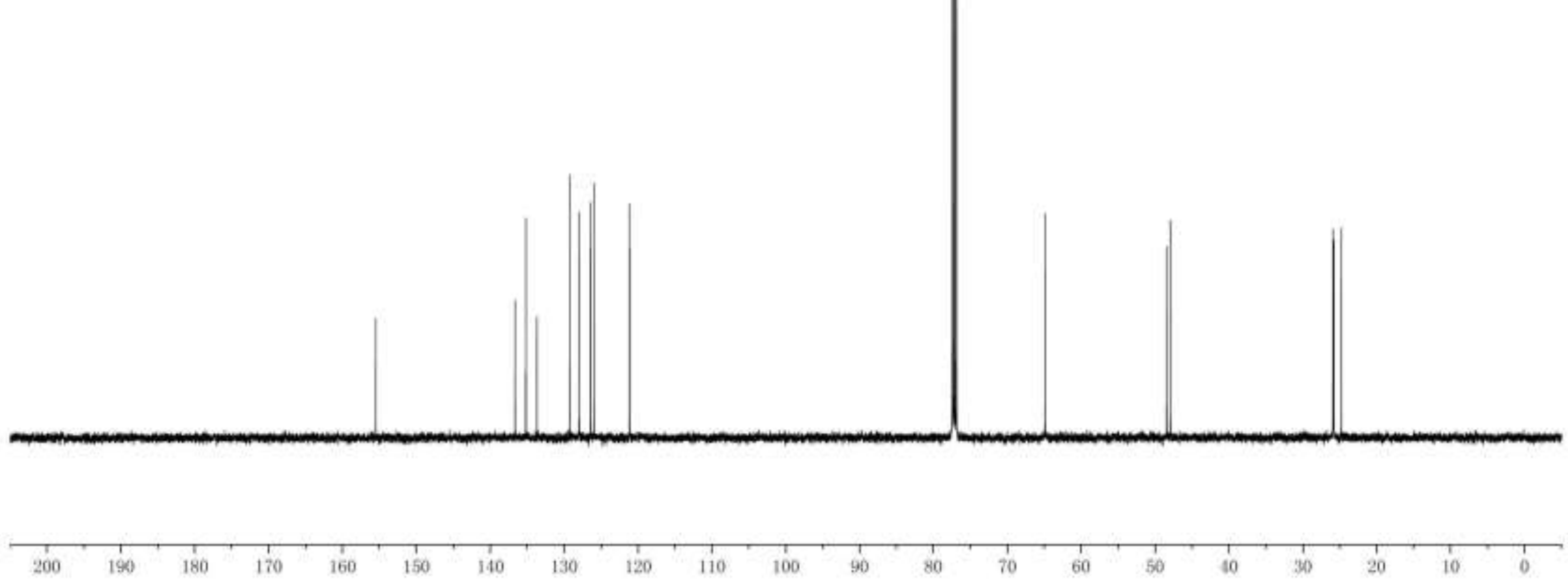

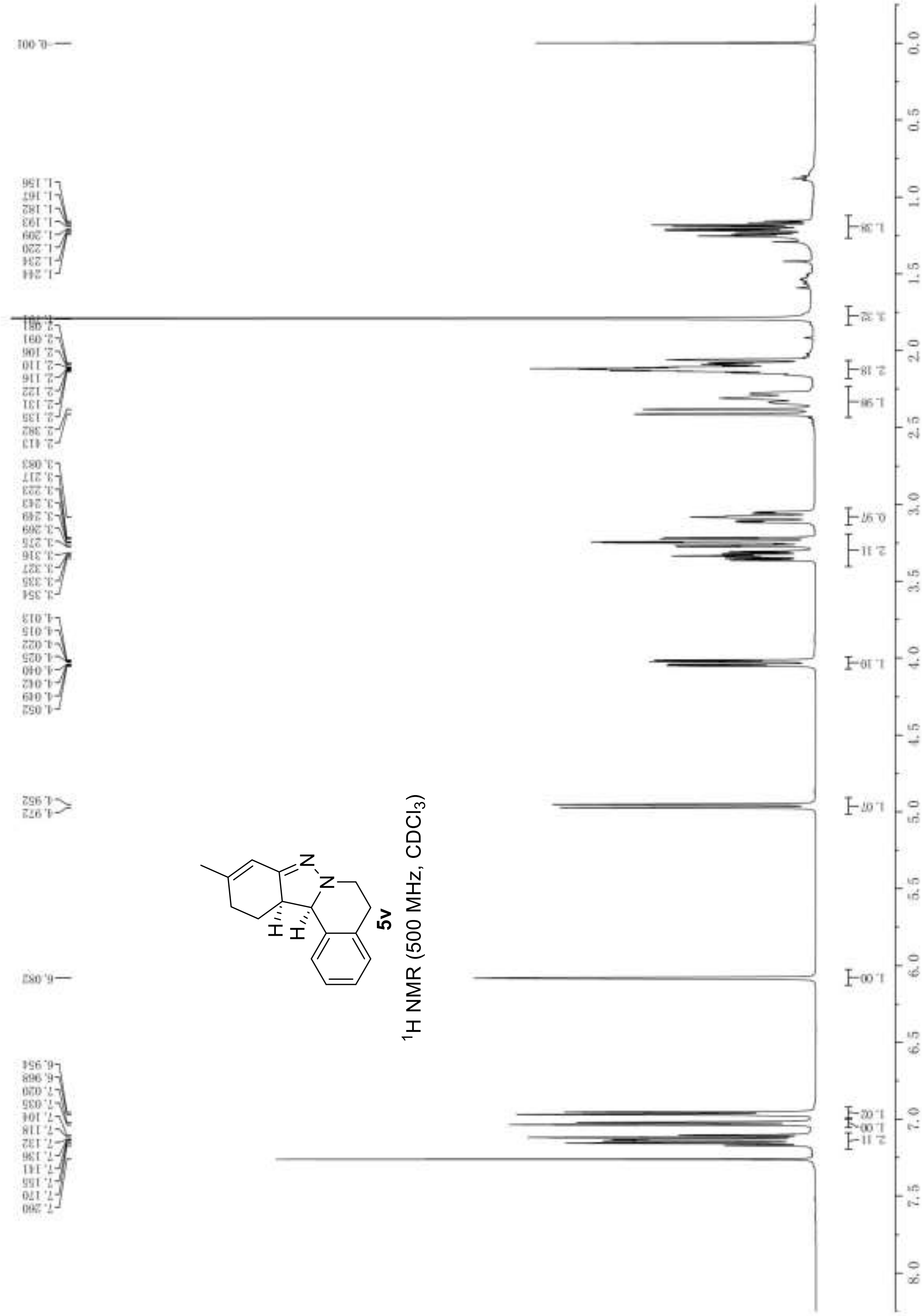

5v

^1H NMR (500 MHz, $CDCl_3$)

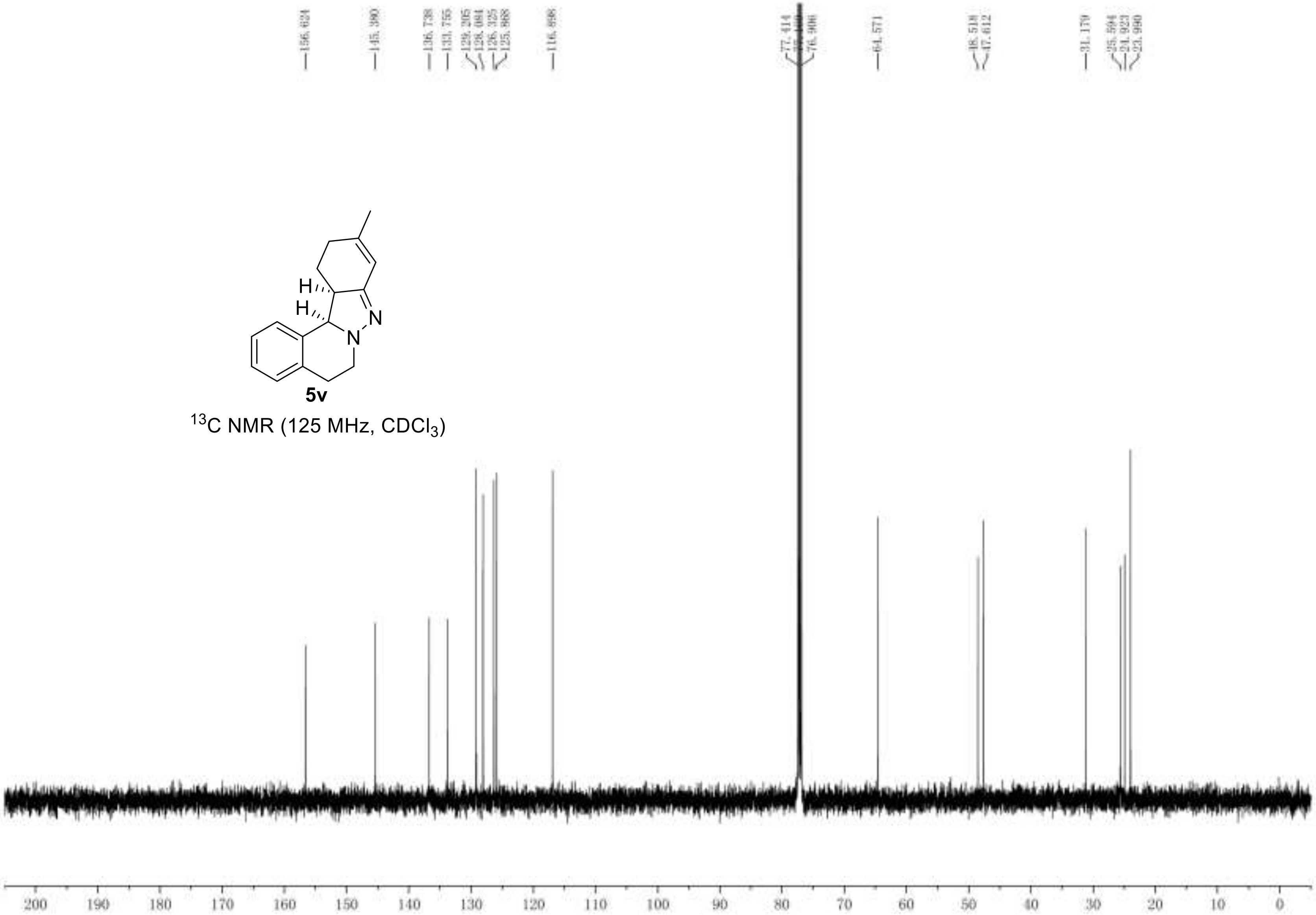

5v

^{13}C NMR (125 MHz, $CDCl_3$)

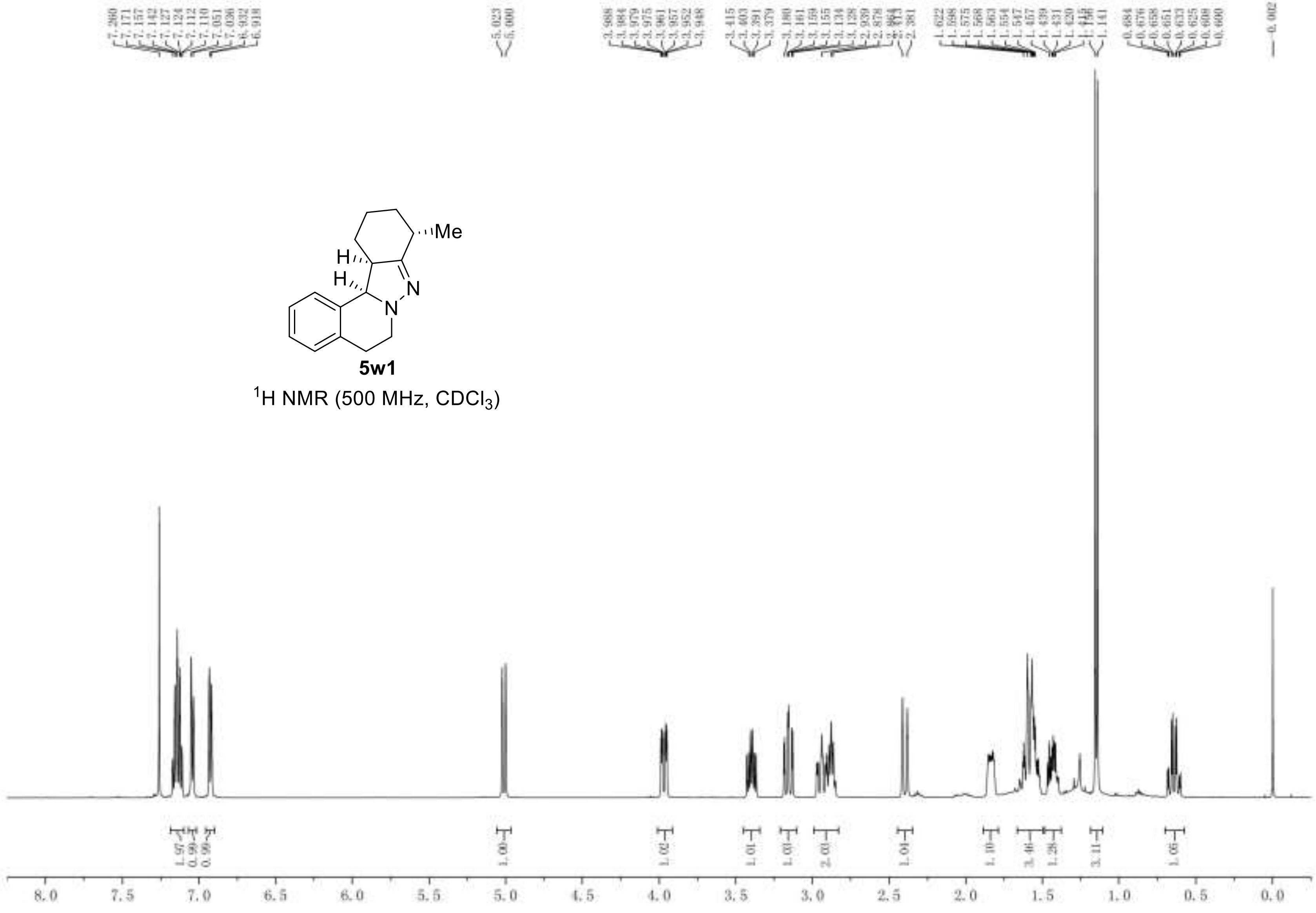

5w1

^{1}H NMR (500 MHz, $CDCl_3$)

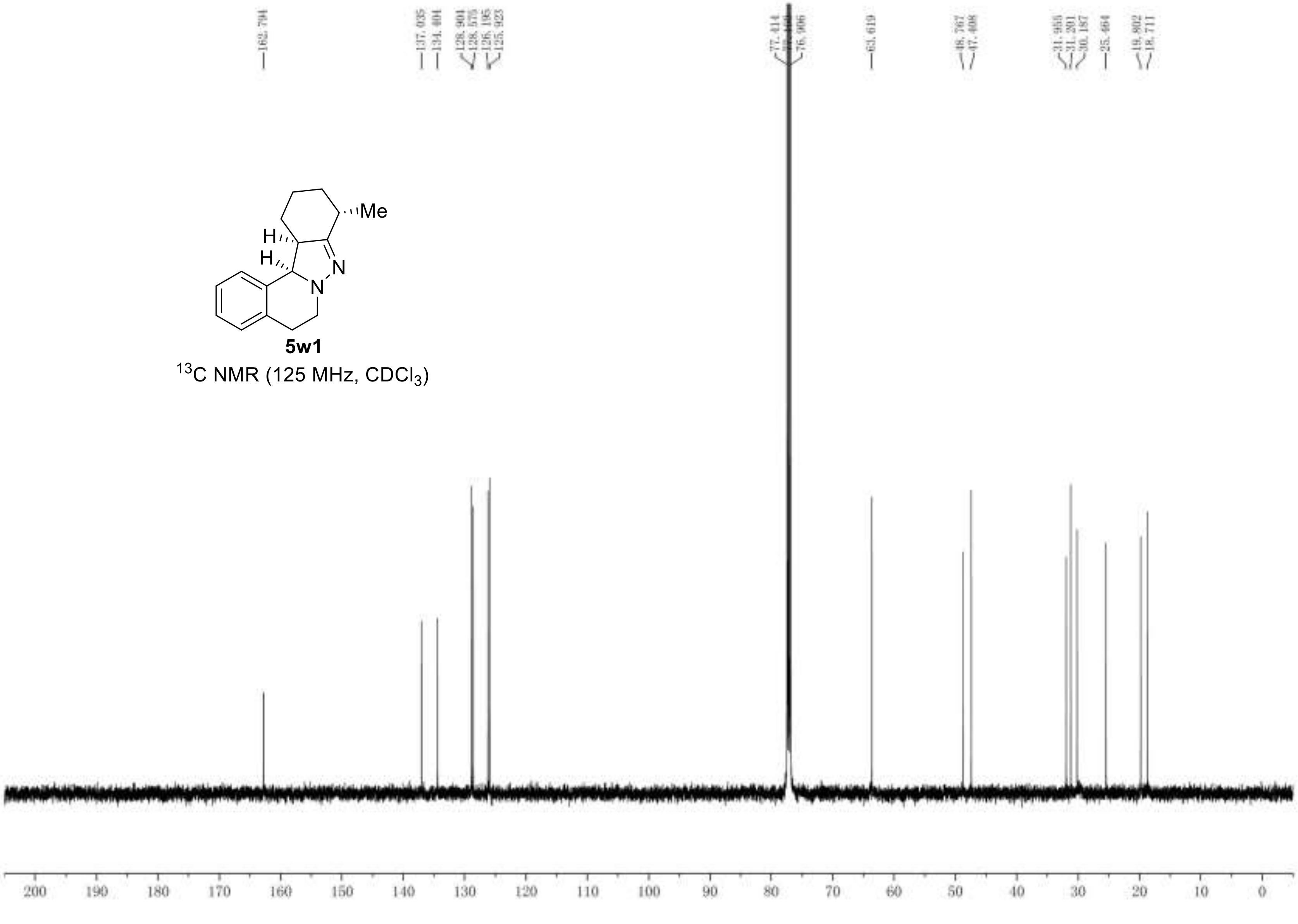

5w1

^{13}C NMR (125 MHz, $CDCl_3$)

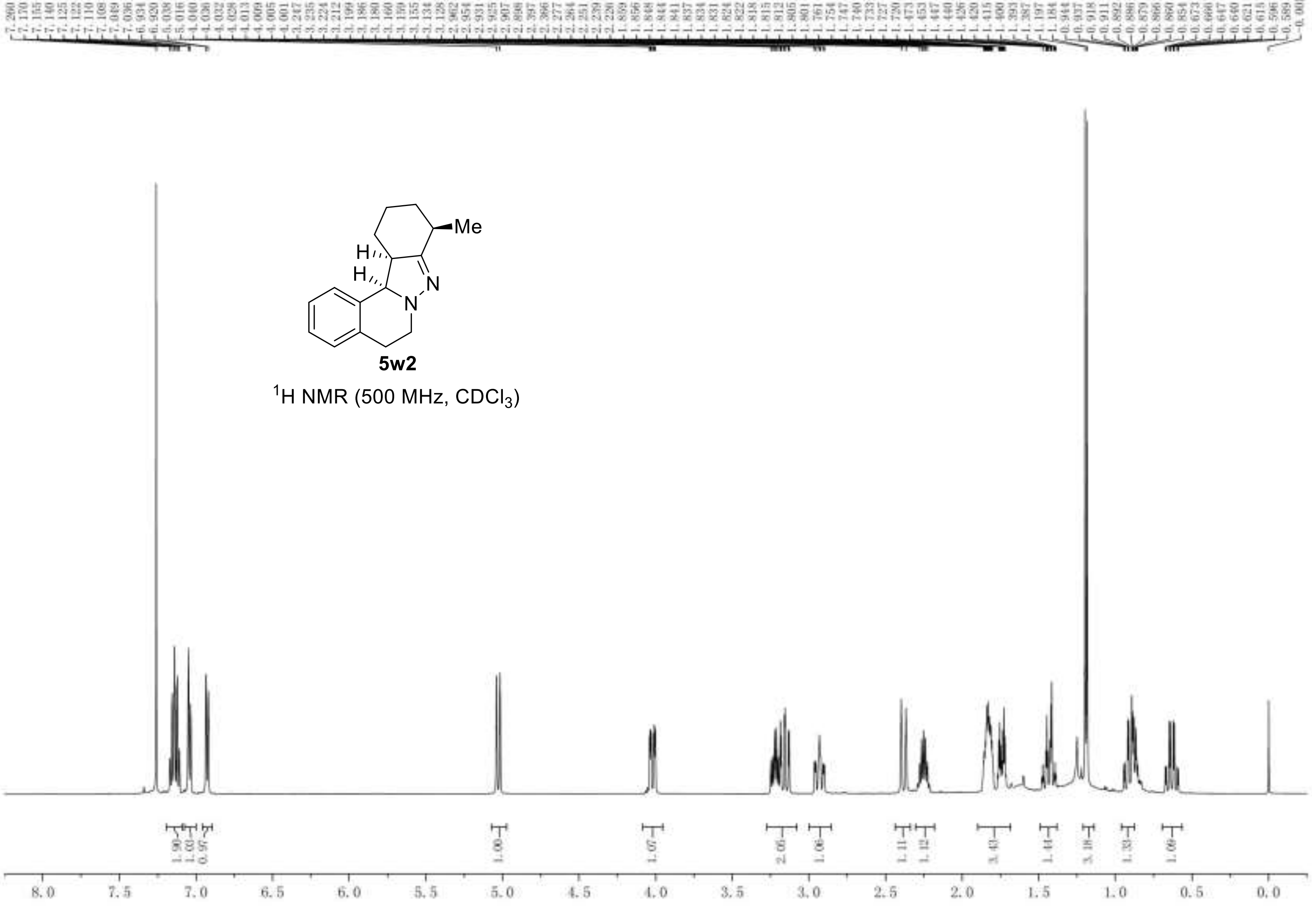

5w2

^{1}H NMR (500 MHz, $CDCl_3$)

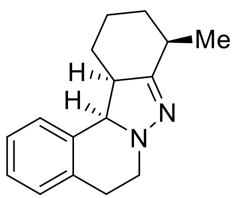

5w2

^{13}C NMR (125 MHz, $CDCl_3$)

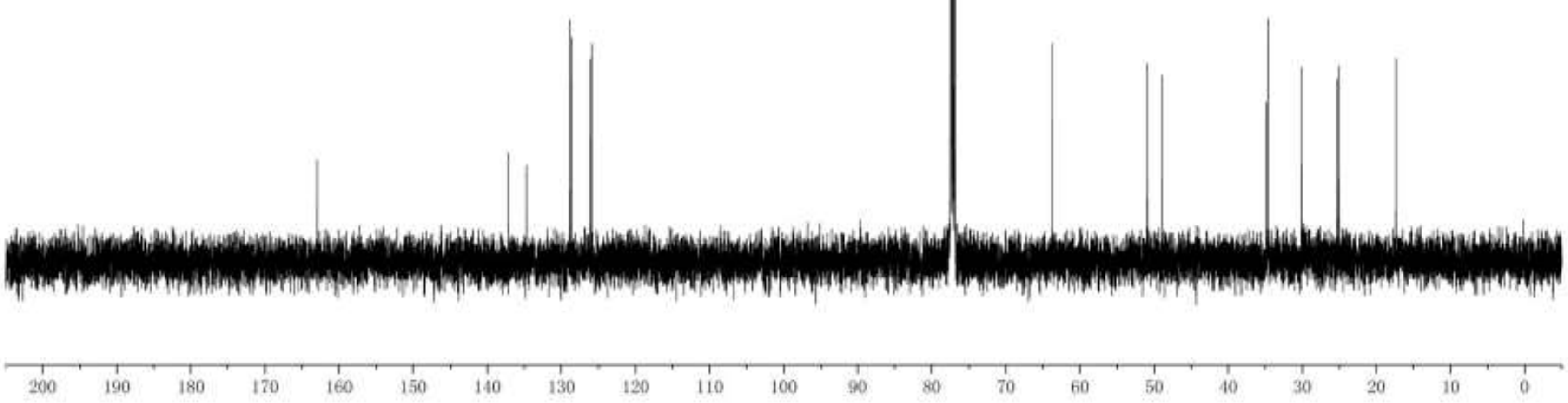

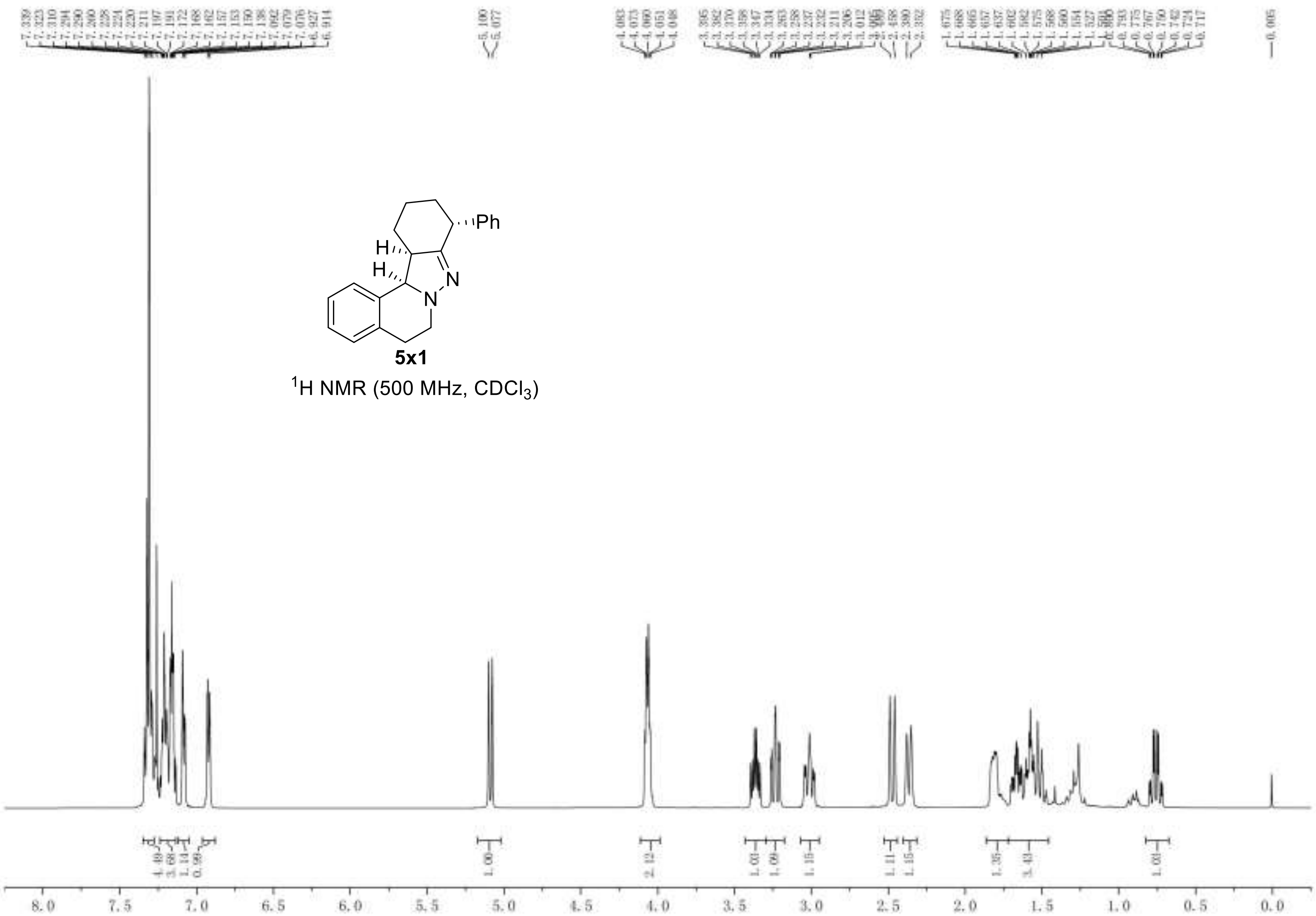

5x1

^1H NMR (500 MHz, $CDCl_3$)

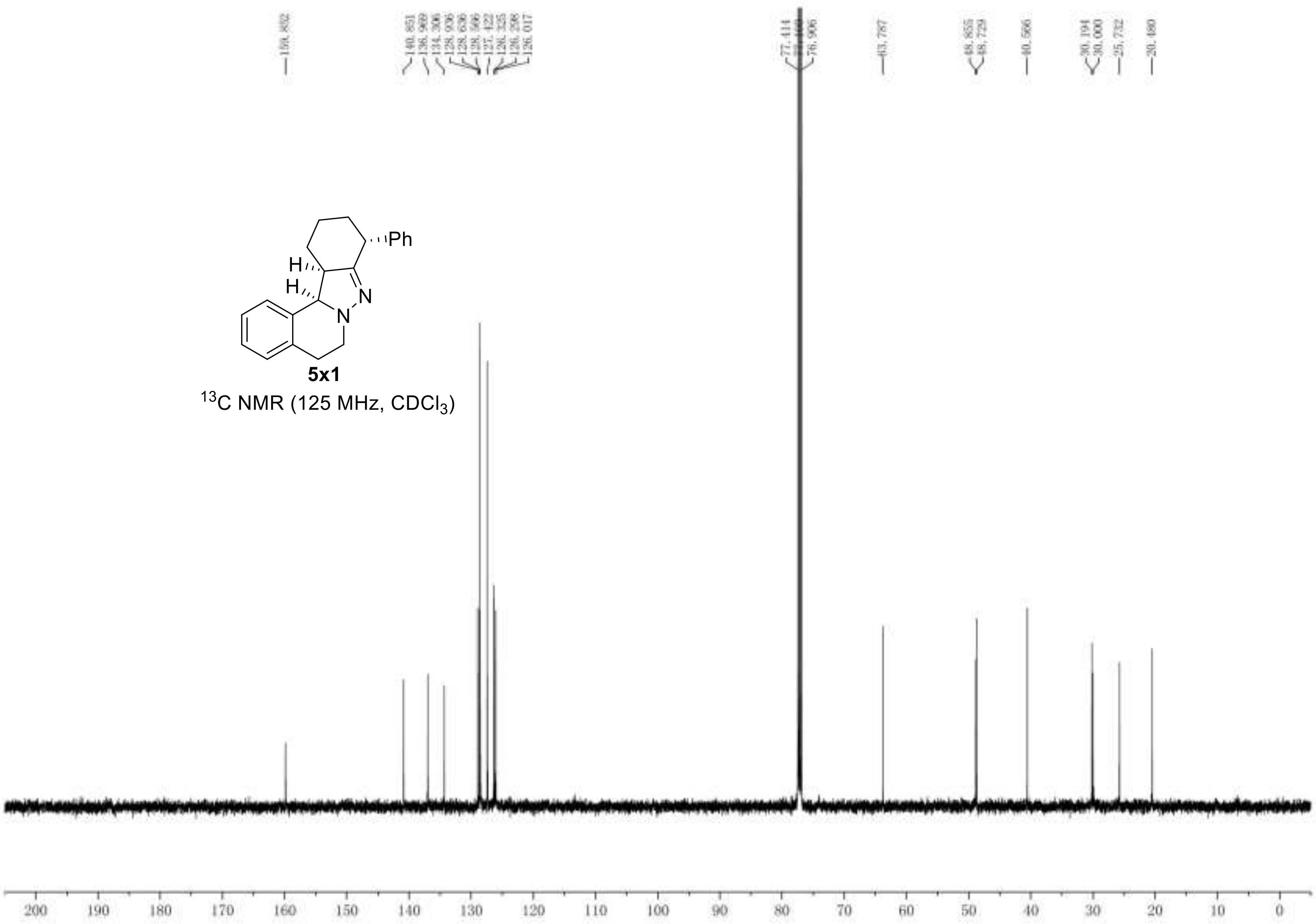
5x1
^{13}C NMR (125 MHz, CDCl$_3$)
Ph
H
H
N
N
159.802
140.851
136.969
134.306
128.926
128.636
128.566
127.422
126.325
126.298
126.017
77.414
76.906
63.787
48.855
48.729
40.566
30.194
30.000
25.732
20.480
200 190 180 170 160 150 140 130 120 110 100 90 80 70 60 50 40 30 20 10 0

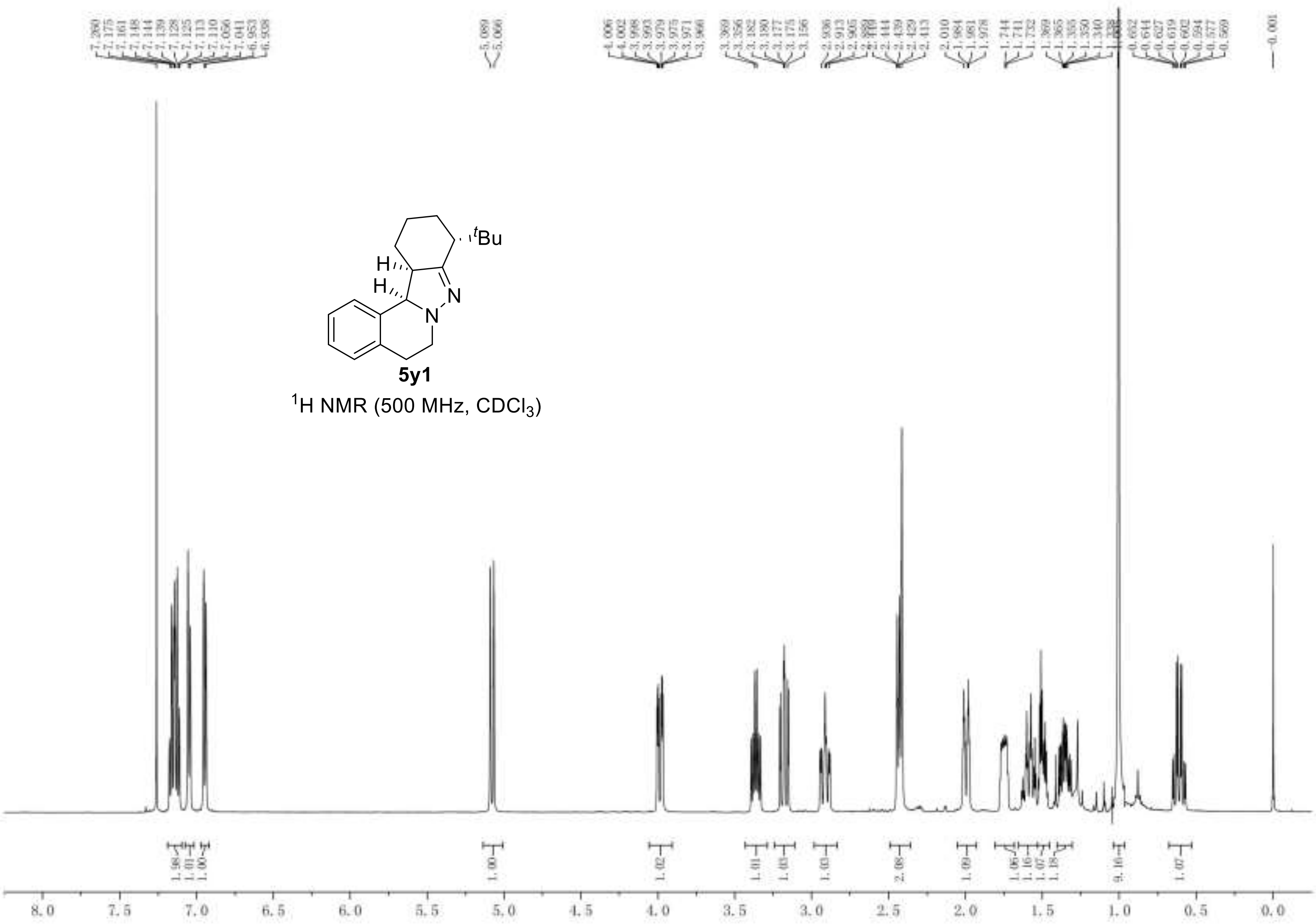

5y1

^{1}H NMR (500 MHz, $CDCl_3$)

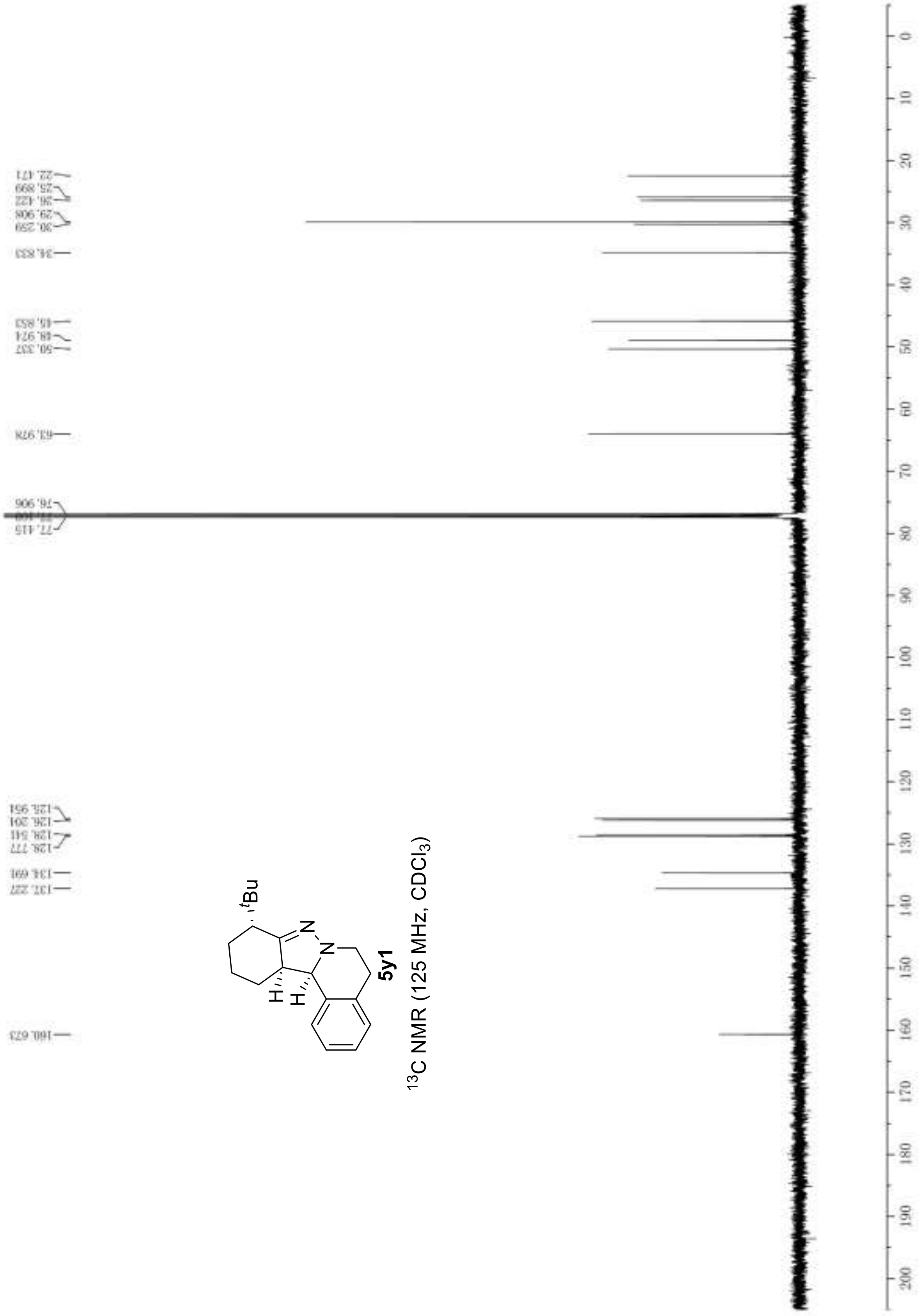

5y1

^{13}C NMR (125 MHz, $CDCl_3$)

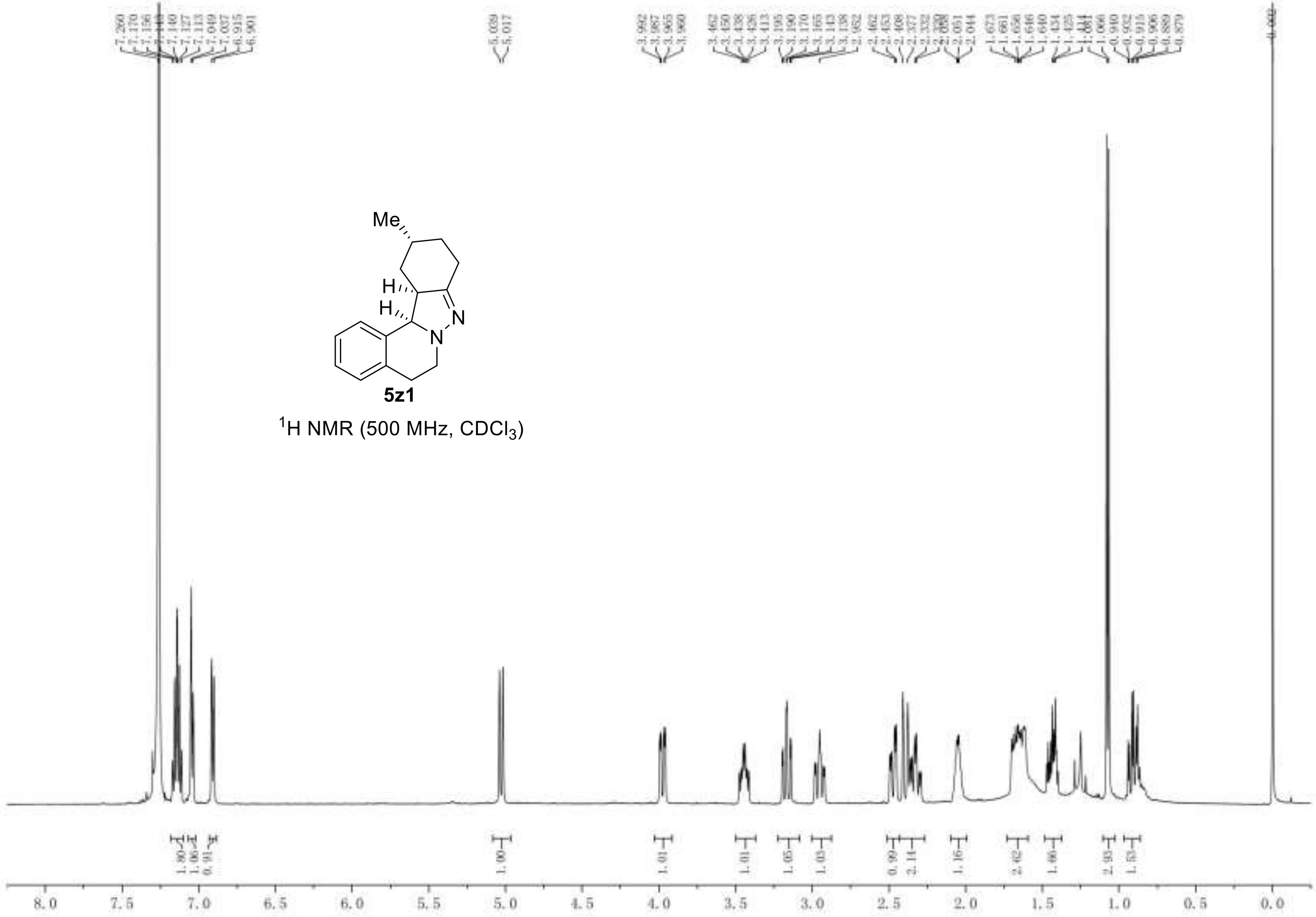

5z1

^{1}H NMR (500 MHz, $CDCl_3$)

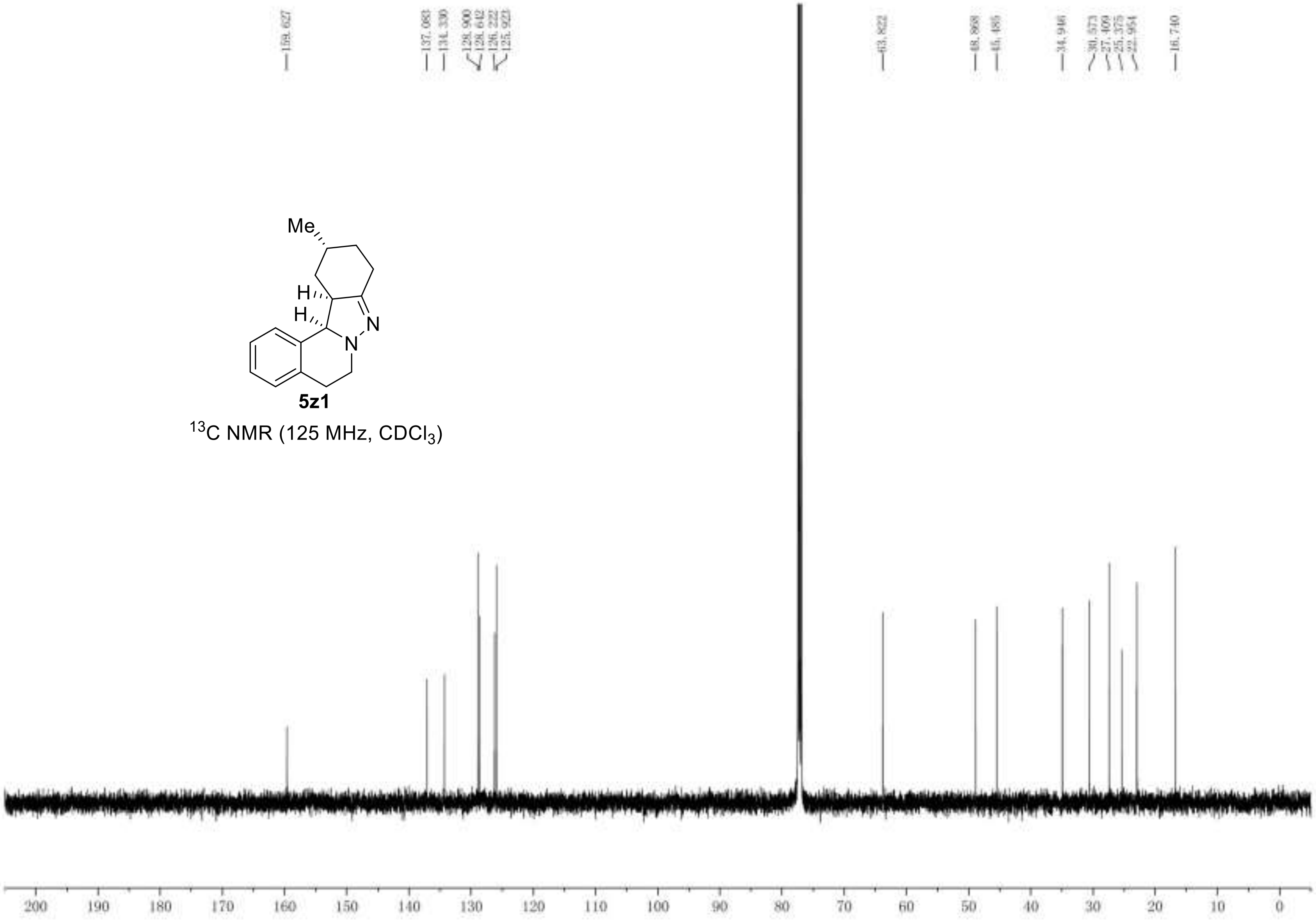

5z1

^{13}C NMR (125 MHz, $CDCl_3$)

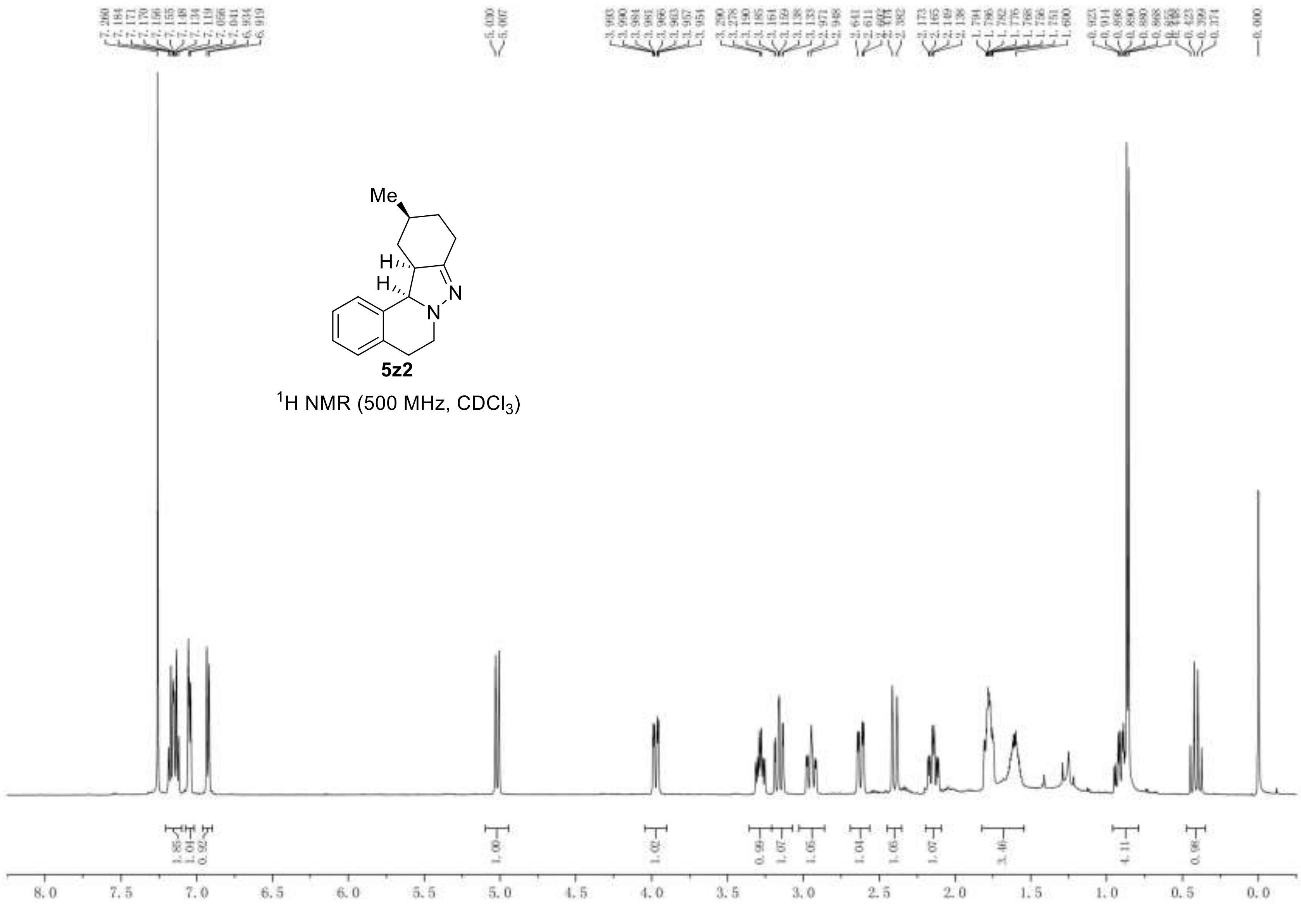

5z2

^{1}H NMR (500 MHz, $CDCl_3$)

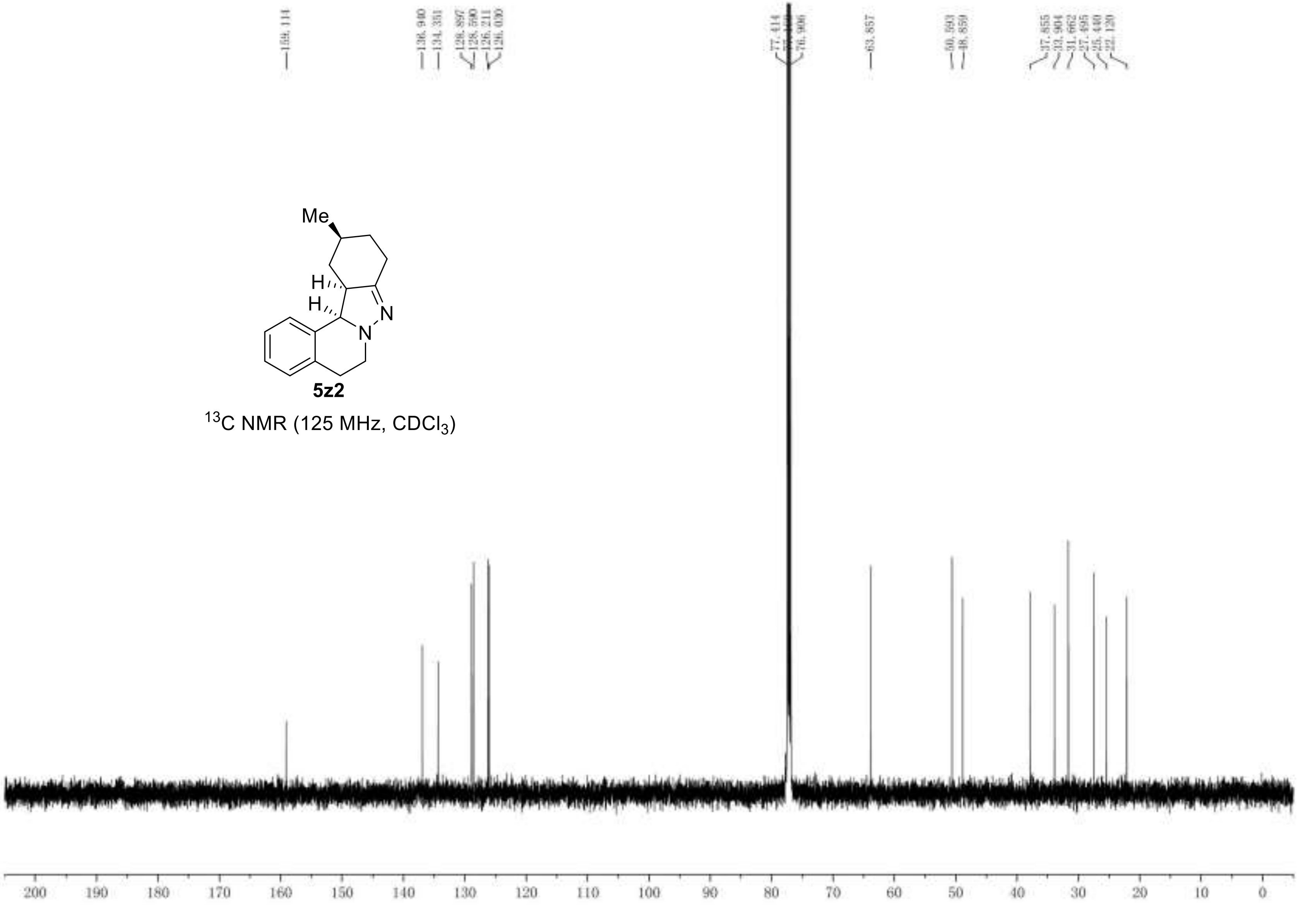

5z2

^{13}C NMR (125 MHz, $CDCl_3$)

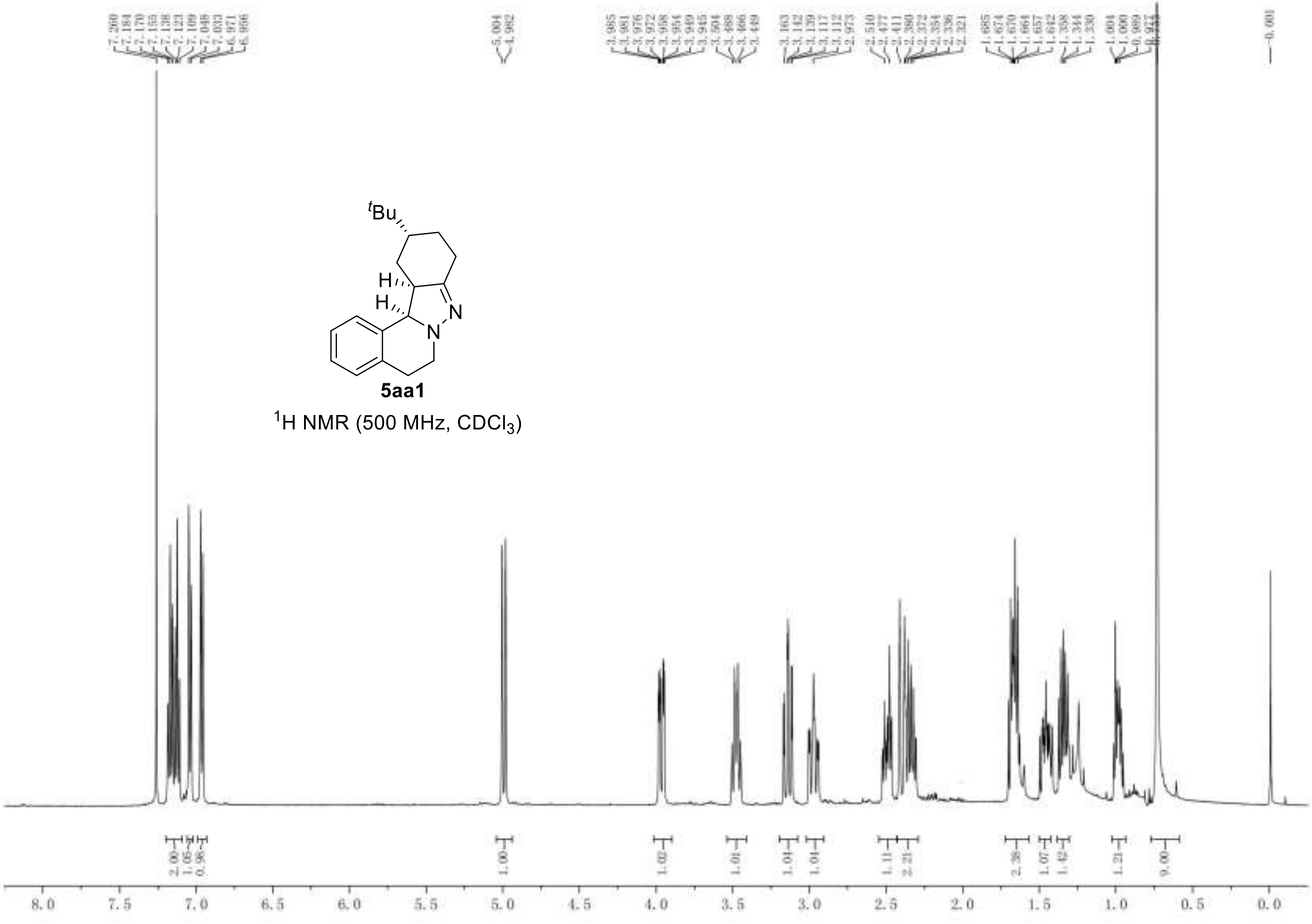

5aa1

^1H NMR (500 MHz, $CDCl_3$)

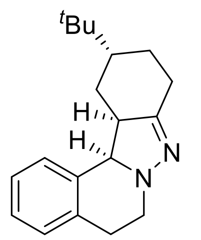

5aa1

^{13}C NMR (125 MHz, $CDCl_3$)

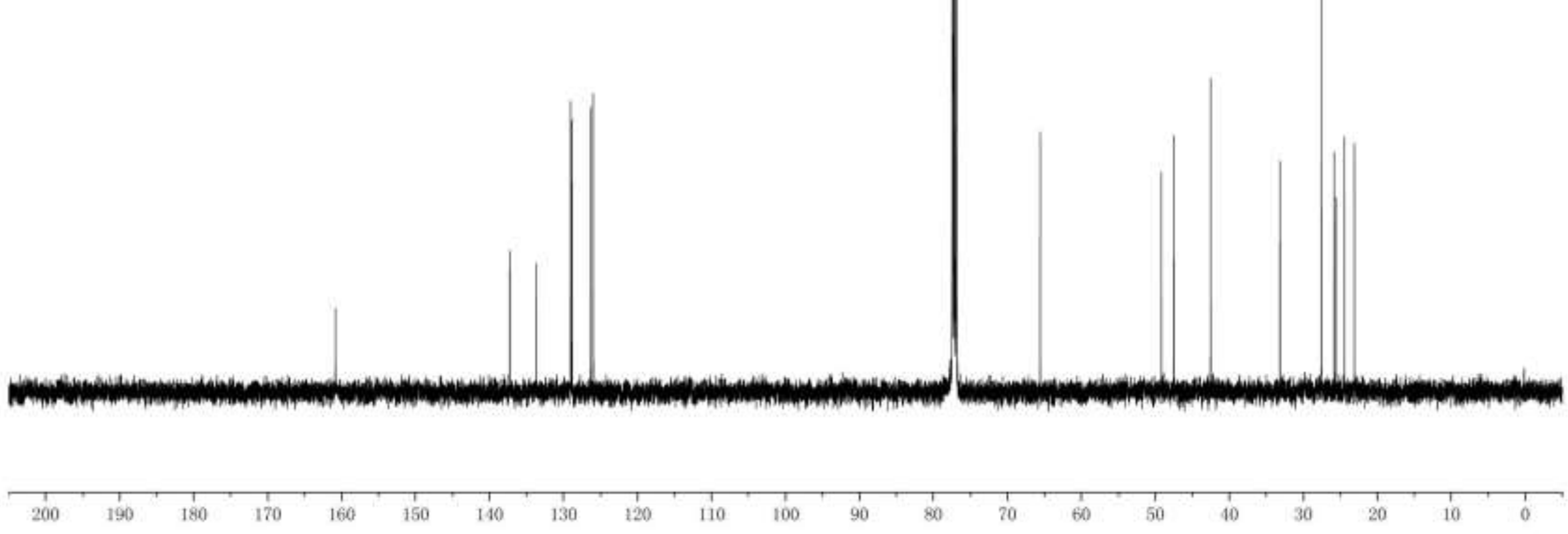

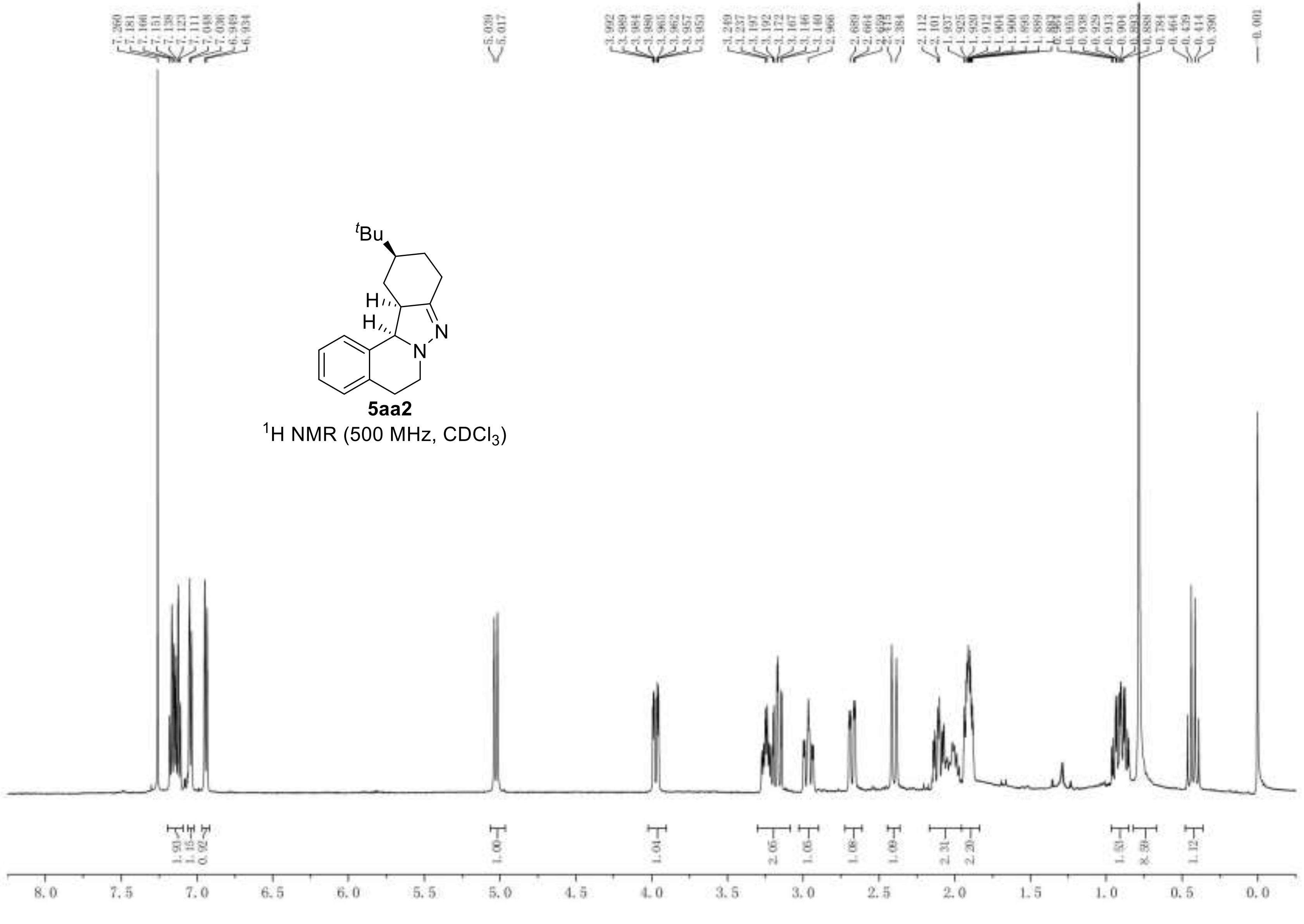

5aa2

^1H NMR (500 MHz, $CDCl_3$)

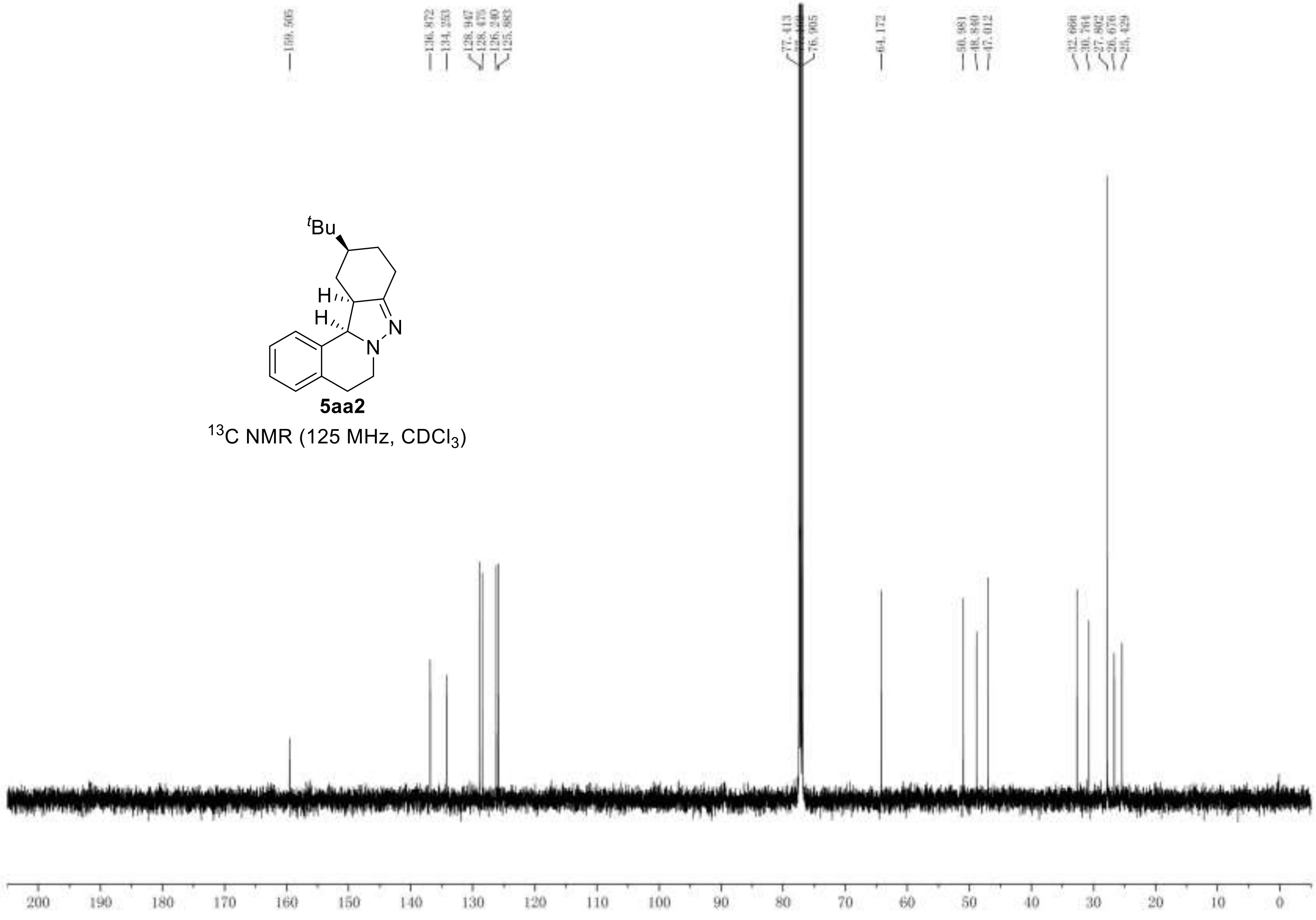

5aa2

^{13}C NMR (125 MHz, $CDCl_3$)

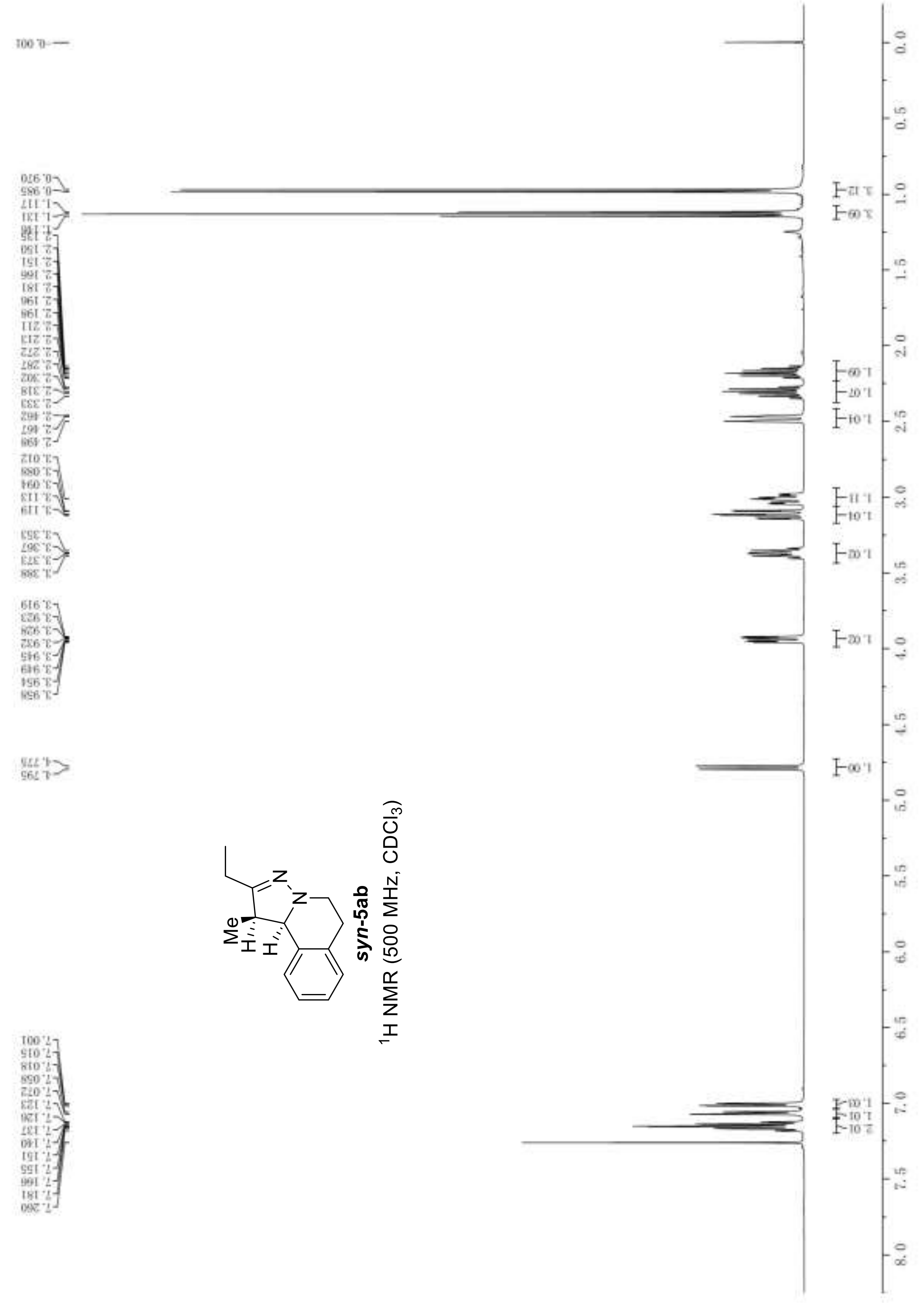

***syn*-5ab**
^{1}H NMR (500 MHz, $CDCl_3$)

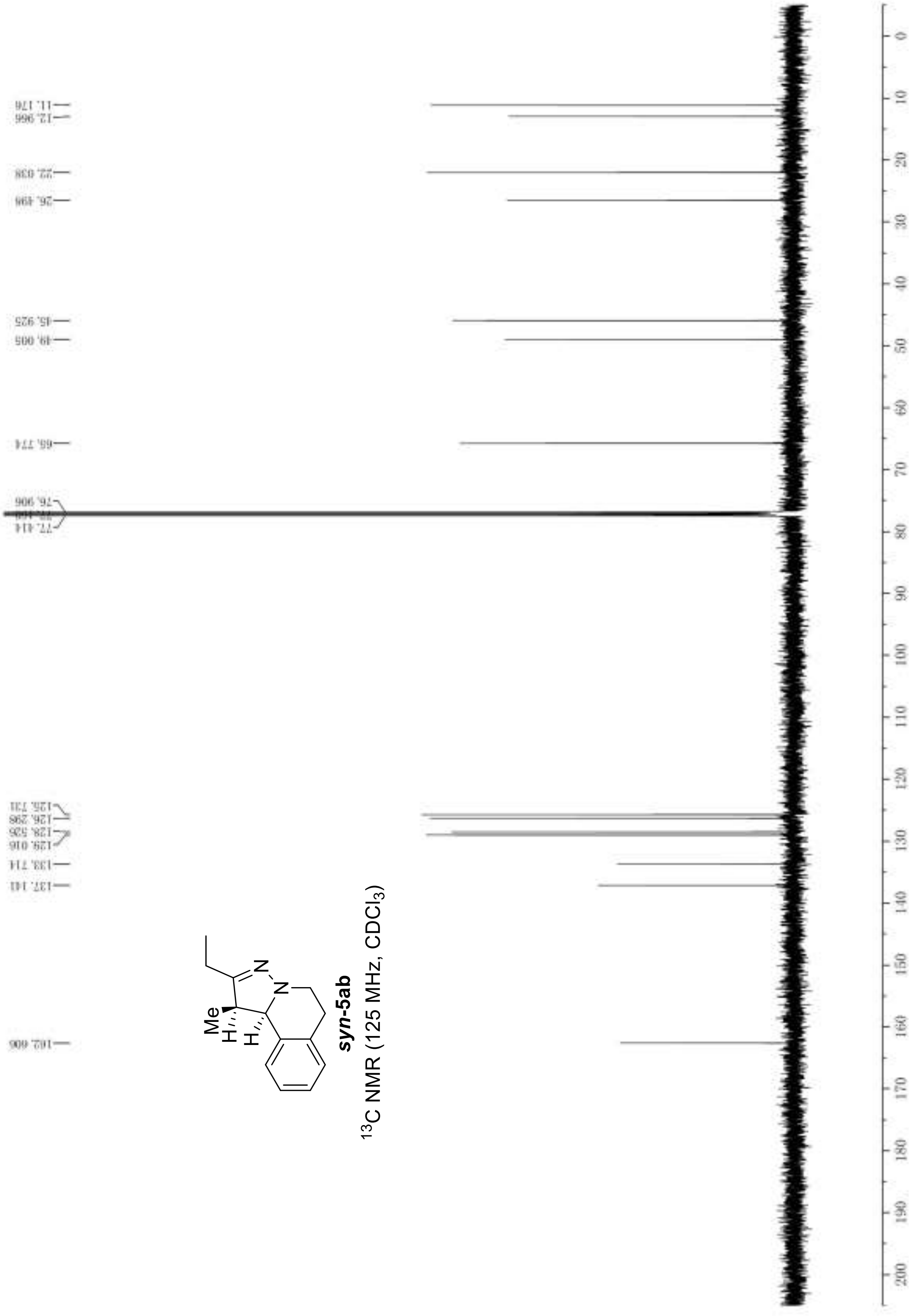

***syn*-5ab**

^{13}C NMR (125 MHz, $CDCl_3$)

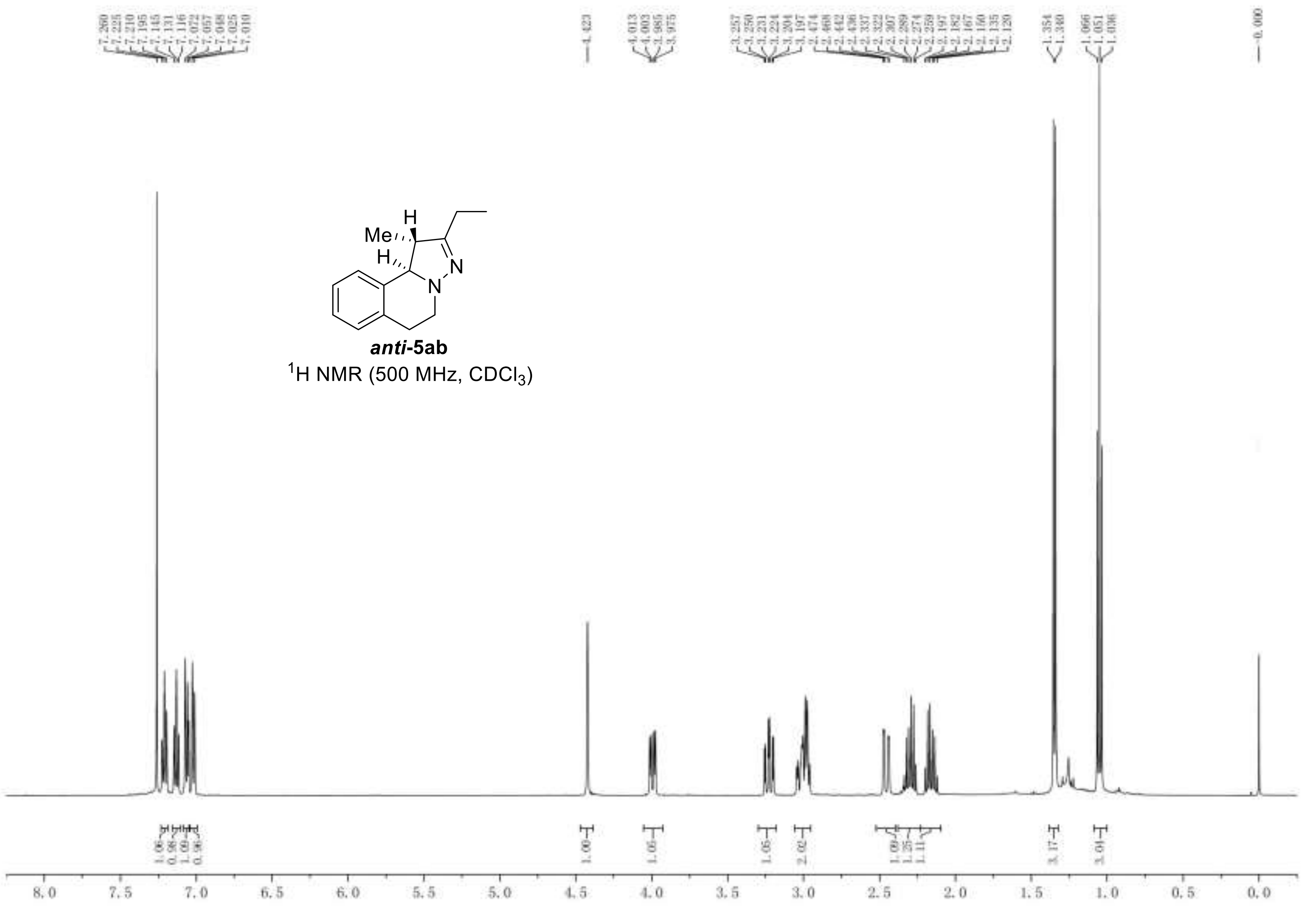

***anti*-5ab**

^{1}H NMR (500 MHz, $CDCl_3$)

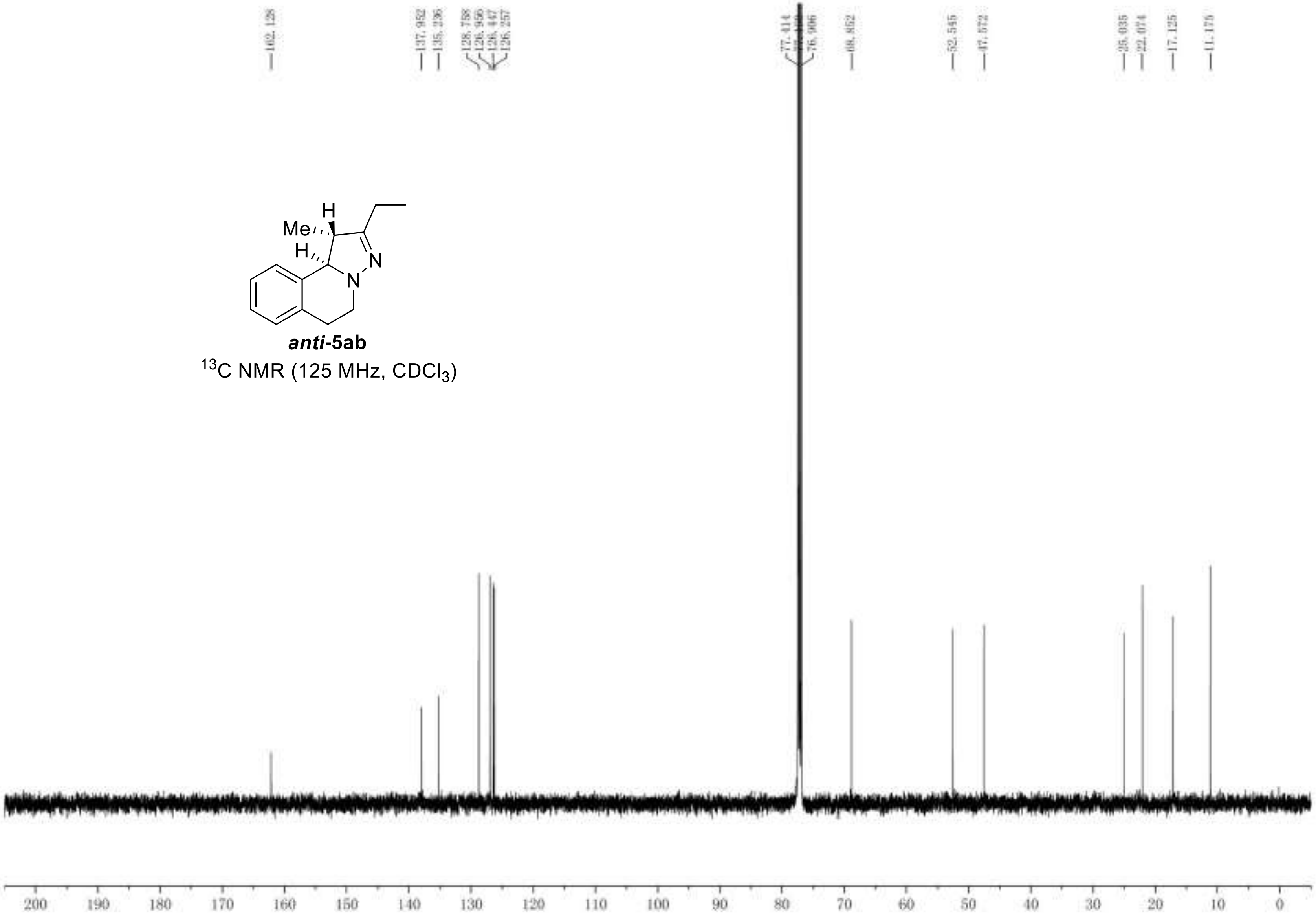

***anti*-5ab**

^{13}C NMR (125 MHz, $CDCl_3$)

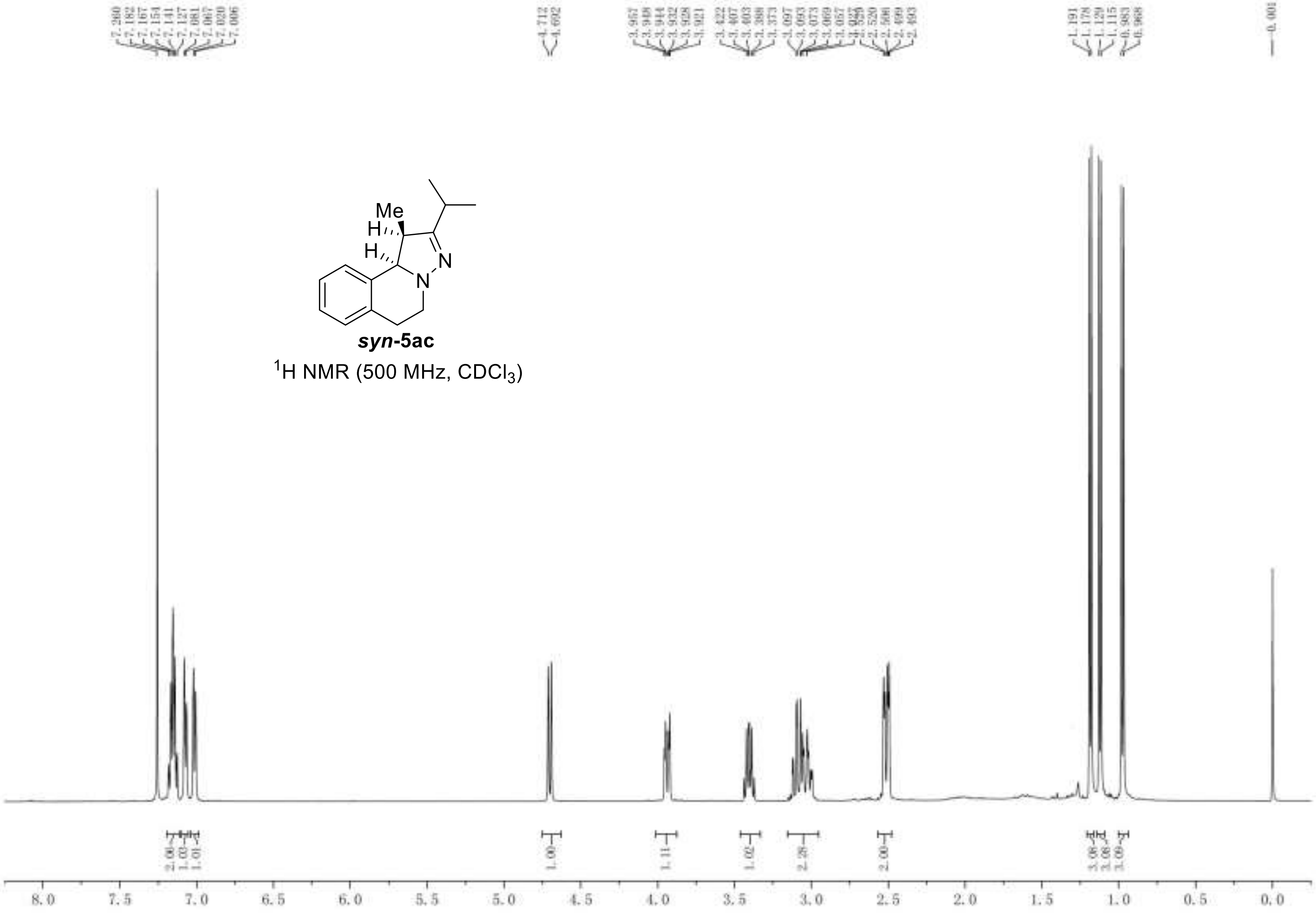

***syn*-5ac**

^{1}H NMR (500 MHz, $CDCl_3$)

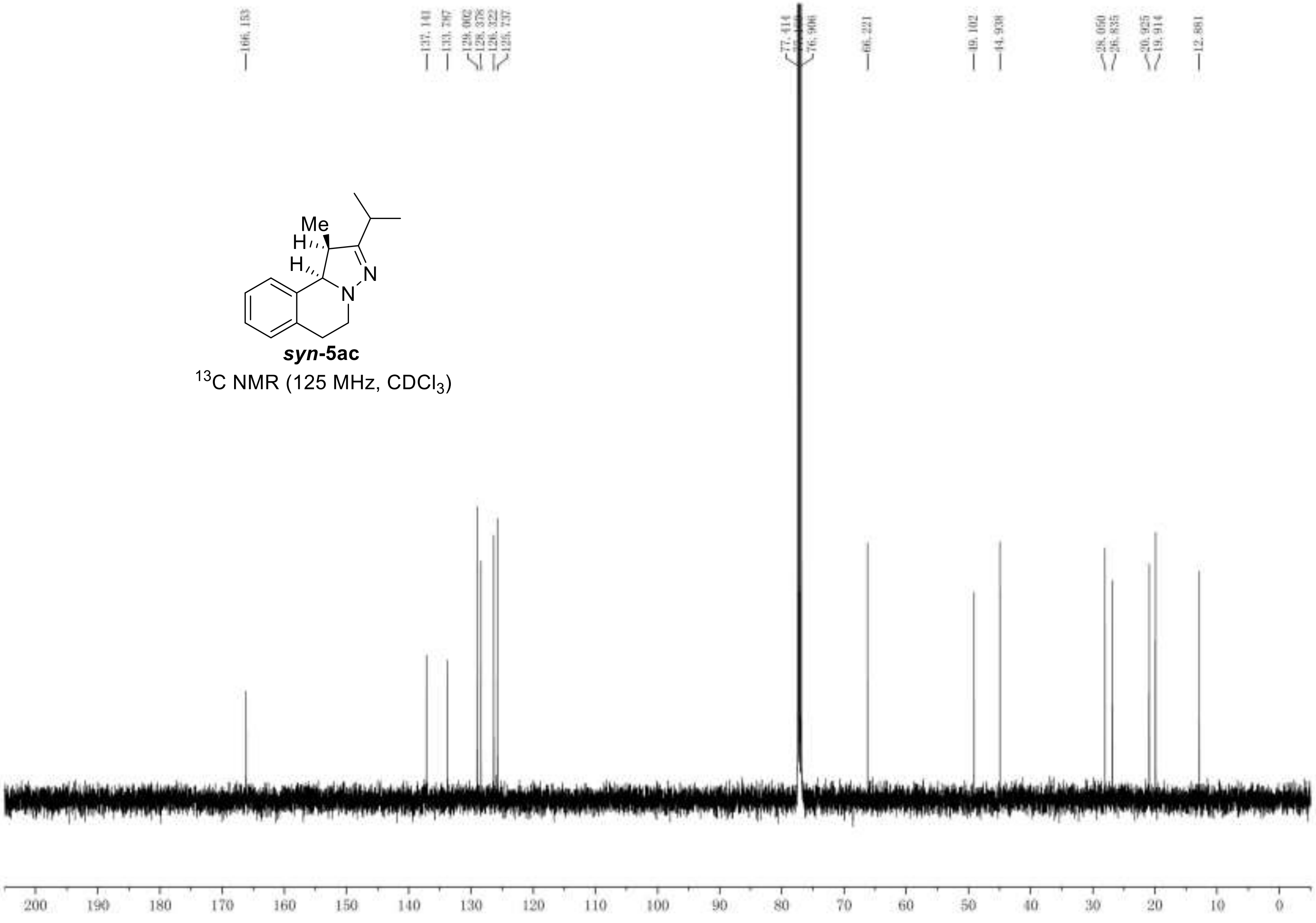

***syn*-5ac**

^{13}C NMR (125 MHz, $CDCl_3$)

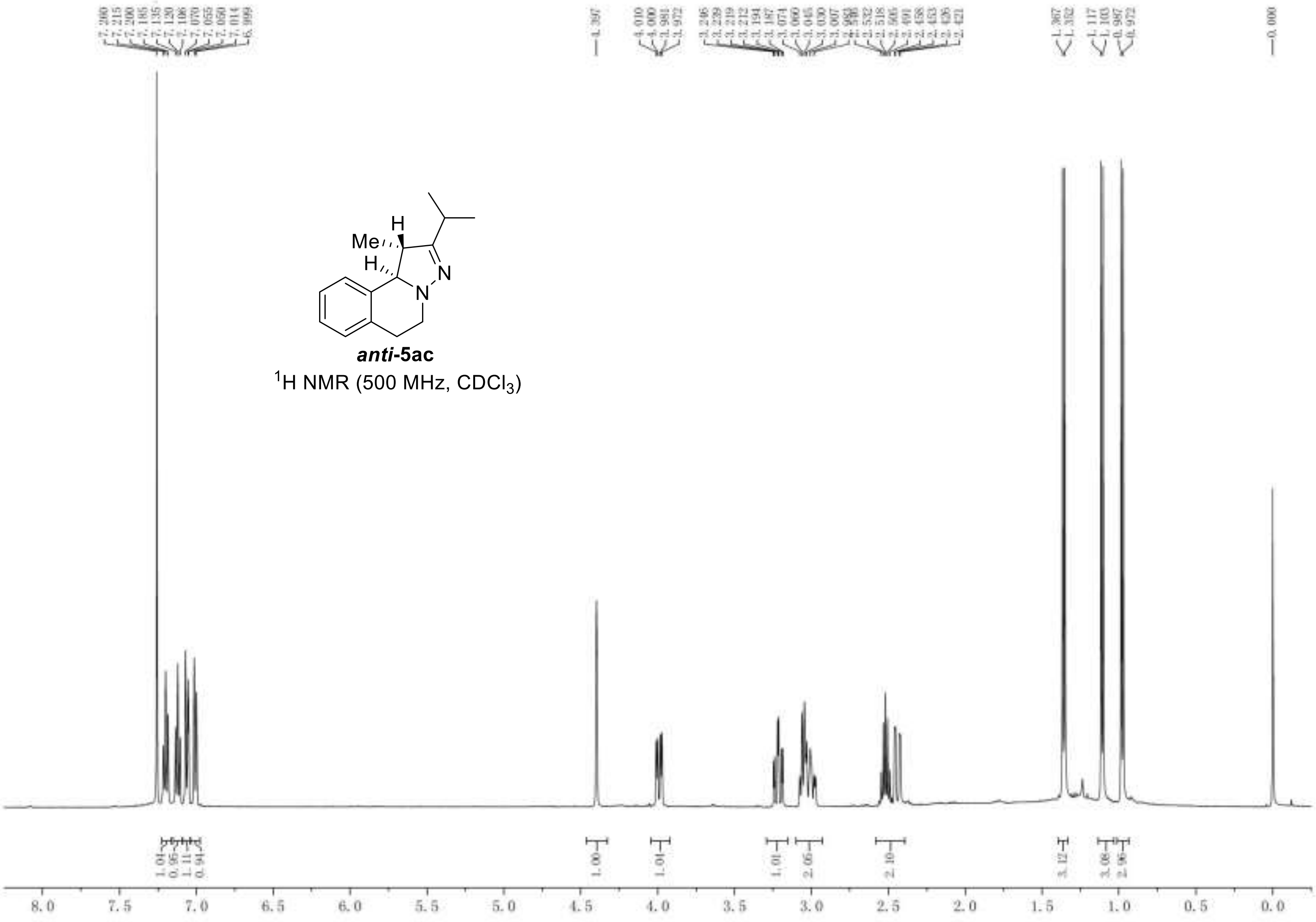

***anti*-5ac**

^{1}H NMR (500 MHz, $CDCl_3$)

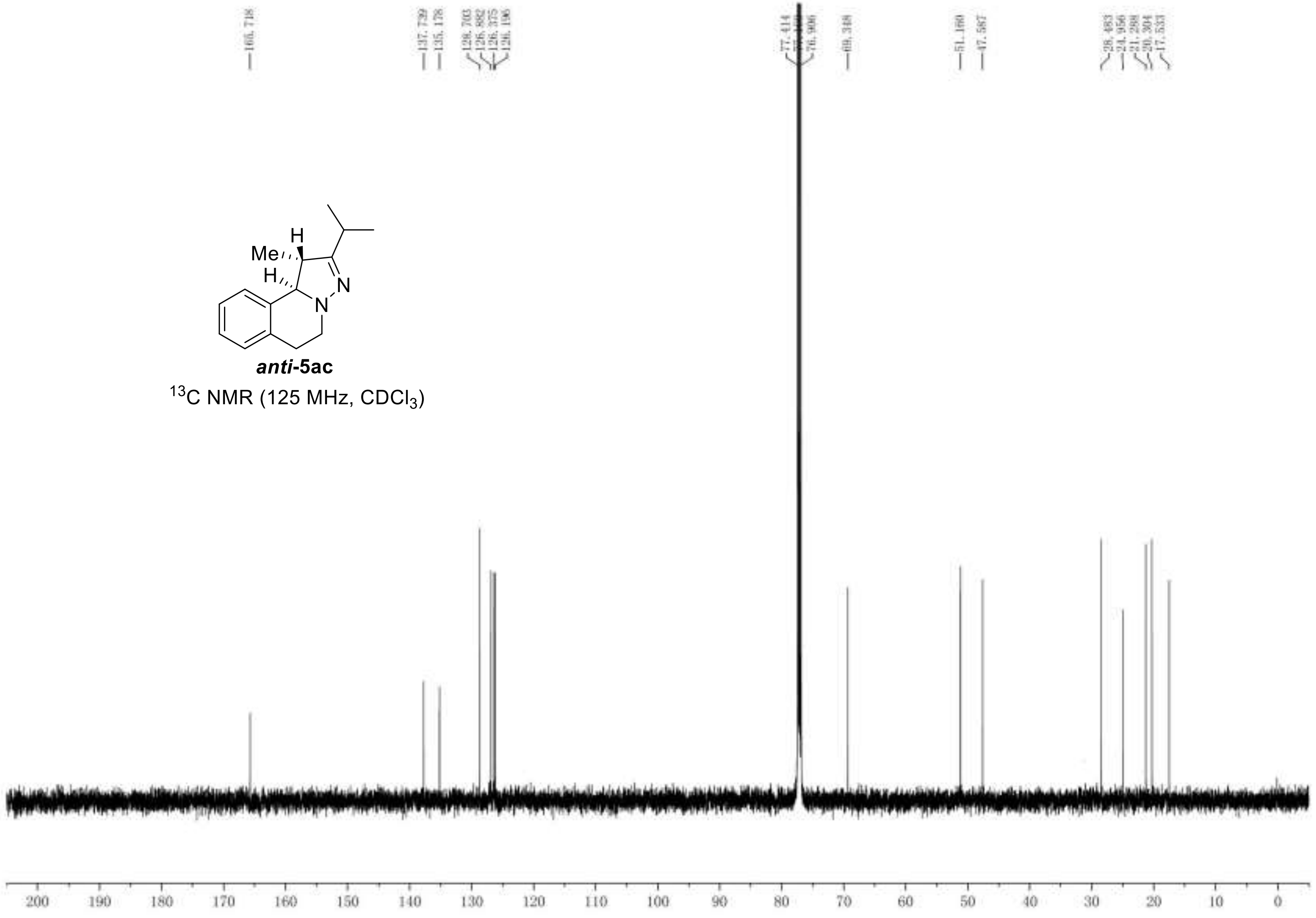

***anti*-5ac**

^{13}C NMR (125 MHz, $CDCl_3$)

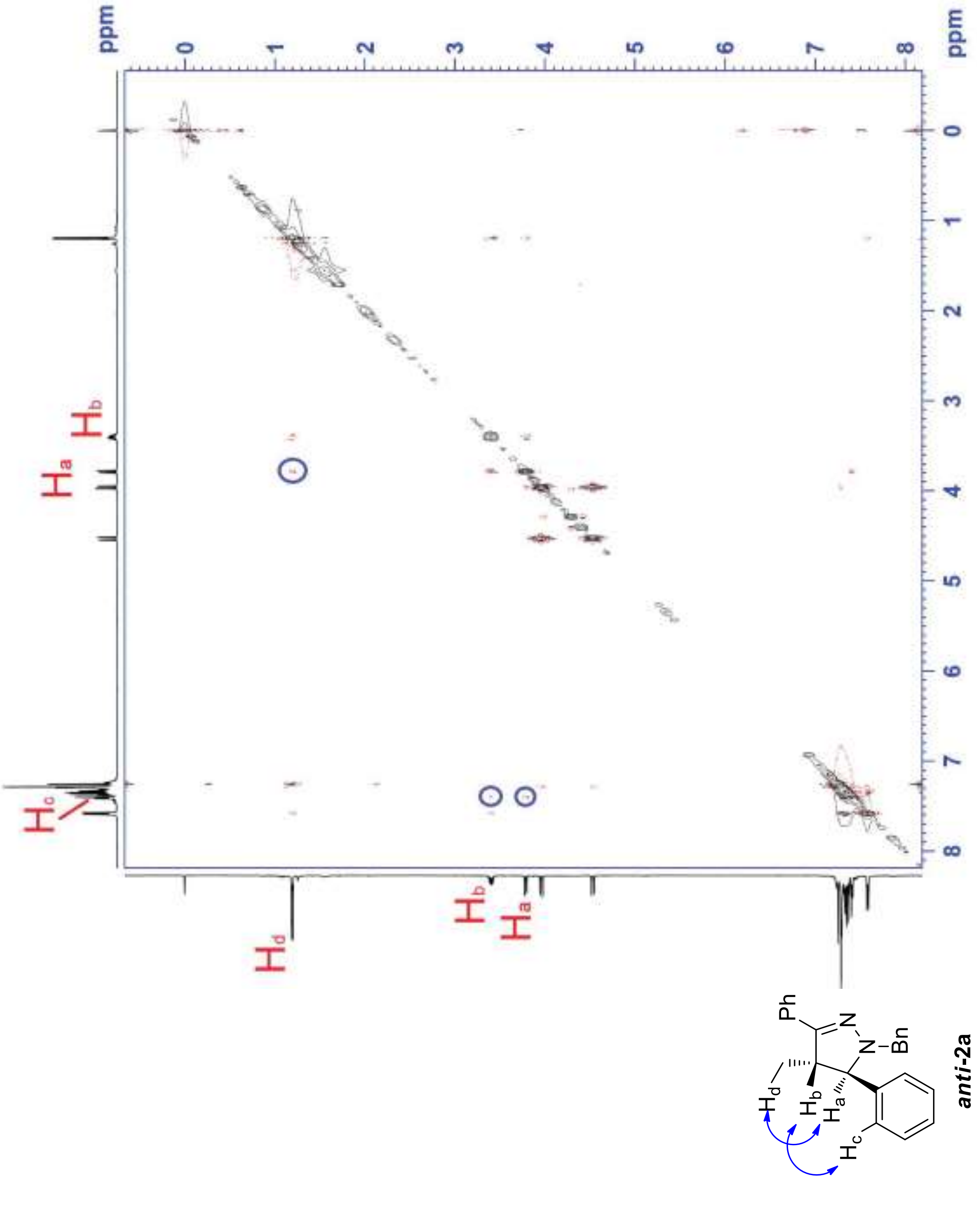

ppm
0
1
2
3
4
5
6
7
8
Ha
Hb
Hc
Hd
Ph
N
N
Bn
anti-2a

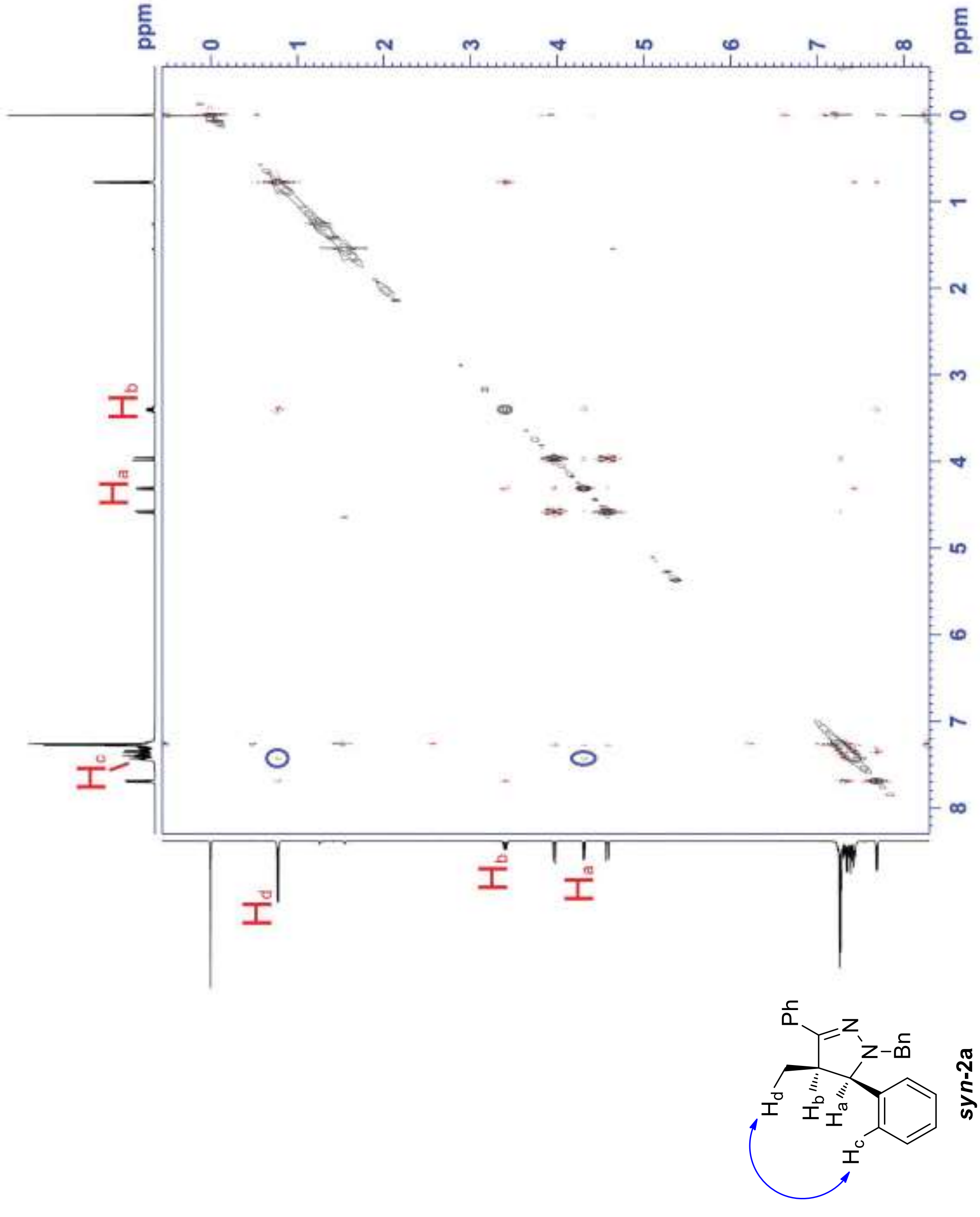
ppm
Ha
Hb
Hc
Hd
Ph
N
N
Bn
syn-2a

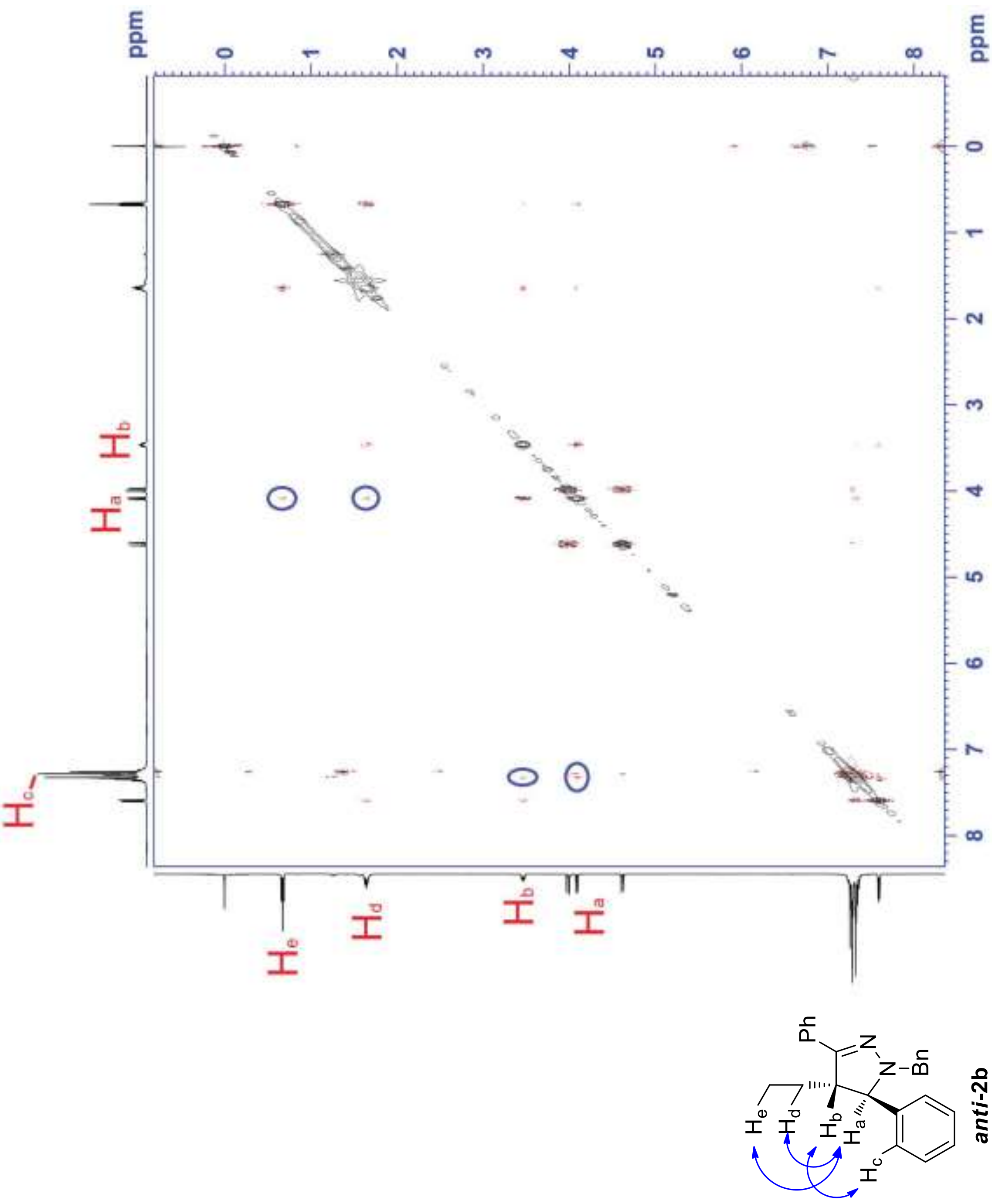

***anti*-2b**

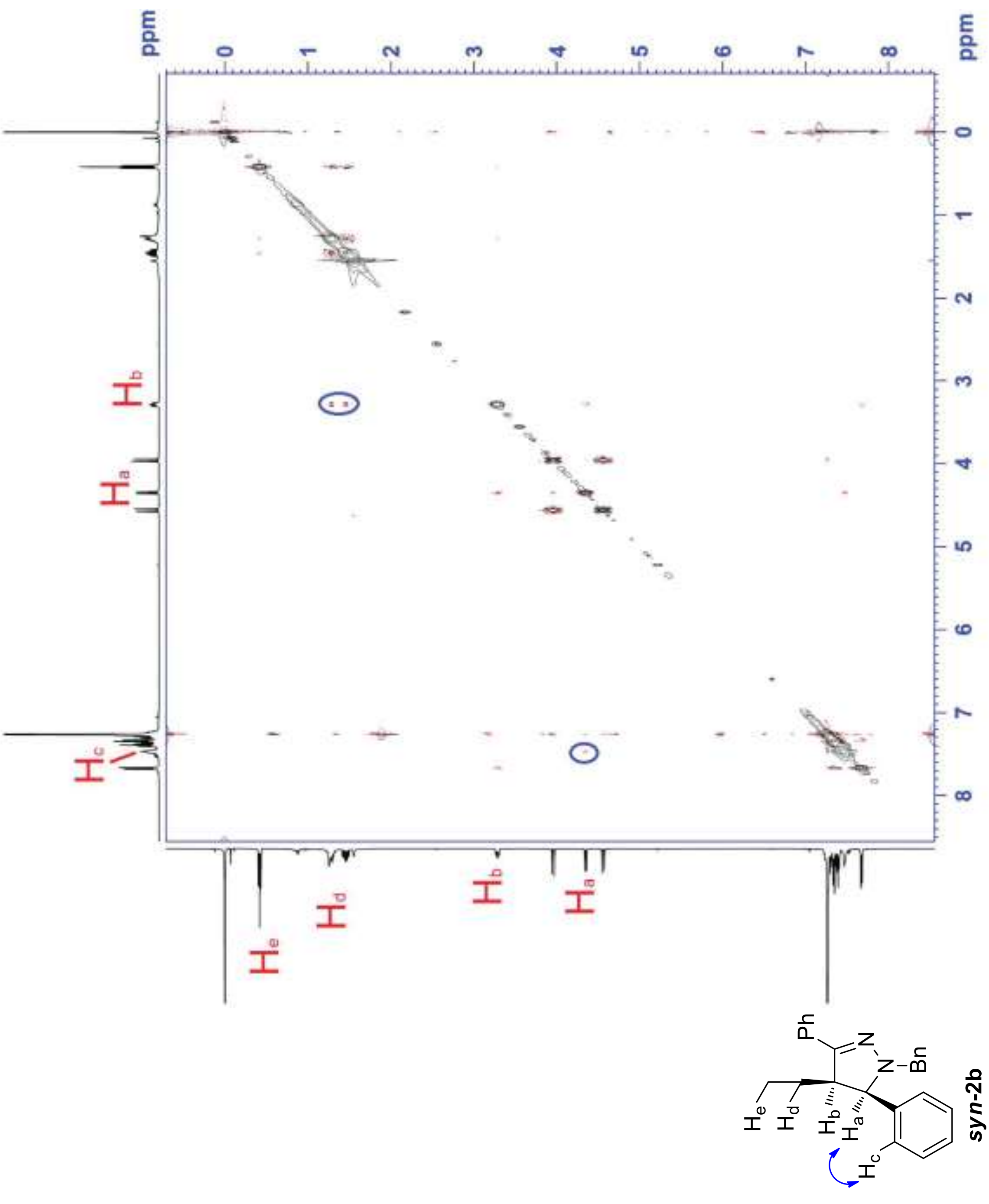
ppm
Ha
Hb
Hc
Hd
He
Ph
N
N
Bn
syn-2b

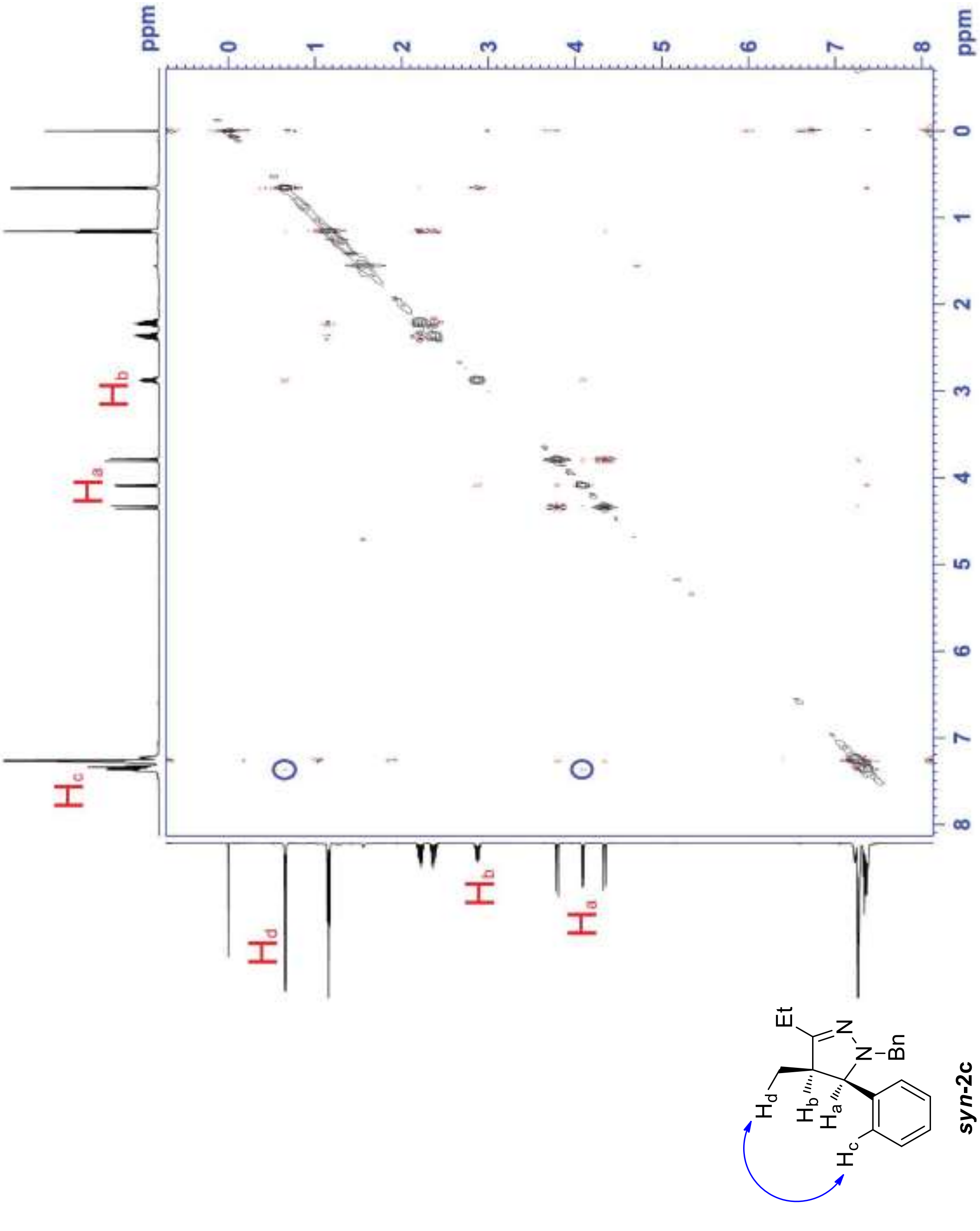
ppm
Hc
Ha
Hb
Hd
Et
N
N–Bn
syn-2c

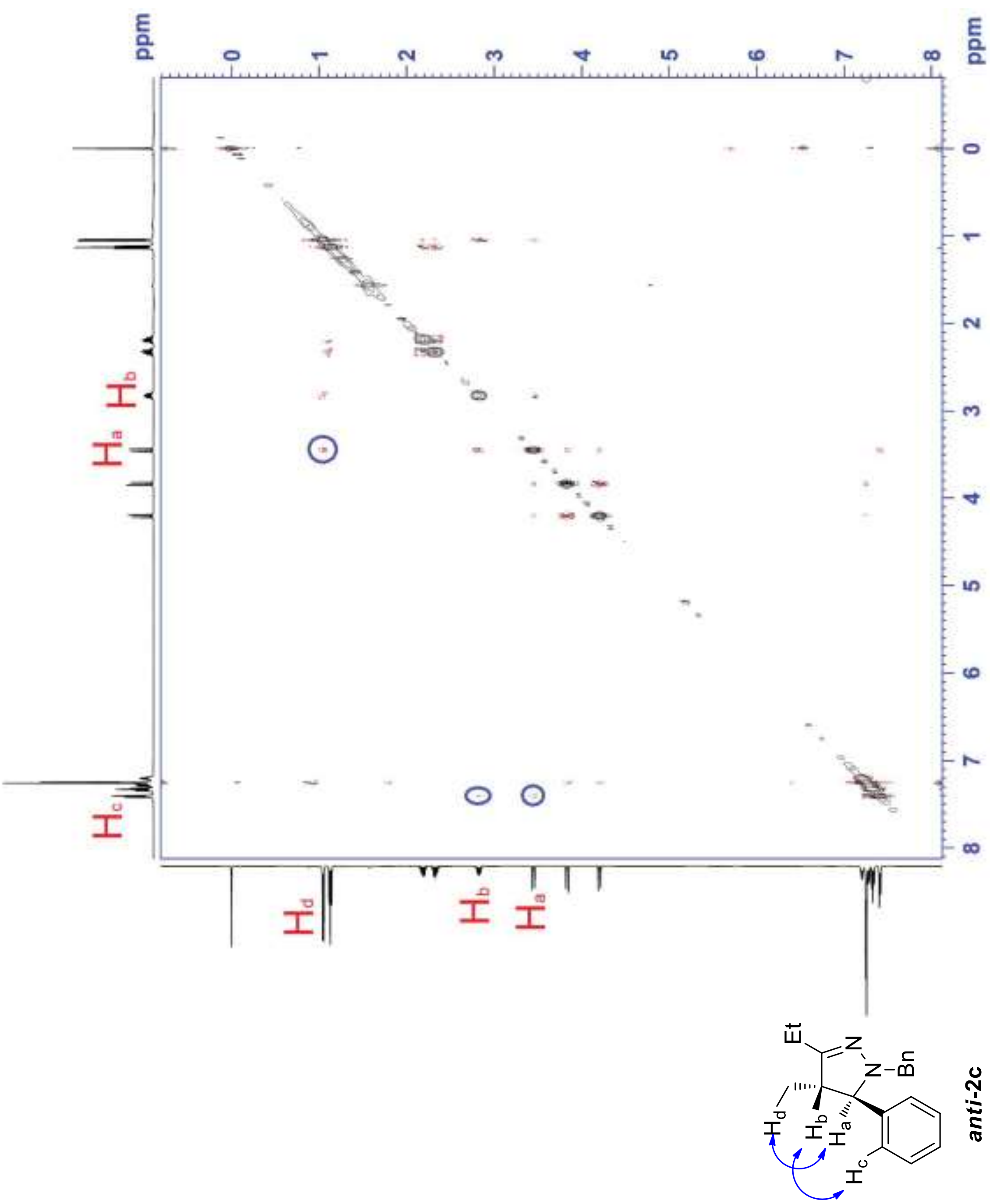

anti-2c

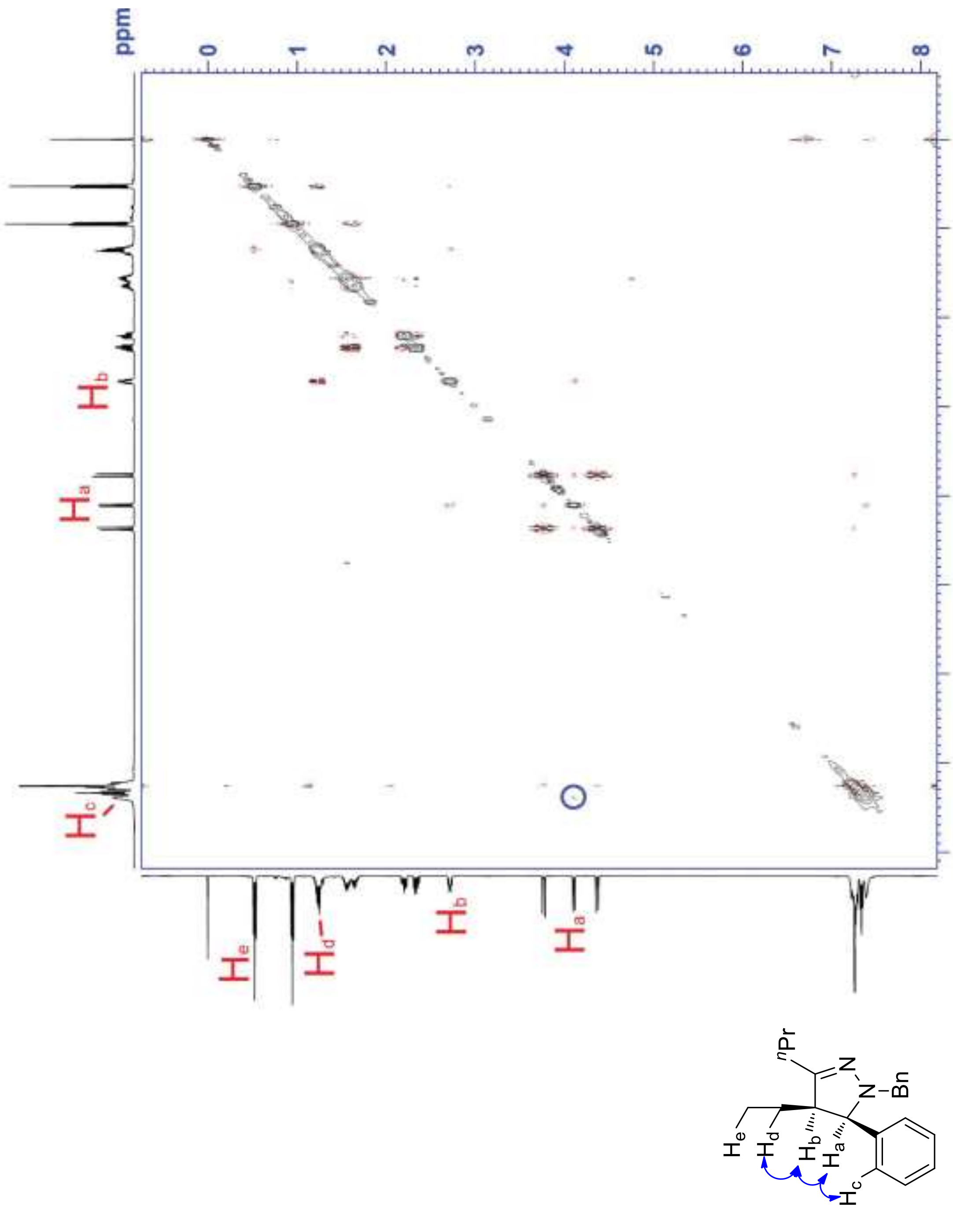

***syn*-2d**

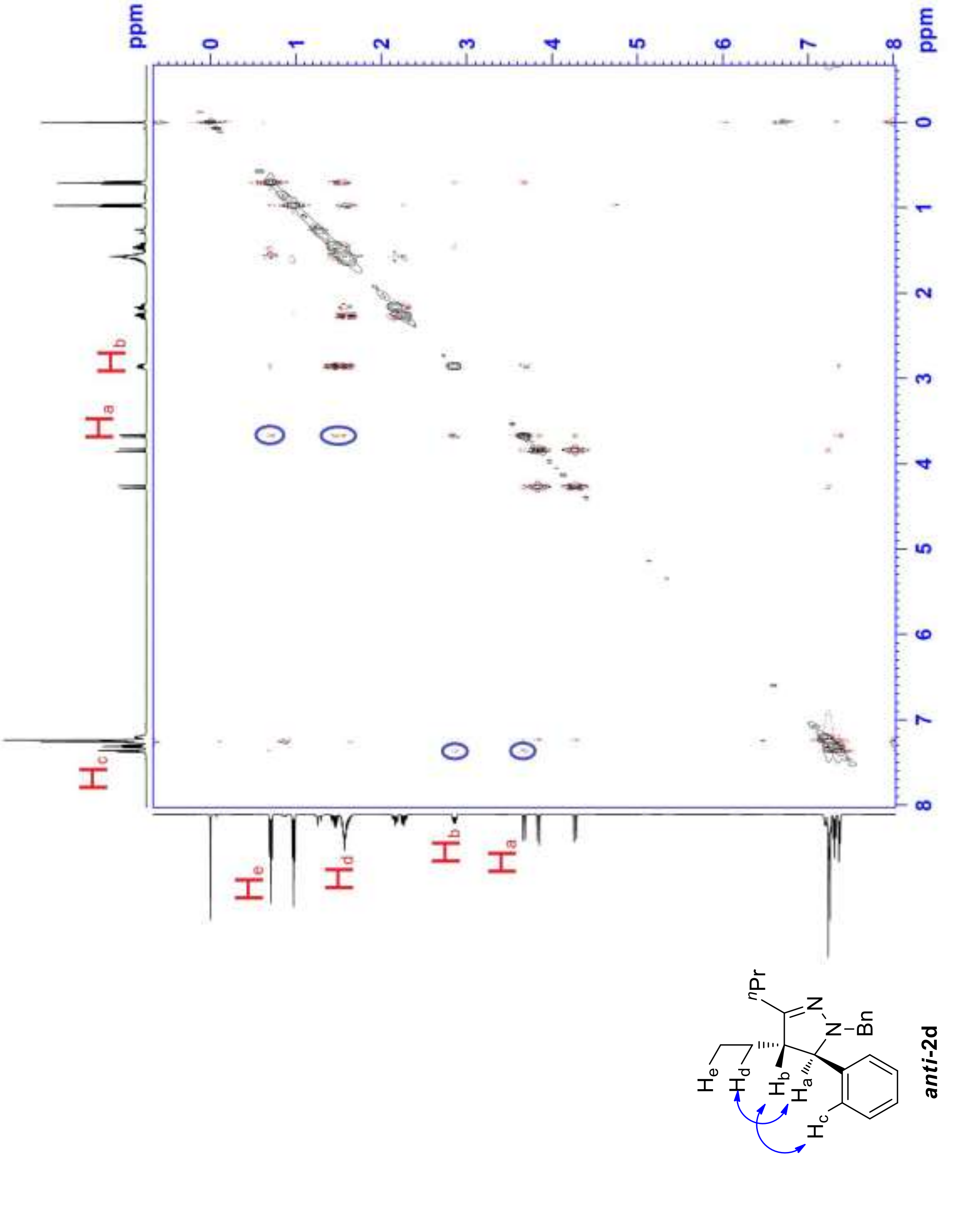

anti-2d

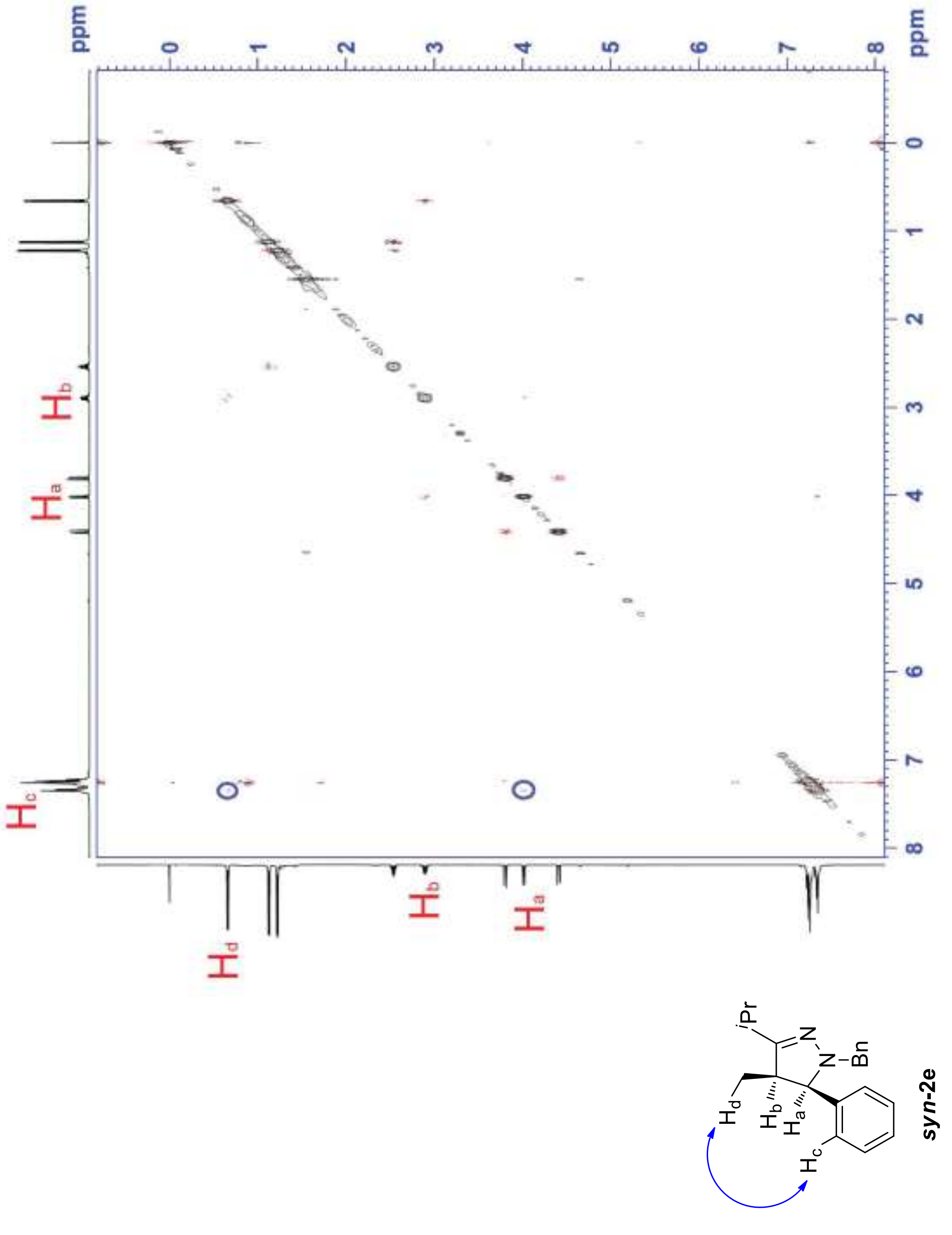

***syn*-2e**

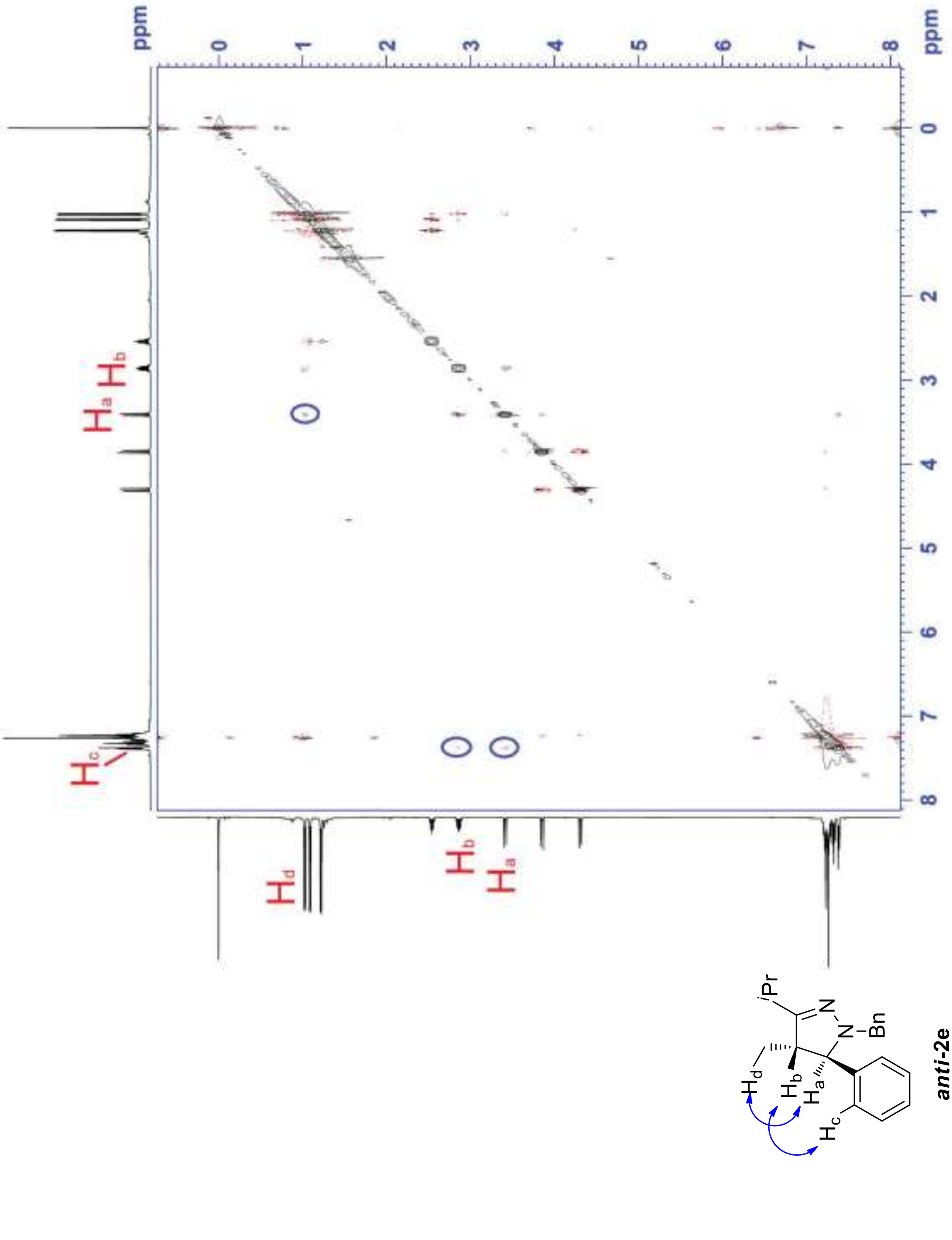

***anti*-2e**

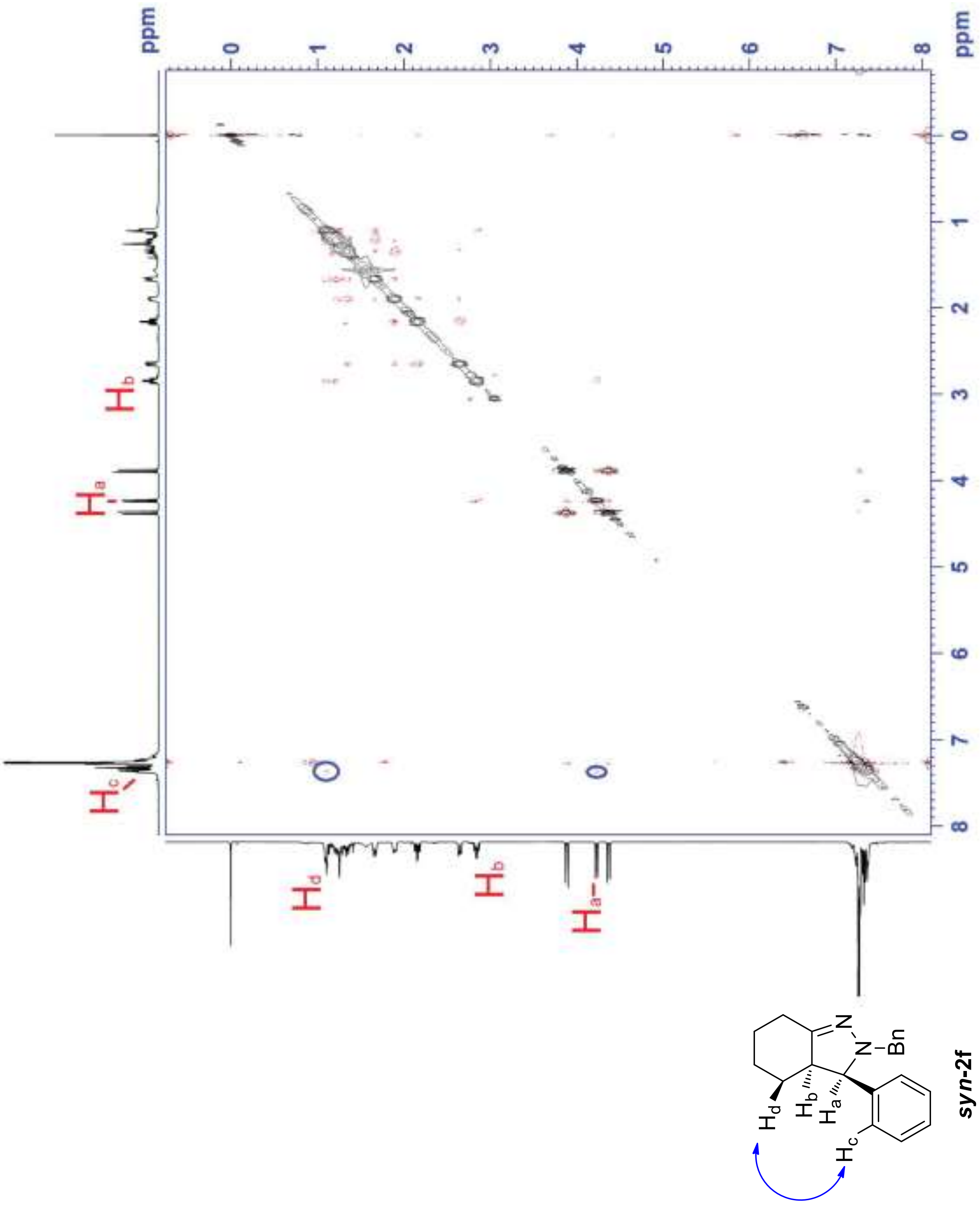

syn-2f

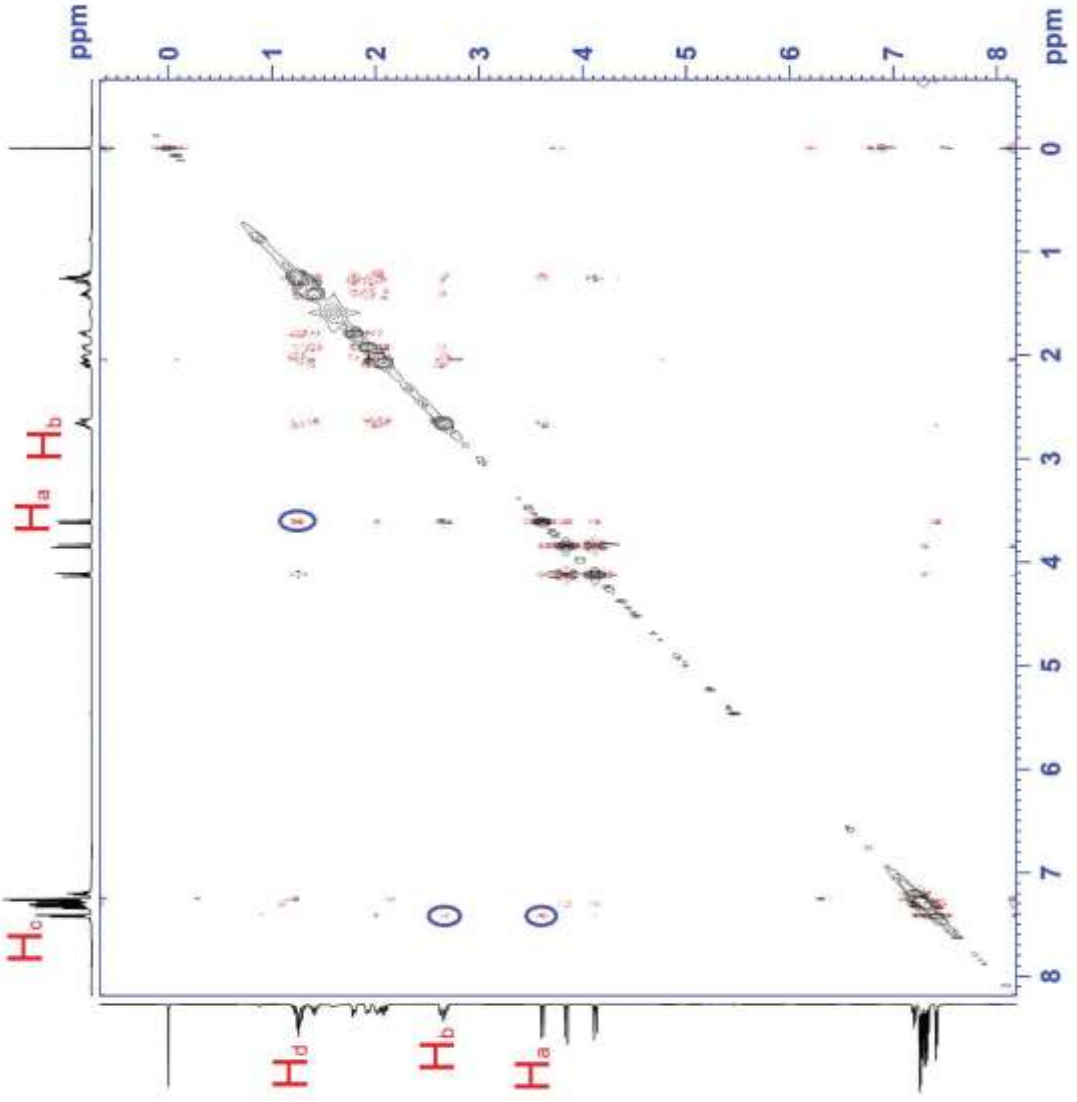

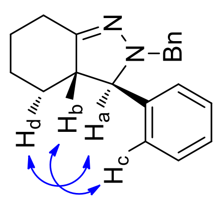

***anti*-2f**

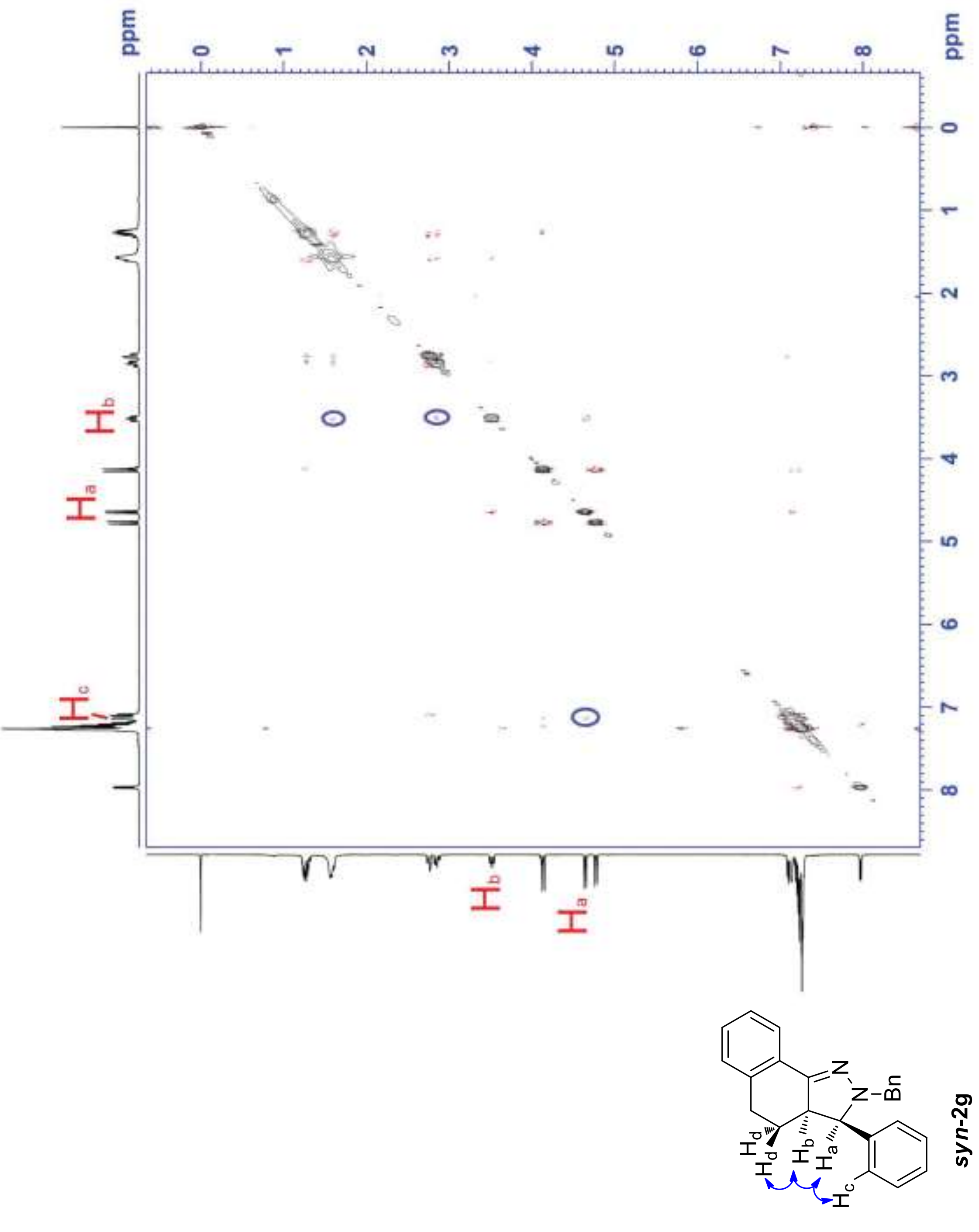
ppm
Ha
Hb
Hc
Hd
Bn
N
syn-2g

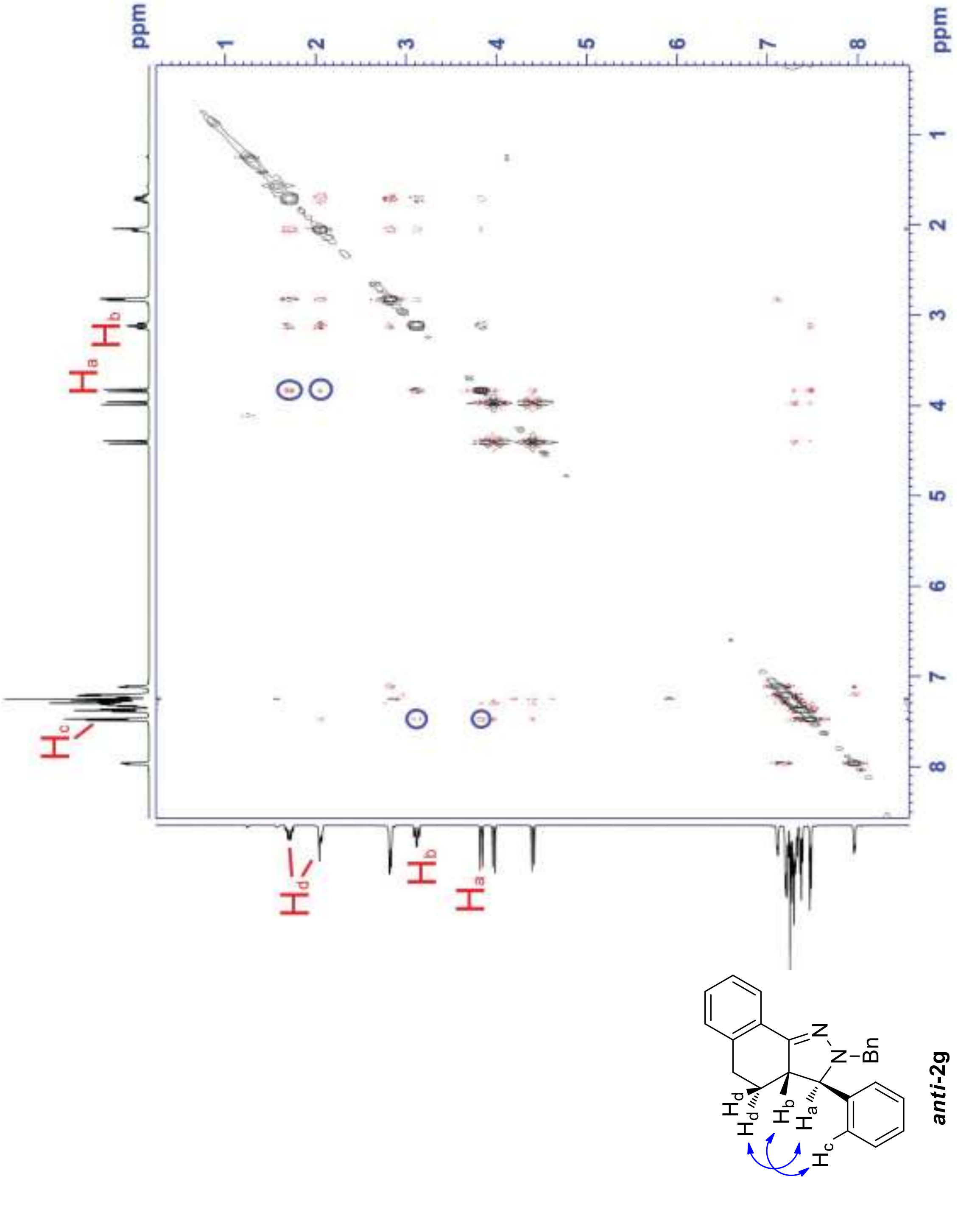

***anti*-2g**

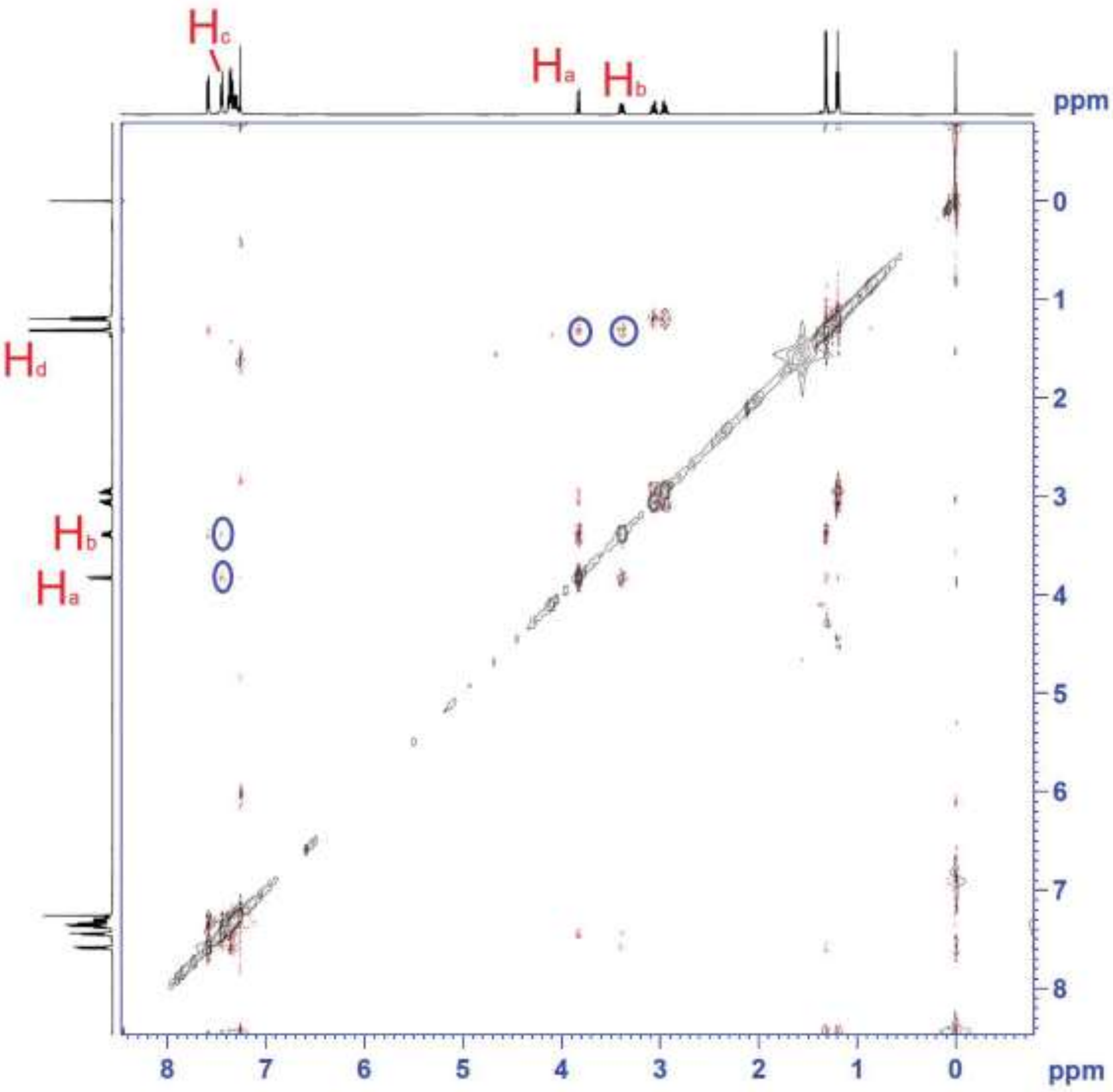

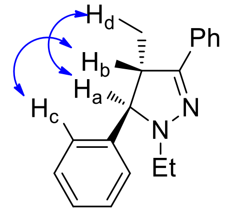

***anti*-2i**

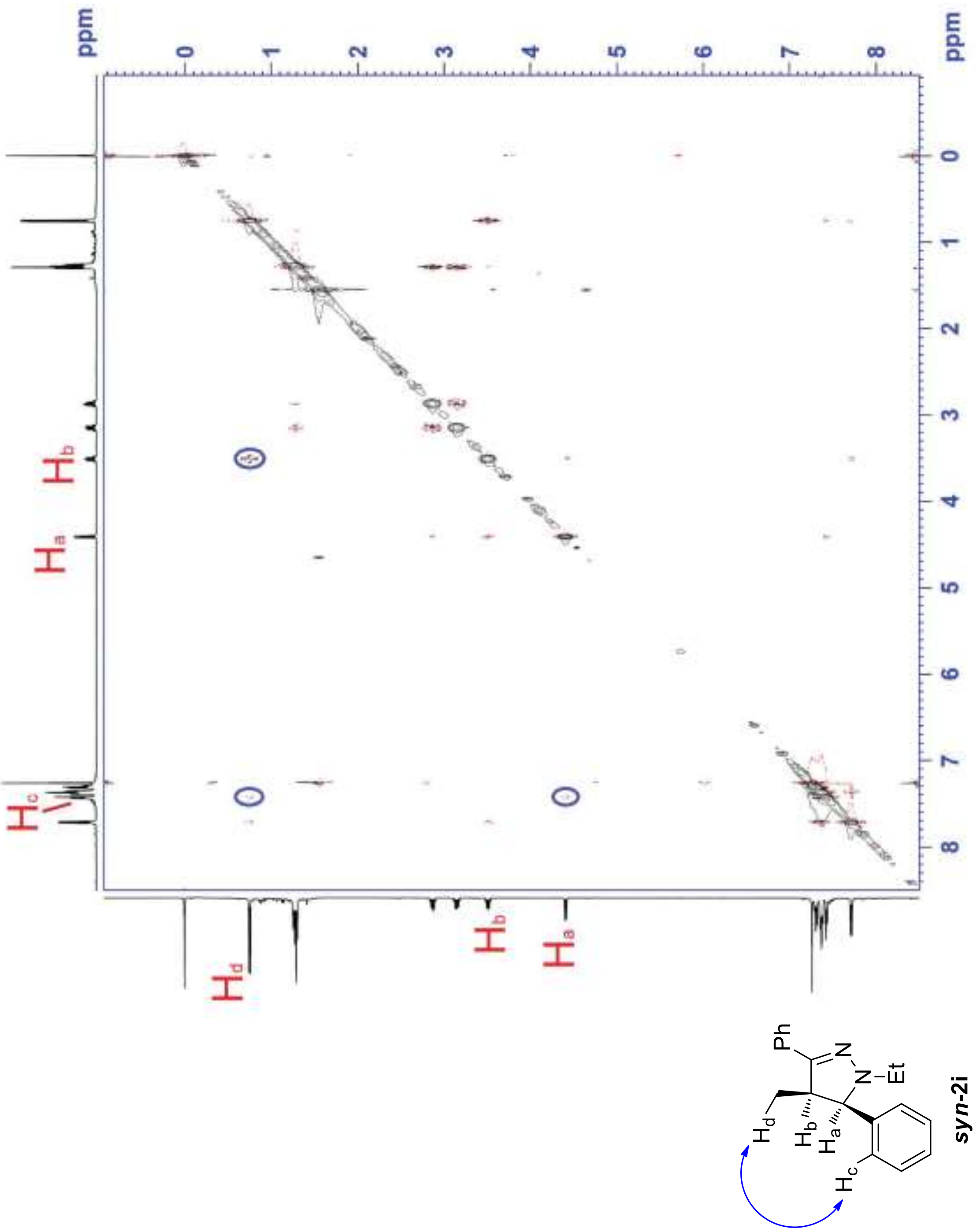

syn-2i

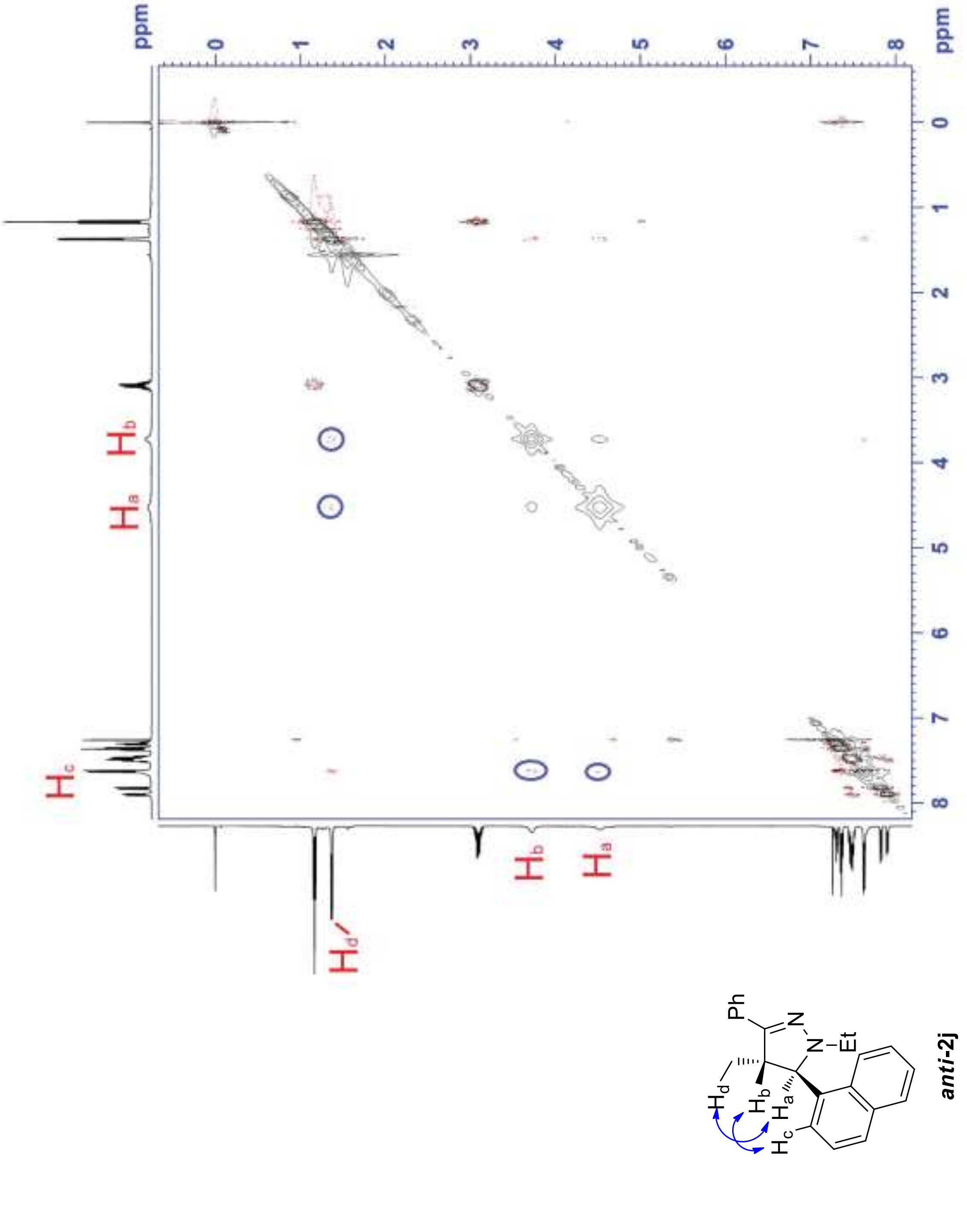

***anti*-2j**

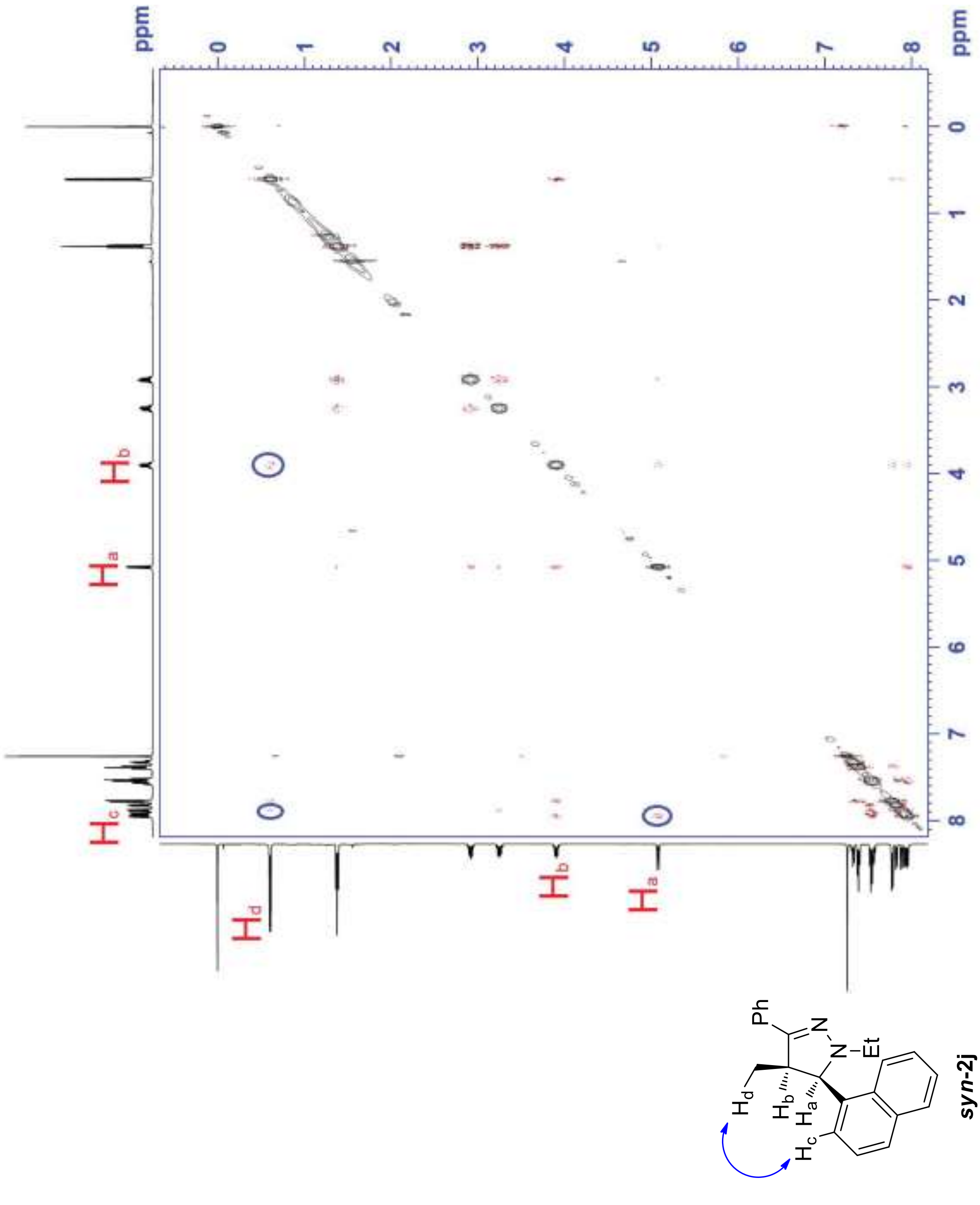
ppm
Ha
Hb
Hc
Hd
Ph
N
N-Et
syn-2j

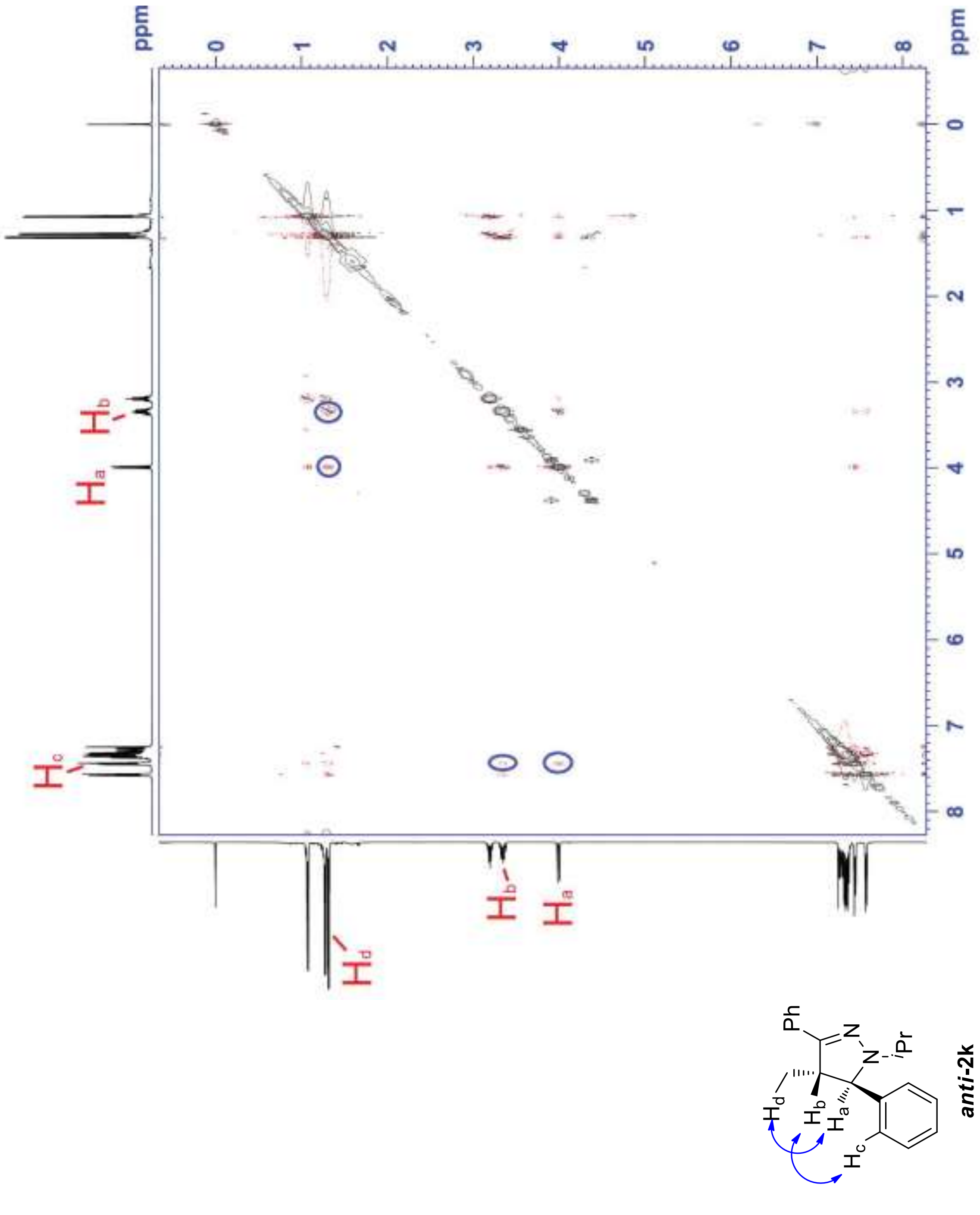

ppm
Ha
Hb
Hc
Hd
Ph
N
N
iPr
anti-2k

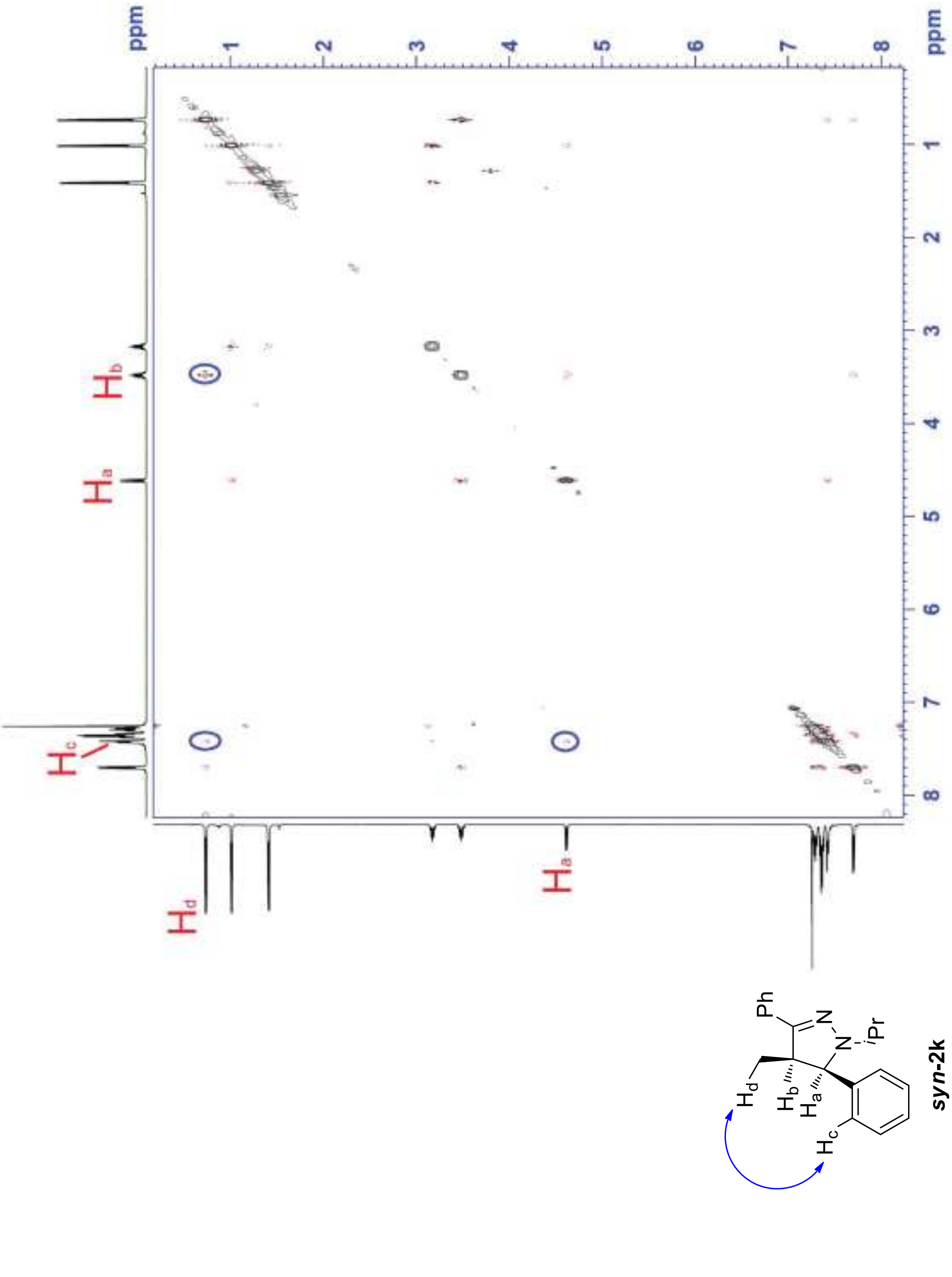

syn-**2k**

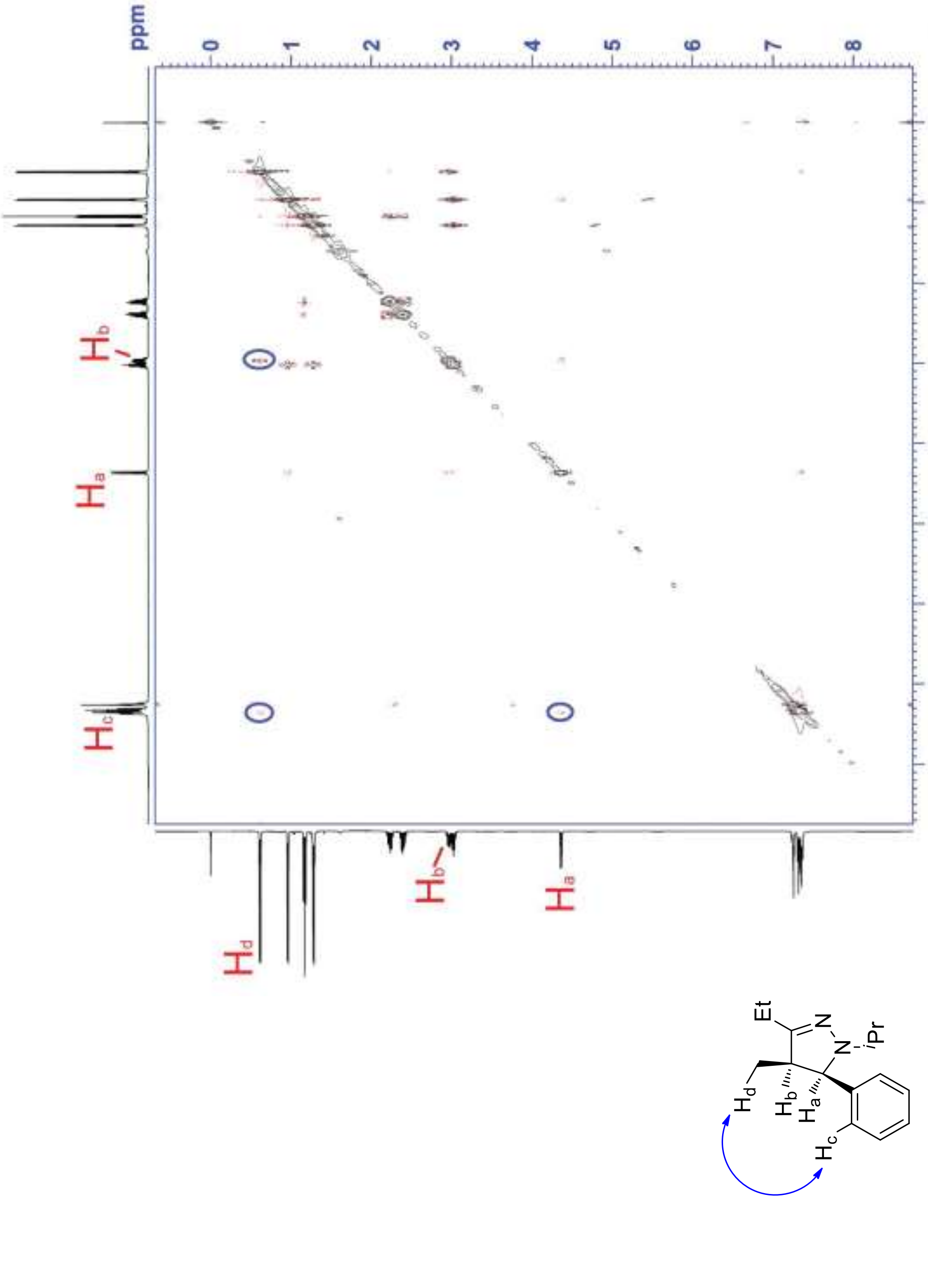

syn-2l

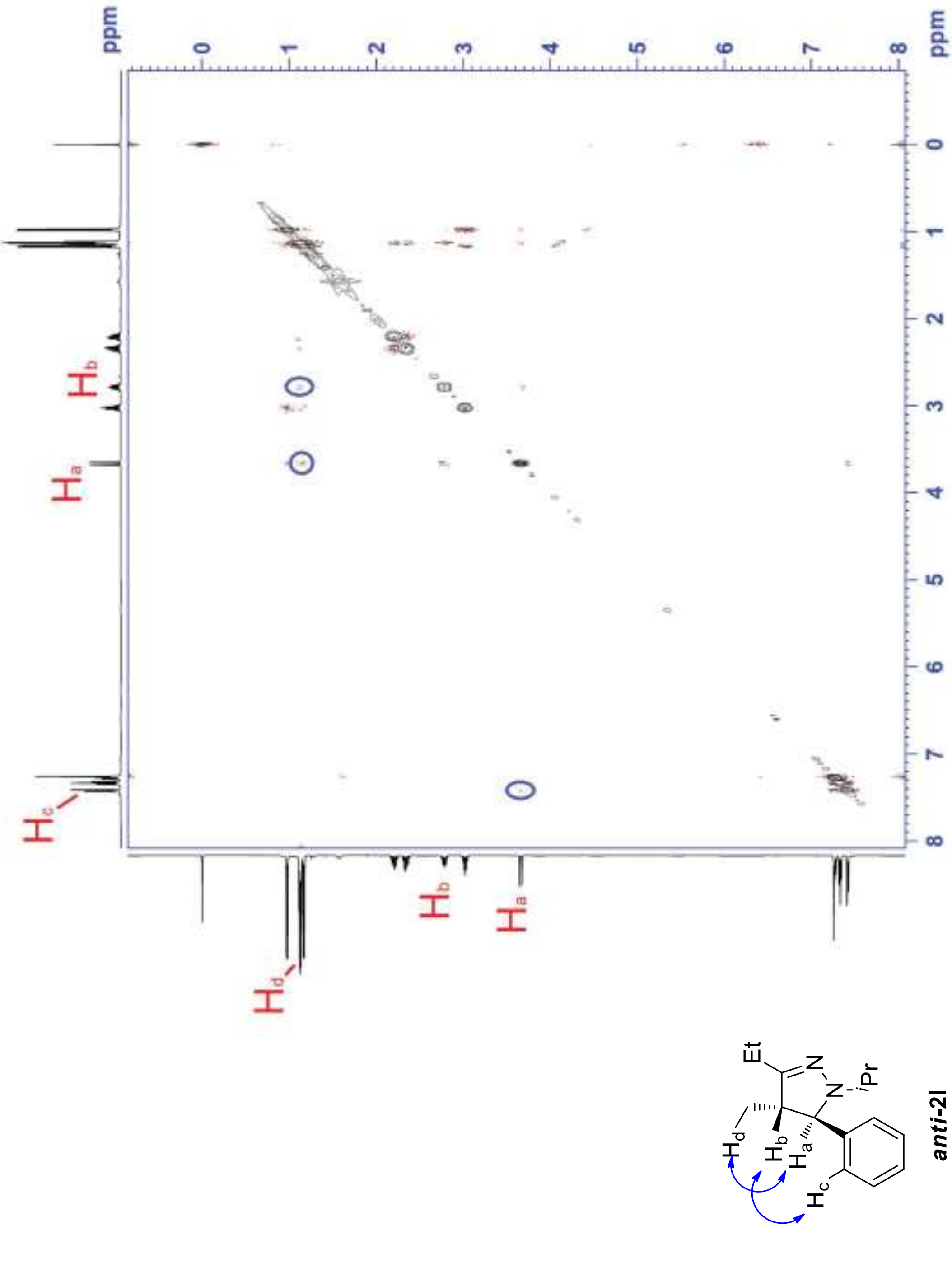

anti-2l

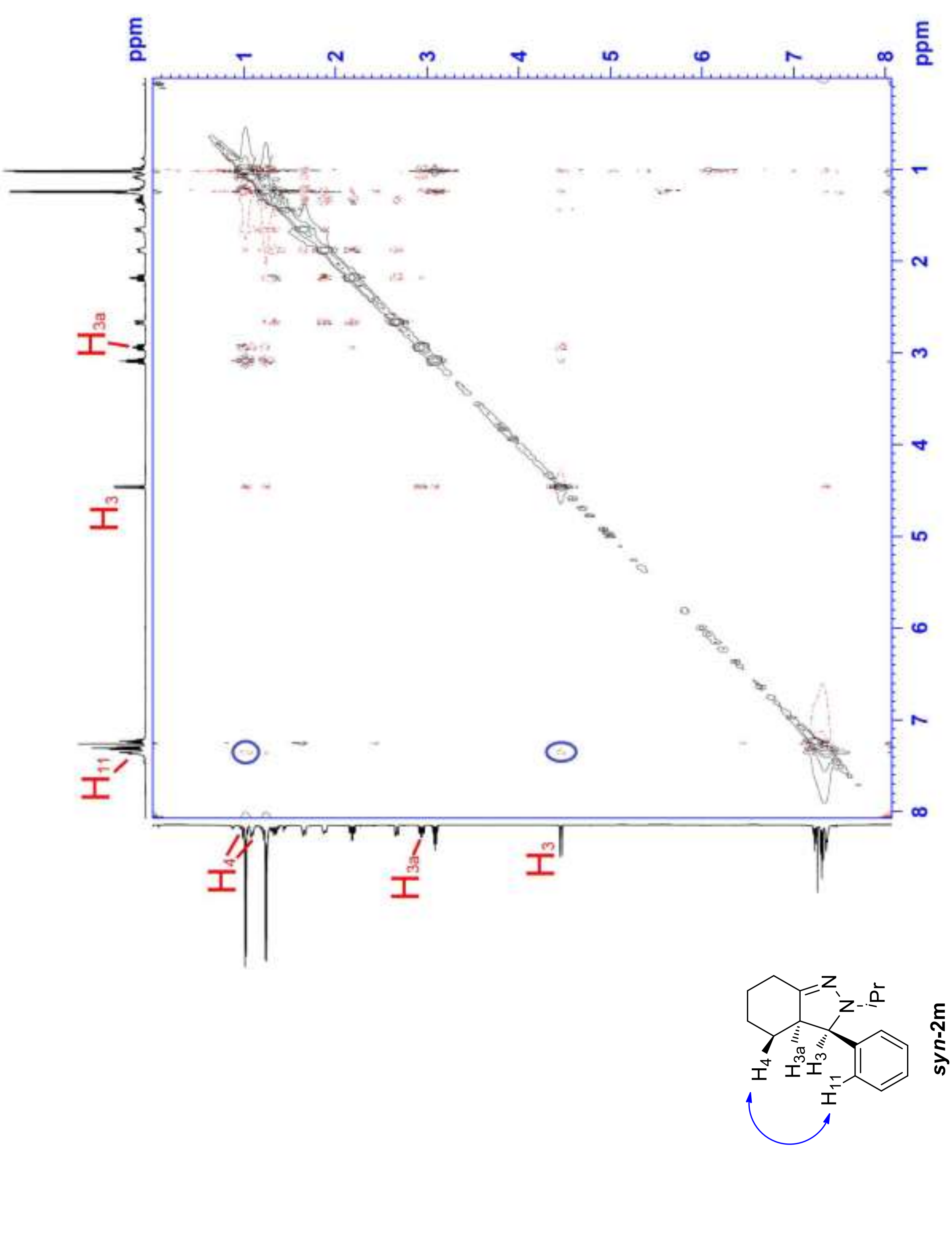

H11
H3
H3a
H4
ppm
N
iPr
syn-2m

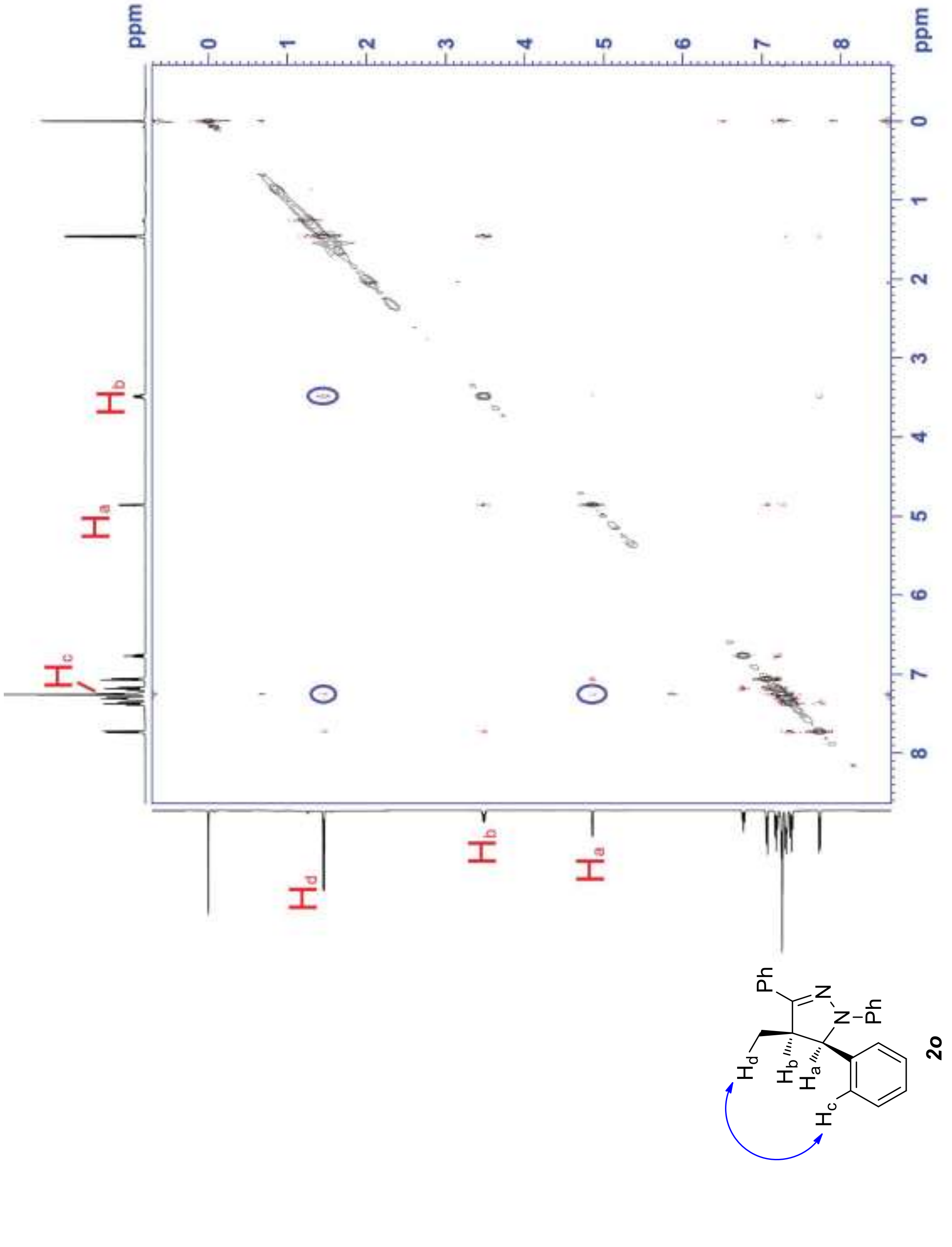
Hc
Hb
Ha
Hd
ppm
2o
Ph
N
Ph

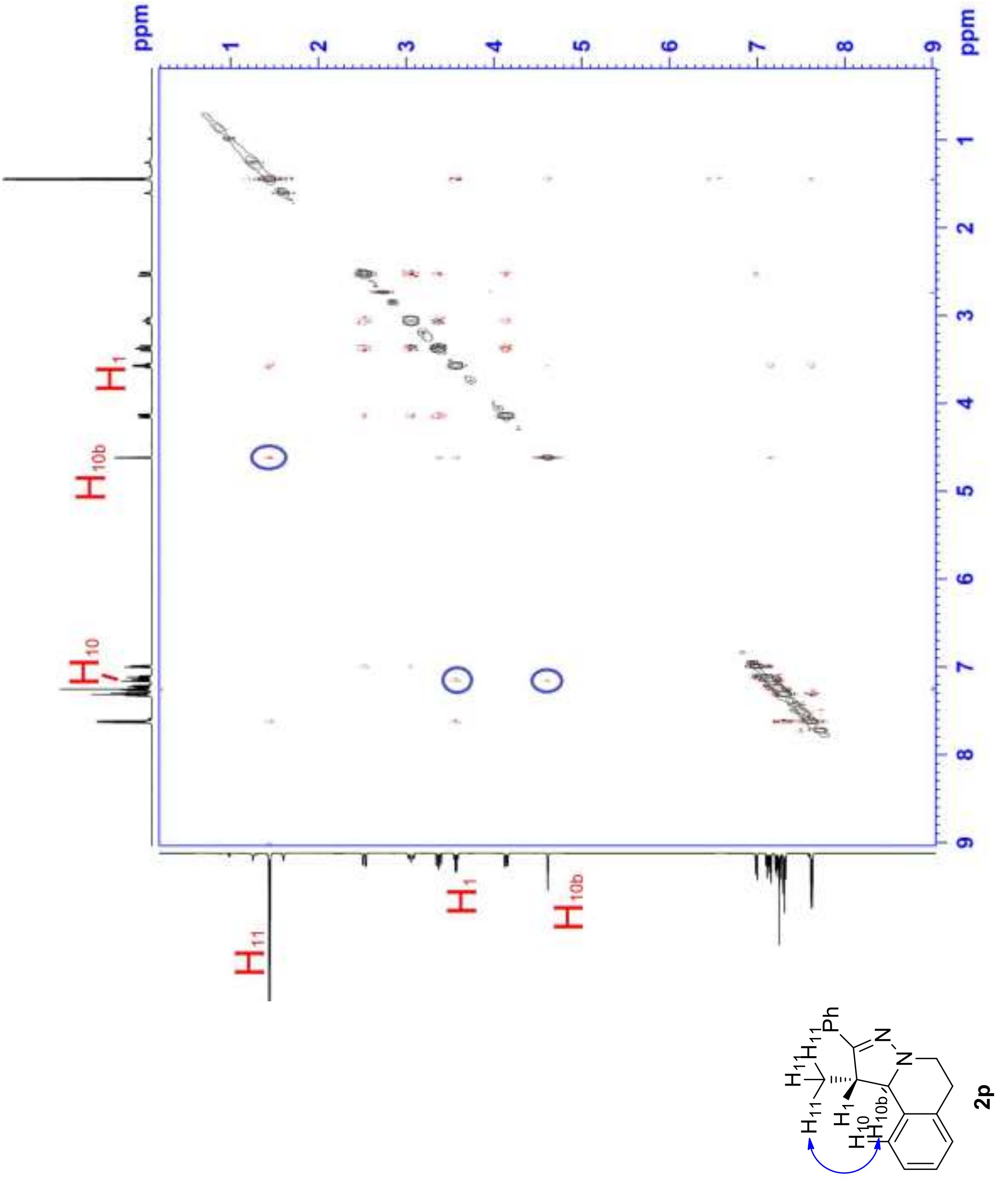
ppm
H1
H10b
H10
H11
Ph
2p

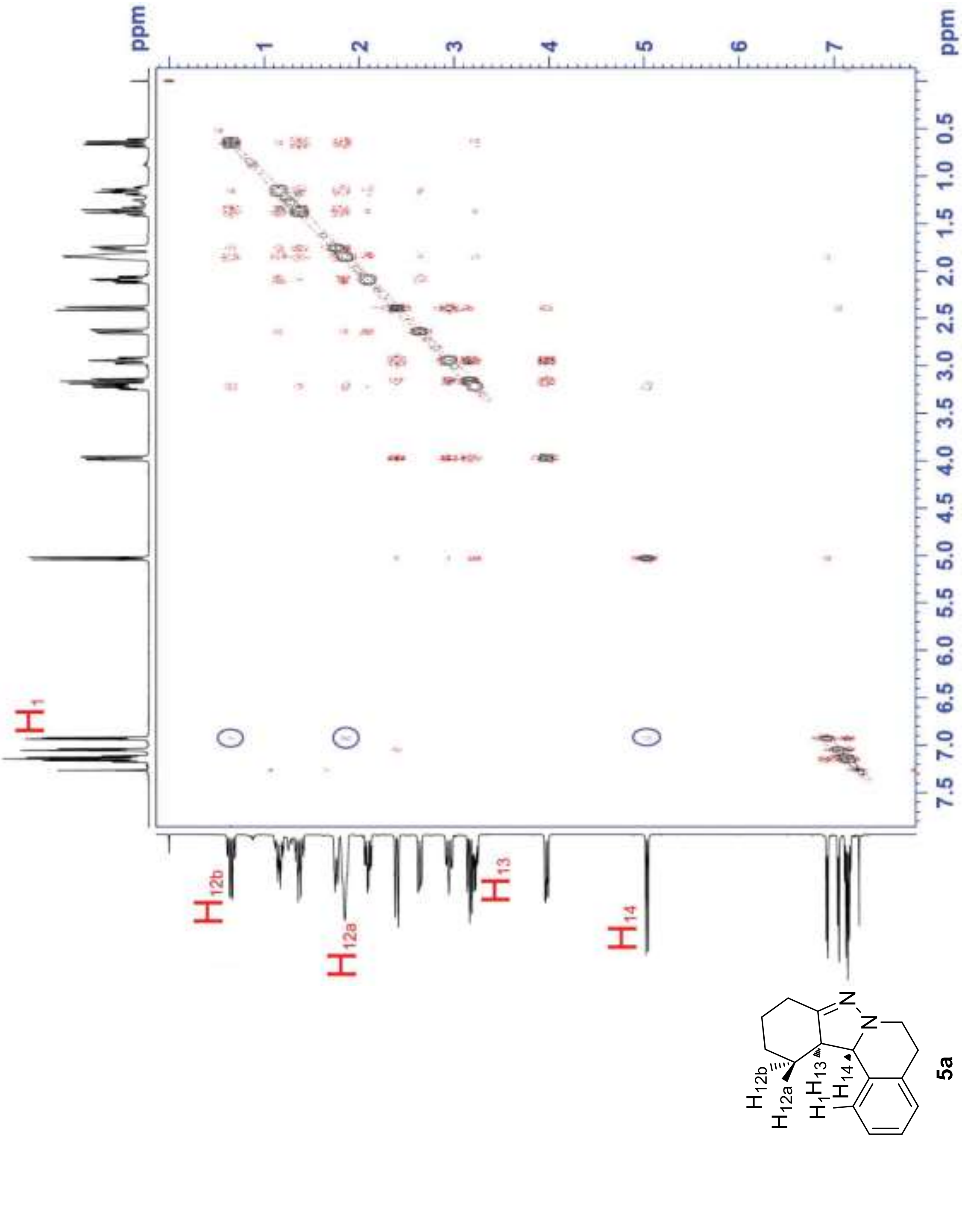

ppm
H1
H12b
H12a
H13
H14
5a

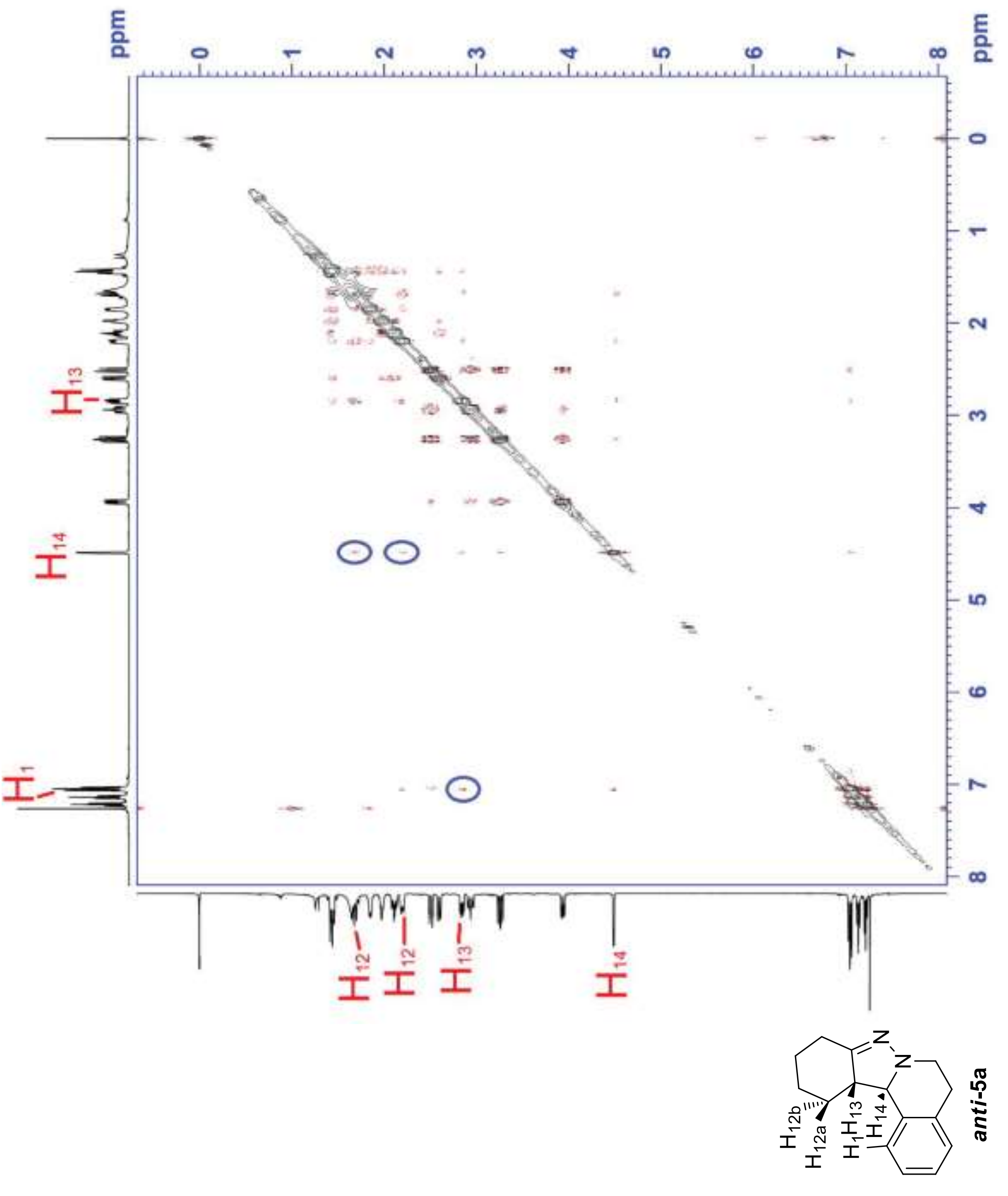
ppm
ppm
H1
H13
H14
H12
H12
H13
H14
H12b
H12a
H1
H13
H14
N
N
anti-5a

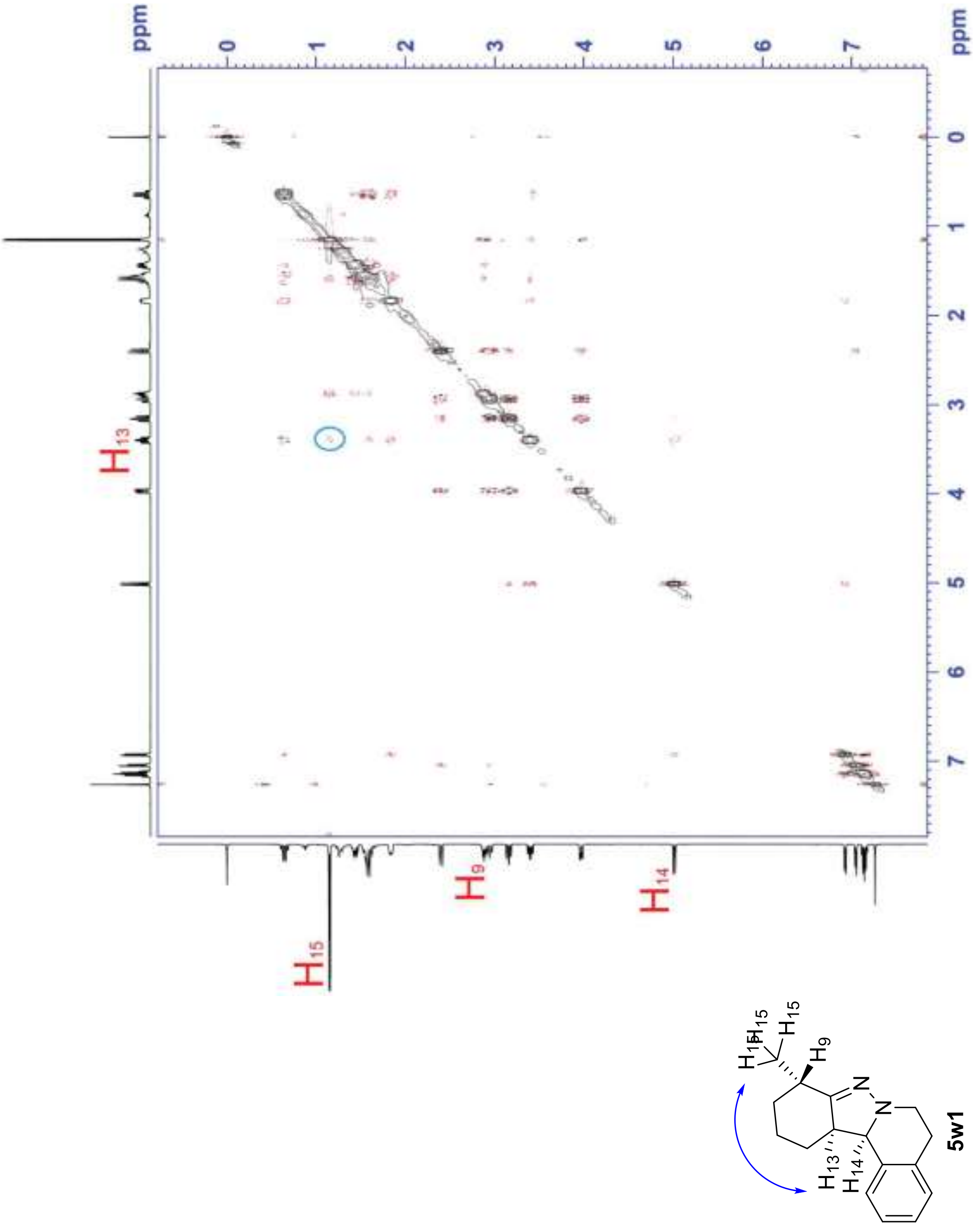

ppm
H13
H9
H14
H15
5w1

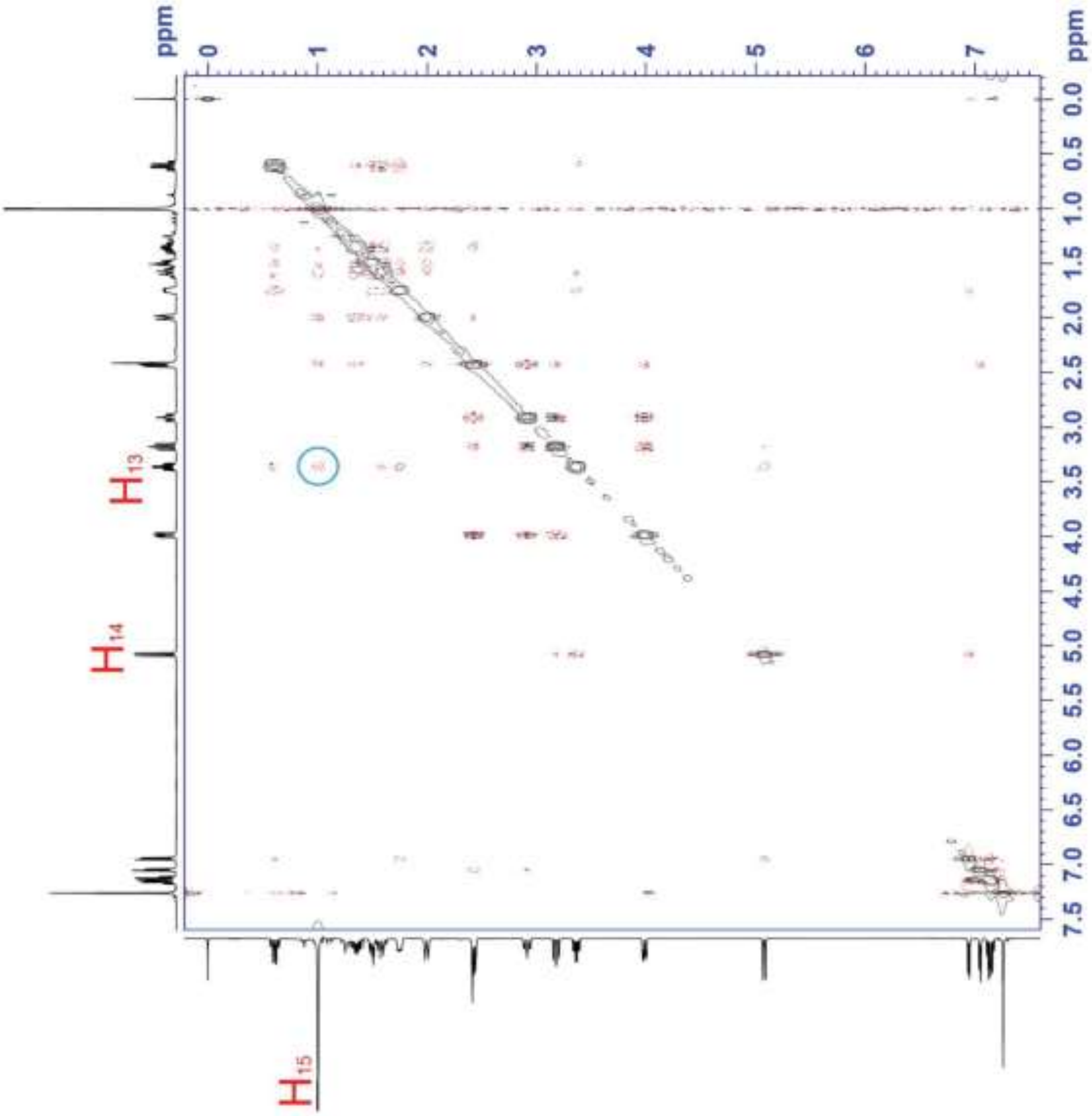

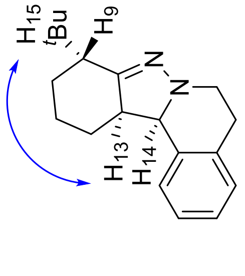

5y1

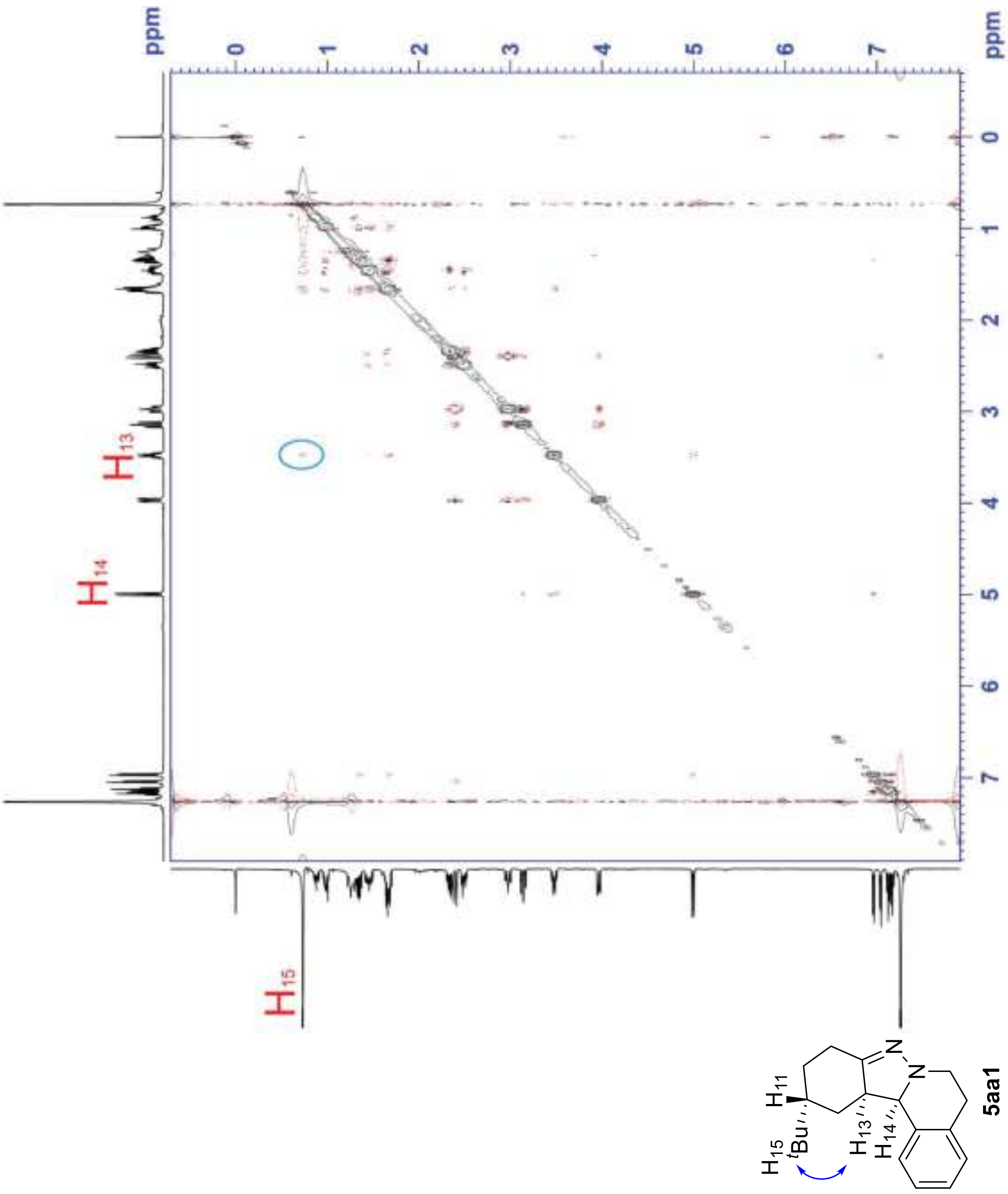
ppm
H13
H14
H15
5aa1

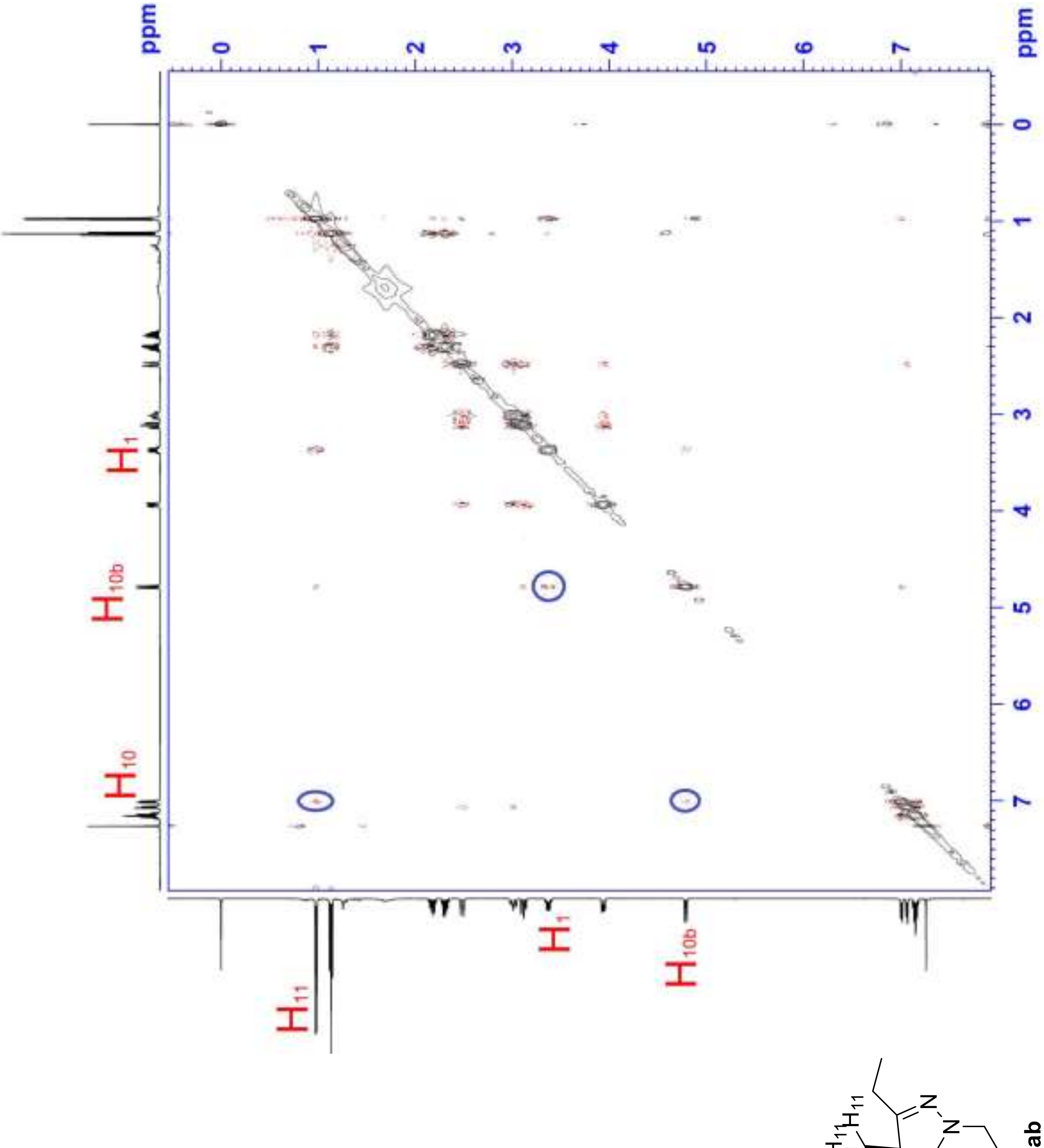

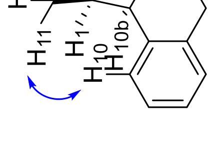

***syn*-5ab**

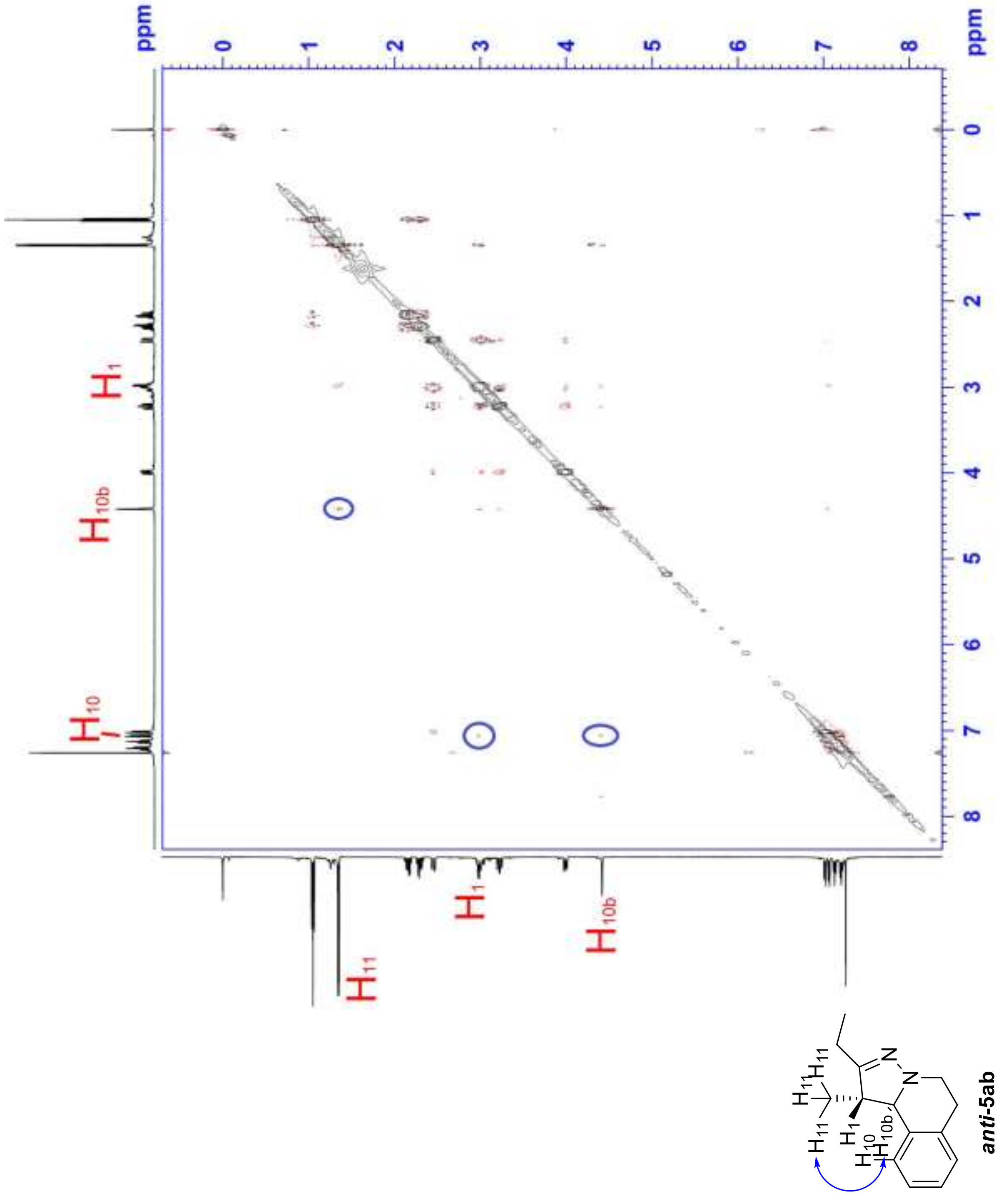

***anti*-5ab**
